# Identification of potential candidate genes and pathways in atrioventricular nodal reentry tachycardia by whole‐exome sequencing

**DOI:** 10.1002/ctm2.25

**Published:** 2020-04-30

**Authors:** Rong Luo, Chenqing Zheng, Hao Yang, Xuepin Chen, Panpan Jiang, Xiushan Wu, Zhenglin Yang, Xia Shen, Xiaoping Li

**Affiliations:** ^1^ Institute of Geriatric Cardiovascular Disease Chengdu Medical College Chengdu People's Republic of China; ^2^ State Key Laboratory of Biocontrol School of Life Sciences Sun Yat‐sen University Guangzhou China; ^3^ Department of Cardiology Hospital of the University of Electronic Science and Technology of China and Sichuan Provincial People's Hospital Chengdu Sichuan China; ^4^ Shenzhen RealOmics (Biotech) Co., Ltd. Shenzhen China; ^5^ The Center of Heart Development College of Life Sciences Hunan Norma University Changsha China; ^6^ Centre for Global Health Research Usher Institute of Population Health Sciences and Informatics University of Edinburgh Edinburgh United Kingdom; ^7^ Department of Medical Epidemiology and Biostatistics Karolinska Institutet Stockholm Sweden

**Keywords:** atrioventricular nodal reentry tachycardia, whole‐exome sequencing, gene‐based collapsing analysis, neurotransmitter release cycles pathway, ion channels–related pathway, ion channel genes

## Abstract

**Background:**

Atrioventricular nodal reentry tachycardia (AVNRT) is the most common manifestation of paroxysmal supraventricular tachycardia (PSVT). Increasing data have indicated familial clustering and participation of genetic factors in AVNRT, and no pathogenic genes related to AVNRT have been reported.

**Methods:**

Whole‐exome sequencing (WES) was performed in 82 patients with AVNRT and 100 controls. Reference genes, genome‐wide association analysis, gene‐based collapsing, and pathway enrichment analysis were performed. A protein‐protein interaction (PPI) network was then established; WES database in the UK Biobank and one only genetic study of AVNRT in Denmark were used for external data validation.

**Results:**

Among 95 reference genes, 126 rare variants in 48 genes were identified in the cases (minor allele frequency < 0.001). Gene‐based collapsing analysis and pathway enrichment analysis revealed six functional pathways related to AVNRT as with neuronal system/neurotransmitter release cycles and ion channel/cardiac conduction among the top 30 enriched pathways, and then 36 candidate pathogenic genes were selected. By combining with PPI analysis, 10 candidate genes were identified, including *RYR2, NOS1, SCN1A, CFTR*, *EPHB4, ROBO1, PRKAG2, MMP2, ASPH*, and *ABCC8*. From the UK Biobank database, 18 genes from candidate genes including *SCN1A, PRKAG2, NOS1*, and *CFTR* had rare variants in arrhythmias, and the rare variants in *PIK3CB, GAD2*, and *HIP1R* were in patients with PSVT. Moreover, one rare variant of *RYR2* (c.4652A > G, p.Asn1551Ser) in our study was also detected in the Danish study. Considering the gene functional roles and external data validation, the most likely candidate genes were *SCN1A, PRKAG2, RYR2, CFTR*, *NOS1, PIK3CB, GAD2*, and *HIP1R*.

**Conclusion:**

The preliminary results first revealed potential candidate genes such as *SCN1A, PRKAG2, RYR2, CFTR*, *NOS1, PIK3CB, GAD2*, and *HIP1R*, and the pathways mediated by these genes, including neuronal system/neurotransmitter release cycles or ion channels/cardiac conduction, might be involved in AVNRT.

## 1 BACKGROUND

Atrioventricular nodal reentrant tachycardia (AVNRT) is one of the most common types of paroxysmal supraventricular tachycardia (PSVT), which caused by a reentry circuit involving fast and slow atrioventricular nodal pathways.^1^ Although radiofrequency ablation has a satisfactory success rate in AVNRT, the precise anatomic structures that constitute the reentrant circuit are unresolved, and the specific pathogenesis has remained the subject of study over several decades.^2^, ^3^ Because most patients with AVNRT experience the onset of their symptoms in early adulthood and lack other structural heart disease, AVNRT was once believed a congenital functional abnormality developed during cardiac development.^2^

However, there are some reports of AVNRT occurring in twins and members in the same family,^4^, ^5^, ^6^, ^7^ and first‐degree relatives of patients with AVNRT present a hazard ratio of at least 3.6 for manifesting AVNRT compared with the general population,^7^ indicating that genetic factors are involved in the etiology and mechanism of this disease. Familial Wolff‐Parkinson‐White syndrome, another type of PSVT, has been well recognized as a disease that is partly caused by gene mutations, and a couple of responsible mutations in the *PRKAG2* gene have been confirmed.^8^, ^9^ However, little is known on the potential hereditary contribution to AVNRT, and no related pathogenic genes have been reported to date.

Exome sequencing is an efficient approach to identify pathogenic genes involved in Mendelian and/or non‐Mendelian hereditary diseases. However, factors such as lack of large multiplex families, locus heterogeneity, and incomplete penetrance have hampered such efforts to identify pathogenic genes in many diseases. Recent advances in gene‐based collapsing analysis might overcome some of these limitations.^10^ In addition, rather than investigating associations between single genetic variant and a phenotype, pathway analysis of exome sequencing data interrogates alterations in biological pathways and helps us identify the underlying genes that cause disease. Therefore, we hypothesize that the application of this more integrated approach may help elucidate the genetic etiology of AVNRT.

To our knowledge, there are no published studies identifying the pathogenic genes in AVNRT. In the current pilot study, we examined AVNRT using whole‐exome sequencing (WES) to verify possible pathogenic genes by gene‐base burden, pathway enrichment, and protein‐protein interaction (PPI) analyses.

## 2 SUBJECTS AND METHODS

The study participators were identified among patients treated with radiofrequency catheter ablation at the Department of Cardiology of the Sichuan Academy of Medical Sciences and the Sichuan Provincial People's Hospital in the period from 2014 to 2017. A total of 100 unrelated ethnically matched healthy control subjects were enlisted from the visitors to the Health Evaluation and Promotion Center of the Sichuan Academy of Medical Sciences and the Sichuan Provincial People's Hospital. Upon inclusion, blood sample tests, 12 lead electrocardiograms, echocardiography, and cardiac history were recorded. The control subjects were free of any cardiovascular diseases, arrhythmia, chronic anemia, diabetes mellitus, thyroid disorders, electrolyte disturbance, systemic immune diseases, malignant tumors, or any other diseases known to cause arrhythmias. A written informed consent for genetic screening was obtained from all participants. Ethical approval for this study was acquired from the ethics committee of the Sichuan Academy of Medical Sciences and the Sichuan Provincial People's Hospital.

### 2.1 Intracardiac electrophysiological study

Baseline intracardiac electrophysiological studies included atrial stimulation (burst or extra stimulus pacing) and ventricular stimulation in cases. AVNRT diagnosis was set up according to published criteria and pacing maneuvers as applicable.^11^ Dual atrial ventricular (AV) node physiology was defined as a ≥50‐ms increment in the atrial‐His (AH) interval after a 10‐ms decrement interval during single‐atrial extra stimulation or a ≥50‐ms increment in the AH interval after shortening the pacing cycle length by 10 ms. If persistent AVNRT (lasting ≥30 seconds) was not induced, the same pacing maneuvers were repeated under isoproterenol administration and withdrawal as previously described.^12^

### 2.2 Next‐generation DNA sequencing, variant calling, and annotation

DNA samples were extracted from peripheral blood using the QIAamp DNA Blood Mini and Maxi Kits (Qiagen, Hilden, Germany) according to the manufacturer's instructions. Entire exon sequences were enriched by using a SureSelect Human All Exon kit V6 (Agilent Technologies, Santa Clara, CA, USA), and the libraries were sequenced on the Illumina HiSeq NovaSeq platform (Illumina, San Diego, CA, USA). The average read depth was 123, and on average 96.4, 98.6, and 99.4% of exons were covered by at least 20 reads, 10 reads, and 4 reads, respectively (Supplement Figure 1, S14). Qualified sequence reads were arrayed to the human reference genome (NCBI GRCh37) using the Burrows‐Wheeler Aligner(version 0.5.17; http://bio-bwa.sourceforge.net/). SAMtools (version 0.1.18, http://samtools.sourceforge.net/), Picard (http://picard.sourceforge.net/), and GATK (http://www.broadinstitute.org/gsa/wiki/index.php/Home_Page) were used for removing duplicated reads, realignment, and recalibration. Potential single nucleotide variants (SNVs) and small insertions and deletions (indel) were called and filtered by using GATK3.7. Then, high‐confidence SNV and indel variants were noted using snpEff (Version 4.2; http://snpeff.sourceforge.net/). Furthermore, all variants were annotated according to the control population of the 1000 Genomes Project (2014 Oct release, http://www.1000genomes.org), ExAC (http://exac.broadinstitute.org), EVS (http://evs.gs.washington.edu/EVS), the disease databases of ClinVar (http://www.ncbi.nlm.nih.gov/clinvar), and OMIM (http://www.omim.org).

### 2.3 Rare variants in reference genes

A total of 95 reference AVNRT genes were selected to detect rare variants in AVNRT cases and controls. The genes were elected based on the following criteria according to another pioneering study on gene rare variants in AVNRT^13^: (1) genes involved in PR interval in electrocardiogram identified by genome‐wide association studies,^14^ (2) genes selected based on expression levels in human atrioventricular conduction axis,^15^ (3) plausible genes based on protein function, and association with other cardiac diseases, especially arrhythmic diseases.^13^, ^16^, ^17^, ^18^ Selected genes are listed in Supplementary Table S1.

### 2.4 Single‐marker association analysis

We used GATK v3.7 CombineGVCFs to combine the WES dataset with ethnically matched and unrelated subjects in the AVNRT cohort and the control group, followed by filtering with VQSR and PLINK1.9 (–geno 0.1 –hwe 0.0001) to obtain high‐confidence variant datasets.^19^ Furthermore, PLINK1.9 was applied to check the multidimensional scaling dataset based on raw Hamming distances for population stratification, identity by descent calculation for sample pairs, and Hardy‐Weinberg equilibrium deviation for all markers. Genome‐wide association analysis (GWAS) for the qualified high‐confidence datasets was performed to compute the odds ratios (ORs) and *P* values in PLINK using *Fisher*’s exact test for dichotomous phenotypes (cases vs controls for AVNRT). Finally, we used a genome‐wide threshold for significance of *P* < 1 × 10^−6^. A quantile‐quantile (Q‐Q) plot was used to evaluate the resulting *P* values.

### 2.5 Gene‐based collapsing analysis and pathway enrichment

We performed gene‐based collapsing to combine the information on multiple deleterious rare variants into a single value per gene, with ethnically matched and unrelated subjects in the AVNRT cohort (*n* = 82) and the control group (*n* = 100). We defined deleterious rare (minor allele frequency [MAF] < 0.01) variants as nonsense, missense, splice‐site, indel, and frameshift mutations. For statistical considerations, Fisher's exact test methods were preferred to calculate the gene‐based collapsing. Two groups with MAFs below 0.1% and 1% in the Exome Aggregation Consortium (ExAC) and 1000 Genomes Project databases were calculated separately. A statistical significance was determined by *P *< 0.05. The significant genes were submitted to the KOBAS3.0 web server (http://kobas.cbi.pku.edu.cn/kobas3) to obtain the functional gene set Reactome Pathway enrichment. Then, the rich factor was calculated, and the top 30 enriched pathways are shown based on the corrected*P* value.

### 2.6 Construction of the PPI network

The Search Tool for the Retrieval of Interacting Genes database (STRING) (Version 10.0, http://string-db.org) was used to predict the relationships among the screened genes and identify the most relevant genes.^20^ Based on experimental data, database entries, and coexpression, PPI node pairs with a score of combination > 0.4 (medium confidence) were considered to be significant. Then, Cytoscape software (version 3.7.1) was used to visualize the resulting PPI network.

## 3 RESULTS

### 3.1 Clinical data of the cases

Our analysis included WES data from 82 cases and 100 controls. All AVNRT patients were diagnosed by electrophysiologic examination and underwent radiofrequency ablation. Among the 82 cases recruited in our analyses, the mean age at onset was 54.1 ± 17.1 years old, and the ratio of females/males was 2.28:1. The median disease course was 4.0 years. Five patients had a history of syncope or approximate syncope, three had a familial history or suspected familial history of AVNRT, and no patients exhibited any structural heart disease. In the electrophysiological study, nine patients presented no AH jump, isoproterenol infusion was used in six cases to induce the onset of AVNRT. Except for one patient who exhibited slow‐slow and one with slow‐fast, all other cases exhibited typical slow‐fast AVNRT, and all cases were treated successfully with radiofrequency ablation during the operation, with only one case relapsing in 6 months after operation; for more details, see Table 1.

**TABLE 1:** Demographic baseline of patients

Variables	Total patients (n = 82)
Sex, male (%)	25 (30.5)
Age at onset, year	44.1 ± 17.1
BMI, kg/m^2^	23.8 (22.4‐25.9)
Disease course, year	6.7 ± 8.4
Synicope/approximate syncope, *n* (%)	5 (6.0)
Chest distress, *n* (%)	10 (12.2)
Familial history, *n* (%)	3 (3.7)
Heart rate at onset, bpm	172.7 ± 20.5
Atypical of AVNRT, *n* (%)	2 (2.4)
Use of isoproterenol during operation, *n* (%)	6 (7.3)
Cases without AH jump, *n* (%)	9 (11.0)
Antegrade Wenckebach's point of atrioventricular node (ms)	332.8 ± 58.3

### 3.2 Rare variants in reference genes

**FIGURE 1:**
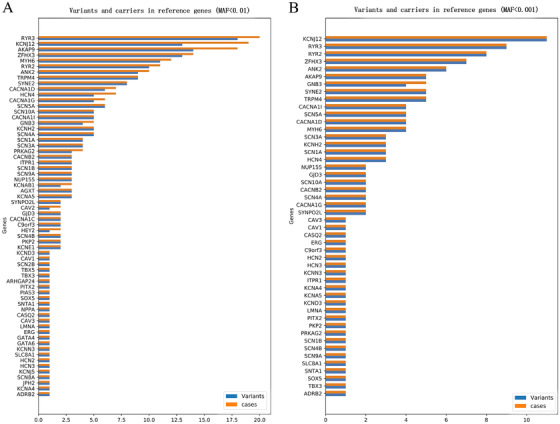
The number of rare variants and cases in referential genes (A, MAF < 0.01; B, MAF < 0.001). The blue box represented the number of the rare variants of the referential gene, and the red box represented the numbers of the patients who carried the rare variants

Among the 95 reference genes, 126 deleterious rare variants in 48 genes were detected according to the definition of rare variants with an MAF < 0.001 in the ExAC and 1000 Genomes Project databases: 11 rare variants in *KCNJ12* (*n* = 11), nine in *RYR3* (*n* = 9), eight in *RYR2* (*n* = 8), seven in *ZFHX3* (*n* = 7), six in *ANK2* (*n* = 6); five in *AKAP9* (n = 5), *SYNE2* (*n* = 5), *TRPM4* (n = 5); four in *CACNA1D* (*n* = 4), *CACNA1I* (*n* = 4), *GNB3* (*n* = 5), *MYH6* (*n* = 4), *SCN5A* (*n* = 4); three in *HCN4* (*n* = 3), *KCNH2* (*n* = 3), *SCN1A* (*n* = 3), *SCN3A* (*n* = 3); two in *CACNA1G* (*n* = 2), *CACNB2* (*n* = 2), *GJD3* (*n* = 2), *NUP155* (*n* = 2), *SCN4A* (*n* = 2), *SCN10A* (*n* = 2), *SYNP02L* (*n* = 2); one rare variant in one case in following genes: *ADRB2, C9orf3, CASQ2, CAV1, CAV3, ERG, HCN2, HCN3, ITPR1, KCNA4, KCNA5, KCND3, KCNN3, LMNA, PITX2, PKP2, PRKAG2, SCN1B, SCN4B, SCN9A, SLC8A1, SNTA1, SOX5*, and *TBX3* (Supplementary Table S2; Figure 1). Among the above rare variants in the listed genes, only two controls exhibited two rare variants in *KCNJ12* and one rare variant was found in one control subject in each of *HCN4*, *ANK2*, and *RYR2*.

As PSVT has a prevalence of 22.5/10 000 persons and an incidence of 35/100 000 person‐years,^21^ and the sample examined in the current study was relatively small, we chose another definition of rare variants with an MAF < 0.01 in the ExAC and 1000 Genomes Project databases, and a total of 227 rare variants in 64 genes were detected. The details of the rare variants are presented in Supplementary Table S3.

### 3.3 GWAS study for common variants

**FIGURE 2:**
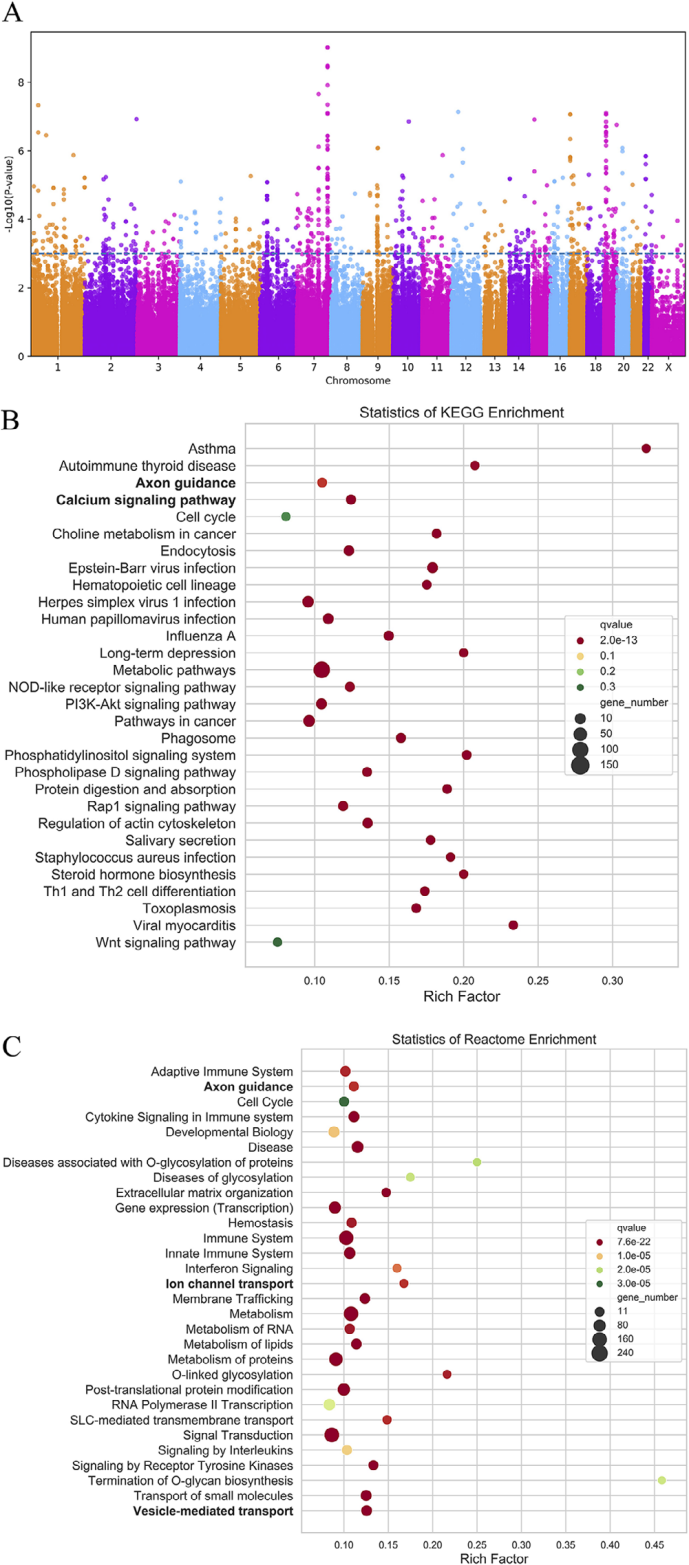
Manhattan plot (A) and pathway enrichment analysis of KEGG (B) and Reactome (C). A, The Manhattan plot showed the significant locus along the genome (*P *< 10^−6^); B, The bubble chart of top30 pathways enriched by KEGG database; C, The bubble chart of top 30 pathways enriched by Reactome database

Single‐nucleotide polymorphisms (SNPs) were removed from the preimputation dataset if they exhibited an MAF < 0.01 or a *P* value for Hardy‐Weinberg equilibrium < 1 × 10^−4^ (Supplementary Figure 2, S15). Association *P* values from the GWAS were reported in Q‐Q plots and Manhattan plots (Figure 2, Supplementary Figure 2, S15). In the limited number of samples, SNPs with *P* values (*Fish* test) of less than 10^−6^ are shown in Supplementary Table S4.

Then, pathway enrichment was performed under the condition of including SNPs with *P *< 0.01 according to *Kyoto Encyclopedia of Genes and Genomes* (KEGG) and Reactome databases. As shown in Figure 2, the following four related traits were among the top 30 pathways in the two databases: (1) vesicle‐mediated transport, (2) axon guidance, (3) the Ca^2+^ signaling pathway, and (4) ion channel transport, and there were 20 genes in both the KEGG and Reactome database analyses, including *ABLIM2, ASPH, ATP2B4, CACNA1G, DPYSL2, EPHA2, FES, MYL12A, NE01, PLXNA4, PLXNB1, PLXNC1, ROBO2, RYR1, RYR2, RYR3, SEMA5A, SEMA6D, SLIT3*, and *UNC5B* (Table 2).

**TABLE 2:** SNPs in genes from the pathway enrichment analysis according to both KEGG and Reactome

Genes	A1	F_A	F_U	A2	*P*	OR	Functions	Hgv.c	Hgv.p
SEMA6D	G	0.414	0.225	T	1.10E−4	2.44	Intron variant	c.1646+105G > T	.
ROBO2	G	0.207	0.075	C	3.18E−4	3.23	Intron variant	c.109+121G > C	.
MYL12A	C	0.161	0.041	CGT	3.48E−4	4.51	Intron variant	c.199+192_199+193delGT	.
ABLIM2	T	0.177	0.345	C	3.49E−4	0.41	Intron variant	c.838‐131G > A	.
ATP2B2	G	0.000	0.060	A	7.05E−4	0.00	Intron variant	c.397+71C > T	.
ATP2B2	A	0.110	0.025	G	1.02E−3	4.81	Synonymous variant	c.1626C > T	p.Ile542Ile
ATP2B2	T	0.110	0.025	C	1.02E−3	4.81	Intron variant	c.1659+102G > A	.
UNC5B	T	0.120	0.027	C	1.05E−3	4.89	Intron variant	c.305‐277C > T	.
NEO1	C	0.342	0.195	T	1.80E−3	2.14	Intron variant	c.1511+90T > C	.
NEO1	TA	0.348	0.200	T	1.90E−3	2.13	3‐prime UTR variant	c.*52_*53insA	.
ATP2B2	T	0.183	0.325	G	2.62E−3	0.47	Intron variant	c.1417‐186C > A	.
NEO1	G	0.342	0.200	A	2.77E−3	2.07	Synonymous variant	c.1779A > G	p.Lys593Lys
ASPH	C	0.518	0.359	A	2.80E−3	1.93	Intron variant	c.1108‐121T > G	.
ABLIM2	A	0.232	0.380	G	3.07E−3	0.49	Intron variant	c.915+26C > T	.
PLXNB1	A	0.043	0.000	G	3.51E−3		Non‐coding transcript exon variant	n.5120C > T	.
DPYSL2	T	0.108	0.028	C	3.56E−3	4.22	Intron variant	c.936+251C > T	.
PLXNC1	T	0.043	0.000	C	3.65E−3		Intron variant	c.1062+161C > T	.
CACNA1G	CTGTGTGTGTGTTTGTG	0.049	0.140	C	4.41E−3	0.32	Intron variant	c.5782‐165_5782‐164insTGTGTGTGTGTTTGTG	.
ASPH	C	0.488	0.340	G	5.26E−3	1.85	Intron variant	c.1346‐79C > G	.
UNC5B	T	0.134	0.050	C	5.31E−3	2.94	Splice region variant	c.732C > T	p.Tyr244Tyr
UNC5B	A	0.134	0.050	G	5.31E−3	2.94	Intron variant	c.734‐173G > A	.
UNC5B	G	0.134	0.050	A	5.31E−3	2.94	Missense variant	c.724A > G	p.Ile242Val
UNC5B	CTG	0.134	0.050	C	5.31E−3	2.94	Intron variant	c.1100‐35_1100‐34insTG	.
UNC5B	T	0.134	0.050	C	5.31E−3	2.94	Intron variant	c.901+33C > T	.
ASPH	T	0.512	0.365	C	5.67E−3	1.83	Intron variant	c.1195‐57G > A	.
UNC5B	A	0.128	0.045	G	6.47E−3	3.12	Intron variant	c.80‐87G > A	.
EPHA2	CAG	0.041	0.000	C	6.82E−3		Intron variant	c.86‐344_86‐343dupCT	.
RYR3	C	0.073	0.015	G	6.92E−3	5.18	Synonymous variant	c.2403G > C	p.Leu801Leu
RYR3	G	0.073	0.015	A	6.92E−3	5.18	Intron variant	c.3556+34A > G	.
RYR2	A	0.061	0.010	G	7.78E−3	6.43	Intron variant	c.13317+48G > A	.
RYR3	C	0.370	0.240	G	7.95E−3	1.86	Intron variant	c.5861‐174C > G	.
PLXNA4	CACACACAAACAT	0.024	0.095	C	8.05E−3	0.24	Intron variant	c.3874+275_3874+276insATGTTTGTGTGT	.
SEMA5A	C	0.352	0.223	T	8.65E−3	1.89	Intron variant	c.1599+327G > A	.
RYR1	G	0.012	0.070	A	8.74E−3	0.17	Intron variant	c.11689+68A > G	.
SLIT3	G	0.253	0.383	A	9.35E−3	0.55	Intron variant	c.1459+4296C > T	.
RYR2	G	0.438	0.299	A	9.66E−3	1.82	Intron variant	c.9128+133A > G	.
FES	T	0.037	0.11	C	9.71E−3	0.31	Non coding transcript exon variant	n.616T > C	.
FES	A	0.037	0.11	G	9.71E−3	0.31	Intron variant	c.388‐212A > G	.

*Notes*: F_A, frequency of the affected; F_U, frequency of the unaffected; hgv.c, human genome variation c.DNA; hgv.p, human genome variation protein.

### 3.4 Gene‐based collapsing analysis and pathway enrichment for rare variants

We carried out gene‐based collapsing tests under two frequency categories (MAF < 0.01 and MAF < 0.001) with *P* values of less than 0.05 were included. The Q‐Q plots, Manhattan figures, and rare variants of the genes are shown in Supplemental Figure‐3 S16 and Supplemental Tables S5 and S6.

**FIGURE 3:**
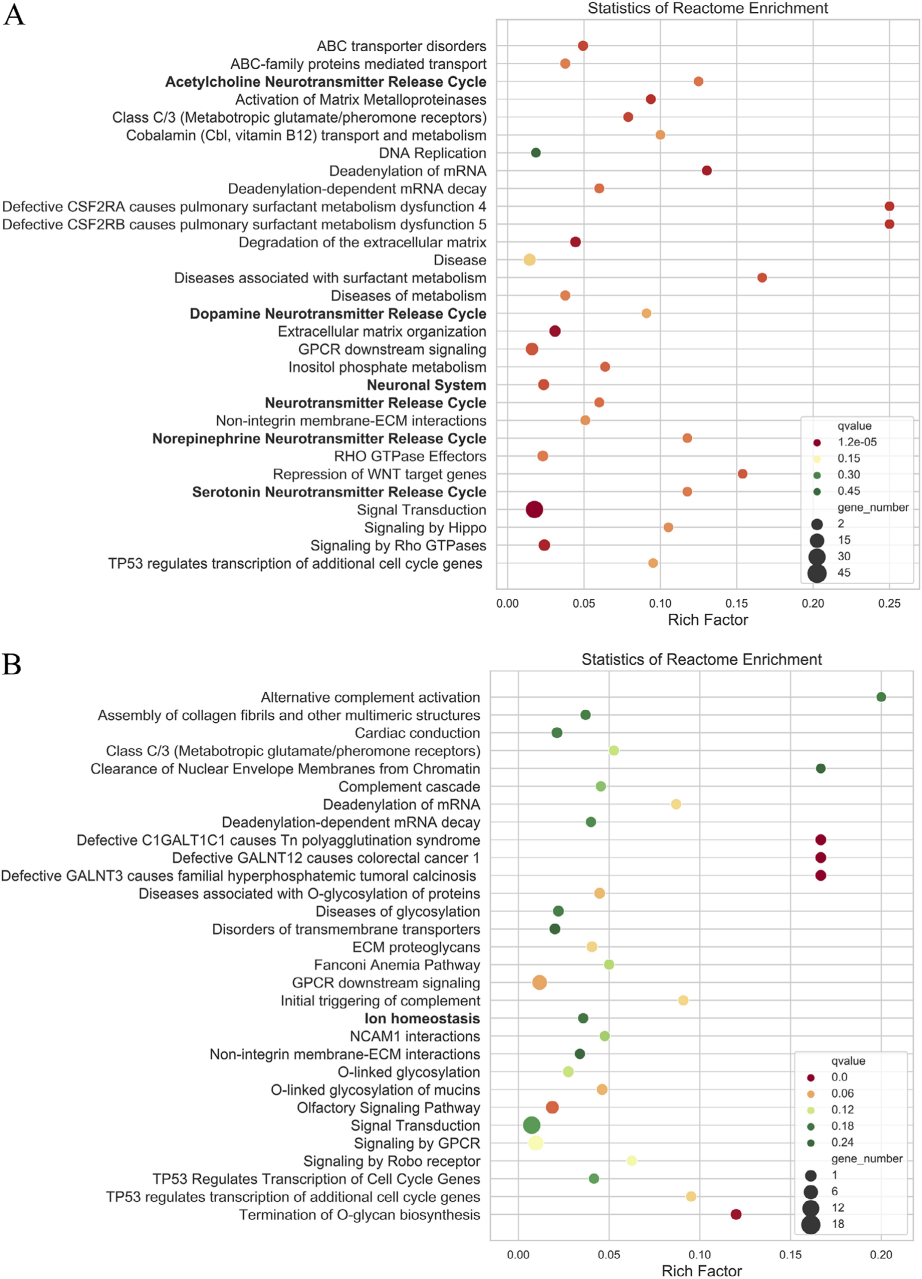
The top 30 pathways in Reactome pathway enrichment. A, The bubble chart of top30 pathways enriched by Reactome database (MAF < 0.001); B, The bubble chart of top30 pathways enriched by Reactome database (MAF < 0.001)

In pathway analysis, rare variants are associated with genes, and genes are placed into sets. The pathway enrichment analysis was performed using the Reactome database, and there were 517 and 343 pathways enriched with an MAF < 0.01 and MAF < 0.001, respectively (Supplementary Tables S7 and S8). Among the top 30 enriched pathways, there were six related pathways (MAF < 0.01) and two pathways related to AVNRT (MAF *P *< 0.001) (Figure 3). In addition, 14 pathways other than the top 30 pathways exhibited potential functions associated with AVNRT with either an MAF < 0.01 or MAF < 0.001 (Tables 3 and 4).

**TABLE 3:** Gene‐based pathway enrichment according to Reactome (MAF < 0.01)

Pathways	ID	Input number	Background number	*P*‐Value	Corrected *P*‐Value	Genes
Neuronal system	R‐HSA‐112316	8	339	0.004	0.048	A*BCC8, PPFIA1, LRFN4, TSPOAP1, BEGAIN, GAD2, SYT10, KCNV2*
Neurotransmitter release cycle	R‐HSA‐112310	3	50	0.007	0.064	*TSPOAP1, PPFIA1, GAD2*
Acetylcholine neurotransmitter release cycle	R‐HSA‐264642	2	16	0.008	0.068	*TSPOAP1, PPFIA1*
Serotonin neurotransmitter release cycle	R‐HSA‐181429	2	17	0.008	0.072	*TSPOAP1, PPFIA1*
Norepinephrine neurotransmitter release cycle	R‐HSA‐181430	2	17	0.008	0.072	*TSPOAP1, PPFIA1*
Dopamine neurotransmitter release cycle	R‐HSA‐212676	2	22	0.013	0.096	*TSPOAP1, PPFIA1*
Glutamate neurotransmitter release cycle	R‐HSA‐210500	2	23	0.014	0.100	*TSPOAP1, PPFIA1*
Axon guidance	R‐HSA‐422475	9	549	0.023	0.133	*EPHB4, LAMC1, MMP2, DOK4, CSF2RB, SCN1A, EVL, ROBO1, PIK3CB*
Cation‐coupled chloride cotransporters	R‐HSA‐426117	1	7	0.057	0.197	*SLC12A4*
Interactions of neurexins and neuroligins at synapses	R‐HSA‐6794361	2	57	0.070	0.212	*BEGAIN, SYT10*
Protein‐protein interactions at synapses	R‐HSA‐6794362	2	57	0.070	0.212	*BEGAIN, SYT10*
SLAM protein interactions at the synapses	R‐HSA‐8849932	1	21	0.149	0.284	*LRFN4*
Potassium channels	R‐HSA‐1296071	2	99	0.169	0.300	*ABCC8, KCNV2*
Metal ion SLC transporters	R‐HSA‐425410	1	25	0.174	0.304	*HEPH*
Transmission across chemical synapses	R‐HSA‐112315	3	208	0.201	0.329	*TSPOAP1, PPFIA1, GAD2*
Inwardly rectifying K^+^ channels	R‐HSA‐1296065	1	31	0.209	0.334	*ABCC8*
Vesicle‐mediated transport	R‐HSA‐5653656	6	573	0.251	0.369	*AP1G2, CD163, CFTR, PRKAG2, HIP1R,COG4*
Voltage gated potassium channels	R‐HSA‐1296072	1	43	0.276	0.389	*KCNV2*
Cardiac conduction	R‐HSA‐5576891	2	141	0.281	0.393	*SCN1A, NOS1*
Ion homeostasis	R‐HSA‐5578775	1	56	0.342	0.439	*NOS1*
Ca^2+^ pathway	R‐HSA‐4086398	1	61	0.366	0.456	*TCF7L1*

SLAM, signaling lymphocytic activation; SLC, solute carrier.

**TABLE 4:** Gene‐based pathway enrichment according to Reactome (MAF < 0.001)

Pathways	ID	Input number	Background number	*P*‐Value	Corrected *P*‐Value	Genes
Cardiac conduction	R‐HSA‐5576891	3	141	0.0246	0.177	*HIPK2, NOS1, ASPH*
Ion homeostasis	R‐HSA‐5578775	2	56	0.026	0.179	*NOS1, ASPH*
Axon guidance	R‐HSA‐422475	6	549	0.034	0.191	*COL4A3, EPHB4, PSMB11, COL5A1, EVL, ROBO1*
Ion channel transport	R‐HSA‐983712	3	211	0.066	0.229	*SLC9B1, ATP2C2, ASPH*
SLAM protein interactions at the synapses	R‐HSA‐8849932	1	21	0.091	0.261	*LRFN4*
Ion transport by P‐type ATPases	R‐HSA‐936837	1	57	0.222	0.379	*ATP2C2*
Transport of inorganic cations/anions and amino acids/oligopeptides	R‐HSA‐425393	1	100	0.354	0.486	*SLC26A4*
Neuronal System	R‐HSA‐112316	1	339	0.771	0.800	*LRFN4*
Vesicle‐mediated transport	R‐HSA‐5653656	1	573	0.918	0.9236	*CD163*

SLAM, signaling lymphocytic activation.

From the above‐related pathways, 36 candidate pathogenic genes were selected: *ABCC8, AP1G2, ASPH, ATP2C2, BEGAIN, CD163, CFTR, COG4, COL5A1, COL4A3, CSF2RB, DOK4, EPHB4, EVL, GAD2, HEPH, HIPK2, HIP1R, KCNV2, LAMC1, LRFN4, MMP2, NOS1, PIK3CB, PPFIA1, PRKAG2, PSMB11, ROBO1, SCN1A, SFTPA2, SLC9B1, SLC26A4, SLC12A4, SYT10, TCF7L1*, and *TSPOAP1* (Table 5). The variant information for these candidate genes is listed in Supplemental Tables S9 and S10. Among the candidate genes, *SCN1A* and *PRKAG2* were identified in arrhythmia diseases as reference genes.

**TABLE 5:** Gene‐based burden results for candidate genes

Gene	MAF < 0.01				MAF < 0.001			
	OR	*P* value	Cases	Controls	OR	*P* value	Cases	Controls
CFTR	4.67	4.546E−6	68	51	1.89	2.570E−1	6	4
EVL	NA*	4.352E−5	12	0	NA	4.352E−05	12	0
HIP1R	NA	3.256E−3	7	0	NA	2.016E−1	2	0
ABCC8	NA	7.539E−3	6	0	NA	8.961E−2	3	0
COG4	9.24	1.629E−2	7	1	NA	8.961E−2	3	0
LAMC1	9.24	1.629E−2	7	1	1.22	6.995E−1	1	1
AP1G2	9.24	1.629E−2	7	1	NA	4.505E−1	1	0
GAD2	NA	1.733E−2	5	0	NA	4.505E−1	1	0
CSF2RB	NA	1.733E−2	5	0	NA	2.016E−1	2	0
BEGAIN	NA	1.733E‐2	5	0	NA	8.961E−2	3	0
SYT10	NA	1.733E−2	5	0	NA	4.505E−1	1	0
LRFN4	NA	1.733E−2	5	0	NA	1.733E−2	5	0
NOS1	1.95	2.000E−2	42	35	1.93	2.337E−2	39	32
SLC12A4	5.	2.433E−2	8	2	3.18	1.489E−1	5	2
ROBO1	5.30	2.433E−2	8	2	9.24	1.628E−2	7	1
SFTPA2	3.26	2.447E−2	12	5	5.01	9.055E−3	11	3
TSPOAP1	3.99	3.123E−2	9	3	1.66	3.9110E−1	4	3
KCNV2	7.82	3.324E−2	6	1	2.48	4.257E−1	2	1
PIK3CB	7.82	3.324E−2	6	1	2.48	4.257E−1	2	1
CD163	NA	3.955E−2	4	0	NA	3.955E−2	4	0
PRKAG2	NA	3.955E−2	4	0	NA	4.505E−1	1	0
DOK4	NA	3.955E−2	4	0	NA	2.016E−1	2	0
HEPH	NA	3.955E−2	4	0	NA	2.016E−1	2	0
SCN1A	NA	3.955E−2	4	0	NA	8.961E−2	3	0
PPFIA1	NA	3.955E−2	4	0	NA	8.961E−2	3	0
EPHB4	4.57	4.592E−2	7	2	7.82	3.324E‐2	6	1
MMP2	4.57	4.592E−2	7	2	5.08	1.284E−1	4	1
TCF3	4.57	4.592E−2	7	2	3.76	2.398E−1	3	1
COL5A1	3.50	5.570E‐2	8	3	4.57	4.592E−2	7	2
ATP2C2	2.59	1.046E‐1	8	4	7.82	3.324E−2	6	1
SLC26A4	2.55	1.606E‐1	6	3	NA	3.955E−2	4	0
ASPH	2.55	1.606E−1	6	3	NA	3.955E−2	4	0
PSMB11	2.24	1.673E−1	7	4	NA	3.955E−2	4	0
RYR2	1.60	2.433E−1	10	8	3.50	5.570E−2	8	3
COL4A3	1.89	2.570E−1	6	4	NA	1.733E−2	5	0
HIPK2	0.94	6.424E−1	7	9	NA	1.733E−2	5	0
SLC9B1		1.00E0	82	100	2.93	3.021E−2	77	84

*Note*: * NA, not available as the number of cases or controls is zero; MAF, minor allele frequency.

### 3.5 PPI network construction and analysis

**FIGURE 4:**
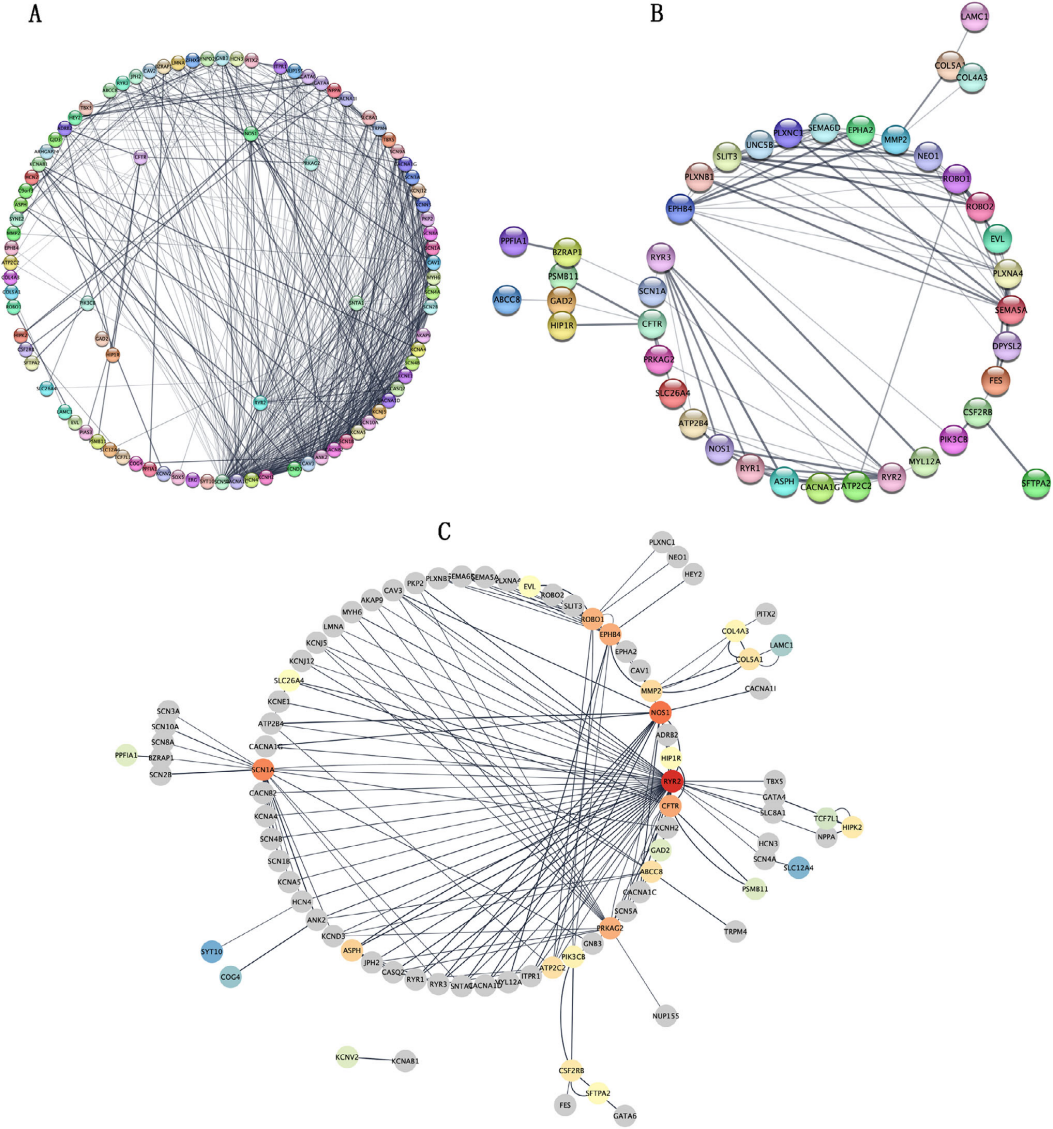
Protein‐protein interaction networks. A, The interaction network among the 36 candidate genes in gene‐based collapsing analysis and the 64 reference genes in the present study. B, The interaction network among the 37 candidate genes (including *RYR2*) in gene‐based collapsing analysis and the genes selected by pathway enrichment analysis in GWAS. C, The interaction network among the genes selected by (A) and (B)

**FIGURE 5:**
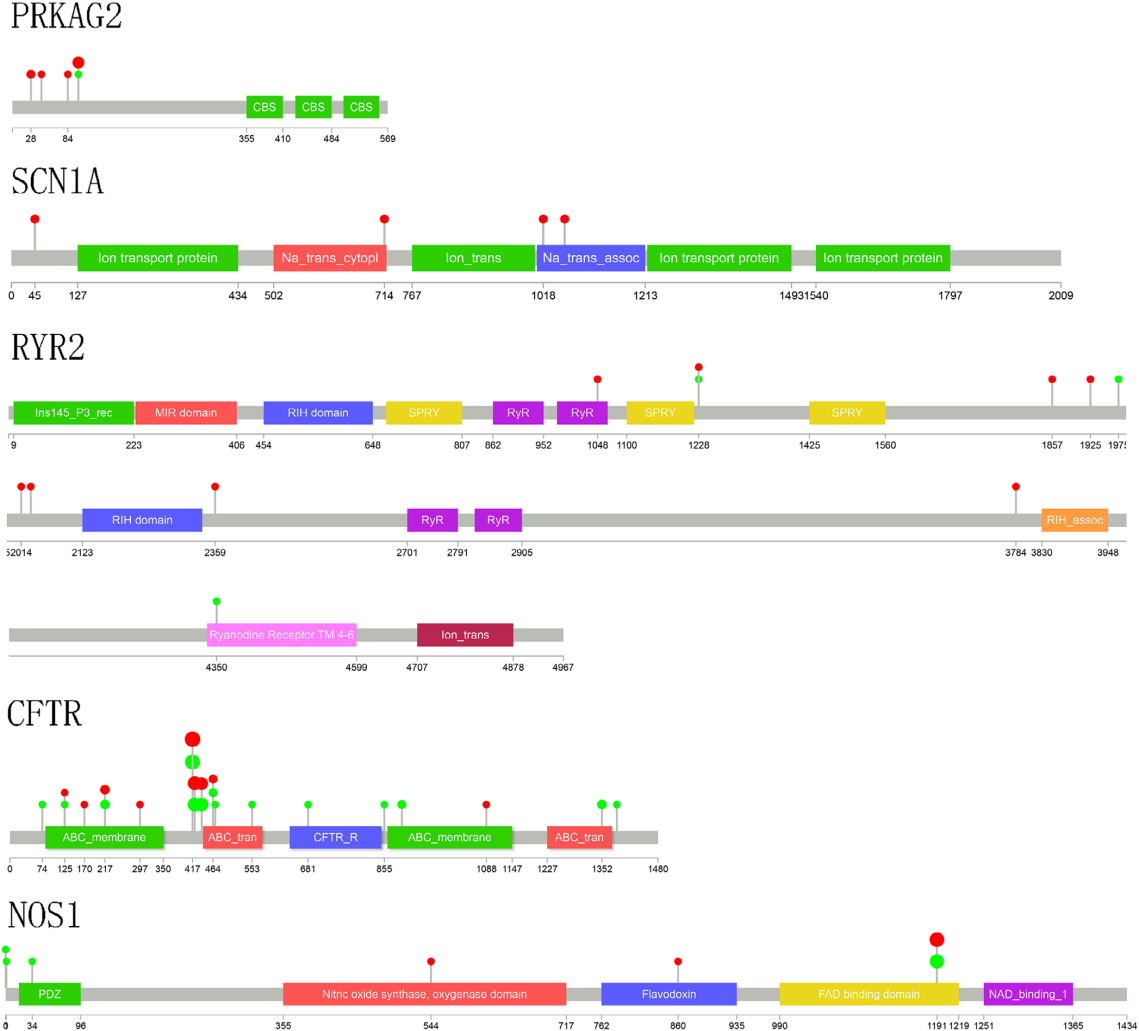
The rare variants in the five candidate genes such as *SCN1A, PRKAG2, RYR2, CFTR*, and *NOS1*

To determine the most relevant genes among the above 36 candidate genes from gene‐based collapsing analysis, the PPI network was constructed with STRING, which combined 64 reference genes with rare variants from the present study and 20 selected genes among the top 30 enriched pathways according to both the KEGG and Reactome databases in a GWAS. The nine most significant genes according to scores and nodes were *NOS1* (score = 6.795, nodes = 8.5), *SCN1A* (score = 6.071, nodes = 10.5), *CFTR* (score = 4.673, nodes = 6.5), *EPHB4* (score = 4.483, nodes = 7.5), *PRKAG2* (score = 4.335, nodes = 8), *ROBO1* (score = 4.241, nodes = 6.5), *ASPH* (score = 3.001, nodes = 3.5), *MMP2* (score = 2.665, nodes = 4), and *ABCC8* (score = 2.387, nodes = 4.5). Remarkably, *RYR2* (score = 14.88, nodes = 23.5) was ranked as the first PPI node among the reference genes with rare variants in the present study, and the *P* value of the burden gene test was nearly 0.05 (*P *= 0.55) with frequency categories (MAF < 0.001). Considering the functional roles of the genes and previous studies, the most likely candidate genes were *SCN1A, PRKAG2, RYR2, CFTR*, and *NOS1* (Figure 4 and Table 6), and the rare variants information for the selected top five genes is illustrated in Figure 5 and listed in Supplementary Table S11.

**TABLE 6:** PPI network combined scores

Genes	Number of nodes in group 1	Total scores	Mean scores	Number of nodes in group 2	Total scores	Mean scores	Combined mean total scores	Combined mean number of nodes
RYR2	37	23.226	0.628	10	6.533	0.653	14.880	23.5
NOS1	12	9.121	0.760	5	4.469	0.894	6.795	8.5
SCN1A	18	10.844	0.602	3	1.297	0.432	6.071	10.5
CFTR	8	5.596	0.700	5	3.750	0.75	4.673	6.5
EPHB4	4	2.463	0.616	11	6.508	0.592	4.483	7.5
PRKAG2	14	7.370	0.526	2	1.299	0.650	4.335	8
ROBO1	3	1.958	0.653	10	6.524	0.652	4.241	6.5
ASPH	4	3.105	0.776	3	2.897	0.966	3.001	3.5
MMP2	4	2.741	0.685	4	2.589	0.647	2.665	4
ABCC8	8	4.278	0.535	1	0.496	0.496	2.387	4.5
ATP2C2	4	2.077	0.519	5	2.687	0.537	2.382	4.5
COL5A1	3	2.28	0.760	3	2.280	0.760	2.280	3
CSF2RB	2	1.819	0.910	3	2.340	0.780	2.080	2.5
PIK3CB	3	1.977	0.659	2	1.361	0.681	1.669	2.5
COL4A3	3	1.782	0.594	2	1.356	0.678	1.569	2.5
HIPK2	3	2.433	0.811	1	0.625	0.625	1.529	2
SFTPA2	2	1.841	0.921	1	0.917	0.917	1.379	1.5
HIP1R	2	1.800	0.900	1	0.900	0.900	1.350	1.5
EVL	1	0.925	0.925	2	1.503	0.752	1.214	1.5
SLC26A4	2	1.143	0.572	2	1.186	0.593	1.165	2
PPFIA1	1	0.933	0.933	1	0.933	0.933	0.933	1
GAD2	2	0.929	0.465	2	0.929	0.465	0.929	2
PSMB11	1	0.905	0.905	1	0.905	0.905	0.905	1
TCF7L1	1	0.625	0.625	1	0.625	0.625	0.625	1
LAMC1	1	0.553	0.553	1	0.553	0.553	0.553	1
KCNV2	1	0.926	0.926	NA	NA	NA	0.463	0.5
COG4	1	0.902	0.902	NA	NA	NA	0.451	0.5
SLC12A4	1	0.591	0.591	NA	NA	NA	0.296	0.5
SYT10	1	0.400	0.400	NA	NA	NA	0.200	0.5

*Notes*: Group 1 means the PPI network was constructed with 36 candidate genes from gene‐based collapsing analysis and 64 referential target genes with mutations in the present study; group 2 means the PPI network was constructed with 37 candidate genes (including *RYR2*) from gene‐based collapsing analysis and 20 selected genes in the top 30 enrichment pathways with both KEGG and Reactome databases in GWAS analysis; NA, not available.

### 3.6 External data validation

**FIGURE 6:**
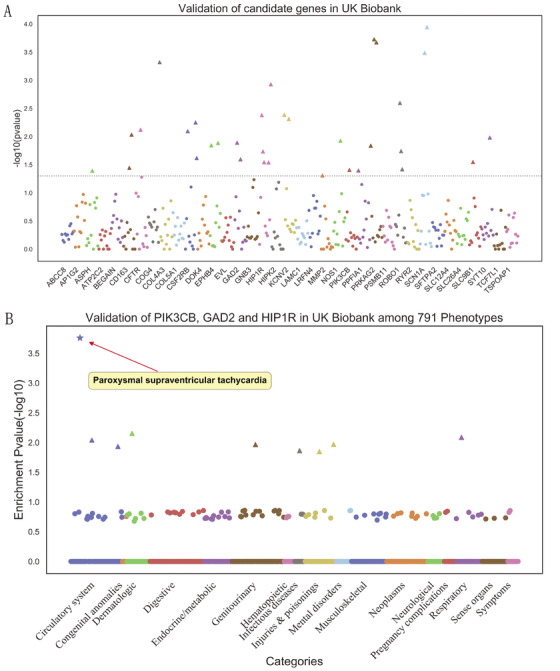
Verification of candidate 37 genes in UK Biobank (A); the three of candidate burden genes, *PIK3CB, GAD2*, and *HIP1R*, showed the most significant enrichment in PSVT (*P *= 0.000174) among 791 phenotypes in UK Biobank (B)

To verify the candidate pathogenic genes that we screened, we selected the UK Biobank resource for external data validation. The database was the most recent upload of the total exome sequencing data from 49 960 participators.^22^ We searched for rare variants in our candidate genes that were associated with arrhythmias in UK Biobank summary statistics database. Among these 37 candidate genes (36 genes from the gene‐based collapsing analysis and *RYR2*), we obtained information about 33 rare variants in 18 genes in this database of arrhythmia patients; these genes were S*CN1A, PRKAG2, CFTR, NOS1, PIK3CB, GAD2, HIP1R, ASPH, CD163, SLC9B1, ROBO1, EPHB4, KCNV2, PPFIA1, SYT10, COG4, MMP2*, and *CSF2RB*. In particular, rare variants in three genes, *PIK3CB, GAD2* and *HIP1R*, were present even in patients with PSVT (Figure 6, Table 7, and supplementary Table S12). Moreover, we applied enrichment analysis to explore the correlation between phenotypes and burden gene. Consequently, *PIK3CB*, *GAD2*, and *HIP1R* genes showed the most significant enrichment in PSVT (*P *= 0.000174) among 791 phenotypes in UK Biobank (Figure 6, Table 8, Supplementary Table S13).

**TABLE 7:** The external data validation of candidate genes by the UK Biobank resource

Genes	Start position	End position	Number of rare variants	MAC cases	MAC controls	Cases	Controls	*P*‐value	Phenotype code	Phenotype name
ASPH	8:61503374:G:C	8:61651126:T:C	220	89.00	1663.043487	2570	42 728	4.035E−2	427.00	Cardiac dysrhythmias
CD163	12:7479895:T:C	12:7502526:T:C	69	4	150.0004211	95	9 405	3.605E−2	426.91	Cardiac pacemaker in situ
CD163	12:7479895:T:C	12:7502526:T:C	79	5	171.00	108	10 692	9.246E−3	426.90	Cardiac pacemaker/device in situ
CFTR	7:117480105:C:T	7:117592658:G:A	197	14.00	932.09	95	9 405	7.580E−3	426.91	Cardiac pacemaker in situ
COG4	16:70481032:T:C	16:70512433:C:T	62	4	151	93	9 207	4.767E−4	427.50	Arrhythmia (cardiac) NOS
CSF2RB	22:36922267:C:A	22:36938476:G:A	92	4	234.00	108	10 692	8.011E−3	426.9	Cardiac pacemaker/device in situ
CSF2RB	22:36922267:C:A	22:36938476:G:A	84	4	202.00	95	9 405	5.619E−3	426.91	Cardiac pacemaker in situ
CSF2RB	22:36922267:C:A	22:36938476:G:A	79	5	219	104	10 296	2.413E−2	427.40	Cardiac arrest and ventricular fibrillation
EPHB4	7:100803517:A:G	7:100826991:C:T	107	10	487.00	116	11 484	1.433E−2	425.00	Cardiomyopathy
EPHB4	7:100805215:C:T	7:100826991:C:T	81	5	181.01	78	7 722	1.302E−2	427.12	Paroxysmal ventricular tachycardia
GAD2	10:26216843:G:A	10:26245919:A:G	76	17	1590.00	276	27 324	1.285E−2	427.11	Paroxysmal supraventricular tachycardia
GAD2	10:26216843:G:A	10:26245919:A:G	85	19	2030.00	354	35 046	2.535E−2	427.10	Paroxysmal tachycardia, unspecified
HIP1R	12:122834989:T:C	12:122860524:G:A	144	11	472.00	108	10 692	4.155E−3	426.90	Cardiac pacemaker/device in situ
HIP1R	12:122834978:A:C	12:122861041:T:C	291	24.00	1507.01	354	35 046	1.837E−2	427.10	Paroxysmal tachycardia, unspecified
HIP1R	12:122834989:T:C	12:122860524:G:A	126	8	355.00	78	7 722	2.860E−2	427.12	Paroxysmal ventricular tachycardia
HIP1R	12:122834989:T:C	12:122860524:G:A	249	17.00	1185.01	276	27 324	2.887E−2	427.11	Paroxysmal supraventricular tachycardia
HIP1R	12:122834989:T:C	12:122860524:G:A	134	11	418.00	95	9 405	1.179E−3	426.91	Cardiac pacemaker in situ
KCNV2	9:2717744:D:4	9:2729710:D:5	88	5.00	199.00	87	8 613	4.110E−3	427.42	Cardiac arrest
KCNV2	9:2717744:D:4	9:2729710:D:5	91	5.00	218.00	104	10 296	4.879553E−3	427.40	Cardiac arrest and ventricular fibrillation
MMP2	16:55479557:C:A	16:55505425:G:A	53	2	91.00	78	7 722	4.910E−2	427.12	Paroxysmal ventricular tachycardia
NOS1	12:117218074:T:C	12:117278099:C:G	272	143.00	3398.00	1468	42 760	1.183E−2	427.20	Atrial fibrillation and flutter
PIK3CB	3:138655414:G:A	3:138759288:G:A	95	5	213.00	276	27 324	3.915E−2	427.11	Paroxysmal supraventricular tachycardia
PPFIA1	11:70272251:C:G	11:70382137:C:G	73	3	137.00	78	7 722	4.004E−2	427.12	Paroxysmal ventricular tachycardia
PRKAG2	7:151557224:G:A	7:151675419:C:T	38	2	51	78	7 722	1.452E−2	427.12	Paroxysmal ventricular tachycardia
PRKAG2	7:151557224:G:A	7:151595452:C:T	36	4	52.00	87	8 613	1.868E−4	427.42	Cardiac arrest
PRKAG2	7:151557224:G:A	7:151595452:C:T	41	4	62.00	104	10 296	2.122E−4	427.40	Cardiac arrest and ventricular fibrillation
ROBO1	3:78600114:T:A	3:79018406:C:T	344	56.00	1702.02	1468	42 760	2.524E−3	427.20	Atrial fibrillation and flutter
ROBO1	3:78600114:T:A	3:79018406:C:T	350	92.01	1700.02	2570	42 728	1.813E−2	427.00	Cardiac dysrhythmias
ROBO1	3:78600114:T:A	3:78598929:T:C	118	8.00	344.01	93	9 207	3.817E−2	427.50	Arrhythmia (cardiac) NOS
SCN1A	2:165991287:T:G	2:166041470:C:A	85	6	274.02	104	10 296	3.253E−4	427.40	Cardiac arrest and ventricular fibrillation
SCN1A	2:165991287:T:G	2:166041470:C:A	69	6	225.02	87	8 613	1.139E−4	427.42	Cardiac arrest
SLC9B1	4:102901162:A:T	4:102991714:T:C	47	17	736.01	104	10 296	2.837E−2	427.40	Cardiac arrest and ventricular fibrillation
SYT10	12:33376837:G:A	12:33439521:A:G	46	3	97.00	116	11 484	1.042E−2	425.00	Cardiomyopathy

*Note*: MAC: Minor allele counts.

**TABLE 8:** Candidate Genes Enrichment Analysis for Phenotype Category in UK Biobank (p < 0.05)

Phenotype category name	Enrichment *P* value	−Log10(*P* value)	Category name	Phenotype code
Paroxysmal supraventricular tachycardia	0.000174146	3.759	Circulatory system	427.11
Paroxysmal tachycardia, unspecified	0.009124029	2.040	Circulatory system	427.1
Aneurysm and dissection of heart	0.01165878	1.933	Circulatory system	411.41
Other hypertrophic and atrophic conditions of skin	0.007023506	2.153	Dermatologic	701
Nephritis; nephrosis; renal sclerosis	0.010817149	1.966	Genitourinary	580
Intestinal infection due to C. difficile	0.0137383	1.862	Infectious diseases	008.52
Anaphylactic shock NOS	0.014210121	1.847	Injuries and poisonings	946
Opiates and related narcotics causing adverse effects in therapeutic use	0.01073407	1.969	Injuries and poisonings	965.1
Bacterial pneumonia	0.008187189	2.087	Respiratory	480.1

Because the disease information of UK Biobank was not specific enough, we chose the only known AVNRT genetic sequencing study to further validate our candidate pathogenic genes. The study, published in 2018, was carried out in Denmark, and 67 known arrhythmia target genes were detected in AVNRT cases by next‐generation sequencing.^13^ Among our candidate genes, *SCN1A, RYR2*, and *PRKAG2*, there were 11 rare variants in *SCN1A* and three rare variants in *RYR2* detected in AVNRT patients in the Danish study, especially, a rare variant in *RYR2* (c.4652A > G, p.Asn1551Ser, rs185237690) in our present study was also found in one Danish AVNRT case, which supports *SCN1A* and *RYR2* gene as candidate pathogenic genes in our study. The detail rare variants information is shown in Table 9.

**TABLE 9:** Overlapped rare variants of candidate genes in an AVNRT study from Denmark

Our present study									Danish AVNRT study							
Gene	cDNA	Protein variant	Transcript	Translation	KEGG EAS AF	ExAC EAS AF	Cases (*n*)	Controls (*n*)	Gene	cDNA	Protein variant	Transcript	Translation	MAF ExAC	MAF D2K^†^	Allele count
RYR2	*c.4652A > G*	*p.Asn1551Ser*	*XM_005273224.1*	*missense*	.	*4.52E*−*03*	*1*	*0*	RYR2	*c.4652A > G*	*p.Asn1551Ser*	*ENST00000366574*	*missense*	*3.50E*−*04*	*5.00E*−*04*	*1*
	c.4094C > T	p.Ala1365Val	XM_005273224.1	missense	7.90E−03	4.21E‐03	2	4		c.1088T > C	p.Ile363Thr	ENST00000366574	missense	0	0	1
	c.7076G > A	p.Arg2359Gln	XM_005273224.1	missense	.	7.00E−04	1	0		c.1115T > A	p.Leu372His	ENST00000366574	missense	1.00E−05	2.50E−04	1
	c.3143A > G	p.Asp1048Gly	XM_005273224.1	missense	.	.	1	0		c.1250G > A	p.Arg417Gln	ENST00000366574	missense	2.00E−05	0.00E+00	2
	c.6040G > T	p.Asp2014Tyr	XM_005273224.1	missense	.	.	1	0		c.2828T > C	p.Leu943Ser	ENST00000366574	missense	2.30E−04	0.00E+00	1
	c.5774T > C	p.Ile1925Thr	XM_005273224.1	missense	.	0	1	0		c.3251G > A	p.Arg1084Lys	ENST00000366574	missense	1.50E−04	7.50E−04	1
	c.11352T > G	p.Ile3784Met	XM_005273224.1	missense	.	.	1	0		c.5186T > C	p.Met1729Thr	ENST00000366574	missense	0	0	1
	c.13050A > C	p.Leu4350Phe	XM_005273224.1	missense	.	.	0	1		c.8162T > C	p.Ile2721Thr	ENST00000366574	missense	5.70E−04	4.13E−03	1
	c.5923A > G	p.Met1975Val	XM_005273224.1	missense	.	2.34E−04	0	1		c.10468G > T	p.Ala3490Ser	ENST00000366574	missense	0	2.50E−04	1
	c.5570C > T	p.Pro1857Leu	XM_005273224.1	missense	0	1.17E−46	1	0		c.10528C > A	p.Arg3510Ser	ENST00000366574	missense	3.00E−05	2.50E−04	1
	c.6092C > T	p.Ser2031Phe	XM_005273224.1	missense	.	1.16E−04	1	0		c.10846G > T	p.Ala3616Ser	ENST00000366574	missense	0	0	1
	c.3683C > A	p.Thr1228Asn	XM_005273224.1	missense	.	.	1	1		.	.	.	.	.	.	.
	c.3721G > A	p.Val1241Ile	XM_005273224.1	missense	2.00E‐03	2.33E−04	0	1		.	.	.	.	.	.	.
SCN1A	c.3176A > T	p.Asp1059Val	NM_001165963.1	missense	.	3.52E−08	1	0	SCN1A	c.3521C > G	p.Thr1174Ser	ENST00000303395	missense	1.77E−03	3.25E−03	2
	c.3053G > A	p.Arg1018Lys	NM_001165963.1	missense	.	2.32E−08	1	0		c.1625G > A	p.Arg542Gln	ENST00000303395	missense	1.53E−03	1.75E−03	1
	c.2141T > G	p.Met714Arg	NM_001165963.1	missense	.	.	1	0		c.1199T > C	p.Met400Thr	ENST00000303395	missense	0	0	1
	c.135C > G	p.Asp45Glu	NM_001165963.1	missense	1.00E−03	8.09E−04	1	0		.	.	.	.	.	.	.

*†*2000 Danish Exomes; KEGG, *Kyoto Encyclopedia of Genes and Genomes*;ExAC, Exome Aggregation Consortium; EAS, East Asian; MAF, Minor allele frequency.

## 4 DISCUSSION

To our knowledge, this is the first study with the primary aim of investigating the genetic contribution of AVNRT using a WES approach. In the present study and an external data validation, genes such as *SCN1A, PRKAG2, RYR2, CFTR*, *NOS1, PIK3CB, GAD2*, and *HIP1R*, responsible for neuronal system/neurotransmitter release or ion channel/cardiac conduction, are likely to be candidate genes and pathways for AVNRT. As this is only a pilot study of the genetic investigation of AVNRT, further genetic functional studies are needed.

Recently, an increasing number of clinical reports have suggested that there may be a hereditary contribution to AVNRT.^4^, ^5^, ^6^, ^7^ However, little is known about the hereditary role in AVNRT compared with that for Wolff‐Parkinson‐White syndrome.^8^, ^9^ A recent study involving the sequencing of 67 selected genes associated with arrhythmia in 298 AVNRT patients found the greatest number of variants in sodium and calcium channels, indicating that AVNRT might be an arrhythmic disease with abnormal sodium and calcium handling.^13^ Among the reference genes from the present study, many rare variants were detected in *KCNJ12, RYR3, RYR2, ZFHX3, ANK2, AKAP9, GNB3, SYNE2, CACNA1D, CACNA1I, GNB3, MYH6, SCN5A, SCN1A, SCN3A, HCN4*, and *KCNH2*. Most of these genes, such as *KCNJ12, RYR3*, *RYR2, CACNA1D, CACNA1I, SCN5A, SCN1A, SCN3A, HCN4*, and *KCNH2*, encode ion channels, indicating that AVNRT was associated with ion channels. Interestingly, the causal gene of Wolff‐Parkinson‐White syndrome, *PRKAG2*, was also identified in our AVNRT cases.

The autonomic nervous system is known to take part in the triggering and termination of AVNRT.^23^, ^24^, ^25^, ^26^ There are extrinsic and intrinsic components of the cardiac autonomic nervous system, and the extrinsic component is divided into sympathetic and parasympathetic systems, involving the main neurotransmitters of norepinephrine and acetylcholine, respectively.^27^, ^28^ The intrinsic cardiac autonomic nervous system of the ganglionated plexi contains both sympathetic and parasympathetic fibers and is connected with a wide range of neurotransmitters.^29^, ^30^ The AV node exhibits dense parasympathetic innervation, and changes in the cardiac autonomic nervous system could lead to arrhythmias.^31^ Usually, sympathetic stimulation is used to facilitate the induction of AVNRT.^23^, ^24^ However, the onset of AVNRT occurs at times of increased vagal tone, as the vagal tone increases the refractory period of the fast pathway and a premature atrial complex may be conducted antegrade via the slow pathway with subsequent retrograde conduction, thus initiating AVNRT.^25^, ^26^ In our present study, many rare variants in genes involved in the neurotransmitter release cycle and neuronal system pathways, such as the neurotransmitter release cycle, serotonin neurotransmitter release cycle, acetylcholine neurotransmitter release cycle, and norepinephrine neurotransmitter release cycle, were present among the top 30 enriched pathways; the similar outcomes were also presented in GWAS analysis, indicating that neurotransmitter release affects the sympathetic or parasympathetic system and then induces AVNRT.

The autonomic nervous system also shows a close relationship with cardiac ionic conductance. Vagal nerve endings release acetylcholine, activate the ACh‐activated K^+^ current (*I*_k‐ACh_), and inhibit the funny current (*I*_f_) and the L‐type Ca^2+^ current.^32^ In contrast, sympathetic nerve endings release noradrenaline to increase the *I*_f_ and the L‐type Ca^2+^ current and induce changes in intracellular Ca^2+^ handling.^33^ In addition, many arrhythmias occur due to genetic mutations in ion channels themselves, and the mutations will affect the sodium, potassium, and calcium channels responsible for ion transport across the myocardial cell membrane, then, the action potential is altered and induces arrhythmias.^16^, ^17^, ^18^ In the present study, the pathways of ion channels and cardiac conduction were among the top 30 enriched pathways, and genes such as those encoding sodium channels (*SCN1A*) and potassium channels (*KCNV2*) were selected as candidate genes; in particular, *SCN1A* is regarded as one of the most likely candidate pathogenic genes. Mutations in ion channel genes might affect the conduction of AV nodes, and differences in conduction velocity will lead to dual AV node physiology and AVNRT.

There were three genes with rare variants reported to be associated with arrhythmia among the candidate genes in the present study. The first was the *PRKAG2* gene, encoding the gamma2 regulatory subunit of adenosine monophosphate‐activated protein kinase, which was identified as the pathogenic gene of Wolff‐Parkinson‐White syndrome.^8^, ^9^ *PRKAG2* mutations induce the slowing of sodium channel inactivation and increase the likelihood of channel activation at more negative potentials.^34^ The integral of the sodium current (total inward current) is a major determiner of conduction velocity, and this process can be speeded up by increasing in inward sodium current, resulting in a conduction velocity change in the AV node.^35^ The second gene was *SCN1A*, which is primarily a neuronal gene. Nav1.1, a product of *SCN1A*, is present in various regions of the heart.^36^, ^37^ *SCN1A* mutations are found in up to 80% of patients with Dravet syndrome, a type of epilepsy observed in infancy, and sudden unexpected death results in 38% of all deaths in patients with a childhood onset.^38^ Although the mechanism remains poorly understood, the sodium channel‐dependent cardiac current is increased in *SCN1A‐R1407X* knock‐in mice,^39^ and autonomic dysfunctions such as abnormalities in heart rate variability, QT and P wave dispersion are observed in patients with Dravet syndrome,^40^ suggesting that some *SCN1A* variants might cause sudden death or lethal arrhythmia through neurocardiac or solely cardiac mechanisms. The third gene was *RYR2*, encoding cardiac ryanodine receptors (RYR2s), which are large intracellular Ca^2+^ channels that regulate the release of Ca^2+^ from the sarcoplasmic reticulum in cardiomyocytes.^41^ Mutations in *RYR2* can increase the probability of channel open during diastole, resulting in excess diastolic SR Ca^2+^ release, and the increased SR Ca^2+^ leak during diastole can increase the frequency of spontaneous Ca^2+^ sparks, resulting in an untimely depolarizing inward current that triggers delayed after depolarization and ventricular arrhythmia or atrial fibrillation.^41^ As mutations in these three genes have been proven to cause arrhythmia by experimental and clinical data, and were verified by the external data of UK Biobank and the genetic study from Denmark, it is reasonable for us to assume that the gene rare variants identified in the present study could also cause AVNRT.

Among the other two candidate genes, the first was *CFTR*. It encodes a cAMP‐activated chloride channel (cystic fibrosis transmembrane conductance regulator, CFTR) and is presented mainly in epithelial cells of the respiratory and digestive tracts; mutations in this gene cause cystic fibrosis.^42^ Subsequent studies demonstrated that CFTR acts not only as an ATP‐gated chloride channel but also as a regulator of other ion channels, such as amiloride‐sensitive Na^+^ channels, ATP channels, and inward rectifier K^+^ channels.^43^, ^44^, ^45^ Recently, in cardiac CFTR‐overexpressing mice, intracardiac electrophysiological studies showed remarkable slowing of conduction parameters, including high‐grade AV block, with easily inducible nonsustained ventricular tachycardia following isoproterenol administration.^46^ The second of these genes was *NOS1*. It encodes neuronal nitric oxide synthase (nNOS) and is a major isoform within the brain.^47^ nNOS, together with its chaperone protein (CAPON), is also found in both postganglionic sympathetic neurons of the stellate ganglia and intrinsic cardiac vagal neurons.^48^, ^49^ Moreover, the overexpression of nNOS increases acetylcholine release,^50^ and CAPON overexpression in myocytes attenuates the L‐type calcium current, slightly increases the rapid delayed rectifier current (*I*_kr_), and shortens action potential,^51^ which causes arrhythmia susceptibility. Thus, although the two candidate genes *CFTR* and *NOS1* have not been proven to directly cause arrhythmia, all the potential evidence listed above shows that these gene mutations can change some characteristics of atrioventricular node conduction by affecting the autonomic nervous system/neurotransmitter release or ion channels, which can lead to changes in cardiac depolarization, action potentials, cardiac conduction velocity, the refractory period, etc., and such changes will lead to dual AV node physiology and AVNRT.

As for the three candidate genes, *PIK3CB, GAD2*, and *HIP1R*, were present even in patients with PSVT in the UK Biobank resource. However, there was no data indicating the three genes play any role in cardiac arrhythmia. *PIK3CB* encodes an isoform of the catalytic subunit of phosphoinositide 3‐kinase beta (PI3Kβ), recent data showed PI3K signaling activation affected currents of multiple ion channels, including calcium and sodium channels, and suppression of PI3K activation displayed a prolonged QT interval.^52^ *GAD2* is a glutamate decarboxylase 2 coding gene, diseases associated with *GAD2* include autoimmune polyendocrine syndrome and stiff‐person syndrome, among its related pathways are neurotransmitter release cycle and database. Diseases associated with *HIP1R* (Huntingtin interacting protein 1 related) include expressive language disorder and cataract. At present, there is a lack of data about mutations of the three candidate genes in arrhythmia or AVNRT, which needs to be confirmed by other large samples genetic research or functional verification.

## 5 LIMITATIONS

The major limitation of the current study is the small sample size as AVNRT with low prevalence in population. Although we identified a few candidate genes, such as *SCN1A, PRKAG2, RYR2, CFTR*, *NOS1, PIK3CB, GAD2*, and *HIP1R*, in AVNRT in the present study, the genes were not verified experimentally, and further research is needed to explore the potential mechanisms of these genes. Since AVNRT is caused by complex molecular mechanisms, a single pathway is not sufficient to explain the pathogenesis of this disease. Therefore, further experimental research is needed to confirm the current findings. Finally, the controls included in the present study did not undergo invasive electrophysiological examination, and it is possible that the controls were not completely devoid of AVNRT.

## 6 CONCLUSIONS

**FIGURE 7:**
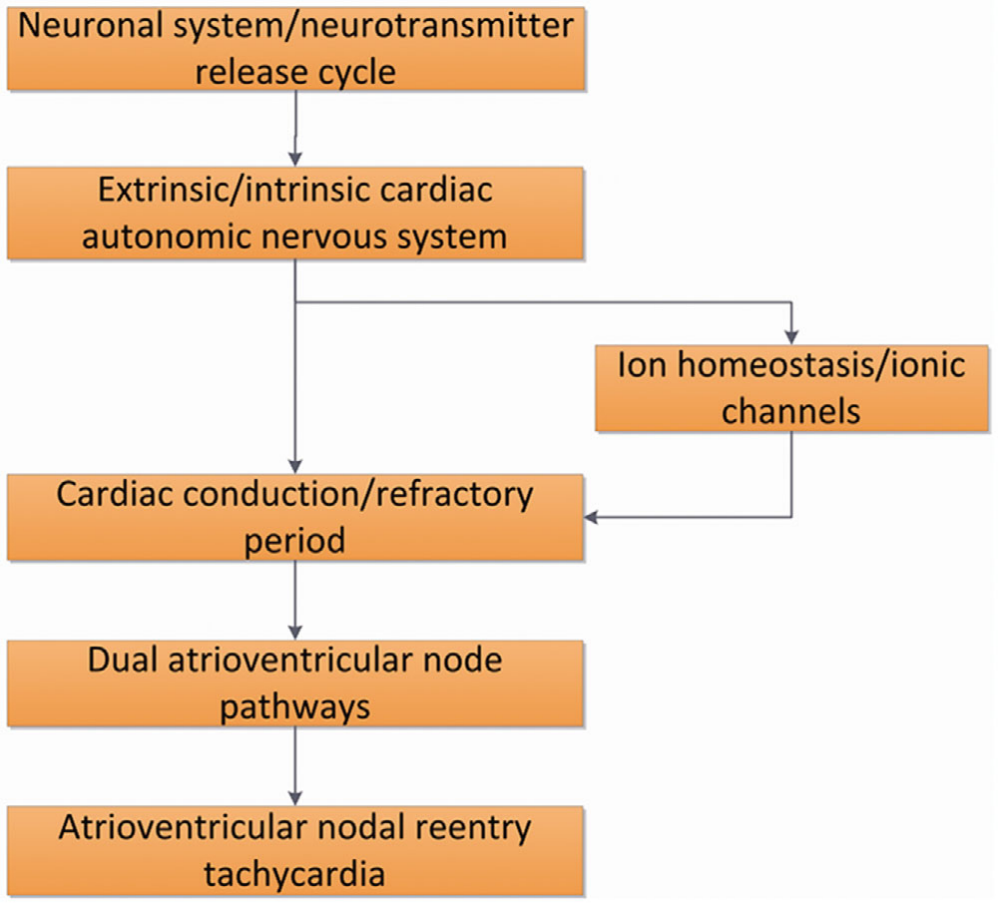
Summary of the associated signal pathways and the potential links with AVNRT

Our study identified a number of potentially disease‐related genes, such as *SCN1A, PRKAG2, RYR2, CFTR*, *NOS1, PIK3CB, GAD2*, and *HIP1R*, in the pathways of neuronal system/neurotransmitter release cycles or ion channel/cardiac conduction, which require further replication in larger cohorts and functional confirmation. Because the anatomic substrate in AVNRT remains unclear, our findings may provide insight into the molecular basis of AVNRT and provide a new view of AVNRT as shown in Figure 7.

## FUNDING

This work was supported by National Natural Science Foundation of China (No. 81770379, 81500297, 81470521, and 81670290).

## CONFLICTS OF INTEREST

The authors report no conflicts of interest. The authors alone are responsible for the content and writing of this article. The manuscript has been approved by the responsible authorities of the institutions where the work was conducted, and all authors have read the manuscript and approved its submission to your journal.

## Supporting information

Supporting Information

Supporting Information S1

Supporting Information S2

Supporting Information S3

Supporting Information S4

Supporting Information S5

Supporting Information S6

Supporting Information S7

Supporting Information S8

Supporting Information S9

Supporting Information S10

Supporting Information S11

Supporting Information S12

Supporting Information S13

Supporting Information S14

Supporting Information S15

Supporting Information S16
