# ERRATUM

**DOI:** 10.1002/ctm2.274

**Published:** 2021-02-14

In Luo et al.,^1^ information on the co‐first authors of the article was omitted.

Rong Luo and Chenqing Zheng are co‐first authors of the article.

The author byline should thus read:

Rong Luo PhD^1†^ | Chenqing Zheng MD^2†^ | Hao Yang MD^3^ | Xuepin Chen MD^3^ | Panpan Jiang PhD^4^ | Xiushan Wu PhD^5^ | Zhenglin Yang PhD^3^ | Xia Shen PhD^2,6,7^ | Xiaoping Li MD, PhD^3^

And the following should have been added as a footnote:

^†^Co‐first authors

The online version of this article was corrected.
