# Supplementary material for: Identification of potential candidate genes and pathways in atrioventricular nodal reentry tachycardia by whole‐exome sequencing
Source: Clin Transl Med. 2020 Apr 30;10(1):238–57. doi: 10.1002/ctm2.25 (PMC7240861; doi:10.1002/ctm2.25)
Supplement: Supplementary file 1 — Supporting Information [file CTM2-10-238-s001.docx]

**S1: Reference genes list**

| **Reference genes** | **Ensembl**  **gene ID** | **Functional**  **classification** | **Included**  **reason** | **References** |
| --- | --- | --- | --- | --- |
| ABCC9 | ENSG00000069431.6 | ATP-binding cassette, sub-family C (CFTR/MRP), member 9 | Involved in Brugada syndrome | Antzelevitch C, et al. 2016 |
| ADRB1 | ENSG00000043591.4 | Adrenergic receptors | Expressed in human atrioventricular conduction axis | Greener ID, et al. 2011 |
| ADRB2 | ENSG00000169252.4 | Adrenergic receptors | Expressed in human atrioventricular conduction axis | Greener ID, et al. 2011 |
| AGT | ENSG00000135744.7 | Angiotensinogen | Associated with atrial fibrillation | Tsai CT, et al. 2008 |
| AKAP9 | ENSG00000127914.12 | A kinase (PRKA) anchor protein 9 | Involved in long QT syndrome | Andreasen L, et al. 2018  Giudicessia JR, et al. 2018 |
| ANK2 | ENSG00000145362.12 | Ankyrin 2, neuronal | Involved in AVNRT | Andreasen L, et al. 2018 |
| ANP | ENSG00000175206.10 | Hormone expressed by atrial muscle | Expressed in human atrioventricular conduction axis | Greener ID, et al. 2011 |
| ARHGAP24 | ENSG00000138639.13 | Rho GTPase activating protein 24 | Associated with PR interval; Involed in AVNRT | Andreasen L, et al. 2018  Pfeufer A, et al. 2010 |
| ATBF1 | ENSG00000140836.10 | Zinc finger homeobox 3 | In volved in atrial fibrillation | Pfeufer A, et al. 2010 |
| ATP2A2 | ENSG00000174437.12 | ATPase, Ca^2+^ transporting, cardiac muscle | Involed in AVNRT | Andreasen L, et al. 2018 |
| C9orf3 | ENSG00000285807.1 | AL353768.2 (Clone-based (Ensembl) gene) | In volved in atrial fibrillation | Martin RI, et al. 2015 |
| CACNA1C | ENSG00000151067.16 | Ca^2+^ channels | Expressed in human atrioventricular conduction axis, involved in long QT sydrome | Greener ID, et al. 2011  Giudicessia JR, et al. 2018 |
| CACNA1D | ENSG00000157388.9 | Ca^2+^ channels | Expressed in human atrioventricular conduction axis | Greener ID, et al. 2011 |
| CACNA1G | ENSG00000006283.13 | Ca^2+^ channels | Expressed in human atrioventricular conduction axis | Greener ID, et al. 2011 |
| CACNA1I | ENSG00000100346.13 | Ca^2+^ channels | Expressed in human atrioventricular conduction axis | Greener ID, et al. 2011 |
| CACNB2 | ENSG00000165995.20 | Calcium voltage-gated channel auxiliary subunit beta 2 | Involved in AVNRT | Andreasen L, et al. 2018 |
| CASQ2 | ENSG00000118729.11 | Calsequestrin 2 | Involved in AVNRT | Andreasen L, et al. 2018 |
| CAV1 | ENSG00000105974.7 | Caveolae involved in signal transduction | Expressed in atrial myocytes | Pfeufer A, et al. 2010 |
| CAV2 | ENSG00000105971.10 | Caveolae involved in signal transduction | Associated with PR interval | Pfeufer A, et al. 2010 |
| CAV3 | ENSG00000182533.7 | Caveolae involved in signal transduction | Involved in long QT syndrome | Pfeufer A, et al. 2010  Giudicessia JR, et al. 2018 |
| Cx31.9 | ENSG00000183153.7 | Connexins, gap junction protein delta 3 | Expressed in human atrioventricular conduction axis | Greener ID, et al. 2011 |
| Cx40 | ENSG00000177291.4 | Connexins, gap junction protein delta 4 | Expressed in human atrioventricular conduction axis | Greener ID, et al. 2011 |
| Cx43 | ENSG00000152661.9 | Connexins, gap junction protein alpha 1 | Expressed in human atrioventricular conduction axis | Greener ID, et al. 2011 |
| Cx45 | ENSG00000182963.10 | Connexins, gap junction protein gamma 1 | Expressed in human atrioventricular conduction axis | Greener ID, et al. 2011 |
| DPP6 | ENSG00000130226.12 | Accessory proteins for voltage-gated K^+^channels | Expressed in human atrioventricular conduction axis | Greener ID, et al. 2011 |
| ERG | ENSG00000157554.14 | Delayed rectifier K^+^ channels | Expressed in human atrioventricular conduction axis | Greener ID, et al. 2011 |
| FREQ | ENSG00000107130.10 | Voltage-gated K^+^ channel regulatory proteins neuronal calcium sensor 1 | Expressed in human atrioventricular conduction axis | Greener ID, et al. 2011 |
| GATA4 | ENSG00000136574.17 | GATA binding protein 4 | Involved in heart development | Peterkin T, et al. 2005 |
| GATA5 | ENSG00000130700.7 | GATA binding protein 5 | Involved in heart development | Peterkin T, et al. 2005 |
| GATA6 | ENSG00000141448.10 | GATA binding protein 6 | Involved in heart development | Peterkin T, et al. 2005 |
| GNB3 | ENSG00000111664.10 | G protein subunit beta 3 | Associated with atrial fibrillation | Schreieck J, et al. 2004 |
| GPD1L | ENSG00000152642.6 | Glycerol-3-phosphate dehydrogenase 1 like | Involved in AVNRT | Andreasen L, et al. 2018 |
| HCN1 | ENSG00000164588.4 | HCN channels | Expressed in human atrioventricular conduction axis | Greener ID, et al. 2011 |
| HCN2 | ENSG00000099822.2 | HCN channels | Expressed in human atrioventricular conduction axis | Greener ID, et al. 2011 |
| HCN3 | ENSG00000263324.1 | HCN channels | Expressed in human atrioventricular conduction axis | Greener ID, et al. 2011 |
| HCN4 | ENSG00000138622.3 | HCN channels | Expressed in human atrioventricular conduction axis | Greener ID, et al. 2011 |
| HEY2 | ENSG00000135547.9 | Hes related family bHLH transcription factor with YRPW motif 2 | Involved in Brugada syndrome | Antzelevitch C, et al. 2016 |
| IP3R1 | ENSG00000150995.19 | Inositol 1,4,5-trisphosphate receptor type 1 | Expressed in human atrioventricular conduction axis | Greener ID, et al. 2011 |
| ITPR1 | ENSG00000150995.19 | Inositol 1,4,5-trisphosphate receptor type 1 | Involved in AVNRT | Andreasen L, et al. 2018 |
| JPH2 | ENSG00000149596.6 | Junctophilin 2 | Involved in heart failure | [Hu J](https://www.ncbi.nlm.nih.gov/pubmed/?term=Hu%20J%5BAuthor%5D&cauthor=true&cauthor_uid=30867288), et al. 2019 |
| KchAP | ENSG00000131788.16 | Voltage-gated K^+^ channel regulatory proteins | Expressed in human atrioventricular conduction axis | Greener ID, et al. 2011 |
| KchIP2 | ENSG00000120049.19 | Potassium voltage-gated channel interacting protein 2 | Expressed in human atrioventricular conduction axis | Greener ID, et al. 2011 |
| KCNA4 | ENSG00000182255.6 | Transient outward K^+^ channels | Expressed in human atrioventricular conduction axis | Greener ID, et al. 2011 |
| KCNA5 | ENSG00000130037.3 | Delayed rectifier K^+^ channels | Expressed in human atrioventricular conduction axis | Greener ID, et al. 2011 |
| KCNAB1 | ENSG00000169282.13 | Potassium voltage-gated channel, shaker-related subfamily, beta member 1 | Involved in AVNRT | Andreasen L, et al. 2018 |
| KCNAB2 | ENSG00000069424.15 | Potassium voltage-gated channel subfamily A regulatory beta subunit 2 | Involved in AVNRT | Andreasen L, et al. 2018 |
| KCND2 | ENSG00000184408.5 | Transient outward K^+^ channels | Expressed in human atrioventricular conduction axis | Greener ID, et al. 2011 |
| KCND3 | ENSG00000171385.5 | Transient outward K^+^ channels | Expressed in human atrioventricular conduction axis | Greener ID, et al. 2011 |
| KCNE1 | ENSG00000180509.7 | Potassium voltage-gated channel, Isk-related family, member 1 | Involved in long QT syndrome | Giudicessia JR, et al. 2018 |
| KCNE2 | ENSG00000159197.3 | Potassium voltage-gated channel subfamily E regulatory subunit 2 | Involved in long QT syndrome | Giudicessia JR, et al. 2018 |
| KCNE3 | ENSG00000175538.6 | potassium voltage-gated channel, Isk-related family, member 3 | Involved in AVNRT | Andreasen L, et al. 2018 |
| KCNE5 | ENSG00000176076.7 | Potassium voltage-gated channel subfamily E regulatory subunit 5 | Involved in arrhythmogenesis | [Abbott GW](https://www.ncbi.nlm.nih.gov/pubmed/?term=Abbott%20GW%5BAuthor%5D&cauthor=true&cauthor_uid=27484720). 2016 |
| KCNH2 | ENSG00000055118.15 | Potassium voltage-gated channel subfamily H member 2 | Involved in long QT syndrome; involved in AVNRT | Giudicessia JR, et al., 2018 Andreasen L, et al. 2018 |
| KCNJ12 | ENSG00000184185.5 | Inward rectifier K^+^ channels | Expressed in human atrioventricular conduction axis | Greener ID, et al. 2011 |
| KCNJ2 | ENSG00000123700.4 | Inward rectifier K^+^ channels | Expressed in human atrioventricular conduction axis | Greener ID, et al. 2011 |
| KCNJ3 | ENSG00000162989.3 | Inward rectifier K^+^ channels | Expressed in human atrioventricular conduction axis | Greener ID, et al. 2011 |
| KCNJ4 | ENSG00000168135.4 | Inward rectifier K^+^ channels | Expressed in human atrioventricular conduction axis | Greener ID, et al. 2011 |
| KCNJ5 | ENSG00000120457.7 | Inward rectifier K^+^ channels | Expressed in human atrioventricular conduction axis; Involved in long QT syndrome | Gr Greener ID, et al. 2011  Giudicessia JR, et al. 2018 |
| KCNN3 | ENSG00000143603.14 | Potassium intermediate/small conductance calcium-activated channel, subfamily N, member 3 | Involved in atrial fibrillation | Pfeufer A, et al. 2010 |
| KCNQ1 | ENSG00000053918.18 | Potassium voltage-gated channel subfamily Q member 1 | Involved in long QT syndrome | Giudicessia JR, et al. 2018 |
| KvLQT1 | ENSG00000269821.1 | Delayed rectifier K^+^ channels  KCNQ1 opposite strand/antisense transcript 1 | Expressed in human atrioventricular conduction axis | Greener ID, et al. 2011 |
| LMNA | ENSG00000160789.20 | Lamin A/C | Involved in dilated cardiomyopathy | McNally EM, et al. 2017 |
| MEIS1 | ENSG00000143995.15 | Transcription factor | Involved in AVNRT | Andreasen L, et al. 2018 |
| MYH6 | ENSG00000197616.12 | Myosin heavy chain 6 | Involved in dilated cardiomyopathy | McNally EM, et al. 2017 |
| NCX1 | ENSG00000183023.18 | Na^+^-Ca^2+^ exchanger  Solute carrier family 8 member A1 | Expressed in human atrioventricular conduction axis | Greener ID, et al. 2011 |
| NKX2-5 | ENSG00000183072.10 | Transcription factor | Associated with PR interval | Pfeufer A, et al. 2010 |
| NUP155 | ENSG00000113569.16 | Nucleoporin 155 | implicated in cardiovascular disorders | [Leonard RJ](https://www.ncbi.nlm.nih.gov/pubmed/?term=Leonard%20RJ%5BAuthor%5D&cauthor=true&cauthor_uid=32118046), et al. 2020 |
| PIAS3 | ENSG00000131788.16 | Protein inhibitor of activated STAT 3 | Involved in AVNRT | Andreasen L, et al. 2018 |
| PITX2 | ENSG00000164093.17 | Paired like homeodomain 2 | PITX2 gene is important in  cardiac development | Tsai CT, et al. 2008 |
| PKP2 | ENSG00000057294.14 | Plakophilin 2 | Involved in Brugada syndrome | Antzelevitch C, et al. 2016 |
| PLN | ENSG00000198523.6 | Phospholamban | Expressed in human atrioventricular conduction axis | Greener ID, et al. 2011 |
| PRKAG2 | ENSG00000106617.14 | Protein kinase AMP-activated non-catalytic subunit gamma 2 | Involved in dilated cardiomyopathy | McNally EM, et al. 2017 |
| RYR2 | ENSG00000198626.11 | Ca^2+^-handling proteins | Expressed in human atrioventricular conduction axis | Greener ID, et al. 2011 |
| RYR3 | ENSG00000198838.7 | Ca^2+^-handling proteins | Expressed in human atrioventricular conduction axis | Greener ID, et al. 2011 |
| SCN10A | ENSG00000185313.8 | Sodium voltage-gated channel alpha subunit 10 | Associated with PR interval | Pfeufer A, et al. 2010 |
| SCN1A | ENSG00000144285.11 | Na^+^ channels | Expressed in human atrioventricular conduction axis | Greener ID, et al. 2011 |
| SCN1B | ENSG00000105711.12 | Sodium voltage-gated channel beta subunit 1 | Involved in AVNRT；Expressed in human atrioventricular conduction axis | Andreasen L, et al. 2018；Greener ID, et al. 2011 |
| SCN2B | ENSG00000149575.6 | Sodium voltage-gated channel beta subunit 2 | Involved in AVNRT；Expressed in human atrioventricular conduction axis | Andreasen L, et al. 2018；Greener ID, et al. 2011 |
| SCN3A | ENSG00000153253.18 | Sodium voltage-gated channel alpha subunit 3 | Expressed in human atrioventricular conduction axis | Greener ID, et al. 2011 |
| SCN3B | ENSG00000166257.9 | Sodium voltage-gated channel beta subunit 3 | Involved in AVNRT；Expressed in human atrioventricular conduction axis | Andreasen L, et al. 2018；Greener ID, et al. 2011 |
| SCN4A | ENSG00000007314.7 | Sodium voltage-gated channel alpha subunit 4 | Expressed in human atrioventricular conduction axis | Greener ID, et al. 2011 |
| SCN4B | ENSG00000177098.8 | Sodium voltage-gated channel beta subunit 4 | Involved in long QT syndrome；Expressed in human atrioventricular conduction axis | Giudicessia JR, et al. 2018  Greener ID, et al. 2011 |
| SCN5A | ENSG00000183873.11 | Na+ channels | Expressed in human atrioventricular conduction axis | Greener ID, et al. 2011  Giudicessia JR, et al. 2018 |
| SCN8A | ENSG00000196876.9 | Na^+^ channels | Expressed in human atrioventricular conduction axis | Greener ID, et al. 2011 |
| SCN9A | ENSG00000169432.10 | Na^+^ channels | Expressed in human atrioventricular conduction axis | Greener ID, et al. 2011 |
| SERCA2a | ENSG00000174437.17 | ATPase sarcoplasmic/endoplasmic reticulum Ca^2+^ transporting 2 | Expressed in human atrioventricular conduction axis | Greener ID, et al. 2011 |
| SLN | ENSG00000170290.4 | Sarcolipin | Involved in atrial fibrillation and long QT syndrome. | Nyberg MT, et al. 2007 |
| SNTA1 | ENSG00000101400.6 | Syntrophin alpha 1 | Involved in long QT syndrome | Giudicessia Giudicessia JR, et al. 2018 2018 |
| SOX5 | ENSG00000134532.11 | Transcription factors | Associated with atrial fibrillation | Pfeufer A, et al. 2010 |
| SYNE2 | ENSG00000054654.17 | Spectrin repeat containing nuclear envelope protein 2 | Associated with atrial fibrillation | [Martin RI](https://www.ncbi.nlm.nih.gov/pubmed/?term=Martin%20RI%5BAuthor%5D&cauthor=true&cauthor_uid=26073630), et al. 2015 |
| SYNPO2L | ENSG00000166317.12 | Synaptopodin 2 like | Involved in atrial fibrillation | Lubitz SA, et al. 2016 |
| TBX3 | ENSG00000135111.10 | Transcription factor | Expressed in human atrioventricular conduction axis | Greener ID, et al. 2011 |
| TBX5 | ENSG00000089225.15 | Transcription factor | Expressed in atrioventricular and bundle branch conduction system | Pfeufer A, et al. 2010 |
| TRPM4 | ENSG00000130529.16 | Transient receptor potential cation channel subfamily M member 4 | Involved in Brugada syndrome | Antzelevitch C, et al. 2016 |
| WNT11 | ENSG00000085741.8 | Wnt family member 11 | Associated with PR interval | Pfeufer A, et al. 2010 |

***References***

*Greener ID , Monfredi O, Inada S, et al. Molecular architecture of the human specialised atrioventricular conduction axis. Journal of Molecular and Cellular Cardiology. 2011, 50(4): 642-651*

*Pfeufer A, Noord* ***C,*** *Marciante KD, et al. Genome-wide association study of PR interval. Nature genetics. 2010, 42(2):153-159*

*Andreasen L, Gustav Ahlberg G, Tang C, et al. Next-generation sequencing of AV nodal reentrant tachycardia patients identifies broad spectrum of variants in ion channel genes. European Journal of Human Genetics. 2018, 26(5):660-668*

*Giudicessia JR, Wilde AA, Ackermand MJ. The genetic architecture of long QT syndrome: A critical reappraisal. Trends Cardiovasc Med. 2018, 28(7): 453-464.*

*Tsai CT，Lai LP, Hwang JJ, et al., molecular genetics of atrial fibrillation. Journal of the American College of Cardiology. 2008,52(4):241-250*

*Schreieck J, Dostal S, von Beckerath N, et al. C825T polymorphism of the G-protein beta3 subunit gene and atrial fibrillation: association of the TT genotype with a reduced risk for atrial fibrillation. Am Heart J 2004, 148(3):545-50.*

*Nyberg MT, Stoevring B, Behr ER, et al., The variation of the sarcolipin gene (SLN) in atrial fibrillation, long QT syndrome and sudden arrhythmic death syndrome. Clin Chim Acta. 2007, 375(1-2):87-91.*

*Antzelevitch C, Patocskai B. Brugada Syndrome: Clinical, Genetic, Molecular, Cellular, and Ionic Aspects. Curr Probl Cardiol. 2016, 41(1):7-57.*

*Martin RI, Babaei MS, Choy MK, et al., Genetic variants associated with risk of atrial fibrillation regulate expression of PITX2, CAV1, MYOZ1, C9orf3 and FANCC.J Mol Cell Cardiol. 2015, 85:207-214.*

*Peterkin T, Gibson A, Loose M, et al., The roles of GATA-4, -5 and -6 in vertebrate heart development.Semin Cell Dev Biol. 2005,16(1):83-94.*

*Hu J, Gao C, Wei C, et al., RBFox2-miR-34a-Jph2 axis contributes to cardiac decompensation during heart failure. Proc Natl Acad Sci U S A. 2019,116(13):6172-6180.*

*McNally EM, Mestroni L. Dilated Cardiomyopathy: Genetic Determinants and Mechanisms. Circ Res. 2017, 121(7):731-748.*

*Abbott GW. KCNE4 and KCNE5: K(+) channel regulation and cardiac arrhythmogenesis. Gene. 2016, 593(2):249-260.*

*Lubitz SA, Brody JA, Bihlmeyer NA, et al., Whole Exome Sequencing in Atrial Fibrillation. PLoS Genet. 2016,12(9):e1006284.*

*Leonard RJ, Preston CC, Gucwa ME, et al. Protein Subdomain Enrichment of NUP155 Variants Identify a Novel Predicted Pathogenic Hotspot. Front Cardiovasc Med. 2020,7:8.*
