# Supplementary material for: Identification of potential candidate genes and pathways in atrioventricular nodal reentry tachycardia by whole‐exome sequencing
Source: Clin Transl Med. 2020 Apr 30;10(1):238–57. doi: 10.1002/ctm2.25 (PMC7240861; doi:10.1002/ctm2.25)
Supplement: Supplementary file 2 — Supporting Information S1 [file CTM2-10-238-s006.docx]

**S2: Rare variants of reference genes (MAF<0.001)**

| **Gene name** | **Transcription impact** | **Transcript** | **Hgv. p** | **Hgv. c** | **MAF KEGG EAS** | **MAF ExAC EAS** | **Cases** | **Mutation Type** |
| --- | --- | --- | --- | --- | --- | --- | --- | --- |
| KCND3 | missense | NM_004980.4 | p.Asn648Ser | c.1943A>G | . | . | 1 | het |
| CASQ2 | missense | NM_001232.3 | p.Val133Ala | c.398T>C | . | . | 1 | het |
| KCNN3 | missense | NM_001204087.1 | p.Gln76Arg | c.227A>G | . | . | 1 | hom |
| HCN3 | missense | NM_020897.2 | p.Arg710His | c.2129G>A | 0 | 0 | 1 | het |
| LMNA | missense | NM_170707.3 | p.Arg336Gln | c.1007G>A | . | 0 | 1 | het |
| RYR2 | missense | XM_005273224.1 | p.Asp1048Gly | c.3143A>G | . | . | 1 | het |
| RYR2 | missense | XM_005273224.1 | p.Thr1228Asn | c.3683C>A | . | . | 1 | het |
| RYR2 | missense | XM_005273224.1 | p.Pro1857Leu | c.5570C>T | 0 | 0.000116686 | 1 | het |
| RYR2 | missense | XM_005273224.1 | p.Ile1925Thr | c.5774T>C | . | 0 | 1 | het |
| RYR2 | missense | XM_005273224.1 | p.Asp2014Tyr | c.6040G>T | . | . | 1 | het |
| RYR2 | missense | XM_005273224.1 | p.Ser2031Phe | c.6092C>T | . | 0.000116117 | 1 | het |
| RYR2 | missense | XM_005273224.1 | p.Arg2359Gln | c.7076G>A | . | 0.000700117 | 1 | het |
| RYR2 | missense | XM_005273224.1 | p.Ile3784Met | c.11352T>G | . | . | 1 | het |
| SLC8A1 | missense | XM_005264512.1 | p.Ser555Pro | c.1663T>C | . | . | 1 | het |
| SCN3A | missense | NM_006922.3 | p.Arg1023Trp | c.3067C>T | . | 0.000231428 | 1 | het |
| SCN3A | missense | NM_006922.3 | p.Arg520Thr | c.1559G>C | . | 0.000924642 | 1 | het |
| SCN3A | missense | NM_006922.3 | p.Ala107Val | c.320C>T | . | . | 1 | het |
| SCN1A | missense | NM_001165963.1 | p.Asp1059Val | c.3176A>T | . | 0.000351617 | 1 | het |
| SCN1A | missense | NM_001165963.1 | p.Arg1018Lys | c.3053G>A | . | 0.000232234 | 1 | het |
| SCN1A | missense | NM_001165963.1 | p.Met714Arg | c.2141T>G | . | . | 1 | het |
| SCN9A | missense | XM_005246757.1 | p.Ala1044Gly | c.3131C>G | . | . | 1 | het |
| ITPR1 | missense | XM_005265108.1 | p.Gly1313Ser | c.3937G>A | . | 0 | 1 | het |
| CAV3 | missense | NM_001234.4 | p.Gly56Ser | c.166G>A | 0 | 0.000578436 | 1 | het |
| SCN5A | missense | NM_001099404.1 | p.Arg1944Gln | c.5831G>A | . | 0.000120019 | 1 | het |
| SCN5A | missense | NM_001099404.1 | p.Arg433Cys | c.1297C>T | . | 0.000232126 | 1 | het |
| SCN5A | missense | NM_001099404.1 | p.Ala344Val | c.1031C>T | . | . | 1 | het |
| SCN5A | missense | NM_001099404.1 | p.Ala166Thr | c.496G>A | . | 0 | 1 | het |
| SCN10A | missense | XM_005265371.1 | p.Thr875Met | c.2624C>T | . | 0 | 1 | het |
| SCN10A | missense | XM_005265371.1 | p.Asn334Lys | c.1002C>G | . | . | 1 | het |
| CACNA1D | missense | NM_000720.3 | p.Gln67Lys | c.199C>A | . | . | 1 | het |
| CACNA1D | missense | NM_000720.3 | p.Tyr1827Asn | c.5479T>A | . | . | 1 | het |
| CACNA1D | missense | NM_000720.3 | p.Arg1949His | c.5846G>A | . | 0 | 1 | het |
| CACNA1D | missense | NM_000720.3 | p.Ala2078Val | c.6233C>T | . | 0 | 1 | het |
| PITX2 | missense | NM_000325.5 | p.Glu66Gly | c.197A>G | . | 0 | 1 | het |
| ANK2 | missense | XM_005262941.1 | p.Arg411Cys | c.1231C>T | 0 | 0.000115554 | 1 | het |
| ANK2 | missense | XM_005262941.1 | p.Leu700Met | c.2098C>A | . | . | 1 | het |
| ANK2 | missense | XM_005262941.1 | p.Ser1984Leu | c.5951C>T | . | . | 1 | het |
| ANK2 | missense | XM_005262941.1 | p.Val2421Ile | c.7261G>A | . | . | 1 | het |
| ANK2 | missense | XM_005262941.1 | p.Arg3466Ser | c.10398A>T | . | . | 1 | hom |
| ANK2 | missense | XM_005262941.1 | p.Arg3874Trp | c.11620C>T | 0 | 0.000116496 | 1 | het |
| NUP155 | missense | NM_153485.2 | p.Ile1376Asn | c.4127T>A | . | . | 1 | het |
| NUP155 | frameshift | NM_153485.2 | p.Gln1365fs | c.4093_4094delCA | . | . | 1 | het |
| ADRB2 | missense | XM_005268382.1 | p.Asn69Ser | c.206A>G | 0 | 0.00023218 | 1 | het |
| AKAP9 | missense | XM_005250102.1 | p.Glu104Gly | c.311A>G | . | . | 1 | het |
| AKAP9 | missense | XM_005250102.1 | p.Asp920Asn | c.2758G>A | . | . | 1 | het |
| AKAP9 | missense | XM_005250102.1 | p.Lys1053Asn | c.3159A>C | . | . | 1 | het |
| AKAP9 | missense | XM_005250102.1 | p.Thr3193Met | c.9578C>T | . | . | 1 | het |
| AKAP9 | missense | XM_005250102.1 | p.Thr3513Ala | c.10537A>G | . | . | 1 | het |
| CAV1 | missense | NM_001753.4 | p.Val152Ile | c.454G>A | . | . | 1 | het |
| KCNH2 | missense | NM_000238.3 | p.Thr1072Met | c.3215C>T | . | 0 | 1 | het |
| KCNH2 | missense | NM_000238.3 | p.Pro923Leu | c.2768C>T | . | 0 | 1 | het |
| KCNH2 | missense | NM_000238.3 | p.Arg892Cys | c.2674C>T | 0 | 0.000237756 | 1 | het |
| PRKAG2 | missense | XM_005250002.1 | p.Arg84Trp | c.250C>T | 0 | 0 | 1 | het |
| C9orf3 | missense | NM_001193329.1 | p.Gly233Arg | c.697G>A | . | . | 1 | het |
| CACNB2 | missense | NM_201596.2 | p.Leu480Val | c.1438T>G | . | . | 1 | het |
| CACNB2 | missense | NM_201596.2 | p.Arg614Gln | c.1841G>A | . | 0 | 1 | het |
| SYNPO2L | frameshift | NM_001114133.1 | p.Pro708fs | c.2120dupC | . | . | 1 | het |
| SYNPO2L | frameshift | NM_001114133.1 | p.Ala25fs | c.73delG | . | . | 1 | het |
| KCNA4 | missense | NM_002233.3 | p.Arg83Trp | c.247C>T | . | 0 | 1 | het |
| SCN4B | missense | NM_174934.3 | p.Gly214Ser | c.640G>A | . | 0.000809436 | 1 | het |
| KCNA5 | missense | NM_002234.3 | p.Arg54Ser | c.160C>A | . | . | 1 | het |
| GNB3 | missense | NM_002075.2 | p.Ile262Thr | c.785T>C | . | 0.000115929 | 1 | het |
| GNB3 | missense | NM_002075.2 | p.Ser279Cys | c.836C>G | . | . | 1 | het |
| GNB3 | missense | NM_002075.2 | p.Ala287Ser | c.859G>T | . | 0.000347222 | 2 | het,het |
| GNB3 | missense | NM_002075.2 | p.Leu308Phe | c.922C>T | . | . | 1 | het |
| SOX5 | missense | NM_006940.4 | p.Thr494Ile | c.1481C>T | . | 0.000115794 | 1 | het |
| PKP2 | missense | NM_004572.3 | p.Arg101Cys | c.301C>T | . | . | 1 | het |
| TBX3 | missense | NM_016569.3 | p.Gly672Asp | c.2015G>A | . | . | 1 | het |
| MYH6 | missense | XM_005267694.1 | p.Arg1967Cys | c.5899C>T | . | . | 1 | het |
| MYH6 | missense | XM_005267694.1 | p.Arg1739Trp | c.5215C>T | . | 0.00011558 | 1 | het |
| MYH6 | missense | XM_005267694.1 | p.Val933Met | c.2797G>A | . | . | 1 | het |
| MYH6 | missense | XM_005267694.1 | p.Ala809Val | c.2426C>T | . | 0.00058126 | 1 | het |
| SYNE2 | missense | XM_005267454.1 | p.Ser334Pro | c.1000T>C | . | . | 1 | het |
| SYNE2 | missense | XM_005267454.1 | p.Ala1033Thr | c.3097G>A | . | 0.000468823 | 1 | het |
| SYNE2 | missense | XM_005267454.1 | p.Glu1690Gly | c.5069A>G | . | 0 | 1 | het |
| SYNE2 | missense | XM_005267454.1 | p.Ala6735Thr | c.20203G>A | . | 0.000577901 | 1 | het |
| SYNE2 | missense | XM_005267454.1 | p.Glu6778Lys | c.20332G>A | . | . | 1 | het |
| RYR3 | missense | NM_001036.3 | p.Ile504Thr | c.1511T>C | . | . | 1 | het |
| RYR3 | missense | NM_001036.3 | p.Ala651Thr | c.1951G>A | . | 0 | 1 | het |
| RYR3 | missense | NM_001036.3 | p.Arg3356Gly | c.10066C>G | . | . | 1 | het |
| RYR3 | missense | NM_001036.3 | p.Leu3571Trp | c.10712T>G | . | . | 1 | het |
| RYR3 | missense | NM_001036.3 | p.Glu3796Gln | c.11386G>C | . | 0.000582479 | 1 | het |
| RYR3 | missense | NM_001036.3 | p.Asn3801Ser | c.11402A>G | . | . | 1 | het |
| RYR3 | missense | NM_001036.3 | p.Thr3955Met | c.11864C>T | . | 0 | 1 | het |
| RYR3 | missense | NM_001036.3 | p.Ala4127Val | c.12380C>T | . | 0 | 1 | het |
| RYR3 | missense | NM_001036.3 | p.Ser4135Phe | c.12404C>T | . | . | 1 | het |
| HCN4 | missense | NM_005477.2 | p.Arg1102His | c.3305G>A | 0 | 0 | 1 | het |
| HCN4 | missense | NM_005477.2 | p.Ile1086Leu | c.3256A>C | . | . | 1 | het |
| HCN4 | missense | NM_005477.2 | p.Arg612His | c.1835G>A | 0 | 0.000115554 | 1 | het |
| ZFHX3 | missense | NM_006885.3 | p.Gly3692Ala | c.11075G>C | . | . | 1 | het |
| ZFHX3 | frameshift | NM_006885.3 | p.Lys3689fs | c.11064delC | . | . | 1 | het |
| ZFHX3 | missense | NM_006885.3 | p.Lys3390Glu | c.10168A>G | . | . | 1 | het |
| ZFHX3 | missense | NM_006885.3 | p.Ser2379Thr | c.7136G>C | . | . | 1 | het |
| ZFHX3 | missense | NM_006885.3 | p.Gly761Val | c.2282G>T | . | 0.000116877 | 1 | het |
| ZFHX3 | missense | NM_006885.3 | p.Glu593Lys | c.1777G>A | . | 0.000115554 | 1 | het |
| ZFHX3 | missense | NM_006885.3 | p.Pro71Leu | c.212C>T | . | . | 1 | het |
| KCNJ12 | missense | NM_021012.4 | p.Val36Leu | c.106G>T | . | 0.000463392 | 1 | het |
| KCNJ12 | missense | NM_021012.4 | p.Ser88Leu | c.263C>T | . | 0.00023116 | 1 | het |
| KCNJ12 | missense | NM_021012.4 | p.Ile138Val | c.412A>G | . | . | 1 | het |
| KCNJ12 | missense | NM_021012.4 | p.Ala158Val | c.473C>T | . | 0 | 1 | het |
| KCNJ12 | missense | NM_021012.4 | p.Val168Met | c.502G>A | . | 0.000925712 | 1 | het |
| KCNJ12 | missense | NM_021012.4 | p.Ala230Thr | c.688G>A | 0 | 0.000231214 | 1 | het |
| KCNJ12 | missense | NM_021012.4 | p.Leu232Phe | c.694C>T | . | . | 1 | het |
| KCNJ12 | missense | NM_021012.4 | p.Arg326His | c.977G>A | 0 | 0 | 1 | het |
| KCNJ12 | missense | NM_021012.4 | p.Glu380Lys | c.1138G>A | . | 0 | 1 | het |
| KCNJ12 | missense | NM_021012.4 | p.Arg429Gln | c.1286G>A | . | . | 1 | het |
| KCNJ12 | missense | NM_021012.4 | p.Glu430Lys | c.1288G>A | . | . | 1 | het |
| GJD3 | frameshift | NM_152219.3 | p.Glu258fs | c.749_770dupTGCCCTCCCGGCGCCCCGGCCC | . | . | 1 | het |
| GJD3 | frameshift | NM_152219.3 | p.Cys125fs | c.372_387delGTGCGCCCTGCGCGCC | . | . | 1 | het |
| CACNA1G | missense | NM_018896.4 | p.Ala1238Val | c.3713C>T | . | 0 | 1 | het |
| CACNA1G | missense | NM_018896.4 | p.Thr1811Ala | c.5431A>G | . | . | 1 | het |
| SCN4A | missense | NM_000334.4 | p.Pro72Leu | c.215C>T | . | . | 1 | het |
| SCN4A | missense | NM_000334.4 | p.Thr21Pro | c.61A>C | . | . | 1 | het |
| HCN2 | missense | NM_001194.3 | p.Pro794Thr | c.2380C>A | . | . | 1 | hom |
| SCN1B | missense | NM_199037.3 | p.Gly257Arg | c.769G>A | 0 | 0 | 1 | het |
| TRPM4 | missense | NM_017636.3 | p.Gly163Cys | c.487G>T | . | 0 | 1 | het |
| TRPM4 | missense | NM_017636.3 | p.Thr239Ile | c.716C>T | . | . | 1 | het |
| TRPM4 | missense | NM_017636.3 | p.Arg250Cys | c.748C>T | . | 0 | 1 | het |
| TRPM4 | missense | NM_017636.3 | p.Ser546Leu | c.1637C>T | . | 0.000116469 | 1 | het |
| TRPM4 | missense | NM_017636.3 | p.His774Tyr | c.2320C>T | . | . | 1 | het |
| SNTA1 | missense | NM_003098.2 | p.Thr246Ile | c.737C>T | . | . | 1 | het |
| ERG | missense | NM_001136154.1 | p.Pro411Ala | c.1231C>G | . | 0.000578035 | 1 | het |
| CACNA1I | missense | NM_021096.3 | p.Arg418Cys | c.1252C>T | . | . | 1 | het |
| CACNA1I | missense | NM_021096.3 | p.Ile1343Thr | c.4028T>C | . | . | 1 | het |
| CACNA1I | missense | NM_021096.3 | p.Pro2000Ala | c.5998C>G | . | . | 1 | het |
| CACNA1I | missense | NM_021096.3 | p.His2123Arg | c.6368A>G | . | . | 1 | het |

*Note: KEGG, Kyoto Encyclopedia of Genes and Genomes；ExAC, Exome Aggregation Consortium; EAS, East Asian; MAF, Minor allele frequency.*
