# Supplementary material for: Identification of potential candidate genes and pathways in atrioventricular nodal reentry tachycardia by whole‐exome sequencing
Source: Clin Transl Med. 2020 Apr 30;10(1):238–57. doi: 10.1002/ctm2.25 (PMC7240861; doi:10.1002/ctm2.25)
Supplement: Supplementary file 4 — Supporting Information S3 [file CTM2-10-238-s008.doc]

**S4: SNPs in GWAS analysis (P<10-E6)**

| **CHR** | **SNP** | **A1** | **FA** | **FU** | **A2** | **P** | **OR** | **Variant Type** | **Gene Symbol** | **Functions** | **Transcript** | **Hgv.p** | **Hgv.c** | **MAF**  **KEGG EAS** | **MAF**  **ExAC EAS** |
| --- | --- | --- | --- | --- | --- | --- | --- | --- | --- | --- | --- | --- | --- | --- | --- |
| 12 | 12:54405179:D:1 | A | 0.402 | 0.071 | AC | 1.60E-14 | 8.85 | InDel | HOXC8 | 3_prime_UTR_variant | NM_022658.3 | . | c.*15delC | . | . |
| 15 | 15:25932718:D:66 | A | 0.315 | 0.030 | ACAGGCGAAGGAGGATGGAGCAGGTTGTCCATGGCCTCCCATCTGCTCCATCCTCCTTCGCCTCATT | 3.35E-14 | 14.70 | InDel | ATP10A | intron_variant | NM_024490.3 | . | c.3291+66_3291+131delAATGAGGCGAAGGAGGATGGAGCAGATGGGAGGCCATGGACAACCTGCTCCATCCTCCTTCGCCTG | . | . |
| 19 | 19:1775123:I:4 | TCGCC | 0.290 | 0.036 | T | 1.25E-11 | 11.03 | InDel | ONECUT3 | intron_variant | NM_001080488.1 | . | c.1193-29_1193-28insCGCC | . | 0 |
| 4 | 4:108935771:C:T | C | 0.342 | 0.070 | T | 7.09E-11 | 6.89 | SNP | HADH | intron_variant | NM_001184705.2 | . | c.419+27T>C | 0.002 | 0.142986153 |
| 14 | 14:58907006:D:2 | T | 0.323 | 0.065 | TTC | 1.71E-10 | 6.87 | InDel | KIAA0586 | intron_variant | NM_001244189.1 | . | c.447-932_447-931delTC | . | . |
| 17 | 17:74017554:T:G | T | 0.031 | 0.260 | G | 2.54E-10 | 0.09 | SNP | EVPL | missense_variant | XM_005257143.1 | p.Arg336Ser | c.1006C>A | . | 0.165170132 |
| 7 | 7:142468213:G:A | G | 0.037 | 0.270 | A | 2.80E-10 | 0.10 | SNP | TRB | intragenic_variant | TRB | . | n.142468213A>G | . | . |
| 1 | 1:150230422:D:2 | G | 0.329 | 0.075 | GCT | 5.69E-10 | 6.06 | InDel | CA14 | non_coding_transcript_exon_variant | XR_241076.1 | . | n.870_871delCT | . | . |
| 10 | 10:38990771:G:C | G | 0.335 | 0.067 | C | 9.34E-10 | 7.02 | SNP | ACTR3BP5 | non_coding_transcript_exon_variant | NR_045000.1 | . | n.1045C>G | . | . |
| 7 | 7:142468205:C:A | C | 0.049 | 0.285 | A | 1.03E-09 | 0.13 | SNP | TRB | intragenic_variant | TRB | . | n.142468205A>C | . | . |
| 11 | 11:1018266:A:G | A | 0.305 | 0.065 | G | 1.51E-09 | 6.31 | SNP | MUC6 | missense_variant | NM_005961.2 | p.Ala1512Val | c.4535C>T | . | 0.000115929 |
| 10 | 10:38990765:A:G | A | 0.335 | 0.075 | G | 1.79E-09 | 6.25 | SNP | ACTR3BP5 | non_coding_transcript_exon_variant | NR_045000.1 | . | n.1039G>A | . | . |
| 11 | 11:1018303:A:G | A | 0.232 | 0.030 | G | 2.33E-09 | 9.75 | SNP | MUC6 | missense_variant | NM_005961.2 | p.Pro1500Ser | c.4498C>T | . | . |
| 20 | 20:61987512:D:22 | G | 0.161 | 0.000 | GGGGCAGGGGCAGGGCCAGGGCA | 3.05E-09 | NaN | InDel | CHRNA4 | intron_variant | NM_000744.6 | . | c.274-98_274-77delTGCCCTGGCCCTGCCCCTGCCC | . | . |
| 11 | 11:1018256:G:T | G | 0.299 | 0.065 | T | 3.08E-09 | 6.13 | SNP | MUC6 | synonymous_variant | NM_005961.2 | p.Ser1515Ser | c.4545A>C | . | 0.000115929 |
| 10 | 10:38990769:C:T | C | 0.335 | 0.073 | T | 3.09E-09 | 6.39 | SNP | ACTR3BP5 | non_coding_transcript_exon_variant | NR_045000.1 | . | n.1043T>C | . | . |
| 10 | 10:38990768:G:T | G | 0.335 | 0.073 | T | 3.09E-09 | 6.39 | SNP | ACTR3BP5 | non_coding_transcript_exon_variant | NR_045000.1 | . | n.1042T>G | . | . |
| 17 | 17:74017573:G:C | G | 0.037 | 0.255 | C | 3.89E-09 | 0.11 | SNP | EVPL | synonymous_variant | XM_005257143.1 | p.Leu329Leu | c.987G>C | . | 0.110500379 |
| 1 | 1:84956161:I:1 | CT | 0.323 | 0.085 | C | 9.40E-09 | 5.14 | InDel | RPF1 | splice_region_variant | NM_025065.6 | . | c.616+6_616+7insT | 0.184 | . |
| 23 | 23:51075841:G:A | G | 0.000 | 0.145 | A | 1.33E-08 | 0.00 | SNP | NUDT10 | synonymous_variant | NM_153183.2 | p.Thr8Thr | c.24A>G | . | 0.352039203 |
| 23 | 23:24665391:D:2 | C | 0.191 | 0.017 | CCT | 2.28E-08 | 13.96 | InDel | PCYT1B | intron_variant | NM_001163264.1 | . | c.63+25294_63+25295delAG | . | . |
| 11 | 11:1018272:G:C | G | 0.177 | 0.015 | C | 2.45E-08 | 14.11 | SNP | MUC6 | missense_variant | NM_005961.2 | p.Ser1510Thr | c.4529G>C | . | . |
| 3 | 3:160134208:I:1 | GT | 0.329 | 0.095 | G | 3.46E-08 | 4.68 | InDel | SMC4 | splice_region_variant | NM_001002800.1 | . | c.1437+5_1437+6insT | . | . |
| 6 | 6:32605204:G:A | G | 0.134 | 0.000 | A | 3.80E-08 | NaN | SNP | HLA-DQA1 | 5_prime_UTR_variant | NM_002122.3 | . | c.-32A>G | 0.053 | 0.009651076 |
| 23 | 23:72433663:D:3 | G | 0.198 | 0.025 | GTCC | 4.21E-08 | 9.60 | InDel | NAP1L2 | disruptive_inframe_deletion | NM_021963.3 | p.Glu221del | c.663_665delGGA | 0.007 | . |
| 17 | 17:74017594:G:A | G | 0.037 | 0.230 | A | 5.21E-08 | 0.13 | SNP | EVPL | synonymous_variant | XM_005257143.1 | p.Cys322Cys | c.966T>C | . | 0.030033203 |
| 16 | 16:14738116:I:1 | GT | 0.179 | 0.016 | G | 6.38E-08 | 13.74 | InDel | BFAR | intron_variant | NM_016561.2 | . | c.-73-15_-73-14insT | . | . |
| 7 | 7:142032786:I:1 | AC | 0.317 | 0.092 | A | 9.63E-08 | 4.59 | InDel | TRBV6-1 | downstream_gene_variant | TRANSCRIPT_TRBV6-1 | . | c.*4176_*4177insC | . | . |
| 7 | 7:142032788:G:T | G | 0.317 | 0.092 | T | 9.63E-08 | 4.59 | SNP | TRBV6-1 | downstream_gene_variant | TRANSCRIPT_TRBV6-1 | . | c.*4178T>G | . | . |
| 14 | 14:23924767:I:1 | AT | 0.281 | 0.566 | A | 1.59E-07 | 0.30 | InDel | MYH7-NGDN | intergenic_region | MYH7-NGDN | . | n.23924768delT | 0.639 | . |
| 15 | 15:76054835:T:C | T | 0.274 | 0.064 | C | 1.59E-07 | 5.54 | SNP | LOC101929408 | intron_variant | XR_243186.1 | . | n.188+67C>T | . | . |
| 17 | 17:16097899:T:C | T | 0.037 | 0.220 | C | 1.78E-07 | 0.14 | SNP | NCOR1 | 5_prime_UTR_variant | XM_005256866.1 | . | c.-16G>A | . | 0.310687891 |
| 15 | 15:76248427:I:1 | GT | 0.256 | 0.060 | G | 1.99E-07 | 5.39 | InDel | NRG4 | intron_variant | NM_138573.3 | . | c.252-76delA | . | . |
| 9 | 9:33799285:C:T | C | 0.018 | 0.180 | T | 2.04E-07 | 0.08 | SNP | LOC101929665 | intron_variant | XR_242542.1 | . | n.1145+500A>G | . | . |
| 15 | 15:69715487:D:1 | T | 0.244 | 0.055 | TC | 2.43E-07 | 5.54 | InDel | KIF23 | intron_variant | XM_005254796.1 | . | c.564-10delC | 0.265 | 0.222541691 |
| 1 | 1:205615947:C:A | C | 0.274 | 0.071 | A | 2.69E-07 | 4.97 | SNP | ELK4-SLC45A3 | intergenic_region | ELK4-SLC45A3 | . | n.205615947C>A | 0.999 | 0.533898305 |
| 6 | 6:167708226:G:T | G | 0.030 | 0.200 | T | 3.07E-07 | 0.13 | SNP | UNC93A | intron_variant | NM_018974.3 | . | c.269+40T>G | . | . |
| 11 | 11:1018281:A:C | A | 0.183 | 0.025 | C | 3.37E-07 | 8.73 | SNP | MUC6 | missense_variant | NM_005961.2 | p.Ser1507Ile | c.4520G>T | . | . |
| 19 | 19:54573523:I:1 | GT | 0.244 | 0.056 | G | 3.98E-07 | 5.48 | InDel | TARM1 | intron_variant | XM_005258952.1 | . | c.683-110dupA | . | . |
| 15 | 15:69715488:C:T | C | 0.250 | 0.061 | T | 4.09E-07 | 5.17 | SNP | KIF23 | intron_variant | XM_005254796.1 | . | c.564-10C>T | 1.000 | 0.765034014 |
| 13 | 13:41301768:I:1 | GA | 0.244 | 0.056 | G | 4.29E-07 | 5.42 | InDel | MIR320D1 | downstream_gene_variant | NR_031723.1 | . | n.*195_*196insT | . | . |
| 7 | 7:142032796:C:T | C | 0.272 | 0.071 | T | 4.54E-07 | 4.91 | SNP | TRBV6-1 | downstream_gene_variant | TRANSCRIPT_TRBV6-1 | . | c.*4186T>C | . | . |
| 6 | 6:32487310:A:G | A | 0.234 | 0.051 | G | 4.63E-07 | 5.69 | SNP | HLA-DRB5 | synonymous_variant | NM_002125.3 | p.Asn163Asn | c.489C>T | . | 0.204707379 |
| 3 | 3:12944143:I:1 | CA | 0.226 | 0.050 | C | 6.04E-07 | 5.54 | InDel | IQSEC1 | intron_variant | XM_005265623.1 | . | c.2850+129dupT | . | . |
| 12 | 12:27803097:D:2 | C | 0.293 | 0.090 | CCT | 6.12E-07 | 4.18 | InDel | PPFIBP1 | intron_variant | XM_005253505.1 | . | c.603+24_603+25delCT | . | . |
| 6 | 6:33054550:G:A | G | 0.348 | 0.617 | A | 6.44E-07 | 0.33 | SNP | HLA-DPB1 | 3_prime_UTR_variant | NM_002121.5 | . | c.*239G>A | 0.618 | . |
| 6 | 6:33054552:C:G | C | 0.348 | 0.617 | G | 6.44E-07 | 0.33 | SNP | HLA-DPB1 | 3_prime_UTR_variant | NM_002121.5 | . | c.*241C>G | 0.618 | . |
| 14 | 14:64066453:A:G | A | 0.177 | 0.025 | G | 6.83E-07 | 8.38 | SNP | WDR89 | missense_variant | NM_001008726.2 | p.Pro70Ser | c.208C>T | . | . |
| 14 | 14:64066451:G:A | G | 0.177 | 0.025 | A | 6.83E-07 | 8.38 | SNP | GCATP1 | upstream_gene_variant | TRANSCRIPT_GCATP1 | . | n.-4407T>C | . | 0 |
| 14 | 14:64066398:C:G | C | 0.195 | 0.035 | G | 7.66E-07 | 6.68 | SNP | WDR89 | missense_variant | NM_001008726.2 | p.Ala88Gly | c.263C>G | . | . |
| 14 | 14:64066402:G:A | G | 0.195 | 0.035 | A | 7.66E-07 | 6.68 | SNP | WDR89 | missense_variant | NM_001008726.2 | p.Ser87Pro | c.259T>C | . | . |
| 14 | 14:64066394:G:A | G | 0.195 | 0.035 | A | 7.66E-07 | 6.68 | SNP | GCATP1 | upstream_gene_variant | TRANSCRIPT_GCATP1 | . | n.-4350T>C | . | . |
| 1 | 1:12888371:G:T | G | 0.189 | 0.030 | T | 7.73E-07 | 7.54 | SNP | PRAMEF11 | missense_variant | NM_001146344.1 | p.Gln51His | c.153A>C | . | 0.229098806 |
| 14 | 14:64066379:T:C | T | 0.189 | 0.030 | C | 7.73E-07 | 7.54 | SNP | GCATP1 | upstream_gene_variant | TRANSCRIPT_GCATP1 | . | n.-4335G>A | . | 0 |
| 19 | 19:58385762:G:C | G | 0.000 | 0.115 | C | 7.74E-07 | 0.00 | SNP | ZNF814 | synonymous_variant | NM_001144989.1 | p.Ser332Ser | c.996G>C | . | 0.27247191 |
| 9 | 9:85597727:D:3 | A | 0.354 | 0.135 | AGAG | 1.01E-06 | 3.51 | InDel | RASEF | intron_variant | NM_152573.3 | . | c.2118-33_2118-31delCTC | 0.039 | . |
| 19 | 19:51856728:T:C | T | 0.111 | 0.000 | C | 1.06E-06 | NaN | SNP | ETFB | intron_variant | NM_001014763.1 | . | c.490-184G>A | . | . |
| 12 | 12:31244703:T:C | T | 0.037 | 0.205 | C | 1.09E-06 | 0.15 | SNP | DDX11 | synonymous_variant | XM_005253331.1 | p.Ala416Ala | c.1248C>T | . | 0.026726058 |
| 7 | 7:142499854:C:T | C | 0.006 | 0.135 | T | 1.12E-06 | 0.04 | SNP | TRBJ2-6 | downstream_gene_variant | TRANSCRIPT_TRBJ2-6 | . | c.*4879T>C | . | 0.043290043 |
| 7 | 7:142499862:T:C | T | 0.006 | 0.130 | C | 1.17E-06 | 0.04 | SNP | TRBJ2-6 | downstream_gene_variant | TRANSCRIPT_TRBJ2-6 | . | c.*4887C>T | . | 0.0418429 |
| 7 | 7:142499874:C:T | C | 0.006 | 0.130 | T | 1.17E-06 | 0.04 | SNP | TRBJ2-6 | downstream_gene_variant | TRANSCRIPT_TRBJ2-6 | . | c.*4899T>C | . | 0.00207216 |
| 18 | 18:60382986:D:42 | C | 0.136 | 0.010 | CCGGCGGCCGCCGCTGCGGCAGCAGCAGCAGCAGCGGCGGCCG | 1.25E-06 | 15.56 | InDel | PHLPP1 | conservative_inframe_deletion | NM_194449.3 | p.Ala25_Ala38del | c.71_112delCGGCGGCCGCCGCTGCGGCAGCAGCAGCAGCAGCGGCGGCCG | . | . |
| 9 | 9:68744062:G:A | G | 0.136 | 0.010 | A | 1.25E-06 | 15.56 | SNP | LOC100132352 | intron_variant | NR_034006.1 | . | n.817+832A>G | . | . |
| 11 | 11:94040969:I:10 | AGGGACTGGGT | 0.000 | 0.113 | A | 1.26E-06 | 0.00 | InDel | FOLR4 | downstream_gene_variant | NM_001199206.1 | . | c.*111_*112insGGGACTGGGT | . | . |
| 7 | 7:39379287:D:3 | C | 0.201 | 0.040 | CCAG | 1.32E-06 | 6.05 | InDel | POU6F2 | conservative_inframe_deletion | NM_007252.3 | p.Gln187del | c.559_561delCAG | . | . |
| 11 | 11:1018248:A:G | A | 0.134 | 0.010 | G | 1.35E-06 | 15.34 | SNP | MUC6 | missense_variant | NM_005961.2 | p.Thr1518Ile | c.4553C>T | . | . |
| 14 | 14:64066471:A:G | A | 0.134 | 0.010 | G | 1.35E-06 | 15.34 | SNP | GCATP1 | upstream_gene_variant | TRANSCRIPT_GCATP1 | . | n.-4427C>T | . | 0 |
| 9 | 9:33799304:C:G | C | 0.018 | 0.160 | G | 1.44E-06 | 0.10 | SNP | LOC101929665 | intron_variant | XR_242542.1 | . | n.1145+481C>G | . | . |
| 14 | 14:64066431:T:C | T | 0.183 | 0.030 | C | 1.50E-06 | 7.24 | SNP | WDR89 | missense_variant | NM_001008726.2 | p.Arg77Lys | c.230G>A | . | . |
| 14 | 14:64066424:C:T | C | 0.183 | 0.030 | T | 1.50E-06 | 7.24 | SNP | GCATP1 | upstream_gene_variant | TRANSCRIPT_GCATP1 | . | n.-4380A>G | . | . |
| 17 | 17:16097870:A:C | A | 0.000 | 0.110 | C | 1.53E-06 | 0.00 | SNP | NCOR1 | missense_variant | XM_005256866.1 | p.Gly5Val | c.14G>T | . | 0.314675278 |
| 14 | 14:64066363:A:G | A | 0.189 | 0.035 | G | 1.53E-06 | 6.43 | SNP | GCATP1 | upstream_gene_variant | TRANSCRIPT_GCATP1 | . | n.-4319C>T | . | . |
| 14 | 14:64066373:A:G | A | 0.189 | 0.035 | G | 1.53E-06 | 6.43 | SNP | GCATP1 | upstream_gene_variant | TRANSCRIPT_GCATP1 | . | n.-4329C>T | . | . |
| 14 | 14:64066395:T:C | T | 0.189 | 0.035 | C | 1.53E-06 | 6.43 | SNP | WDR89 | missense_variant | NM_001008726.2 | p.Cys89Tyr | c.266G>A | . | . |
| 17 | 17:1761436:G:A | G | 0.303 | 0.100 | A | 1.60E-06 | 3.90 | SNP | RPA1 | intron_variant | NM_002945.3 | . | c.361+4953A>G | . | . |
| 23 | 23:7175842:D:2 | A | 0.106 | 0.000 | ATG | 1.88E-06 | NaN | InDel | STS | intron_variant | XM_005274511.1 | . | c.418+214_418+215delTG | . | . |
| 1 | 1:76253623:C:A | C | 0.098 | 0.000 | A | 1.89E-06 | NaN | SNP | SNORD45A | non_coding_transcript_exon_variant | NR_002749.1 | . | n.50A>C | . | . |
| 1 | 1:76253632:G:T | G | 0.098 | 0.000 | T | 1.89E-06 | NaN | SNP | SNORD45A | non_coding_transcript_exon_variant | NR_002749.1 | . | n.59T>G | . | . |
| 1 | 1:76253629:T:C | T | 0.098 | 0.000 | C | 1.89E-06 | NaN | SNP | SNORD45A | non_coding_transcript_exon_variant | NR_002749.1 | . | n.56C>T | . | . |
| 1 | 1:76253606:D:3 | T | 0.098 | 0.000 | TCTA | 1.89E-06 | NaN | InDel | SNORD45A | non_coding_transcript_exon_variant | NR_002749.1 | . | n.34_36delCTA | . | . |
| 1 | 1:76253622:C:A | C | 0.098 | 0.000 | A | 1.89E-06 | NaN | SNP | SNORD45A | non_coding_transcript_exon_variant | NR_002749.1 | . | n.49A>C | . | . |
| 17 | 17:16097912:C:T | C | 0.043 | 0.210 | T | 1.98E-06 | 0.17 | SNP | NCOR1 | 5_prime_UTR_variant | XM_005256866.1 | . | c.-29A>G | . | 0.246733282 |
| 17 | 17:1761457:T:C | T | 0.309 | 0.105 | C | 2.11E-06 | 3.81 | SNP | RPA1 | intron_variant | NM_002945.3 | . | c.361+4974C>T | . | . |
| 17 | 17:1761445:T:C | T | 0.309 | 0.105 | C | 2.11E-06 | 3.81 | SNP | RPA1 | intron_variant | NM_002945.3 | . | c.361+4962C>T | . | . |
| 17 | 17:1761447:A:G | A | 0.309 | 0.105 | G | 2.11E-06 | 3.81 | SNP | RPA1 | intron_variant | NM_002945.3 | . | c.361+4964G>A | . | . |
| 19 | 19:614028:I:34 | GGGGGCGTGGCCGGGGCGGGTGCCCTGGCGGGGGA | 0.142 | 0.015 | G | 2.23E-06 | 10.87 | InDel | HCN2 | intron_variant | NM_001194.3 | . | c.1990+12_1990+13insGGGGCGTGGCCGGGGCGGGTGCCCTGGCGGGGGA | . | 0.000415743 |
| 10 | 10:42949895:A:G | A | 0.207 | 0.045 | G | 2.23E-06 | 5.55 | SNP | CCNYL2 | intron_variant | NR_103829.1 | . | n.624-2764C>T | . | . |
| 10 | 10:42949893:T:C | T | 0.207 | 0.045 | C | 2.23E-06 | 5.55 | SNP | CCNYL2 | intron_variant | NR_103829.1 | . | n.624-2762G>A | . | . |
| 10 | 10:72468587:C:A | C | 0.000 | 0.115 | A | 2.26E-06 | 0.00 | SNP | ADAMTS14 | intron_variant | NM_139155.2 | . | c.870+53A>C | . | . |
| 14 | 14:100594940:D:3 | C | 0.098 | 0.000 | CAGT | 2.27E-06 | NaN | InDel | EVL | disruptive_inframe_deletion | XM_005267749.1 | p.Val196del | c.585_587delAGT | . | 0.003392706 |
| 4 | 4:71009033:A:G | A | 0.269 | 0.078 | G | 2.31E-06 | 4.34 | SNP | CSN1S2BP | intron_variant | NR_033311.1 | . | n.347+110A>G | 0.764 | . |
| 6 | 6:144814625:D:2 | G | 0.195 | 0.040 | GTC | 2.56E-06 | 5.82 | InDel | UTRN | intron_variant | XM_005267127.1 | . | c.4608+34_4608+35delTC | . | . |
| 14 | 14:64066328:T:C | T | 0.165 | 0.025 | C | 2.76E-06 | 7.69 | SNP | GCATP1 | upstream_gene_variant | TRANSCRIPT_GCATP1 | . | n.-4284G>A | . | 0 |
| 14 | 14:64066326:T:C | T | 0.165 | 0.025 | C | 2.76E-06 | 7.69 | SNP | WDR89 | missense_variant | NM_001008726.2 | p.Gly112Asp | c.335G>A | . | 0 |
| 14 | 14:64066340:G:A | G | 0.165 | 0.025 | A | 2.76E-06 | 7.69 | SNP | GCATP1 | upstream_gene_variant | TRANSCRIPT_GCATP1 | . | n.-4296T>C | . | . |
| 14 | 14:64066472:G:T | G | 0.128 | 0.010 | T | 2.89E-06 | 14.54 | SNP | GCATP1 | upstream_gene_variant | TRANSCRIPT_GCATP1 | . | n.-4428A>C | . | . |
| 14 | 14:64066367:T:A | T | 0.183 | 0.035 | A | 3.03E-06 | 6.17 | SNP | WDR89 | missense_variant | NM_001008726.2 | p.Asp98Glu | c.294T>A | . | . |
| 1 | 1:28817969:I:1 | CA | 0.293 | 0.100 | C | 3.19E-06 | 3.72 | InDel | PHACTR4 | intron_variant | XM_005245970.1 | . | c.1933-193_1933-192insA | . | . |
| 16 | 16:66506:A:G | A | 0.179 | 0.031 | G | 3.28E-06 | 6.76 | SNP | DDX11L10 | downstream_gene_variant | NR_045117.1 | . | n.*2416G>A | . | . |
| 6 | 6:29857098:D:2 | C | 0.161 | 0.021 | CCG | 3.32E-06 | 8.79 | InDel | HLA-H | splice_region_variant | NR_001434.2 | . | n.772-6_772-5delCG | . | 0.005129959 |
| 9 | 9:139407369:D:3 | C | 0.100 | 0.000 | CTGG | 3.39E-06 | NaN | InDel | NOTCH1 | intron_variant | NM_017617.3 | . | c.2467+101_2467+103delCCA | . | . |
| 14 | 14:51374819:I:1 | GT | 0.148 | 0.016 | G | 3.52E-06 | 10.38 | InDel | PYGL | intron_variant | NM_002863.4 | . | c.2379+169dupA | . | . |
| 10 | 10:46952565:G:A | G | 0.216 | 0.051 | A | 3.64E-06 | 5.18 | SNP | LOC101927699 | intron_variant | XR_246211.1 | . | n.312+782A>G | . | . |
| 7 | 7:142468228:G:A | G | 0.067 | 0.245 | A | 3.67E-06 | 0.22 | SNP | TRB | intragenic_variant | TRB | . | n.142468228A>G | . | . |
| 18 | 18:52551370:I:1 | TA | 0.303 | 0.105 | T | 3.68E-06 | 3.70 | InDel | RAB27B | intron_variant | NM_004163.4 | . | c.240-194_240-193insA | . | . |
| 10 | 10:42949897:C:T | C | 0.201 | 0.045 | T | 4.21E-06 | 5.35 | SNP | CCNYL2 | intron_variant | NR_103829.1 | . | n.624-2766A>G | . | . |
| 19 | 19:18331859:I:20 | TCCGCCAGGCCCCGCCCCACC | 0.247 | 0.068 | T | 4.28E-06 | 4.46 | InDel | PDE4C | intron_variant | NM_000923.4 | . | c.545+55_545+56insGGTGGGGCGGGGCCTGGCGG | 0.190 | . |
| 5 | 5:40832821:G:A | G | 0.091 | 0.000 | A | 4.43E-06 | NaN | SNP | SNORD72 | non_coding_transcript_exon_variant | NR_002583.1 | . | n.17T>C | . | . |
| 5 | 5:40832820:G:C | G | 0.091 | 0.000 | C | 4.43E-06 | NaN | SNP | SNORD72 | non_coding_transcript_exon_variant | NR_002583.1 | . | n.18G>C | . | . |
| 5 | 5:40832814:C:T | C | 0.091 | 0.000 | T | 4.43E-06 | NaN | SNP | SNORD72 | non_coding_transcript_exon_variant | NR_002583.1 | . | n.24A>G | . | . |
| 3 | 3:197362829:C:A | C | 0.185 | 0.033 | A | 4.52E-06 | 6.59 | SNP | LOC101926986 | upstream_gene_variant | TRANSCRIPT_LOC101926986 | . | n.-887A>C | . | . |
| 11 | 11:1030387:D:2 | C | 0.140 | 0.015 | CTG | 4.59E-06 | 10.60 | InDel | MUC6 | intron_variant | NM_005961.2 | . | c.893-54_893-53delCA | . | . |
| 1 | 1:148741630:G:C | G | 0.195 | 0.039 | C | 4.99E-06 | 5.92 | SNP | LOC101927312 | intron_variant | XR_241110.1 | . | n.499+492G>C | . | . |
| 10 | 10:101295274:D:3 | G | 0.216 | 0.055 | GGGC | 5.34E-06 | 4.74 | InDel | NKX2-3 | conservative_inframe_deletion | NM_145285.2 | p.Gly298del | c.892_894delGGC | . | . |
| 21 | 21:46911299:I:1 | GC | 0.543 | 0.304 | G | 5.53E-06 | 2.72 | InDel | COL18A1 | intron_variant | XM_005261178.1 | . | c.2697+71_2697+72insC | . | . |
| 14 | 14:64066352:A:T | A | 0.171 | 0.030 | T | 5.59E-06 | 6.66 | SNP | WDR89 | missense_variant | NM_001008726.2 | p.Arg103Ser | c.309A>T | . | . |
| 4 | 4:9982191:C:T | C | 0.360 | 0.600 | T | 5.63E-06 | 0.37 | SNP | SLC2A9 | intron_variant | NM_020041.2 | . | c.681+25G>A | 0.418 | 0.425766729 |
| 20 | 20:25755812:G:A | G | 0.124 | 0.010 | A | 5.64E-06 | 13.94 | SNP | FAM182B | non_coding_transcript_exon_variant | XR_244224.1 | . | n.445T>C | . | 0.006289308 |
| 9 | 9:94985153:I:1 | GA | 0.181 | 0.029 | G | 5.66E-06 | 7.31 | InDel | IARS | intron_variant | NM_002161.5 | . | c.3554-200dupT | . | . |
| 11 | 11:46473389:D:2 | T | 0.000 | 0.100 | TGA | 5.73E-06 | 0.00 | InDel | MIR3160-1 | non_coding_transcript_exon_variant | NR_036117.1 | . | n.49_50delTC | . | . |
| 9 | 9:33799267:T:C | T | 0.024 | 0.165 | C | 5.81E-06 | 0.13 | SNP | LOC101929665 | intron_variant | XR_242542.1 | . | n.1145+518G>A | . | . |
| 3 | 3:122640541:C:T | C | 0.409 | 0.186 | T | 6.38E-06 | 3.02 | SNP | SEMA5B | intron_variant | NM_001256347.1 | . | c.1850+221A>G | 0.295 | . |
| 16 | 16:89775433:I:4 | GCCCA | 0.210 | 0.049 | G | 6.44E-06 | 5.11 | InDel | VPS9D1 | intron_variant | XM_005256329.1 | . | c.1601-73_1601-70dupTGGG | . | . |
| 8 | 8:53536112:I:1 | TA | 0.232 | 0.065 | T | 6.62E-06 | 4.34 | InDel | RB1CC1 | 3_prime_UTR_variant | NM_014781.4 | . | c.*229dupT | . | . |
| 4 | 4:71008940:T:C | T | 0.232 | 0.066 | C | 7.07E-06 | 4.29 | SNP | CSN1S2BP | intron_variant | NR_033311.1 | . | n.347+17C>T | 0.206 | 0.234848485 |
| 1 | 1:12888310:G:A | G | 0.122 | 0.010 | A | 7.72E-06 | 13.33 | SNP | PRAMEF11 | intron_variant | NM_001146344.1 | . | c.167+47T>C | . | 0.097087379 |
| 1 | 1:33476396:A:G | A | 0.055 | 0.215 | G | 8.84E-06 | 0.21 | SNP | AK2 | non_coding_transcript_exon_variant | NR_037591.1 | . | n.934C>T | . | 0.187319127 |
| 10 | 10:103892605:C:T | C | 0.104 | 0.000 | T | 9.86E-06 | NaN | SNP | PPRC1 | upstream_gene_variant | NM_015062.3 | . | c.-221T>C | 0.053 | . |
| 16 | 16:14700254:G:A | G | 0.210 | 0.055 | A | 9.94E-06 | 4.56 | SNP | PARN | intron_variant | NM_002582.3 | . | c.702+87C>T | 0.724 | . |

*Note: CHR, Chromosome code; BP, Base-pair coordinate; A1, Allele 1 the minor; FA, Allele 1 frequency among cases; FU, Allele 1 frequency among controls; KEGG, Kyoto Encyclopedia of Genes and Genomes；ExAC, Exome Aggregation Consortium; EAS, East Asian; MAF, Minor allele frequency.*
