# Supplementary material for: Identification of potential candidate genes and pathways in atrioventricular nodal reentry tachycardia by whole‐exome sequencing
Source: Clin Transl Med. 2020 Apr 30;10(1):238–57. doi: 10.1002/ctm2.25 (PMC7240861; doi:10.1002/ctm2.25)
Supplement: Supplementary file 5 — Supporting Information S4 [file CTM2-10-238-s009.doc]

**S7: Reactome-kobas pathway enrichment (MAF <0.01)**

| **Term** | **Data Base** | **ID** | **Input Number** | **Background Number** | **P Value** | **Corrected P Value** | **Gene Name** |
| --- | --- | --- | --- | --- | --- | --- | --- |
| Signal Transduction | Reactome | R-HSA-162582 | 43 | 2448 | 1.55E-07 | 1.19E-05 | OR6C76|RDH16|CTBP2|CLPS|SMARCA4|SAV1|PIK3CB|RSPO4|E2F3|KDM4C|HDAC4|OR2T35|OR11H2|CIT|CFTR|MKL1|TSC1|IFT122|TAS2R46|SYDE1|NPY4R|KTN1|BCR|TAS2R31|NOXO1|TAS2R19|ARHGAP11A|OR2T2|EVL|OR51S1|CSF2RB|EVC|OR8B3|TCF7L1|SSTR1|AMOTL2|ESRP1|PRKAG2|OR8U1|DHRS4|GIPR|SPP1|MKS1 |
| Extracellular matrix organization | Reactome | R-HSA-1474244 | 9 | 290 | 3.91E-04 | 9.22E-03 | LAMC3|LAMC1|MMP2|ITGAL|MMP11|TNC|PRSS1|CTSS|SPP1 |
| Degradation of the extracellular matrix | Reactome | R-HSA-1474228 | 6 | 135 | 6.25E-04 | 1.33E-02 | LAMC1|MMP2|MMP11|PRSS1|CTSS|SPP1 |
| Deadenylation of mRNA | Reactome | R-HSA-429947 | 3 | 23 | 8.89E-04 | 1.73E-02 | CNOT1|CNOT2|PAN2 |
| Signaling by Rho GTPases | Reactome | R-HSA-194315 | 10 | 416 | 1.28E-03 | 2.13E-02 | SYDE1|CFTR|BCR|CIT|MKL1|ARHGAP11A|NOXO1|EVL|KDM4C|KTN1 |
| Activation of Matrix Metalloproteinases | Reactome | R-HSA-1592389 | 3 | 32 | 2.13E-03 | 3.05E-02 | MMP11|MMP2|PRSS1 |
| Disease | Reactome | R-HSA-1643685 | 13 | 902 | 1.81E-02 | 1.17E-01 | ABCC8|MUT|CFTR|BCR|GTF2H4|GALNS|PIK3CB|MUC2|ABCC6|CTBP2|SFTPA2|HDAC4|CSF2RB |
| Inositol phosphate metabolism | Reactome | R-HSA-1483249 | 3 | 47 | 5.89E-03 | 5.85E-02 | PLCD1|INPP5A|IPPK |
| Defective CSF2RA causes pulmonary surfactant metabolism dysfunction 4 (SMDP4) | Reactome | R-HSA-5688890 | 2 | 8 | 2.31E-03 | 3.23E-02 | SFTPA2|CSF2RB |
| Defective CSF2RB causes pulmonary surfactant metabolism dysfunction 5 (SMDP5) | Reactome | R-HSA-5688849 | 2 | 8 | 2.31E-03 | 3.23E-02 | SFTPA2|CSF2RB |
| Class C/3 (Metabotropic glutamate/pheromone receptors) | Reactome | R-HSA-420499 | 3 | 38 | 3.36E-03 | 4.19E-02 | TAS2R46|TAS2R31|TAS2R19 |
| ABC transporter disorders | Reactome | R-HSA-5619084 | 4 | 81 | 3.57E-03 | 4.35E-02 | ABCC8|SFTPA2|ABCC6|CFTR |
| Neuronal System | Reactome | R-HSA-112316 | 8 | 339 | 4.27E-03 | 4.82E-02 | ABCC8|PPFIA1|LRFN4|TSPOAP1|BEGAIN|GAD2|SYT10|KCNV2 |
| Diseases associated with surfactant metabolism | Reactome | R-HSA-5687613 | 2 | 12 | 4.58E-03 | 4.95E-02 | SFTPA2|CSF2RB |
| GPCR downstream signaling | Reactome | R-HSA-388396 | 15 | 940 | 4.88E-03 | 5.13E-02 | OR6C76|OR11H2|OR8B3|TAS2R31|OR2T35|TAS2R46|NPY4R|SSTR1|TAS2R19|OR8U1|PIK3CB|OR2T2|GIPR|CSF2RB|OR51S1 |
| Repression of WNT target genes | Reactome | R-HSA-4641265 | 2 | 13 | 5.26E-03 | 5.34E-02 | TCF7L1|CTBP2 |
| Neurotransmitter Release Cycle | Reactome | R-HSA-112310 | 3 | 50 | 6.93E-03 | 6.44E-02 | TSPOAP1|PPFIA1|GAD2 |
| Deadenylation-dependent mRNA decay | Reactome | R-HSA-429914 | 3 | 50 | 6.93E-03 | 6.44E-02 | CNOT1|CNOT2|PAN2 |
| Acetylcholine Neurotransmitter Release Cycle | Reactome | R-HSA-264642 | 2 | 16 | 7.55E-03 | 6.84E-02 | TSPOAP1|PPFIA1 |
| RHO GTPase Effectors | Reactome | R-HSA-195258 | 7 | 304 | 8.31E-03 | 7.23E-02 | CFTR|CIT|NOXO1|MKL1|EVL|KDM4C|KTN1 |
| Serotonin Neurotransmitter Release Cycle | Reactome | R-HSA-181429 | 2 | 17 | 8.40E-03 | 7.24E-02 | TSPOAP1|PPFIA1 |
| Norepinephrine Neurotransmitter Release Cycle | Reactome | R-HSA-181430 | 2 | 17 | 8.40E-03 | 7.24E-02 | TSPOAP1|PPFIA1 |
| Diseases of metabolism | Reactome | R-HSA-5668914 | 4 | 106 | 8.84E-03 | 7.44E-02 | CSF2RB|SFTPA2|MUT|GALNS |
| ABC-family proteins mediated transport | Reactome | R-HSA-382556 | 4 | 106 | 8.84E-03 | 7.44E-02 | ABCC8|ABCA7|ABCC6|CFTR |
| DNA Replication | Reactome | R-HSA-69306 | 2 | 108 | 1.93E-01 | 3.20E-01 | POLD1|E2F3 |
| Signaling by Hippo | Reactome | R-HSA-2028269 | 2 | 19 | 1.02E-02 | 8.14E-02 | SAV1|AMOTL2 |
| Non-integrin membrane-ECM interactions | Reactome | R-HSA-3000171 | 3 | 59 | 1.07E-02 | 8.37E-02 | LAMC3|LAMC1|TNC |
| Cobalamin (Cbl, vitamin B12) transport and metabolism | Reactome | R-HSA-196741 | 2 | 20 | 1.12E-02 | 8.68E-02 | PRSS1|MUT |
| TP53 regulates transcription of additional cell cycle genes whose exact role in the p53 pathway remain uncertain | Reactome | R-HSA-6804115 | 2 | 21 | 1.22E-02 | 9.14E-02 | CNOT1|CNOT2 |
| Dopamine Neurotransmitter Release Cycle | Reactome | R-HSA-212676 | 2 | 22 | 1.32E-02 | 9.62E-02 | TSPOAP1|PPFIA1 |
| RA biosynthesis pathway | Reactome | R-HSA-5365859 | 2 | 22 | 1.32E-02 | 9.62E-02 | RDH16|DHRS4 |
| Gap-filling DNA repair synthesis and ligation in TC-NER | Reactome | R-HSA-6782210 | 3 | 65 | 1.37E-02 | 9.76E-02 | POLD1|AQR|GTF2H4 |
| Dual incision in TC-NER | Reactome | R-HSA-6782135 | 3 | 66 | 1.42E-02 | 9.99E-02 | POLD1|AQR|GTF2H4 |
| Glutamate Neurotransmitter Release Cycle | Reactome | R-HSA-210500 | 2 | 23 | 1.43E-02 | 9.99E-02 | TSPOAP1|PPFIA1 |
| PI3K Cascade | Reactome | R-HSA-109704 | 3 | 72 | 1.78E-02 | 1.15E-01 | TSC1|PIK3CB|PRKAG2 |
| Energy dependent regulation of mTOR by LKB1-AMPK | Reactome | R-HSA-380972 | 2 | 28 | 2.03E-02 | 1.25E-01 | TSC1|PRKAG2 |
| Transcription-Coupled Nucleotide Excision Repair (TC-NER) | Reactome | R-HSA-6781827 | 3 | 79 | 2.25E-02 | 1.33E-01 | POLD1|AQR|GTF2H4 |
| Axon guidance | Reactome | R-HSA-422475 | 9 | 549 | 2.26E-02 | 1.33E-01 | EPHB4|LAMC1|MMP2|DOK4|CSF2RB|SCN1A|EVL|ROBO1|PIK3CB |
| Olfactory Signaling Pathway | Reactome | R-HSA-381753 | 7 | 374 | 2.28E-02 | 1.33E-01 | OR6C76|OR11H2|OR8B3|OR2T35|OR8U1|OR2T2|OR51S1 |
| RNA Polymerase I Promoter Escape | Reactome | R-HSA-73772 | 2 | 30 | 2.29E-02 | 1.33E-01 | GTF2H4|TAF1C |
| Surfactant metabolism | Reactome | R-HSA-5683826 | 2 | 30 | 2.29E-02 | 1.33E-01 | SFTPA2|CSF2RB |
| Laminin interactions | Reactome | R-HSA-3000157 | 2 | 30 | 2.29E-02 | 1.33E-01 | LAMC3|LAMC1 |
| RNA Polymerase I Transcription Termination | Reactome | R-HSA-73863 | 2 | 31 | 2.43E-02 | 1.37E-01 | GTF2H4|TAF1C |
| Signaling by Robo receptor | Reactome | R-HSA-376176 | 2 | 32 | 2.57E-02 | 1.42E-01 | ROBO1|EVL |
| Disorders of transmembrane transporters | Reactome | R-HSA-5619115 | 4 | 149 | 2.64E-02 | 1.45E-01 | ABCC8|SFTPA2|ABCC6|CFTR |
| Integrin cell surface interactions | Reactome | R-HSA-216083 | 3 | 85 | 2.70E-02 | 1.46E-01 | ITGAL|SPP1|TNC |
| Metabolism of proteins | Reactome | R-HSA-392499 | 17 | 1378 | 2.88E-02 | 1.52E-01 | USP17L11|ATXN3|CFTR|OTUD7A|GSN|GCNT3|ACE2|CSF2RB|MGAT4C|XRN2|MMP2|SP100|LDHD|COG4|SFTPA2|MUC2|TIMM23 |
| G alpha (i) signalling events | Reactome | R-HSA-418594 | 5 | 242 | 3.58E-02 | 1.76E-01 | TAS2R46|NPY4R|SSTR1|TAS2R31|TAS2R19 |
| Anchoring of the basal body to the plasma membrane | Reactome | R-HSA-5620912 | 3 | 96 | 3.64E-02 | 1.76E-01 | PCM1|CEP70|MKS1 |
| Metabolism | Reactome | R-HSA-1430728 | 22 | 1975 | 3.77E-02 | 1.80E-01 | ABCC8|ACOT8|CLPS|IPPK|GAMT|INPP5A|PIK3CB|TECRL|PLCD1|PRSS1|XDH|PSTK|MUT|PNPLA8|GALNS|TPTE2|GSTZ1|CYB5R2|POLD1|SCLY|PRKAG2|CYP2F1 |
| Fanconi Anemia Pathway | Reactome | R-HSA-6783310 | 2 | 40 | 3.79E-02 | 1.80E-01 | FAAP20|FAN1 |
| mTOR signalling | Reactome | R-HSA-165159 | 2 | 40 | 3.79E-02 | 1.80E-01 | TSC1|PRKAG2 |
| PKB-mediated events | Reactome | R-HSA-109703 | 2 | 41 | 3.96E-02 | 1.81E-01 | TSC1|PRKAG2 |
| Dual Incision in GG-NER | Reactome | R-HSA-5696400 | 2 | 41 | 3.96E-02 | 1.81E-01 | POLD1|GTF2H4 |
| Signaling by Retinoic Acid | Reactome | R-HSA-5362517 | 2 | 42 | 4.12E-02 | 1.81E-01 | RDH16|DHRS4 |
| Beta-oxidation of very long chain fatty acids | Reactome | R-HSA-390247 | 1 | 5 | 4.31E-02 | 1.81E-01 | ACOT8 |
| G2 Phase | Reactome | R-HSA-68911 | 1 | 5 | 4.31E-02 | 1.81E-01 | E2F3 |
| Alternative complement activation | Reactome | R-HSA-173736 | 1 | 5 | 4.31E-02 | 1.81E-01 | CFD |
| Propionyl-CoA catabolism | Reactome | R-HSA-71032 | 1 | 5 | 4.31E-02 | 1.81E-01 | MUT |
| Signaling by GPCR | Reactome | R-HSA-372790 | 15 | 1248 | 4.63E-02 | 1.87E-01 | OR6C76|OR11H2|OR8B3|TAS2R31|OR2T35|TAS2R46|NPY4R|SSTR1|TAS2R19|OR8U1|PIK3CB|OR2T2|GIPR|CSF2RB|OR51S1 |
| RNA Polymerase I Transcription Initiation | Reactome | R-HSA-73762 | 2 | 47 | 5.01E-02 | 1.87E-01 | GTF2H4|TAF1C |
| G beta:gamma signalling through PI3Kgamma | Reactome | R-HSA-392451 | 2 | 47 | 5.01E-02 | 1.87E-01 | PIK3CB|CSF2RB |
| Nucleotide Excision Repair | Reactome | R-HSA-5696398 | 3 | 111 | 5.15E-02 | 1.91E-01 | POLD1|AQR|GTF2H4 |
| Clearance of Nuclear Envelope Membranes from Chromatin | Reactome | R-HSA-2993913 | 1 | 6 | 5.01E-02 | 1.87E-01 | LEMD3 |
| Defective ABCA3 causes pulmonary surfactant metabolism dysfunction type 3 (SMDP3) | Reactome | R-HSA-5683678 | 1 | 6 | 5.01E-02 | 1.87E-01 | SFTPA2 |
| Developmental Biology | Reactome | R-HSA-1266738 | 11 | 841 | 5.04E-02 | 1.88E-01 | EPHB4|LAMC1|MMP2|ADAM22|DOK4|CSF2RB|TCF3|EVL|ROBO1|SCN1A|PIK3CB |
| TP53 Regulates Transcription of Cell Cycle Genes | Reactome | R-HSA-6791312 | 2 | 48 | 5.19E-02 | 1.92E-01 | CNOT1|CNOT2 |
| EPH-ephrin mediated repulsion of cells | Reactome | R-HSA-3928665 | 2 | 49 | 5.38E-02 | 1.97E-01 | EPHB4|MMP2 |
| Fatty Acyl-CoA Biosynthesis | Reactome | R-HSA-75105 | 2 | 49 | 5.38E-02 | 1.97E-01 | TECRL|ACOT8 |
| Apoptotic execution phase | Reactome | R-HSA-75153 | 2 | 50 | 5.57E-02 | 1.97E-01 | GSN|HIST1H1A |
| G-protein beta:gamma signalling | Reactome | R-HSA-397795 | 2 | 50 | 5.57E-02 | 1.97E-01 | PIK3CB|CSF2RB |
| Cation-coupled Chloride cotransporters | Reactome | R-HSA-426117 | 1 | 7 | 5.71E-02 | 1.97E-01 | SLC12A4 |
| Binding of TCF/LEF:CTNNB1 to target gene promoters | Reactome | R-HSA-4411364 | 1 | 7 | 5.71E-02 | 1.97E-01 | TCF7L1 |
| Assembly of the primary cilium | Reactome | R-HSA-5617833 | 4 | 199 | 6.26E-02 | 2.05E-01 | MKS1|PCM1|CEP70|IFT122 |
| Mitochondrial protein import | Reactome | R-HSA-1268020 | 2 | 54 | 6.35E-02 | 2.05E-01 | LDHD|TIMM23 |
| Formation of TC-NER Pre-Incision Complex | Reactome | R-HSA-6781823 | 2 | 54 | 6.35E-02 | 2.05E-01 | AQR|GTF2H4 |
| AMPK inhibits chREBP transcriptional activation activity | Reactome | R-HSA-163680 | 1 | 8 | 6.39E-02 | 2.05E-01 | PRKAG2 |
| Rho GTPase cycle | Reactome | R-HSA-194840 | 3 | 122 | 6.43E-02 | 2.06E-01 | ARHGAP11A|SYDE1|BCR |
| GPVI-mediated activation cascade | Reactome | R-HSA-114604 | 2 | 55 | 6.55E-02 | 2.08E-01 | PIK3CB|CSF2RB |
| Interactions of neurexins and neuroligins at synapses | Reactome | R-HSA-6794361 | 2 | 57 | 6.95E-02 | 2.12E-01 | BEGAIN|SYT10 |
| Protein-protein interactions at synapses | Reactome | R-HSA-6794362 | 2 | 57 | 6.95E-02 | 2.12E-01 | BEGAIN|SYT10 |
| Metabolism of ingested SeMet, Sec, MeSec into H2Se | Reactome | R-HSA-2408508 | 1 | 9 | 7.08E-02 | 2.12E-01 | SCLY |
| Beta-oxidation of pristanoyl-CoA | Reactome | R-HSA-389887 | 1 | 9 | 7.08E-02 | 2.12E-01 | ACOT8 |
| Role of Abl in Robo-Slit signaling | Reactome | R-HSA-428890 | 1 | 9 | 7.08E-02 | 2.12E-01 | ROBO1 |
| Inactivation of Cdc42 and Rac | Reactome | R-HSA-428543 | 1 | 9 | 7.08E-02 | 2.12E-01 | ROBO1 |
| GP1b-IX-V activation signalling | Reactome | R-HSA-430116 | 1 | 9 | 7.08E-02 | 2.12E-01 | GP1BA |
| PI Metabolism | Reactome | R-HSA-1483255 | 2 | 60 | 7.58E-02 | 2.22E-01 | TPTE2|PIK3CB |
| Activation of PPARGC1A (PGC-1alpha) by phosphorylation | Reactome | R-HSA-2151209 | 1 | 10 | 7.76E-02 | 2.22E-01 | PRKAG2 |
| Synthesis of pyrophosphates in the cytosol | Reactome | R-HSA-1855167 | 1 | 10 | 7.76E-02 | 2.22E-01 | IPPK |
| Removal of the Flap Intermediate from the C-strand | Reactome | R-HSA-174437 | 1 | 10 | 7.76E-02 | 2.22E-01 | POLD1 |
| Initiation of Nuclear Envelope Reformation | Reactome | R-HSA-2995383 | 1 | 10 | 7.76E-02 | 2.22E-01 | LEMD3 |
| Activation of SMO | Reactome | R-HSA-5635838 | 1 | 10 | 7.76E-02 | 2.22E-01 | EVC |
| Nuclear Envelope Reassembly | Reactome | R-HSA-2995410 | 1 | 10 | 7.76E-02 | 2.22E-01 | LEMD3 |
| DNA Repair | Reactome | R-HSA-73894 | 5 | 311 | 8.36E-02 | 2.26E-01 | FAAP20|FAN1|GTF2H4|AQR|POLD1 |
| Collagen degradation | Reactome | R-HSA-1442490 | 2 | 64 | 8.43E-02 | 2.26E-01 | MMP11|MMP2 |
| CYP2E1 reactions | Reactome | R-HSA-211999 | 1 | 11 | 8.43E-02 | 2.26E-01 | CYP2F1 |
| Purine catabolism | Reactome | R-HSA-74259 | 1 | 11 | 8.43E-02 | 2.26E-01 | XDH |
| Phenylalanine and tyrosine catabolism | Reactome | R-HSA-71182 | 1 | 11 | 8.43E-02 | 2.26E-01 | GSTZ1 |
| Import of palmitoyl-CoA into the mitochondrial matrix | Reactome | R-HSA-200425 | 1 | 11 | 8.43E-02 | 2.26E-01 | PRKAG2 |
| Mucopolysaccharidoses | Reactome | R-HSA-2206281 | 1 | 11 | 8.43E-02 | 2.26E-01 | GALNS |
| Endosomal/Vacuolar pathway | Reactome | R-HSA-1236977 | 1 | 11 | 8.43E-02 | 2.26E-01 | CTSS |
| CDC6 association with the ORC:origin complex | Reactome | R-HSA-68689 | 1 | 11 | 8.43E-02 | 2.26E-01 | E2F3 |
| Processive synthesis on the C-strand of the telomere | Reactome | R-HSA-174414 | 1 | 11 | 8.43E-02 | 2.26E-01 | POLD1 |
| RHO GTPases activate KTN1 | Reactome | R-HSA-5625970 | 1 | 11 | 8.43E-02 | 2.26E-01 | KTN1 |
| Synthesis of IP2, IP, and Ins in the cytosol | Reactome | R-HSA-1855183 | 1 | 11 | 8.43E-02 | 2.26E-01 | INPP5A |
| Creatine metabolism | Reactome | R-HSA-71288 | 1 | 11 | 8.43E-02 | 2.26E-01 | GAMT |
| Fatty acid, triacylglycerol, and ketone body metabolism | Reactome | R-HSA-535734 | 4 | 223 | 8.61E-02 | 2.29E-01 | TECRL|ACOT8|MUT|PRKAG2 |
| O-linked glycosylation of mucins | Reactome | R-HSA-913709 | 2 | 65 | 8.65E-02 | 2.29E-01 | GCNT3|MUC2 |
| Apoptosis | Reactome | R-HSA-109581 | 2 | 161 | 3.35E-01 | 4.34E-01 | GSN|HIST1H1A |
| Macroautophagy | Reactome | R-HSA-1632852 | 2 | 67 | 9.10E-02 | 2.31E-01 | TSC1|PRKAG2 |
| Josephin domain DUBs | Reactome | R-HSA-5689877 | 1 | 12 | 9.10E-02 | 2.31E-01 | ATXN3 |
| alpha-linolenic (omega3) and linoleic (omega6) acid metabolism | Reactome | R-HSA-2046104 | 1 | 12 | 9.10E-02 | 2.31E-01 | ACOT8 |
| Scavenging of heme from plasma | Reactome | R-HSA-2168880 | 1 | 12 | 9.10E-02 | 2.31E-01 | CD163 |
| Keratan sulfate degradation | Reactome | R-HSA-2022857 | 1 | 12 | 9.10E-02 | 2.31E-01 | GALNS |
| Caspase-mediated cleavage of cytoskeletal proteins | Reactome | R-HSA-264870 | 1 | 12 | 9.10E-02 | 2.31E-01 | GSN |
| alpha-linolenic acid (ALA) metabolism | Reactome | R-HSA-2046106 | 1 | 12 | 9.10E-02 | 2.31E-01 | ACOT8 |
| Loss of proteins required for interphase microtubule organization聽from the centrosome | Reactome | R-HSA-380284 | 2 | 68 | 9.32E-02 | 2.35E-01 | PCM1|CEP70 |
| Loss of Nlp from mitotic centrosomes | Reactome | R-HSA-380259 | 2 | 68 | 9.32E-02 | 2.35E-01 | PCM1|CEP70 |
| Metabolism of vitamins and cofactors | Reactome | R-HSA-196854 | 3 | 144 | 9.37E-02 | 2.36E-01 | PRSS1|MUT|CLPS |
| Erythrocytes take up carbon dioxide and release oxygen | Reactome | R-HSA-1237044 | 1 | 13 | 9.77E-02 | 2.36E-01 | CYB5R2 |
| O2/CO2 exchange in erythrocytes | Reactome | R-HSA-1480926 | 1 | 13 | 9.77E-02 | 2.36E-01 | CYB5R2 |
| Apoptosis induced DNA fragmentation | Reactome | R-HSA-140342 | 1 | 13 | 9.77E-02 | 2.36E-01 | HIST1H1A |
| Cytosolic iron-sulfur cluster assembly | Reactome | R-HSA-2564830 | 1 | 13 | 9.77E-02 | 2.36E-01 | POLD1 |
| Trafficking and processing of endosomal TLR | Reactome | R-HSA-1679131 | 1 | 13 | 9.77E-02 | 2.36E-01 | CTSS |
| RHO GTPases Activate NADPH Oxidases | Reactome | R-HSA-5668599 | 1 | 13 | 9.77E-02 | 2.36E-01 | NOXO1 |
| Activation of DNA fragmentation factor | Reactome | R-HSA-211227 | 1 | 13 | 9.77E-02 | 2.36E-01 | HIST1H1A |
| Depolymerisation of the Nuclear Lamina | Reactome | R-HSA-4419969 | 1 | 13 | 9.77E-02 | 2.36E-01 | LEMD3 |
| Passive transport by Aquaporins | Reactome | R-HSA-432047 | 1 | 13 | 9.77E-02 | 2.36E-01 | AQP7 |
| Clathrin derived vesicle budding | Reactome | R-HSA-421837 | 2 | 70 | 9.77E-02 | 2.36E-01 | HIP1R|AP1G2 |
| trans-Golgi Network Vesicle Budding | Reactome | R-HSA-199992 | 2 | 70 | 9.77E-02 | 2.36E-01 | HIP1R|AP1G2 |
| Signaling by Hedgehog | Reactome | R-HSA-5358351 | 3 | 148 | 9.95E-02 | 2.40E-01 | EVC|MKS1|IFT122 |
| AURKA Activation by TPX2 | Reactome | R-HSA-8854518 | 2 | 71 | 1.00E-01 | 2.41E-01 | PCM1|CEP70 |
| Signal regulatory protein (SIRP) family interactions | Reactome | R-HSA-391160 | 1 | 14 | 1.04E-01 | 2.43E-01 | SFTPA2 |
| Removal of the Flap Intermediate | Reactome | R-HSA-69166 | 1 | 14 | 1.04E-01 | 2.43E-01 | POLD1 |
| Defects in cobalamin (B12) metabolism | Reactome | R-HSA-3296469 | 1 | 14 | 1.04E-01 | 2.43E-01 | MUT |
| Mismatch repair (MMR) directed by MSH2:MSH6 (MutSalpha) | Reactome | R-HSA-5358565 | 1 | 14 | 1.04E-01 | 2.43E-01 | POLD1 |
| Polymerase switching on the C-strand of the telomere | Reactome | R-HSA-174411 | 1 | 14 | 1.04E-01 | 2.43E-01 | POLD1 |
| Leading Strand Synthesis | Reactome | R-HSA-69109 | 1 | 14 | 1.04E-01 | 2.43E-01 | POLD1 |
| LGI-ADAM interactions | Reactome | R-HSA-5682910 | 1 | 14 | 1.04E-01 | 2.43E-01 | ADAM22 |
| Mismatch repair (MMR) directed by MSH2:MSH3 (MutSbeta) | Reactome | R-HSA-5358606 | 1 | 14 | 1.04E-01 | 2.43E-01 | POLD1 |
| Polymerase switching | Reactome | R-HSA-69091 | 1 | 14 | 1.04E-01 | 2.43E-01 | POLD1 |
| Triglyceride Biosynthesis | Reactome | R-HSA-75109 | 2 | 74 | 1.07E-01 | 2.46E-01 | TECRL|ACOT8 |
| ECM proteoglycans | Reactome | R-HSA-3000178 | 2 | 74 | 1.07E-01 | 2.46E-01 | LAMC1|TNC |
| Organelle biogenesis and maintenance | Reactome | R-HSA-1852241 | 5 | 339 | 1.09E-01 | 2.47E-01 | MKS1|PCM1|CEP70|IFT122|PRKAG2 |
| Fatty acids | Reactome | R-HSA-211935 | 1 | 15 | 1.11E-01 | 2.47E-01 | CYP2F1 |
| Mismatch Repair | Reactome | R-HSA-5358508 | 1 | 15 | 1.11E-01 | 2.47E-01 | POLD1 |
| Association of licensing factors with the pre-replicative complex | Reactome | R-HSA-69298 | 1 | 15 | 1.11E-01 | 2.47E-01 | E2F3 |
| Signaling by cytosolic FGFR1 fusion mutants | Reactome | R-HSA-1839117 | 1 | 15 | 1.11E-01 | 2.47E-01 | BCR |
| Activation of Rac | Reactome | R-HSA-428540 | 1 | 15 | 1.11E-01 | 2.47E-01 | ROBO1 |
| N-Glycan antennae elongation | Reactome | R-HSA-975577 | 1 | 15 | 1.11E-01 | 2.47E-01 | MGAT4C |
| Platelet Adhesion to exposed collagen | Reactome | R-HSA-75892 | 1 | 15 | 1.11E-01 | 2.47E-01 | GP1BA |
| Processive synthesis on the lagging strand | Reactome | R-HSA-69183 | 1 | 15 | 1.11E-01 | 2.47E-01 | POLD1 |
| Transmembrane transport of small molecules | Reactome | R-HSA-382551 | 8 | 657 | 1.17E-01 | 2.55E-01 | ABCC8|SLC10A6|CFTR|SLC12A4|HEPH|ABCC6|AQP7|ABCA7 |
| Digestion of dietary lipid | Reactome | R-HSA-192456 | 1 | 16 | 1.17E-01 | 2.55E-01 | CLPS |
| RHO GTPases activate CIT | Reactome | R-HSA-5625900 | 1 | 16 | 1.17E-01 | 2.55E-01 | CIT |
| Formation of Senescence-Associated Heterochromatin Foci (SAHF) | Reactome | R-HSA-2559584 | 1 | 16 | 1.17E-01 | 2.55E-01 | HIST1H1A |
| Processing of Intronless Pre-mRNAs | Reactome | R-HSA-77595 | 1 | 16 | 1.17E-01 | 2.55E-01 | CPSF7 |
| GPCR ligand binding | Reactome | R-HSA-500792 | 6 | 451 | 1.20E-01 | 2.60E-01 | TAS2R31|TAS2R46|NPY4R|SSTR1|TAS2R19|GIPR |
| Mitochondrial Fatty Acid Beta-Oxidation | Reactome | R-HSA-77289 | 1 | 17 | 1.24E-01 | 2.63E-01 | MUT |
| Tie2 Signaling | Reactome | R-HSA-210993 | 1 | 17 | 1.24E-01 | 2.63E-01 | PIK3CB |
| Metabolism of Angiotensinogen to Angiotensins | Reactome | R-HSA-2022377 | 1 | 17 | 1.24E-01 | 2.63E-01 | ACE2 |
| Transcriptional Regulation by TP53 | Reactome | R-HSA-3700989 | 5 | 355 | 1.26E-01 | 2.66E-01 | CNOT1|TSC1|CNOT2|GTF2H4|PRKAG2 |
| TP53 Regulates Metabolic Genes | Reactome | R-HSA-5628897 | 2 | 82 | 1.26E-01 | 2.66E-01 | TSC1|PRKAG2 |
| Degradation of beta-catenin by the destruction complex | Reactome | R-HSA-195253 | 2 | 82 | 1.26E-01 | 2.66E-01 | TCF7L1|CTBP2 |
| Defective C1GALT1C1 causes Tn polyagglutination syndrome (TNPS) | Reactome | R-HSA-5083632 | 1 | 18 | 1.30E-01 | 2.69E-01 | MUC2 |
| GABA synthesis, release, reuptake and degradation | Reactome | R-HSA-888590 | 1 | 18 | 1.30E-01 | 2.69E-01 | GAD2 |
| Defective GALNT12 causes colorectal cancer 1 (CRCS1) | Reactome | R-HSA-5083636 | 1 | 18 | 1.30E-01 | 2.69E-01 | MUC2 |
| Synthesis of PIPs at the Golgi membrane | Reactome | R-HSA-1660514 | 1 | 18 | 1.30E-01 | 2.69E-01 | TPTE2 |
| Ephrin signaling | Reactome | R-HSA-3928664 | 1 | 18 | 1.30E-01 | 2.69E-01 | EPHB4 |
| ABC transporters in lipid homeostasis | Reactome | R-HSA-1369062 | 1 | 18 | 1.30E-01 | 2.69E-01 | ABCA7 |
| Defective GALNT3 causes familial hyperphosphatemic tumoral calcinosis (HFTC) | Reactome | R-HSA-5083625 | 1 | 18 | 1.30E-01 | 2.69E-01 | MUC2 |
| Phospholipid metabolism | Reactome | R-HSA-1483257 | 3 | 168 | 1.31E-01 | 2.69E-01 | TPTE2|PNPLA8|PIK3CB |
| Global Genome Nucleotide Excision Repair (GG-NER) | Reactome | R-HSA-5696399 | 2 | 84 | 1.31E-01 | 2.69E-01 | POLD1|GTF2H4 |
| Regulation of PLK1 Activity at G2/M Transition | Reactome | R-HSA-2565942 | 2 | 85 | 1.33E-01 | 2.73E-01 | PCM1|CEP70 |
| Regulation of TLR by endogenous ligand | Reactome | R-HSA-5686938 | 1 | 19 | 1.37E-01 | 2.74E-01 | SFTPA2 |
| Lagging Strand Synthesis | Reactome | R-HSA-69186 | 1 | 20 | 1.43E-01 | 2.80E-01 | POLD1 |
| Purine metabolism | Reactome | R-HSA-73847 | 1 | 35 | 2.32E-01 | 3.53E-01 | XDH |
| mRNA Splicing - Major Pathway | Reactome | R-HSA-72163 | 3 | 177 | 1.46E-01 | 2.83E-01 | CPSF7|CWC27|AQR |
| Formation of the beta-catenin:TCF transactivating complex | Reactome | R-HSA-201722 | 2 | 90 | 1.46E-01 | 2.83E-01 | TCF7L1|SMARCA4 |
| RNA Polymerase I Chain Elongation | Reactome | R-HSA-73777 | 2 | 90 | 1.46E-01 | 2.83E-01 | GTF2H4|TAF1C |
| Centrosome maturation | Reactome | R-HSA-380287 | 2 | 91 | 1.48E-01 | 2.84E-01 | PCM1|CEP70 |
| Recruitment of mitotic centrosome proteins and complexes | Reactome | R-HSA-380270 | 2 | 91 | 1.48E-01 | 2.84E-01 | PCM1|CEP70 |
| Regulation of FZD by ubiquitination | Reactome | R-HSA-4641263 | 1 | 21 | 1.49E-01 | 2.84E-01 | RSPO4 |
| Xenobiotics | Reactome | R-HSA-211981 | 1 | 21 | 1.49E-01 | 2.84E-01 | CYP2F1 |
| Regulation of signaling by CBL | Reactome | R-HSA-912631 | 1 | 21 | 1.49E-01 | 2.84E-01 | PIK3CB |
| Regulation of Insulin-like Growth Factor (IGF) transport and uptake by Insulin-like Growth Factor Binding Proteins (IGFBPs) | Reactome | R-HSA-381426 | 1 | 21 | 1.49E-01 | 2.84E-01 | MMP2 |
| PCNA-Dependent Long Patch Base Excision Repair | Reactome | R-HSA-5651801 | 1 | 21 | 1.49E-01 | 2.84E-01 | POLD1 |
| Nephrin interactions | Reactome | R-HSA-373753 | 1 | 21 | 1.49E-01 | 2.84E-01 | PIK3CB |
| SALM protein interactions at the synapses | Reactome | R-HSA-8849932 | 1 | 21 | 1.49E-01 | 2.84E-01 | LRFN4 |
| IRS-mediated signalling | Reactome | R-HSA-112399 | 4 | 276 | 1.50E-01 | 2.86E-01 | CSF2RB|TSC1|PIK3CB|PRKAG2 |
| Platelet activation, signaling and aggregation | Reactome | R-HSA-76002 | 4 | 277 | 1.52E-01 | 2.88E-01 | CFD|PIK3CB|GP1BA|CSF2RB |
| EPH-Ephrin signaling | Reactome | R-HSA-2682334 | 2 | 93 | 1.54E-01 | 2.89E-01 | EPHB4|MMP2 |
| Insulin receptor signalling cascade | Reactome | R-HSA-74751 | 4 | 279 | 1.55E-01 | 2.89E-01 | CSF2RB|TSC1|PIK3CB|PRKAG2 |
| Initial triggering of complement | Reactome | R-HSA-166663 | 1 | 22 | 1.55E-01 | 2.89E-01 | CFD |
| Intrinsic Pathway of Fibrin Clot Formation | Reactome | R-HSA-140837 | 1 | 22 | 1.55E-01 | 2.89E-01 | GP1BA |
| IGF1R signaling cascade | Reactome | R-HSA-2428924 | 4 | 280 | 1.56E-01 | 2.89E-01 | CSF2RB|TSC1|PIK3CB|PRKAG2 |
| Signaling by Type 1 Insulin-like Growth Factor 1 Receptor (IGF1R) | Reactome | R-HSA-2404192 | 4 | 280 | 1.56E-01 | 2.89E-01 | CSF2RB|TSC1|PIK3CB|PRKAG2 |
| IRS-related events triggered by IGF1R | Reactome | R-HSA-2428928 | 4 | 280 | 1.56E-01 | 2.89E-01 | CSF2RB|TSC1|PIK3CB|PRKAG2 |
| Metabolism of lipids and lipoproteins | Reactome | R-HSA-556833 | 8 | 707 | 1.56E-01 | 2.89E-01 | ACOT8|CLPS|TECRL|PRKAG2|TPTE2|MUT|PNPLA8|PIK3CB |
| Metabolism of water-soluble vitamins and cofactors | Reactome | R-HSA-196849 | 2 | 94 | 1.56E-01 | 2.89E-01 | PRSS1|MUT |
| Diseases of signal transduction | Reactome | R-HSA-5663202 | 4 | 282 | 1.59E-01 | 2.93E-01 | BCR|CTBP2|HDAC4|PIK3CB |
| mRNA Splicing | Reactome | R-HSA-72172 | 3 | 185 | 1.60E-01 | 2.93E-01 | CPSF7|CWC27|AQR |
| Cellular responses to stress | Reactome | R-HSA-2262752 | 5 | 387 | 1.61E-01 | 2.93E-01 | E2F3|TSC1|CCS|HIST1H1A|PRKAG2 |
| Synthesis of bile acids and bile salts via 7alpha-hydroxycholesterol | Reactome | R-HSA-193368 | 1 | 23 | 1.62E-01 | 2.93E-01 | ACOT8 |
| Defects in vitamin and cofactor metabolism | Reactome | R-HSA-3296482 | 1 | 23 | 1.62E-01 | 2.93E-01 | MUT |
| The canonical retinoid cycle in rods (twilight vision) | Reactome | R-HSA-2453902 | 1 | 23 | 1.62E-01 | 2.93E-01 | RDH16 |
| Nitric oxide stimulates guanylate cyclase | Reactome | R-HSA-392154 | 1 | 23 | 1.62E-01 | 2.93E-01 | NOS1 |
| Mitotic G2-G2/M phases | Reactome | R-HSA-453274 | 3 | 187 | 1.63E-01 | 2.96E-01 | PCM1|CEP70|E2F3 |
| Hemostasis | Reactome | R-HSA-109582 | 7 | 605 | 1.64E-01 | 2.97E-01 | GP1BA|ITGAL|PIK3CB|DOCK5|CFD|CSF2RB|NOS1 |
| Visual phototransduction | Reactome | R-HSA-2187338 | 2 | 98 | 1.66E-01 | 2.99E-01 | RDH16|CLPS |
| Resolution of AP sites via the multiple-nucleotide patch replacement pathway | Reactome | R-HSA-110373 | 1 | 24 | 1.68E-01 | 2.99E-01 | POLD1 |
| Telomere C-strand (Lagging Strand) Synthesis | Reactome | R-HSA-174417 | 1 | 24 | 1.68E-01 | 2.99E-01 | POLD1 |
| Role of phospholipids in phagocytosis | Reactome | R-HSA-2029485 | 1 | 24 | 1.68E-01 | 2.99E-01 | PIK3CB |
| Potassium Channels | Reactome | R-HSA-1296071 | 2 | 99 | 1.69E-01 | 3.00E-01 | ABCC8|KCNV2 |
| Post-translational protein modification | Reactome | R-HSA-597592 | 9 | 840 | 1.72E-01 | 3.04E-01 | USP17L11|ATXN3|CFTR|OTUD7A|GCNT3|MGAT4C|SP100|COG4|MUC2 |
| Processing of Capped Intronless Pre-mRNA | Reactome | R-HSA-75067 | 1 | 25 | 1.74E-01 | 3.04E-01 | CPSF7 |
| Gap-filling DNA repair synthesis and ligation in GG-NER | Reactome | R-HSA-5696397 | 1 | 25 | 1.74E-01 | 3.04E-01 | POLD1 |
| TNFs bind their physiological receptors | Reactome | R-HSA-5669034 | 1 | 25 | 1.74E-01 | 3.04E-01 | EDA2R |
| Termination of O-glycan biosynthesis | Reactome | R-HSA-977068 | 1 | 25 | 1.74E-01 | 3.04E-01 | MUC2 |
| FGFR1 mutant receptor activation | Reactome | R-HSA-1839124 | 1 | 25 | 1.74E-01 | 3.04E-01 | BCR |
| Peroxisomal lipid metabolism | Reactome | R-HSA-390918 | 1 | 25 | 1.74E-01 | 3.04E-01 | ACOT8 |
| Metal ion SLC transporters | Reactome | R-HSA-425410 | 1 | 25 | 1.74E-01 | 3.04E-01 | HEPH |
| Synthesis of very long-chain fatty acyl-CoAs | Reactome | R-HSA-75876 | 1 | 25 | 1.74E-01 | 3.04E-01 | TECRL |
| Transport of glucose and other sugars, bile salts and organic acids, metal ions and amine compounds | Reactome | R-HSA-425366 | 2 | 101 | 1.74E-01 | 3.04E-01 | HEPH|SLC10A6 |
| Deubiquitination | Reactome | R-HSA-5688426 | 4 | 294 | 1.76E-01 | 3.06E-01 | USP17L11|OTUD7A|ATXN3|CFTR |
| Cell surface interactions at the vascular wall | Reactome | R-HSA-202733 | 2 | 102 | 1.77E-01 | 3.07E-01 | ITGAL|PIK3CB |
| N-glycan antennae elongation in the medial/trans-Golgi | Reactome | R-HSA-975576 | 1 | 26 | 1.80E-01 | 3.09E-01 | MGAT4C |
| Synthesis of bile acids and bile salts | Reactome | R-HSA-192105 | 1 | 26 | 1.80E-01 | 3.09E-01 | ACOT8 |
| FGFR2 alternative splicing | Reactome | R-HSA-6803529 | 1 | 26 | 1.80E-01 | 3.09E-01 | ESRP1 |
| RNA Pol II CTD phosphorylation and interaction with CE | Reactome | R-HSA-77075 | 1 | 27 | 1.86E-01 | 3.14E-01 | GTF2H4 |
| Acyl chain remodelling of PC | Reactome | R-HSA-1482788 | 1 | 27 | 1.86E-01 | 3.14E-01 | PNPLA8 |
| Syndecan interactions | Reactome | R-HSA-3000170 | 1 | 27 | 1.86E-01 | 3.14E-01 | TNC |
| NoRC negatively regulates rRNA expression | Reactome | R-HSA-427413 | 2 | 106 | 1.87E-01 | 3.15E-01 | GTF2H4|TAF1C |
| Signaling by Insulin receptor | Reactome | R-HSA-74752 | 4 | 303 | 1.89E-01 | 3.17E-01 | CSF2RB|TSC1|PIK3CB|PRKAG2 |
| Interleukin-7 signaling | Reactome | R-HSA-1266695 | 1 | 28 | 1.92E-01 | 3.19E-01 | SMARCA4 |
| Synthesis of IP3 and IP4 in the cytosol | Reactome | R-HSA-1855204 | 1 | 28 | 1.92E-01 | 3.19E-01 | PLCD1 |
| Signaling by NOTCH | Reactome | R-HSA-157118 | 2 | 108 | 1.93E-01 | 3.20E-01 | HDAC4|E2F3 |
| Negative epigenetic regulation of rRNA expression | Reactome | R-HSA-5250941 | 2 | 109 | 1.95E-01 | 3.23E-01 | GTF2H4|TAF1C |
| O-linked glycosylation | Reactome | R-HSA-5173105 | 2 | 109 | 1.95E-01 | 3.23E-01 | GCNT3|MUC2 |
| Integration of energy metabolism | Reactome | R-HSA-163685 | 2 | 109 | 1.95E-01 | 3.23E-01 | ABCC8|PRKAG2 |
| RNA Polymerase I Promoter Clearance | Reactome | R-HSA-73854 | 2 | 109 | 1.95E-01 | 3.23E-01 | GTF2H4|TAF1C |
| Acyl chain remodelling of PE | Reactome | R-HSA-1482839 | 1 | 29 | 1.98E-01 | 3.25E-01 | PNPLA8 |
| Myogenesis | Reactome | R-HSA-525793 | 1 | 29 | 1.98E-01 | 3.25E-01 | TCF3 |
| Pre-NOTCH Transcription and Translation | Reactome | R-HSA-1912408 | 1 | 29 | 1.98E-01 | 3.25E-01 | E2F3 |
| CDO in myogenesis | Reactome | R-HSA-375170 | 1 | 29 | 1.98E-01 | 3.25E-01 | TCF3 |
| mRNA Capping | Reactome | R-HSA-72086 | 1 | 29 | 1.98E-01 | 3.25E-01 | GTF2H4 |
| RNA Polymerase I Transcription | Reactome | R-HSA-73864 | 2 | 111 | 2.00E-01 | 3.28E-01 | GTF2H4|TAF1C |
| Transmission across Chemical Synapses | Reactome | R-HSA-112315 | 3 | 208 | 2.01E-01 | 3.29E-01 | TSPOAP1|PPFIA1|GAD2 |
| Glucagon-type ligand receptors | Reactome | R-HSA-420092 | 1 | 30 | 2.04E-01 | 3.29E-01 | GIPR |
| Recognition of DNA damage by PCNA-containing replication complex | Reactome | R-HSA-110314 | 1 | 30 | 2.04E-01 | 3.29E-01 | POLD1 |
| Oncogene Induced Senescence | Reactome | R-HSA-2559585 | 1 | 30 | 2.04E-01 | 3.29E-01 | E2F3 |
| Extension of Telomeres | Reactome | R-HSA-180786 | 1 | 30 | 2.04E-01 | 3.29E-01 | POLD1 |
| Hedgehog 'off' state | Reactome | R-HSA-5610787 | 2 | 113 | 2.06E-01 | 3.31E-01 | MKS1|IFT122 |
| Inwardly rectifying K+ channels | Reactome | R-HSA-1296065 | 1 | 31 | 2.09E-01 | 3.34E-01 | ABCC8 |
| Interaction between L1 and Ankyrins | Reactome | R-HSA-445095 | 1 | 31 | 2.09E-01 | 3.34E-01 | SCN1A |
| L1CAM interactions | Reactome | R-HSA-373760 | 2 | 115 | 2.11E-01 | 3.35E-01 | LAMC1|SCN1A |
| Detoxification of Reactive Oxygen Species | Reactome | R-HSA-3299685 | 1 | 32 | 2.15E-01 | 3.37E-01 | CCS |
| Termination of translesion DNA synthesis | Reactome | R-HSA-5656169 | 1 | 32 | 2.15E-01 | 3.37E-01 | POLD1 |
| DNA strand elongation | Reactome | R-HSA-69190 | 1 | 32 | 2.15E-01 | 3.37E-01 | POLD1 |
| Signaling by FGFR1 in disease | Reactome | R-HSA-5655302 | 1 | 32 | 2.15E-01 | 3.37E-01 | BCR |
| Signaling by FGFR1 | Reactome | R-HSA-5654736 | 2 | 325 | 6.92E-01 | 7.20E-01 | PIK3CB|CSF2RB |
| Selenoamino acid metabolism | Reactome | R-HSA-2408522 | 2 | 117 | 2.17E-01 | 3.39E-01 | PSTK|SCLY |
| Formation of the HIV-1 Early Elongation Complex | Reactome | R-HSA-167158 | 1 | 33 | 2.21E-01 | 3.43E-01 | GTF2H4 |
| Keratan sulfate/keratin metabolism | Reactome | R-HSA-1638074 | 1 | 33 | 2.21E-01 | 3.43E-01 | GALNS |
| Generation of second messenger molecules | Reactome | R-HSA-202433 | 1 | 33 | 2.21E-01 | 3.43E-01 | EVL |
| Formation of the Early Elongation Complex | Reactome | R-HSA-113418 | 1 | 33 | 2.21E-01 | 3.43E-01 | GTF2H4 |
| Base Excision Repair | Reactome | R-HSA-73884 | 1 | 36 | 2.38E-01 | 3.58E-01 | POLD1 |
| TCR signaling | Reactome | R-HSA-202403 | 2 | 119 | 2.22E-01 | 3.44E-01 | PIK3CB|EVL |
| Immunoregulatory interactions between a Lymphoid and a non-Lymphoid cell | Reactome | R-HSA-198933 | 2 | 120 | 2.25E-01 | 3.47E-01 | ITGAL|CD1B |
| Diseases of carbohydrate metabolism | Reactome | R-HSA-5663084 | 1 | 34 | 2.27E-01 | 3.49E-01 | GALNS |
| ROS, RNS production in response to bacteria | Reactome | R-HSA-1222556 | 1 | 34 | 2.27E-01 | 3.49E-01 | NOS1 |
| Bile acid and bile salt metabolism | Reactome | R-HSA-194068 | 1 | 34 | 2.27E-01 | 3.49E-01 | ACOT8 |
| Signaling by WNT in cancer | Reactome | R-HSA-4791275 | 1 | 34 | 2.27E-01 | 3.49E-01 | CTBP2 |
| Ovarian tumor domain proteases | Reactome | R-HSA-5689896 | 1 | 34 | 2.27E-01 | 3.49E-01 | OTUD7A |
| Signaling by Wnt | Reactome | R-HSA-195721 | 4 | 329 | 2.28E-01 | 3.50E-01 | TCF7L1|CTBP2|RSPO4|SMARCA4 |
| Synthesis of PIPs at the plasma membrane | Reactome | R-HSA-1660499 | 1 | 35 | 2.32E-01 | 3.53E-01 | PIK3CB |
| Platelet Aggregation (Plug Formation) | Reactome | R-HSA-76009 | 1 | 35 | 2.32E-01 | 3.53E-01 | GP1BA |
| Lysosome Vesicle Biogenesis | Reactome | R-HSA-432720 | 1 | 35 | 2.32E-01 | 3.53E-01 | AP1G2 |
| Resolution of Abasic Sites (AP sites) | Reactome | R-HSA-73933 | 1 | 36 | 2.38E-01 | 3.58E-01 | POLD1 |
| Glutathione conjugation | Reactome | R-HSA-156590 | 1 | 36 | 2.38E-01 | 3.58E-01 | GSTZ1 |
| Apoptotic cleavage of cellular proteins | Reactome | R-HSA-111465 | 1 | 36 | 2.38E-01 | 3.58E-01 | GSN |
| Metabolism of amino acids and derivatives | Reactome | R-HSA-71291 | 4 | 339 | 2.44E-01 | 3.61E-01 | GSTZ1|PSTK|SCLY|GAMT |
| Processing of Capped Intron-Containing Pre-mRNA | Reactome | R-HSA-72203 | 3 | 231 | 2.45E-01 | 3.64E-01 | CPSF7|CWC27|AQR |
| TCF dependent signaling in response to WNT | Reactome | R-HSA-201681 | 3 | 232 | 2.47E-01 | 3.66E-01 | TCF7L1|RSPO4|SMARCA4 |
| RHO GTPases Activate Formins | Reactome | R-HSA-5663220 | 2 | 129 | 2.49E-01 | 3.66E-01 | MKL1|EVL |
| G1 Phase | Reactome | R-HSA-69236 | 1 | 38 | 2.49E-01 | 3.66E-01 | E2F3 |
| Cyclin D associated events in G1 | Reactome | R-HSA-69231 | 1 | 38 | 2.49E-01 | 3.66E-01 | E2F3 |
| tRNA modification in the nucleus and cytosol | Reactome | R-HSA-6782315 | 1 | 38 | 2.49E-01 | 3.66E-01 | CDKAL1 |
| Association of TriC/CCT with target proteins during biosynthesis | Reactome | R-HSA-390471 | 1 | 38 | 2.49E-01 | 3.66E-01 | XRN2 |
| Vesicle-mediated transport | Reactome | R-HSA-5653656 | 6 | 573 | 2.51E-01 | 3.69E-01 | AP1G2|CD163|CFTR|PRKAG2|HIP1R|COG4 |
| Formation of Fibrin Clot (Clotting Cascade) | Reactome | R-HSA-140877 | 1 | 39 | 2.55E-01 | 3.71E-01 | GP1BA |
| Binding and Uptake of Ligands by Scavenger Receptors | Reactome | R-HSA-2173782 | 1 | 39 | 2.55E-01 | 3.71E-01 | CD163 |
| Translesion synthesis by Y family DNA polymerases bypasses lesions on DNA template | Reactome | R-HSA-110313 | 1 | 39 | 2.55E-01 | 3.71E-01 | POLD1 |
| Cell-Cell communication | Reactome | R-HSA-1500931 | 2 | 133 | 2.60E-01 | 3.75E-01 | SFTPA2|PIK3CB |
| Clathrin-mediated endocytosis | Reactome | R-HSA-8856828 | 2 | 133 | 2.60E-01 | 3.75E-01 | HIP1R|CFTR |
| EPHB-mediated forward signaling | Reactome | R-HSA-3928662 | 1 | 41 | 2.65E-01 | 3.80E-01 | EPHB4 |
| Netrin-1 signaling | Reactome | R-HSA-373752 | 1 | 41 | 2.65E-01 | 3.80E-01 | ROBO1 |
| Histidine, lysine, phenylalanine, tyrosine, proline and tryptophan catabolism | Reactome | R-HSA-6788656 | 1 | 41 | 2.65E-01 | 3.80E-01 | GSTZ1 |
| Retinoid metabolism and transport | Reactome | R-HSA-975634 | 1 | 42 | 2.71E-01 | 3.84E-01 | CLPS |
| Deactivation of the beta-catenin transactivating complex | Reactome | R-HSA-3769402 | 1 | 42 | 2.71E-01 | 3.84E-01 | TCF7L1 |
| Voltage gated Potassium channels | Reactome | R-HSA-1296072 | 1 | 43 | 2.76E-01 | 3.89E-01 | KCNV2 |
| Formation of Incision Complex in GG-NER | Reactome | R-HSA-5696395 | 1 | 43 | 2.76E-01 | 3.89E-01 | GTF2H4 |
| Iron uptake and transport | Reactome | R-HSA-917937 | 1 | 43 | 2.76E-01 | 3.89E-01 | HEPH |
| Cardiac conduction | Reactome | R-HSA-5576891 | 2 | 141 | 2.81E-01 | 3.93E-01 | SCN1A|NOS1 |
| Intra-Golgi traffic | Reactome | R-HSA-6811438 | 1 | 44 | 2.81E-01 | 3.93E-01 | COG4 |
| Complement cascade | Reactome | R-HSA-166658 | 1 | 44 | 2.81E-01 | 3.93E-01 | CFD |
| RET signaling | Reactome | R-HSA-8853659 | 3 | 251 | 2.85E-01 | 3.97E-01 | PIK3CB|DOK4|CSF2RB |
| RNA Polymerase II Transcription | Reactome | R-HSA-73857 | 2 | 143 | 2.87E-01 | 3.97E-01 | CPSF7|GTF2H4 |
| Nuclear Envelope Breakdown | Reactome | R-HSA-2980766 | 1 | 45 | 2.87E-01 | 3.97E-01 | LEMD3 |
| NOTCH1 Intracellular Domain Regulates Transcription | Reactome | R-HSA-2122947 | 1 | 45 | 2.87E-01 | 3.97E-01 | HDAC4 |
| Pre-NOTCH Expression and Processing | Reactome | R-HSA-1912422 | 1 | 45 | 2.87E-01 | 3.97E-01 | E2F3 |
| Phase 0 - rapid depolarisation | Reactome | R-HSA-5576892 | 1 | 46 | 2.92E-01 | 4.00E-01 | SCN1A |
| HIV Transcription Initiation | Reactome | R-HSA-167161 | 1 | 46 | 2.92E-01 | 4.00E-01 | GTF2H4 |
| RNA Polymerase II Transcription Initiation And Promoter Clearance | Reactome | R-HSA-76042 | 1 | 46 | 2.92E-01 | 4.00E-01 | GTF2H4 |
| RNA Polymerase II HIV Promoter Escape | Reactome | R-HSA-167162 | 1 | 46 | 2.92E-01 | 4.00E-01 | GTF2H4 |
| RNA Polymerase II Transcription Pre-Initiation And Promoter Opening | Reactome | R-HSA-73779 | 1 | 46 | 2.92E-01 | 4.00E-01 | GTF2H4 |
| RNA Polymerase II Transcription Initiation | Reactome | R-HSA-75953 | 1 | 46 | 2.92E-01 | 4.00E-01 | GTF2H4 |
| RNA Polymerase II Promoter Escape | Reactome | R-HSA-73776 | 1 | 46 | 2.92E-01 | 4.00E-01 | GTF2H4 |
| Epigenetic regulation of gene expression | Reactome | R-HSA-212165 | 2 | 146 | 2.95E-01 | 4.03E-01 | GTF2H4|TAF1C |
| DNA Damage Bypass | Reactome | R-HSA-73893 | 1 | 48 | 3.02E-01 | 4.08E-01 | POLD1 |
| RNA Polymerase I, RNA Polymerase III, and Mitochondrial Transcription | Reactome | R-HSA-504046 | 2 | 149 | 3.03E-01 | 4.08E-01 | GTF2H4|TAF1C |
| Toll-Like Receptors Cascades | Reactome | R-HSA-168898 | 2 | 150 | 3.06E-01 | 4.11E-01 | CTSS|SFTPA2 |
| HIV Transcription Elongation | Reactome | R-HSA-167169 | 1 | 49 | 3.07E-01 | 4.12E-01 | GTF2H4 |
| Tat-mediated elongation of the HIV-1 transcript | Reactome | R-HSA-167246 | 1 | 49 | 3.07E-01 | 4.12E-01 | GTF2H4 |
| Mitochondrial biogenesis | Reactome | R-HSA-1592230 | 1 | 49 | 3.07E-01 | 4.12E-01 | PRKAG2 |
| Formation of HIV-1 elongation complex containing HIV-1 Tat | Reactome | R-HSA-167200 | 1 | 49 | 3.07E-01 | 4.12E-01 | GTF2H4 |
| Constitutive Signaling by Aberrant PI3K in Cancer | Reactome | R-HSA-2219530 | 1 | 49 | 3.07E-01 | 4.12E-01 | PIK3CB |
| Retrograde transport at the Trans-Golgi-Network | Reactome | R-HSA-6811440 | 1 | 49 | 3.07E-01 | 4.12E-01 | COG4 |
| HDMs demethylate histones | Reactome | R-HSA-3214842 | 1 | 50 | 3.12E-01 | 4.16E-01 | KDM4C |
| Chromatin organization | Reactome | R-HSA-4839726 | 3 | 265 | 3.13E-01 | 4.16E-01 | KMT2B|KDM4C|SMARCA4 |
| SLC-mediated transmembrane transport | Reactome | R-HSA-425407 | 3 | 265 | 3.13E-01 | 4.16E-01 | HEPH|SLC10A6|SLC12A4 |
| Chromatin modifying enzymes | Reactome | R-HSA-3247509 | 3 | 265 | 3.13E-01 | 4.16E-01 | KMT2B|KDM4C|SMARCA4 |
| Cell Cycle, Mitotic | Reactome | R-HSA-69278 | 5 | 503 | 3.15E-01 | 4.19E-01 | POLD1|LEMD3|PCM1|CEP70|E2F3 |
| Metabolism of fat-soluble vitamins | Reactome | R-HSA-6806667 | 1 | 51 | 3.18E-01 | 4.20E-01 | CLPS |
| Formation of HIV elongation complex in the absence of HIV Tat | Reactome | R-HSA-167152 | 1 | 51 | 3.18E-01 | 4.20E-01 | GTF2H4 |
| Golgi Associated Vesicle Biogenesis | Reactome | R-HSA-432722 | 1 | 52 | 3.23E-01 | 4.22E-01 | HIP1R |
| Aquaporin-mediated transport | Reactome | R-HSA-445717 | 1 | 52 | 3.23E-01 | 4.22E-01 | AQP7 |
| Intraflagellar transport | Reactome | R-HSA-5620924 | 1 | 54 | 3.32E-01 | 4.31E-01 | IFT122 |
| Assembly of collagen fibrils and other multimeric structures | Reactome | R-HSA-2022090 | 1 | 54 | 3.32E-01 | 4.31E-01 | CTSS |
| mRNA 3'-end processing | Reactome | R-HSA-72187 | 1 | 55 | 3.37E-01 | 4.35E-01 | CPSF7 |
| Signaling by NOTCH1 in Cancer | Reactome | R-HSA-2644603 | 1 | 56 | 3.42E-01 | 4.39E-01 | HDAC4 |
| Ion homeostasis | Reactome | R-HSA-5578775 | 1 | 56 | 3.42E-01 | 4.39E-01 | NOS1 |
| Constitutive Signaling by NOTCH1 PEST Domain Mutants | Reactome | R-HSA-2644606 | 1 | 56 | 3.42E-01 | 4.39E-01 | HDAC4 |
| Signaling by NOTCH1 PEST Domain Mutants in Cancer | Reactome | R-HSA-2644602 | 1 | 56 | 3.42E-01 | 4.39E-01 | HDAC4 |
| Signaling by NOTCH1 HD+PEST Domain Mutants in Cancer | Reactome | R-HSA-2894858 | 1 | 56 | 3.42E-01 | 4.39E-01 | HDAC4 |
| Constitutive Signaling by NOTCH1 HD+PEST Domain Mutants | Reactome | R-HSA-2894862 | 1 | 56 | 3.42E-01 | 4.39E-01 | HDAC4 |
| Programmed Cell Death | Reactome | R-HSA-5357801 | 2 | 164 | 3.43E-01 | 4.40E-01 | GSN|HIST1H1A |
| Signaling by FGFR in disease | Reactome | R-HSA-1226099 | 1 | 57 | 3.47E-01 | 4.42E-01 | BCR |
| Signaling by FGFR | Reactome | R-HSA-190236 | 3 | 355 | 4.88E-01 | 5.56E-01 | PIK3CB|ESRP1|CSF2RB |
| RNA Polymerase II Transcription Elongation | Reactome | R-HSA-75955 | 1 | 57 | 3.47E-01 | 4.42E-01 | GTF2H4 |
| Formation of RNA Pol II elongation complex | Reactome | R-HSA-112382 | 1 | 57 | 3.47E-01 | 4.42E-01 | GTF2H4 |
| Membrane Trafficking | Reactome | R-HSA-199991 | 5 | 537 | 3.64E-01 | 4.56E-01 | CFTR|HIP1R|AP1G2|COG4|PRKAG2 |
| Ca2+ pathway | Reactome | R-HSA-4086398 | 1 | 61 | 3.66E-01 | 4.56E-01 | TCF7L1 |
| Defective CFTR causes cystic fibrosis | Reactome | R-HSA-5678895 | 1 | 61 | 3.66E-01 | 4.56E-01 | CFTR |
| Cytochrome P450 - arranged by substrate type | Reactome | R-HSA-211897 | 1 | 63 | 3.75E-01 | 4.62E-01 | CYP2F1 |
| Cleavage of Growing Transcript in the Termination Region | Reactome | R-HSA-109688 | 1 | 64 | 3.80E-01 | 4.67E-01 | CPSF7 |
| RNA Polymerase II Transcription Termination | Reactome | R-HSA-73856 | 1 | 64 | 3.80E-01 | 4.67E-01 | CPSF7 |
| PKMTs methylate histone lysines | Reactome | R-HSA-3214841 | 1 | 64 | 3.80E-01 | 4.67E-01 | KMT2B |
| Transport to the Golgi and subsequent modification | Reactome | R-HSA-948021 | 2 | 180 | 3.85E-01 | 4.71E-01 | MGAT4C|COG4 |
| Immune System | Reactome | R-HSA-168256 | 13 | 1583 | 3.86E-01 | 4.72E-01 | KLRC2|SMARCA4|ITGAL|SP100|SFTPA2|CTSS|NOS1|EDA2R|EVL|CFD|CSF2RB|CD1B|PIK3CB |
| Activated PKN1 stimulates transcription of AR (androgen receptor) regulated genes KLK2 and KLK3 | Reactome | R-HSA-5625886 | 1 | 67 | 3.93E-01 | 4.78E-01 | KDM4C |
| HDR through Homologous Recombination (HRR) | Reactome | R-HSA-5685942 | 1 | 67 | 3.93E-01 | 4.78E-01 | POLD1 |
| Diseases associated with O-glycosylation of proteins | Reactome | R-HSA-3906995 | 1 | 67 | 3.93E-01 | 4.78E-01 | MUC2 |
| TP53 Regulates Transcription of DNA Repair Genes | Reactome | R-HSA-6796648 | 1 | 68 | 3.98E-01 | 4.81E-01 | GTF2H4 |
| SIRT1 negatively regulates rRNA Expression | Reactome | R-HSA-427359 | 1 | 68 | 3.98E-01 | 4.81E-01 | TAF1C |
| Assembly of the pre-replicative complex | Reactome | R-HSA-68867 | 1 | 68 | 3.98E-01 | 4.81E-01 | E2F3 |
| G2/M Transition | Reactome | R-HSA-69275 | 2 | 185 | 3.98E-01 | 4.81E-01 | PCM1|CEP70 |
| SUMOylation of DNA damage response and repair proteins | Reactome | R-HSA-3108214 | 1 | 70 | 4.07E-01 | 4.89E-01 | SP100 |
| Signaling by NOTCH1 | Reactome | R-HSA-1980143 | 1 | 70 | 4.07E-01 | 4.89E-01 | HDAC4 |
| PI5P, PP2A and IER3 Regulate PI3K/AKT Signaling | Reactome | R-HSA-6811558 | 1 | 70 | 4.07E-01 | 4.89E-01 | PIK3CB |
| Cellular Senescence | Reactome | R-HSA-2559583 | 2 | 192 | 4.16E-01 | 4.97E-01 | HIST1H1A|E2F3 |
| PI3K/AKT Signaling in Cancer | Reactome | R-HSA-2219528 | 1 | 74 | 4.24E-01 | 5.05E-01 | PIK3CB |
| Peptide ligand-binding receptors | Reactome | R-HSA-375276 | 2 | 196 | 4.26E-01 | 5.07E-01 | NPY4R|SSTR1 |
| Transcription of the HIV genome | Reactome | R-HSA-167172 | 1 | 75 | 4.28E-01 | 5.08E-01 | GTF2H4 |
| Biological oxidations | Reactome | R-HSA-211859 | 2 | 198 | 4.31E-01 | 5.11E-01 | GSTZ1|CYP2F1 |
| Regulation of DNA replication | Reactome | R-HSA-69304 | 1 | 76 | 4.32E-01 | 5.12E-01 | E2F3 |
| RMTs methylate histone arginines | Reactome | R-HSA-3214858 | 1 | 77 | 4.36E-01 | 5.16E-01 | SMARCA4 |
| Negative regulation of the PI3K/AKT network | Reactome | R-HSA-199418 | 1 | 77 | 4.36E-01 | 5.16E-01 | PIK3CB |
| RNA Polymerase II Pre-transcription Events | Reactome | R-HSA-674695 | 1 | 79 | 4.45E-01 | 5.22E-01 | GTF2H4 |
| Muscle contraction | Reactome | R-HSA-397014 | 2 | 204 | 4.46E-01 | 5.23E-01 | SCN1A|NOS1 |
| DAP12 signaling | Reactome | R-HSA-2424491 | 3 | 333 | 4.46E-01 | 5.24E-01 | PIK3CB|KLRC2|CSF2RB |
| Regulation of insulin secretion | Reactome | R-HSA-422356 | 1 | 80 | 4.49E-01 | 5.24E-01 | ABCC8 |
| DNA Damage/Telomere Stress Induced Senescence | Reactome | R-HSA-2559586 | 1 | 80 | 4.49E-01 | 5.24E-01 | HIST1H1A |
| Peptide hormone metabolism | Reactome | R-HSA-2980736 | 1 | 81 | 4.53E-01 | 5.27E-01 | ACE2 |
| Translocation of GLUT4 to the plasma membrane | Reactome | R-HSA-1445148 | 1 | 81 | 4.53E-01 | 5.27E-01 | PRKAG2 |
| Fcgamma receptor (FCGR) dependent phagocytosis | Reactome | R-HSA-2029480 | 1 | 82 | 4.57E-01 | 5.31E-01 | PIK3CB |
| Telomere Maintenance | Reactome | R-HSA-157579 | 1 | 82 | 4.57E-01 | 5.31E-01 | POLD1 |
| Hedgehog 'on' state | Reactome | R-HSA-5632684 | 1 | 83 | 4.61E-01 | 5.34E-01 | EVC |
| Cell Cycle | Reactome | R-HSA-1640170 | 5 | 607 | 4.65E-01 | 5.38E-01 | POLD1|LEMD3|PCM1|CEP70|E2F3 |
| DNA Replication Pre-Initiation | Reactome | R-HSA-69002 | 1 | 85 | 4.69E-01 | 5.41E-01 | E2F3 |
| M/G1 Transition | Reactome | R-HSA-68874 | 1 | 85 | 4.69E-01 | 5.41E-01 | E2F3 |
| Metabolism of polyamines | Reactome | R-HSA-351202 | 1 | 85 | 4.69E-01 | 5.41E-01 | GAMT |
| DAP12 interactions | Reactome | R-HSA-2172127 | 3 | 345 | 4.69E-01 | 5.41E-01 | PIK3CB|KLRC2|CSF2RB |
| Signaling by FGFR2 | Reactome | R-HSA-5654738 | 3 | 349 | 4.76E-01 | 5.48E-01 | PIK3CB|ESRP1|CSF2RB |
| Collagen formation | Reactome | R-HSA-1474290 | 1 | 88 | 4.80E-01 | 5.51E-01 | CTSS |
| Class B/2 (Secretin family receptors) | Reactome | R-HSA-373080 | 1 | 88 | 4.80E-01 | 5.51E-01 | GIPR |
| Platelet homeostasis | Reactome | R-HSA-418346 | 1 | 88 | 4.80E-01 | 5.51E-01 | NOS1 |
| Ub-specific processing proteases | Reactome | R-HSA-5689880 | 2 | 220 | 4.84E-01 | 5.53E-01 | USP17L11|CFTR |
| Cargo recognition for clathrin-mediated endocytosis | Reactome | R-HSA-8856825 | 1 | 90 | 4.88E-01 | 5.56E-01 | CFTR |
| B-WICH complex positively regulates rRNA expression | Reactome | R-HSA-5250924 | 1 | 90 | 4.88E-01 | 5.56E-01 | TAF1C |
| Cytokine Signaling in Immune system | Reactome | R-HSA-1280215 | 5 | 624 | 4.88E-01 | 5.56E-01 | SP100|CSF2RB|PIK3CB|EDA2R|SMARCA4 |
| Signaling by PDGF | Reactome | R-HSA-186797 | 3 | 356 | 4.89E-01 | 5.58E-01 | PIK3CB|SPP1|CSF2RB |
| Interferon gamma signaling | Reactome | R-HSA-877300 | 1 | 91 | 4.91E-01 | 5.59E-01 | SP100 |
| Regulation of TP53 Activity through Phosphorylation | Reactome | R-HSA-6804756 | 1 | 91 | 4.91E-01 | 5.59E-01 | PRKAG2 |
| Metabolism of nucleotides | Reactome | R-HSA-15869 | 1 | 92 | 4.95E-01 | 5.61E-01 | XDH |
| Phase 1 - Functionalization of compounds | Reactome | R-HSA-211945 | 1 | 92 | 4.95E-01 | 5.61E-01 | CYP2F1 |
| RHO GTPases activate PKNs | Reactome | R-HSA-5625740 | 1 | 93 | 4.99E-01 | 5.65E-01 | KDM4C |
| Selenocysteine synthesis | Reactome | R-HSA-2408557 | 1 | 93 | 4.99E-01 | 5.65E-01 | PSTK |
| SUMO E3 ligases SUMOylate target proteins | Reactome | R-HSA-3108232 | 1 | 93 | 4.99E-01 | 5.65E-01 | SP100 |
| Innate Immune System | Reactome | R-HSA-168249 | 6 | 769 | 5.01E-01 | 5.67E-01 | KLRC2|CSF2RB|CTSS|CFD|PIK3CB|SFTPA2 |
| Chaperonin-mediated protein folding | Reactome | R-HSA-390466 | 1 | 94 | 5.03E-01 | 5.68E-01 | XRN2 |
| Toll Like Receptor 2 (TLR2) Cascade | Reactome | R-HSA-181438 | 1 | 95 | 5.06E-01 | 5.71E-01 | SFTPA2 |
| Toll Like Receptor TLR1:TLR2 Cascade | Reactome | R-HSA-168179 | 1 | 95 | 5.06E-01 | 5.71E-01 | SFTPA2 |
| COPI-mediated anterograde transport | Reactome | R-HSA-6807878 | 1 | 96 | 5.10E-01 | 5.74E-01 | COG4 |
| TNFR2 non-canonical NF-kB pathway | Reactome | R-HSA-5668541 | 1 | 97 | 5.13E-01 | 5.77E-01 | EDA2R |
| Amyloid fiber formation | Reactome | R-HSA-977225 | 1 | 97 | 5.13E-01 | 5.77E-01 | GSN |
| Lipid digestion, mobilization, and transport | Reactome | R-HSA-73923 | 1 | 97 | 5.13E-01 | 5.77E-01 | CLPS |
| SUMOylation | Reactome | R-HSA-2990846 | 1 | 98 | 5.17E-01 | 5.80E-01 | SP100 |
| tRNA processing | Reactome | R-HSA-72306 | 1 | 98 | 5.17E-01 | 5.80E-01 | CDKAL1 |
| Downstream TCR signaling | Reactome | R-HSA-202424 | 1 | 98 | 5.17E-01 | 5.80E-01 | PIK3CB |
| Antigen processing-Cross presentation | Reactome | R-HSA-1236975 | 1 | 99 | 5.21E-01 | 5.83E-01 | CTSS |
| Interleukin receptor SHC signaling | Reactome | R-HSA-912526 | 2 | 236 | 5.21E-01 | 5.83E-01 | PIK3CB|CSF2RB |
| Protein folding | Reactome | R-HSA-391251 | 1 | 100 | 5.24E-01 | 5.85E-01 | XRN2 |
| Synthesis of DNA | Reactome | R-HSA-69239 | 1 | 100 | 5.24E-01 | 5.85E-01 | POLD1 |
| Transport of inorganic cations/anions and amino acids/oligopeptides | Reactome | R-HSA-425393 | 1 | 100 | 5.24E-01 | 5.85E-01 | SLC12A4 |
| Phase II conjugation | Reactome | R-HSA-156580 | 1 | 100 | 5.24E-01 | 5.85E-01 | GSTZ1 |
| Interleukin-2 signaling | Reactome | R-HSA-451927 | 2 | 243 | 5.36E-01 | 5.96E-01 | PIK3CB|CSF2RB |
| Positive epigenetic regulation of rRNA expression | Reactome | R-HSA-5250913 | 1 | 105 | 5.41E-01 | 5.99E-01 | TAF1C |
| PI-3K cascade:FGFR4 | Reactome | R-HSA-5654720 | 1 | 106 | 5.45E-01 | 6.01E-01 | PIK3CB |
| PI-3K cascade:FGFR3 | Reactome | R-HSA-5654710 | 1 | 106 | 5.45E-01 | 6.01E-01 | PIK3CB |
| PI-3K cascade:FGFR1 | Reactome | R-HSA-5654689 | 1 | 106 | 5.45E-01 | 6.01E-01 | PIK3CB |
| PI3K events in ERBB4 signaling | Reactome | R-HSA-1250342 | 1 | 106 | 5.45E-01 | 6.01E-01 | PIK3CB |
| PIP3 activates AKT signaling | Reactome | R-HSA-1257604 | 1 | 106 | 5.45E-01 | 6.01E-01 | PIK3CB |
| PI-3K cascade:FGFR2 | Reactome | R-HSA-5654695 | 1 | 106 | 5.45E-01 | 6.01E-01 | PIK3CB |
| GAB1 signalosome | Reactome | R-HSA-180292 | 1 | 109 | 5.55E-01 | 6.09E-01 | PIK3CB |
| Glycerophospholipid biosynthesis | Reactome | R-HSA-1483206 | 1 | 109 | 5.55E-01 | 6.09E-01 | PNPLA8 |
| PI3K/AKT activation | Reactome | R-HSA-198203 | 1 | 109 | 5.55E-01 | 6.09E-01 | PIK3CB |
| Interleukin-3, 5 and GM-CSF signaling | Reactome | R-HSA-512988 | 2 | 252 | 5.56E-01 | 6.10E-01 | PIK3CB|CSF2RB |
| Chromosome Maintenance | Reactome | R-HSA-73886 | 1 | 110 | 5.58E-01 | 6.11E-01 | POLD1 |
| Signaling by Interleukins | Reactome | R-HSA-449147 | 3 | 400 | 5.67E-01 | 6.19E-01 | CSF2RB|PIK3CB|SMARCA4 |
| Role of LAT2/NTAL/LAB on calcium mobilization | Reactome | R-HSA-2730905 | 1 | 114 | 5.71E-01 | 6.21E-01 | PIK3CB |
| MHC class II antigen presentation | Reactome | R-HSA-2132295 | 1 | 119 | 5.86E-01 | 6.32E-01 | CTSS |
| Oxidative Stress Induced Senescence | Reactome | R-HSA-2559580 | 1 | 122 | 5.95E-01 | 6.39E-01 | E2F3 |
| Glycosaminoglycan metabolism | Reactome | R-HSA-1630316 | 1 | 122 | 5.95E-01 | 6.39E-01 | GALNS |
| Toll Like Receptor 4 (TLR4) Cascade | Reactome | R-HSA-166016 | 1 | 125 | 6.04E-01 | 6.47E-01 | SFTPA2 |
| S Phase | Reactome | R-HSA-69242 | 1 | 128 | 6.13E-01 | 6.55E-01 | POLD1 |
| Platelet degranulation | Reactome | R-HSA-114608 | 1 | 128 | 6.13E-01 | 6.55E-01 | CFD |
| Gene Expression | Reactome | R-HSA-74160 | 12 | 1719 | 6.16E-01 | 6.58E-01 | CDKAL1|CPSF7|CWC27|TSC1|XRN2|CNOT2|TAF1C|CNOT1|GTF2H4|PRKAG2|PAN2|AQR |
| HDR through Homologous Recombination (HR) or Single Strand Annealing (SSA) | Reactome | R-HSA-5693567 | 1 | 132 | 6.24E-01 | 6.65E-01 | POLD1 |
| Mitotic Prophase | Reactome | R-HSA-68875 | 1 | 132 | 6.24E-01 | 6.65E-01 | LEMD3 |
| Response to elevated platelet cytosolic Ca2+ | Reactome | R-HSA-76005 | 1 | 133 | 6.27E-01 | 6.67E-01 | CFD |
| Late Phase of HIV Life Cycle | Reactome | R-HSA-162599 | 1 | 134 | 6.29E-01 | 6.69E-01 | GTF2H4 |
| Diseases of glycosylation | Reactome | R-HSA-3781865 | 1 | 136 | 6.35E-01 | 6.74E-01 | MUC2 |
| Homology Directed Repair | Reactome | R-HSA-5693538 | 1 | 138 | 6.40E-01 | 6.78E-01 | POLD1 |
| Mitotic G1-G1/S phases | Reactome | R-HSA-453279 | 1 | 139 | 6.43E-01 | 6.80E-01 | E2F3 |
| Asparagine N-linked glycosylation | Reactome | R-HSA-446203 | 2 | 299 | 6.48E-01 | 6.85E-01 | MGAT4C|COG4 |
| G alpha (s) signalling events | Reactome | R-HSA-418555 | 1 | 142 | 6.51E-01 | 6.87E-01 | GIPR |
| Beta-catenin independent WNT signaling | Reactome | R-HSA-3858494 | 1 | 145 | 6.58E-01 | 6.93E-01 | TCF7L1 |
| HIV Life Cycle | Reactome | R-HSA-162587 | 1 | 145 | 6.58E-01 | 6.93E-01 | GTF2H4 |
| VEGFA-VEGFR2 Pathway | Reactome | R-HSA-4420097 | 2 | 309 | 6.66E-01 | 7.00E-01 | PIK3CB|CSF2RB |
| ER to Golgi Anterograde Transport | Reactome | R-HSA-199977 | 1 | 149 | 6.68E-01 | 7.02E-01 | COG4 |
| Signaling by SCF-KIT | Reactome | R-HSA-1433557 | 2 | 313 | 6.72E-01 | 7.05E-01 | PIK3CB|CSF2RB |
| Regulation of TP53 Activity | Reactome | R-HSA-5633007 | 1 | 152 | 6.76E-01 | 7.08E-01 | PRKAG2 |
| Signaling by ERBB4 | Reactome | R-HSA-1236394 | 2 | 317 | 6.79E-01 | 7.11E-01 | PIK3CB|CSF2RB |
| Downstream signaling of activated FGFR4 | Reactome | R-HSA-5654716 | 2 | 317 | 6.79E-01 | 7.11E-01 | PIK3CB|CSF2RB |
| Downstream signaling of activated FGFR3 | Reactome | R-HSA-5654708 | 2 | 317 | 6.79E-01 | 7.11E-01 | PIK3CB|CSF2RB |
| Downstream signaling of activated FGFR2 | Reactome | R-HSA-5654696 | 2 | 317 | 6.79E-01 | 7.11E-01 | PIK3CB|CSF2RB |
| Signaling by VEGF | Reactome | R-HSA-194138 | 2 | 318 | 6.81E-01 | 7.12E-01 | PIK3CB|CSF2RB |
| Signaling by FGFR4 | Reactome | R-HSA-5654743 | 2 | 320 | 6.84E-01 | 7.15E-01 | PIK3CB|CSF2RB |
| Downstream signaling of activated FGFR1 | Reactome | R-HSA-5654687 | 2 | 320 | 6.84E-01 | 7.15E-01 | PIK3CB|CSF2RB |
| Signaling by FGFR3 | Reactome | R-HSA-5654741 | 2 | 321 | 6.86E-01 | 7.16E-01 | PIK3CB|CSF2RB |
| Adaptive Immune System | Reactome | R-HSA-1280218 | 5 | 787 | 6.90E-01 | 7.19E-01 | ITGAL|PIK3CB|CTSS|CD1B|EVL |
| Class A/1 (Rhodopsin-like receptors) | Reactome | R-HSA-373076 | 2 | 325 | 6.92E-01 | 7.20E-01 | NPY4R|SSTR1 |
| Downstream signal transduction | Reactome | R-HSA-186763 | 2 | 329 | 6.99E-01 | 7.26E-01 | PIK3CB|CSF2RB |
| DNA Double-Strand Break Repair | Reactome | R-HSA-5693532 | 1 | 166 | 7.07E-01 | 7.34E-01 | POLD1 |
| Signaling by EGFR | Reactome | R-HSA-177929 | 2 | 338 | 7.13E-01 | 7.38E-01 | PIK3CB|CSF2RB |
| Factors involved in megakaryocyte development and platelet production | Reactome | R-HSA-983231 | 1 | 171 | 7.18E-01 | 7.42E-01 | DOCK5 |
| Major pathway of rRNA processing in the nucleolus and cytosol | Reactome | R-HSA-6791226 | 1 | 173 | 7.22E-01 | 7.46E-01 | XRN2 |
| Generic Transcription Pathway | Reactome | R-HSA-212436 | 5 | 822 | 7.25E-01 | 7.49E-01 | CNOT1|TSC1|CNOT2|GTF2H4|PRKAG2 |
| Downstream signaling events of B Cell Receptor (BCR) | Reactome | R-HSA-1168372 | 1 | 177 | 7.30E-01 | 7.53E-01 | PIK3CB |
| Fc epsilon receptor (FCERI) signaling | Reactome | R-HSA-2454202 | 2 | 350 | 7.30E-01 | 7.53E-01 | PIK3CB|CSF2RB |
| rRNA processing in the nucleus and cytosol | Reactome | R-HSA-8868773 | 1 | 184 | 7.44E-01 | 7.64E-01 | XRN2 |
| NGF signalling via TRKA from the plasma membrane | Reactome | R-HSA-187037 | 2 | 363 | 7.48E-01 | 7.68E-01 | PIK3CB|CSF2RB |
| Mitotic Anaphase | Reactome | R-HSA-68882 | 1 | 188 | 7.51E-01 | 7.70E-01 | LEMD3 |
| Mitotic Metaphase and Anaphase | Reactome | R-HSA-2555396 | 1 | 189 | 7.53E-01 | 7.71E-01 | LEMD3 |
| Interferon Signaling | Reactome | R-HSA-913531 | 1 | 191 | 7.57E-01 | 7.75E-01 | SP100 |
| rRNA processing | Reactome | R-HSA-72312 | 1 | 194 | 7.62E-01 | 7.79E-01 | XRN2 |
| Intra-Golgi and retrograde Golgi-to-ER traffic | Reactome | R-HSA-6811442 | 1 | 195 | 7.64E-01 | 7.81E-01 | COG4 |
| Signaling by the B Cell Receptor (BCR) | Reactome | R-HSA-983705 | 1 | 207 | 7.84E-01 | 7.98E-01 | PIK3CB |
| HIV Infection | Reactome | R-HSA-162906 | 1 | 224 | 8.09E-01 | 8.22E-01 | GTF2H4 |
| SHC1 events in EGFR signaling | Reactome | R-HSA-180336 | 1 | 226 | 8.12E-01 | 8.24E-01 | CSF2RB |
| SOS-mediated signalling | Reactome | R-HSA-112412 | 1 | 226 | 8.12E-01 | 8.24E-01 | CSF2RB |
| GRB2 events in EGFR signaling | Reactome | R-HSA-179812 | 1 | 226 | 8.12E-01 | 8.24E-01 | CSF2RB |
| SHC1 events in ERBB4 signaling | Reactome | R-HSA-1250347 | 1 | 226 | 8.12E-01 | 8.24E-01 | CSF2RB |
| RAF/MAP kinase cascade | Reactome | R-HSA-5673001 | 1 | 226 | 8.12E-01 | 8.24E-01 | CSF2RB |
| FRS-mediated FGFR1 signaling | Reactome | R-HSA-5654693 | 1 | 227 | 8.13E-01 | 8.25E-01 | CSF2RB |
| FRS-mediated FGFR3 signaling | Reactome | R-HSA-5654706 | 1 | 227 | 8.13E-01 | 8.25E-01 | CSF2RB |
| FRS-mediated FGFR4 signaling | Reactome | R-HSA-5654712 | 1 | 227 | 8.13E-01 | 8.25E-01 | CSF2RB |
| FRS-mediated FGFR2 signaling | Reactome | R-HSA-5654700 | 1 | 227 | 8.13E-01 | 8.25E-01 | CSF2RB |
| ARMS-mediated activation | Reactome | R-HSA-170984 | 1 | 230 | 8.17E-01 | 8.29E-01 | CSF2RB |
| Signalling to p38 via RIT and RIN | Reactome | R-HSA-187706 | 1 | 230 | 8.17E-01 | 8.29E-01 | CSF2RB |
| MAPK1/MAPK3 signaling | Reactome | R-HSA-5684996 | 1 | 231 | 8.19E-01 | 8.30E-01 | CSF2RB |
| Frs2-mediated activation | Reactome | R-HSA-170968 | 1 | 231 | 8.19E-01 | 8.30E-01 | CSF2RB |
| Signaling by Leptin | Reactome | R-HSA-2586552 | 1 | 232 | 8.20E-01 | 8.31E-01 | CSF2RB |
| Prolonged ERK activation events | Reactome | R-HSA-169893 | 1 | 233 | 8.21E-01 | 8.32E-01 | CSF2RB |
| Signalling to RAS | Reactome | R-HSA-167044 | 1 | 237 | 8.27E-01 | 8.37E-01 | CSF2RB |
| VEGFR2 mediated cell proliferation | Reactome | R-HSA-5218921 | 1 | 238 | 8.28E-01 | 8.38E-01 | CSF2RB |
| Signalling to ERKs | Reactome | R-HSA-187687 | 1 | 244 | 8.35E-01 | 8.45E-01 | CSF2RB |
| Signalling by NGF | Reactome | R-HSA-166520 | 2 | 441 | 8.37E-01 | 8.46E-01 | PIK3CB|CSF2RB |
| FCERI mediated MAPK activation | Reactome | R-HSA-2871796 | 1 | 249 | 8.41E-01 | 8.50E-01 | CSF2RB |
| NCAM signaling for neurite out-growth | Reactome | R-HSA-375165 | 1 | 262 | 8.56E-01 | 8.63E-01 | CSF2RB |
| MAPK family signaling cascades | Reactome | R-HSA-5683057 | 1 | 269 | 8.63E-01 | 8.69E-01 | CSF2RB |
| Metabolism of carbohydrates | Reactome | R-HSA-71387 | 1 | 281 | 8.75E-01 | 8.80E-01 | GALNS |
| M Phase | Reactome | R-HSA-68886 | 1 | 313 | 9.01E-01 | 9.05E-01 | LEMD3 |
| Infectious disease | Reactome | R-HSA-5663205 | 1 | 348 | 9.24E-01 | 9.27E-01 | GTF2H4 |
| Class I MHC mediated antigen processing & presentation | Reactome | R-HSA-983169 | 1 | 369 | 9.35E-01 | 9.37E-01 | CTSS |
| Gastrin-CREB signalling pathway via PKC and MAPK | Reactome | R-HSA-881907 | 1 | 421 | 9.56E-01 | 9.56E-01 | CSF2RB |
