# Supplementary material for: Identification of potential candidate genes and pathways in atrioventricular nodal reentry tachycardia by whole‐exome sequencing
Source: Clin Transl Med. 2020 Apr 30;10(1):238–57. doi: 10.1002/ctm2.25 (PMC7240861; doi:10.1002/ctm2.25)
Supplement: Supplementary file 6 — Supporting Information S5 [file CTM2-10-238-s010.doc]

**S8: Reactome-kobas pathway enrichment (MAF< 0.001)**

| **Term** | **Data Base** | **ID** | **Input number** | **Background Number** | **P Value** | **Corrected P Value** | **Gene Name** |
| --- | --- | --- | --- | --- | --- | --- | --- |
| Defective C1GALT1C1 causes Tn polyagglutination syndrome (TNPS) | Reactome | R-HSA-5083632 | 3 | 18 | 9.86E-05 | 7.41E-03 | MUC5B|MUC6|MUC2 |
| Defective GALNT12 causes colorectal cancer 1 (CRCS1) | Reactome | R-HSA-5083636 | 3 | 18 | 9.86E-05 | 7.41E-03 | MUC5B|MUC6|MUC2 |
| Defective GALNT3 causes familial hyperphosphatemic tumoral calcinosis (HFTC) | Reactome | R-HSA-5083625 | 3 | 18 | 9.86E-05 | 7.41E-03 | MUC5B|MUC6|MUC2 |
| Termination of O-glycan biosynthesis | Reactome | R-HSA-977068 | 3 | 25 | 2.38E-04 | 1.30E-02 | MUC5B|MUC6|MUC2 |
| Olfactory Signaling Pathway | Reactome | R-HSA-381753 | 7 | 374 | 1.39E-03 | 4.10E-02 | OR5T2|OR6C76|OR11H2|OR2T35|OR2M2|OR8U1|OR2T2 |
| GPCR downstream signaling | Reactome | R-HSA-388396 | 11 | 940 | 2.90E-03 | 5.95E-02 | OR5T2|OR6C76|OR11H2|TAS2R43|OR2T35|TAS2R46|SSTR1|OR2M2|OR8U1|ARHGEF4|OR2T2 |
| O-linked glycosylation of mucins | Reactome | R-HSA-913709 | 3 | 65 | 3.21E-03 | 6.34E-02 | MUC5B|MUC6|MUC2 |
| Diseases associated with O-glycosylation of proteins | Reactome | R-HSA-3906995 | 3 | 67 | 3.48E-03 | 6.56E-02 | MUC5B|MUC6|MUC2 |
| TP53 regulates transcription of additional cell cycle genes whose exact role in the p53 pathway remain uncertain | Reactome | R-HSA-6804115 | 2 | 21 | 4.40E-03 | 7.38E-02 | CNOT1|CNOT2 |
| ECM proteoglycans | Reactome | R-HSA-3000178 | 3 | 74 | 4.55E-03 | 7.46E-02 | COL4A3|TNXB|COL5A1 |
| Initial triggering of complement | Reactome | R-HSA-166663 | 2 | 22 | 4.78E-03 | 7.61E-02 | CFD|C4A |
| Deadenylation of mRNA | Reactome | R-HSA-429947 | 2 | 23 | 5.18E-03 | 7.69E-02 | CNOT1|CNOT2 |
| Signaling by GPCR | Reactome | R-HSA-372790 | 12 | 1248 | 8.70E-03 | 1.00E-01 | OR5T2|OR6C76|OR11H2|PSMB11|TAS2R43|OR2T35|TAS2R46|SSTR1|OR2M2|OR8U1|ARHGEF4|OR2T2 |
| Signaling by Robo receptor | Reactome | R-HSA-376176 | 2 | 32 | 9.45E-03 | 1.04E-01 | ROBO1|EVL |
| O-linked glycosylation | Reactome | R-HSA-5173105 | 3 | 109 | 1.27E-02 | 1.23E-01 | MUC5B|MUC6|MUC2 |
| Class C/3 (Metabotropic glutamate/pheromone receptors) | Reactome | R-HSA-420499 | 2 | 38 | 1.29E-02 | 1.23E-01 | TAS2R46|TAS2R43 |
| Fanconi Anemia Pathway | Reactome | R-HSA-6783310 | 2 | 40 | 1.42E-02 | 1.31E-01 | FAAP20|EME1 |
| NCAM1 interactions | Reactome | R-HSA-419037 | 2 | 42 | 1.55E-02 | 1.38E-01 | COL4A3|COL5A1 |
| Complement cascade | Reactome | R-HSA-166658 | 2 | 44 | 1.69E-02 | 1.47E-01 | CFD|C4A |
| TP53 Regulates Transcription of Cell Cycle Genes | Reactome | R-HSA-6791312 | 2 | 48 | 1.97E-02 | 1.64E-01 | CNOT1|CNOT2 |
| Signal Transduction | Reactome | R-HSA-162582 | 18 | 2448 | 2.01E-02 | 1.66E-01 | COL4A3|OR6C76|OR11H2|PSMB11|TAS2R43|OR2T35|EVC|BCR|SSTR1|EVL|COL5A1|OR2M2|OR5T2|OR8U1|ARHGEF4|OR2T2|TAS2R46|KDM4C |
| Deadenylation-dependent mRNA decay | Reactome | R-HSA-429914 | 2 | 50 | 2.12E-02 | 1.72E-01 | CNOT1|CNOT2 |
| Diseases of glycosylation | Reactome | R-HSA-3781865 | 3 | 136 | 2.25E-02 | 1.77E-01 | MUC5B|MUC6|MUC2 |
| Assembly of collagen fibrils and other multimeric structures | Reactome | R-HSA-2022090 | 2 | 54 | 2.44E-02 | 1.77E-01 | COL4A3|COL5A1 |
| Cardiac conduction | Reactome | R-HSA-5576891 | 3 | 141 | 2.46E-02 | 1.77E-01 | HIPK2|NOS1|ASPH |
| Alternative complement activation | Reactome | R-HSA-173736 | 1 | 5 | 2.56E-02 | 1.77E-01 | CFD |
| Ion homeostasis | Reactome | R-HSA-5578775 | 2 | 56 | 2.60E-02 | 1.79E-01 | NOS1|ASPH |
| Disorders of transmembrane transporters | Reactome | R-HSA-5619115 | 3 | 149 | 2.83E-02 | 1.85E-01 | SLC26A4|PSMB11|SFTPA2 |
| Non-integrin membrane-ECM interactions | Reactome | R-HSA-3000171 | 2 | 59 | 2.86E-02 | 1.85E-01 | COL4A3|COL5A1 |
| Clearance of Nuclear Envelope Membranes from Chromatin | Reactome | R-HSA-2993913 | 1 | 6 | 2.98E-02 | 1.85E-01 | LEMD3 |
| Defective ABCA3 causes pulmonary surfactant metabolism dysfunction type 3 (SMDP3) | Reactome | R-HSA-5683678 | 1 | 6 | 2.98E-02 | 1.85E-01 | SFTPA2 |
| Collagen degradation | Reactome | R-HSA-1442490 | 2 | 64 | 3.30E-02 | 1.91E-01 | COL4A3|COL5A1 |
| Axon guidance | Reactome | R-HSA-422475 | 6 | 549 | 3.37E-02 | 1.91E-01 | COL4A3|EPHB4|PSMB11|COL5A1|EVL|ROBO1 |
| Multifunctional anion exchangers | Reactome | R-HSA-427601 | 1 | 7 | 3.39E-02 | 1.91E-01 | SLC26A4 |
| Collagen biosynthesis and modifying enzymes | Reactome | R-HSA-1650814 | 2 | 67 | 3.58E-02 | 1.94E-01 | COL4A3|COL5A1 |
| Loss of proteins required for interphase microtubule organization聽from the centrosome | Reactome | R-HSA-380284 | 2 | 68 | 3.68E-02 | 1.94E-01 | CEP290|CDK5RAP2 |
| Loss of Nlp from mitotic centrosomes | Reactome | R-HSA-380259 | 2 | 68 | 3.68E-02 | 1.94E-01 | CEP290|CDK5RAP2 |
| Defective CSF2RA causes pulmonary surfactant metabolism dysfunction 4 (SMDP4) | Reactome | R-HSA-5688890 | 1 | 8 | 3.81E-02 | 1.94E-01 | SFTPA2 |
| Defective CSF2RB causes pulmonary surfactant metabolism dysfunction 5 (SMDP5) | Reactome | R-HSA-5688849 | 1 | 8 | 3.81E-02 | 1.94E-01 | SFTPA2 |
| Activation of C3 and C5 | Reactome | R-HSA-174577 | 1 | 8 | 3.81E-02 | 1.94E-01 | C4A |
| Extracellular matrix organization | Reactome | R-HSA-1474244 | 4 | 290 | 3.88E-02 | 1.97E-01 | COL4A3|TNXB|COL5A1|LTBP1 |
| AURKA Activation by TPX2 | Reactome | R-HSA-8854518 | 2 | 71 | 3.97E-02 | 1.99E-01 | CEP290|CDK5RAP2 |
| Role of Abl in Robo-Slit signaling | Reactome | R-HSA-428890 | 1 | 9 | 4.23E-02 | 2.01E-01 | ROBO1 |
| Inactivation of Cdc42 and Rac | Reactome | R-HSA-428543 | 1 | 9 | 4.23E-02 | 2.01E-01 | ROBO1 |
| GP1b-IX-V activation signalling | Reactome | R-HSA-430116 | 1 | 9 | 4.23E-02 | 2.01E-01 | GP1BA |
| Initiation of Nuclear Envelope Reformation | Reactome | R-HSA-2995383 | 1 | 10 | 4.64E-02 | 2.08E-01 | LEMD3 |
| Activation of SMO | Reactome | R-HSA-5635838 | 1 | 10 | 4.64E-02 | 2.08E-01 | EVC |
| Nuclear Envelope Reassembly | Reactome | R-HSA-2995410 | 1 | 10 | 4.64E-02 | 2.08E-01 | LEMD3 |
| G2/M Transition | Reactome | R-HSA-69275 | 3 | 185 | 4.81E-02 | 2.13E-01 | CEP290|CDK5RAP2|PSMB11 |
| Mitotic G2-G2/M phases | Reactome | R-HSA-453274 | 3 | 187 | 4.93E-02 | 2.13E-01 | CEP290|CDK5RAP2|PSMB11 |
| ABC transporter disorders | Reactome | R-HSA-5619084 | 2 | 81 | 5.00E-02 | 2.13E-01 | PSMB11|SFTPA2 |
| DSCAM interactions | Reactome | R-HSA-376172 | 1 | 11 | 5.05E-02 | 2.13E-01 | DSCAML1 |
| Hedgehog 'on' state | Reactome | R-HSA-5632684 | 2 | 83 | 5.22E-02 | 2.19E-01 | EVC|PSMB11 |
| Integrin cell surface interactions | Reactome | R-HSA-216083 | 2 | 85 | 5.44E-02 | 2.20E-01 | COL4A3|COL5A1 |
| Regulation of PLK1 Activity at G2/M Transition | Reactome | R-HSA-2565942 | 2 | 85 | 5.44E-02 | 2.20E-01 | CEP290|CDK5RAP2 |
| Josephin domain DUBs | Reactome | R-HSA-5689877 | 1 | 12 | 5.46E-02 | 2.20E-01 | ATXN3 |
| Diseases associated with surfactant metabolism | Reactome | R-HSA-5687613 | 1 | 12 | 5.46E-02 | 2.20E-01 | SFTPA2 |
| Scavenging of heme from plasma | Reactome | R-HSA-2168880 | 1 | 12 | 5.46E-02 | 2.20E-01 | CD163 |
| Physiological factors | Reactome | R-HSA-5578768 | 1 | 12 | 5.46E-02 | 2.20E-01 | HIPK2 |
| Collagen formation | Reactome | R-HSA-1474290 | 2 | 88 | 5.77E-02 | 2.20E-01 | COL4A3|COL5A1 |
| Depolymerisation of the Nuclear Lamina | Reactome | R-HSA-4419969 | 1 | 13 | 5.87E-02 | 2.20E-01 | LEMD3 |
| Passive transport by Aquaporins | Reactome | R-HSA-432047 | 1 | 13 | 5.87E-02 | 2.20E-01 | AQP7 |
| Muscle contraction | Reactome | R-HSA-397014 | 3 | 204 | 6.06E-02 | 2.25E-01 | HIPK2|NOS1|ASPH |
| Centrosome maturation | Reactome | R-HSA-380287 | 2 | 91 | 6.12E-02 | 2.25E-01 | CEP290|CDK5RAP2 |
| Recruitment of mitotic centrosome proteins and complexes | Reactome | R-HSA-380270 | 2 | 91 | 6.12E-02 | 2.25E-01 | CEP290|CDK5RAP2 |
| Signal regulatory protein (SIRP) family interactions | Reactome | R-HSA-391160 | 1 | 14 | 6.27E-02 | 2.25E-01 | SFTPA2 |
| Ion channel transport | Reactome | R-HSA-983712 | 3 | 211 | 6.56E-02 | 2.29E-01 | SLC9B1|ATP2C2|ASPH |
| Anchoring fibril formation | Reactome | R-HSA-2214320 | 1 | 15 | 6.67E-02 | 2.29E-01 | COL4A3 |
| Signaling by cytosolic FGFR1 fusion mutants | Reactome | R-HSA-1839117 | 1 | 15 | 6.67E-02 | 2.29E-01 | BCR |
| Platelet Adhesion to exposed collagen | Reactome | R-HSA-75892 | 1 | 15 | 6.67E-02 | 2.29E-01 | GP1BA |
| Activation of Rac | Reactome | R-HSA-428540 | 1 | 15 | 6.67E-02 | 2.29E-01 | ROBO1 |
| Anchoring of the basal body to the plasma membrane | Reactome | R-HSA-5620912 | 2 | 96 | 6.70E-02 | 2.29E-01 | CEP290|CDK5RAP2 |
| Transmembrane transport of small molecules | Reactome | R-HSA-382551 | 6 | 657 | 6.80E-02 | 2.31E-01 | SLC26A4|PSMB11|ATP2C2|SLC9B1|AQP7|ASPH |
| Processing of Intronless Pre-mRNAs | Reactome | R-HSA-77595 | 1 | 16 | 7.08E-02 | 2.33E-01 | CPSF7 |
| Post-translational protein modification | Reactome | R-HSA-597592 | 7 | 840 | 7.47E-02 | 2.38E-01 | USP17L11|PSMB11|ATXN3|MUC5B|MUC6|EDEM3|MUC2 |
| Metabolism of proteins | Reactome | R-HSA-392499 | 10 | 1378 | 7.84E-02 | 2.43E-01 | USP17L11|PSMB11|TIMM23|MUC5B|RAB27A|MUC6|EDEM3|SFTPA2|MUC2|ATXN3 |
| Ephrin signaling | Reactome | R-HSA-3928664 | 1 | 18 | 7.88E-02 | 2.43E-01 | EPHB4 |
| NoRC negatively regulates rRNA expression | Reactome | R-HSA-427413 | 2 | 106 | 7.93E-02 | 2.44E-01 | DNMT1|TAF1C |
| Stimuli-sensing channels | Reactome | R-HSA-2672351 | 2 | 108 | 8.18E-02 | 2.48E-01 | SLC9B1|ASPH |
| Regulation of TLR by endogenous ligand | Reactome | R-HSA-5686938 | 1 | 19 | 8.27E-02 | 2.48E-01 | SFTPA2 |
| Negative epigenetic regulation of rRNA expression | Reactome | R-HSA-5250941 | 2 | 109 | 8.31E-02 | 2.48E-01 | DNMT1|TAF1C |
| G alpha (i) signalling events | Reactome | R-HSA-418594 | 3 | 242 | 8.98E-02 | 2.61E-01 | TAS2R46|SSTR1|TAS2R43 |
| SALM protein interactions at the synapses | Reactome | R-HSA-8849932 | 1 | 21 | 9.06E-02 | 2.61E-01 | LRFN4 |
| ER Quality Control Compartment (ERQC) | Reactome | R-HSA-901032 | 1 | 21 | 9.06E-02 | 2.61E-01 | EDEM3 |
| Intrinsic Pathway of Fibrin Clot Formation | Reactome | R-HSA-140837 | 1 | 22 | 9.45E-02 | 2.64E-01 | GP1BA |
| TCR signaling | Reactome | R-HSA-202403 | 2 | 119 | 9.61E-02 | 2.67E-01 | PSMB11|EVL |
| Nitric oxide stimulates guanylate cyclase | Reactome | R-HSA-392154 | 1 | 23 | 9.84E-02 | 2.69E-01 | NOS1 |
| Disease | Reactome | R-HSA-1643685 | 7 | 902 | 9.92E-02 | 2.70E-01 | SLC26A4|PSMB11|MUC5B|BCR|MUC6|SFTPA2|MUC2 |
| Rho GTPase cycle | Reactome | R-HSA-194840 | 2 | 122 | 1.00E-01 | 2.72E-01 | BCR|ARHGEF4 |
| Processing of Capped Intronless Pre-mRNA | Reactome | R-HSA-75067 | 1 | 25 | 1.06E-01 | 2.79E-01 | CPSF7 |
| FGFR1 mutant receptor activation | Reactome | R-HSA-1839124 | 1 | 25 | 1.06E-01 | 2.79E-01 | BCR |
| NCAM signaling for neurite out-growth | Reactome | R-HSA-375165 | 3 | 262 | 1.07E-01 | 2.79E-01 | COL4A3|PSMB11|COL5A1 |
| Signaling by Rho GTPases | Reactome | R-HSA-194315 | 4 | 416 | 1.09E-01 | 2.82E-01 | KDM4C|BCR|ARHGEF4|EVL |
| Calnexin/calreticulin cycle | Reactome | R-HSA-901042 | 1 | 26 | 1.10E-01 | 2.82E-01 | EDEM3 |
| Insulin processing | Reactome | R-HSA-264876 | 1 | 26 | 1.10E-01 | 2.82E-01 | RAB27A |
| Syndecan interactions | Reactome | R-HSA-3000170 | 1 | 27 | 1.14E-01 | 2.85E-01 | COL5A1 |
| Cell-Cell communication | Reactome | R-HSA-1500931 | 2 | 133 | 1.15E-01 | 2.88E-01 | DSCAML1|SFTPA2 |
| Regulation of Complement cascade | Reactome | R-HSA-977606 | 1 | 28 | 1.18E-01 | 2.90E-01 | C4A |
| Degradation of the extracellular matrix | Reactome | R-HSA-1474228 | 2 | 135 | 1.18E-01 | 2.90E-01 | COL4A3|COL5A1 |
| YAP1- and WWTR1 (TAZ)-stimulated gene expression | Reactome | R-HSA-2032785 | 1 | 29 | 1.22E-01 | 2.94E-01 | HIPK2 |
| Hemostasis | Reactome | R-HSA-109582 | 5 | 605 | 1.25E-01 | 2.97E-01 | DOCK5|CFD|GP1BA|NOS1|DOCK11 |
| RNA Polymerase I Promoter Escape | Reactome | R-HSA-73772 | 1 | 30 | 1.25E-01 | 2.97E-01 | TAF1C |
| Surfactant metabolism | Reactome | R-HSA-5683826 | 1 | 30 | 1.25E-01 | 2.97E-01 | SFTPA2 |
| Laminin interactions | Reactome | R-HSA-3000157 | 1 | 30 | 1.25E-01 | 2.97E-01 | COL4A3 |
| RNA Polymerase I Transcription Termination | Reactome | R-HSA-73863 | 1 | 31 | 1.29E-01 | 3.02E-01 | TAF1C |
| Detoxification of Reactive Oxygen Species | Reactome | R-HSA-3299685 | 1 | 32 | 1.33E-01 | 3.05E-01 | CCS |
| Signaling by FGFR1 in disease | Reactome | R-HSA-5655302 | 1 | 32 | 1.33E-01 | 3.05E-01 | BCR |
| @@Signaling by FGFR1 | Reactome | R-HSA-5654736 | 1 | 325 | 7.57E-01 | 7.88E-01 | PSMB11 |
| Epigenetic regulation of gene expression | Reactome | R-HSA-212165 | 2 | 146 | 1.34E-01 | 3.06E-01 | DNMT1|TAF1C |
| Resolution of D-loop Structures through Holliday Junction Intermediates | Reactome | R-HSA-5693568 | 1 | 33 | 1.37E-01 | 3.06E-01 | EME1 |
| Generation of second messenger molecules | Reactome | R-HSA-202433 | 1 | 33 | 1.37E-01 | 3.06E-01 | EVL |
| Signaling by Hedgehog | Reactome | R-HSA-5358351 | 2 | 148 | 1.37E-01 | 3.06E-01 | EVC|PSMB11 |
| Deubiquitination | Reactome | R-HSA-5688426 | 3 | 294 | 1.37E-01 | 3.06E-01 | USP17L11|PSMB11|ATXN3 |
| ROS, RNS production in response to bacteria | Reactome | R-HSA-1222556 | 1 | 34 | 1.40E-01 | 3.10E-01 | NOS1 |
| Resolution of D-Loop Structures | Reactome | R-HSA-5693537 | 1 | 34 | 1.40E-01 | 3.10E-01 | EME1 |
| N-glycan trimming in the ER and Calnexin/Calreticulin cycle | Reactome | R-HSA-532668 | 1 | 35 | 1.44E-01 | 3.13E-01 | EDEM3 |
| Platelet Aggregation (Plug Formation) | Reactome | R-HSA-76009 | 1 | 35 | 1.44E-01 | 3.13E-01 | GP1BA |
| Antigen processing: Ubiquitination & Proteasome degradation | Reactome | R-HSA-983168 | 3 | 307 | 1.50E-01 | 3.19E-01 | LMO7|ASB10|PSMB11 |
| Molecules associated with elastic fibres | Reactome | R-HSA-2129379 | 1 | 38 | 1.55E-01 | 3.23E-01 | LTBP1 |
| Formation of Fibrin Clot (Clotting Cascade) | Reactome | R-HSA-140877 | 1 | 39 | 1.59E-01 | 3.26E-01 | GP1BA |
| Binding and Uptake of Ligands by Scavenger Receptors | Reactome | R-HSA-2173782 | 1 | 39 | 1.59E-01 | 3.26E-01 | CD163 |
| Developmental Biology | Reactome | R-HSA-1266738 | 6 | 841 | 1.60E-01 | 3.29E-01 | COL4A3|EPHB4|PSMB11|COL5A1|EVL|ROBO1 |
| EPHB-mediated forward signaling | Reactome | R-HSA-3928662 | 1 | 41 | 1.66E-01 | 3.35E-01 | EPHB4 |
| Netrin-1 signaling | Reactome | R-HSA-373752 | 1 | 41 | 1.66E-01 | 3.35E-01 | ROBO1 |
| Factors involved in megakaryocyte development and platelet production | Reactome | R-HSA-983231 | 2 | 171 | 1.71E-01 | 3.40E-01 | DOCK5|DOCK11 |
| Cell Cycle, Mitotic | Reactome | R-HSA-69278 | 4 | 503 | 1.76E-01 | 3.44E-01 | CEP290|CDK5RAP2|PSMB11|LEMD3 |
| Nuclear Envelope Breakdown | Reactome | R-HSA-2980766 | 1 | 45 | 1.80E-01 | 3.48E-01 | LEMD3 |
| Elastic fibre formation | Reactome | R-HSA-1566948 | 1 | 45 | 1.80E-01 | 3.48E-01 | LTBP1 |
| RNA Polymerase I Transcription Initiation | Reactome | R-HSA-73762 | 1 | 47 | 1.87E-01 | 3.55E-01 | TAF1C |
| NRAGE signals death through JNK | Reactome | R-HSA-193648 | 1 | 47 | 1.87E-01 | 3.55E-01 | ARHGEF4 |
| EPH-ephrin mediated repulsion of cells | Reactome | R-HSA-3928665 | 1 | 49 | 1.94E-01 | 3.63E-01 | EPHB4 |
| Mitotic Anaphase | Reactome | R-HSA-68882 | 2 | 188 | 1.98E-01 | 3.65E-01 | LEMD3|PSMB11 |
| Cross-presentation of soluble exogenous antigens (endosomes) | Reactome | R-HSA-1236978 | 1 | 50 | 1.98E-01 | 3.65E-01 | PSMB11 |
| HDMs demethylate histones | Reactome | R-HSA-3214842 | 1 | 50 | 1.98E-01 | 3.65E-01 | KDM4C |
| Regulation of activated PAK-2p34 by proteasome mediated degradation | Reactome | R-HSA-211733 | 1 | 50 | 1.98E-01 | 3.65E-01 | PSMB11 |
| Mitotic Metaphase and Anaphase | Reactome | R-HSA-2555396 | 2 | 189 | 1.99E-01 | 3.66E-01 | LEMD3|PSMB11 |
| Transcriptional Regulation by TP53 | Reactome | R-HSA-3700989 | 3 | 355 | 2.01E-01 | 3.67E-01 | CNOT1|HIPK2|CNOT2 |
| Ubiquitin-dependent degradation of Cyclin D | Reactome | R-HSA-75815 | 1 | 51 | 2.01E-01 | 3.67E-01 | PSMB11 |
| Regulation of ornithine decarboxylase (ODC) | Reactome | R-HSA-350562 | 1 | 51 | 2.01E-01 | 3.67E-01 | PSMB11 |
| Regulation of Apoptosis | Reactome | R-HSA-169911 | 1 | 51 | 2.01E-01 | 3.67E-01 | PSMB11 |
| CDK-mediated phosphorylation and removal of Cdc6 | Reactome | R-HSA-69017 | 1 | 51 | 2.01E-01 | 3.67E-01 | PSMB11 |
| Ubiquitin-dependent degradation of Cyclin D1 | Reactome | R-HSA-69229 | 1 | 51 | 2.01E-01 | 3.67E-01 | PSMB11 |
| Signaling by PDGF | Reactome | R-HSA-186797 | 3 | 356 | 2.02E-01 | 3.69E-01 | COL4A3|PSMB11|COL5A1 |
| Vpu mediated degradation of CD4 | Reactome | R-HSA-180534 | 1 | 52 | 2.05E-01 | 3.69E-01 | PSMB11 |
| Aquaporin-mediated transport | Reactome | R-HSA-445717 | 1 | 52 | 2.05E-01 | 3.69E-01 | AQP7 |
| Autodegradation of the E3 ubiquitin ligase COP1 | Reactome | R-HSA-349425 | 1 | 52 | 2.05E-01 | 3.69E-01 | PSMB11 |
| p53-Independent DNA Damage Response | Reactome | R-HSA-69610 | 1 | 53 | 2.08E-01 | 3.70E-01 | PSMB11 |
| Vif-mediated degradation of APOBEC3G | Reactome | R-HSA-180585 | 1 | 53 | 2.08E-01 | 3.70E-01 | PSMB11 |
| Ubiquitin Mediated Degradation of Phosphorylated Cdc25A | Reactome | R-HSA-69601 | 1 | 53 | 2.08E-01 | 3.70E-01 | PSMB11 |
| p53-Independent G1/S DNA damage checkpoint | Reactome | R-HSA-69613 | 1 | 53 | 2.08E-01 | 3.70E-01 | PSMB11 |
| Mitochondrial protein import | Reactome | R-HSA-1268020 | 1 | 54 | 2.11E-01 | 3.72E-01 | TIMM23 |
| Assembly of the primary cilium | Reactome | R-HSA-5617833 | 2 | 199 | 2.15E-01 | 3.72E-01 | CEP290|CDK5RAP2 |
| mRNA 3'-end processing | Reactome | R-HSA-72187 | 1 | 55 | 2.15E-01 | 3.72E-01 | CPSF7 |
| SCF-beta-TrCP mediated degradation of Emi1 | Reactome | R-HSA-174113 | 1 | 55 | 2.15E-01 | 3.72E-01 | PSMB11 |
| Degradation of AXIN | Reactome | R-HSA-4641257 | 1 | 55 | 2.15E-01 | 3.72E-01 | PSMB11 |
| AUF1 (hnRNP D0) binds and destabilizes mRNA | Reactome | R-HSA-450408 | 1 | 55 | 2.15E-01 | 3.72E-01 | PSMB11 |
| Stabilization of p53 | Reactome | R-HSA-69541 | 1 | 55 | 2.15E-01 | 3.72E-01 | PSMB11 |
| Class I MHC mediated antigen processing & presentation | Reactome | R-HSA-983169 | 3 | 369 | 2.17E-01 | 3.75E-01 | LMO7|ASB10|PSMB11 |
| Hh mutants that don't undergo autocatalytic processing are degraded by ERAD | Reactome | R-HSA-5362768 | 1 | 56 | 2.18E-01 | 3.77E-01 | PSMB11 |
| Signaling by FGFR in disease | Reactome | R-HSA-1226099 | 1 | 57 | 2.22E-01 | 3.79E-01 | BCR |
| @@Signaling by FGFR | Reactome | R-HSA-190236 | 1 | 355 | 7.86E-01 | 8.12E-01 | PSMB11 |
| Degradation of DVL | Reactome | R-HSA-4641258 | 1 | 57 | 2.22E-01 | 3.79E-01 | PSMB11 |
| Ion transport by P-type ATPases | Reactome | R-HSA-936837 | 1 | 57 | 2.22E-01 | 3.79E-01 | ATP2C2 |
| Hh mutants abrogate ligand secretion | Reactome | R-HSA-5387390 | 1 | 59 | 2.28E-01 | 3.87E-01 | PSMB11 |
| CDT1 association with the CDC6:ORC:origin complex | Reactome | R-HSA-68827 | 1 | 59 | 2.28E-01 | 3.87E-01 | PSMB11 |
| NIK-->noncanonical NF-kB signaling | Reactome | R-HSA-5676590 | 1 | 59 | 2.28E-01 | 3.87E-01 | PSMB11 |
| Degradation of GLI2 by the proteasome | Reactome | R-HSA-5610783 | 1 | 60 | 2.32E-01 | 3.89E-01 | PSMB11 |
| GLI3 is processed to GLI3R by the proteasome | Reactome | R-HSA-5610785 | 1 | 60 | 2.32E-01 | 3.89E-01 | PSMB11 |
| Dectin-1 mediated noncanonical NF-kB signaling | Reactome | R-HSA-5607761 | 1 | 60 | 2.32E-01 | 3.89E-01 | PSMB11 |
| Degradation of GLI1 by the proteasome | Reactome | R-HSA-5610780 | 1 | 60 | 2.32E-01 | 3.89E-01 | PSMB11 |
| Defective CFTR causes cystic fibrosis | Reactome | R-HSA-5678895 | 1 | 61 | 2.35E-01 | 3.93E-01 | PSMB11 |
| Cell death signalling via NRAGE, NRIF and NADE | Reactome | R-HSA-204998 | 1 | 63 | 2.42E-01 | 3.98E-01 | ARHGEF4 |
| Innate Immune System | Reactome | R-HSA-168249 | 5 | 769 | 2.42E-01 | 3.98E-01 | CFD|PSMB11|KLRC2|C4A|SFTPA2 |
| Cleavage of Growing Transcript in the Termination Region | Reactome | R-HSA-109688 | 1 | 64 | 2.45E-01 | 4.00E-01 | CPSF7 |
| RNA Polymerase II Transcription Termination | Reactome | R-HSA-73856 | 1 | 64 | 2.45E-01 | 4.00E-01 | CPSF7 |
| p53-Dependent G1/S DNA damage checkpoint | Reactome | R-HSA-69580 | 1 | 64 | 2.45E-01 | 4.00E-01 | PSMB11 |
| Asymmetric localization of PCP proteins | Reactome | R-HSA-4608870 | 1 | 64 | 2.45E-01 | 4.00E-01 | PSMB11 |
| p53-Dependent G1 DNA Damage Response | Reactome | R-HSA-69563 | 1 | 64 | 2.45E-01 | 4.00E-01 | PSMB11 |
| Ub-specific processing proteases | Reactome | R-HSA-5689880 | 2 | 220 | 2.48E-01 | 4.03E-01 | USP17L11|PSMB11 |
| Hedgehog ligand biogenesis | Reactome | R-HSA-5358346 | 1 | 65 | 2.48E-01 | 4.03E-01 | PSMB11 |
| DNA methylation | Reactome | R-HSA-5334118 | 1 | 65 | 2.48E-01 | 4.03E-01 | DNMT1 |
| Immune System | Reactome | R-HSA-168256 | 9 | 1583 | 2.49E-01 | 4.04E-01 | LMO7|PSMB11|KLRC2|C4A|EVL|CFD|ASB10|SFTPA2|NOS1 |
| G1/S DNA Damage Checkpoints | Reactome | R-HSA-69615 | 1 | 66 | 2.51E-01 | 4.05E-01 | PSMB11 |
| HDR through Homologous Recombination (HRR) | Reactome | R-HSA-5685942 | 1 | 67 | 2.55E-01 | 4.08E-01 | EME1 |
| APC/C:Cdc20 mediated degradation of Securin | Reactome | R-HSA-174154 | 1 | 67 | 2.55E-01 | 4.08E-01 | PSMB11 |
| Activation of NF-kappaB in B cells | Reactome | R-HSA-1169091 | 1 | 67 | 2.55E-01 | 4.08E-01 | PSMB11 |
| Activated PKN1 stimulates transcription of AR (androgen receptor) regulated genes KLK2 and KLK3 | Reactome | R-HSA-5625886 | 1 | 67 | 2.55E-01 | 4.08E-01 | KDM4C |
| Regulation of RAS by GAPs | Reactome | R-HSA-5658442 | 1 | 68 | 2.58E-01 | 4.09E-01 | PSMB11 |
| SLC transporter disorders | Reactome | R-HSA-5619102 | 1 | 68 | 2.58E-01 | 4.09E-01 | SLC26A4 |
| SIRT1 negatively regulates rRNA Expression | Reactome | R-HSA-427359 | 1 | 68 | 2.58E-01 | 4.09E-01 | TAF1C |
| Assembly of the pre-replicative complex | Reactome | R-HSA-68867 | 1 | 68 | 2.58E-01 | 4.09E-01 | PSMB11 |
| RNA polymerase II transcribes snRNA genes | Reactome | R-HSA-6807505 | 1 | 70 | 2.64E-01 | 4.13E-01 | INTS1 |
| Orc1 removal from chromatin | Reactome | R-HSA-68949 | 1 | 71 | 2.67E-01 | 4.17E-01 | PSMB11 |
| Switching of origins to a post-replicative state | Reactome | R-HSA-69052 | 1 | 71 | 2.67E-01 | 4.17E-01 | PSMB11 |
| Cell Cycle | Reactome | R-HSA-1640170 | 4 | 607 | 2.70E-01 | 4.19E-01 | CEP290|CDK5RAP2|PSMB11|LEMD3 |
| Cdc20:Phospho-APC/C mediated degradation of Cyclin A | Reactome | R-HSA-174184 | 1 | 72 | 2.71E-01 | 4.19E-01 | PSMB11 |
| APC/C:Cdh1 mediated degradation of Cdc20 and other APC/C:Cdh1 targeted proteins in late mitosis/early G1 | Reactome | R-HSA-174178 | 1 | 72 | 2.71E-01 | 4.19E-01 | PSMB11 |
| Removal of licensing factors from origins | Reactome | R-HSA-69300 | 1 | 73 | 2.74E-01 | 4.22E-01 | PSMB11 |
| PRC2 methylates histones and DNA | Reactome | R-HSA-212300 | 1 | 73 | 2.74E-01 | 4.22E-01 | DNMT1 |
| APC:Cdc20 mediated degradation of cell cycle proteins prior to satisfation of the cell cycle checkpoint | Reactome | R-HSA-179419 | 1 | 73 | 2.74E-01 | 4.22E-01 | PSMB11 |
| APC/C:Cdc20 mediated degradation of mitotic proteins | Reactome | R-HSA-176409 | 1 | 75 | 2.80E-01 | 4.26E-01 | PSMB11 |
| Regulation of DNA replication | Reactome | R-HSA-69304 | 1 | 76 | 2.83E-01 | 4.27E-01 | PSMB11 |
| Activation of APC/C and APC/C:Cdc20 mediated degradation of mitotic proteins | Reactome | R-HSA-176814 | 1 | 76 | 2.83E-01 | 4.27E-01 | PSMB11 |
| G alpha (12/13) signalling events | Reactome | R-HSA-416482 | 1 | 77 | 2.86E-01 | 4.30E-01 | ARHGEF4 |
| The role of GTSE1 in G2/M progression after G2 checkpoint | Reactome | R-HSA-8852276 | 1 | 77 | 2.86E-01 | 4.30E-01 | PSMB11 |
| Regulation of APC/C activators between G1/S and early anaphase | Reactome | R-HSA-176408 | 1 | 80 | 2.95E-01 | 4.39E-01 | PSMB11 |
| Peptide hormone metabolism | Reactome | R-HSA-2980736 | 1 | 81 | 2.98E-01 | 4.42E-01 | RAB27A |
| FCERI mediated NF-kB activation | Reactome | R-HSA-2871837 | 1 | 82 | 3.01E-01 | 4.45E-01 | PSMB11 |
| Degradation of beta-catenin by the destruction complex | Reactome | R-HSA-195253 | 1 | 82 | 3.01E-01 | 4.45E-01 | PSMB11 |
| ER-Phagosome pathway | Reactome | R-HSA-1236974 | 1 | 83 | 3.04E-01 | 4.48E-01 | PSMB11 |
| DNA Replication Pre-Initiation | Reactome | R-HSA-69002 | 1 | 85 | 3.10E-01 | 4.52E-01 | PSMB11 |
| M/G1 Transition | Reactome | R-HSA-68874 | 1 | 85 | 3.10E-01 | 4.52E-01 | PSMB11 |
| Metabolism of polyamines | Reactome | R-HSA-351202 | 1 | 85 | 3.10E-01 | 4.52E-01 | PSMB11 |
| p75 NTR receptor-mediated signalling | Reactome | R-HSA-193704 | 1 | 85 | 3.10E-01 | 4.52E-01 | ARHGEF4 |
| GPCR ligand binding | Reactome | R-HSA-500792 | 3 | 451 | 3.11E-01 | 4.53E-01 | TAS2R46|SSTR1|TAS2R43 |
| APC/C-mediated degradation of cell cycle proteins | Reactome | R-HSA-174143 | 1 | 86 | 3.13E-01 | 4.54E-01 | PSMB11 |
| Regulation of mitotic cell cycle | Reactome | R-HSA-453276 | 1 | 86 | 3.13E-01 | 4.54E-01 | PSMB11 |
| Regulation of mRNA stability by proteins that bind AU-rich elements | Reactome | R-HSA-450531 | 1 | 87 | 3.16E-01 | 4.55E-01 | PSMB11 |
| MAPK6/MAPK4 signaling | Reactome | R-HSA-5687128 | 1 | 88 | 3.19E-01 | 4.58E-01 | PSMB11 |
| Platelet homeostasis | Reactome | R-HSA-418346 | 1 | 88 | 3.19E-01 | 4.58E-01 | NOS1 |
| RNA Polymerase I Chain Elongation | Reactome | R-HSA-73777 | 1 | 90 | 3.25E-01 | 4.62E-01 | TAF1C |
| B-WICH complex positively regulates rRNA expression | Reactome | R-HSA-5250924 | 1 | 90 | 3.25E-01 | 4.62E-01 | TAF1C |
| Regulation of TP53 Activity through Phosphorylation | Reactome | R-HSA-6804756 | 1 | 91 | 3.28E-01 | 4.65E-01 | HIPK2 |
| Gene Expression | Reactome | R-HSA-74160 | 9 | 1719 | 3.28E-01 | 4.65E-01 | PSMB11|CPSF7|DNMT1|ZNF729|HIPK2|CNOT2|TAF1C|CNOT1|INTS1 |
| PCP/CE pathway | Reactome | R-HSA-4086400 | 1 | 92 | 3.31E-01 | 4.67E-01 | PSMB11 |
| RHO GTPases activate PKNs | Reactome | R-HSA-5625740 | 1 | 93 | 3.34E-01 | 4.70E-01 | KDM4C |
| EPH-Ephrin signaling | Reactome | R-HSA-2682334 | 1 | 93 | 3.34E-01 | 4.70E-01 | EPHB4 |
| Platelet activation, signaling and aggregation | Reactome | R-HSA-76002 | 2 | 277 | 3.38E-01 | 4.74E-01 | CFD|GP1BA |
| Toll Like Receptor 2 (TLR2) Cascade | Reactome | R-HSA-181438 | 1 | 95 | 3.40E-01 | 4.75E-01 | SFTPA2 |
| Toll Like Receptor TLR1:TLR2 Cascade | Reactome | R-HSA-168179 | 1 | 95 | 3.40E-01 | 4.75E-01 | SFTPA2 |
| TNFR2 non-canonical NF-kB pathway | Reactome | R-HSA-5668541 | 1 | 97 | 3.45E-01 | 4.81E-01 | PSMB11 |
| Diseases of signal transduction | Reactome | R-HSA-5663202 | 2 | 282 | 3.46E-01 | 4.82E-01 | BCR|PSMB11 |
| Downstream TCR signaling | Reactome | R-HSA-202424 | 1 | 98 | 3.48E-01 | 4.83E-01 | PSMB11 |
| Antigen processing-Cross presentation | Reactome | R-HSA-1236975 | 1 | 99 | 3.51E-01 | 4.85E-01 | PSMB11 |
| Synthesis of DNA | Reactome | R-HSA-69239 | 1 | 100 | 3.54E-01 | 4.86E-01 | PSMB11 |
| Transport of inorganic cations/anions and amino acids/oligopeptides | Reactome | R-HSA-425393 | 1 | 100 | 3.54E-01 | 4.86E-01 | SLC26A4 |
| CLEC7A (Dectin-1) signaling | Reactome | R-HSA-5607764 | 1 | 100 | 3.54E-01 | 4.86E-01 | PSMB11 |
| UCH proteinases | Reactome | R-HSA-5689603 | 1 | 102 | 3.59E-01 | 4.89E-01 | PSMB11 |
| Positive epigenetic regulation of rRNA expression | Reactome | R-HSA-5250913 | 1 | 105 | 3.68E-01 | 4.94E-01 | TAF1C |
| Diseases of metabolism | Reactome | R-HSA-5668914 | 1 | 106 | 3.70E-01 | 4.96E-01 | SFTPA2 |
| ABC-family proteins mediated transport | Reactome | R-HSA-382556 | 1 | 106 | 3.70E-01 | 4.96E-01 | PSMB11 |
| DNA Replication | Reactome | R-HSA-69306 | 1 | 108 | 3.76E-01 | 4.99E-01 | PSMB11 |
| RNA Polymerase I Promoter Clearance | Reactome | R-HSA-73854 | 1 | 109 | 3.78E-01 | 5.01E-01 | TAF1C |
| RHO GTPase Effectors | Reactome | R-HSA-195258 | 2 | 304 | 3.80E-01 | 5.02E-01 | KDM4C|EVL |
| RNA Polymerase I Transcription | Reactome | R-HSA-73864 | 1 | 111 | 3.84E-01 | 5.05E-01 | TAF1C |
| Hedgehog 'off' state | Reactome | R-HSA-5610787 | 1 | 113 | 3.89E-01 | 5.09E-01 | PSMB11 |
| DNA Repair | Reactome | R-HSA-73894 | 2 | 311 | 3.91E-01 | 5.11E-01 | FAAP20|EME1 |
| M Phase | Reactome | R-HSA-68886 | 2 | 313 | 3.94E-01 | 5.13E-01 | LEMD3|PSMB11 |
| Host Interactions of HIV factors | Reactome | R-HSA-162909 | 1 | 123 | 4.15E-01 | 5.29E-01 | PSMB11 |
| C-type lectin receptors (CLRs) | Reactome | R-HSA-5621481 | 1 | 124 | 4.17E-01 | 5.31E-01 | PSMB11 |
| Toll Like Receptor 4 (TLR4) Cascade | Reactome | R-HSA-166016 | 1 | 125 | 4.20E-01 | 5.33E-01 | SFTPA2 |
| DAP12 signaling | Reactome | R-HSA-2424491 | 2 | 333 | 4.24E-01 | 5.37E-01 | PSMB11|KLRC2 |
| S Phase | Reactome | R-HSA-69242 | 1 | 128 | 4.27E-01 | 5.40E-01 | PSMB11 |
| Platelet degranulation | Reactome | R-HSA-114608 | 1 | 128 | 4.27E-01 | 5.40E-01 | CFD |
| RHO GTPases Activate Formins | Reactome | R-HSA-5663220 | 1 | 129 | 4.30E-01 | 5.42E-01 | EVL |
| Organelle biogenesis and maintenance | Reactome | R-HSA-1852241 | 2 | 339 | 4.33E-01 | 5.44E-01 | CEP290|CDK5RAP2 |
| HDR through Homologous Recombination (HR) or Single Strand Annealing (SSA) | Reactome | R-HSA-5693567 | 1 | 132 | 4.37E-01 | 5.48E-01 | EME1 |
| Mitotic Prophase | Reactome | R-HSA-68875 | 1 | 132 | 4.37E-01 | 5.48E-01 | LEMD3 |
| Response to elevated platelet cytosolic Ca2+ | Reactome | R-HSA-76005 | 1 | 133 | 4.40E-01 | 5.49E-01 | CFD |
| DAP12 interactions | Reactome | R-HSA-2172127 | 2 | 345 | 4.41E-01 | 5.51E-01 | PSMB11|KLRC2 |
| Adaptive Immune System | Reactome | R-HSA-1280218 | 4 | 787 | 4.44E-01 | 5.51E-01 | LMO7|ASB10|PSMB11|EVL |
| Homology Directed Repair | Reactome | R-HSA-5693538 | 1 | 138 | 4.52E-01 | 5.57E-01 | EME1 |
| RNA Polymerase II Transcription | Reactome | R-HSA-73857 | 1 | 143 | 4.64E-01 | 5.66E-01 | CPSF7 |
| Beta-catenin independent WNT signaling | Reactome | R-HSA-3858494 | 1 | 145 | 4.68E-01 | 5.69E-01 | PSMB11 |
| RNA Polymerase I, RNA Polymerase III, and Mitochondrial Transcription | Reactome | R-HSA-504046 | 1 | 149 | 4.77E-01 | 5.77E-01 | TAF1C |
| Generic Transcription Pathway | Reactome | R-HSA-212436 | 4 | 822 | 4.77E-01 | 5.77E-01 | CNOT1|ZNF729|HIPK2|CNOT2 |
| Toll-Like Receptors Cascades | Reactome | R-HSA-168898 | 1 | 150 | 4.80E-01 | 5.79E-01 | SFTPA2 |
| Regulation of TP53 Activity | Reactome | R-HSA-5633007 | 1 | 152 | 4.84E-01 | 5.81E-01 | HIPK2 |
| Apoptosis | Reactome | R-HSA-109581 | 1 | 161 | 5.04E-01 | 5.95E-01 | PSMB11 |
| Programmed Cell Death | Reactome | R-HSA-5357801 | 1 | 164 | 5.10E-01 | 6.01E-01 | PSMB11 |
| DNA Double-Strand Break Repair | Reactome | R-HSA-5693532 | 1 | 166 | 5.14E-01 | 6.04E-01 | EME1 |
| G2/M Checkpoints | Reactome | R-HSA-69481 | 1 | 168 | 5.19E-01 | 6.07E-01 | PSMB11 |
| mRNA Splicing - Major Pathway | Reactome | R-HSA-72163 | 1 | 177 | 5.37E-01 | 6.21E-01 | CPSF7 |
| Downstream signaling events of B Cell Receptor (BCR) | Reactome | R-HSA-1168372 | 1 | 177 | 5.37E-01 | 6.21E-01 | PSMB11 |
| Separation of Sister Chromatids | Reactome | R-HSA-2467813 | 1 | 180 | 5.43E-01 | 6.25E-01 | PSMB11 |
| mRNA Splicing | Reactome | R-HSA-72172 | 1 | 185 | 5.53E-01 | 6.31E-01 | CPSF7 |
| Signalling by NGF | Reactome | R-HSA-166520 | 2 | 441 | 5.71E-01 | 6.47E-01 | ARHGEF4|PSMB11 |
| Peptide ligand-binding receptors | Reactome | R-HSA-375276 | 1 | 196 | 5.74E-01 | 6.50E-01 | SSTR1 |
| Cell Cycle Checkpoints | Reactome | R-HSA-69620 | 1 | 200 | 5.81E-01 | 6.56E-01 | PSMB11 |
| Signaling by the B Cell Receptor (BCR) | Reactome | R-HSA-983705 | 1 | 207 | 5.94E-01 | 6.66E-01 | PSMB11 |
| HIV Infection | Reactome | R-HSA-162906 | 1 | 224 | 6.22E-01 | 6.89E-01 | PSMB11 |
| SHC1 events in EGFR signaling | Reactome | R-HSA-180336 | 1 | 226 | 6.26E-01 | 6.91E-01 | PSMB11 |
| SOS-mediated signalling | Reactome | R-HSA-112412 | 1 | 226 | 6.26E-01 | 6.91E-01 | PSMB11 |
| GRB2 events in EGFR signaling | Reactome | R-HSA-179812 | 1 | 226 | 6.26E-01 | 6.91E-01 | PSMB11 |
| SHC1 events in ERBB4 signaling | Reactome | R-HSA-1250347 | 1 | 226 | 6.26E-01 | 6.91E-01 | PSMB11 |
| RAF/MAP kinase cascade | Reactome | R-HSA-5673001 | 1 | 226 | 6.26E-01 | 6.91E-01 | PSMB11 |
| FRS-mediated FGFR1 signaling | Reactome | R-HSA-5654693 | 1 | 227 | 6.27E-01 | 6.92E-01 | PSMB11 |
| FRS-mediated FGFR3 signaling | Reactome | R-HSA-5654706 | 1 | 227 | 6.27E-01 | 6.92E-01 | PSMB11 |
| FRS-mediated FGFR4 signaling | Reactome | R-HSA-5654712 | 1 | 227 | 6.27E-01 | 6.92E-01 | PSMB11 |
| FRS-mediated FGFR2 signaling | Reactome | R-HSA-5654700 | 1 | 227 | 6.27E-01 | 6.92E-01 | PSMB11 |
| Signalling to p38 via RIT and RIN | Reactome | R-HSA-187706 | 1 | 230 | 6.32E-01 | 6.96E-01 | PSMB11 |
| ARMS-mediated activation | Reactome | R-HSA-170984 | 1 | 230 | 6.32E-01 | 6.96E-01 | PSMB11 |
| Processing of Capped Intron-Containing Pre-mRNA | Reactome | R-HSA-72203 | 1 | 231 | 6.34E-01 | 6.97E-01 | CPSF7 |
| Frs2-mediated activation | Reactome | R-HSA-170968 | 1 | 231 | 6.34E-01 | 6.97E-01 | PSMB11 |
| MAPK1/MAPK3 signaling | Reactome | R-HSA-5684996 | 1 | 231 | 6.34E-01 | 6.97E-01 | PSMB11 |
| TCF dependent signaling in response to WNT | Reactome | R-HSA-201681 | 1 | 232 | 6.35E-01 | 6.98E-01 | PSMB11 |
| Signaling by Leptin | Reactome | R-HSA-2586552 | 1 | 232 | 6.35E-01 | 6.98E-01 | PSMB11 |
| Prolonged ERK activation events | Reactome | R-HSA-169893 | 1 | 233 | 6.37E-01 | 6.99E-01 | PSMB11 |
| Interleukin receptor SHC signaling | Reactome | R-HSA-912526 | 1 | 236 | 6.42E-01 | 7.03E-01 | PSMB11 |
| Signalling to RAS | Reactome | R-HSA-167044 | 1 | 237 | 6.43E-01 | 7.04E-01 | PSMB11 |
| VEGFR2 mediated cell proliferation | Reactome | R-HSA-5218921 | 1 | 238 | 6.45E-01 | 7.05E-01 | PSMB11 |
| Interleukin-2 signaling | Reactome | R-HSA-451927 | 1 | 243 | 6.52E-01 | 7.10E-01 | PSMB11 |
| Signalling to ERKs | Reactome | R-HSA-187687 | 1 | 244 | 6.54E-01 | 7.12E-01 | PSMB11 |
| FCERI mediated MAPK activation | Reactome | R-HSA-2871796 | 1 | 249 | 6.61E-01 | 7.18E-01 | PSMB11 |
| RET signaling | Reactome | R-HSA-8853659 | 1 | 251 | 6.64E-01 | 7.20E-01 | PSMB11 |
| Interleukin-3, 5 and GM-CSF signaling | Reactome | R-HSA-512988 | 1 | 252 | 6.66E-01 | 7.20E-01 | PSMB11 |
| Chromatin organization | Reactome | R-HSA-4839726 | 1 | 265 | 6.84E-01 | 7.32E-01 | KDM4C |
| SLC-mediated transmembrane transport | Reactome | R-HSA-425407 | 1 | 265 | 6.84E-01 | 7.32E-01 | SLC26A4 |
| Chromatin modifying enzymes | Reactome | R-HSA-3247509 | 1 | 265 | 6.84E-01 | 7.32E-01 | KDM4C |
| MAPK family signaling cascades | Reactome | R-HSA-5683057 | 1 | 269 | 6.89E-01 | 7.36E-01 | PSMB11 |
| IRS-mediated signalling | Reactome | R-HSA-112399 | 1 | 276 | 6.99E-01 | 7.43E-01 | PSMB11 |
| Insulin receptor signalling cascade | Reactome | R-HSA-74751 | 1 | 279 | 7.03E-01 | 7.46E-01 | PSMB11 |
| IGF1R signaling cascade | Reactome | R-HSA-2428924 | 1 | 280 | 7.04E-01 | 7.47E-01 | PSMB11 |
| Signaling by Type 1 Insulin-like Growth Factor 1 Receptor (IGF1R) | Reactome | R-HSA-2404192 | 1 | 280 | 7.04E-01 | 7.47E-01 | PSMB11 |
| IRS-related events triggered by IGF1R | Reactome | R-HSA-2428928 | 1 | 280 | 7.04E-01 | 7.47E-01 | PSMB11 |
| Asparagine N-linked glycosylation | Reactome | R-HSA-446203 | 1 | 299 | 7.27E-01 | 7.67E-01 | EDEM3 |
| Signaling by Insulin receptor | Reactome | R-HSA-74752 | 1 | 303 | 7.32E-01 | 7.70E-01 | PSMB11 |
| VEGFA-VEGFR2 Pathway | Reactome | R-HSA-4420097 | 1 | 309 | 7.39E-01 | 7.76E-01 | PSMB11 |
| Signaling by SCF-KIT | Reactome | R-HSA-1433557 | 1 | 313 | 7.44E-01 | 7.80E-01 | PSMB11 |
| Signaling by ERBB4 | Reactome | R-HSA-1236394 | 1 | 317 | 7.48E-01 | 7.84E-01 | PSMB11 |
| Downstream signaling of activated FGFR4 | Reactome | R-HSA-5654716 | 1 | 317 | 7.48E-01 | 7.84E-01 | PSMB11 |
| Downstream signaling of activated FGFR2 | Reactome | R-HSA-5654696 | 1 | 317 | 7.48E-01 | 7.84E-01 | PSMB11 |
| Downstream signaling of activated FGFR3 | Reactome | R-HSA-5654708 | 1 | 317 | 7.48E-01 | 7.84E-01 | PSMB11 |
| Signaling by VEGF | Reactome | R-HSA-194138 | 1 | 318 | 7.49E-01 | 7.84E-01 | PSMB11 |
| Signaling by FGFR4 | Reactome | R-HSA-5654743 | 1 | 320 | 7.51E-01 | 7.86E-01 | PSMB11 |
| Downstream signaling of activated FGFR1 | Reactome | R-HSA-5654687 | 1 | 320 | 7.51E-01 | 7.86E-01 | PSMB11 |
| Signaling by FGFR3 | Reactome | R-HSA-5654741 | 1 | 321 | 7.52E-01 | 7.87E-01 | PSMB11 |
| Class A/1 (Rhodopsin-like receptors) | Reactome | R-HSA-373076 | 1 | 325 | 7.57E-01 | 7.88E-01 | SSTR1 |
| Signaling by Wnt | Reactome | R-HSA-195721 | 1 | 329 | 7.61E-01 | 7.92E-01 | PSMB11 |
| Downstream signal transduction | Reactome | R-HSA-186763 | 1 | 329 | 7.61E-01 | 7.92E-01 | PSMB11 |
| Signaling by EGFR | Reactome | R-HSA-177929 | 1 | 338 | 7.70E-01 | 8.00E-01 | PSMB11 |
| Metabolism of amino acids and derivatives | Reactome | R-HSA-71291 | 1 | 339 | 7.71E-01 | 8.00E-01 | PSMB11 |
| Neuronal System | Reactome | R-HSA-112316 | 1 | 339 | 7.71E-01 | 8.00E-01 | LRFN4 |
| Infectious disease | Reactome | R-HSA-5663205 | 1 | 348 | 7.80E-01 | 8.07E-01 | PSMB11 |
| Signaling by FGFR2 | Reactome | R-HSA-5654738 | 1 | 349 | 7.81E-01 | 8.08E-01 | PSMB11 |
| Fc epsilon receptor (FCERI) signaling | Reactome | R-HSA-2454202 | 1 | 350 | 7.82E-01 | 8.09E-01 | PSMB11 |
| NGF signalling via TRKA from the plasma membrane | Reactome | R-HSA-187037 | 1 | 363 | 7.94E-01 | 8.17E-01 | PSMB11 |
| Cellular responses to stress | Reactome | R-HSA-2262752 | 1 | 387 | 8.14E-01 | 8.34E-01 | CCS |
| Signaling by Interleukins | Reactome | R-HSA-449147 | 1 | 400 | 8.24E-01 | 8.43E-01 | PSMB11 |
| Gastrin-CREB signalling pathway via PKC and MAPK | Reactome | R-HSA-881907 | 1 | 421 | 8.40E-01 | 8.55E-01 | PSMB11 |
| Vesicle-mediated transport | Reactome | R-HSA-5653656 | 1 | 573 | 9.18E-01 | 9.24E-01 | CD163 |
| Cytokine Signaling in Immune system | Reactome | R-HSA-1280215 | 1 | 624 | 9.34E-01 | 9.38E-01 | PSMB11 |
| Metabolism | Reactome | R-HSA-1430728 | 1 | 1975 | 1.00E+00 | 1.00E+00 | PSMB11 |
