# Supplementary material for: Identification of potential candidate genes and pathways in atrioventricular nodal reentry tachycardia by whole‐exome sequencing
Source: Clin Transl Med. 2020 Apr 30;10(1):238–57. doi: 10.1002/ctm2.25 (PMC7240861; doi:10.1002/ctm2.25)
Supplement: Supplementary file 8 — Supporting Information S7 [file CTM2-10-238-s012.doc]

**S10: Rare variant information in candidate gene from burden analysis (MAF < 0.001)**

| **Gene**  **name** | **Variant**  **type** | **Functional** | **Hgv.c** | **Hgv.p** | **KEGG**  **EAS AF** | **ExAC**  **EAS AF** | **GnomAD**  **Exome AF** | **Cases**  **(n)** | **Cases**  **Mut. Type** | **Controls**  **(n)** | **Controls**  **Mut. Type** |
| --- | --- | --- | --- | --- | --- | --- | --- | --- | --- | --- | --- |
| ABCC8 | SNP | missense_variant | c.1069G>A | p.Val357Ile | 0 | 0 | 3.66E-05 | 1 | het | 0 |  |
|  |  |  | c.2114G>A | p.Arg705Gln | 0 | 0 | 1.63E-05 | 1 | het | 0 |  |
|  |  |  | c.2266C>T | p.Arg756Trp | 0 | 0 | 1.68E-05 | 1 | het | 0 |  |
| AP1G2 | SNP | missense_variant | c.1109C>T | p.Thr370Met | 0 | 0 | 1.22E-05 | 1 | het | 0 |  |
| ASPH | InDel | frameshift_variant | c.1955delA | p.Asn652fs | 0 | 0 | 0 | 1 | het | 0 |  |
|  | SNP | missense_variant | c.1288C>T | p.Pro430Ser | 0 | 0 | 0 | 1 | het | 0 |  |
|  |  |  | c.2141G>C | p.Gly714Ala | 0 | 0 | 0 | 1 | het | 0 |  |
|  |  |  | c.461A>G | p.Glu154Gly | 0 | 0 | 0 | 1 | het | 0 |  |
| ATP2C2 | SNP | missense_variant | c.1024G>A | p.Val342Ile | 0 | 0 | 2.44E-05 | 1 | het | 0 |  |
|  |  |  | c.1078A>G | p.Ile360Val | 0 | 0 | 8.12E-06 | 1 | het | 0 |  |
|  |  |  | c.2096G>C | p.Ser699Thr | 0 | 0 | 0 | 1 | het | 0 |  |
|  |  |  | c.2635G>C | p.Asp879His | 0 | 0 | 0 | 1 | het | 0 |  |
|  |  |  | c.2638C>T | p.Leu880Phe | 0 | 0 | 0 | 1 | het | 0 |  |
|  |  |  | c.2898G>T | p.Lys966Asn | 0 | 0.001 | 0 | 1 | het | 0 |  |
|  |  |  | c.629C>T | p.Thr210Met | 0 | 0 | 9.77E-05 | 1 | het | 0 |  |
|  |  |  | c.724C>T | p.Leu242Phe | 0 | 0 | 1.22E-05 | 0 |  | 1 | het |
| BEGAIN | SNP | missense_variant | c.1526G>C | p.Gly509Ala | 0 | 0.001 | 2.69E-05 | 1 | het | 0 |  |
|  |  |  | c.692C>A | p.Pro231Gln | 0 | 0 | 0 | 1 | het | 0 |  |
|  |  |  | c.755G>A | p.Arg252Gln | 0 | 0 | 0 | 1 | het | 0 |  |
| CD163 | SNP | missense_variant | c.1456C>T | p.Pro486Ser | 0 | 0 | 0 | 1 | het | 0 |  |
|  |  |  | c.2939A>G | p.Lys980Arg | 0 | 0 | 0 | 1 | het | 0 |  |
|  |  |  | c.3035G>A | p.Arg1012His | 0 | 0 | 4.07E-06 | 1 | het | 0 |  |
|  |  |  | c.3470A>G | p.Tyr1157Cys | 0 | 0 | 0 | 1 | het | 0 |  |
| CFTR | SNP | missense_variant | c.1392G>T | p.Lys464Asn | 0 | 0 | 0 | 2 | het,het | 2 | het,het |
|  |  |  | c.1407G>T | p.Met469Ile | 0 | 0 | 0 | 0 |  | 1 | het |
|  |  |  | c.3263A>G | p.Asn1088Ser | 0 | 0 | 0 | 1 | het | 0 |  |
|  |  |  | c.4157G>T | p.Arg1386Ile | 0 | 0 | 0 | 0 |  | 1 | het |
|  |  |  | c.509G>A | p.Arg170His | 0 | 0 | 5.18E-04 | 1 | het | 0 |  |
|  |  |  | c.890G>A | p.Arg297Gln | 0 | 0 | 5.62E-04 | 1 | het | 0 |  |
|  |  | stop_gained | c.1657C>T | p.Arg553* | 0 | 0 | 7.33E-05 | 1 | het | 0 |  |
| COG4 | SNP | missense_variant | c.2203T>G | p.Ser735Ala | 0 | 0 | 0 | 1 | het | 0 |  |
|  |  |  | c.342C>A | p.Ser114Arg | 0 | 0.001 | 4.47E-05 | 1 | het | 0 |  |
|  |  |  | c.40C>G | p.Leu14Val | 0 | 0 | 0 | 1 | het | 0 |  |
| COL4A3 | SNP | missense_variant | c.1960G>A | p.Val654Ile | 0 | 0 | 4.06E-06 | 1 | het | 0 |  |
|  |  |  | c.247C>A | p.Leu83Ile | 0 | 0 | 1.22E-05 | 1 | het | 0 |  |
|  |  |  | c.3659T>A | p.Ile1220Lys | 0 | 0 | 0 | 1 | het | 0 |  |
|  |  |  | c.4295G>A | p.Arg1432His | 0 | 0.001 | 3.33E-04 | 1 | het | 0 |  |
|  |  |  | c.685C>T | p.Arg229Trp | 0 | 0 | 4.88E-05 | 1 | het | 0 |  |
| COL5A1 | SNP | missense_variant | c.1052C>T | p.Pro351Leu | 0 | 0 | 1.22E-05 | 1 | het | 0 |  |
|  |  |  | c.1291G>A | p.Gly431Arg | 0 | 0 | 2.05E-05 | 0 |  | 1 | het |
|  |  |  | c.1345C>T | p.Arg449Trp | 0 | 0 | 1.02E-04 | 1 | het | 0 |  |
|  |  |  | c.257C>G | p.Pro86Arg | 0 | 0 | 3.25E-05 | 1 | het | 0 |  |
|  |  |  | c.4136C>G | p.Pro1379Arg | 0 | 0 | 0 | 1 | het | 0 |  |
|  |  |  | c.487G>A | p.Gly163Ser | 0 | 0 | 4.21E-05 | 1 | het | 0 |  |
|  |  |  | c.5293C>T | p.Arg1765Cys | 0 | 0 | 1.02E-04 | 1 | het | 1 | het |
|  |  |  | c.889G>A | p.Val297Met | 0 | 0 | 8.13E-06 | 1 | het | 0 |  |
| CSF2RB | SNP | missense_variant | c.1648C>T | p.His550Tyr | 0 | 0 | 4.06E-06 | 1 | het | 0 |  |
|  |  |  | c.1816C>T | p.Arg606Cys | 0 | 0 | 3.31E-05 | 1 | het | 0 |  |
| DOK4 | SNP | missense_variant | c.193G>A | p.Val65Ile | 0 | 0 | 0 | 1 | het | 0 |  |
|  |  |  | c.577C>T | p.Arg193Cys | 0 | 0 | 3.26E-05 | 1 | het | 0 |  |
| EPHB4 | SNP | missense_variant | c.118G>A | p.Gly40Arg | 0 | 0 | 1.22E-05 | 1 | het | 0 |  |
|  |  |  | c.1603C>T | p.Arg535Trp | 0 | 0 | 3.67E-05 | 1 | het | 0 |  |
|  |  |  | c.2674G>T | p.Gly892Cys | 0 | 0 | 0 | 0 |  | 1 | het |
|  |  |  | c.351G>C | p.Glu117Asp | 0 | 0 | 4.09E-06 | 1 | het | 0 |  |
|  |  |  | c.365C>T | p.Thr122Met | 0 | 0 | 1.64E-05 | 1 | het | 0 |  |
|  |  |  | c.37G>A | p.Ala13Thr | 0 | 0 | 0 | 1 | het | 0 |  |
|  |  |  | c.964A>G | p.Thr322Ala | 0 | 0 | 0 | 1 | het | 0 |  |
| EVL | InDel | frameshift_variant | c.567_570delACCG | p.Pro192fs | 0 | 0 | 8.66E-06 | 12 | het,het,het,het,het,het,het,het,het,het,het,het | 0 |  |
| GAD2 | SNP | missense_variant | c.1194G>A | p.Met398Ile | 0 | 0 | 0 | 1 | het | 0 |  |
| HEPH | SNP | missense_variant | c.1191G>T | p.Trp397Cys | 0 | 0 | 0 | 1 | het | 0 |  |
|  |  |  | c.2555G>A | p.Arg852Gln | 0 | 0 | 2.96E-05 | 1 | het | 0 |  |
| HIP1R | SNP | missense_variant | c.1012G>A | p.Asp338Asn | 0 | 0 | 6.70E-06 | 1 | het | 0 |  |
|  |  |  | c.824G>A | p.Arg275Gln | 0 | 0 | 0 | 1 | het | 0 |  |
| HIPK2 | SNP | missense_variant | c.2237C>T | p.Ser746Phe | 0 | 0 | 0 | 1 | het | 0 |  |
|  |  |  | c.833T>C | p.Leu278Pro | 0 | 0 | 1.73E-05 | 3 | het,het,het | 0 |  |
|  |  |  | c.911T>C | p.Leu304Pro | 0 | 0.001 | 8.12E-05 | 1 | het | 0 |  |
| KCNV2 | SNP | missense_variant | c.1381G>A | p.Gly461Arg | 0 | 0 | 1.30E-04 | 0 |  | 1 | het |
|  |  |  | c.66G>C | p.Glu22Asp | 0 | 0 | 8.12E-06 | 1 | het | 0 |  |
|  |  |  | c.7A>C | p.Lys3Gln | 0 | 0 | 0 | 1 | het | 0 |  |
| LAMC1 | SNP | missense_variant | c.3194T>A | p.Met1065Lys | 0 | 0 | 0 | 1 | het | 0 |  |
|  |  |  | c.3643C>T | p.Leu1215Phe | 0 | 0 | 8.13E-06 | 0 |  | 1 | het |
|  |  |  | c.4000C>T | p.Arg1334Trp | 0 | 0 | 8.12E-06 | 1 | het | 0 |  |
|  |  |  | c.4828G>C | p.Ala1610Pro | 0 | 0 | 0 | 0 |  | 1 | het |
|  |  |  | c.5023G>A | p.Ala1675Thr | 0 | 0 | 0 | 0 |  | 1 | het |
|  |  |  | c.742A>T | p.Asn248Tyr | 0 | 0 | 0 | 0 |  | 1 | het |
| LRFN4 | SNP | missense_variant | c.1631C>T | p.Ala544Val | 0 | 0 | 2.34E-05 | 1 | het | 0 |  |
|  |  |  | c.1864G>T | p.Gly622Trp | 0 | 0 | 0 | 1 | het | 0 |  |
|  |  |  | c.473G>C | p.Arg158Pro | 0 | 0.001 | 0 | 1 | het | 0 |  |
|  |  |  | c.520C>T | p.Leu174Phe | 0 | 0 | 4.09E-06 | 1 | het | 0 |  |
|  |  |  | c.895A>G | p.Thr299Ala | 0 | 0 | 7.65E-06 | 1 | het | 0 |  |
| MMP2 | SNP | missense_variant | c.1366G>A | p.Gly456Ser | 0 | 0 | 6.50E-05 | 1 | het | 0 |  |
|  |  |  | c.1382T>G | p.Leu461Arg | 0 | 0 | 0 | 1 | het | 0 |  |
|  |  |  | c.496G>A | p.Glu166Lys | 0 | 0 | 1.89E-03 | 0 |  | 1 | het |
|  |  |  | c.685G>A | p.Asp229Asn | 0 | 0 | 8.12E-06 | 1 | het | 0 |  |
|  |  |  | c.751G>A | p.Gly251Ser | 0 | 0 | 0 | 1 | het | 0 |  |
| NOS1 | InDel | frameshift_variant | c.3557-1_3569dupACACACACACACAC | p.Leu1191fs | 0 | 0 | 0 | 11 | het,het,het,het,het,het,hom,het,het,het,het | 12 | hom,hom,het,hom,hom,het,het,het,hom,het,het,het |
|  |  |  | c.3557-3_3569dupACACACACACACACAC | p.Leu1191fs | 0 | 0 | 0 | 25 | het,het,het,het,het,het,het,hom,het,het,het,het,het,het,hom,het,het,het,het,hom,het,het,het,het,het | 18 | het,hom,hom,het,het,het,het,het,het,het,het,het,het,hom,het,het,hom,hom |
|  |  |  | c.3568_3569dupAC | p.Leu1191fs | 0 | 0 | 0 | 7 | het,het,het,het,het,het,het | 3 | het,het,het |
|  | SNP | missense_variant | c.102G>C | p.Glu34Asp | 0 | 0 | 0 | 0 |  | 1 | het |
|  |  |  | c.1631A>G | p.Glu544Gly | 0 | 0 | 0 | 1 | het | 0 |  |
|  |  |  | c.2579C>T | p.Pro860Leu | 0 | 0 | 0 | 1 | het | 0 |  |
|  |  | splice_acceptor_variant | c.3557-1A>T | . | 0 | 0 | 3.27E-05 | 0 |  | 1 | het |
| PIK3CB | SNP | missense_variant | c.2255G>A | p.Arg752Gln | 0 | 0 | 0 | 1 | het | 0 |  |
|  |  |  | c.458G>A | p.Arg153His | 0 | 0 | 1.63E-05 | 1 | het | 0 |  |
|  |  |  | c.49A>G | p.Ile17Val | 0 | 0 | 0 | 0 |  | 1 | het |
| PPFIA1 | SNP | missense_variant | c.206A>G | p.His69Arg | 0 | 0 | 1.22E-05 | 1 | het | 0 |  |
|  |  |  | c.2543T>G | p.Leu848Trp | 0 | 0 | 8.13E-06 | 1 | het | 0 |  |
|  |  |  | c.2836A>G | p.Ser946Gly | 0 | 0 | 4.08E-06 | 1 | het | 0 |  |
| PRKAG2 | SNP | missense_variant | c.250C>T | p.Arg84Trp | 0 | 0 | 1.46E-04 | 1 | het | 0 |  |
| PSMB11 | SNP | missense_variant | c.338G>A | p.Arg113Gln | 0 | 0 | 8.95E-05 | 1 | het | 0 |  |
|  |  |  | c.569G>A | p.Arg190His | 0 | 0 | 0 | 1 | het | 0 |  |
|  |  |  | c.601G>A | p.Ala201Thr | 0 | 0 | 0 | 1 | het | 0 |  |
|  |  |  | c.76G>A | p.Gly26Ser | 0 | 0 | 1.51E-04 | 1 | het | 0 |  |
| ROBO1 | SNP | missense_variant | c.1663A>G | p.Thr555Ala | 0 | 0 | 0 | 1 | het | 0 |  |
|  |  |  | c.185G>A | p.Arg62His | 0 | 0 | 2.89E-05 | 1 | het | 0 |  |
|  |  |  | c.2150A>G | p.Asn717Ser | 0 | 0 | 0 | 1 | het | 0 |  |
|  |  |  | c.2189C>T | p.Thr730Met | 0 | 0 | 1.47E-04 | 1 | het | 0 |  |
|  |  |  | c.3151A>C | p.Lys1051Gln | 0 | 0 | 0 | 0 |  | 1 | het |
|  |  |  | c.4286G>A | p.Arg1429His | 0 | 0.001 | 9.50E-04 | 1 | het | 0 |  |
|  |  |  | c.4360G>T | p.Val1454Leu | 0 | 0 | 1.13E-03 | 1 | het | 0 |  |
|  |  |  | c.731A>G | p.Asn244Ser | 0 | 0 | 0 | 1 | het | 0 |  |
| SCN1A | SNP | missense_variant | c.2141T>G | p.Met714Arg | 0 | 0 | 0 | 1 | het | 0 |  |
|  |  |  | c.3053G>A | p.Arg1018Lys | 0 | 0 | 2.52E-05 | 1 | het | 0 |  |
|  |  |  | c.3176A>T | p.Asp1059Val | 0 | 0 | 4.48E-05 | 1 | het | 0 |  |
| SFTPA2 | SNP | missense_variant | c.107C>T | p.Ala36Val | 0 | 0 | 9.83E-04 | 9 | het,het,het,het,het,hom,het,het,het | 0 |  |
|  |  |  | c.16A>G | p.Ser6Gly | 0 | 0 | 0 | 0 |  | 1 | het |
|  |  |  | c.199G>C | p.Val67Leu | 0 | 0.001 | 2.40E-02 | 1 | het | 0 |  |
|  |  |  | c.292G>A | p.Val98Ile | 0 | 0 | 0 | 0 |  | 1 | het |
|  |  |  | c.389C>T | p.Ala130Val | 0 | 0 | 0 | 1 | het | 0 |  |
|  |  |  | c.637G>A | p.Gly213Ser | 0 | 0 | 0 | 0 |  | 1 | het |
|  |  |  | c.778A>T | p.Asn260Tyr | 0 | 0 | 0 | 1 | het | 0 |  |
| SLC12A4 | SNP | missense_variant | c.1394C>T | p.Ser465Phe | 0 | 0 | 1.22E-05 | 0 |  | 1 | het |
|  |  |  | c.1454G>A | p.Arg485Gln | 0 | 0 | 2.90E-05 | 1 | het | 0 |  |
|  |  |  | c.1558G>A | p.Ala520Thr | 0 | 0 | 8.13E-06 | 1 | het | 0 |  |
|  |  |  | c.212G>C | p.Arg71Pro | 0 | 0 | 0 | 0 |  | 1 | het |
|  |  |  | c.31G>A | p.Val11Ile | 0 | 0 | 0 | 1 | het | 0 |  |
|  |  |  | c.3239G>A | p.Arg1080His | 0 | 0 | 0 | 1 | het | 0 |  |
|  |  |  | c.871C>T | p.Leu291Phe | 0 | 0 | 4.06E-06 | 1 | het | 0 |  |
| SLC26A4 | SNP | missense_variant | c.1405C>T | p.Pro469Ser | 0 | 0 | 4.07E-06 | 1 | het | 0 |  |
|  |  |  | c.1826T>G | p.Val609Gly | 0 | 0 | 1.04E-02 | 1 | het | 0 |  |
|  |  |  | c.1882G>T | p.Asp628Tyr | 0 | 0 | 0 | 1 | het | 0 |  |
|  |  |  | c.853G>A | p.Val285Ile | 0 | 0 | 0 | 1 | het | 0 |  |
| SLC9B1 | SNP | missense_variant | c.1549G>A | p.Ala517Thr | 0 | 0 | 4.09E-06 | 4 | het,het,het,het | 4 | het,het,het,het |
|  |  |  | c.1562G>A | p.Arg521Gln | 0 | 0 | 4.09E-06 | 4 | het,het,het,het | 3 | het,het,het |
|  |  |  | c.1573A>G | p.Thr525Ala | 0 | 0 | 1.45E-04 | 77 | het,het,het,het,het,het,het,het,het,het,het,het,het,het,het,het,het,het,het,het,het,het,het,het,het,het,het,het,het,het,het,het,het,het,het,het,het,het,het,het,het,het,het,het,het,het,het,het,het,het,het,het,het,het,het,het,het,het,het,het,het,het,het,het,het,het,het,het,het,het,het,het,het,het,het,het,het | 82 | het,het,het,het,het,het,het,het,het,het,het,het,het,het,het,het,het,het,het,het,het,het,het,het,het,het,het,het,het,het,het,het,het,het,het,het,het,het,het,het,het,het,het,het,het,het,het,het,het,het,het,het,het,het,het,het,het,het,het,het,het,het,het,het,het,het,het,het,het,het,het,het,het,het,het,het,het,het,het,het,het,het |
|  |  |  | c.1723G>C | p.Ala575Pro | 0 | 0 | 1.23E-05 | 1 | het | 0 |  |
|  |  |  | c.1724C>T | p.Ala575Val | 0 | 0 | 2.26E-04 | 1 | het | 0 |  |
|  |  |  | c.647T>C | p.Ile216Thr | 0 | 0 | 1.23E-05 | 1 | het | 0 |  |
| SYT10 | SNP | missense_variant | c.1003C>G | p.Leu335Val | 0 | 0 | 4.07E-06 | 1 | het | 0 |  |
| TCF7L1 | SNP | missense_variant | c.1616C>T | p.Ala539Val | 0 | 0 | 0 | 1 | het | 0 |  |
|  |  |  | c.1633C>T | p.Arg545Trp | 0 | 0 | 0 | 1 | het | 0 |  |
|  |  |  | c.1717G>A | p.Ala573Thr | 0 | 0 | 0 | 0 |  | 0 |  |
|  |  |  | c.406G>A | p.Ala136Thr | 0 | 0.001 | 1.57E-04 | 0 |  | 1 | het |
|  |  |  | c.538C>T | p.Pro180Ser | 0 | 0 | 1.58E-05 | 1 | het | 0 |  |
|  |  |  | c.943G>A | p.Val315Ile | 0 | 0 | 2.45E-05 | 1 | het | 0 |  |
| TSPOAP1 | SNP | missense_variant | c.1955G>A | p.Gly652Glu | 0 | 0 | 1.63E-05 | 1 | het | 0 |  |
|  |  |  | c.2383C>T | p.Arg795Cys | 0 | 0 | 0 | 0 |  | 1 | het |
|  |  |  | c.2472T>G | p.His824Gln | 0 | 0 | 0 | 1 | het | 0 |  |
|  |  |  | c.3394C>G | p.Pro1132Ala | 0 | 0 | 2.71E-05 | 0 |  | 1 | het |
|  |  |  | c.4064G>C | p.Arg1355Pro | 0 | 0 | 0 | 0 |  | 1 | het |
|  |  |  | c.4258C>T | p.Arg1420Trp | 0 | 0 | 0 | 1 | het | 0 |  |
|  |  |  | c.4339C>T | p.Arg1447Trp | 0 | 0.001 | 4.74E-05 | 1 | het | 0 |  |
|  |  |  | c.5551C>T | p.Pro1851Ser | 0 | 0 | 0 | 0 |  | 1 | het |

*Note: KEGG, Kyoto Encyclopedia of Genes and Genomes；ExAC, Exome Aggregation Consortium; EAS, East Asian; GnomAD, Genome Aggregation Database; AF, allele frequency.*
