# Supplementary material for: Identification of potential candidate genes and pathways in atrioventricular nodal reentry tachycardia by whole‐exome sequencing
Source: Clin Transl Med. 2020 Apr 30;10(1):238–57. doi: 10.1002/ctm2.25 (PMC7240861; doi:10.1002/ctm2.25)
Supplement: Supplementary file 9 — Supporting Information S8 [file CTM2-10-238-s013.docx]

**S12: Validation of candidate genes in UK Biobank**

| **Gene Name** | **Start Position** | **End Position** | **Rare Variants(N)** | **MAC Case** | **MAC Control** | **Case** | **Control** | **P Value** | **Pheno** | **Phenostring** |
| --- | --- | --- | --- | --- | --- | --- | --- | --- | --- | --- |
| ABCC8 | 11:17393057:A:T | 11:17442719:C:T | 122 | 5 | 306.0013982 | 93 | 9207 | 0.5483204 | 427.5 | Arrhythmia (cardiac) NOS |
| ABCC8 | 11:17393005:G:A | 11:17442719:C:A | 110 | 2 | 306.0015793 | 95 | 9405 | 0.700250685 | 426.91 | Cardiac pacemaker in situ |
| ABCC8 | 11:17393005:G:A | 11:17470099:A:G | 319 | 46.00011306 | 1353.003505 | 1468 | 42760 | 0.750372233 | 427.2 | Atrial fibrillation and flutter |
| ABCC8 | 11:17393076:C:G | 11:17396914:A:C | 95 | 1 | 254.0010259 | 78 | 7722 | 0.535945588 | 427.12 | Paroxysmal ventricular tachycardia |
| ABCC8 | 11:17393005:G:A | 11:17470099:A:G | 235 | 6.000072469 | 878.0024642 | 276 | 27324 | 0.568353311 | 427.11 | Paroxysmal supraventricular tachycardia |
| ABCC8 | 11:17393005:G:A | 11:17442719:C:A | 121 | 2 | 357.0027785 | 108 | 10692 | 0.554576963 | 426.9 | Cardiac pacemaker/device in situ |
| ABCC8 | 11:17393005:G:A | 11:17453117:A:G | 124 | 2 | 356.0012933 | 116 | 11484 | 0.670951978 | 425 | Cardiomyopathy |
| ABCC8 | 11:17393005:G:A | 11:17470099:A:G | 284 | 7.000056502 | 1118.003786 | 354 | 35046 | 0.385012748 | 427.1 | Paroxysmal tachycardia, unspecified |
| ABCC8 | 11:17393005:G:A | 11:17453117:A:G | 121 | 1 | 332.0029817 | 104 | 10296 | 0.352196286 | 427.4 | Cardiac arrest and ventricular fibrillation |
| ABCC8 | 11:17393005:G:A | 11:17470099:A:G | 324 | 73.00015454 | 1353.003466 | 2570 | 42728 | 0.534463102 | 427 | Cardiac dysrhythmias |
| ABCC8 | 11:17393005:G:A | 11:17453117:A:G | 107 | 1 | 272.0016096 | 87 | 8613 | 0.49482257 | 427.42 | Cardiac arrest |
| AP1G2 | 14:23559762:D:1 | 14:23567449:A:G | 209 | 75.00046409 | 1433.014844 | 2570 | 42728 | 0.475361598 | 427 | Cardiac dysrhythmias |
| AP1G2 | 14:23559766:C:T | 14:23567449:A:G | 101 | 3 | 376.0060391 | 116 | 11484 | 0.922648274 | 425 | Cardiomyopathy |
| AP1G2 | 14:23559762:D:1 | 14:23567449:A:G | 181 | 6 | 1180.009922 | 354 | 35046 | 0.169168992 | 427.1 | Paroxysmal tachycardia, unspecified |
| AP1G2 | 14:23559762:D:1 | 14:23567449:A:G | 159 | 5 | 933.0077957 | 276 | 27324 | 0.268057826 | 427.11 | Paroxysmal supraventricular tachycardia |
| AP1G2 | 14:23559762:D:1 | 14:23567449:A:G | 206 | 51.00038483 | 1434.015181 | 1468 | 42760 | 0.908724973 | 427.2 | Atrial fibrillation and flutter |
| AP1G2 | 14:23559786:A:G | 14:23567449:A:G | 91 | 5.00009261 | 378.003706 | 108 | 10692 | 0.489180544 | 426.9 | Cardiac pacemaker/device in situ |
| AP1G2 | 14:23559762:D:1 | 14:23567449:A:G | 75 | 1 | 248 | 78 | 7722 | 0.512614343 | 427.12 | Paroxysmal ventricular tachycardia |
| AP1G2 | 14:23559766:C:T | 14:23567449:A:G | 88 | 0 | 332.0036588 | 93 | 9207 | 0.146021565 | 427.5 | Arrhythmia (cardiac) NOS |
| AP1G2 | 14:23559762:D:1 | 14:23567449:A:G | 99 | 0 | 345.0059652 | 104 | 10296 | 0.106649922 | 427.4 | Cardiac arrest and ventricular fibrillation |
| AP1G2 | 14:23559786:A:G | 14:23567449:A:G | 79 | 5.000105285 | 331.0040029 | 95 | 9405 | 0.309103683 | 426.91 | Cardiac pacemaker in situ |
| AP1G2 | 14:23559766:C:T | 14:23567449:A:G | 87 | 0 | 289.0069017 | 87 | 8613 | 0.15282899 | 427.42 | Cardiac arrest |
| ASPH | 8:61503374:G:C | 8:61651126:T:C | 190 | 12.0000565 | 1363.043809 | 354 | 35046 | 0.86384223 | 427.1 | Paroxysmal tachycardia, unspecified |
| ASPH | 8:61503374:G:C | 8:61643385:C:T | 108 | 2 | 366.0106392 | 95 | 9405 | 0.651631123 | 426.91 | Cardiac pacemaker in situ |
| ASPH | 8:61503374:G:C | 8:61651049:C:T | 102 | 5 | 443.002587 | 116 | 11484 | 0.77042658 | 425 | Cardiomyopathy |
| ASPH | 8:61503374:G:C | 8:61651126:T:C | 217 | 51 | 1664.043476 | 1468 | 42760 | 0.161469601 | 427.2 | Atrial fibrillation and flutter |
| ASPH | 8:61503374:G:C | 8:61643385:C:T | 117 | 2 | 404.0094503 | 108 | 10692 | 0.56655078 | 426.9 | Cardiac pacemaker/device in situ |
| ASPH | 8:61503374:G:C | 8:61651126:T:C | 220 | 89.00059642 | 1663.043487 | 2570 | 42728 | 0.04035335 | 427 | Cardiac dysrhythmias |
| ASPH | 8:61503374:G:C | 8:61651126:T:C | 175 | 11.00007247 | 1078.041764 | 276 | 27324 | 1 | 427.11 | Paroxysmal supraventricular tachycardia |
| ASPH | 8:61503374:G:C | 8:61618977:C:A | 82 | 0 | 332.0125395 | 87 | 8613 | 0.148089537 | 427.42 | Cardiac arrest |
| ASPH | 8:61503378:C:T | 8:61618977:C:A | 87 | 0 | 311.009109 | 78 | 7722 | 0.186717597 | 427.12 | Paroxysmal ventricular tachycardia |
| ASPH | 8:61503374:G:C | 8:61618977:C:A | 85 | 0 | 386.0169387 | 104 | 10296 | 0.122838297 | 427.4 | Cardiac arrest and ventricular fibrillation |
| ASPH | 8:61503374:G:C | 8:61618977:C:A | 82 | 4 | 384.0079619 | 93 | 9207 | 0.876766846 | 427.5 | Arrhythmia (cardiac) NOS |
| ATP2C2 | 16:84368617:T:C | 16:84451919:A:T | 170 | 6.001218472 | 644.1806411 | 93 | 9207 | 1 | 427.5 | Arrhythmia (cardiac) NOS |
| ATP2C2 | 16:84368652:C:T | 16:84453237:T:C | 186 | 9.002737995 | 676.0302523 | 95 | 9405 | 0.552234642 | 426.91 | Cardiac pacemaker in situ |
| ATP2C2 | 16:84368652:C:T | 16:84459189:G:A | 423 | 104.0063446 | 3043.28868 | 1468 | 42760 | 0.688780289 | 427.2 | Atrial fibrillation and flutter |
| ATP2C2 | 16:84368617:T:C | 16:84459189:G:A | 428 | 184.0134048 | 3039.285356 | 2570 | 42728 | 0.456379819 | 427 | Cardiac dysrhythmias |
| ATP2C2 | 16:84368662:C:A | 16:84459119:G:A | 162 | 7.000115274 | 626.0432779 | 87 | 8613 | 1 | 427.42 | Cardiac arrest |
| ATP2C2 | 16:84368652:C:T | 16:84453372:G:T | 335 | 21.00032692 | 1950.09177 | 276 | 27324 | 0.582032401 | 427.11 | Paroxysmal supraventricular tachycardia |
| ATP2C2 | 16:84368652:C:T | 16:84453236:G:T | 139 | 3 | 547.0323502 | 78 | 7722 | 0.485022302 | 427.12 | Paroxysmal ventricular tachycardia |
| ATP2C2 | 16:84368652:C:T | 16:84459189:G:A | 382 | 26.00173361 | 2491.276686 | 354 | 35046 | 0.874531012 | 427.1 | Paroxysmal tachycardia, unspecified |
| ATP2C2 | 16:84368652:C:T | 16:84453236:G:T | 188 | 8 | 812.044102 | 116 | 11484 | 1 | 425 | Cardiomyopathy |
| ATP2C2 | 16:84368662:C:A | 16:84459119:G:A | 178 | 7.000096413 | 735.0493929 | 104 | 10296 | 1 | 427.4 | Cardiac arrest and ventricular fibrillation |
| ATP2C2 | 16:84368652:C:T | 16:84453237:T:C | 202 | 11.00268643 | 775.0367163 | 108 | 10692 | 0.528713095 | 426.9 | Cardiac pacemaker/device in situ |
| BEGAIN | 14:100538013:C:T | 14:100546601:C:T | 37 | 3 | 205.0006453 | 93 | 9207 | 0.429486711 | 427.5 | Arrhythmia (cardiac) NOS |
| BEGAIN | 14:100538013:C:G | 14:100544999:C:T | 88 | 3.002731308 | 609.1860346 | 276 | 27324 | 0.334616099 | 427.11 | Paroxysmal supraventricular tachycardia |
| BEGAIN | 14:100538034:C:T | 14:100544999:C:T | 33 | 4.000479157 | 204.0841973 | 87 | 8613 | 0.252589017 | 427.42 | Cardiac arrest |
| BEGAIN | 14:100538013:C:G | 14:100544999:C:T | 100 | 3.002839145 | 781.1863401 | 354 | 35046 | 0.140763945 | 427.1 | Paroxysmal tachycardia, unspecified |
| BEGAIN | 14:100538034:C:T | 14:100546634:G:A | 43 | 4.001168473 | 246.1377908 | 116 | 11484 | 0.105846279 | 425 | Cardiomyopathy |
| BEGAIN | 14:100538001:T:C | 14:100544999:C:T | 113 | 31.00912998 | 971.2753046 | 1468 | 42760 | 0.291225896 | 427.2 | Atrial fibrillation and flutter |
| BEGAIN | 14:100538001:T:C | 14:100544999:C:T | 115 | 58.01670623 | 969.2688723 | 2570 | 42728 | 0.666993 | 427 | Cardiac dysrhythmias |
| BEGAIN | 14:100538034:C:T | 14:100544999:C:T | 39 | 4.000901442 | 244.1245794 | 104 | 10296 | 0.417309312 | 427.4 | Cardiac arrest and ventricular fibrillation |
| BEGAIN | 14:100538013:C:G | 14:100546634:G:A | 44 | 3.001164935 | 226.0959041 | 108 | 10692 | 0.758457934 | 426.9 | Cardiac pacemaker/device in situ |
| BEGAIN | 14:100538153:C:A | 14:100544999:C:T | 31 | 0.001342823 | 160.0934605 | 78 | 7722 | 0.314902967 | 427.12 | Paroxysmal ventricular tachycardia |
| BEGAIN | 14:100538013:C:G | 14:100546634:G:A | 39 | 3.000882223 | 199.0943979 | 95 | 9405 | 0.620793897 | 426.91 | Cardiac pacemaker in situ |
| CD163 | 12:7479969:C:T | 12:7502534:G:A | 53 | 1 | 130 | 93 | 9207 | 0.887297126 | 427.5 | Arrhythmia (cardiac) NOS |
| CD163 | 12:7479893:A:G | 12:7483366:C:T | 147 | 8.000056513 | 547.0031643 | 354 | 35046 | 0.591639036 | 427.1 | Paroxysmal tachycardia, unspecified |
| CD163 | 12:7479897:D:1 | 12:7501402:D:1 | 63 | 1 | 131.0002564 | 78 | 7722 | 1 | 427.12 | Paroxysmal ventricular tachycardia |
| CD163 | 12:7479893:A:G | 12:7502534:G:A | 57 | 2 | 137.0002299 | 87 | 8613 | 0.335768268 | 427.42 | Cardiac arrest |
| CD163 | 12:7479893:A:G | 12:7502534:G:A | 64 | 2 | 167.0003847 | 104 | 10296 | 0.508438496 | 427.4 | Cardiac arrest and ventricular fibrillation |
| CD163 | 12:7479895:T:C | 12:7502526:T:C | 69 | 4 | 150.0004211 | 95 | 9405 | 0.036049873 | 426.91 | Cardiac pacemaker in situ |
| CD163 | 12:7479893:A:G | 12:7483366:C:T | 127 | 7.000036241 | 434.0014132 | 276 | 27324 | 0.450606524 | 427.11 | Paroxysmal supraventricular tachycardia |
| CD163 | 12:7479895:T:C | 12:7502526:T:C | 79 | 5 | 171.0009261 | 108 | 10692 | 0.009246366 | 426.9 | Cardiac pacemaker/device in situ |
| CD163 | 12:7479893:A:G | 12:7502565:C:A | 186 | 44.00017663 | 689.0032456 | 2570 | 42728 | 1 | 427 | Cardiac dysrhythmias |
| CD163 | 12:7479893:A:G | 12:7502565:C:A | 181 | 21.00011306 | 689.0032563 | 1468 | 42760 | 0.654756565 | 427.2 | Atrial fibrillation and flutter |
| CD163 | 12:7479924:C:T | 12:7502526:T:C | 72 | 2 | 193.0006898 | 116 | 11484 | 1 | 425 | Cardiomyopathy |
| CFTR | 7:117480102:G:T | 7:117592658:G:A | 178 | 14.00023162 | 991.0424194 | 87 | 8613 | 0.101229376 | 427.42 | Cardiac arrest |
| CFTR | 7:117480105:C:T | 7:117606754:G:A | 178 | 9.000397667 | 862.040848 | 78 | 7722 | 0.539597432 | 427.12 | Paroxysmal ventricular tachycardia |
| CFTR | 7:117480102:G:T | 7:117592658:G:A | 192 | 15.00039263 | 1188.081907 | 104 | 10296 | 0.116267803 | 427.4 | Cardiac arrest and ventricular fibrillation |
| CFTR | 7:117480102:G:T | 7:117606754:G:A | 366 | 35.00394149 | 3385.508206 | 354 | 35046 | 1 | 427.1 | Paroxysmal tachycardia, unspecified |
| CFTR | 7:117480105:C:T | 7:117592658:G:A | 197 | 14.00032319 | 932.0956906 | 95 | 9405 | 0.007580472 | 426.91 | Cardiac pacemaker in situ |
| CFTR | 7:117480105:C:T | 7:117592658:G:A | 208 | 17.00075055 | 1261.130913 | 108 | 10692 | 0.05271082 | 426.9 | Cardiac pacemaker/device in situ |
| CFTR | 7:117480105:C:T | 7:117592658:G:T | 187 | 9.00133363 | 1071.144322 | 93 | 9207 | 0.805135231 | 427.5 | Arrhythmia (cardiac) NOS |
| CFTR | 7:117480105:C:T | 7:117592658:G:A | 210 | 12.00123977 | 1305.213014 | 116 | 11484 | 0.904340309 | 425 | Cardiomyopathy |
| CFTR | 7:117480102:G:T | 7:117606754:G:A | 415 | 149.0199111 | 4194.570854 | 1468 | 42760 | 0.411990303 | 427.2 | Atrial fibrillation and flutter |
| CFTR | 7:117480102:G:T | 7:117606754:G:A | 417 | 266.0399893 | 4190.564788 | 2570 | 42728 | 0.5760455 | 427 | Cardiac dysrhythmias |
| CFTR | 7:117480102:G:T | 7:117592658:G:T | 316 | 25.0025821 | 2641.421896 | 276 | 27324 | 0.927689508 | 427.11 | Paroxysmal supraventricular tachycardia |
| COG4 | 16:70481012:A:G | 16:70512433:C:T | 154 | 9.000169501 | 548.0011583 | 354 | 35046 | 0.269324455 | 427.1 | Paroxysmal tachycardia, unspecified |
| COG4 | 16:70481012:A:G | 16:70481358:C:T | 73 | 0 | 179.000463 | 108 | 10692 | 0.343840244 | 426.9 | Cardiac pacemaker/device in situ |
| COG4 | 16:70481012:A:G | 16:70481358:C:T | 63 | 0 | 168.0003847 | 104 | 10296 | 0.369902141 | 427.4 | Cardiac arrest and ventricular fibrillation |
| COG4 | 16:70481012:A:G | 16:70481358:C:T | 61 | 0 | 153.0002299 | 87 | 8613 | 0.405675045 | 427.42 | Cardiac arrest |
| COG4 | 16:70481012:A:G | 16:70512433:C:T | 175 | 35.00015454 | 671.0010377 | 2570 | 42728 | 0.742263925 | 427 | Cardiac dysrhythmias |
| COG4 | 16:70481012:A:G | 16:70481358:C:T | 73 | 2 | 167 | 116 | 11484 | 0.327791657 | 425 | Cardiomyopathy |
| COG4 | 16:70481017:C:T | 16:70512433:C:T | 135 | 8.0001087 | 425.0007609 | 276 | 27324 | 0.197847162 | 427.11 | Paroxysmal supraventricular tachycardia |
| COG4 | 16:70481012:A:G | 16:70481358:C:T | 48 | 0 | 115 | 78 | 7722 | 0.45426559 | 427.12 | Paroxysmal ventricular tachycardia |
| COG4 | 16:70481012:A:G | 16:70481358:C:T | 68 | 0 | 164.0005264 | 95 | 9405 | 0.40527311 | 426.91 | Cardiac pacemaker in situ |
| COG4 | 16:70481012:A:G | 16:70512433:C:T | 174 | 21 | 671.0009045 | 1468 | 42760 | 0.758522561 | 427.2 | Atrial fibrillation and flutter |
| COG4 | 16:70481032:T:C | 16:70512433:C:T | 62 | 4 | 151 | 93 | 9207 | 0.000476676 | 427.5 | Arrhythmia (cardiac) NOS |
| COL4A3 | 2:227164737:G:A | 2:227303932:T:C | 116 | 8.00042123 | 727.0068449 | 95 | 9405 | 0.603276187 | 426.91 | Cardiac pacemaker in situ |
| COL4A3 | 2:227164732:C:G | 2:227307709:G:A | 237 | 19 | 1589.00819 | 276 | 27324 | 0.704978969 | 427.11 | Paroxysmal supraventricular tachycardia |
| COL4A3 | 2:227164737:G:A | 2:227307709:G:A | 88 | 5 | 444.0028214 | 78 | 7722 | 0.188011388 | 427.12 | Paroxysmal ventricular tachycardia |
| COL4A3 | 2:227164732:C:G | 2:227307709:G:A | 299 | 150.0012366 | 2434.015679 | 2570 | 42728 | 1 | 427 | Cardiac dysrhythmias |
| COL4A3 | 2:227164736:C:G | 2:227311851:G:A | 132 | 6 | 582.0018273 | 104 | 10296 | 0.363133762 | 427.4 | Cardiac arrest and ventricular fibrillation |
| COL4A3 | 2:227164737:G:A | 2:227294963:G:T | 139 | 10.00025871 | 893.006382 | 116 | 11484 | 0.81792897 | 425 | Cardiomyopathy |
| COL4A3 | 2:227164732:C:G | 2:227307709:G:A | 297 | 93.00047493 | 2434.015312 | 1468 | 42760 | 0.56719639 | 427.2 | Atrial fibrillation and flutter |
| COL4A3 | 2:227164736:C:G | 2:227311851:G:A | 125 | 6 | 488.0017244 | 87 | 8613 | 0.293011213 | 427.42 | Cardiac arrest |
| COL4A3 | 2:227164737:G:A | 2:227294963:G:T | 110 | 5 | 528.0115131 | 93 | 9207 | 1 | 427.5 | Arrhythmia (cardiac) NOS |
| COL4A3 | 2:227164737:G:A | 2:227303932:T:C | 125 | 10.00037051 | 822.0068544 | 108 | 10692 | 0.138778734 | 426.9 | Cardiac pacemaker/device in situ |
| COL4A3 | 2:227164732:C:G | 2:227307709:G:A | 269 | 24 | 1985.010935 | 354 | 35046 | 0.60625934 | 427.1 | Paroxysmal tachycardia, unspecified |
| COL5A1 | 9:134642198:A:C | 9:134830107:G:A | 413 | 85.03607433 | 2885.961432 | 1468 | 42760 | 0.225257622 | 427.2 | Atrial fibrillation and flutter |
| COL5A1 | 9:134642198:A:C | 9:134830107:G:A | 182 | 4.005230895 | 633.2855778 | 95 | 9405 | 0.562942862 | 426.91 | Cardiac pacemaker in situ |
| COL5A1 | 9:134642198:A:C | 9:134830107:G:A | 418 | 153.0634416 | 2880.938245 | 2570 | 42728 | 0.392620004 | 427 | Cardiac dysrhythmias |
| COL5A1 | 9:134642215:C:T | 9:134830107:G:A | 166 | 5.007623813 | 588.2841922 | 87 | 8613 | 0.846443606 | 427.42 | Cardiac arrest |
| COL5A1 | 9:134642216:G:A | 9:134842263:A:C | 199 | 12.00429364 | 773.2298726 | 116 | 11484 | 0.274603093 | 425 | Cardiomyopathy |
| COL5A1 | 9:134642248:C:T | 9:134830107:G:A | 177 | 4.000558073 | 616.1952377 | 93 | 9207 | 0.690955326 | 427.5 | Arrhythmia (cardiac) NOS |
| COL5A1 | 9:134642198:A:C | 9:134830107:G:A | 366 | 19.01162856 | 2343.874786 | 354 | 35046 | 0.538816594 | 427.1 | Paroxysmal tachycardia, unspecified |
| COL5A1 | 9:134642198:A:C | 9:134830107:G:A | 195 | 4.004603323 | 731.2898347 | 108 | 10692 | 0.390192557 | 426.9 | Cardiac pacemaker/device in situ |
| COL5A1 | 9:134642198:A:C | 9:134780146:I:4 | 318 | 15.00881574 | 1832.715886 | 276 | 27324 | 0.641550341 | 427.11 | Paroxysmal supraventricular tachycardia |
| COL5A1 | 9:134642216:G:A | 9:134830107:G:A | 156 | 3.006553681 | 494.3021578 | 78 | 7722 | 0.655878891 | 427.12 | Paroxysmal ventricular tachycardia |
| COL5A1 | 9:134642215:C:T | 9:134830107:G:A | 183 | 7.008082398 | 681.3327321 | 104 | 10296 | 0.342896858 | 427.4 | Cardiac arrest and ventricular fibrillation |
| CSF2RB | 22:36922215:T:C | 22:36938500:T:G | 182 | 32.00006784 | 926.0016959 | 1468 | 42760 | 1 | 427.2 | Atrial fibrillation and flutter |
| CSF2RB | 22:36922267:C:A | 22:36938476:G:A | 74 | 3 | 182 | 87 | 8613 | 0.287413558 | 427.42 | Cardiac arrest |
| CSF2RB | 22:36922267:C:A | 22:36938476:G:A | 92 | 4 | 234.0005556 | 108 | 10692 | 0.008010551 | 426.9 | Cardiac pacemaker/device in situ |
| CSF2RB | 22:36923267:C:T | 22:36938500:T:G | 73 | 2 | 185.0004302 | 93 | 9207 | 0.590322138 | 427.5 | Arrhythmia (cardiac) NOS |
| CSF2RB | 22:36922215:T:C | 22:36938500:T:G | 168 | 8 | 762.000678 | 354 | 35046 | 0.749603104 | 427.1 | Paroxysmal tachycardia, unspecified |
| CSF2RB | 22:36922220:D:1 | 22:36938476:G:A | 73 | 4 | 180 | 78 | 7722 | 0.078827812 | 427.12 | Paroxysmal ventricular tachycardia |
| CSF2RB | 22:36922215:T:C | 22:36938500:T:G | 143 | 5 | 606 | 276 | 27324 | 0.880085115 | 427.11 | Paroxysmal supraventricular tachycardia |
| CSF2RB | 22:36922215:T:C | 22:36938500:T:G | 187 | 56.00006624 | 926.0017 | 2570 | 42728 | 1 | 427 | Cardiac dysrhythmias |
| CSF2RB | 22:36922267:C:A | 22:36938500:T:G | 85 | 1 | 245.0006898 | 116 | 11484 | 0.558471783 | 425 | Cardiomyopathy |
| CSF2RB | 22:36922267:C:A | 22:36938476:G:A | 84 | 4 | 202.0004211 | 95 | 9405 | 0.005619058 | 426.91 | Cardiac pacemaker in situ |
| CSF2RB | 22:36922267:C:A | 22:36938476:G:A | 79 | 5 | 219 | 104 | 10296 | 0.024129178 | 427.4 | Cardiac arrest and ventricular fibrillation |
| DOK4 | 16:57473408:T:A | 16:57473612:C:A | 30 | 1.000215077 | 88.00043015 | 93 | 9207 | 1 | 427.5 | Arrhythmia (cardiac) NOS |
| DOK4 | 16:57473412:C:G | 16:57473612:C:A | 24 | 0 | 86 | 95 | 9405 | 0.519421949 | 426.91 | Cardiac pacemaker in situ |
| DOK4 | 16:57473417:I:6 | 16:57475931:G:C | 19 | 1 | 64.00012822 | 78 | 7722 | 0.700112749 | 427.12 | Paroxysmal ventricular tachycardia |
| DOK4 | 16:57473412:C:G | 16:57473612:C:A | 58 | 3 | 318.0008192 | 354 | 35046 | 0.881303603 | 427.1 | Paroxysmal tachycardia, unspecified |
| DOK4 | 16:57473412:C:G | 16:57475935:A:G | 31 | 2 | 82.00048082 | 104 | 10296 | 0.293545463 | 427.4 | Cardiac arrest and ventricular fibrillation |
| DOK4 | 16:57473412:C:G | 16:57475935:A:G | 25 | 2 | 65.00045982 | 87 | 8613 | 0.164831055 | 427.42 | Cardiac arrest |
| DOK4 | 16:57473408:T:A | 16:57473612:C:A | 67 | 13.00027134 | 384.0007462 | 1468 | 42760 | 0.374984383 | 427.2 | Atrial fibrillation and flutter |
| DOK4 | 16:57473412:C:G | 16:57475953:T:G | 30 | 2 | 95.00025864 | 116 | 11484 | 0.11593548 | 425 | Cardiomyopathy |
| DOK4 | 16:57473412:C:G | 16:57473612:C:A | 49 | 1 | 261.0003623 | 276 | 27324 | 0.523283137 | 427.11 | Paroxysmal supraventricular tachycardia |
| DOK4 | 16:57473412:C:G | 16:57473612:C:A | 28 | 0 | 97.0003704 | 108 | 10692 | 0.49244669 | 426.9 | Cardiac pacemaker/device in situ |
| DOK4 | 16:57473408:T:A | 16:57473612:C:A | 68 | 22.00055194 | 384.0007506 | 2570 | 42728 | 0.811144585 | 427 | Cardiac dysrhythmias |
| EPHB4 | 7:100803477:G:A | 7:100813718:T:G | 163 | 15 | 1166.015516 | 276 | 27324 | 0.575612607 | 427.11 | Paroxysmal supraventricular tachycardia |
| EPHB4 | 7:100803517:A:G | 7:100826991:C:T | 107 | 10 | 487.0043125 | 116 | 11484 | 0.014331048 | 425 | Cardiomyopathy |
| EPHB4 | 7:100803477:G:A | 7:100813718:T:G | 223 | 106.0015242 | 1840.02337 | 2570 | 42728 | 0.874998519 | 427 | Cardiac dysrhythmias |
| EPHB4 | 7:100803477:G:A | 7:100826991:C:T | 94 | 3 | 392.0015059 | 93 | 9207 | 0.871227333 | 427.5 | Arrhythmia (cardiac) NOS |
| EPHB4 | 7:100803477:G:A | 7:100813718:T:G | 219 | 65.00074662 | 1842.023167 | 1468 | 42760 | 0.90655227 | 427.2 | Atrial fibrillation and flutter |
| EPHB4 | 7:100803477:G:A | 7:100826991:C:T | 96 | 7 | 449.0035592 | 104 | 10296 | 0.338803679 | 427.4 | Cardiac arrest and ventricular fibrillation |
| EPHB4 | 7:100803477:G:A | 7:100813718:T:G | 97 | 8 | 458.0046323 | 108 | 10692 | 0.170883605 | 426.9 | Cardiac pacemaker/device in situ |
| EPHB4 | 7:100805215:C:T | 7:100826991:C:T | 81 | 5 | 181.0074413 | 78 | 7722 | 0.013016372 | 427.12 | Paroxysmal ventricular tachycardia |
| EPHB4 | 7:100803477:G:A | 7:100813718:T:G | 198 | 20 | 1520.021086 | 354 | 35046 | 0.408471063 | 427.1 | Paroxysmal tachycardia, unspecified |
| EPHB4 | 7:100803477:G:A | 7:100813718:T:G | 93 | 7 | 408.0025276 | 95 | 9405 | 0.23216555 | 426.91 | Cardiac pacemaker in situ |
| EPHB4 | 7:100803477:G:A | 7:100826991:C:T | 84 | 2 | 208.0025296 | 87 | 8613 | 0.707291513 | 427.42 | Cardiac arrest |
| EVL | 14:100084763:C:T | 14:100135969:G:A | 45 | 2.069572335 | 247.9527994 | 116 | 11484 | 0.730488693 | 425 | Cardiomyopathy |
| EVL | 14:100084709:C:T | 14:100132718:G:T | 39 | 1.052075586 | 209.9705331 | 93 | 9207 | 0.697755375 | 427.5 | Arrhythmia (cardiac) NOS |
| EVL | 14:100084709:C:T | 14:100135969:G:A | 40 | 1.053940895 | 230.5582222 | 104 | 10296 | 0.614394755 | 427.4 | Cardiac arrest and ventricular fibrillation |
| EVL | 14:100084723:G:A | 14:100135969:G:A | 84 | 3.155691426 | 792.0333122 | 354 | 35046 | 0.277282618 | 427.1 | Paroxysmal tachycardia, unspecified |
| EVL | 14:100084709:C:T | 14:100135969:G:A | 93 | 26.80064536 | 980.6944724 | 1468 | 42760 | 0.50395395 | 427.2 | Atrial fibrillation and flutter |
| EVL | 14:100084709:C:T | 14:100135969:G:A | 94 | 47.37993561 | 980.6903971 | 2570 | 42728 | 0.374343105 | 427 | Cardiac dysrhythmias |
| EVL | 14:100084763:C:T | 14:100135969:G:A | 37 | 2.045298828 | 200.9453432 | 78 | 7722 | 0.702748712 | 427.12 | Paroxysmal ventricular tachycardia |
| EVL | 14:100084723:G:A | 14:100135969:G:A | 77 | 2.101807356 | 646.372011 | 276 | 27324 | 0.271198476 | 427.11 | Paroxysmal supraventricular tachycardia |
| EVL | 14:100084723:G:A | 14:100135969:G:A | 41 | 2.04647944 | 260.1056522 | 108 | 10692 | 0.90571926 | 426.9 | Cardiac pacemaker/device in situ |
| EVL | 14:100084723:G:A | 14:100135969:G:A | 37 | 2.022613433 | 220.2909931 | 95 | 9405 | 1 | 426.91 | Cardiac pacemaker in situ |
| EVL | 14:100084709:C:T | 14:100135969:G:A | 37 | 1.052516019 | 182.4516455 | 87 | 8613 | 0.761524055 | 427.42 | Cardiac arrest |
| GAD2 | 10:26216852:G:A | 10:26245919:A:G | 43 | 8 | 552.0001852 | 108 | 10692 | 0.301242012 | 426.9 | Cardiac pacemaker/device in situ |
| GAD2 | 10:26216856:G:T | 10:26300927:T:C | 40 | 2 | 626 | 104 | 10296 | 0.11746837 | 427.4 | Cardiac arrest and ventricular fibrillation |
| GAD2 | 10:26216843:G:A | 10:26245919:A:G | 76 | 17 | 1590.000036 | 276 | 27324 | 0.012852018 | 427.11 | Paroxysmal supraventricular tachycardia |
| GAD2 | 10:26216852:G:A | 10:26300884:A:G | 45 | 9 | 623 | 116 | 11484 | 0.427240584 | 425 | Cardiomyopathy |
| GAD2 | 10:26216856:G:T | 10:26300927:T:C | 36 | 2 | 528 | 87 | 8613 | 0.210393663 | 427.42 | Cardiac arrest |
| GAD2 | 10:26216843:G:A | 10:26245919:A:G | 85 | 19 | 2030.005339 | 354 | 35046 | 0.025346652 | 427.1 | Paroxysmal tachycardia, unspecified |
| GAD2 | 10:26216856:G:T | 10:26300927:T:C | 41 | 6 | 545 | 93 | 9207 | 1 | 427.5 | Arrhythmia (cardiac) NOS |
| GAD2 | 10:26216843:G:A | 10:26245919:A:G | 93 | 146 | 2436.005365 | 2570 | 42728 | 0.75320395 | 427 | Cardiac dysrhythmias |
| GAD2 | 10:26216843:G:A | 10:26245919:A:G | 92 | 79 | 2441.005427 | 1468 | 42760 | 0.407015131 | 427.2 | Atrial fibrillation and flutter |
| GAD2 | 10:26216852:G:A | 10:26245919:A:G | 38 | 6 | 466.0001053 | 95 | 9405 | 0.687317882 | 426.91 | Cardiac pacemaker in situ |
| GAD2 | 10:26216856:G:T | 10:26300927:T:C | 33 | 3 | 407 | 78 | 7722 | 0.716397475 | 427.12 | Paroxysmal ventricular tachycardia |
| GNB3 | 12:6841310:G:A | 12:6845743:T:C | 38 | 0 | 70.00037042 | 108 | 10692 | 0.604543959 | 426.9 | Cardiac pacemaker/device in situ |
| GNB3 | 12:6841310:G:A | 12:6845778:G:A | 56 | 3.000036235 | 176.0008697 | 276 | 27324 | 0.390460635 | 427.11 | Paroxysmal supraventricular tachycardia |
| GNB3 | 12:6841310:G:A | 12:6845778:G:A | 72 | 21.00002208 | 278.0013909 | 2570 | 42728 | 0.634370408 | 427 | Cardiac dysrhythmias |
| GNB3 | 12:6841310:G:A | 12:6845743:T:C | 35 | 0 | 63.00042112 | 95 | 9405 | 0.637080725 | 426.91 | Cardiac pacemaker in situ |
| GNB3 | 12:6841310:G:A | 12:6845757:G:A | 35 | 0 | 67.00019233 | 104 | 10296 | 0.602058772 | 427.4 | Cardiac arrest and ventricular fibrillation |
| GNB3 | 12:6841310:G:A | 12:6845778:G:A | 65 | 4.00002825 | 220.0009323 | 354 | 35046 | 0.080002445 | 427.1 | Paroxysmal tachycardia, unspecified |
| GNB3 | 12:6841310:G:A | 12:6845685:G:A | 30 | 2 | 60 | 93 | 9207 | 0.058410798 | 427.5 | Arrhythmia (cardiac) NOS |
| GNB3 | 12:6841310:G:A | 12:6845778:G:A | 71 | 9 | 278.0014019 | 1468 | 42760 | 0.668458897 | 427.2 | Atrial fibrillation and flutter |
| GNB3 | 12:6841310:G:A | 12:6845757:G:A | 32 | 0 | 57.00022991 | 87 | 8613 | 0.646427668 | 427.42 | Cardiac arrest |
| GNB3 | 12:6841310:G:A | 12:6845778:G:A | 33 | 0 | 74.0005173 | 116 | 11484 | 0.59676328 | 425 | Cardiomyopathy |
| GNB3 | 12:6841310:G:A | 12:6845685:G:A | 26 | 1 | 48.00128231 | 78 | 7722 | 0.226340899 | 427.12 | Paroxysmal ventricular tachycardia |
| HIP1R | 12:122834989:T:C | 12:122848794:A:T | 157 | 4 | 511.0018968 | 116 | 11484 | 0.822584972 | 425 | Cardiomyopathy |
| HIP1R | 12:122834989:T:C | 12:122861742:G:A | 132 | 7 | 426.0006732 | 104 | 10296 | 0.120270765 | 427.4 | Cardiac arrest and ventricular fibrillation |
| HIP1R | 12:122834989:T:C | 12:122860524:G:A | 144 | 11 | 472.0016669 | 108 | 10692 | 0.004154786 | 426.9 | Cardiac pacemaker/device in situ |
| HIP1R | 12:122834978:A:C | 12:122861041:T:C | 291 | 24.00008478 | 1507.007459 | 354 | 35046 | 0.018372905 | 427.1 | Paroxysmal tachycardia, unspecified |
| HIP1R | 12:122834989:T:C | 12:122860524:G:A | 126 | 8 | 355.0012822 | 78 | 7722 | 0.028602246 | 427.12 | Paroxysmal ventricular tachycardia |
| HIP1R | 12:122834978:A:C | 12:122861041:T:C | 326 | 74.00011309 | 1880.008209 | 1468 | 42760 | 0.389130256 | 427.2 | Atrial fibrillation and flutter |
| HIP1R | 12:122834978:A:C | 12:122861041:T:C | 329 | 124.0002208 | 1878.008346 | 2570 | 42728 | 0.517505346 | 427 | Cardiac dysrhythmias |
| HIP1R | 12:122835558:G:A | 12:122861041:T:C | 131 | 6 | 402.001183 | 93 | 9207 | 0.578644371 | 427.5 | Arrhythmia (cardiac) NOS |
| HIP1R | 12:122834989:T:C | 12:122860524:G:A | 249 | 17.00003624 | 1185.006015 | 276 | 27324 | 0.028869693 | 427.11 | Paroxysmal supraventricular tachycardia |
| HIP1R | 12:122834989:T:C | 12:122861742:G:A | 117 | 6 | 374.0008047 | 87 | 8613 | 0.30051932 | 427.42 | Cardiac arrest |
| HIP1R | 12:122834989:T:C | 12:122860524:G:A | 134 | 11 | 418.0010528 | 95 | 9405 | 0.001179254 | 426.91 | Cardiac pacemaker in situ |
| HIPK2 | 7:139573084:C:G | 7:139716985:G:A | 91 | 1 | 136.0017213 | 93 | 9207 | 0.909563102 | 427.5 | Arrhythmia (cardiac) NOS |
| HIPK2 | 7:139573028:G:C | 7:139716836:C:T | 77 | 1 | 138.0025971 | 104 | 10296 | 1 | 427.4 | Cardiac arrest and ventricular fibrillation |
| HIPK2 | 7:139573088:C:T | 7:139716985:G:A | 65 | 0 | 103.0003847 | 78 | 7722 | 0.495432961 | 427.12 | Paroxysmal ventricular tachycardia |
| HIPK2 | 7:139573001:C:T | 7:139716995:C:G | 158 | 5.000181296 | 391.0081554 | 276 | 27324 | 0.631767716 | 427.11 | Paroxysmal supraventricular tachycardia |
| HIPK2 | 7:139573088:C:T | 7:139716985:G:A | 79 | 3.000092704 | 150.0028719 | 108 | 10692 | 0.085064497 | 426.9 | Cardiac pacemaker/device in situ |
| HIPK2 | 7:139573001:C:T | 7:139716995:C:G | 222 | 31.00044183 | 611.009651 | 2570 | 42728 | 0.591705984 | 427 | Cardiac dysrhythmias |
| HIPK2 | 7:139573088:C:T | 7:139716931:G:A | 74 | 3.000105396 | 136.0030544 | 95 | 9405 | 0.064989859 | 426.91 | Cardiac pacemaker in situ |
| HIPK2 | 7:139573088:C:T | 7:139716985:G:A | 85 | 1.000172563 | 170.0032775 | 116 | 11484 | 0.836283341 | 425 | Cardiomyopathy |
| HIPK2 | 7:139573001:C:T | 7:139716995:C:G | 184 | 5.000141352 | 471.0083366 | 354 | 35046 | 1 | 427.1 | Paroxysmal tachycardia, unspecified |
| HIPK2 | 7:139573028:G:C | 7:139716836:C:T | 70 | 1 | 118.00184 | 87 | 8613 | 1 | 427.42 | Cardiac arrest |
| HIPK2 | 7:139573001:C:T | 7:139716995:C:G | 219 | 20.00015839 | 611.0095905 | 1468 | 42760 | 1 | 427.2 | Atrial fibrillation and flutter |
| KCNV2 | 9:2717744:D:4 | 9:2729710:D:5 | 88 | 5.000230123 | 199.0034496 | 87 | 8613 | 0.004109755 | 427.42 | Cardiac arrest |
| KCNV2 | 9:2717744:D:4 | 9:2729721:G:C | 103 | 3 | 241.0045288 | 95 | 9405 | 0.299294535 | 426.91 | Cardiac pacemaker in situ |
| KCNV2 | 9:2717744:D:4 | 9:2729721:G:C | 100 | 5.000086214 | 255.0075066 | 116 | 11484 | 0.084056637 | 425 | Cardiomyopathy |
| KCNV2 | 9:2717741:T:C | 9:2729721:G:C | 247 | 35.00312198 | 983.0313423 | 1468 | 42760 | 0.387759271 | 427.2 | Atrial fibrillation and flutter |
| KCNV2 | 9:2717744:D:4 | 9:2729710:D:5 | 91 | 5.000192493 | 218.0033665 | 104 | 10296 | 0.004879553 | 427.4 | Cardiac arrest and ventricular fibrillation |
| KCNV2 | 9:2717741:T:C | 9:2729721:G:C | 214 | 7.000141433 | 816.0151213 | 354 | 35046 | 0.443081757 | 427.1 | Paroxysmal tachycardia, unspecified |
| KCNV2 | 9:2717741:T:C | 9:2729721:G:C | 251 | 60.00395442 | 981.0313309 | 2570 | 42728 | 0.489615459 | 427 | Cardiac dysrhythmias |
| KCNV2 | 9:2717741:T:C | 9:2729721:G:C | 184 | 6.00010896 | 640.0120732 | 276 | 27324 | 0.353386407 | 427.11 | Paroxysmal supraventricular tachycardia |
| KCNV2 | 9:2717744:D:4 | 9:2729721:G:C | 107 | 3 | 267.0043543 | 108 | 10692 | 0.364345362 | 426.9 | Cardiac pacemaker/device in situ |
| KCNV2 | 9:2717768:C:G | 9:2729721:G:C | 72 | 2.000128222 | 179.0008976 | 78 | 7722 | 0.506805933 | 427.12 | Paroxysmal ventricular tachycardia |
| KCNV2 | 9:2717780:G:A | 9:2729705:T:G | 66 | 0 | 171.0025816 | 93 | 9207 | 0.306960654 | 427.5 | Arrhythmia (cardiac) NOS |
| LAMC1 | 1:183023771:I:9 | 1:183142731:G:T | 131 | 7.000583034 | 466.0811301 | 104 | 10296 | 0.41648206 | 427.4 | Cardiac arrest and ventricular fibrillation |
| LAMC1 | 1:183023757:G:C | 1:183142731:G:T | 136 | 4.000105285 | 425.0162958 | 95 | 9405 | 0.662657971 | 426.91 | Cardiac pacemaker in situ |
| LAMC1 | 1:183023757:G:C | 1:183136586:G:T | 116 | 1 | 331.0492459 | 78 | 7722 | 0.411237978 | 427.12 | Paroxysmal ventricular tachycardia |
| LAMC1 | 1:183023741:C:G | 1:183136586:G:T | 238 | 14.00192393 | 1254.090576 | 276 | 27324 | 0.805423646 | 427.11 | Paroxysmal supraventricular tachycardia |
| LAMC1 | 1:183023751:G:T | 1:183142731:G:T | 146 | 4.00009261 | 479.0166619 | 108 | 10692 | 0.756475603 | 426.9 | Cardiac pacemaker/device in situ |
| LAMC1 | 1:183023732:C:T | 1:183136586:G:T | 311 | 77.01627502 | 1965.100491 | 1468 | 42760 | 0.530019685 | 427.2 | Atrial fibrillation and flutter |
| LAMC1 | 1:183023757:G:C | 1:183136586:G:T | 144 | 3 | 525.007421 | 116 | 11484 | 0.578357361 | 425 | Cardiomyopathy |
| LAMC1 | 1:183023732:C:T | 1:183136586:G:T | 318 | 129.0173366 | 1964.099 | 2570 | 42728 | 0.6282466 | 427 | Cardiac dysrhythmias |
| LAMC1 | 1:183023741:C:G | 1:183136586:G:T | 274 | 18.00215173 | 1591.100707 | 354 | 35046 | 0.82504672 | 427.1 | Paroxysmal tachycardia, unspecified |
| LAMC1 | 1:183023751:G:T | 1:183142707:A:G | 133 | 7.000216802 | 410.0141559 | 93 | 9207 | 0.31332578 | 427.5 | Arrhythmia (cardiac) NOS |
| LAMC1 | 1:183023771:I:9 | 1:183142731:G:T | 117 | 6.00023218 | 394.0570044 | 87 | 8613 | 0.522791513 | 427.42 | Cardiac arrest |
| LRFN4 | 11:66857759:D:3 | 11:66860184:A:T | 118 | 5.000028249 | 985.0742539 | 354 | 35046 | 0.205041869 | 427.1 | Paroxysmal tachycardia, unspecified |
| LRFN4 | 11:66857759:D:3 | 11:66860167:C:T | 45 | 1 | 221.0179533 | 78 | 7722 | 0.618005022 | 427.12 | Paroxysmal ventricular tachycardia |
| LRFN4 | 11:66857748:G:T | 11:66860172:C:T | 62 | 2 | 259.0297945 | 93 | 9207 | 0.906116062 | 427.5 | Arrhythmia (cardiac) NOS |
| LRFN4 | 11:66857748:G:T | 11:66860184:A:T | 139 | 34.00911372 | 1188.073317 | 1468 | 42760 | 0.522709237 | 427.2 | Atrial fibrillation and flutter |
| LRFN4 | 11:66857748:G:T | 11:66860172:C:T | 53 | 3 | 229.0269058 | 87 | 8613 | 0.111498216 | 427.42 | Cardiac arrest |
| LRFN4 | 11:66857759:D:3 | 11:66860176:T:C | 66 | 6 | 335.035357 | 116 | 11484 | 0.187419698 | 425 | Cardiomyopathy |
| LRFN4 | 11:66857759:D:3 | 11:66860172:C:T | 65 | 5 | 293.0273224 | 108 | 10692 | 0.186685541 | 426.9 | Cardiac pacemaker/device in situ |
| LRFN4 | 11:66857759:D:3 | 11:66860184:A:T | 101 | 3.000036233 | 779.0464576 | 276 | 27324 | 0.141306036 | 427.11 | Paroxysmal supraventricular tachycardia |
| LRFN4 | 11:66857759:D:3 | 11:66860172:C:T | 59 | 4 | 251.026324 | 95 | 9405 | 0.53655619 | 426.91 | Cardiac pacemaker in situ |
| LRFN4 | 11:66857748:G:T | 11:66860184:A:T | 141 | 62.01861881 | 1188.081801 | 2570 | 42728 | 0.475536949 | 427 | Cardiac dysrhythmias |
| LRFN4 | 11:66857748:G:T | 11:66860172:C:T | 60 | 3 | 273.0281812 | 104 | 10296 | 0.259000024 | 427.4 | Cardiac arrest and ventricular fibrillation |
| MMP2 | 16:55479479:D:2 | 16:55497063:G:A | 119 | 4 | 417.00113 | 354 | 35046 | 1 | 427.1 | Paroxysmal tachycardia, unspecified |
| MMP2 | 16:55479557:C:A | 16:55505425:G:A | 53 | 2 | 91.00076936 | 78 | 7722 | 0.049098552 | 427.12 | Paroxysmal ventricular tachycardia |
| MMP2 | 16:55479517:C:G | 16:55497063:G:A | 62 | 1 | 123.0004211 | 95 | 9405 | 1 | 426.91 | Cardiac pacemaker in situ |
| MMP2 | 16:55479511:C:T | 16:55497063:G:A | 52 | 2 | 93.00022991 | 87 | 8613 | 0.171965159 | 427.42 | Cardiac arrest |
| MMP2 | 16:55479511:C:T | 16:55497063:G:A | 58 | 2 | 116.000577 | 104 | 10296 | 0.290220206 | 427.4 | Cardiac arrest and ventricular fibrillation |
| MMP2 | 16:55479517:C:T | 16:55505425:G:A | 63 | 1 | 150 | 116 | 11484 | 0.918396248 | 425 | Cardiomyopathy |
| MMP2 | 16:55479511:C:T | 16:55505425:G:A | 50 | 1 | 111.0002151 | 93 | 9207 | 0.65131166 | 427.5 | Arrhythmia (cardiac) NOS |
| MMP2 | 16:55479479:D:2 | 16:55505425:G:A | 94 | 2 | 310.0004348 | 276 | 27324 | 0.798488774 | 427.11 | Paroxysmal supraventricular tachycardia |
| MMP2 | 16:55479517:C:G | 16:55497063:G:A | 68 | 1 | 139.0007409 | 108 | 10692 | 1 | 426.9 | Cardiac pacemaker/device in situ |
| MMP2 | 16:55479479:D:2 | 16:55498450:T:C | 139 | 26 | 503.001148 | 2570 | 42728 | 0.903580384 | 427 | Cardiac dysrhythmias |
| MMP2 | 16:55479479:D:2 | 16:55498450:T:C | 138 | 14 | 503.0011758 | 1468 | 42760 | 0.926844596 | 427.2 | Atrial fibrillation and flutter |
| NOS1 | 12:117218085:G:A | 12:117278099:C:G | 207 | 17 | 2185.00058 | 276 | 27324 | 0.477040732 | 427.11 | Paroxysmal supraventricular tachycardia |
| NOS1 | 12:117218074:T:C | 12:117331055:C:T | 114 | 4 | 744.0002105 | 95 | 9405 | 0.333894265 | 426.91 | Cardiac pacemaker in situ |
| NOS1 | 12:117218074:T:C | 12:117215326:T:C | 126 | 7 | 814.0001923 | 104 | 10296 | 0.824999749 | 427.4 | Cardiac arrest and ventricular fibrillation |
| NOS1 | 12:117218100:C:T | 12:117278099:C:G | 118 | 5 | 742 | 93 | 9207 | 0.538226373 | 427.5 | Arrhythmia (cardiac) NOS |
| NOS1 | 12:117218074:T:C | 12:117278099:C:G | 242 | 29 | 2776.000819 | 354 | 35046 | 0.836603438 | 427.1 | Paroxysmal tachycardia, unspecified |
| NOS1 | 12:117218074:T:C | 12:117331055:C:T | 121 | 4 | 828.0001852 | 108 | 10692 | 0.215375652 | 426.9 | Cardiac pacemaker/device in situ |
| NOS1 | 12:117218074:T:C | 12:117278099:C:G | 272 | 143.0001131 | 3398.000882 | 1468 | 42760 | 0.011836453 | 427.2 | Atrial fibrillation and flutter |
| NOS1 | 12:117218074:T:C | 12:117278099:C:G | 275 | 233.0001325 | 3397.000861 | 2570 | 42728 | 0.103431839 | 427 | Cardiac dysrhythmias |
| NOS1 | 12:117218074:T:C | 12:117215326:T:C | 119 | 6 | 687.0002299 | 87 | 8613 | 0.882964534 | 427.42 | Cardiac arrest |
| NOS1 | 12:117218101:G:A | 12:117331055:C:T | 112 | 6 | 920 | 116 | 11484 | 0.325345952 | 425 | Cardiomyopathy |
| NOS1 | 12:117218074:T:C | 12:117226772:T:C | 104 | 10 | 624.0005129 | 78 | 7722 | 0.278360337 | 427.12 | Paroxysmal ventricular tachycardia |
| PIK3CB | 3:138655414:G:A | 3:138759264:T:G | 54 | 1 | 84.00010527 | 95 | 9405 | 0.677545317 | 426.91 | Cardiac pacemaker in situ |
| PIK3CB | 3:138655414:G:A | 3:138759288:G:A | 113 | 5.000028251 | 272.0018928 | 354 | 35046 | 0.218502227 | 427.1 | Paroxysmal tachycardia, unspecified |
| PIK3CB | 3:138655414:G:A | 3:138759264:T:G | 56 | 1 | 91.0000926 | 108 | 10692 | 0.720351976 | 426.9 | Cardiac pacemaker/device in situ |
| PIK3CB | 3:138655414:G:A | 3:138759288:G:A | 95 | 5 | 213.0013044 | 276 | 27324 | 0.039154327 | 427.11 | Paroxysmal supraventricular tachycardia |
| PIK3CB | 3:138655414:G:A | 3:138734631:I:1 | 138 | 8.000067837 | 341.0019446 | 1468 | 42760 | 0.511869708 | 427.2 | Atrial fibrillation and flutter |
| PIK3CB | 3:138655414:G:A | 3:138734631:I:1 | 63 | 0 | 95.00183942 | 87 | 8613 | 0.620830102 | 427.42 | Cardiac arrest |
| PIK3CB | 3:138655414:G:A | 3:138759294:A:G | 68 | 1 | 110 | 116 | 11484 | 0.886273706 | 425 | Cardiomyopathy |
| PIK3CB | 3:138655414:G:A | 3:138734631:I:1 | 70 | 0 | 111.0020196 | 104 | 10296 | 0.567918167 | 427.4 | Cardiac arrest and ventricular fibrillation |
| PIK3CB | 3:138655502:C:T | 3:138759288:G:A | 46 | 0 | 67.00038466 | 78 | 7722 | 0.644983584 | 427.12 | Paroxysmal ventricular tachycardia |
| PIK3CB | 3:138655414:G:A | 3:138734631:I:1 | 139 | 20.00011039 | 340.001987 | 2570 | 42728 | 1 | 427 | Cardiac dysrhythmias |
| PIK3CB | 3:138657690:C:T | 3:138759288:G:A | 46 | 0 | 68 | 93 | 9207 | 0.609978132 | 427.5 | Arrhythmia (cardiac) NOS |
| PPFIA1 | 11:70272251:C:G | 11:70382137:C:G | 73 | 3 | 137.006547 | 78 | 7722 | 0.040036719 | 427.12 | Paroxysmal ventricular tachycardia |
| PPFIA1 | 11:70272180:G:C | 11:70382137:C:G | 111 | 2.00009261 | 218.0095509 | 108 | 10692 | 0.66030763 | 426.9 | Cardiac pacemaker/device in situ |
| PPFIA1 | 11:70272180:G:C | 11:70382137:C:G | 222 | 43.0007963 | 803.0274963 | 2570 | 42728 | 0.782979383 | 427 | Cardiac dysrhythmias |
| PPFIA1 | 11:70272251:C:G | 11:70382137:C:G | 94 | 4 | 180.0078289 | 87 | 8613 | 0.070978109 | 427.42 | Cardiac arrest |
| PPFIA1 | 11:70272251:C:G | 11:70382137:C:G | 192 | 8.000169756 | 644.0228253 | 354 | 35046 | 0.613058384 | 427.1 | Paroxysmal tachycardia, unspecified |
| PPFIA1 | 11:70272180:G:C | 11:70382137:C:G | 102 | 2.000105285 | 189.0087483 | 95 | 9405 | 0.458525949 | 426.91 | Cardiac pacemaker in situ |
| PPFIA1 | 11:70272213:G:C | 11:70382135:I:1 | 101 | 4 | 226.0076808 | 116 | 11484 | 0.140422372 | 425 | Cardiomyopathy |
| PPFIA1 | 11:70272213:G:C | 11:70382122:D:2 | 83 | 0 | 185.0085094 | 93 | 9207 | 0.291694244 | 427.5 | Arrhythmia (cardiac) NOS |
| PPFIA1 | 11:70272251:C:G | 11:70382135:I:1 | 169 | 5.000072581 | 502.009467 | 276 | 27324 | 0.934983206 | 427.11 | Paroxysmal supraventricular tachycardia |
| PPFIA1 | 11:70272251:C:G | 11:70382137:C:G | 100 | 4 | 212.0147373 | 104 | 10296 | 0.150102537 | 427.4 | Cardiac arrest and ventricular fibrillation |
| PPFIA1 | 11:70272180:G:C | 11:70382137:C:G | 221 | 23.00058921 | 804.0278907 | 1468 | 42760 | 0.679653172 | 427.2 | Atrial fibrillation and flutter |
| PRKAG2 | 7:151557224:G:A | 7:151675419:C:T | 38 | 2 | 51 | 78 | 7722 | 0.01452269 | 427.12 | Paroxysmal ventricular tachycardia |
| PRKAG2 | 7:151557218:C:G | 7:151675419:C:T | 40 | 1 | 57.00010754 | 93 | 9207 | 0.260307453 | 427.5 | Arrhythmia (cardiac) NOS |
| PRKAG2 | 7:151557224:G:A | 7:151675419:C:T | 39 | 0 | 65.00042123 | 95 | 9405 | 0.632599908 | 426.91 | Cardiac pacemaker in situ |
| PRKAG2 | 7:151557224:G:A | 7:151595452:C:T | 36 | 4 | 52.00138026 | 87 | 8613 | 0.000186805 | 427.42 | Cardiac arrest |
| PRKAG2 | 7:151557208:G:A | 7:151675419:C:T | 87 | 1 | 189.0009423 | 276 | 27324 | 0.743407082 | 427.11 | Paroxysmal supraventricular tachycardia |
| PRKAG2 | 7:151557224:G:A | 7:151595452:C:T | 41 | 4 | 62.00115451 | 104 | 10296 | 0.000212168 | 427.4 | Cardiac arrest and ventricular fibrillation |
| PRKAG2 | 7:151557218:C:G | 7:151675419:C:G | 50 | 0 | 85.00034487 | 116 | 11484 | 0.572090974 | 425 | Cardiomyopathy |
| PRKAG2 | 7:151557224:G:A | 7:151675419:C:T | 42 | 0 | 73.00037051 | 108 | 10692 | 0.602085582 | 426.9 | Cardiac pacemaker/device in situ |
| PRKAG2 | 7:151557208:G:A | 7:151675419:C:G | 111 | 14 | 307.0028049 | 2570 | 42728 | 0.451056964 | 427 | Cardiac dysrhythmias |
| PRKAG2 | 7:151557208:G:A | 7:151675419:C:G | 109 | 7 | 307.0028728 | 1468 | 42760 | 0.395956587 | 427.2 | Atrial fibrillation and flutter |
| PRKAG2 | 7:151557208:G:A | 7:151675419:C:G | 99 | 3 | 236.0012717 | 354 | 35046 | 0.620793688 | 427.1 | Paroxysmal tachycardia, unspecified |
| PSMB11 | 14:23042230:C:T | 14:23043053:D:4 | 52 | 3 | 296 | 78 | 7722 | 0.39364808 | 427.12 | Paroxysmal ventricular tachycardia |
| PSMB11 | 14:23042230:C:T | 14:23043121:C:T | 98 | 13 | 1402.020453 | 354 | 35046 | 0.581829458 | 427.1 | Paroxysmal tachycardia, unspecified |
| PSMB11 | 14:23042230:C:T | 14:23043121:C:T | 87 | 9 | 1064.019857 | 276 | 27324 | 0.749624496 | 427.11 | Paroxysmal supraventricular tachycardia |
| PSMB11 | 14:23042293:G:A | 14:23043121:C:T | 50 | 5 | 394.011184 | 93 | 9207 | 0.204887164 | 427.5 | Arrhythmia (cardiac) NOS |
| PSMB11 | 14:23042230:C:T | 14:23043093:G:C | 62 | 6.000288489 | 434.011155 | 104 | 10296 | 0.688700925 | 427.4 | Cardiac arrest and ventricular fibrillation |
| PSMB11 | 14:23042230:C:T | 14:23043121:C:T | 108 | 103.0004415 | 1735.021371 | 2570 | 42728 | 0.271395824 | 427 | Cardiac dysrhythmias |
| PSMB11 | 14:23042230:C:T | 14:23043093:G:C | 59 | 6.000344867 | 367.0109209 | 87 | 8613 | 0.391594376 | 427.42 | Cardiac arrest |
| PSMB11 | 14:23042230:C:T | 14:23043121:C:T | 62 | 7 | 464.0091387 | 116 | 11484 | 0.413283586 | 425 | Cardiomyopathy |
| PSMB11 | 14:23042230:C:T | 14:23043121:C:T | 57 | 6 | 445.0213002 | 108 | 10692 | 0.751300339 | 426.9 | Cardiac pacemaker/device in situ |
| PSMB11 | 14:23042230:C:T | 14:23043121:C:T | 107 | 63 | 1735.021209 | 1468 | 42760 | 0.871141025 | 427.2 | Atrial fibrillation and flutter |
| PSMB11 | 14:23042230:C:T | 14:23043121:C:T | 54 | 4 | 391.021057 | 95 | 9405 | 1 | 426.91 | Cardiac pacemaker in situ |
| ROBO1 | 3:78600143:T:A | 3:78598929:T:C | 143 | 2 | 463.0078578 | 116 | 11484 | 0.364401111 | 425 | Cardiomyopathy |
| ROBO1 | 3:78600135:D:3 | 3:79589835:T:G | 142 | 2 | 426.0051988 | 104 | 10296 | 0.397963801 | 427.4 | Cardiac arrest and ventricular fibrillation |
| ROBO1 | 3:78600135:D:3 | 3:79589824:C:T | 125 | 2 | 356.0052941 | 87 | 8613 | 0.564990356 | 427.42 | Cardiac arrest |
| ROBO1 | 3:78600114:T:A | 3:78598929:T:C | 129 | 1 | 385.0005264 | 95 | 9405 | 0.248676027 | 426.91 | Cardiac pacemaker in situ |
| ROBO1 | 3:78600114:T:A | 3:79018406:C:T | 344 | 56.00423183 | 1702.0221 | 1468 | 42760 | 0.002524084 | 427.2 | Atrial fibrillation and flutter |
| ROBO1 | 3:78600114:T:A | 3:79018406:C:T | 350 | 92.00777662 | 1700.024367 | 2570 | 42728 | 0.018131181 | 427 | Cardiac dysrhythmias |
| ROBO1 | 3:78600114:T:A | 3:78598929:T:C | 118 | 8.00010784 | 344.0061376 | 93 | 9207 | 0.038172951 | 427.5 | Arrhythmia (cardiac) NOS |
| ROBO1 | 3:78600114:T:A | 3:79018406:C:T | 254 | 11.00231917 | 1076.007981 | 276 | 27324 | 0.613585868 | 427.11 | Paroxysmal supraventricular tachycardia |
| ROBO1 | 3:78600114:T:A | 3:78598929:T:C | 138 | 2 | 438.000463 | 108 | 10692 | 0.422671355 | 426.9 | Cardiac pacemaker/device in situ |
| ROBO1 | 3:78600143:T:A | 3:78717274:C:T | 109 | 2.001923324 | 300.0021832 | 78 | 7722 | 0.777797796 | 427.12 | Paroxysmal ventricular tachycardia |
| ROBO1 | 3:78600114:T:A | 3:79018406:C:T | 295 | 14.0052549 | 1379.012308 | 354 | 35046 | 0.763516577 | 427.1 | Paroxysmal tachycardia, unspecified |
| RYR2 | 1:237270524:G:A | 1:237832627:T:A | 216 | 9.015334633 | 724.5959184 | 78 | 7722 | 0.770506854 | 427.12 | Paroxysmal ventricular tachycardia |
| RYR2 | 1:237270591:T:G | 1:237784973:I:4 | 273 | 8.013623978 | 956.6812539 | 104 | 10296 | 0.806168221 | 427.4 | Cardiac arrest and ventricular fibrillation |
| RYR2 | 1:237270516:A:G | 1:237784973:I:4 | 252 | 3 | 827.6597803 | 95 | 9405 | 0.135851078 | 426.91 | Cardiac pacemaker in situ |
| RYR2 | 1:237270516:A:G | 1:237706947:A:G | 307 | 14.02725871 | 1067.107363 | 116 | 11484 | 0.198715582 | 425 | Cardiomyopathy |
| RYR2 | 1:237270516:A:G | 1:237784973:I:4 | 271 | 5 | 952.908639 | 108 | 10692 | 0.276182447 | 426.9 | Cardiac pacemaker/device in situ |
| RYR2 | 1:237270591:T:G | 1:237784973:I:4 | 246 | 6 | 802.3908379 | 87 | 8613 | 0.73001129 | 427.42 | Cardiac arrest |
| RYR2 | 1:237042532:G:C | 1:237786037:G:A | 697 | 233.3558997 | 3968.02165 | 2570 | 42728 | 0.91693182 | 427 | Cardiac dysrhythmias |
| RYR2 | 1:237042532:G:C | 1:237786037:G:A | 679 | 119.1706674 | 3973.011391 | 1468 | 42760 | 0.365598983 | 427.2 | Atrial fibrillation and flutter |
| RYR2 | 1:237042532:G:C | 1:237786037:G:A | 492 | 25.04140095 | 2571.028333 | 276 | 27324 | 1 | 427.11 | Paroxysmal supraventricular tachycardia |
| RYR2 | 1:237042532:G:C | 1:237832627:T:A | 228 | 8.012022462 | 817.6070774 | 93 | 9207 | 0.467076704 | 427.5 | Arrhythmia (cardiac) NOS |
| RYR2 | 1:237042532:G:C | 1:237786037:G:A | 578 | 32.04008895 | 3255.028278 | 354 | 35046 | 1 | 427.1 | Paroxysmal tachycardia, unspecified |
| SCN1A | 2:165991287:T:G | 2:166041470:C:A | 82 | 2 | 304.0177197 | 108 | 10692 | 0.809419854 | 426.9 | Cardiac pacemaker/device in situ |
| SCN1A | 2:165991287:T:G | 2:166041470:C:A | 206 | 80.00170563 | 1179.073621 | 2570 | 42728 | 0.479649118 | 427 | Cardiac dysrhythmias |
| SCN1A | 2:165991287:T:G | 2:166041470:C:A | 86 | 6.000259673 | 302.0244481 | 116 | 11484 | 0.109570046 | 425 | Cardiomyopathy |
| SCN1A | 2:165991287:T:G | 2:166041470:C:A | 205 | 53.00111161 | 1179.074702 | 1468 | 42760 | 0.111896725 | 427.2 | Atrial fibrillation and flutter |
| SCN1A | 2:165991287:T:G | 2:166041470:C:A | 85 | 6 | 274.0166662 | 104 | 10296 | 0.000325345 | 427.4 | Cardiac arrest and ventricular fibrillation |
| SCN1A | 2:165991287:T:G | 2:166041470:C:A | 172 | 8.000198446 | 957.0585786 | 354 | 35046 | 0.82370864 | 427.1 | Paroxysmal tachycardia, unspecified |
| SCN1A | 2:165991287:T:G | 2:166041470:C:A | 69 | 6 | 225.0162363 | 87 | 8613 | 0.000113857 | 427.42 | Cardiac arrest |
| SCN1A | 2:165991405:T:C | 2:166041470:C:A | 60 | 5 | 254.0093725 | 93 | 9207 | 0.10478776 | 427.5 | Arrhythmia (cardiac) NOS |
| SCN1A | 2:165991287:T:G | 2:166041470:C:A | 58 | 3 | 205.008993 | 78 | 7722 | 0.478189234 | 427.12 | Paroxysmal ventricular tachycardia |
| SCN1A | 2:165991287:T:G | 2:166041470:C:A | 151 | 6.000254543 | 764.0593112 | 276 | 27324 | 0.783529432 | 427.11 | Paroxysmal supraventricular tachycardia |
| SCN1A | 2:165991287:T:G | 2:166041470:C:A | 70 | 2 | 265.0168761 | 95 | 9405 | 0.901194745 | 426.91 | Cardiac pacemaker in situ |
| SFTPA2 | 10:79557231:C:T | 10:79559311:C:G | 35 | 0 | 168.0095751 | 116 | 11484 | 0.336651897 | 425 | Cardiomyopathy |
| SFTPA2 | 10:79557231:C:T | 10:79559511:C:A | 29 | 0.132621951 | 141.0712335 | 93 | 9207 | 0.401549394 | 427.5 | Arrhythmia (cardiac) NOS |
| SFTPA2 | 10:79557231:C:T | 10:79559311:C:G | 30 | 0 | 125.0144953 | 87 | 8613 | 0.4142178 | 427.42 | Cardiac arrest |
| SFTPA2 | 10:79557231:C:T | 10:79559511:C:A | 61 | 3.388278928 | 572.5515518 | 354 | 35046 | 0.460091181 | 427.1 | Paroxysmal tachycardia, unspecified |
| SFTPA2 | 10:79557319:G:C | 10:79559506:C:T | 30 | 1.000257069 | 129.022467 | 78 | 7722 | 0.890675182 | 427.12 | Paroxysmal ventricular tachycardia |
| SFTPA2 | 10:79557231:C:T | 10:79559511:C:A | 57 | 2.388074785 | 448.4869589 | 276 | 27324 | 0.460540004 | 427.11 | Paroxysmal supraventricular tachycardia |
| SFTPA2 | 10:79557231:C:T | 10:79559511:C:A | 35 | 2.134387352 | 171.1171016 | 108 | 10692 | 0.642916216 | 426.9 | Cardiac pacemaker/device in situ |
| SFTPA2 | 10:79557231:C:T | 10:79559311:C:G | 30 | 0 | 147.012894 | 104 | 10296 | 0.366981324 | 427.4 | Cardiac arrest and ventricular fibrillation |
| SFTPA2 | 10:79557231:C:T | 10:79559511:C:A | 68 | 22.55342273 | 709.1572737 | 1468 | 42760 | 0.877038613 | 427.2 | Atrial fibrillation and flutter |
| SFTPA2 | 10:79557231:C:T | 10:79559511:C:A | 32 | 2.134036145 | 156.1927881 | 95 | 9405 | 0.561351231 | 426.91 | Cardiac pacemaker in situ |
| SFTPA2 | 10:79557231:C:T | 10:79559511:C:A | 69 | 37.35800811 | 707.3188366 | 2570 | 42728 | 0.564986251 | 427 | Cardiac dysrhythmias |
| SLC12A4 | 16:67944865:C:T | 16:67966752:C:A | 113 | 2.038806852 | 411.231229 | 95 | 9405 | 0.483003656 | 426.91 | Cardiac pacemaker in situ |
| SLC12A4 | 16:67944851:T:C | 16:67966752:C:A | 260 | 9.093160331 | 1492.900441 | 354 | 35046 | 0.238660956 | 427.1 | Paroxysmal tachycardia, unspecified |
| SLC12A4 | 16:67944892:C:T | 16:67968652:C:T | 105 | 1.021432905 | 334.5128889 | 78 | 7722 | 0.330194105 | 427.12 | Paroxysmal ventricular tachycardia |
| SLC12A4 | 16:67944865:C:T | 16:67945950:C:T | 105 | 7.033624748 | 421.510557 | 93 | 9207 | 0.136342702 | 427.5 | Arrhythmia (cardiac) NOS |
| SLC12A4 | 16:67944865:C:T | 16:67966752:C:A | 125 | 2.045240651 | 469.6053767 | 108 | 10692 | 0.373562616 | 426.9 | Cardiac pacemaker/device in situ |
| SLC12A4 | 16:67944875:C:T | 16:67946936:C:T | 141 | 3.042040066 | 506.6259937 | 116 | 11484 | 0.523700656 | 425 | Cardiomyopathy |
| SLC12A4 | 16:67944851:T:C | 16:67966752:C:A | 296 | 110.565314 | 1806.757669 | 2570 | 42728 | 1 | 427 | Cardiac dysrhythmias |
| SLC12A4 | 16:67944851:T:C | 16:67966752:C:A | 292 | 67.31214538 | 1807.851563 | 1468 | 42760 | 0.689548287 | 427.2 | Atrial fibrillation and flutter |
| SLC12A4 | 16:67944892:C:T | 16:67968652:C:T | 100 | 1.033377041 | 373.0429048 | 87 | 8613 | 0.308219268 | 427.42 | Cardiac arrest |
| SLC12A4 | 16:67944892:C:T | 16:67968652:C:T | 112 | 2.031932941 | 438.3887763 | 104 | 10296 | 0.465985326 | 427.4 | Cardiac arrest and ventricular fibrillation |
| SLC12A4 | 16:67944851:T:C | 16:67966752:C:A | 220 | 8.061042377 | 1167.916589 | 276 | 27324 | 0.487180032 | 427.11 | Paroxysmal supraventricular tachycardia |
| SLC26A4 | 7:107661643:T:C | 7:107715421:A:G | 77 | 3 | 431.0030803 | 78 | 7722 | 0.799202947 | 427.12 | Paroxysmal ventricular tachycardia |
| SLC26A4 | 7:107661643:T:C | 7:107698112:G:A | 104 | 4 | 657.006044 | 116 | 11484 | 0.543902654 | 425 | Cardiomyopathy |
| SLC26A4 | 7:107661643:T:C | 7:107715421:A:G | 158 | 12.0005435 | 1527.012302 | 276 | 27324 | 0.599292653 | 427.11 | Paroxysmal supraventricular tachycardia |
| SLC26A4 | 7:107661643:T:C | 7:107715421:A:G | 86 | 7 | 524.0016134 | 93 | 9207 | 0.187636008 | 427.5 | Arrhythmia (cardiac) NOS |
| SLC26A4 | 7:107661643:T:C | 7:107715421:A:G | 80 | 6 | 481.0034543 | 87 | 8613 | 0.83139599 | 427.42 | Cardiac arrest |
| SLC26A4 | 7:107661643:T:C | 7:107683538:G:A | 96 | 4 | 624.0033377 | 108 | 10692 | 0.577395718 | 426.9 | Cardiac pacemaker/device in situ |
| SLC26A4 | 7:107661643:T:C | 7:107715421:A:G | 98 | 8 | 586.0039493 | 104 | 10296 | 0.571804777 | 427.4 | Cardiac arrest and ventricular fibrillation |
| SLC26A4 | 7:107661643:T:C | 7:107715421:A:G | 199 | 148.0013034 | 2479.014456 | 2570 | 42728 | 0.670926018 | 427 | Cardiac dysrhythmias |
| SLC26A4 | 7:107661643:T:C | 7:107683538:G:A | 91 | 2 | 560.0028458 | 95 | 9405 | 0.28936875 | 426.91 | Cardiac pacemaker in situ |
| SLC26A4 | 7:107661643:T:C | 7:107715421:A:G | 173 | 14.00050849 | 2009.013831 | 354 | 35046 | 0.310547175 | 427.1 | Paroxysmal tachycardia, unspecified |
| SLC26A4 | 7:107661643:T:C | 7:107715421:A:G | 197 | 73.00113121 | 2485.014761 | 1468 | 42760 | 0.22104756 | 427.2 | Atrial fibrillation and flutter |
| SLC9B1 | 4:102901162:A:T | 4:102991714:T:C | 89 | 199.0000663 | 3136.040048 | 2570 | 42728 | 0.838055625 | 427 | Cardiac dysrhythmias |
| SLC9B1 | 4:102901190:C:T | 4:102989798:A:G | 48 | 9 | 714.0178525 | 93 | 9207 | 0.711612447 | 427.5 | Arrhythmia (cardiac) NOS |
| SLC9B1 | 4:102901162:A:T | 4:102991714:T:C | 76 | 25.00002827 | 2504.003984 | 354 | 35046 | 0.763343457 | 427.1 | Paroxysmal tachycardia, unspecified |
| SLC9B1 | 4:102901162:A:T | 4:102991714:T:C | 47 | 17 | 736.0145215 | 104 | 10296 | 0.028371104 | 427.4 | Cardiac arrest and ventricular fibrillation |
| SLC9B1 | 4:102901190:C:T | 4:102949256:C:A | 45 | 13 | 802.022933 | 116 | 11484 | 0.123583733 | 425 | Cardiomyopathy |
| SLC9B1 | 4:102901162:A:T | 4:102991714:T:C | 45 | 12 | 629.0143704 | 87 | 8613 | 0.175157262 | 427.42 | Cardiac arrest |
| SLC9B1 | 4:102901162:A:T | 4:102991714:T:C | 71 | 22.00003627 | 1942.004168 | 276 | 27324 | 0.560571103 | 427.11 | Paroxysmal supraventricular tachycardia |
| SLC9B1 | 4:102901190:C:T | 4:102991714:T:C | 47 | 10 | 686.0210548 | 95 | 9405 | 0.513269544 | 426.91 | Cardiac pacemaker in situ |
| SLC9B1 | 4:102901190:C:T | 4:102991714:T:C | 50 | 10 | 792.0221319 | 108 | 10692 | 0.639209469 | 426.9 | Cardiac pacemaker/device in situ |
| SLC9B1 | 4:102901162:A:T | 4:102991714:T:C | 87 | 113.0000226 | 3138.040135 | 1468 | 42760 | 0.890743989 | 427.2 | Atrial fibrillation and flutter |
| SLC9B1 | 4:102901196:A:C | 4:102911430:C:T | 41 | 5 | 518.0007693 | 78 | 7722 | 0.401838289 | 427.12 | Paroxysmal ventricular tachycardia |
| SYT10 | 12:33376853:G:C | 12:33439521:A:C | 37 | 1 | 80.00387252 | 93 | 9207 | 0.438870339 | 427.5 | Arrhythmia (cardiac) NOS |
| SYT10 | 12:33376853:G:C | 12:33376901:D:1 | 36 | 0 | 79.00805119 | 87 | 8613 | 0.557343148 | 427.42 | Cardiac arrest |
| SYT10 | 12:33376837:G:A | 12:33379963:T:G | 104 | 23.00061832 | 341.0084359 | 2570 | 42728 | 0.674387 | 427 | Cardiac dysrhythmias |
| SYT10 | 12:33376837:G:A | 12:33379963:T:G | 103 | 16.0002036 | 341.0082554 | 1468 | 42760 | 0.404402411 | 427.2 | Atrial fibrillation and flutter |
| SYT10 | 12:33376853:G:C | 12:33376901:D:1 | 38 | 0 | 90.00692698 | 104 | 10296 | 0.522011075 | 427.4 | Cardiac arrest and ventricular fibrillation |
| SYT10 | 12:33376837:G:A | 12:33379963:T:G | 86 | 4.000395558 | 281.0077992 | 354 | 35046 | 0.349654002 | 427.1 | Paroxysmal tachycardia, unspecified |
| SYT10 | 12:33376847:G:T | 12:33379963:T:G | 40 | 0 | 80.00273859 | 95 | 9405 | 0.585736655 | 426.91 | Cardiac pacemaker in situ |
| SYT10 | 12:33376837:G:A | 12:33439521:A:G | 46 | 3 | 97.00379522 | 116 | 11484 | 0.010416075 | 425 | Cardiomyopathy |
| SYT10 | 12:33376837:G:A | 12:33379963:T:G | 75 | 3.0004711 | 217.0067777 | 276 | 27324 | 0.464726217 | 427.11 | Paroxysmal supraventricular tachycardia |
| SYT10 | 12:33376853:G:C | 12:33376901:D:1 | 35 | 1 | 57.00076943 | 78 | 7722 | 0.196597786 | 427.12 | Paroxysmal ventricular tachycardia |
| SYT10 | 12:33376847:G:T | 12:33379963:T:G | 42 | 0 | 86.00389095 | 108 | 10692 | 0.563240901 | 426.9 | Cardiac pacemaker/device in situ |
| TCF7L1 | 2:85133688:C:A | 2:85309442:G:A | 125 | 154.8709492 | 2536.388328 | 2570 | 42728 | 0.873436509 | 427 | Cardiac dysrhythmias |
| TCF7L1 | 2:85133696:I:9 | 2:85309442:G:A | 91 | 16.25263952 | 1594.707436 | 276 | 27324 | 1 | 427.11 | Paroxysmal supraventricular tachycardia |
| TCF7L1 | 2:85133705:D:6 | 2:85309400:C:T | 56 | 8.096635114 | 607.3040906 | 108 | 10692 | 0.538887189 | 426.9 | Cardiac pacemaker/device in situ |
| TCF7L1 | 2:85133696:I:9 | 2:85309442:G:A | 103 | 18.31401619 | 2034.349305 | 354 | 35046 | 0.865003171 | 427.1 | Paroxysmal tachycardia, unspecified |
| TCF7L1 | 2:85133698:G:A | 2:85309400:C:T | 67 | 6.094516313 | 666.6126965 | 116 | 11484 | 1 | 425 | Cardiomyopathy |
| TCF7L1 | 2:85133696:I:9 | 2:85309400:C:T | 61 | 7.074550429 | 591.73611 | 104 | 10296 | 0.837789328 | 427.4 | Cardiac arrest and ventricular fibrillation |
| TCF7L1 | 2:85133705:D:6 | 2:85309400:C:T | 59 | 2.164117409 | 724.581586 | 93 | 9207 | 0.2040254 | 427.5 | Arrhythmia (cardiac) NOS |
| TCF7L1 | 2:85133696:I:9 | 2:85309400:C:T | 55 | 6.057176449 | 491.6620248 | 87 | 8613 | 0.789162723 | 427.42 | Cardiac arrest |
| TCF7L1 | 2:85133688:C:A | 2:85309442:G:A | 122 | 86.69998358 | 2537.432472 | 1468 | 42760 | 1 | 427.2 | Atrial fibrillation and flutter |
| TCF7L1 | 2:85133705:I:3 | 2:85309400:C:T | 51 | 5.058465878 | 537.3728312 | 95 | 9405 | 1 | 426.91 | Cardiac pacemaker in situ |
| TCF7L1 | 2:85133705:D:6 | 2:85309229:C:T | 42 | 2.040940103 | 483.6903677 | 78 | 7722 | 0.408163191 | 427.12 | Paroxysmal ventricular tachycardia |
| TSPOAP1 | 17:58304375:A:G | 17:58323546:C:T | 318 | 15.00138532 | 1906.143757 | 276 | 27324 | 0.488840833 | 427.11 | Paroxysmal supraventricular tachycardia |
| TSPOAP1 | 17:58304375:A:G | 17:58323546:C:T | 411 | 163.0162447 | 2934.275814 | 2570 | 42728 | 0.446555101 | 427 | Cardiac dysrhythmias |
| TSPOAP1 | 17:58304375:A:G | 17:58323546:C:T | 352 | 17.00148917 | 2375.156703 | 354 | 35046 | 0.247794392 | 427.1 | Paroxysmal tachycardia, unspecified |
| TSPOAP1 | 17:58304375:A:G | 17:58323546:C:T | 407 | 104.0104954 | 2934.281596 | 1468 | 42760 | 0.928145241 | 427.2 | Atrial fibrillation and flutter |
| TSPOAP1 | 17:58304377:T:C | 17:58305867:T:C | 156 | 5.000107759 | 638.0109768 | 93 | 9207 | 0.796369805 | 427.5 | Arrhythmia (cardiac) NOS |
| TSPOAP1 | 17:58304385:D:3 | 17:58327896:G:A | 160 | 5 | 602.0073612 | 87 | 8613 | 0.779216497 | 427.42 | Cardiac arrest |
| TSPOAP1 | 17:58304375:A:G | 17:58323546:C:T | 196 | 4 | 757.0031488 | 108 | 10692 | 0.27415253 | 426.9 | Cardiac pacemaker/device in situ |
| TSPOAP1 | 17:58304375:A:G | 17:58323546:C:T | 183 | 3 | 665.0027375 | 95 | 9405 | 0.229447642 | 426.91 | Cardiac pacemaker in situ |
| TSPOAP1 | 17:58304377:T:C | 17:58305867:T:C | 157 | 3 | 516.0083364 | 78 | 7722 | 0.543794693 | 427.12 | Paroxysmal ventricular tachycardia |
| TSPOAP1 | 17:58304385:D:3 | 17:58327896:G:A | 176 | 6 | 706.0076005 | 104 | 10296 | 0.778578629 | 427.4 | Cardiac arrest and ventricular fibrillation |
| TSPOAP1 | 17:58304375:A:G | 17:58323546:C:T | 189 | 5.003494806 | 735.2531109 | 116 | 11484 | 0.58893874 | 425 | Cardiomyopathy |

*Note: MAC, minor allele count.*
