# Supplementary material for: Identification of potential candidate genes and pathways in atrioventricular nodal reentry tachycardia by whole‐exome sequencing
Source: Clin Transl Med. 2020 Apr 30;10(1):238–57. doi: 10.1002/ctm2.25 (PMC7240861; doi:10.1002/ctm2.25)
Supplement: Supplementary file 10 — Supporting Information S9 [file CTM2-10-238-s002.docx]

**S13: Enrichment Analysis for Phenotype Category in UK Biobank**

| **Phenotype category name** | **Phenotype code** | **Burden**  **input numbers** | **Non-burden input numbers** | **Burden**  **background numbers** | **Non-burden**  **background numbers** | ***P* value** |
| --- | --- | --- | --- | --- | --- | --- |
| Paroxysmal supraventricular tachycardia | 427.11 | 3 | 0 | 987 | 16772 | 0.000 |
| Other hypertrophic and atrophic conditions of skin | 701 | 2 | 1 | 872 | 16918 | 0.007 |
| Bacterial pneumonia | 480.1 | 2 | 1 | 943 | 16846 | 0.008 |
| Paroxysmal tachycardia, unspecified | 427.1 | 2 | 1 | 996 | 16780 | 0.009 |
| Opiates and related narcotics causing adverse effects in therapeutic use | 965.1 | 2 | 1 | 1077 | 16610 | 0.011 |
| Nephritis; nephrosis; renal sclerosis | 580 | 2 | 1 | 1082 | 16617 | 0.011 |
| Aneurysm and dissection of heart | 411.41 | 2 | 1 | 1121 | 16525 | 0.012 |
| Intestinal infection due to C. difficile | 008.52 | 2 | 1 | 1212 | 16325 | 0.014 |
| Anaphylactic shock NOS | 946 | 2 | 1 | 1239 | 16380 | 0.014 |
| Ovarian cyst | 628 | 1 | 2 | 857 | 16891 | 0.138 |
| Irregular menstrual cycle/bleeding | 626.1 | 1 | 2 | 861 | 16888 | 0.139 |
| Depression | 296.2 | 1 | 2 | 863 | 16925 | 0.139 |
| Poisoning by antibiotics | 960 | 1 | 2 | 864 | 16925 | 0.139 |
| Diaphragmatic hernia | 550.2 | 1 | 2 | 865 | 16925 | 0.139 |
| Genital prolapse | 618 | 1 | 2 | 865 | 16889 | 0.139 |
| Nausea and vomiting | 789 | 1 | 2 | 867 | 16923 | 0.139 |
| Mood disorders | 296 | 1 | 2 | 872 | 16916 | 0.140 |
| Disorders of menstruation and other abnormal bleeding from female genital tract | 626 | 1 | 2 | 880 | 16871 | 0.142 |
| Urinary incontinence | 599.4 | 1 | 2 | 882 | 16907 | 0.142 |
| Missed abortion/Hydatidiform mole | 634.1 | 1 | 2 | 886 | 16845 | 0.143 |
| stress incontinence, female | 624.9 | 1 | 2 | 889 | 16865 | 0.143 |
| Excessive or frequent menstruation | 626.12 | 1 | 2 | 889 | 16859 | 0.143 |
| Hemorrhage of rectum and anus | 578.8 | 1 | 2 | 898 | 16890 | 0.144 |
| Occlusion of cerebral arteries | 433.2 | 1 | 2 | 910 | 16871 | 0.146 |
| Type 2 diabetes | 250.2 | 1 | 2 | 915 | 16875 | 0.147 |
| Gastrointestinal hemorrhage | 578 | 1 | 2 | 917 | 16872 | 0.147 |
| Diseases of hard tissues of teeth | 521 | 1 | 2 | 920 | 16869 | 0.148 |
| Hemorrhoids | 455 | 1 | 2 | 924 | 16865 | 0.148 |
| Diabetes mellitus | 250 | 1 | 2 | 927 | 16863 | 0.149 |
| Inflammatory bowel disease and other gastroenteritis and colitis | 555 | 1 | 2 | 930 | 16845 | 0.149 |
| Placenta previa and abruptio placenta | 635.3 | 1 | 2 | 931 | 16811 | 0.150 |
| Pneumococcal pneumonia | 480.11 | 1 | 2 | 934 | 16855 | 0.150 |
| Syncope and collapse | 788 | 1 | 2 | 935 | 16855 | 0.150 |
| Duodenal ulcer | 531.3 | 1 | 2 | 945 | 16828 | 0.151 |
| Benign neoplasm of colon | 208 | 1 | 2 | 953 | 16837 | 0.153 |
| Other specified gastritis | 535.8 | 1 | 2 | 953 | 16832 | 0.153 |
| Open wounds of extremities | 871 | 1 | 2 | 958 | 16831 | 0.153 |
| Colorectal cancer | 153 | 1 | 2 | 961 | 16825 | 0.154 |
| Degenerative skin conditions and other dermatoses | 702 | 1 | 2 | 969 | 16821 | 0.155 |
| Rheumatoid arthritis | 714.1 | 1 | 2 | 970 | 16814 | 0.155 |
| Pulmonary heart disease | 415 | 1 | 2 | 973 | 16811 | 0.156 |
| Sleep apnea | 327.3 | 1 | 2 | 984 | 16806 | 0.157 |
| Malignant neoplasm of rectum, rectosigmoid junction, and anus | 153.3 | 1 | 2 | 982 | 16757 | 0.157 |
| Other abnormality of urination | 599.9 | 1 | 2 | 983 | 16755 | 0.157 |
| Atrioventricular [AV] block | 426.2 | 1 | 2 | 983 | 16735 | 0.158 |
| Colon cancer | 153.2 | 1 | 2 | 989 | 16779 | 0.158 |
| Scar conditions and fibrosis of skin | 701.2 | 1 | 2 | 993 | 16766 | 0.159 |
| Degeneration of intervertebral disc | 722.6 | 1 | 2 | 995 | 16779 | 0.159 |
| Pain in joint | 745 | 1 | 2 | 1001 | 16789 | 0.160 |
| Rheumatoid arthritis and other inflammatory polyarthropathies | 714 | 1 | 2 | 1002 | 16782 | 0.160 |
| Infection/inflammation of internal prosthetic device; implant; and graft | 081 | 1 | 2 | 1002 | 16749 | 0.160 |
| Calculus of bile duct | 574.2 | 1 | 2 | 1006 | 16759 | 0.161 |
| Septal Deviations/Turbinate Hypertrophy | 470 | 1 | 2 | 1008 | 16780 | 0.161 |
| Viral infection | 079 | 1 | 2 | 1009 | 16766 | 0.161 |
| Effects of other external causes | 1015 | 1 | 2 | 1023 | 16757 | 0.163 |
| Regional enteritis | 555.1 | 1 | 2 | 1021 | 16719 | 0.163 |
| Abnormal findings on examination of urine | 598 | 1 | 2 | 1031 | 16751 | 0.164 |
| Seborrheic keratosis | 702.2 | 1 | 2 | 1033 | 16733 | 0.165 |
| Blood in stool | 578.2 | 1 | 2 | 1038 | 16727 | 0.166 |
| Noninflammatory disorders of vagina | 619.4 | 1 | 2 | 1039 | 16690 | 0.166 |
| Chronic sinusitis | 475 | 1 | 2 | 1045 | 16737 | 0.166 |
| Other inflammatory spondylopathies | 715 | 1 | 2 | 1048 | 16676 | 0.167 |
| Inflammatory and toxic neuropathy | 357 | 1 | 2 | 1066 | 16653 | 0.170 |
| Hyposmolality and/or hyponatremia | 276.12 | 1 | 2 | 1069 | 16646 | 0.171 |
| Noninflammatory disorders of vulva and perineum | 619.5 | 1 | 2 | 1071 | 16673 | 0.171 |
| Cancer of kidney and renal pelvis | 189.1 | 1 | 2 | 1081 | 16603 | 0.173 |
| Malignant neoplasm of kidney, except pelvis | 189.11 | 1 | 2 | 1087 | 16587 | 0.174 |
| Orthostatic hypotension | 458.1 | 1 | 2 | 1091 | 16615 | 0.174 |
| Benign neoplasm of ovary | 220 | 1 | 2 | 1094 | 16625 | 0.174 |
| Complications of cardiac/vascular device, implant, and graft | 854 | 1 | 2 | 1095 | 16615 | 0.175 |
| Pelvic inflammatory disease (PID) | 614.3 | 1 | 2 | 1094 | 16587 | 0.175 |
| Arterial embolism and thrombosis | 444 | 1 | 2 | 1094 | 16542 | 0.175 |
| Purpura and other hemorrhagic conditions | 287 | 1 | 2 | 1100 | 16628 | 0.175 |
| Thrombocytopenia | 287.3 | 1 | 2 | 1102 | 16620 | 0.175 |
| Digestive congenital anomalies | 750 | 1 | 2 | 1099 | 16555 | 0.176 |
| Acid-base balance disorder | 276.4 | 1 | 2 | 1103 | 16587 | 0.176 |
| Hypertrophy of female genital organs | 623 | 1 | 2 | 1116 | 16617 | 0.177 |
| Empyema and pneumothorax | 506 | 1 | 2 | 1117 | 16536 | 0.178 |
| Polymyalgia Rheumatica | 717 | 1 | 2 | 1122 | 16542 | 0.179 |
| Acidosis | 276.41 | 1 | 2 | 1127 | 16555 | 0.180 |
| Malunion and nonunion of fracture | 733.8 | 1 | 2 | 1129 | 16574 | 0.180 |
| Cardiac pacemaker/device in situ | 426.9 | 1 | 2 | 1132 | 16540 | 0.180 |
| Poisoning by anticonvulsants and anti-Parkinsonism drugs | 966 | 1 | 2 | 1126 | 16447 | 0.180 |
| Paroxysmal ventricular tachycardia | 427.12 | 1 | 2 | 1130 | 16503 | 0.180 |
| Cervicitis and endocervicitis | 614.51 | 1 | 2 | 1136 | 16569 | 0.181 |
| Cardiac pacemaker in situ | 426.91 | 1 | 2 | 1138 | 16517 | 0.181 |
| Simple and unspecified goiter | 240 | 1 | 2 | 1146 | 16516 | 0.183 |
| Other diseases of blood and blood-forming organs | 289 | 1 | 2 | 1160 | 16529 | 0.184 |
| Strabismus and other disorders of binocular eye movements | 378 | 1 | 2 | 1165 | 16555 | 0.185 |
| Sleep disorders | 327 | 1 | 2 | 1164 | 16485 | 0.185 |
| Crystal arthropathies | 274.2 | 1 | 2 | 1163 | 16453 | 0.186 |
| Fracture of pelvis | 802 | 1 | 2 | 1169 | 16433 | 0.187 |
| Hyperparathyroidism | 252.1 | 1 | 2 | 1175 | 16494 | 0.187 |
| Trigeminal nerve disorders [CN5] | 352.1 | 1 | 2 | 1175 | 16443 | 0.187 |
| Strabismus (not specified as paralytic) | 378.1 | 1 | 2 | 1181 | 16472 | 0.188 |
| Myeloproliferative disease | 200 | 1 | 2 | 1184 | 16487 | 0.188 |
| Symptoms affecting skin | 687 | 1 | 2 | 1186 | 16427 | 0.189 |
| Disorders of parathyroid gland | 252 | 1 | 2 | 1194 | 16493 | 0.189 |
| Cellulitis and abscess of face/neck | 681.2 | 1 | 2 | 1192 | 16421 | 0.190 |
| Respiratory abnormalities | 513 | 1 | 2 | 1198 | 16417 | 0.191 |
| Anorexia | 260.6 | 1 | 2 | 1210 | 16407 | 0.192 |
| Varicose veins of lower extremity, symptomtic | 454.11 | 1 | 2 | 1214 | 16400 | 0.193 |
| Infection of the eye | 369 | 1 | 2 | 1218 | 16446 | 0.193 |
| Abnormal function study of cardiovascular system | 429.2 | 1 | 2 | 1225 | 16403 | 0.195 |
| Ill-defined descriptions and complications of heart disease | 429 | 1 | 2 | 1229 | 16432 | 0.195 |
| Disorders of the pituitary gland and its hypothalamic control | 253 | 1 | 2 | 1232 | 16372 | 0.196 |
| Laxity of ligament or hypermobility syndrome | 728.2 | 1 | 2 | 1266 | 16278 | 0.201 |
| Cellulitis and abscess of fingers/toes | 681.1 | 1 | 2 | 1328 | 16226 | 0.210 |
| Chronic bronchitis | 496.2 | 0 | 3 | 967 | 16815 | 1.000 |
| Abdominal hernia | 550 | 0 | 3 | 931 | 16859 | 1.000 |
| Rupture of synovium | 727.5 | 0 | 3 | 1190 | 16425 | 1.000 |
| Delirium due to conditions classified elsewhere | 290.2 | 0 | 3 | 1155 | 16400 | 1.000 |
| Pneumonia | 480 | 0 | 3 | 964 | 16826 | 1.000 |
| Lump or mass in breast | 611.3 | 0 | 3 | 937 | 16825 | 1.000 |
| Other disorders of soft tissues | 729 | 0 | 3 | 907 | 16881 | 1.000 |
| Senile cataract | 366.2 | 0 | 3 | 901 | 16888 | 1.000 |
| Noninfectious gastroenteritis | 558 | 0 | 3 | 849 | 16926 | 1.000 |
| Synovitis and tenosynovitis | 727.1 | 0 | 3 | 1011 | 16753 | 1.000 |
| Streptococcus infection | 041.2 | 0 | 3 | 1081 | 16660 | 1.000 |
| Acquired toe deformities | 735.2 | 0 | 3 | 952 | 16836 | 1.000 |
| Subjective visual disturbances | 368.9 | 0 | 3 | 1177 | 16432 | 1.000 |
| Tinnitus | 389.4 | 0 | 3 | 1187 | 16338 | 1.000 |
| Chronic periodontitis | 523.32 | 0 | 3 | 1202 | 16407 | 1.000 |
| Myocardial infarction | 411.2 | 0 | 3 | 922 | 16864 | 1.000 |
| Preeclampsia and eclampsia | 642.1 | 0 | 3 | 933 | 16637 | 1.000 |
| Stricture of artery | 447.1 | 0 | 3 | 1159 | 16447 | 1.000 |
| Malaise and fatigue | 798 | 0 | 3 | 969 | 16802 | 1.000 |
| Cellulitis and abscess of arm/hand | 681.3 | 0 | 3 | 955 | 16833 | 1.000 |
| Atherosclerosis | 440 | 0 | 3 | 1026 | 16666 | 1.000 |
| Heart valve disorders | 395 | 0 | 3 | 945 | 16842 | 1.000 |
| Other disorders of stomach and duodenum | 537 | 0 | 3 | 981 | 16803 | 1.000 |
| Ingrowing nail | 703.1 | 0 | 3 | 1082 | 16607 | 1.000 |
| Degenerative disease of the spinal cord | 334 | 0 | 3 | 1116 | 16615 | 1.000 |
| Antepartum hemorrhage, abruptio placentae, and placenta previa | 635.2 | 0 | 3 | 862 | 16856 | 1.000 |
| Mechanical complication of unspecified genitourinary device, implant, and graft | 857 | 0 | 3 | 1138 | 16566 | 1.000 |
| Crushing or internal injury to organs | 1008 | 0 | 3 | 1135 | 16548 | 1.000 |
| Benign mammary dysplasias | 610 | 0 | 3 | 967 | 16783 | 1.000 |
| Complications of labor and delivery NEC | 669 | 0 | 3 | 899 | 16853 | 1.000 |
| Arthropathy NOS | 716.9 | 0 | 3 | 965 | 16824 | 1.000 |
| Other benign neoplasm of connective and other soft tissue | 215 | 0 | 3 | 1065 | 16624 | 1.000 |
| Diseases of the salivary glands | 527 | 0 | 3 | 1158 | 16474 | 1.000 |
| Osteopenia or other disorder of bone and cartilage | 743.9 | 0 | 3 | 1181 | 16434 | 1.000 |
| Perforation of tympanic membrane | 384.4 | 0 | 3 | 1159 | 16519 | 1.000 |
| Mitral valve disease | 394.2 | 0 | 3 | 986 | 16776 | 1.000 |
| Infertility, female | 626.8 | 0 | 3 | 961 | 16744 | 1.000 |
| Glomerulonephritis | 580.1 | 0 | 3 | 1082 | 16578 | 1.000 |
| Fracture of upper limb | 803 | 0 | 3 | 950 | 16839 | 1.000 |
| Contusion | 916 | 0 | 3 | 1113 | 16591 | 1.000 |
| Polyp of female genital organs | 622 | 0 | 3 | 855 | 16899 | 1.000 |
| Noninfectious disorders of lymphatic channels | 450 | 0 | 3 | 1189 | 16431 | 1.000 |
| Hammer toe (acquired) | 735.21 | 0 | 3 | 1024 | 16709 | 1.000 |
| Swelling, mass, or lump in head and neck [Space-occupying lesion, intracranial NOS] | 293.1 | 0 | 3 | 1141 | 16515 | 1.000 |
| Stricture/obstruction of ureter | 586.4 | 0 | 3 | 1146 | 16540 | 1.000 |
| Cellulitis and abscess of foot, toe | 681.6 | 0 | 3 | 957 | 16831 | 1.000 |
| Hepatitis NOS | 070.9 | 0 | 3 | 1254 | 16303 | 1.000 |
| Ulceration of intestine | 556.1 | 0 | 3 | 1136 | 16512 | 1.000 |
| Heart valve replaced | 395.6 | 0 | 3 | 1069 | 16643 | 1.000 |
| Osteoarthrosis NOS | 740.9 | 0 | 3 | 927 | 16860 | 1.000 |
| Circulatory disease NEC | 459.9 | 0 | 3 | 941 | 16849 | 1.000 |
| Occlusion and stenosis of precerebral arteries | 433.1 | 0 | 3 | 1102 | 16564 | 1.000 |
| Osteoarthrosis, generalized | 740.2 | 0 | 3 | 1054 | 16608 | 1.000 |
| Diseases of the larynx and vocal cords | 473 | 0 | 3 | 1034 | 16743 | 1.000 |
| Inflammatory diseases of female pelvic organs | 614 | 0 | 3 | 869 | 16885 | 1.000 |
| Cellulitis and abscess of leg, except foot | 681.5 | 0 | 3 | 957 | 16831 | 1.000 |
| Fetal distress and abnormal forces of labor | 661 | 0 | 3 | 870 | 16883 | 1.000 |
| Foreign body injury | 1001 | 0 | 3 | 1135 | 16563 | 1.000 |
| Disorders of refraction and accommodation; blindness and low vision | 367 | 0 | 3 | 1018 | 16729 | 1.000 |
| Chronic prostatitis | 601.12 | 0 | 3 | 1040 | 16665 | 1.000 |
| Dysuria | 599.3 | 0 | 3 | 1181 | 16526 | 1.000 |
| Asthma with exacerbation | 495.2 | 0 | 3 | 1100 | 16638 | 1.000 |
| Manlignant and unknown neoplasms of brain and nervous system | 191 | 0 | 3 | 1310 | 16261 | 1.000 |
| Acute renal failure | 585.1 | 0 | 3 | 1028 | 16751 | 1.000 |
| Other forms of chronic heart disease | 414 | 0 | 3 | 1043 | 16669 | 1.000 |
| Diseases of the jaws | 526 | 0 | 3 | 1092 | 16589 | 1.000 |
| Pruritus and related conditions | 698 | 0 | 3 | 1169 | 16484 | 1.000 |
| Constipation | 563 | 0 | 3 | 870 | 16908 | 1.000 |
| Other anemias | 285 | 0 | 3 | 906 | 16884 | 1.000 |
| Leukemia | 204 | 0 | 3 | 1137 | 16585 | 1.000 |
| Cardiac dysrhythmias | 427 | 0 | 3 | 892 | 16897 | 1.000 |
| Rash and other nonspecific skin eruption | 687.1 | 0 | 3 | 1063 | 16695 | 1.000 |
| Inflammatory disease of breast | 613.1 | 0 | 3 | 1142 | 16494 | 1.000 |
| Other diseases of the teeth and supporting structures | 525 | 0 | 3 | 1002 | 16775 | 1.000 |
| Cancer of other lymphoid, histiocytic tissue | 202 | 0 | 3 | 1045 | 16706 | 1.000 |
| Left bundle branch block | 426.32 | 0 | 3 | 1075 | 16654 | 1.000 |
| Other upper respiratory disease | 479 | 0 | 3 | 953 | 16835 | 1.000 |
| Viral warts & HPV | 078 | 0 | 3 | 1118 | 16570 | 1.000 |
| Cerebrovascular disease | 433 | 0 | 3 | 946 | 16844 | 1.000 |
| Malignant neoplasm of other and ill-defined sites within the digestive organs and peritoneum | 159 | 0 | 3 | 973 | 16815 | 1.000 |
| Osteoporosis, osteopenia and pathological fracture | 743 | 0 | 3 | 881 | 16909 | 1.000 |
| Malignant neoplasm of testis | 187.2 | 0 | 3 | 973 | 16773 | 1.000 |
| Ulcer of esophagus | 530.12 | 0 | 3 | 899 | 16886 | 1.000 |
| Diseases of hair and hair follicles | 704 | 0 | 3 | 938 | 16852 | 1.000 |
| Other derangement of joint | 742.9 | 0 | 3 | 1081 | 16664 | 1.000 |
| Injury, NOS | 1009 | 0 | 3 | 939 | 16851 | 1.000 |
| Aortic aneurysm | 442.1 | 0 | 3 | 998 | 16678 | 1.000 |
| Neutropenia | 288.11 | 0 | 3 | 1035 | 16737 | 1.000 |
| Abnormal findings on mammogram or breast exam | 611 | 0 | 3 | 922 | 16841 | 1.000 |
| Intervertebral disc disorders | 722 | 0 | 3 | 908 | 16882 | 1.000 |
| Peripheral vascular disease | 443 | 0 | 3 | 1008 | 16769 | 1.000 |
| Influenza | 481 | 0 | 3 | 1260 | 16287 | 1.000 |
| Decreased white blood cell count | 288.1 | 0 | 3 | 1035 | 16737 | 1.000 |
| Early or threatened labor; hemorrhage in early pregnancy | 636 | 0 | 3 | 873 | 16880 | 1.000 |
| Respiratory failure | 509.1 | 0 | 3 | 1005 | 16749 | 1.000 |
| Anxiety disorder | 300.1 | 0 | 3 | 942 | 16844 | 1.000 |
| Aphasia/speech disturbance | 292.1 | 0 | 3 | 1066 | 16643 | 1.000 |
| Inflammation of the eye | 371 | 0 | 3 | 925 | 16859 | 1.000 |
| Liver abscess and sequelae of chronic liver disease | 571.8 | 0 | 3 | 1079 | 16590 | 1.000 |
| Non-Hodgkins lymphoma | 202.2 | 0 | 3 | 1094 | 16644 | 1.000 |
| Disorders of uterus, NEC | 619.2 | 0 | 3 | 961 | 16792 | 1.000 |
| Disorders of tooth development | 520 | 0 | 3 | 1063 | 16696 | 1.000 |
| Polyarteritis nodosa and allied conditions | 446 | 0 | 3 | 1091 | 16539 | 1.000 |
| Aplastic anemia | 284 | 0 | 3 | 1233 | 16324 | 1.000 |
| Cellulitis and abscess of trunk | 681.7 | 0 | 3 | 1188 | 16421 | 1.000 |
| Cancer of bladder | 189.2 | 0 | 3 | 1070 | 16689 | 1.000 |
| Vitamin D deficiency | 261.4 | 0 | 3 | 1181 | 16409 | 1.000 |
| Chronic airway obstruction | 496 | 0 | 3 | 928 | 16855 | 1.000 |
| Disturbances in tooth eruption | 520.2 | 0 | 3 | 1061 | 16698 | 1.000 |
| Multiple myeloma | 204.4 | 0 | 3 | 1177 | 16374 | 1.000 |
| Injuries to the nervous system | 907 | 0 | 3 | 1120 | 16581 | 1.000 |
| Other disorders of eyelids | 374 | 0 | 3 | 909 | 16881 | 1.000 |
| Ventral hernia | 550.5 | 0 | 3 | 997 | 16785 | 1.000 |
| Shortness of breath | 512.7 | 0 | 3 | 903 | 16887 | 1.000 |
| Anxiety disorders | 300 | 0 | 3 | 931 | 16855 | 1.000 |
| Other unspecified back disorders | 724.9 | 0 | 3 | 1047 | 16683 | 1.000 |
| Thyrotoxicosis with or without goiter | 242 | 0 | 3 | 1055 | 16692 | 1.000 |
| Renal failure | 585 | 0 | 3 | 980 | 16810 | 1.000 |
| Urinary tract infection | 591 | 0 | 3 | 912 | 16875 | 1.000 |
| Gout | 274.1 | 0 | 3 | 903 | 16872 | 1.000 |
| Hemorrhage from gastrointestinal ulcer | 531.1 | 0 | 3 | 1234 | 16324 | 1.000 |
| Glucocorticoid deficiency | 255.21 | 0 | 3 | 1265 | 16285 | 1.000 |
| Lipoma of skin and subcutaneous tissue | 214.1 | 0 | 3 | 952 | 16838 | 1.000 |
| Abnormal involuntary movements | 350.1 | 0 | 3 | 1149 | 16493 | 1.000 |
| Adrenal hypofunction | 255.2 | 0 | 3 | 1265 | 16285 | 1.000 |
| Neuralgia, neuritis, and radiculitis NOS | 766 | 0 | 3 | 1096 | 16624 | 1.000 |
| Esophageal bleeding (varices/hemorrhage) | 530.2 | 0 | 3 | 1031 | 16696 | 1.000 |
| Miscarriage; stillbirth | 634 | 0 | 3 | 919 | 16833 | 1.000 |
| Urinary calculus | 594 | 0 | 3 | 932 | 16858 | 1.000 |
| Other tests | 1010 | 0 | 3 | 925 | 16865 | 1.000 |
| Superficial cellulitis and abscess | 681 | 0 | 3 | 908 | 16881 | 1.000 |
| Osteoarthrosis | 740 | 0 | 3 | 901 | 16889 | 1.000 |
| Peripheral enthesopathies and allied syndromes | 726 | 0 | 3 | 876 | 16910 | 1.000 |
| Nontoxic multinodular goiter | 241.2 | 0 | 3 | 1174 | 16470 | 1.000 |
| Other paralytic syndromes | 344 | 0 | 3 | 1191 | 16429 | 1.000 |
| Fracture of ribs | 807 | 0 | 3 | 1172 | 16452 | 1.000 |
| Right bundle branch block | 426.31 | 0 | 3 | 1074 | 16644 | 1.000 |
| Macular degeneration (senile) of retina NOS | 362.29 | 0 | 3 | 1020 | 16728 | 1.000 |
| Subarachnoid hemorrhage | 430.1 | 0 | 3 | 1217 | 16418 | 1.000 |
| Ascites (non malignant) | 572 | 0 | 3 | 1136 | 16554 | 1.000 |
| Other diseases of respiratory system, not elsewhere classified | 519 | 0 | 3 | 891 | 16899 | 1.000 |
| Postinflammatory pulmonary fibrosis | 502 | 0 | 3 | 1125 | 16467 | 1.000 |
| Renal dialysis | 585.31 | 0 | 3 | 1257 | 16305 | 1.000 |
| Extrapyramidal disease and abnormal movement disorders | 333 | 0 | 3 | 1179 | 16489 | 1.000 |
| Other dyspnea | 512.9 | 0 | 3 | 1169 | 16445 | 1.000 |
| Fracture of unspecified bones | 809 | 0 | 3 | 1099 | 16606 | 1.000 |
| Reflux esophagitis | 530.14 | 0 | 3 | 917 | 16869 | 1.000 |
| Ganglion and cyst of synovium, tendon, and bursa | 727.4 | 0 | 3 | 983 | 16797 | 1.000 |
| Rheumatic disease of the heart valves | 394 | 0 | 3 | 1003 | 16787 | 1.000 |
| Coronary atherosclerosis | 411.4 | 0 | 3 | 884 | 16902 | 1.000 |
| Other ill-defined and unknown causes of morbidity and mortality | 1019 | 0 | 3 | 881 | 16909 | 1.000 |
| Other disorders of eye | 379 | 0 | 3 | 968 | 16802 | 1.000 |
| Joint effusions | 741.4 | 0 | 3 | 1109 | 16590 | 1.000 |
| Inflammatory diseases of prostate | 601 | 0 | 3 | 916 | 16820 | 1.000 |
| Back pain | 760 | 0 | 3 | 913 | 16877 | 1.000 |
| Iron deficiency anemias | 280 | 0 | 3 | 914 | 16873 | 1.000 |
| Degeneration of macula and posterior pole of retina | 362.2 | 0 | 3 | 1020 | 16728 | 1.000 |
| Neoplasm of unspecified nature of digestive system | 158 | 0 | 3 | 1101 | 16579 | 1.000 |
| Sepsis and SIRS | 994 | 0 | 3 | 991 | 16769 | 1.000 |
| Swelling of limb | 771.1 | 0 | 3 | 971 | 16819 | 1.000 |
| Fracture of neck of femur | 800.1 | 0 | 3 | 1074 | 16638 | 1.000 |
| Nonrheumatic mitral valve disorders | 395.1 | 0 | 3 | 1005 | 16755 | 1.000 |
| Personal history of diseases of digestive system | 564.9 | 0 | 3 | 881 | 16896 | 1.000 |
| Disorders of muscle, ligament, and fascia | 728 | 0 | 3 | 1013 | 16768 | 1.000 |
| Chronic ulcer of skin | 707 | 0 | 3 | 1040 | 16704 | 1.000 |
| Other arthropathies | 716 | 0 | 3 | 975 | 16814 | 1.000 |
| Vitamin B-complex deficiencies | 261.2 | 0 | 3 | 1141 | 16497 | 1.000 |
| Abdominal pain | 785 | 0 | 3 | 887 | 16903 | 1.000 |
| Other disorders of bone and cartilage | 733 | 0 | 3 | 976 | 16745 | 1.000 |
| Malignant neoplasm of ovary and other uterine adnexa | 184.1 | 0 | 3 | 1018 | 16717 | 1.000 |
| Prolapse of vaginal walls | 618.1 | 0 | 3 | 881 | 16873 | 1.000 |
| Other specified nonpsychotic and/or transient mental disorders | 291 | 0 | 3 | 1236 | 16381 | 1.000 |
| Bacterial infection NOS | 041 | 0 | 3 | 867 | 16922 | 1.000 |
| Abdominal aortic aneurysm | 442.11 | 0 | 3 | 1040 | 16568 | 1.000 |
| Vascular hamartomas and non-neoplastic nevi | 217 | 0 | 3 | 1217 | 16353 | 1.000 |
| Other retinal disorders | 362 | 0 | 3 | 1008 | 16767 | 1.000 |
| Peritoneal or intestinal adhesions | 560.3 | 0 | 3 | 1095 | 16563 | 1.000 |
| Sepsis | 994.2 | 0 | 3 | 991 | 16769 | 1.000 |
| Appendicitis | 540.1 | 0 | 3 | 1000 | 16774 | 1.000 |
| Malposition and malpresentation of fetus or obstruction | 652 | 0 | 3 | 911 | 16842 | 1.000 |
| Appendiceal conditions | 540 | 0 | 3 | 971 | 16808 | 1.000 |
| Iron deficiency anemia secondary to blood loss (chronic) | 280.2 | 0 | 3 | 1137 | 16442 | 1.000 |
| Chronic pain | 338.2 | 0 | 3 | 1174 | 16384 | 1.000 |
| Chemotherapy | 197 | 0 | 3 | 842 | 16946 | 1.000 |
| GERD | 530.11 | 0 | 3 | 864 | 16924 | 1.000 |
| Bundle branch block | 426.3 | 0 | 3 | 1059 | 16716 | 1.000 |
| Tachycardia NOS | 427.7 | 0 | 3 | 1044 | 16699 | 1.000 |
| Other conditions of brain | 348 | 0 | 3 | 1198 | 16373 | 1.000 |
| Other abnormal blood chemistry | 790.6 | 0 | 3 | 942 | 16848 | 1.000 |
| Urethral stricture (not specified as infectious) | 597.1 | 0 | 3 | 1012 | 16765 | 1.000 |
| Hematuria | 593 | 0 | 3 | 860 | 16929 | 1.000 |
| Other chronic ischemic heart disease, unspecified | 411.8 | 0 | 3 | 909 | 16879 | 1.000 |
| Benign neoplasm of other endocrine glands and related structures | 227 | 0 | 3 | 1170 | 16506 | 1.000 |
| Ulceration of the lower GI tract | 556 | 0 | 3 | 1089 | 16614 | 1.000 |
| Anemia of chronic disease | 285.2 | 0 | 3 | 1119 | 16469 | 1.000 |
| Peritonitis and retroperitoneal infections | 567 | 0 | 3 | 1215 | 16416 | 1.000 |
| First degree AV block | 426.21 | 0 | 3 | 1072 | 16566 | 1.000 |
| Myalgia and myositis unspecified | 770 | 0 | 3 | 1140 | 16468 | 1.000 |
| Cancer of other female genital organs | 184 | 0 | 3 | 1012 | 16733 | 1.000 |
| Irregular menstrual bleeding | 626.14 | 0 | 3 | 850 | 16897 | 1.000 |
| Pyelonephritis | 590 | 0 | 3 | 1028 | 16702 | 1.000 |
| Dementias | 290.1 | 0 | 3 | 1104 | 16462 | 1.000 |
| Decubitus ulcer | 707.1 | 0 | 3 | 1130 | 16521 | 1.000 |
| Functional digestive disorders | 564 | 0 | 3 | 875 | 16905 | 1.000 |
| Contracture of joint | 739 | 0 | 3 | 1223 | 16368 | 1.000 |
| Nontoxic nodular goiter | 241 | 0 | 3 | 1122 | 16585 | 1.000 |
| Diverticulosis | 562.1 | 0 | 3 | 924 | 16857 | 1.000 |
| Diverticulosis and diverticulitis | 562 | 0 | 3 | 924 | 16857 | 1.000 |
| Other disorders of cervical region | 723 | 0 | 3 | 1245 | 16274 | 1.000 |
| Chronic fatigue syndrome | 798.1 | 0 | 3 | 1178 | 16382 | 1.000 |
| Iron deficiency anemias, unspecified or not due to blood loss | 280.1 | 0 | 3 | 909 | 16878 | 1.000 |
| Other cerebral degenerations | 331 | 0 | 3 | 1144 | 16503 | 1.000 |
| Congenital anomalies of urinary system | 751.2 | 0 | 3 | 1152 | 16495 | 1.000 |
| Cervical cancer and dysplasia | 180 | 0 | 3 | 877 | 16869 | 1.000 |
| Phobia | 300.13 | 0 | 3 | 1192 | 16393 | 1.000 |
| Other peripheral nerve disorders | 351 | 0 | 3 | 903 | 16887 | 1.000 |
| Sarcoidosis | 697 | 0 | 3 | 1222 | 16396 | 1.000 |
| Adverse drug events and drug allergies | 979 | 0 | 3 | 1179 | 16461 | 1.000 |
| Acquired foot deformities | 735 | 0 | 3 | 925 | 16865 | 1.000 |
| Mycoses | 117 | 0 | 3 | 1224 | 16371 | 1.000 |
| Other headache syndromes | 339 | 0 | 3 | 927 | 16863 | 1.000 |
| Jaundice (not of newborn) | 573.5 | 0 | 3 | 1195 | 16485 | 1.000 |
| Symptoms involving head and neck | 293 | 0 | 3 | 1030 | 16738 | 1.000 |
| Carbuncle and furuncle | 686.1 | 0 | 3 | 971 | 16795 | 1.000 |
| Fasciitis | 728.7 | 0 | 3 | 1012 | 16773 | 1.000 |
| Meniere's disease | 386.1 | 0 | 3 | 1187 | 16357 | 1.000 |
| Prostatitis | 601.1 | 0 | 3 | 1007 | 16729 | 1.000 |
| Irritable Bowel Syndrome | 564.1 | 0 | 3 | 931 | 16847 | 1.000 |
| Other disorders of circulatory system | 459 | 0 | 3 | 942 | 16848 | 1.000 |
| Bipolar | 296.1 | 0 | 3 | 1083 | 16623 | 1.000 |
| Contracture of palmar fascia [Dupuytren's disease] | 728.71 | 0 | 3 | 1026 | 16752 | 1.000 |
| Spinal stenosis | 720 | 0 | 3 | 956 | 16824 | 1.000 |
| Obstructive chronic bronchitis | 496.21 | 0 | 3 | 995 | 16787 | 1.000 |
| Retinal vascular changes and abnomalities | 362.4 | 0 | 3 | 1060 | 16627 | 1.000 |
| Acute tonsillitis | 474.1 | 0 | 3 | 1185 | 16457 | 1.000 |
| Atopic/contact dermatitis due to other or unspecified | 939 | 0 | 3 | 1040 | 16716 | 1.000 |
| Cystic mastopathy | 610.1 | 0 | 3 | 1111 | 16573 | 1.000 |
| Internal derangement of knee | 835 | 0 | 3 | 936 | 16854 | 1.000 |
| Multiple gestation | 651 | 0 | 3 | 967 | 16646 | 1.000 |
| Unstable angina (intermediate coronary syndrome) | 411.1 | 0 | 3 | 887 | 16899 | 1.000 |
| Early onset of delivery | 636.2 | 0 | 3 | 929 | 16744 | 1.000 |
| Inflammatory disease of cervix, vagina, and vulva | 614.5 | 0 | 3 | 1015 | 16737 | 1.000 |
| Chronic pharyngitis and nasopharyngitis | 472 | 0 | 3 | 1193 | 16498 | 1.000 |
| Benign neoplasm of other parts of digestive system | 211 | 0 | 3 | 943 | 16844 | 1.000 |
| Psychogenic and somatoform disorders | 303 | 0 | 3 | 1189 | 16477 | 1.000 |
| Other specified cardiac dysrhythmias | 427.3 | 0 | 3 | 997 | 16777 | 1.000 |
| Complications of surgical and medical procedures | 1011 | 0 | 3 | 913 | 16877 | 1.000 |
| Chronic liver disease and cirrhosis | 571 | 0 | 3 | 1037 | 16733 | 1.000 |
| Vertiginous syndromes and other disorders of vestibular system | 386 | 0 | 3 | 1032 | 16696 | 1.000 |
| Secondary malignancy of respiratory organs | 198.2 | 0 | 3 | 1027 | 16705 | 1.000 |
| Symptoms involving female genital tract | 624 | 0 | 3 | 1142 | 16483 | 1.000 |
| Diseases of lips | 528.5 | 0 | 3 | 1197 | 16445 | 1.000 |
| Benign neoplasm of brain, cranial nerves, meninges | 225.1 | 0 | 3 | 1173 | 16467 | 1.000 |
| Ectropion or entropion | 374.1 | 0 | 3 | 1083 | 16576 | 1.000 |
| Retinal detachments and defects | 361 | 0 | 3 | 1013 | 16765 | 1.000 |
| Abnormal heart sounds | 396 | 0 | 3 | 1181 | 16510 | 1.000 |
| Celiac disease | 557.1 | 0 | 3 | 973 | 16778 | 1.000 |
| Pain | 338 | 0 | 3 | 1115 | 16571 | 1.000 |
| Lymphadenitis | 289.4 | 0 | 3 | 1040 | 16726 | 1.000 |
| Kyphoscoliosis and scoliosis | 737.3 | 0 | 3 | 1130 | 16565 | 1.000 |
| Fracture of tibia and fibula | 800.3 | 0 | 3 | 1120 | 16627 | 1.000 |
| Voice disturbance | 473.4 | 0 | 3 | 1070 | 16627 | 1.000 |
| Diffuse diseases of connective tissue | 709 | 0 | 3 | 982 | 16807 | 1.000 |
| Cataract | 366 | 0 | 3 | 803 | 16987 | 1.000 |
| Esophagitis, GERD and related diseases | 530.1 | 0 | 3 | 892 | 16898 | 1.000 |
| Nonspecific chest pain | 418 | 0 | 3 | 864 | 16926 | 1.000 |
| Abnormality of gait | 350.2 | 0 | 3 | 1123 | 16584 | 1.000 |
| Abnormal findings examination of lungs | 514 | 0 | 3 | 994 | 16767 | 1.000 |
| Cancer of mouth | 145 | 0 | 3 | 1209 | 16404 | 1.000 |
| Benign neoplasm of brain and other parts of nervous system | 225 | 0 | 3 | 1191 | 16460 | 1.000 |
| Other mental disorder | 306 | 0 | 3 | 870 | 16919 | 1.000 |
| Allergy/adverse effect of penicillin | 960.2 | 0 | 3 | 872 | 16917 | 1.000 |
| Benign neoplasm of lip, oral cavity, and pharynx | 210 | 0 | 3 | 1139 | 16524 | 1.000 |
| Open-angle glaucoma | 365.1 | 0 | 3 | 1112 | 16495 | 1.000 |
| Pulmonary collapse; interstitial and compensatory emphysema | 508 | 0 | 3 | 1004 | 16737 | 1.000 |
| Diseases of white blood cells | 288 | 0 | 3 | 1045 | 16736 | 1.000 |
| Malignant neoplasm of ovary | 184.11 | 0 | 3 | 1036 | 16697 | 1.000 |
| Ileostomy status | 559 | 0 | 3 | 1025 | 16689 | 1.000 |
| Epilepsy | 345.1 | 0 | 3 | 1064 | 16605 | 1.000 |
| Sprains and strains | 840 | 0 | 3 | 1258 | 16312 | 1.000 |
| Diseases of sebaceous glands | 706 | 0 | 3 | 902 | 16888 | 1.000 |
| Secondary malignant neoplasm of liver | 198.4 | 0 | 3 | 1018 | 16729 | 1.000 |
| Complication due to other implant and internal device | 859 | 0 | 3 | 979 | 16791 | 1.000 |
| Alteration of consciousness | 291.8 | 0 | 3 | 1285 | 16268 | 1.000 |
| Allergic rhinitis | 476 | 0 | 3 | 1075 | 16634 | 1.000 |
| Raynaud's syndrome | 443.1 | 0 | 3 | 1069 | 16638 | 1.000 |
| Aphakia and other disorders of lens | 379.3 | 0 | 3 | 1073 | 16605 | 1.000 |
| Other dyschromia | 694.2 | 0 | 3 | 1114 | 16555 | 1.000 |
| Diseases of pancreas | 577 | 0 | 3 | 1018 | 16744 | 1.000 |
| Ischemic Heart Disease | 411 | 0 | 3 | 934 | 16857 | 1.000 |
| Curvature of spine | 737 | 0 | 3 | 1136 | 16561 | 1.000 |
| Delirium dementia and amnestic and other cognitive disorders | 290 | 0 | 3 | 1002 | 16710 | 1.000 |
| Functional disorders of bladder | 596.5 | 0 | 3 | 1088 | 16617 | 1.000 |
| Primary open angle glaucoma | 365.11 | 0 | 3 | 1112 | 16495 | 1.000 |
| Nevus, non-neoplastic | 217.1 | 0 | 3 | 1215 | 16339 | 1.000 |
| Pernicious anemia | 281.11 | 0 | 3 | 1151 | 16483 | 1.000 |
| Other acquired deformities of limbs | 736 | 0 | 3 | 1074 | 16635 | 1.000 |
| Adverse effects of sedatives or other central nervous system depressants and anesthetics | 967 | 0 | 3 | 1169 | 16443 | 1.000 |
| Acute appendicitis | 540.11 | 0 | 3 | 1042 | 16718 | 1.000 |
| Diabetic retinopathy | 250.7 | 0 | 3 | 1113 | 16572 | 1.000 |
| Other diseases of respiratory system, NEC | 519.8 | 0 | 3 | 885 | 16905 | 1.000 |
| Disorders of other cranial nerves | 352 | 0 | 3 | 1086 | 16637 | 1.000 |
| Malignant neoplasm of bladder | 189.21 | 0 | 3 | 1068 | 16681 | 1.000 |
| Dental caries | 521.1 | 0 | 3 | 950 | 16839 | 1.000 |
| Skull and face fracture and other intercranial injury | 819 | 0 | 3 | 1039 | 16729 | 1.000 |
| Hypotension | 458 | 0 | 3 | 918 | 16869 | 1.000 |
| Umbilical hernia | 550.4 | 0 | 3 | 965 | 16818 | 1.000 |
| Diplopia and disorders of binocular vision | 368.2 | 0 | 3 | 1236 | 16371 | 1.000 |
| Sebaceous cyst | 706.2 | 0 | 3 | 897 | 16893 | 1.000 |
| Disorder of skin and subcutaneous tissue NOS | 689 | 0 | 3 | 945 | 16845 | 1.000 |
| Cyst or abscess of Bartholin's gland | 614.53 | 0 | 3 | 1168 | 16491 | 1.000 |
| Schizophrenia | 295.1 | 0 | 3 | 1231 | 16358 | 1.000 |
| Effects radiation NOS | 990 | 0 | 3 | 1036 | 16730 | 1.000 |
| Postmenopausal atrophic vaginitis | 627.3 | 0 | 3 | 1100 | 16561 | 1.000 |
| Frequency of urination and polyuria | 599.5 | 0 | 3 | 971 | 16818 | 1.000 |
| Benign neoplasm of unspecified sites | 229 | 0 | 3 | 1040 | 16726 | 1.000 |
| Other symptoms involving abdomen and pelvis | 579 | 0 | 3 | 1088 | 16645 | 1.000 |
| Visual disturbances | 368 | 0 | 3 | 1020 | 16744 | 1.000 |
| Fracture of vertebral column without mention of spinal cord injury | 805 | 0 | 3 | 1086 | 16625 | 1.000 |
| Postoperative infection | 080 | 0 | 3 | 946 | 16844 | 1.000 |
| Stiffness of joint | 741.2 | 0 | 3 | 1169 | 16506 | 1.000 |
| Hemoptysis | 516.1 | 0 | 3 | 1004 | 16748 | 1.000 |
| Type 1 diabetes | 250.1 | 0 | 3 | 977 | 16784 | 1.000 |
| Atrial fibrillation and flutter | 427.2 | 0 | 3 | 919 | 16870 | 1.000 |
| Flatulence | 561.2 | 0 | 3 | 924 | 16826 | 1.000 |
| Disorders of calcium/phosphorus metabolism | 275.5 | 0 | 3 | 1144 | 16559 | 1.000 |
| Poisoning by other anti-infectives | 961 | 0 | 3 | 1253 | 16296 | 1.000 |
| Phlebitis and thrombophlebitis | 451 | 0 | 3 | 946 | 16841 | 1.000 |
| Disorders of mineral metabolism | 275 | 0 | 3 | 1058 | 16698 | 1.000 |
| Malignant neoplasm, other | 195.1 | 0 | 3 | 875 | 16914 | 1.000 |
| Bronchitis | 497 | 0 | 3 | 1070 | 16615 | 1.000 |
| Normal delivery | 650 | 0 | 3 | 906 | 16848 | 1.000 |
| Ptosis of eyelid | 374.3 | 0 | 3 | 982 | 16762 | 1.000 |
| Symptoms of the muscles | 772 | 0 | 3 | 1186 | 16384 | 1.000 |
| Breast conditions, congenital or relating to hormones | 612 | 0 | 3 | 1009 | 16704 | 1.000 |
| Dizziness and giddiness (Light-headedness and vertigo) | 386.9 | 0 | 3 | 970 | 16819 | 1.000 |
| Other disorders of synovium, tendon, and bursa | 727 | 0 | 3 | 904 | 16883 | 1.000 |
| Alcoholic liver damage | 317.11 | 0 | 3 | 1121 | 16534 | 1.000 |
| Aortic valve disease | 394.3 | 0 | 3 | 1039 | 16659 | 1.000 |
| Fracture of clavicle or scapula | 803.3 | 0 | 3 | 1066 | 16645 | 1.000 |
| Other chronic nonalcoholic liver disease | 571.5 | 0 | 3 | 1098 | 16651 | 1.000 |
| Noninflammatory disorders of ovary, fallopian tube, and broad ligament | 619.1 | 0 | 3 | 1169 | 16469 | 1.000 |
| Cholelithiasis with other cholecystitis | 574.12 | 0 | 3 | 902 | 16884 | 1.000 |
| Problems associated with amniotic cavity and membranes | 653 | 0 | 3 | 938 | 16816 | 1.000 |
| Hydronephrosis | 595 | 0 | 3 | 1051 | 16688 | 1.000 |
| Carcinoma in situ of skin | 172.3 | 0 | 3 | 1130 | 16492 | 1.000 |
| Bronchiectasis | 496.3 | 0 | 3 | 1008 | 16761 | 1.000 |
| Endometrial hyperplasia | 621 | 0 | 3 | 1088 | 16639 | 1.000 |
| Genitourinary congenital anomalies | 751 | 0 | 3 | 1049 | 16680 | 1.000 |
| Renal failure NOS | 585.2 | 0 | 3 | 1152 | 16550 | 1.000 |
| Superficial injury without mention of infection | 915 | 0 | 3 | 969 | 16821 | 1.000 |
| Secondary malignancy of brain/spine | 198.5 | 0 | 3 | 1239 | 16320 | 1.000 |
| Other disorders of bladder | 596 | 0 | 3 | 929 | 16861 | 1.000 |
| Diseases of pulp and periapical tissues | 522 | 0 | 3 | 996 | 16761 | 1.000 |
| Psychogenic disorder | 303.3 | 0 | 3 | 1169 | 16405 | 1.000 |
| Other nonmalignant breast conditions | 613 | 0 | 3 | 1000 | 16733 | 1.000 |
| Malignant neoplasm of unspecified male genital organ | 187.1 | 0 | 3 | 977 | 16769 | 1.000 |
| Uterine/Uterovaginal prolapse | 618.2 | 0 | 3 | 911 | 16841 | 1.000 |
| Other abnormal glucose | 250.42 | 0 | 3 | 1305 | 16243 | 1.000 |
| Abnormal sputum | 516 | 0 | 3 | 1013 | 16740 | 1.000 |
| Alcohol-related disorders | 317 | 0 | 3 | 891 | 16898 | 1.000 |
| Poisoning by psychotropic agents | 969 | 0 | 3 | 1016 | 16726 | 1.000 |
| Other symptoms | 1005 | 0 | 3 | 1100 | 16611 | 1.000 |
| Obstetrical/birth trauma | 665 | 0 | 3 | 948 | 16806 | 1.000 |
| Respiratory failure, insufficiency, arrest | 509 | 0 | 3 | 1023 | 16745 | 1.000 |
| Poisoning by analgesics, antipyretics, and antirheumatics | 965 | 0 | 3 | 961 | 16828 | 1.000 |
| Overweight, obesity and other hyperalimentation | 278 | 0 | 3 | 884 | 16906 | 1.000 |
| Other disorders of middle ear and mastoid | 385 | 0 | 3 | 1284 | 16297 | 1.000 |
| Congenital anomalies of genital organs | 751.1 | 0 | 3 | 1151 | 16490 | 1.000 |
| Other disorders of liver | 573 | 0 | 3 | 1078 | 16652 | 1.000 |
| Peripheral vascular disease, unspecified | 443.9 | 0 | 3 | 979 | 16765 | 1.000 |
| Other disorders of testis | 603 | 0 | 3 | 1058 | 16679 | 1.000 |
| Hyperplasia of prostate | 600 | 0 | 3 | 852 | 16893 | 1.000 |
| Acute gastritis | 535.1 | 0 | 3 | 1098 | 16586 | 1.000 |
| Unspecified diffuse connective tissue disease | 709.7 | 0 | 3 | 1002 | 16784 | 1.000 |
| Pain in limb | 773 | 0 | 3 | 1009 | 16781 | 1.000 |
| Disorders of iron metabolism | 275.1 | 0 | 3 | 1145 | 16466 | 1.000 |
| Angina pectoris | 411.3 | 0 | 3 | 972 | 16817 | 1.000 |
| Late pregnancy and failed induction | 645 | 0 | 3 | 921 | 16826 | 1.000 |
| Staphylococcus infections | 041.1 | 0 | 3 | 1013 | 16756 | 1.000 |
| Hypotension NOS | 458.9 | 0 | 3 | 1017 | 16756 | 1.000 |
| Hydrocele | 603.1 | 0 | 3 | 1099 | 16608 | 1.000 |
| Musculoskeletal symptoms referable to limbs | 771 | 0 | 3 | 1102 | 16571 | 1.000 |
| Facial nerve disorders [CN7] | 352.2 | 0 | 3 | 1190 | 16475 | 1.000 |
| Congenital anomalies of female genital organs | 751.11 | 0 | 3 | 1211 | 16357 | 1.000 |
| Diseases and other conditions of the tongue | 529 | 0 | 3 | 1075 | 16652 | 1.000 |
| Alcoholism | 317.1 | 0 | 3 | 966 | 16823 | 1.000 |
| Cholelithiasis and cholecystitis | 574 | 0 | 3 | 868 | 16922 | 1.000 |
| Other disorders of peritoneum | 568 | 0 | 3 | 1004 | 16766 | 1.000 |
| Otitis media | 381.1 | 0 | 3 | 1073 | 16651 | 1.000 |
| Secondary hypothyroidism | 244.1 | 0 | 3 | 1125 | 16570 | 1.000 |
| Viral Enteritis | 008.6 | 0 | 3 | 1262 | 16371 | 1.000 |
| Burns | 1000 | 0 | 3 | 1162 | 16413 | 1.000 |
| Peritoneal adhesions (postoperative) (postinfection) | 568.1 | 0 | 3 | 1006 | 16762 | 1.000 |
| Acute and chronic tonsillitis | 474 | 0 | 3 | 1069 | 16687 | 1.000 |
| Cervicalgia | 761 | 0 | 3 | 1004 | 16735 | 1.000 |
| Migraine | 340 | 0 | 3 | 955 | 16813 | 1.000 |
| Heart failure NOS | 428.2 | 0 | 3 | 990 | 16790 | 1.000 |
| Other complications of pregnancy NEC | 646 | 0 | 3 | 818 | 16936 | 1.000 |
| Benign neoplasm of uterus | 218 | 0 | 3 | 878 | 16875 | 1.000 |
| Articular cartilage disorder | 742.8 | 0 | 3 | 1194 | 16423 | 1.000 |
| Secondary malignant neoplasm of digestive systems | 198.3 | 0 | 3 | 1060 | 16642 | 1.000 |
| Inguinal hernia | 550.1 | 0 | 3 | 907 | 16875 | 1.000 |
| Precordial pain | 418.1 | 0 | 3 | 973 | 16811 | 1.000 |
| Symptoms involving nervous and musculoskeletal systems | 781 | 0 | 3 | 1060 | 16699 | 1.000 |
| Ulcerative colitis | 555.2 | 0 | 3 | 958 | 16813 | 1.000 |
| Sicca syndrome | 709.2 | 0 | 3 | 1065 | 16510 | 1.000 |
| Dyschromia and Vitiligo | 694 | 0 | 3 | 1096 | 16596 | 1.000 |
| Cholecystitis without cholelithiasis | 574.3 | 0 | 3 | 988 | 16785 | 1.000 |
| Benign neoplasm of breast | 610.4 | 0 | 3 | 1060 | 16665 | 1.000 |
| Spondylosis without myelopathy | 721.1 | 0 | 3 | 945 | 16845 | 1.000 |
| Asthma | 495 | 0 | 3 | 906 | 16884 | 1.000 |
| Epilepsy, recurrent seizures, convulsions | 345 | 0 | 3 | 1000 | 16788 | 1.000 |
| Other disorders of male genital organs | 608 | 0 | 3 | 1013 | 16724 | 1.000 |
| Open wounds of head; neck; and trunk | 870 | 0 | 3 | 1026 | 16754 | 1.000 |
| Diseases of the oral soft tissues, excluding lesions specific for gingiva and tongue | 528 | 0 | 3 | 1007 | 16783 | 1.000 |
| Disturbance of skin sensation | 687.4 | 0 | 3 | 964 | 16796 | 1.000 |
| Redundant prepuce and phimosis/BXO | 604.1 | 0 | 3 | 976 | 16761 | 1.000 |
| Cardiomyopathy | 425 | 0 | 3 | 1090 | 16601 | 1.000 |
| Cardiac congenital anomalies | 747.1 | 0 | 3 | 1103 | 16649 | 1.000 |
| Hearing loss | 389 | 0 | 3 | 1002 | 16773 | 1.000 |
| Hypertension complicating pregnancy, childbirth, and the puerperium | 642 | 0 | 3 | 931 | 16797 | 1.000 |
| Retention of urine | 599.2 | 0 | 3 | 907 | 16881 | 1.000 |
| Osteoarthrosis, localized, primary | 740.11 | 0 | 3 | 984 | 16802 | 1.000 |
| Hypertensive heart and/or renal disease | 401.2 | 0 | 3 | 1058 | 16681 | 1.000 |
| Nasal polyps | 471 | 0 | 3 | 917 | 16870 | 1.000 |
| Malignant neoplasm of female breast | 174.11 | 0 | 3 | 809 | 16944 | 1.000 |
| Coagulation defects | 286 | 0 | 3 | 1151 | 16551 | 1.000 |
| Noninflammatory female genital disorders | 619 | 0 | 3 | 888 | 16866 | 1.000 |
| Irregular menstrual cycle | 626.13 | 0 | 3 | 982 | 16763 | 1.000 |
| Disorders of penis | 604 | 0 | 3 | 923 | 16814 | 1.000 |
| Polyp of corpus uteri | 622.1 | 0 | 3 | 846 | 16908 | 1.000 |
| Unspecified monoarthritis | 716.2 | 0 | 3 | 944 | 16841 | 1.000 |
| Menopausal and postmenopausal disorders | 627 | 0 | 3 | 874 | 16874 | 1.000 |
| Cancer of prostate | 185 | 0 | 3 | 836 | 16902 | 1.000 |
| Other aneurysm | 442 | 0 | 3 | 1011 | 16704 | 1.000 |
| Heartburn | 530.9 | 0 | 3 | 1061 | 16683 | 1.000 |
| Fracture of hand or wrist | 804 | 0 | 3 | 1029 | 16749 | 1.000 |
| Congenital coagulation defects | 286.1 | 0 | 3 | 1206 | 16419 | 1.000 |
| Congenital anomalies of great vessels | 747.13 | 0 | 3 | 1120 | 16577 | 1.000 |
| Peptic ulcer (excl. esophageal) | 531 | 0 | 3 | 902 | 16888 | 1.000 |
| Spondylosis and allied disorders | 721 | 0 | 3 | 963 | 16827 | 1.000 |
| Large cell lymphoma | 202.24 | 0 | 3 | 1169 | 16397 | 1.000 |
| Generalized convulsive epilepsy | 345.11 | 0 | 3 | 1217 | 16334 | 1.000 |
| Abnormal findings on exam of gastrointestinal tract/ abdominal area | 564.8 | 0 | 3 | 1004 | 16721 | 1.000 |
| Other open wound of head and face | 870.3 | 0 | 3 | 1001 | 16768 | 1.000 |
| Cancer of urinary organs (incl. kidney and bladder) | 189 | 0 | 3 | 950 | 16836 | 1.000 |
| Agorophobia, social phobia, and panic disorder | 300.12 | 0 | 3 | 1246 | 16389 | 1.000 |
| Cervical cancer | 180.1 | 0 | 3 | 1053 | 16665 | 1.000 |
| Other disorders of metabolism | 277 | 0 | 3 | 1159 | 16490 | 1.000 |
| Other and unspecified disc disorder | 722.9 | 0 | 3 | 971 | 16819 | 1.000 |
| Erythematous conditions | 695 | 0 | 3 | 1005 | 16745 | 1.000 |
| Calculus of kidney | 594.1 | 0 | 3 | 1009 | 16773 | 1.000 |
| Intracerebral hemorrhage | 430.2 | 0 | 3 | 1209 | 16389 | 1.000 |
| Obstruction of bile duct | 575.2 | 0 | 3 | 1206 | 16440 | 1.000 |
| Hypertensive chronic kidney disease | 401.22 | 0 | 3 | 1060 | 16659 | 1.000 |
| Vascular insufficiency of intestine | 441 | 0 | 3 | 1183 | 16359 | 1.000 |
| Convulsions | 345.3 | 0 | 3 | 1069 | 16673 | 1.000 |
| Phlebitis and thrombophlebitis of lower extremities | 451.2 | 0 | 3 | 966 | 16821 | 1.000 |
| Breast cancer [female] | 174.1 | 0 | 3 | 828 | 16925 | 1.000 |
| Viral hepatitis | 070 | 0 | 3 | 1247 | 16385 | 1.000 |
| Atherosclerosis of the extremities | 440.2 | 0 | 3 | 1100 | 16510 | 1.000 |
| Chronic glomerulonephritis, NOS | 580.14 | 0 | 3 | 1137 | 16476 | 1.000 |
| Cerebral ischemia | 433.3 | 0 | 3 | 964 | 16808 | 1.000 |
| Disorders of fluid, electrolyte, and acid-base balance | 276 | 0 | 3 | 946 | 16844 | 1.000 |
| Secondary malignant neoplasm | 198 | 0 | 3 | 871 | 16918 | 1.000 |
| Renal colic | 594.8 | 0 | 3 | 994 | 16777 | 1.000 |
| Elevated blood pressure reading without diagnosis of hypertension | 402 | 0 | 3 | 1071 | 16649 | 1.000 |
| Cholelithiasis with acute cholecystitis | 574.11 | 0 | 3 | 1049 | 16676 | 1.000 |
| Fracture of radius and ulna | 803.2 | 0 | 3 | 922 | 16867 | 1.000 |
| Nontoxic uninodular goiter | 241.1 | 0 | 3 | 1183 | 16379 | 1.000 |
| Other and unspecified disorders of back | 724 | 0 | 3 | 1092 | 16653 | 1.000 |
| Memory loss | 292.3 | 0 | 3 | 1173 | 16473 | 1.000 |
| Hypertrophy of breast (Gynecomastia) | 612.2 | 0 | 3 | 1025 | 16684 | 1.000 |
| Other disorders of gallbladder | 575.7 | 0 | 3 | 1084 | 16620 | 1.000 |
| Primary/intrinsic cardiomyopathies | 425.1 | 0 | 3 | 1097 | 16590 | 1.000 |
| Fracture of ankle and foot | 801 | 0 | 3 | 1025 | 16721 | 1.000 |
| Symptoms involving digestive system | 561 | 0 | 3 | 869 | 16914 | 1.000 |
| Fever of unknown origin | 783 | 0 | 3 | 957 | 16833 | 1.000 |
| Cancer, suspected or other | 195 | 0 | 3 | 861 | 16928 | 1.000 |
| Epistaxis or throat hemorrhage | 477 | 0 | 3 | 1093 | 16669 | 1.000 |
| Other symptoms/disorders or the urinary system | 599 | 0 | 3 | 883 | 16907 | 1.000 |
| Cough | 512.8 | 0 | 3 | 1029 | 16746 | 1.000 |
| Other local infections of skin and subcutaneous tissue | 686 | 0 | 3 | 1067 | 16651 | 1.000 |
| Malignant neoplasm of uterus | 182 | 0 | 3 | 1077 | 16621 | 1.000 |
| Gram negative septicemia | 038.1 | 0 | 3 | 1219 | 16429 | 1.000 |
| Type 2 diabetes with neurological manifestations | 250.24 | 0 | 3 | 1254 | 16322 | 1.000 |
| Postmenopausal bleeding | 627.1 | 0 | 3 | 841 | 16906 | 1.000 |
| Cardiac and circulatory congenital anomalies | 747 | 0 | 3 | 1102 | 16652 | 1.000 |
| Intracranial hemorrhage | 430 | 0 | 3 | 1111 | 16610 | 1.000 |
| Cervical intraepithelial neoplasia [CIN] [Cervical dysplasia] | 180.3 | 0 | 3 | 953 | 16793 | 1.000 |
| Electrolyte imbalance | 276.1 | 0 | 3 | 968 | 16803 | 1.000 |
| Acute pancreatitis | 577.1 | 0 | 3 | 1073 | 16666 | 1.000 |
| Nonspecific abnormal findings in stool contents | 579.8 | 0 | 3 | 1077 | 16661 | 1.000 |
| Edema | 782.3 | 0 | 3 | 1028 | 16689 | 1.000 |
| Breast cancer | 174 | 0 | 3 | 839 | 16949 | 1.000 |
| Psoriatic arthropathy | 696.42 | 0 | 3 | 1216 | 16392 | 1.000 |
| Other specified benign mammary dysplasias | 610.8 | 0 | 3 | 1216 | 16437 | 1.000 |
| Calculus of lower urinary tract | 594.2 | 0 | 3 | 1144 | 16528 | 1.000 |
| Hypothyroidism NOS | 244.4 | 0 | 3 | 880 | 16910 | 1.000 |
| Inflammation of eyelids | 371.3 | 0 | 3 | 996 | 16780 | 1.000 |
| Abnormal movement | 350 | 0 | 3 | 1053 | 16696 | 1.000 |
| Muscular dystrophies and other myopathies | 359 | 0 | 3 | 1247 | 16318 | 1.000 |
| Gingival and periodontal diseases | 523 | 0 | 3 | 1057 | 16693 | 1.000 |
| Fracture of humerus | 803.1 | 0 | 3 | 1119 | 16594 | 1.000 |
| Pelvic peritoneal adhesions, female (postoperative) (postinfection) | 614.1 | 0 | 3 | 988 | 16766 | 1.000 |
| Other disorders of intestine | 569 | 0 | 3 | 949 | 16839 | 1.000 |
| Disorders of vitreous body | 379.2 | 0 | 3 | 1047 | 16647 | 1.000 |
| Other disorders of biliary tract | 575.8 | 0 | 3 | 1058 | 16619 | 1.000 |
| Chronic cystitis | 592.12 | 0 | 3 | 1110 | 16567 | 1.000 |
| Emphysema | 496.1 | 0 | 3 | 1039 | 16703 | 1.000 |
| Fracture of foot | 801.1 | 0 | 3 | 1043 | 16688 | 1.000 |
| Actinic keratosis | 702.1 | 0 | 3 | 1036 | 16725 | 1.000 |
| Gout and other crystal arthropathies | 274 | 0 | 3 | 931 | 16858 | 1.000 |
| Diseases of esophagus | 530 | 0 | 3 | 902 | 16888 | 1.000 |
| Torus fracture | 823 | 0 | 3 | 1153 | 16578 | 1.000 |
| Unspecified polyarthropathy or polyarthritis | 716.1 | 0 | 3 | 943 | 16833 | 1.000 |
| Acute pain | 338.1 | 0 | 3 | 1223 | 16355 | 1.000 |
| Cancer of esophagus | 150 | 0 | 3 | 1054 | 16559 | 1.000 |
| Other disorders of prostate | 602 | 0 | 3 | 1045 | 16691 | 1.000 |
| Rheumatism, unspecified and fibrositis | 729.1 | 0 | 3 | 1081 | 16584 | 1.000 |
| Hypertension | 401 | 0 | 3 | 876 | 16914 | 1.000 |
| Psoriasis | 696.4 | 0 | 3 | 1102 | 16651 | 1.000 |
| Lupus (localized and systemic) | 695.4 | 0 | 3 | 1181 | 16395 | 1.000 |
| Vaginal enterocele, congenital or acquired | 618.6 | 0 | 3 | 1200 | 16396 | 1.000 |
| Hypoglycemia | 251.1 | 0 | 3 | 1091 | 16592 | 1.000 |
| Hypovolemia | 276.5 | 0 | 3 | 1043 | 16719 | 1.000 |
| Endocarditis | 420.3 | 0 | 3 | 1228 | 16374 | 1.000 |
| Transient cerebral ischemia | 433.31 | 0 | 3 | 1034 | 16722 | 1.000 |
| Lipoma | 214 | 0 | 3 | 952 | 16838 | 1.000 |
| Essential hypertension | 401.1 | 0 | 3 | 874 | 16916 | 1.000 |
| Bursitis | 726.3 | 0 | 3 | 1120 | 16557 | 1.000 |
| Other symptoms of respiratory system | 512 | 0 | 3 | 932 | 16858 | 1.000 |
| Dislocation | 830 | 0 | 3 | 1017 | 16736 | 1.000 |
| Hyperhidrosis | 705.8 | 0 | 3 | 1232 | 16391 | 1.000 |
| Palpitations | 427.9 | 0 | 3 | 977 | 16809 | 1.000 |
| Abnormal results of function study of liver | 573.7 | 0 | 3 | 978 | 16801 | 1.000 |
| Disorders of conjunctiva | 372 | 0 | 3 | 1161 | 16495 | 1.000 |
| Femoral hernia | 550.3 | 0 | 3 | 1178 | 16469 | 1.000 |
| Hemorrhage in early pregnancy | 636.3 | 0 | 3 | 929 | 16784 | 1.000 |
| Hypopotassemia | 276.14 | 0 | 3 | 1043 | 16657 | 1.000 |
| Periodontitis (acute or chronic) | 523.3 | 0 | 3 | 1070 | 16656 | 1.000 |
| Epiphora | 375.2 | 0 | 3 | 1154 | 16489 | 1.000 |
| Varicose veins | 454 | 0 | 3 | 883 | 16905 | 1.000 |
| Acute periodontitis | 523.31 | 0 | 3 | 1133 | 16530 | 1.000 |
| Fracture of lower limb | 800 | 0 | 3 | 920 | 16869 | 1.000 |
| Blindness and low vision | 367.9 | 0 | 3 | 1246 | 16299 | 1.000 |
| Gastric ulcer | 531.2 | 0 | 3 | 950 | 16834 | 1.000 |
| Cancer of larynx, pharynx, nasal cavities | 149 | 0 | 3 | 1172 | 16433 | 1.000 |
| Other intestinal obstruction | 560.4 | 0 | 3 | 934 | 16835 | 1.000 |
| Anal and rectal conditions | 565 | 0 | 3 | 871 | 16917 | 1.000 |
| Periapical abscess | 522.5 | 0 | 3 | 1052 | 16675 | 1.000 |
| Hyperpotassemia | 276.13 | 0 | 3 | 1186 | 16443 | 1.000 |
| Tobacco use disorder | 318 | 0 | 3 | 906 | 16883 | 1.000 |
| Cancer of other male genital organs | 187 | 0 | 3 | 975 | 16771 | 1.000 |
| E. coli | 041.4 | 0 | 3 | 987 | 16774 | 1.000 |
| Derangement of joint, non-traumatic | 742 | 0 | 3 | 928 | 16848 | 1.000 |
| Cystitis | 592.1 | 0 | 3 | 1046 | 16730 | 1.000 |
| Glaucoma | 365 | 0 | 3 | 966 | 16821 | 1.000 |
| Intestinal infection | 008 | 0 | 3 | 909 | 16881 | 1.000 |
| Neoplasm of uncertain behavior | 199 | 0 | 3 | 1069 | 16645 | 1.000 |
| Cardiac conduction disorders | 426 | 0 | 3 | 955 | 16832 | 1.000 |
| Pleurisy; pleural effusion | 507 | 0 | 3 | 994 | 16794 | 1.000 |
| Hemorrhage during pregnancy; childbirth and postpartum | 635 | 0 | 3 | 923 | 16830 | 1.000 |
| Cystitis and urethritis | 592 | 0 | 3 | 1042 | 16736 | 1.000 |
| Other biliary tract disease | 575 | 0 | 3 | 951 | 16827 | 1.000 |
| Acquired absence of breast | 175 | 0 | 3 | 1030 | 16715 | 1.000 |
| Disease of tricuspid valve | 394.7 | 0 | 3 | 1127 | 16556 | 1.000 |
| Cerebral artery occlusion, with cerebral infarction | 433.21 | 0 | 3 | 1042 | 16670 | 1.000 |
| Retinal detachment with retinal defect | 361.1 | 0 | 3 | 1023 | 16713 | 1.000 |
| Noninflammatory disorders of cervix | 619.3 | 0 | 3 | 956 | 16797 | 1.000 |
| Osteoporosis | 743.1 | 0 | 3 | 897 | 16893 | 1.000 |
| Intestinal obstruction without mention of hernia | 560 | 0 | 3 | 912 | 16863 | 1.000 |
| Hallux valgus (Bunion) | 735.3 | 0 | 3 | 911 | 16879 | 1.000 |
| Acute pharyngitis | 465.2 | 0 | 3 | 1099 | 16575 | 1.000 |
| Pilonidal cyst | 686.3 | 0 | 3 | 1152 | 16407 | 1.000 |
| Hemiplegia | 342 | 0 | 3 | 1124 | 16569 | 1.000 |
| Other acute and subacute forms of ischemic heart disease | 411.9 | 0 | 3 | 986 | 16729 | 1.000 |
| Other disorders of thyroid | 246 | 0 | 3 | 1131 | 16429 | 1.000 |
| Lymphoid leukemia, chronic | 204.12 | 0 | 3 | 1212 | 16358 | 1.000 |
| Dysmenorrhea | 626.2 | 0 | 3 | 1029 | 16711 | 1.000 |
| Acute upper respiratory infections of multiple or unspecified sites | 465 | 0 | 3 | 1000 | 16763 | 1.000 |
| Hemorrhage of gastrointestinal tract | 578.9 | 0 | 3 | 952 | 16835 | 1.000 |
| Septicemia | 038 | 0 | 3 | 1021 | 16759 | 1.000 |
| Lymphoid leukemia | 204.1 | 0 | 3 | 1237 | 16355 | 1.000 |
| Dyspareunia | 625.1 | 0 | 3 | 1080 | 16629 | 1.000 |
| Cardiac arrest and ventricular fibrillation | 427.4 | 0 | 3 | 1109 | 16566 | 1.000 |
| Sciatica | 764 | 0 | 3 | 1030 | 16728 | 1.000 |
| Known or suspected fetal abnormality affecting management of mother | 655 | 0 | 3 | 865 | 16889 | 1.000 |
| Cancer within the respiratory system | 165 | 0 | 3 | 956 | 16786 | 1.000 |
| Disorders of external ear | 380 | 0 | 3 | 1232 | 16324 | 1.000 |
| Pneumonitis due to inhalation of food or vomitus | 501 | 0 | 3 | 1188 | 16364 | 1.000 |
| Other disorders of urethra and urinary tract | 597 | 0 | 3 | 980 | 16807 | 1.000 |
| Bacterial enteritis | 008.5 | 0 | 3 | 1056 | 16697 | 1.000 |
| Myopia | 367.1 | 0 | 3 | 1104 | 16568 | 1.000 |
| Osteoporosis NOS | 743.11 | 0 | 3 | 906 | 16884 | 1.000 |
| Prurigo and Lichen | 695.7 | 0 | 3 | 1147 | 16516 | 1.000 |
| Dysphagia | 532 | 0 | 3 | 913 | 16872 | 1.000 |
| Suppurative and unspecified otitis media | 381.11 | 0 | 3 | 1189 | 16429 | 1.000 |
| Neurological disorders | 292 | 0 | 3 | 983 | 16803 | 1.000 |
| Megaloblastic anemia | 281.1 | 0 | 3 | 1108 | 16570 | 1.000 |
| Other deficiency anemia | 281 | 0 | 3 | 1107 | 16573 | 1.000 |
| Melanomas of skin | 172.11 | 0 | 3 | 986 | 16783 | 1.000 |
| Benign neoplasm of skin | 216 | 0 | 3 | 877 | 16913 | 1.000 |
| Melanomas of skin, dx or hx | 172.1 | 0 | 3 | 986 | 16783 | 1.000 |
| Parkinson's disease | 332 | 0 | 3 | 1140 | 16511 | 1.000 |
| Nerve root and plexus disorders | 353 | 0 | 3 | 1115 | 16558 | 1.000 |
| Psoriasis vulgaris | 696.41 | 0 | 3 | 1101 | 16627 | 1.000 |
| Calculus of ureter | 594.3 | 0 | 3 | 975 | 16785 | 1.000 |
| Secondary malignancy of bone | 198.6 | 0 | 3 | 1056 | 16683 | 1.000 |
| Bladder neck obstruction | 596.1 | 0 | 3 | 1029 | 16711 | 1.000 |
| Schizophrenia and other psychotic disorders | 295 | 0 | 3 | 1214 | 16412 | 1.000 |
| Psoriasis and related disorders | 696 | 0 | 3 | 1104 | 16651 | 1.000 |
| Symptoms and disorders of the joints | 741 | 0 | 3 | 994 | 16791 | 1.000 |
| Candidiasis | 112 | 0 | 3 | 1004 | 16743 | 1.000 |
| Gastritis and duodenitis | 535 | 0 | 3 | 880 | 16910 | 1.000 |
| Cardiomegaly | 416 | 0 | 3 | 1004 | 16747 | 1.000 |
| Congestive heart failure; nonhypertensive | 428 | 0 | 3 | 978 | 16812 | 1.000 |
| Hyperlipidemia | 272.1 | 0 | 3 | 922 | 16868 | 1.000 |
| Osteoarthritis; localized | 740.1 | 0 | 3 | 920 | 16869 | 1.000 |
| Cholelithiasis | 574.1 | 0 | 3 | 845 | 16943 | 1.000 |
| Stricture and stenosis of esophagus | 530.3 | 0 | 3 | 992 | 16754 | 1.000 |
| Acquired spondylolisthesis | 738.4 | 0 | 3 | 1081 | 16627 | 1.000 |
| Abnormal glucose | 250.4 | 0 | 3 | 1284 | 16348 | 1.000 |
| Nonspecific abnormal findings on radiological and other examination of other intrathoracic organs (echocardiogram, etc) | 793.2 | 0 | 3 | 1256 | 16310 | 1.000 |
| Other non-epithelial cancer of skin | 172.2 | 0 | 3 | 894 | 16896 | 1.000 |
| Other diseases of lung | 510 | 0 | 3 | 1142 | 16521 | 1.000 |
| Secondary malignancy of lymph nodes | 198.1 | 0 | 3 | 951 | 16829 | 1.000 |
| Enthesopathy | 726.1 | 0 | 3 | 913 | 16873 | 1.000 |
| Corneal opacity and other disorders of cornea | 364 | 0 | 3 | 1199 | 16404 | 1.000 |
| Altered mental status | 292.4 | 0 | 3 | 1051 | 16692 | 1.000 |
| Disorders of lipoid metabolism | 272 | 0 | 3 | 923 | 16867 | 1.000 |
| Premature beats | 427.6 | 0 | 3 | 1237 | 16331 | 1.000 |
| Chronic renal failure [CKD] | 585.3 | 0 | 3 | 992 | 16750 | 1.000 |
| Obesity | 278.1 | 0 | 3 | 892 | 16898 | 1.000 |
| Osteomyelitis | 710.1 | 0 | 3 | 1271 | 16332 | 1.000 |
| Respiratory insufficiency | 509.2 | 0 | 3 | 1026 | 16721 | 1.000 |
| Congestive heart failure (CHF) NOS | 428.1 | 0 | 3 | 1014 | 16722 | 1.000 |
| Otitis media and Eustachian tube disorders | 381 | 0 | 3 | 1052 | 16688 | 1.000 |
| Other disorders of tympanic membrane | 384 | 0 | 3 | 1107 | 16590 | 1.000 |
| Hemorrhage or hematoma complicating a procedure | 850 | 0 | 3 | 996 | 16793 | 1.000 |
| Hemangioma and lymphangioma, any site | 228 | 0 | 3 | 1006 | 16718 | 1.000 |
| Mucous polyp of cervix | 622.2 | 0 | 3 | 901 | 16852 | 1.000 |
| Hallux rigidus | 735.23 | 0 | 3 | 1069 | 16648 | 1.000 |
| Pericarditis | 420.2 | 0 | 3 | 1031 | 16680 | 1.000 |
| Carditis | 420 | 0 | 3 | 1016 | 16725 | 1.000 |
| Hypercholesterolemia | 272.11 | 0 | 3 | 893 | 16897 | 1.000 |
| Hypothyroidism | 244 | 0 | 3 | 891 | 16899 | 1.000 |
| Vitamin deficiency | 261 | 0 | 3 | 1055 | 16651 | 1.000 |
| Symptoms concerning nutrition, metabolism, and development | 1002 | 0 | 3 | 962 | 16828 | 1.000 |
| Hematemesis | 578.1 | 0 | 3 | 1060 | 16689 | 1.000 |
| Chondrocalcinosis | 274.21 | 0 | 3 | 1202 | 16375 | 1.000 |
| Skin cancer | 172 | 0 | 3 | 853 | 16937 | 1.000 |
| Complication of internal orthopedic device | 858 | 0 | 3 | 980 | 16793 | 1.000 |
| Anal and rectal polyp | 565.1 | 0 | 3 | 943 | 16845 | 1.000 |
| Multiple sclerosis | 335 | 0 | 3 | 1091 | 16617 | 1.000 |
| Other acquired musculoskeletal deformity | 738 | 0 | 3 | 1050 | 16676 | 1.000 |
| Bronchopneumonia and lung abscess | 480.5 | 0 | 3 | 1170 | 16452 | 1.000 |
| Fluid overload | 276.6 | 0 | 3 | 1241 | 16279 | 1.000 |
| Uterine leiomyoma | 218.1 | 0 | 3 | 889 | 16864 | 1.000 |
| Chronic tonsillitis and adenoiditis | 474.2 | 0 | 3 | 1075 | 16627 | 1.000 |
| Other disorders of the kidney and ureters | 586 | 0 | 3 | 966 | 16812 | 1.000 |
| Pain and other symptoms associated with female genital organs | 625 | 0 | 3 | 928 | 16824 | 1.000 |
| Osteomyelitis, periostitis, and other infections involving bone | 710 | 0 | 3 | 1263 | 16344 | 1.000 |
| Labyrinthitis | 386.3 | 0 | 3 | 1196 | 16431 | 1.000 |
| Umbilical cord complications during labor and delivery | 663 | 0 | 3 | 901 | 16749 | 1.000 |
| Endometriosis | 615 | 0 | 3 | 876 | 16877 | 1.000 |
| Arrhythmia (cardiac) NOS | 427.5 | 0 | 3 | 1078 | 16585 | 1.000 |
| Cancer of bronchus; lung | 165.1 | 0 | 3 | 986 | 16743 | 1.000 |
| Varicose veins of lower extremity | 454.1 | 0 | 3 | 875 | 16913 | 1.000 |
| Disorders of adrenal glands | 255 | 0 | 3 | 1162 | 16471 | 1.000 |
| Type 2 diabetes with ophthalmic manifestations | 250.23 | 0 | 3 | 1135 | 16546 | 1.000 |
| Cardiac arrest | 427.42 | 0 | 3 | 1099 | 16541 | 1.000 |
| Disorders of lacrimal system | 375 | 0 | 3 | 987 | 16760 | 1.000 |
| Pancreatic cancer | 157 | 0 | 3 | 1209 | 16368 | 1.000 |
| Fracture of patella | 800.4 | 0 | 3 | 1223 | 16408 | 1.000 |
| Cyst of kidney, acquired | 586.2 | 0 | 3 | 1059 | 16641 | 1.000 |
| Disorders of esophageal motility | 530.5 | 0 | 3 | 1144 | 16470 | 1.000 |
| Duodenitis | 535.6 | 0 | 3 | 891 | 16896 | 1.000 |
| Poisoning/allergy of sulfonamides | 961.1 | 0 | 3 | 1060 | 16643 | 1.000 |
| Other disorders of arteries and arterioles | 447 | 0 | 3 | 1076 | 16610 | 1.000 |
| Late effects of cerebrovascular disease | 433.8 | 0 | 3 | 1104 | 16584 | 1.000 |
| Ankylosing spondylitis | 715.2 | 0 | 3 | 1263 | 16306 | 1.000 |
