# Supplementary figures and images for: Identification of potential candidate genes and pathways in atrioventricular nodal reentry tachycardia by whole‐exome sequencing
Source: Clin Transl Med. 2020 Apr 30;10(1):238–57. doi: 10.1002/ctm2.25 (PMC7240861; doi:10.1002/ctm2.25)

**S14 Average sequencing cover of exons**


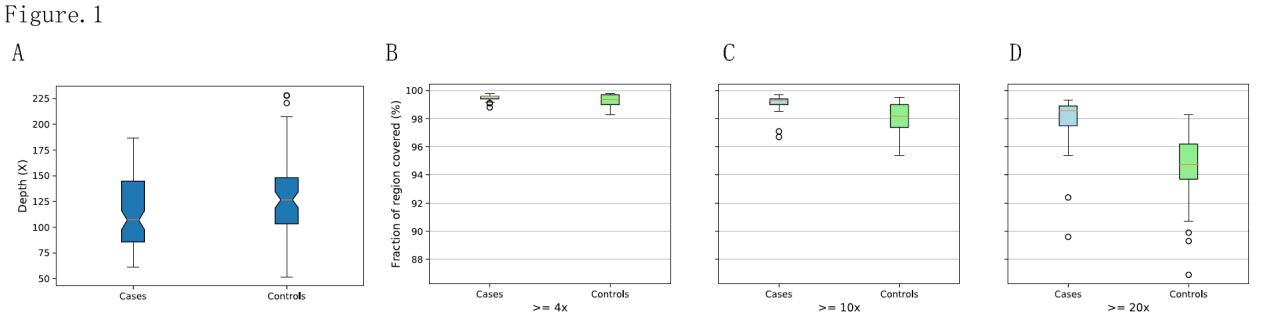

Supplement: Supplementary file 11 — Supporting Information S10 [file CTM2-10-238-s003.docx]
