# Supplementary material for: Identification of potential candidate genes and pathways in atrioventricular nodal reentry tachycardia by whole‐exome sequencing
Source: Clin Transl Med. 2020 Apr 30;10(1):238–57. doi: 10.1002/ctm2.25 (PMC7240861; doi:10.1002/ctm2.25)
Supplement: Supplementary file 12 — Supporting Information S11 [file CTM2-10-238-s004.docx]

**S15: Hardy-Weinberg equilibrium, dendrogram and Quantile-quantile plot in GWAS.**


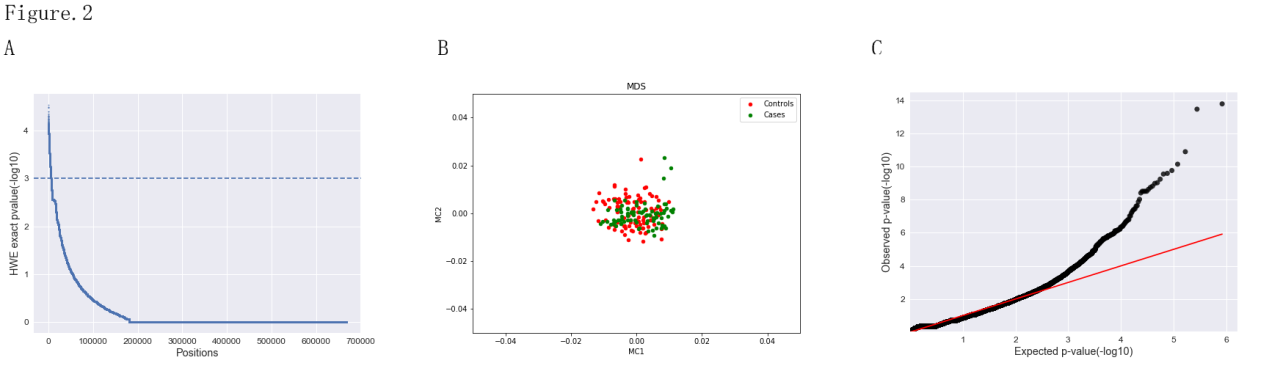


Figure lengends: A, Hardy-Weinberg equilibrium in GWAS; B, Dendrogram in GWAS; C, Quantile- quantile plot in GWAS.
