# Supplementary material for: Identification of potential candidate genes and pathways in atrioventricular nodal reentry tachycardia by whole‐exome sequencing
Source: Clin Transl Med. 2020 Apr 30;10(1):238–57. doi: 10.1002/ctm2.25 (PMC7240861; doi:10.1002/ctm2.25)
Supplement: Supplementary file 13 — Supporting Information S12 [file CTM2-10-238-s005.docx]

**S16: Quantile-quantile and Manhtton plots in pathway enrichment (MAF <0.01 and MAF<0.001)**


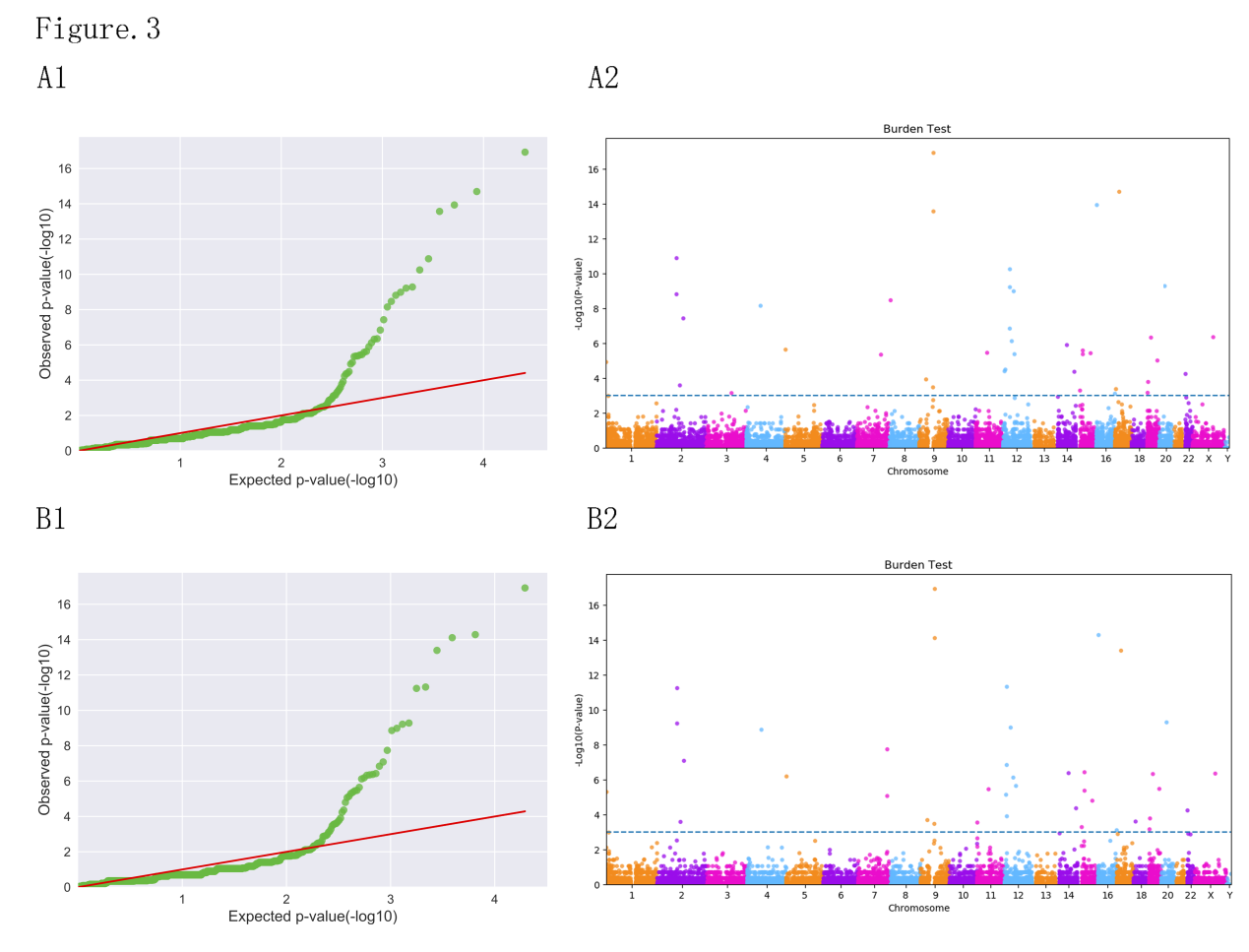


Figure legends: A1, Quantile-quantile plot in pathway enrichment (MAF <0.01); A2, Manhatton plot in pathway enrichment(MAF<0.01); B1, Quantile-quantile plot in pathway enrichment (MAF <0.001); B2, Manhatton plot in pathway enrichment(MAF<0.001).
