# Supplementary material for: Identification of potential candidate genes and pathways in atrioventricular nodal reentry tachycardia by whole‐exome sequencing
Source: Clin Transl Med. 2020 Apr 30;10(1):238–57. doi: 10.1002/ctm2.25 (PMC7240861; doi:10.1002/ctm2.25)
Supplement: Supplementary file 14 — Supporting Information S13 [file CTM2-10-238-s014.docx]

**S6: Gene-based burden analysis (MAF< 0.001)**

| **Gene Name** | **OR** | **P** value | **Case**(N) | **Control**(N) |
| --- | --- | --- | --- | --- |
| LOC101929583 | 55.23809524 | 1.18589E-17 | 80 | 42 |
| TPSB2 | 27.78947368 | 5.19089E-15 | 44 | 4 |
| ANKRD20A4 | 30.91304348 | 7.74155E-15 | 79 | 46 |
| CCDC144NL | 12.10784314 | 4.05202E-14 | 65 | 24 |
| TAS2R43 | 56.28813559 | 4.79522E-12 | 81 | 59 |
| ANKRD36B | inf | 5.72439E-12 | 82 | 63 |
| FAM182B | 18.29943503 | 5.21992E-10 | 79 | 59 |
| ANKRD36 | 43.61538462 | 6.06128E-10 | 81 | 65 |
| TMTC1 | 11.2327044 | 1.03738E-09 | 76 | 53 |
| YTHDC1 | 6.9 | 1.37883E-09 | 62 | 31 |
| TRBV11-2 | 10.70169492 | 1.83108E-08 | 77 | 59 |
| POTEI | 8.802259887 | 8.2918E-08 | 76 | 59 |
| KLRC2 | 6.909465021 | 1.44097E-07 | 73 | 54 |
| GOLGA8A | 31.93548387 | 3.74582E-07 | 20 | 1 |
| WDR89 | 7.36 | 4.20805E-07 | 32 | 8 |
| TCP11X1 | 13.37931034 | 4.46145E-07 | 24 | 3 |
| NXNL1 | inf | 4.72376E-07 | 17 | 0 |
| LOC100506688 | 9.327272727 | 6.53113E-07 | 27 | 5 |
| GXYLT1 | 5.723842196 | 7.56635E-07 | 71 | 53 |
| OR6C76 | 6.566820276 | 2.27662E-06 | 75 | 62 |
| LMTK3 | 6.888888889 | 3.36945E-06 | 28 | 7 |
| OR8U1 | 14.05405405 | 3.53803E-06 | 80 | 74 |
| GOLGA8B | 5.448979592 | 4.27322E-06 | 33 | 11 |
| FAAP20 | 5.194444444 | 5.0527E-06 | 34 | 12 |
| PEX5 | 4.772727273 | 7.29974E-06 | 70 | 55 |
| TRBV3-1 | 3.915584416 | 8.61693E-06 | 54 | 33 |
| GOLGA6B | 3.821978022 | 1.60801E-05 | 47 | 26 |
| EVL | inf | 4.35193E-05 | 12 | 0 |
| LOC101929674 | 3.39408867 | 5.79242E-05 | 53 | 35 |
| TAS2R46 | 3.193548387 | 0.00012515 | 51 | 34 |
| C19orf71 | 4.05 | 0.000166535 | 72 | 64 |
| AQP7 | 4.434697856 | 0.000206539 | 25 | 9 |
| PIEZO2 | inf | 0.000250389 | 10 | 0 |
| WASH2P | 3.185758514 | 0.000262081 | 63 | 51 |
| MUC6 | inf | 0.000285624 | 82 | 87 |
| LOC100996643 | 10.08823529 | 0.000339363 | 14 | 2 |
| POTEB2 | 4.573770492 | 0.000520143 | 21 | 7 |
| CNN2 | 5.5 | 0.000697119 | 78 | 78 |
| LOC101927910 | 2.915151515 | 0.000787292 | 37 | 22 |
| HNRNPCL1 | inf | 0.001060149 | 82 | 89 |
| OR11H2 | 3.185185185 | 0.001193839 | 28 | 14 |
| RIMBP3 | 3.100628931 | 0.001253397 | 29 | 15 |
| GP1BA | 3.028846154 | 0.001301411 | 30 | 16 |
| PATZ1 | 3.707407407 | 0.001367951 | 22 | 9 |
| MUC2 | 2.640232108 | 0.002298556 | 35 | 22 |
| ANKRD36C | 3.783549784 | 0.003049523 | 76 | 77 |
| ANKRD20A1 | 2.393650794 | 0.003100989 | 52 | 42 |
| CNOT1 | 5.393574297 | 0.003174219 | 79 | 83 |
| PCDHGA10 | 3.034482759 | 0.003211253 | 24 | 12 |
| GOLGA8O | 2.474426808 | 0.003440186 | 61 | 54 |
| OR2T2 | 4.253731343 | 0.004264689 | 15 | 5 |
| LOC100996273 | 3.261648746 | 0.00434813 | 20 | 9 |
| SPATA31A7 | 2.94084507 | 0.004536582 | 72 | 71 |
| FRG2B | 5.542857143 | 0.004704616 | 12 | 3 |
| LOC283710 | 2.233716475 | 0.006163142 | 53 | 45 |
| GOLGA6L6 | 2.577272727 | 0.006234185 | 27 | 16 |
| MUC5B | 2.37037037 | 0.006957721 | 64 | 60 |
| EME1 | inf | 0.007539326 | 6 | 0 |
| DISP1 | inf | 0.007539326 | 6 | 0 |
| LOC285556 | inf | 0.007539326 | 6 | 0 |
| DDX60 | 10.7027027 | 0.007810276 | 8 | 1 |
| WDPCP | 10.7027027 | 0.007810276 | 8 | 1 |
| C11orf95 | 10.7027027 | 0.007810276 | 8 | 1 |
| TBC1D3H | 10.7027027 | 0.007810276 | 8 | 1 |
| TTLL10 | 10.7027027 | 0.007810276 | 8 | 1 |
| LOC728026 | 2.903225806 | 0.008114056 | 20 | 10 |
| SFTPA2 | 5.009389671 | 0.009054857 | 11 | 3 |
| ATXN3 | 2.273224044 | 0.010071233 | 64 | 61 |
| TBC1D3B | 3.021978022 | 0.010521466 | 75 | 78 |
| TNXB | 3.220779221 | 0.010522937 | 16 | 7 |
| TBC1D3 | 3.220779221 | 0.010522937 | 16 | 7 |
| FCGBP | 2.119815668 | 0.010871814 | 40 | 31 |
| AHNAK | 4.114285714 | 0.011640255 | 12 | 4 |
| PRAMEF20 | 6.04109589 | 0.012564457 | 9 | 2 |
| ANKRD20A2 | 3.380487805 | 0.012927185 | 77 | 82 |
| CNOT2 | 2.119476268 | 0.013426222 | 35 | 26 |
| TRBV10-1 | inf | 0.013708005 | 82 | 93 |
| BCR | 2.285714286 | 0.015662897 | 68 | 68 |
| SSTR1 | 2.266806723 | 0.015911982 | 26 | 17 |
| ZNF880 | 2.266806723 | 0.015911982 | 26 | 17 |
| SREK1IP1 | 3.225490196 | 0.016112989 | 14 | 6 |
| PRB3 | 9.24 | 0.01627809 | 7 | 1 |
| CCDC180 | 9.24 | 0.01627809 | 7 | 1 |
| CHD7 | 9.24 | 0.01627809 | 7 | 1 |
| ROBO1 | 9.24 | 0.01627809 | 7 | 1 |
| PPP1R37 | 9.24 | 0.01627809 | 7 | 1 |
| OR2T35 | 2.119930975 | 0.016462127 | 63 | 61 |
| RGPD2 | 2.021886792 | 0.016910493 | 57 | 53 |
| LMO7 | inf | 0.017330658 | 5 | 0 |
| COL4A3 | inf | 0.017330658 | 5 | 0 |
| CFD | inf | 0.017330658 | 5 | 0 |
| TMEM255B | inf | 0.017330658 | 5 | 0 |
| LEMD3 | inf | 0.017330658 | 5 | 0 |
| TRIM66 | inf | 0.017330658 | 5 | 0 |
| PRAMEF15 | inf | 0.017330658 | 5 | 0 |
| CCDC114 | inf | 0.017330658 | 5 | 0 |
| TIMM23 | inf | 0.017330658 | 5 | 0 |
| MPP2 | inf | 0.017330658 | 5 | 0 |
| C4A | inf | 0.017330658 | 5 | 0 |
| TAF1C | inf | 0.017330658 | 5 | 0 |
| LRFN4 | inf | 0.017330658 | 5 | 0 |
| KIAA0100 | inf | 0.017330658 | 5 | 0 |
| PRAMEF25 | inf | 0.017330658 | 5 | 0 |
| OR5T2 | inf | 0.017330658 | 5 | 0 |
| HIPK2 | inf | 0.017330658 | 5 | 0 |
| MYO1H | inf | 0.017330658 | 5 | 0 |
| ZNF717 | 1.95 | 0.019998472 | 42 | 35 |
| LOC100287294 | 2.016025641 | 0.020259857 | 34 | 26 |
| AGAP6 | 3.718309859 | 0.021018019 | 11 | 4 |
| PABPC3 | 3.441176471 | 0.02162793 | 78 | 85 |
| NOS1 | 1.927325581 | 0.023370129 | 39 | 32 |
| LTBP1 | 5.297297297 | 0.024329855 | 8 | 2 |
| SLC9B1 | 2.933333333 | 0.030211656 | 77 | 84 |
| ERVW-1 | 1.852941176 | 0.030627912 | 54 | 51 |
| MAP4 | 3.98630137 | 0.03123314 | 9 | 3 |
| ASXL3 | 3.98630137 | 0.03123314 | 9 | 3 |
| FAM186A | 3.98630137 | 0.03123314 | 9 | 3 |
| ZNF729 | 3.98630137 | 0.03123314 | 9 | 3 |
| DSCAML1 | 7.815789474 | 0.033241573 | 6 | 1 |
| MBD3L4 | 7.815789474 | 0.033241573 | 6 | 1 |
| TARP | 7.815789474 | 0.033241573 | 6 | 1 |
| DOCK5 | 7.815789474 | 0.033241573 | 6 | 1 |
| EPHB4 | 7.815789474 | 0.033241573 | 6 | 1 |
| EVC | 7.815789474 | 0.033241573 | 6 | 1 |
| PLEKHH3 | 7.815789474 | 0.033241573 | 6 | 1 |
| CC2D1B | 7.815789474 | 0.033241573 | 6 | 1 |
| ATP2C2 | 7.815789474 | 0.033241573 | 6 | 1 |
| HEATR5A | 7.815789474 | 0.033241573 | 6 | 1 |
| GOLGA8R | 1.811066127 | 0.033851365 | 44 | 39 |
| LOC101059918 | 7.043478261 | 0.034749456 | 81 | 92 |
| USP17L11 | 2.211640212 | 0.03638907 | 19 | 12 |
| LOC101929738 | 4.444444444 | 0.036849803 | 80 | 90 |
| FSCB | 2.046610169 | 0.03695408 | 23 | 16 |
| HMCN1 | 3.333333333 | 0.036968902 | 10 | 4 |
| SPATA24 | 2.151515152 | 0.037594285 | 71 | 75 |
| MFRP | inf | 0.03954945 | 4 | 0 |
| CPSF7 | inf | 0.03954945 | 4 | 0 |
| CD163 | inf | 0.03954945 | 4 | 0 |
| ARHGEF4 | inf | 0.03954945 | 4 | 0 |
| AKAP11 | inf | 0.03954945 | 4 | 0 |
| KDM4C | inf | 0.03954945 | 4 | 0 |
| EDEM3 | inf | 0.03954945 | 4 | 0 |
| PSMB11 | inf | 0.03954945 | 4 | 0 |
| CCS | inf | 0.03954945 | 4 | 0 |
| LCA5L | inf | 0.03954945 | 4 | 0 |
| FBXO42 | inf | 0.03954945 | 4 | 0 |
| CCDC146 | inf | 0.03954945 | 4 | 0 |
| WDR6 | inf | 0.03954945 | 4 | 0 |
| PCDHGA2 | inf | 0.03954945 | 4 | 0 |
| VSTM2L | inf | 0.03954945 | 4 | 0 |
| MIER2 | inf | 0.03954945 | 4 | 0 |
| NEK1 | inf | 0.03954945 | 4 | 0 |
| ENPP3 | inf | 0.03954945 | 4 | 0 |
| SH3RF3 | inf | 0.03954945 | 4 | 0 |
| LRRD1 | inf | 0.03954945 | 4 | 0 |
| PRAMEF3 | inf | 0.03954945 | 4 | 0 |
| SLC26A4 | inf | 0.03954945 | 4 | 0 |
| EVPLL | inf | 0.03954945 | 4 | 0 |
| POC5 | inf | 0.03954945 | 4 | 0 |
| AUP1 | inf | 0.03954945 | 4 | 0 |
| PTPRR | inf | 0.03954945 | 4 | 0 |
| MGME1 | inf | 0.03954945 | 4 | 0 |
| C21orf58 | inf | 0.03954945 | 4 | 0 |
| C1orf94 | inf | 0.03954945 | 4 | 0 |
| PCDHB2 | inf | 0.03954945 | 4 | 0 |
| LOC101928799 | inf | 0.03954945 | 4 | 0 |
| KIAA0754 | inf | 0.03954945 | 4 | 0 |
| KIAA1109 | inf | 0.03954945 | 4 | 0 |
| ASPH | inf | 0.03954945 | 4 | 0 |
| RAB27A | inf | 0.03954945 | 4 | 0 |
| OR2M2 | inf | 0.03954945 | 4 | 0 |
| HIC1 | inf | 0.03954945 | 4 | 0 |
| CCDC170 | inf | 0.03954945 | 4 | 0 |
| DNMT1 | inf | 0.03954945 | 4 | 0 |
| KIAA1522 | inf | 0.03954945 | 4 | 0 |
| TCERG1L | inf | 0.03954945 | 4 | 0 |
| DOCK11 | inf | 0.03954945 | 4 | 0 |
| C2orf72 | inf | 0.03954945 | 4 | 0 |
| FAM230A&USP41 | 2.943661972 | 0.041649102 | 11 | 5 |
| RGPD3 | 2.943661972 | 0.041649102 | 11 | 5 |
| ZNF705B | 2.685714286 | 0.045413446 | 12 | 6 |
| AGAP4 | 4.573333333 | 0.045915439 | 7 | 2 |
| CDK5RAP2 | 4.573333333 | 0.045915439 | 7 | 2 |
| LOC402269 | 4.573333333 | 0.045915439 | 7 | 2 |
| CEP290 | 4.573333333 | 0.045915439 | 7 | 2 |
| COL5A1 | 4.573333333 | 0.045915439 | 7 | 2 |
| INTS1 | 4.573333333 | 0.045915439 | 7 | 2 |
| WIZ | 4.573333333 | 0.045915439 | 7 | 2 |
| FSIP2 | 2.50310559 | 0.048395313 | 13 | 7 |
| ASB10 | 1.914285714 | 0.048748785 | 67 | 70 |
| TRIM64 | 1.857142857 | 0.051025263 | 26 | 20 |
| RYR2 | 3.495495495 | 0.055704251 | 8 | 3 |
| CEP70 | 3.495495495 | 0.055704251 | 8 | 3 |
| PXN | 3.495495495 | 0.055704251 | 8 | 3 |
| BOD1L1 | 3.495495495 | 0.055704251 | 8 | 3 |
| SPDYE6 | 1.687002653 | 0.05843719 | 53 | 52 |
| IGHV7-56 | 1.674403611 | 0.058521378 | 49 | 47 |
| LOC101929455 | 3.956043956 | 0.058972528 | 80 | 91 |
| CAMKK2 | 1.730015083 | 0.060962976 | 31 | 26 |
| TBC1D3G | 1.676470588 | 0.061597423 | 38 | 34 |
| POTED | 2.95890411 | 0.063189532 | 9 | 4 |
| IGHV2-70 | 1.732704403 | 0.065012767 | 29 | 24 |
| TTLL2 | 6.428571429 | 0.066287319 | 5 | 1 |
| FNDC3B | 6.428571429 | 0.066287319 | 5 | 1 |
| ERAP2 | 6.428571429 | 0.066287319 | 5 | 1 |
| KCNG4 | 6.428571429 | 0.066287319 | 5 | 1 |
| XRN2 | 6.428571429 | 0.066287319 | 5 | 1 |
| MIB2 | 6.428571429 | 0.066287319 | 5 | 1 |
| SLC35F2 | 6.428571429 | 0.066287319 | 5 | 1 |
| FAN1 | 6.428571429 | 0.066287319 | 5 | 1 |
| TMEM104 | 6.428571429 | 0.066287319 | 5 | 1 |
| IQGAP2 | 6.428571429 | 0.066287319 | 5 | 1 |
| HNRNPCL2 | 6.428571429 | 0.066287319 | 5 | 1 |
| TSC1 | 6.428571429 | 0.066287319 | 5 | 1 |
| RHOXF2B | 6.428571429 | 0.066287319 | 5 | 1 |
| TNN | 6.428571429 | 0.066287319 | 5 | 1 |
| UPK3BL1 | 6.428571429 | 0.066287319 | 5 | 1 |
| ZNF746 | 6.428571429 | 0.066287319 | 5 | 1 |
| CRAT | 6.428571429 | 0.066287319 | 5 | 1 |
| RIPOR3 | 6.428571429 | 0.066287319 | 5 | 1 |
| ZNF831 | 6.428571429 | 0.066287319 | 5 | 1 |
| ZNF426 | 6.428571429 | 0.066287319 | 5 | 1 |
| ZC3H3 | 6.428571429 | 0.066287319 | 5 | 1 |
| PDGFRB | 6.428571429 | 0.066287319 | 5 | 1 |
| GNB3 | 6.428571429 | 0.066287319 | 5 | 1 |
| FAAH2 | 6.428571429 | 0.066287319 | 5 | 1 |
| LRRC37A2 | 2.638888889 | 0.068870668 | 10 | 5 |
| TNC | 2.638888889 | 0.068870668 | 10 | 5 |
| TEP1 | 2.638888889 | 0.068870668 | 10 | 5 |
| MYO7A | 2.638888889 | 0.068870668 | 10 | 5 |
| NPIPB5 | 1.740495868 | 0.068876838 | 27 | 22 |
| POLR2J3 | 1.746598639 | 0.070713692 | 26 | 21 |
| LRRC37A | 1.754385965 | 0.072470783 | 25 | 20 |
| USP17L22 | 2.427230047 | 0.073134287 | 11 | 6 |
| LOC101060569 | 1.775894539 | 0.075685991 | 23 | 18 |
| ZXDA | 2.27755102 | 0.076273407 | 12 | 7 |
| IGHV1-8 | 2.166666667 | 0.078510538 | 13 | 8 |
| IGHEP1 | 1.85260771 | 0.08035351 | 19 | 14 |
| DNAH17 | 2.014925373 | 0.080923124 | 15 | 10 |
| POTEH | 1.882211538 | 0.080989819 | 18 | 13 |
| ZNF492 | 1.917948718 | 0.081335115 | 17 | 12 |
| UBXN11 | 1.669230769 | 0.08201628 | 62 | 65 |
| GOLGA6L3 | 1.669230769 | 0.08201628 | 62 | 65 |
| ABCA8 | 3.868421053 | 0.084132022 | 6 | 2 |
| PI4KA | 3.868421053 | 0.084132022 | 6 | 2 |
| TIAM2 | 3.868421053 | 0.084132022 | 6 | 2 |
| SMPD3 | 3.868421053 | 0.084132022 | 6 | 2 |
| DICER1 | 3.868421053 | 0.084132022 | 6 | 2 |
| NMRAL1 | 3.868421053 | 0.084132022 | 6 | 2 |
| LOC388849 | 3.868421053 | 0.084132022 | 6 | 2 |
| AKAP12 | 3.868421053 | 0.084132022 | 6 | 2 |
| PSG8 | 3.868421053 | 0.084132022 | 6 | 2 |
| ELMO3 | 3.868421053 | 0.084132022 | 6 | 2 |
| IGHV3-30 | 3.868421053 | 0.084132022 | 6 | 2 |
| PTPRT | 3.868421053 | 0.084132022 | 6 | 2 |
| MICAL3 | 3.868421053 | 0.084132022 | 6 | 2 |
| NLRX1 | 3.868421053 | 0.084132022 | 6 | 2 |
| IGSF9 | 3.868421053 | 0.084132022 | 6 | 2 |
| CLEC18A | 3.868421053 | 0.084132022 | 6 | 2 |
| PAX3 | inf | 0.089612045 | 3 | 0 |
| PIGK | inf | 0.089612045 | 3 | 0 |
| SERPINB12 | inf | 0.089612045 | 3 | 0 |
| ZMYM1 | inf | 0.089612045 | 3 | 0 |
| APLP2 | inf | 0.089612045 | 3 | 0 |
| GLUD2 | inf | 0.089612045 | 3 | 0 |
| WDR18 | inf | 0.089612045 | 3 | 0 |
| RBM3 | inf | 0.089612045 | 3 | 0 |
| ZNF557 | inf | 0.089612045 | 3 | 0 |
| ICAM3 | inf | 0.089612045 | 3 | 0 |
| PIGZ | inf | 0.089612045 | 3 | 0 |
| PRPF38B | inf | 0.089612045 | 3 | 0 |
| KRT73 | inf | 0.089612045 | 3 | 0 |
| ZMYM5 | inf | 0.089612045 | 3 | 0 |
| EXOC6 | inf | 0.089612045 | 3 | 0 |
| NFXL1 | inf | 0.089612045 | 3 | 0 |
| SLCO4C1 | inf | 0.089612045 | 3 | 0 |
| DNPEP | inf | 0.089612045 | 3 | 0 |
| IRS1 | inf | 0.089612045 | 3 | 0 |
| XDH | inf | 0.089612045 | 3 | 0 |
| SLCO4A1 | inf | 0.089612045 | 3 | 0 |
| ADGRG7 | inf | 0.089612045 | 3 | 0 |
| AP1M2 | inf | 0.089612045 | 3 | 0 |
| CHAT | inf | 0.089612045 | 3 | 0 |
| SPDEF | inf | 0.089612045 | 3 | 0 |
| SPATA17 | inf | 0.089612045 | 3 | 0 |
| ZNF41 | inf | 0.089612045 | 3 | 0 |
| PLBD1 | inf | 0.089612045 | 3 | 0 |
| MARVELD2 | inf | 0.089612045 | 3 | 0 |
| BEGAIN | inf | 0.089612045 | 3 | 0 |
| PAF1 | inf | 0.089612045 | 3 | 0 |
| SKOR2 | inf | 0.089612045 | 3 | 0 |
| NDUFAF6 | inf | 0.089612045 | 3 | 0 |
| COL4A1 | inf | 0.089612045 | 3 | 0 |
| GCNT3 | inf | 0.089612045 | 3 | 0 |
| MUT | inf | 0.089612045 | 3 | 0 |
| CCDC12 | inf | 0.089612045 | 3 | 0 |
| IL15RA | inf | 0.089612045 | 3 | 0 |
| MATN3 | inf | 0.089612045 | 3 | 0 |
| GPANK1 | inf | 0.089612045 | 3 | 0 |
| NDUFB6 | inf | 0.089612045 | 3 | 0 |
| TAF5 | inf | 0.089612045 | 3 | 0 |
| AQR | inf | 0.089612045 | 3 | 0 |
| IGLON5 | inf | 0.089612045 | 3 | 0 |
| SRC | inf | 0.089612045 | 3 | 0 |
| ZNF189 | inf | 0.089612045 | 3 | 0 |
| IGLV2-11 | inf | 0.089612045 | 3 | 0 |
| ANKAR | inf | 0.089612045 | 3 | 0 |
| GDAP1L1 | inf | 0.089612045 | 3 | 0 |
| BAG6 | inf | 0.089612045 | 3 | 0 |
| ANKFY1 | inf | 0.089612045 | 3 | 0 |
| COBL | inf | 0.089612045 | 3 | 0 |
| IGSF3 | inf | 0.089612045 | 3 | 0 |
| AQP12B | inf | 0.089612045 | 3 | 0 |
| TTC16 | inf | 0.089612045 | 3 | 0 |
| GDF6 | inf | 0.089612045 | 3 | 0 |
| OTUD6A | inf | 0.089612045 | 3 | 0 |
| CMC1 | inf | 0.089612045 | 3 | 0 |
| FCN1 | inf | 0.089612045 | 3 | 0 |
| ZNF530 | inf | 0.089612045 | 3 | 0 |
| WDR76 | inf | 0.089612045 | 3 | 0 |
| DVL3 | inf | 0.089612045 | 3 | 0 |
| WRN | inf | 0.089612045 | 3 | 0 |
| TAF6L | inf | 0.089612045 | 3 | 0 |
| LARP1B | inf | 0.089612045 | 3 | 0 |
| SZRD1 | inf | 0.089612045 | 3 | 0 |
| RBP3 | inf | 0.089612045 | 3 | 0 |
| GFOD1 | inf | 0.089612045 | 3 | 0 |
| MYBBP1A | inf | 0.089612045 | 3 | 0 |
| DUSP8 | inf | 0.089612045 | 3 | 0 |
| BOD1 | inf | 0.089612045 | 3 | 0 |
| FCHSD1 | inf | 0.089612045 | 3 | 0 |
| TMEM240 | inf | 0.089612045 | 3 | 0 |
| OSGIN1 | inf | 0.089612045 | 3 | 0 |
| TMEM218 | inf | 0.089612045 | 3 | 0 |
| MCM8 | inf | 0.089612045 | 3 | 0 |
| OSR2 | inf | 0.089612045 | 3 | 0 |
| PAM | inf | 0.089612045 | 3 | 0 |
| PALM | inf | 0.089612045 | 3 | 0 |
| XRRA1 | inf | 0.089612045 | 3 | 0 |
| ILF2 | inf | 0.089612045 | 3 | 0 |
| MCM6 | inf | 0.089612045 | 3 | 0 |
| ANKRD62 | inf | 0.089612045 | 3 | 0 |
| MCM4 | inf | 0.089612045 | 3 | 0 |
| FBXO34 | inf | 0.089612045 | 3 | 0 |
| BANK1 | inf | 0.089612045 | 3 | 0 |
| COG4 | inf | 0.089612045 | 3 | 0 |
| ZC3H6 | inf | 0.089612045 | 3 | 0 |
| ZNF23 | inf | 0.089612045 | 3 | 0 |
| NPIPA2 | inf | 0.089612045 | 3 | 0 |
| SSBP4 | inf | 0.089612045 | 3 | 0 |
| CWC27 | inf | 0.089612045 | 3 | 0 |
| GRM7 | inf | 0.089612045 | 3 | 0 |
| LSG1 | inf | 0.089612045 | 3 | 0 |
| SAV1 | inf | 0.089612045 | 3 | 0 |
| TGM2 | inf | 0.089612045 | 3 | 0 |
| LOC101929327 | inf | 0.089612045 | 3 | 0 |
| LRIF1 | inf | 0.089612045 | 3 | 0 |
| TRIM46 | inf | 0.089612045 | 3 | 0 |
| RASGEF1A | inf | 0.089612045 | 3 | 0 |
| CTSS | inf | 0.089612045 | 3 | 0 |
| NUGGC | inf | 0.089612045 | 3 | 0 |
| RSBN1 | inf | 0.089612045 | 3 | 0 |
| CD200R1 | inf | 0.089612045 | 3 | 0 |
| ARMC6 | inf | 0.089612045 | 3 | 0 |
| HR | inf | 0.089612045 | 3 | 0 |
| GAMT | inf | 0.089612045 | 3 | 0 |
| MRRF | inf | 0.089612045 | 3 | 0 |
| KIF18B | inf | 0.089612045 | 3 | 0 |
| ATP11B | inf | 0.089612045 | 3 | 0 |
| CD1B | inf | 0.089612045 | 3 | 0 |
| SMTNL2 | inf | 0.089612045 | 3 | 0 |
| CD19 | inf | 0.089612045 | 3 | 0 |
| CD180 | inf | 0.089612045 | 3 | 0 |
| SCFD2 | inf | 0.089612045 | 3 | 0 |
| GRK3 | inf | 0.089612045 | 3 | 0 |
| GABBR2 | inf | 0.089612045 | 3 | 0 |
| CDH2 | inf | 0.089612045 | 3 | 0 |
| ADAMTS3 | inf | 0.089612045 | 3 | 0 |
| ERAP1 | inf | 0.089612045 | 3 | 0 |
| LRRC3C | inf | 0.089612045 | 3 | 0 |
| SMIM29 | inf | 0.089612045 | 3 | 0 |
| HIPK3 | inf | 0.089612045 | 3 | 0 |
| ASB15 | inf | 0.089612045 | 3 | 0 |
| ATP6AP1L | inf | 0.089612045 | 3 | 0 |
| NRDC | inf | 0.089612045 | 3 | 0 |
| SAG | inf | 0.089612045 | 3 | 0 |
| ATP6V0A4 | inf | 0.089612045 | 3 | 0 |
| MKL1 | inf | 0.089612045 | 3 | 0 |
| FAM166B | inf | 0.089612045 | 3 | 0 |
| ELP4 | inf | 0.089612045 | 3 | 0 |
| KDM2A | inf | 0.089612045 | 3 | 0 |
| DGKG | inf | 0.089612045 | 3 | 0 |
| DSCAM | inf | 0.089612045 | 3 | 0 |
| NKX2-3 | inf | 0.089612045 | 3 | 0 |
| LINC01587 | inf | 0.089612045 | 3 | 0 |
| MT3 | inf | 0.089612045 | 3 | 0 |
| EMB | inf | 0.089612045 | 3 | 0 |
| TPSD1 | inf | 0.089612045 | 3 | 0 |
| TBC1D21 | inf | 0.089612045 | 3 | 0 |
| ADAM18 | inf | 0.089612045 | 3 | 0 |
| ACOT8 | inf | 0.089612045 | 3 | 0 |
| TEKT4P2 | inf | 0.089612045 | 3 | 0 |
| TEX45 | inf | 0.089612045 | 3 | 0 |
| CELA2B | inf | 0.089612045 | 3 | 0 |
| CFAP77 | inf | 0.089612045 | 3 | 0 |
| KISS1R | inf | 0.089612045 | 3 | 0 |
| ZNF76 | inf | 0.089612045 | 3 | 0 |
| HSD17B4 | inf | 0.089612045 | 3 | 0 |
| SMARCA5 | inf | 0.089612045 | 3 | 0 |
| ABCB4 | inf | 0.089612045 | 3 | 0 |
| DDHD1 | inf | 0.089612045 | 3 | 0 |
| AGAP11 | inf | 0.089612045 | 3 | 0 |
| LGI2 | inf | 0.089612045 | 3 | 0 |
| PLEKHO1 | inf | 0.089612045 | 3 | 0 |
| PCNX3 | inf | 0.089612045 | 3 | 0 |
| SCN1A | inf | 0.089612045 | 3 | 0 |
| CCDC186 | inf | 0.089612045 | 3 | 0 |
| HTR1D | inf | 0.089612045 | 3 | 0 |
| MICA | inf | 0.089612045 | 3 | 0 |
| FEM1C | inf | 0.089612045 | 3 | 0 |
| SLC24A4 | inf | 0.089612045 | 3 | 0 |
| GIN1 | inf | 0.089612045 | 3 | 0 |
| CEP57 | inf | 0.089612045 | 3 | 0 |
| AGMAT | inf | 0.089612045 | 3 | 0 |
| ATG14 | inf | 0.089612045 | 3 | 0 |
| SLC25A23 | inf | 0.089612045 | 3 | 0 |
| PPFIA1 | inf | 0.089612045 | 3 | 0 |
| FUT6 | inf | 0.089612045 | 3 | 0 |
| ARHGEF39 | inf | 0.089612045 | 3 | 0 |
| KBTBD7 | inf | 0.089612045 | 3 | 0 |
| id158562 | inf | 0.089612045 | 3 | 0 |
| TOB2 | inf | 0.089612045 | 3 | 0 |
| GTPBP4 | inf | 0.089612045 | 3 | 0 |
| USF3 | inf | 0.089612045 | 3 | 0 |
| id572190 | inf | 0.089612045 | 3 | 0 |
| DECR2 | inf | 0.089612045 | 3 | 0 |
| GZMH | inf | 0.089612045 | 3 | 0 |
| GPR37L1 | inf | 0.089612045 | 3 | 0 |
| MRC1 | inf | 0.089612045 | 3 | 0 |
| TBC1D7 | inf | 0.089612045 | 3 | 0 |
| ABCC8 | inf | 0.089612045 | 3 | 0 |
| MOCOS | inf | 0.089612045 | 3 | 0 |
| DCST2 | inf | 0.089612045 | 3 | 0 |
| SCLY | inf | 0.089612045 | 3 | 0 |
| EFCAB12 | inf | 0.089612045 | 3 | 0 |
| GIT2 | inf | 0.089612045 | 3 | 0 |
| FANCC | inf | 0.089612045 | 3 | 0 |
| LOC440243 | 1.576612903 | 0.09512535 | 34 | 31 |
| TSNARE1 | 3.017777778 | 0.096281802 | 7 | 3 |
| TYRO3 | 3.017777778 | 0.096281802 | 7 | 3 |
| SORBS2 | 3.017777778 | 0.096281802 | 7 | 3 |
| UTP20 | 3.017777778 | 0.096281802 | 7 | 3 |
| ZNF705D | 3.017777778 | 0.096281802 | 7 | 3 |
| CRYBG3 | 3.017777778 | 0.096281802 | 7 | 3 |
| TAS2R30 | 5.170212766 | 0.097414335 | 81 | 94 |
| UCHL3 | 5.170212766 | 0.097414335 | 81 | 94 |
| TRIM64B | 1.66190901 | 0.103149133 | 23 | 19 |
| ARHGEF28 | 2.594594595 | 0.104646473 | 8 | 4 |
| PCDHB14 | 2.594594595 | 0.104646473 | 8 | 4 |
| ABCA7 | 2.594594595 | 0.104646473 | 8 | 4 |
| HRCT1 | 2.594594595 | 0.104646473 | 8 | 4 |
| ANKRD20A3 | 1.693548387 | 0.110131639 | 20 | 16 |
| FASN | 2.342465753 | 0.110386665 | 9 | 5 |
| WNK2 | 2.342465753 | 0.110386665 | 9 | 5 |
| DNAH12 | 2.342465753 | 0.110386665 | 9 | 5 |
| DUX4L8 | 1.652054795 | 0.112658701 | 67 | 73 |
| PRKRA | 1.727678571 | 0.114028974 | 18 | 14 |
| ZNF676 | 1.727678571 | 0.114028974 | 18 | 14 |
| SPTBN5 | 2.175925926 | 0.114239946 | 10 | 6 |
| KMT2D | 2.175925926 | 0.114239946 | 10 | 6 |
| DNAH7 | 2.058350101 | 0.116695609 | 11 | 7 |
| SDK1 | 2.058350101 | 0.116695609 | 11 | 7 |
| POMZP3 | 1.971428571 | 0.118090547 | 12 | 8 |
| EPPK1 | 1.904991948 | 0.11866417 | 13 | 9 |
| CRYBG2 | 1.486486486 | 0.119527357 | 45 | 45 |
| C5orf34 | 5.076923077 | 0.128424619 | 4 | 1 |
| ZDBF2 | 5.076923077 | 0.128424619 | 4 | 1 |
| PABPN1L | 5.076923077 | 0.128424619 | 4 | 1 |
| FAAP100 | 5.076923077 | 0.128424619 | 4 | 1 |
| SLC35F4 | 5.076923077 | 0.128424619 | 4 | 1 |
| BCL6 | 5.076923077 | 0.128424619 | 4 | 1 |
| HIPK4 | 5.076923077 | 0.128424619 | 4 | 1 |
| PTPN14 | 5.076923077 | 0.128424619 | 4 | 1 |
| SREBF1 | 5.076923077 | 0.128424619 | 4 | 1 |
| MMP2 | 5.076923077 | 0.128424619 | 4 | 1 |
| KIAA1551 | 5.076923077 | 0.128424619 | 4 | 1 |
| ABCA1 | 5.076923077 | 0.128424619 | 4 | 1 |
| SHROOM2 | 5.076923077 | 0.128424619 | 4 | 1 |
| PPIAL4A | 5.076923077 | 0.128424619 | 4 | 1 |
| INSC | 5.076923077 | 0.128424619 | 4 | 1 |
| FOXD4 | 5.076923077 | 0.128424619 | 4 | 1 |
| SFMBT2 | 5.076923077 | 0.128424619 | 4 | 1 |
| WASHC4 | 5.076923077 | 0.128424619 | 4 | 1 |
| KRT83 | 5.076923077 | 0.128424619 | 4 | 1 |
| PARP10 | 5.076923077 | 0.128424619 | 4 | 1 |
| RIBC2 | 5.076923077 | 0.128424619 | 4 | 1 |
| TSHZ2 | 5.076923077 | 0.128424619 | 4 | 1 |
| SDCCAG8 | 5.076923077 | 0.128424619 | 4 | 1 |
| CNGA3 | 5.076923077 | 0.128424619 | 4 | 1 |
| SLC22A8 | 5.076923077 | 0.128424619 | 4 | 1 |
| KIAA2026 | 5.076923077 | 0.128424619 | 4 | 1 |
| ITPR3 | 5.076923077 | 0.128424619 | 4 | 1 |
| C19orf44 | 5.076923077 | 0.128424619 | 4 | 1 |
| ACLY | 5.076923077 | 0.128424619 | 4 | 1 |
| NUP205 | 5.076923077 | 0.128424619 | 4 | 1 |
| MCC | 5.076923077 | 0.128424619 | 4 | 1 |
| MPND | 5.076923077 | 0.128424619 | 4 | 1 |
| POTEA | 5.076923077 | 0.128424619 | 4 | 1 |
| FLCN | 5.076923077 | 0.128424619 | 4 | 1 |
| PGC | 5.076923077 | 0.128424619 | 4 | 1 |
| KIF14 | 5.076923077 | 0.128424619 | 4 | 1 |
| STRC | 5.076923077 | 0.128424619 | 4 | 1 |
| DRD5 | 5.076923077 | 0.128424619 | 4 | 1 |
| MASP1 | 5.076923077 | 0.128424619 | 4 | 1 |
| SPEN | 5.076923077 | 0.128424619 | 4 | 1 |
| YEATS2 | 5.076923077 | 0.128424619 | 4 | 1 |
| SIKE1 | 5.076923077 | 0.128424619 | 4 | 1 |
| EPHA2 | 5.076923077 | 0.128424619 | 4 | 1 |
| MTHFR | 5.076923077 | 0.128424619 | 4 | 1 |
| TTLL8 | 5.076923077 | 0.128424619 | 4 | 1 |
| DIS3L | 5.076923077 | 0.128424619 | 4 | 1 |
| CEP68 | 5.076923077 | 0.128424619 | 4 | 1 |
| TOR1B | 5.076923077 | 0.128424619 | 4 | 1 |
| AREL1 | 5.076923077 | 0.128424619 | 4 | 1 |
| KANSL1L | 5.076923077 | 0.128424619 | 4 | 1 |
| CTSE | 5.076923077 | 0.128424619 | 4 | 1 |
| EPHB2 | 5.076923077 | 0.128424619 | 4 | 1 |
| PHF12 | 5.076923077 | 0.128424619 | 4 | 1 |
| IFT122 | 5.076923077 | 0.128424619 | 4 | 1 |
| ARHGAP20 | 5.076923077 | 0.128424619 | 4 | 1 |
| PMPCA | 5.076923077 | 0.128424619 | 4 | 1 |
| COPA | 5.076923077 | 0.128424619 | 4 | 1 |
| EML4 | 5.076923077 | 0.128424619 | 4 | 1 |
| CCDC7 | 5.076923077 | 0.128424619 | 4 | 1 |
| OSBP2 | 5.076923077 | 0.128424619 | 4 | 1 |
| NFATC1 | 5.076923077 | 0.128424619 | 4 | 1 |
| CBWD1 | 5.076923077 | 0.128424619 | 4 | 1 |
| KLHL35 | 5.076923077 | 0.128424619 | 4 | 1 |
| FAM171A1 | 5.076923077 | 0.128424619 | 4 | 1 |
| ADAMTS13 | 5.076923077 | 0.128424619 | 4 | 1 |
| WDR3 | 5.076923077 | 0.128424619 | 4 | 1 |
| KDM5A | 5.076923077 | 0.128424619 | 4 | 1 |
| MAN2A2 | 5.076923077 | 0.128424619 | 4 | 1 |
| ANKHD1 | 5.076923077 | 0.128424619 | 4 | 1 |
| CDT1 | 5.076923077 | 0.128424619 | 4 | 1 |
| COG5 | 5.076923077 | 0.128424619 | 4 | 1 |
| KDM2B | 5.076923077 | 0.128424619 | 4 | 1 |
| LOC100505767 | 5.076923077 | 0.128424619 | 4 | 1 |
| MAMDC4 | 5.076923077 | 0.128424619 | 4 | 1 |
| ARHGEF11 | 5.076923077 | 0.128424619 | 4 | 1 |
| PKLR | 5.076923077 | 0.128424619 | 4 | 1 |
| WIPI2 | 5.076923077 | 0.128424619 | 4 | 1 |
| PRAMEF13 | 5.076923077 | 0.128424619 | 4 | 1 |
| KLHL10 | 5.076923077 | 0.128424619 | 4 | 1 |
| FAM161B | 5.076923077 | 0.128424619 | 4 | 1 |
| CEP89 | 5.076923077 | 0.128424619 | 4 | 1 |
| COL22A1 | 5.076923077 | 0.128424619 | 4 | 1 |
| ADAM19 | 5.076923077 | 0.128424619 | 4 | 1 |
| ZNF618 | 5.076923077 | 0.128424619 | 4 | 1 |
| ADAM22 | 5.076923077 | 0.128424619 | 4 | 1 |
| ARHGEF1 | 5.076923077 | 0.128424619 | 4 | 1 |
| KCNQ5 | 5.076923077 | 0.128424619 | 4 | 1 |
| GATAD2A | 5.076923077 | 0.128424619 | 4 | 1 |
| MRPL4 | 5.076923077 | 0.128424619 | 4 | 1 |
| WFIKKN1 | 5.076923077 | 0.128424619 | 4 | 1 |
| PRPF18 | 5.076923077 | 0.128424619 | 4 | 1 |
| THOP1 | 5.076923077 | 0.128424619 | 4 | 1 |
| LOC100996713 | 1.559322034 | 0.13651685 | 23 | 20 |
| ZNF141 | 1.483516484 | 0.140810699 | 30 | 28 |
| RIMS2 | 3.181818182 | 0.148901684 | 5 | 2 |
| SPEF2 | 3.181818182 | 0.148901684 | 5 | 2 |
| ZNRF3 | 3.181818182 | 0.148901684 | 5 | 2 |
| SLC12A4 | 3.181818182 | 0.148901684 | 5 | 2 |
| MARS | 3.181818182 | 0.148901684 | 5 | 2 |
| MAGEA9B | 3.181818182 | 0.148901684 | 5 | 2 |
| ANKRD30B | 3.181818182 | 0.148901684 | 5 | 2 |
| USP36 | 3.181818182 | 0.148901684 | 5 | 2 |
| PHF3 | 3.181818182 | 0.148901684 | 5 | 2 |
| ATP7B | 3.181818182 | 0.148901684 | 5 | 2 |
| NBEAL1 | 3.181818182 | 0.148901684 | 5 | 2 |
| DOCK10 | 3.181818182 | 0.148901684 | 5 | 2 |
| ZNF582 | 3.181818182 | 0.148901684 | 5 | 2 |
| ANGPTL1 | 3.181818182 | 0.148901684 | 5 | 2 |
| FAT4 | 3.181818182 | 0.148901684 | 5 | 2 |
| SLIT3 | 3.181818182 | 0.148901684 | 5 | 2 |
| ZNF598 | 3.181818182 | 0.148901684 | 5 | 2 |
| LOC100996634 | 3.181818182 | 0.148901684 | 5 | 2 |
| PIK3C2A | 3.181818182 | 0.148901684 | 5 | 2 |
| XKR6 | 3.181818182 | 0.148901684 | 5 | 2 |
| ARHGEF17 | 3.181818182 | 0.148901684 | 5 | 2 |
| PLCH2 | 3.181818182 | 0.148901684 | 5 | 2 |
| SUCO | 3.181818182 | 0.148901684 | 5 | 2 |
| PLXNB3 | 3.181818182 | 0.148901684 | 5 | 2 |
| LAMC3 | 3.181818182 | 0.148901684 | 5 | 2 |
| UVSSA | 3.181818182 | 0.148901684 | 5 | 2 |
| TCP10 | 3.181818182 | 0.148901684 | 5 | 2 |
| ITPR2 | 3.181818182 | 0.148901684 | 5 | 2 |
| RBMS1 | 3.181818182 | 0.148901684 | 5 | 2 |
| PRRC2C | 3.181818182 | 0.148901684 | 5 | 2 |
| ABCC11 | 3.181818182 | 0.148901684 | 5 | 2 |
| ADCY5 | 3.181818182 | 0.148901684 | 5 | 2 |
| SPATA31A7 | 3.181818182 | 0.148901684 | 5 | 2 |
| SEM1 | 3.181818182 | 0.148901684 | 5 | 2 |
| VWA5A | 3.181818182 | 0.148901684 | 5 | 2 |
| DENND2A | 3.181818182 | 0.148901684 | 5 | 2 |
| SRGAP2B | 3.181818182 | 0.148901684 | 5 | 2 |
| NEK9 | 3.181818182 | 0.148901684 | 5 | 2 |
| PRDM2 | 3.181818182 | 0.148901684 | 5 | 2 |
| GTF2IRD2 | 2.552631579 | 0.160565187 | 6 | 3 |
| PATJ | 2.552631579 | 0.160565187 | 6 | 3 |
| FMN2 | 2.552631579 | 0.160565187 | 6 | 3 |
| CTAGE6 | 2.552631579 | 0.160565187 | 6 | 3 |
| OPLAH | 2.552631579 | 0.160565187 | 6 | 3 |
| TRIM43 | 2.552631579 | 0.160565187 | 6 | 3 |
| CEP250 | 2.552631579 | 0.160565187 | 6 | 3 |
| PLB1 | 2.552631579 | 0.160565187 | 6 | 3 |
| ADAM8 | 2.552631579 | 0.160565187 | 6 | 3 |
| MYH1 | 2.552631579 | 0.160565187 | 6 | 3 |
| FBN3 | 2.552631579 | 0.160565187 | 6 | 3 |
| UMODL1 | 1.409090909 | 0.162487048 | 38 | 38 |
| TRBV5-6 | inf | 0.163620909 | 82 | 97 |
| ZNF257 | 1.665775401 | 0.1665325 | 14 | 11 |
| KRTAP10-3 | 2.24 | 0.167292517 | 7 | 4 |
| MXRA5 | 2.24 | 0.167292517 | 7 | 4 |
| CFAP43 | 2.24 | 0.167292517 | 7 | 4 |
| CCDC129 | 1.733333333 | 0.170863892 | 12 | 9 |
| OR11H12 | 2.054054054 | 0.170977579 | 8 | 5 |
| S100G | 2.054054054 | 0.170977579 | 8 | 5 |
| MYOM2 | 2.054054054 | 0.170977579 | 8 | 5 |
| NPIPA8 | 2.054054054 | 0.170977579 | 8 | 5 |
| NXF2 | 2.054054054 | 0.170977579 | 8 | 5 |
| LAMA2 | 1.781690141 | 0.172276136 | 11 | 8 |
| HBZ | 1.931506849 | 0.17266066 | 9 | 6 |
| SNX25 | 1.931506849 | 0.17266066 | 9 | 6 |
| KCNJ12 | 1.931506849 | 0.17266066 | 9 | 6 |
| LRP1B | 1.931506849 | 0.17266066 | 9 | 6 |
| ZAN | 1.845238095 | 0.172962316 | 10 | 7 |
| TRBV7-3 | 1.396551724 | 0.176134868 | 54 | 58 |
| SPDYE2B | 1.46953405 | 0.191442408 | 20 | 18 |
| KRT18 | 1.449324324 | 0.196110133 | 66 | 74 |
| TRBV10-2 | 1.350815851 | 0.198569269 | 38 | 39 |
| LRRC37A3 | 1.381270903 | 0.200262981 | 59 | 65 |
| MED29 | inf | 0.201627102 | 2 | 0 |
| QPCT | inf | 0.201627102 | 2 | 0 |
| C7orf65 | inf | 0.201627102 | 2 | 0 |
| LOC100289187 | inf | 0.201627102 | 2 | 0 |
| CD300LD | inf | 0.201627102 | 2 | 0 |
| STC1 | inf | 0.201627102 | 2 | 0 |
| STIM2 | inf | 0.201627102 | 2 | 0 |
| EFS | inf | 0.201627102 | 2 | 0 |
| UGT3A1 | inf | 0.201627102 | 2 | 0 |
| PHAX | inf | 0.201627102 | 2 | 0 |
| EPS15L1 | inf | 0.201627102 | 2 | 0 |
| EGR2 | inf | 0.201627102 | 2 | 0 |
| CD4 | inf | 0.201627102 | 2 | 0 |
| LRRC69 | inf | 0.201627102 | 2 | 0 |
| LRRN2 | inf | 0.201627102 | 2 | 0 |
| FMO4 | inf | 0.201627102 | 2 | 0 |
| C16orf46 | inf | 0.201627102 | 2 | 0 |
| LOC100652824 | inf | 0.201627102 | 2 | 0 |
| GALM | inf | 0.201627102 | 2 | 0 |
| PGM2 | inf | 0.201627102 | 2 | 0 |
| ZBTB21 | inf | 0.201627102 | 2 | 0 |
| PPP6R1 | inf | 0.201627102 | 2 | 0 |
| GHRHR | inf | 0.201627102 | 2 | 0 |
| PPP4R1 | inf | 0.201627102 | 2 | 0 |
| VILL | inf | 0.201627102 | 2 | 0 |
| PPP2R5D | inf | 0.201627102 | 2 | 0 |
| CD36 | inf | 0.201627102 | 2 | 0 |
| TRMO | inf | 0.201627102 | 2 | 0 |
| GALNT15 | inf | 0.201627102 | 2 | 0 |
| CCDC198 | inf | 0.201627102 | 2 | 0 |
| SRRM4 | inf | 0.201627102 | 2 | 0 |
| EMP1 | inf | 0.201627102 | 2 | 0 |
| PYGL | inf | 0.201627102 | 2 | 0 |
| EFCAB14 | inf | 0.201627102 | 2 | 0 |
| UBAP2L | inf | 0.201627102 | 2 | 0 |
| UBASH3B | inf | 0.201627102 | 2 | 0 |
| PRDM4 | inf | 0.201627102 | 2 | 0 |
| LYPD2 | inf | 0.201627102 | 2 | 0 |
| FN3KRP | inf | 0.201627102 | 2 | 0 |
| STK33 | inf | 0.201627102 | 2 | 0 |
| LINGO4 | inf | 0.201627102 | 2 | 0 |
| MID2 | inf | 0.201627102 | 2 | 0 |
| PRCC | inf | 0.201627102 | 2 | 0 |
| CD1E | inf | 0.201627102 | 2 | 0 |
| LIPK | inf | 0.201627102 | 2 | 0 |
| JADE3 | inf | 0.201627102 | 2 | 0 |
| LUZP2 | inf | 0.201627102 | 2 | 0 |
| LUC7L | inf | 0.201627102 | 2 | 0 |
| TRIM5 | inf | 0.201627102 | 2 | 0 |
| LMBRD1 | inf | 0.201627102 | 2 | 0 |
| GGT6 | inf | 0.201627102 | 2 | 0 |
| MIOX | inf | 0.201627102 | 2 | 0 |
| EFNA1 | inf | 0.201627102 | 2 | 0 |
| PRAMEF22 | inf | 0.201627102 | 2 | 0 |
| EPPIN-WFDC6 | inf | 0.201627102 | 2 | 0 |
| PNPLA5 | inf | 0.201627102 | 2 | 0 |
| PHC3 | inf | 0.201627102 | 2 | 0 |
| ELOVL2 | inf | 0.201627102 | 2 | 0 |
| EMC2 | inf | 0.201627102 | 2 | 0 |
| RNF212B | inf | 0.201627102 | 2 | 0 |
| KIF3B | inf | 0.201627102 | 2 | 0 |
| PPP2R1B | inf | 0.201627102 | 2 | 0 |
| LRRC40 | inf | 0.201627102 | 2 | 0 |
| PFKFB1 | inf | 0.201627102 | 2 | 0 |
| UTP3 | inf | 0.201627102 | 2 | 0 |
| ZBTB7A | inf | 0.201627102 | 2 | 0 |
| CDC14A | inf | 0.201627102 | 2 | 0 |
| USP17L12 | inf | 0.201627102 | 2 | 0 |
| PPIG | inf | 0.201627102 | 2 | 0 |
| PFDN4 | inf | 0.201627102 | 2 | 0 |
| LPXN | inf | 0.201627102 | 2 | 0 |
| USP53 | inf | 0.201627102 | 2 | 0 |
| ZC3H12B | inf | 0.201627102 | 2 | 0 |
| PPIC | inf | 0.201627102 | 2 | 0 |
| C1orf146 | inf | 0.201627102 | 2 | 0 |
| SSX4B | inf | 0.201627102 | 2 | 0 |
| SSX4 | inf | 0.201627102 | 2 | 0 |
| SHROOM1 | inf | 0.201627102 | 2 | 0 |
| KIF12 | inf | 0.201627102 | 2 | 0 |
| C2orf42 | inf | 0.201627102 | 2 | 0 |
| C1orf174 | inf | 0.201627102 | 2 | 0 |
| KIF11 | inf | 0.201627102 | 2 | 0 |
| LOXL1 | inf | 0.201627102 | 2 | 0 |
| ST3GAL3 | inf | 0.201627102 | 2 | 0 |
| FNDC10 | inf | 0.201627102 | 2 | 0 |
| TEX46 | inf | 0.201627102 | 2 | 0 |
| USP38 | inf | 0.201627102 | 2 | 0 |
| USP17L7 | inf | 0.201627102 | 2 | 0 |
| USP20 | inf | 0.201627102 | 2 | 0 |
| MIR1-1HG | inf | 0.201627102 | 2 | 0 |
| ATG7 | inf | 0.201627102 | 2 | 0 |
| LPCAT2 | inf | 0.201627102 | 2 | 0 |
| UTP6 | inf | 0.201627102 | 2 | 0 |
| SLC25A30 | inf | 0.201627102 | 2 | 0 |
| CD99 | inf | 0.201627102 | 2 | 0 |
| VEZT | inf | 0.201627102 | 2 | 0 |
| LILRA1 | inf | 0.201627102 | 2 | 0 |
| LOC101927016 | inf | 0.201627102 | 2 | 0 |
| GID4 | inf | 0.201627102 | 2 | 0 |
| C18orf25 | inf | 0.201627102 | 2 | 0 |
| C4orf26 | inf | 0.201627102 | 2 | 0 |
| PPP1R12A | inf | 0.201627102 | 2 | 0 |
| LRRC25 | inf | 0.201627102 | 2 | 0 |
| SIDT2 | inf | 0.201627102 | 2 | 0 |
| PPP1CA | inf | 0.201627102 | 2 | 0 |
| CD6 | inf | 0.201627102 | 2 | 0 |
| PGLYRP2 | inf | 0.201627102 | 2 | 0 |
| EPYC | inf | 0.201627102 | 2 | 0 |
| CD68 | inf | 0.201627102 | 2 | 0 |
| CD7 | inf | 0.201627102 | 2 | 0 |
| SLC22A5 | inf | 0.201627102 | 2 | 0 |
| EIF2B3 | inf | 0.201627102 | 2 | 0 |
| C3orf35 | inf | 0.201627102 | 2 | 0 |
| FUOM | inf | 0.201627102 | 2 | 0 |
| GIMAP4 | inf | 0.201627102 | 2 | 0 |
| MLXIPL | inf | 0.201627102 | 2 | 0 |
| STAG3 | inf | 0.201627102 | 2 | 0 |
| KIF22 | inf | 0.201627102 | 2 | 0 |
| LRIT3 | inf | 0.201627102 | 2 | 0 |
| UVRAG | inf | 0.201627102 | 2 | 0 |
| PORCN | inf | 0.201627102 | 2 | 0 |
| LOC101929370 | inf | 0.201627102 | 2 | 0 |
| CD8B | inf | 0.201627102 | 2 | 0 |
| FLRT1 | inf | 0.201627102 | 2 | 0 |
| CCDC189 | inf | 0.201627102 | 2 | 0 |
| PRG4 | inf | 0.201627102 | 2 | 0 |
| EEPD1 | inf | 0.201627102 | 2 | 0 |
| LHX4 | inf | 0.201627102 | 2 | 0 |
| MCL1 | inf | 0.201627102 | 2 | 0 |
| TRMT2A | inf | 0.201627102 | 2 | 0 |
| SUCLG2 | inf | 0.201627102 | 2 | 0 |
| WNT2 | inf | 0.201627102 | 2 | 0 |
| BEND6 | inf | 0.201627102 | 2 | 0 |
| MCAM | inf | 0.201627102 | 2 | 0 |
| MBTPS1 | inf | 0.201627102 | 2 | 0 |
| BET1L | inf | 0.201627102 | 2 | 0 |
| E2F2 | inf | 0.201627102 | 2 | 0 |
| CCDC166 | inf | 0.201627102 | 2 | 0 |
| E2F3 | inf | 0.201627102 | 2 | 0 |
| PICK1 | inf | 0.201627102 | 2 | 0 |
| PSG5 | inf | 0.201627102 | 2 | 0 |
| TSSK3 | inf | 0.201627102 | 2 | 0 |
| TSPAN32 | inf | 0.201627102 | 2 | 0 |
| WISP2 | inf | 0.201627102 | 2 | 0 |
| GCNT4 | inf | 0.201627102 | 2 | 0 |
| MATK | inf | 0.201627102 | 2 | 0 |
| CCDC115 | inf | 0.201627102 | 2 | 0 |
| PLA2G2F | inf | 0.201627102 | 2 | 0 |
| CCNDBP1 | inf | 0.201627102 | 2 | 0 |
| SLC10A6 | inf | 0.201627102 | 2 | 0 |
| METTL22 | inf | 0.201627102 | 2 | 0 |
| KIAA2013 | inf | 0.201627102 | 2 | 0 |
| TTC27 | inf | 0.201627102 | 2 | 0 |
| PRRT2 | inf | 0.201627102 | 2 | 0 |
| MARCH7 | inf | 0.201627102 | 2 | 0 |
| PIAS1 | inf | 0.201627102 | 2 | 0 |
| TTC30B | inf | 0.201627102 | 2 | 0 |
| STX19 | inf | 0.201627102 | 2 | 0 |
| PSD3 | inf | 0.201627102 | 2 | 0 |
| CBY1 | inf | 0.201627102 | 2 | 0 |
| CCL4L2 | inf | 0.201627102 | 2 | 0 |
| ADGRB3 | inf | 0.201627102 | 2 | 0 |
| KRT33A | inf | 0.201627102 | 2 | 0 |
| KRT35 | inf | 0.201627102 | 2 | 0 |
| KRT20 | inf | 0.201627102 | 2 | 0 |
| KRT36 | inf | 0.201627102 | 2 | 0 |
| MEF2A | inf | 0.201627102 | 2 | 0 |
| DYNC1I1 | inf | 0.201627102 | 2 | 0 |
| TRPV4 | inf | 0.201627102 | 2 | 0 |
| SPTLC3 | inf | 0.201627102 | 2 | 0 |
| SPZ1 | inf | 0.201627102 | 2 | 0 |
| MEF2C | inf | 0.201627102 | 2 | 0 |
| TRPV5 | inf | 0.201627102 | 2 | 0 |
| CCDC9 | inf | 0.201627102 | 2 | 0 |
| WT1 | inf | 0.201627102 | 2 | 0 |
| GDPD5 | inf | 0.201627102 | 2 | 0 |
| PSMG1 | inf | 0.201627102 | 2 | 0 |
| PTGDR2 | inf | 0.201627102 | 2 | 0 |
| BCKDHB | inf | 0.201627102 | 2 | 0 |
| PIGB | inf | 0.201627102 | 2 | 0 |
| PTPN9 | inf | 0.201627102 | 2 | 0 |
| KRI1 | inf | 0.201627102 | 2 | 0 |
| KRT84 | inf | 0.201627102 | 2 | 0 |
| FOSL2 | inf | 0.201627102 | 2 | 0 |
| FNDC8 | inf | 0.201627102 | 2 | 0 |
| EPB41 | inf | 0.201627102 | 2 | 0 |
| EPHA10 | inf | 0.201627102 | 2 | 0 |
| MCTP1 | inf | 0.201627102 | 2 | 0 |
| GFAP | inf | 0.201627102 | 2 | 0 |
| DYRK1A | inf | 0.201627102 | 2 | 0 |
| METTL12 | inf | 0.201627102 | 2 | 0 |
| TSNAXIP1 | inf | 0.201627102 | 2 | 0 |
| KRT81 | inf | 0.201627102 | 2 | 0 |
| TTC33 | inf | 0.201627102 | 2 | 0 |
| DUSP5 | inf | 0.201627102 | 2 | 0 |
| SLC12A6 | inf | 0.201627102 | 2 | 0 |
| LDHAL6A | inf | 0.201627102 | 2 | 0 |
| SLC15A1 | inf | 0.201627102 | 2 | 0 |
| SRP72 | inf | 0.201627102 | 2 | 0 |
| BTBD1 | inf | 0.201627102 | 2 | 0 |
| LDHD | inf | 0.201627102 | 2 | 0 |
| DUSP18 | inf | 0.201627102 | 2 | 0 |
| CCT7 | inf | 0.201627102 | 2 | 0 |
| SLC15A2 | inf | 0.201627102 | 2 | 0 |
| MAN1C1 | inf | 0.201627102 | 2 | 0 |
| LEFTY1 | inf | 0.201627102 | 2 | 0 |
| PRMT2 | inf | 0.201627102 | 2 | 0 |
| MAGEF1 | inf | 0.201627102 | 2 | 0 |
| PXMP2 | inf | 0.201627102 | 2 | 0 |
| MAGEE1 | inf | 0.201627102 | 2 | 0 |
| CARHSP1 | inf | 0.201627102 | 2 | 0 |
| MAGED2 | inf | 0.201627102 | 2 | 0 |
| CANT1 | inf | 0.201627102 | 2 | 0 |
| GATM | inf | 0.201627102 | 2 | 0 |
| AVPR2 | inf | 0.201627102 | 2 | 0 |
| ENAM | inf | 0.201627102 | 2 | 0 |
| BUB1B | inf | 0.201627102 | 2 | 0 |
| PLEKHM2 | inf | 0.201627102 | 2 | 0 |
| TXNIP | inf | 0.201627102 | 2 | 0 |
| C10orf105 | inf | 0.201627102 | 2 | 0 |
| WARS2 | inf | 0.201627102 | 2 | 0 |
| LGALS9C | inf | 0.201627102 | 2 | 0 |
| EDRF1 | inf | 0.201627102 | 2 | 0 |
| STON1-GTF2A1L | inf | 0.201627102 | 2 | 0 |
| AVEN | inf | 0.201627102 | 2 | 0 |
| PLIN3 | inf | 0.201627102 | 2 | 0 |
| MAGED1 | inf | 0.201627102 | 2 | 0 |
| CARM1 | inf | 0.201627102 | 2 | 0 |
| MFSD8 | inf | 0.201627102 | 2 | 0 |
| SLC13A4 | inf | 0.201627102 | 2 | 0 |
| SRF | inf | 0.201627102 | 2 | 0 |
| MAP4K2 | inf | 0.201627102 | 2 | 0 |
| LAS1L | inf | 0.201627102 | 2 | 0 |
| GCH1 | inf | 0.201627102 | 2 | 0 |
| FAM212A | inf | 0.201627102 | 2 | 0 |
| SLC2A12 | inf | 0.201627102 | 2 | 0 |
| PTTG2 | inf | 0.201627102 | 2 | 0 |
| PUM2 | inf | 0.201627102 | 2 | 0 |
| MAPK3 | inf | 0.201627102 | 2 | 0 |
| SMIM29 | inf | 0.201627102 | 2 | 0 |
| KLHL7 | inf | 0.201627102 | 2 | 0 |
| PRR25 | inf | 0.201627102 | 2 | 0 |
| MFGE8 | inf | 0.201627102 | 2 | 0 |
| B3GNT7 | inf | 0.201627102 | 2 | 0 |
| WDR64 | inf | 0.201627102 | 2 | 0 |
| GCDH | inf | 0.201627102 | 2 | 0 |
| YARS2 | inf | 0.201627102 | 2 | 0 |
| PWP1 | inf | 0.201627102 | 2 | 0 |
| ECI2 | inf | 0.201627102 | 2 | 0 |
| PRKCSH | inf | 0.201627102 | 2 | 0 |
| MAP3K12 | inf | 0.201627102 | 2 | 0 |
| PRPF40B | inf | 0.201627102 | 2 | 0 |
| PRPH | inf | 0.201627102 | 2 | 0 |
| CCDC85A | inf | 0.201627102 | 2 | 0 |
| MFSD4A | inf | 0.201627102 | 2 | 0 |
| GGN | inf | 0.201627102 | 2 | 0 |
| FOXRED1 | inf | 0.201627102 | 2 | 0 |
| GCAT | inf | 0.201627102 | 2 | 0 |
| CASP6 | inf | 0.201627102 | 2 | 0 |
| TTLL3 | inf | 0.201627102 | 2 | 0 |
| STS | inf | 0.201627102 | 2 | 0 |
| PRPH2 | inf | 0.201627102 | 2 | 0 |
| DEF8 | inf | 0.201627102 | 2 | 0 |
| CFLAR | inf | 0.201627102 | 2 | 0 |
| TREM1 | inf | 0.201627102 | 2 | 0 |
| HTR3B | inf | 0.201627102 | 2 | 0 |
| ZNF620 | inf | 0.201627102 | 2 | 0 |
| ZNF615 | inf | 0.201627102 | 2 | 0 |
| OR5H14 | inf | 0.201627102 | 2 | 0 |
| NLGN4X | inf | 0.201627102 | 2 | 0 |
| CRNN | inf | 0.201627102 | 2 | 0 |
| AGRP | inf | 0.201627102 | 2 | 0 |
| HUNK | inf | 0.201627102 | 2 | 0 |
| NKX6-1 | inf | 0.201627102 | 2 | 0 |
| HUWE1 | inf | 0.201627102 | 2 | 0 |
| TK1 | inf | 0.201627102 | 2 | 0 |
| HYI | inf | 0.201627102 | 2 | 0 |
| OR5F1 | inf | 0.201627102 | 2 | 0 |
| CRIP3 | inf | 0.201627102 | 2 | 0 |
| FAM126B | inf | 0.201627102 | 2 | 0 |
| AHR | inf | 0.201627102 | 2 | 0 |
| TAOK3 | inf | 0.201627102 | 2 | 0 |
| NKX2-1 | inf | 0.201627102 | 2 | 0 |
| GPR156 | inf | 0.201627102 | 2 | 0 |
| FASTKD5 | inf | 0.201627102 | 2 | 0 |
| ZNF599 | inf | 0.201627102 | 2 | 0 |
| NKAPL | inf | 0.201627102 | 2 | 0 |
| NKAIN4 | inf | 0.201627102 | 2 | 0 |
| CREB3L4 | inf | 0.201627102 | 2 | 0 |
| ZNF595 | inf | 0.201627102 | 2 | 0 |
| DLGAP1 | inf | 0.201627102 | 2 | 0 |
| GPR157 | inf | 0.201627102 | 2 | 0 |
| CRYBA4 | inf | 0.201627102 | 2 | 0 |
| OR5C1 | inf | 0.201627102 | 2 | 0 |
| HTR1E | inf | 0.201627102 | 2 | 0 |
| CTRB1 | inf | 0.201627102 | 2 | 0 |
| HRASLS2 | inf | 0.201627102 | 2 | 0 |
| HRH4 | inf | 0.201627102 | 2 | 0 |
| GPR83 | inf | 0.201627102 | 2 | 0 |
| RPUSD2 | inf | 0.201627102 | 2 | 0 |
| HS3ST4 | inf | 0.201627102 | 2 | 0 |
| ADNP | inf | 0.201627102 | 2 | 0 |
| SMARCA4 | inf | 0.201627102 | 2 | 0 |
| ADPRH | inf | 0.201627102 | 2 | 0 |
| ADPRM | inf | 0.201627102 | 2 | 0 |
| DHX35 | inf | 0.201627102 | 2 | 0 |
| OR52E6 | inf | 0.201627102 | 2 | 0 |
| ADRB3 | inf | 0.201627102 | 2 | 0 |
| DHX36 | inf | 0.201627102 | 2 | 0 |
| CSRP2 | inf | 0.201627102 | 2 | 0 |
| NOLC1 | inf | 0.201627102 | 2 | 0 |
| CSRP1 | inf | 0.201627102 | 2 | 0 |
| NOL7 | inf | 0.201627102 | 2 | 0 |
| OR52K2 | inf | 0.201627102 | 2 | 0 |
| SERGEF | inf | 0.201627102 | 2 | 0 |
| RPP14 | inf | 0.201627102 | 2 | 0 |
| SERINC5 | inf | 0.201627102 | 2 | 0 |
| CSF2RB | inf | 0.201627102 | 2 | 0 |
| TIGD5 | inf | 0.201627102 | 2 | 0 |
| CSF1 | inf | 0.201627102 | 2 | 0 |
| HSPB9 | inf | 0.201627102 | 2 | 0 |
| AGBL2 | inf | 0.201627102 | 2 | 0 |
| ZNF580 | inf | 0.201627102 | 2 | 0 |
| NOTO | inf | 0.201627102 | 2 | 0 |
| FAM120AOS | inf | 0.201627102 | 2 | 0 |
| FAM120A | inf | 0.201627102 | 2 | 0 |
| RNLS | inf | 0.201627102 | 2 | 0 |
| OS9 | inf | 0.201627102 | 2 | 0 |
| SLC9A8 | inf | 0.201627102 | 2 | 0 |
| ZNF486 | inf | 0.201627102 | 2 | 0 |
| GPN3 | inf | 0.201627102 | 2 | 0 |
| COMMD3-BMI1 | inf | 0.201627102 | 2 | 0 |
| ALOX15 | inf | 0.201627102 | 2 | 0 |
| FADS6 | inf | 0.201627102 | 2 | 0 |
| GPI | inf | 0.201627102 | 2 | 0 |
| GPHN | inf | 0.201627102 | 2 | 0 |
| NDUFV3 | inf | 0.201627102 | 2 | 0 |
| TMEM106C | inf | 0.201627102 | 2 | 0 |
| OR9Q2 | inf | 0.201627102 | 2 | 0 |
| RNF220 | inf | 0.201627102 | 2 | 0 |
| OTUD7A | inf | 0.201627102 | 2 | 0 |
| SPAG9 | inf | 0.201627102 | 2 | 0 |
| TMEM120A | inf | 0.201627102 | 2 | 0 |
| ZNF467 | inf | 0.201627102 | 2 | 0 |
| TMEM125 | inf | 0.201627102 | 2 | 0 |
| RNF217 | inf | 0.201627102 | 2 | 0 |
| JAML | inf | 0.201627102 | 2 | 0 |
| IGKV1D-12 | inf | 0.201627102 | 2 | 0 |
| ZNF445 | inf | 0.201627102 | 2 | 0 |
| TMEM126B | inf | 0.201627102 | 2 | 0 |
| OXA1L | inf | 0.201627102 | 2 | 0 |
| OXCT1 | inf | 0.201627102 | 2 | 0 |
| ZNF470 | inf | 0.201627102 | 2 | 0 |
| ZNF518A | inf | 0.201627102 | 2 | 0 |
| IGFLR1 | inf | 0.201627102 | 2 | 0 |
| DMRTA2 | inf | 0.201627102 | 2 | 0 |
| IDO1 | inf | 0.201627102 | 2 | 0 |
| OR5W2 | inf | 0.201627102 | 2 | 0 |
| RPA4 | inf | 0.201627102 | 2 | 0 |
| DLL1 | inf | 0.201627102 | 2 | 0 |
| NHLRC2 | inf | 0.201627102 | 2 | 0 |
| SLFN11 | inf | 0.201627102 | 2 | 0 |
| SOX8 | inf | 0.201627102 | 2 | 0 |
| SOX9 | inf | 0.201627102 | 2 | 0 |
| SERPINB5 | inf | 0.201627102 | 2 | 0 |
| FAM111B | inf | 0.201627102 | 2 | 0 |
| CPNE9 | inf | 0.201627102 | 2 | 0 |
| CPNE5 | inf | 0.201627102 | 2 | 0 |
| DLX6 | inf | 0.201627102 | 2 | 0 |
| CPEB1 | inf | 0.201627102 | 2 | 0 |
| FBXL5 | inf | 0.201627102 | 2 | 0 |
| IFNGR1 | inf | 0.201627102 | 2 | 0 |
| NFIB | inf | 0.201627102 | 2 | 0 |
| ZNF547 | inf | 0.201627102 | 2 | 0 |
| DMKN | inf | 0.201627102 | 2 | 0 |
| NFE4 | inf | 0.201627102 | 2 | 0 |
| ALDH1L1 | inf | 0.201627102 | 2 | 0 |
| OR8B2 | inf | 0.201627102 | 2 | 0 |
| SLC9C2 | inf | 0.201627102 | 2 | 0 |
| CORO1A | inf | 0.201627102 | 2 | 0 |
| DMRT3 | inf | 0.201627102 | 2 | 0 |
| SHKBP1 | inf | 0.201627102 | 2 | 0 |
| IGFBP1 | inf | 0.201627102 | 2 | 0 |
| OR5T1 | inf | 0.201627102 | 2 | 0 |
| OR52A1 | inf | 0.201627102 | 2 | 0 |
| NOXO1 | inf | 0.201627102 | 2 | 0 |
| RRNAD1 | inf | 0.201627102 | 2 | 0 |
| NXF3 | inf | 0.201627102 | 2 | 0 |
| SNRNP35 | inf | 0.201627102 | 2 | 0 |
| DCAF6 | inf | 0.201627102 | 2 | 0 |
| NUSAP1 | inf | 0.201627102 | 2 | 0 |
| DCAF4L2 | inf | 0.201627102 | 2 | 0 |
| NUPR2 | inf | 0.201627102 | 2 | 0 |
| SCAP | inf | 0.201627102 | 2 | 0 |
| SCAND1 | inf | 0.201627102 | 2 | 0 |
| SNRPA | inf | 0.201627102 | 2 | 0 |
| ABR | inf | 0.201627102 | 2 | 0 |
| OR2A5 | inf | 0.201627102 | 2 | 0 |
| GSDMB | inf | 0.201627102 | 2 | 0 |
| NXN | inf | 0.201627102 | 2 | 0 |
| SCAMP2 | inf | 0.201627102 | 2 | 0 |
| TDRD5 | inf | 0.201627102 | 2 | 0 |
| HEBP1 | inf | 0.201627102 | 2 | 0 |
| SNX15 | inf | 0.201627102 | 2 | 0 |
| NUDT19 | inf | 0.201627102 | 2 | 0 |
| SMIM2 | inf | 0.201627102 | 2 | 0 |
| HEPH | inf | 0.201627102 | 2 | 0 |
| TECRL | inf | 0.201627102 | 2 | 0 |
| SAPCD1 | inf | 0.201627102 | 2 | 0 |
| GRK7 | inf | 0.201627102 | 2 | 0 |
| HES4 | inf | 0.201627102 | 2 | 0 |
| TBC1D24 | inf | 0.201627102 | 2 | 0 |
| TBC1D23 | inf | 0.201627102 | 2 | 0 |
| SBSN | inf | 0.201627102 | 2 | 0 |
| HBS1L | inf | 0.201627102 | 2 | 0 |
| SCFD1 | inf | 0.201627102 | 2 | 0 |
| DCLK1 | inf | 0.201627102 | 2 | 0 |
| OR10C1 | inf | 0.201627102 | 2 | 0 |
| FAM214A | inf | 0.201627102 | 2 | 0 |
| DEFB105A | inf | 0.201627102 | 2 | 0 |
| OPHN1 | inf | 0.201627102 | 2 | 0 |
| OPALIN | inf | 0.201627102 | 2 | 0 |
| ONECUT2 | inf | 0.201627102 | 2 | 0 |
| OR10J5 | inf | 0.201627102 | 2 | 0 |
| OIT3 | inf | 0.201627102 | 2 | 0 |
| SDE2 | inf | 0.201627102 | 2 | 0 |
| TCAP | inf | 0.201627102 | 2 | 0 |
| GZMK | inf | 0.201627102 | 2 | 0 |
| ODF2L | inf | 0.201627102 | 2 | 0 |
| DDOST | inf | 0.201627102 | 2 | 0 |
| ABCB10 | inf | 0.201627102 | 2 | 0 |
| OR13C4 | inf | 0.201627102 | 2 | 0 |
| ZSWIM2 | inf | 0.201627102 | 2 | 0 |
| DDB2 | inf | 0.201627102 | 2 | 0 |
| SDR42E1 | inf | 0.201627102 | 2 | 0 |
| HAPLN1 | inf | 0.201627102 | 2 | 0 |
| OBP2A | inf | 0.201627102 | 2 | 0 |
| HAUS4 | inf | 0.201627102 | 2 | 0 |
| OR14I1 | inf | 0.201627102 | 2 | 0 |
| OAS2 | inf | 0.201627102 | 2 | 0 |
| ABCF3 | inf | 0.201627102 | 2 | 0 |
| SCIN | inf | 0.201627102 | 2 | 0 |
| TCN1 | inf | 0.201627102 | 2 | 0 |
| KRT89P | inf | 0.201627102 | 2 | 0 |
| CYTH3 | inf | 0.201627102 | 2 | 0 |
| NUBPL | inf | 0.201627102 | 2 | 0 |
| HGD | inf | 0.201627102 | 2 | 0 |
| ZNF821 | inf | 0.201627102 | 2 | 0 |
| NR1D2 | inf | 0.201627102 | 2 | 0 |
| ADAD2 | inf | 0.201627102 | 2 | 0 |
| CYC1 | inf | 0.201627102 | 2 | 0 |
| NPY4R | inf | 0.201627102 | 2 | 0 |
| ADAM2 | inf | 0.201627102 | 2 | 0 |
| ADAM20 | inf | 0.201627102 | 2 | 0 |
| OR4M2 | inf | 0.201627102 | 2 | 0 |
| OR4N4 | inf | 0.201627102 | 2 | 0 |
| NPRL3 | inf | 0.201627102 | 2 | 0 |
| ZNF775 | inf | 0.201627102 | 2 | 0 |
| TFIP11 | inf | 0.201627102 | 2 | 0 |
| CYB561 | inf | 0.201627102 | 2 | 0 |
| TFRC | inf | 0.201627102 | 2 | 0 |
| HMGXB3 | inf | 0.201627102 | 2 | 0 |
| HMGXB4 | inf | 0.201627102 | 2 | 0 |
| RUNX2 | inf | 0.201627102 | 2 | 0 |
| OR51E1 | inf | 0.201627102 | 2 | 0 |
| TGM4 | inf | 0.201627102 | 2 | 0 |
| CUTA | inf | 0.201627102 | 2 | 0 |
| DHRS2 | inf | 0.201627102 | 2 | 0 |
| SENP7 | inf | 0.201627102 | 2 | 0 |
| FAM157B | inf | 0.201627102 | 2 | 0 |
| THAP10 | inf | 0.201627102 | 2 | 0 |
| ADARB2 | inf | 0.201627102 | 2 | 0 |
| SEPT14 | inf | 0.201627102 | 2 | 0 |
| SAXO1 | inf | 0.201627102 | 2 | 0 |
| HPD | inf | 0.201627102 | 2 | 0 |
| TBC1D14 | inf | 0.201627102 | 2 | 0 |
| TMEM129 | inf | 0.201627102 | 2 | 0 |
| NR1H4 | inf | 0.201627102 | 2 | 0 |
| S1PR5 | inf | 0.201627102 | 2 | 0 |
| ACOT7 | inf | 0.201627102 | 2 | 0 |
| ACOX2 | inf | 0.201627102 | 2 | 0 |
| FAM180A | inf | 0.201627102 | 2 | 0 |
| GRIN3B | inf | 0.201627102 | 2 | 0 |
| HIF1A | inf | 0.201627102 | 2 | 0 |
| HIF1AN | inf | 0.201627102 | 2 | 0 |
| ZNF808 | inf | 0.201627102 | 2 | 0 |
| DGAT2 | inf | 0.201627102 | 2 | 0 |
| HIP1 | inf | 0.201627102 | 2 | 0 |
| HIP1R | inf | 0.201627102 | 2 | 0 |
| SNX33 | inf | 0.201627102 | 2 | 0 |
| ACSL5 | inf | 0.201627102 | 2 | 0 |
| CYP2F1 | inf | 0.201627102 | 2 | 0 |
| ESS2 | inf | 0.201627102 | 2 | 0 |
| ACSS2 | inf | 0.201627102 | 2 | 0 |
| NRTN | inf | 0.201627102 | 2 | 0 |
| HIST1H2BA | inf | 0.201627102 | 2 | 0 |
| GRIK5 | inf | 0.201627102 | 2 | 0 |
| NRIP3 | inf | 0.201627102 | 2 | 0 |
| FAM71F2 | inf | 0.201627102 | 2 | 0 |
| PPP4R3B | inf | 0.201627102 | 2 | 0 |
| SMCR9 | inf | 0.201627102 | 2 | 0 |
| SELENOK | inf | 0.201627102 | 2 | 0 |
| TEX26 | inf | 0.201627102 | 2 | 0 |
| HIST2H2BF | inf | 0.201627102 | 2 | 0 |
| NR4A3 | inf | 0.201627102 | 2 | 0 |
| NR3C1 | inf | 0.201627102 | 2 | 0 |
| OR4F4 | inf | 0.201627102 | 2 | 0 |
| RNF180 | inf | 0.201627102 | 2 | 0 |
| FZD10 | inf | 0.201627102 | 2 | 0 |
| SPATA19 | inf | 0.201627102 | 2 | 0 |
| ITM2C | inf | 0.201627102 | 2 | 0 |
| TMTC3 | inf | 0.201627102 | 2 | 0 |
| RECQL | inf | 0.201627102 | 2 | 0 |
| AQP5 | inf | 0.201627102 | 2 | 0 |
| ASAP3 | inf | 0.201627102 | 2 | 0 |
| ASAP2 | inf | 0.201627102 | 2 | 0 |
| DOK4 | inf | 0.201627102 | 2 | 0 |
| CHN2 | inf | 0.201627102 | 2 | 0 |
| SGO1 | inf | 0.201627102 | 2 | 0 |
| RCOR1 | inf | 0.201627102 | 2 | 0 |
| GLCCI1 | inf | 0.201627102 | 2 | 0 |
| GNAI2 | inf | 0.201627102 | 2 | 0 |
| FH | inf | 0.201627102 | 2 | 0 |
| KDM3B | inf | 0.201627102 | 2 | 0 |
| PCDHGA1 | inf | 0.201627102 | 2 | 0 |
| PDLIM7 | inf | 0.201627102 | 2 | 0 |
| PDLIM5 | inf | 0.201627102 | 2 | 0 |
| SYPL1 | inf | 0.201627102 | 2 | 0 |
| MTSS1L | inf | 0.201627102 | 2 | 0 |
| RARB | inf | 0.201627102 | 2 | 0 |
| RARS | inf | 0.201627102 | 2 | 0 |
| TRAFD1 | inf | 0.201627102 | 2 | 0 |
| SPHK2 | inf | 0.201627102 | 2 | 0 |
| KDM6B | inf | 0.201627102 | 2 | 0 |
| GJD3 | inf | 0.201627102 | 2 | 0 |
| ERRFI1 | inf | 0.201627102 | 2 | 0 |
| PCDH20 | inf | 0.201627102 | 2 | 0 |
| APLP1 | inf | 0.201627102 | 2 | 0 |
| IRX5 | inf | 0.201627102 | 2 | 0 |
| NAXE | inf | 0.201627102 | 2 | 0 |
| RUBCNL | inf | 0.201627102 | 2 | 0 |
| CDKAL1 | inf | 0.201627102 | 2 | 0 |
| REXO2 | inf | 0.201627102 | 2 | 0 |
| MRPS7 | inf | 0.201627102 | 2 | 0 |
| ZDHHC21 | inf | 0.201627102 | 2 | 0 |
| TNIK | inf | 0.201627102 | 2 | 0 |
| TRAPPC6B | inf | 0.201627102 | 2 | 0 |
| REV1 | inf | 0.201627102 | 2 | 0 |
| RANBP3L | inf | 0.201627102 | 2 | 0 |
| CHST9 | inf | 0.201627102 | 2 | 0 |
| CDKL5 | inf | 0.201627102 | 2 | 0 |
| MPO | inf | 0.201627102 | 2 | 0 |
| MYBPC1 | inf | 0.201627102 | 2 | 0 |
| PDZK1 | inf | 0.201627102 | 2 | 0 |
| ITGAL | inf | 0.201627102 | 2 | 0 |
| ITGB1BP1 | inf | 0.201627102 | 2 | 0 |
| MPP7 | inf | 0.201627102 | 2 | 0 |
| GNPAT | inf | 0.201627102 | 2 | 0 |
| ARSH | inf | 0.201627102 | 2 | 0 |
| PDLIM3 | inf | 0.201627102 | 2 | 0 |
| JUND | inf | 0.201627102 | 2 | 0 |
| MTA1 | inf | 0.201627102 | 2 | 0 |
| KCNB1 | inf | 0.201627102 | 2 | 0 |
| TOMM70 | inf | 0.201627102 | 2 | 0 |
| ARMCX5-GPRASP2 | inf | 0.201627102 | 2 | 0 |
| SH3KBP1 | inf | 0.201627102 | 2 | 0 |
| TOP1MT | inf | 0.201627102 | 2 | 0 |
| TOP3A | inf | 0.201627102 | 2 | 0 |
| GLS2 | inf | 0.201627102 | 2 | 0 |
| PDE8B | inf | 0.201627102 | 2 | 0 |
| ETV4 | inf | 0.201627102 | 2 | 0 |
| RBM23 | inf | 0.201627102 | 2 | 0 |
| TPI1 | inf | 0.201627102 | 2 | 0 |
| KCND1 | inf | 0.201627102 | 2 | 0 |
| KCNH1 | inf | 0.201627102 | 2 | 0 |
| MRPS15 | inf | 0.201627102 | 2 | 0 |
| FGF11 | inf | 0.201627102 | 2 | 0 |
| TPCN2 | inf | 0.201627102 | 2 | 0 |
| DPY19L2 | inf | 0.201627102 | 2 | 0 |
| DPYSL3 | inf | 0.201627102 | 2 | 0 |
| KCNJ14 | inf | 0.201627102 | 2 | 0 |
| SYCP1 | inf | 0.201627102 | 2 | 0 |
| SH3D19 | inf | 0.201627102 | 2 | 0 |
| SPDYE1 | inf | 0.201627102 | 2 | 0 |
| DNTTIP2 | inf | 0.201627102 | 2 | 0 |
| CFHR2 | inf | 0.201627102 | 2 | 0 |
| CFI | inf | 0.201627102 | 2 | 0 |
| CHCHD4 | inf | 0.201627102 | 2 | 0 |
| MTNR1B | inf | 0.201627102 | 2 | 0 |
| JUP | inf | 0.201627102 | 2 | 0 |
| ADA2 | inf | 0.201627102 | 2 | 0 |
| ARHGAP25 | inf | 0.201627102 | 2 | 0 |
| MTMR9 | inf | 0.201627102 | 2 | 0 |
| TPRG1L | inf | 0.201627102 | 2 | 0 |
| ZNF107 | inf | 0.201627102 | 2 | 0 |
| SYNGAP1 | inf | 0.201627102 | 2 | 0 |
| ZMYND10 | inf | 0.201627102 | 2 | 0 |
| CELF1 | inf | 0.201627102 | 2 | 0 |
| GMEB1 | inf | 0.201627102 | 2 | 0 |
| RASAL1 | inf | 0.201627102 | 2 | 0 |
| MTHFD1 | inf | 0.201627102 | 2 | 0 |
| RBM43 | inf | 0.201627102 | 2 | 0 |
| ZFP14 | inf | 0.201627102 | 2 | 0 |
| PDF | inf | 0.201627102 | 2 | 0 |
| PCDHGC5 | inf | 0.201627102 | 2 | 0 |
| MTF2 | inf | 0.201627102 | 2 | 0 |
| SH2D4B | inf | 0.201627102 | 2 | 0 |
| C20orf197 | inf | 0.201627102 | 2 | 0 |
| ARHGEF19 | inf | 0.201627102 | 2 | 0 |
| PCDHGC4 | inf | 0.201627102 | 2 | 0 |
| PELI1 | inf | 0.201627102 | 2 | 0 |
| PCDH8 | inf | 0.201627102 | 2 | 0 |
| MOS | inf | 0.201627102 | 2 | 0 |
| ZNF397 | inf | 0.201627102 | 2 | 0 |
| ATE1 | inf | 0.201627102 | 2 | 0 |
| RMI1 | inf | 0.201627102 | 2 | 0 |
| CNNM1 | inf | 0.201627102 | 2 | 0 |
| DNAJB8 | inf | 0.201627102 | 2 | 0 |
| CDH18 | inf | 0.201627102 | 2 | 0 |
| SF3B2 | inf | 0.201627102 | 2 | 0 |
| IL36B | inf | 0.201627102 | 2 | 0 |
| FAM234B | inf | 0.201627102 | 2 | 0 |
| GPATCH2 | inf | 0.201627102 | 2 | 0 |
| SLC6A13 | inf | 0.201627102 | 2 | 0 |
| ASXL2 | inf | 0.201627102 | 2 | 0 |
| IL7R | inf | 0.201627102 | 2 | 0 |
| CLVS1 | inf | 0.201627102 | 2 | 0 |
| ANKRD66 | inf | 0.201627102 | 2 | 0 |
| MYL6 | inf | 0.201627102 | 2 | 0 |
| SLC38A11 | inf | 0.201627102 | 2 | 0 |
| CLTA | inf | 0.201627102 | 2 | 0 |
| ASTN1 | inf | 0.201627102 | 2 | 0 |
| SPINT2 | inf | 0.201627102 | 2 | 0 |
| TMEM208 | inf | 0.201627102 | 2 | 0 |
| TMEM232 | inf | 0.201627102 | 2 | 0 |
| NBPF3 | inf | 0.201627102 | 2 | 0 |
| RNF103 | inf | 0.201627102 | 2 | 0 |
| GIPR | inf | 0.201627102 | 2 | 0 |
| SPATA22 | inf | 0.201627102 | 2 | 0 |
| ERCC6L2 | inf | 0.201627102 | 2 | 0 |
| ANAPC5 | inf | 0.201627102 | 2 | 0 |
| SLC7A10 | inf | 0.201627102 | 2 | 0 |
| F13B | inf | 0.201627102 | 2 | 0 |
| NCOA2 | inf | 0.201627102 | 2 | 0 |
| ATG16L2 | inf | 0.201627102 | 2 | 0 |
| IGLV1-36 | inf | 0.201627102 | 2 | 0 |
| TMEM176B | inf | 0.201627102 | 2 | 0 |
| COL10A1 | inf | 0.201627102 | 2 | 0 |
| NCDN | inf | 0.201627102 | 2 | 0 |
| TMEM150C | inf | 0.201627102 | 2 | 0 |
| RNF133 | inf | 0.201627102 | 2 | 0 |
| COASY | inf | 0.201627102 | 2 | 0 |
| ZC3HAV1 | inf | 0.201627102 | 2 | 0 |
| TMEM161A | inf | 0.201627102 | 2 | 0 |
| RAD23A | inf | 0.201627102 | 2 | 0 |
| GPC1 | inf | 0.201627102 | 2 | 0 |
| MNS1 | inf | 0.201627102 | 2 | 0 |
| COG7 | inf | 0.201627102 | 2 | 0 |
| DNAJC9 | inf | 0.201627102 | 2 | 0 |
| PDE1C | inf | 0.201627102 | 2 | 0 |
| DNALI1 | inf | 0.201627102 | 2 | 0 |
| ANTXR2 | inf | 0.201627102 | 2 | 0 |
| CDIP1 | inf | 0.201627102 | 2 | 0 |
| CLEC1A | inf | 0.201627102 | 2 | 0 |
| AP1M1 | inf | 0.201627102 | 2 | 0 |
| CDK11A | inf | 0.201627102 | 2 | 0 |
| CDK12 | inf | 0.201627102 | 2 | 0 |
| IP6K1 | inf | 0.201627102 | 2 | 0 |
| AP3B2 | inf | 0.201627102 | 2 | 0 |
| IPO11 | inf | 0.201627102 | 2 | 0 |
| ZNF28 | inf | 0.201627102 | 2 | 0 |
| RTL5 | inf | 0.201627102 | 2 | 0 |
| PIP4P1 | inf | 0.201627102 | 2 | 0 |
| AP5M1 | inf | 0.201627102 | 2 | 0 |
| RFX6 | inf | 0.201627102 | 2 | 0 |
| PAX8 | inf | 0.201627102 | 2 | 0 |
| TMEM63A | inf | 0.201627102 | 2 | 0 |
| PBRM1 | inf | 0.201627102 | 2 | 0 |
| CDK13 | inf | 0.201627102 | 2 | 0 |
| IRAK1 | inf | 0.201627102 | 2 | 0 |
| TMEM70 | inf | 0.201627102 | 2 | 0 |
| CLDN4 | inf | 0.201627102 | 2 | 0 |
| RAD54L2 | inf | 0.201627102 | 2 | 0 |
| MYRFL | inf | 0.201627102 | 2 | 0 |
| DNASE2 | inf | 0.201627102 | 2 | 0 |
| ANXA10 | inf | 0.201627102 | 2 | 0 |
| ASS1 | inf | 0.201627102 | 2 | 0 |
| TRAV6 | inf | 0.201627102 | 2 | 0 |
| CLEC4M | inf | 0.201627102 | 2 | 0 |
| INSIG1 | inf | 0.201627102 | 2 | 0 |
| PRKN | inf | 0.201627102 | 2 | 0 |
| RGS20 | inf | 0.201627102 | 2 | 0 |
| IGFN1 | 1.4765625 | 0.201986349 | 18 | 16 |
| NPIPA1 | 1.482051282 | 0.207284106 | 17 | 15 |
| CNTNAP3 | 1.482051282 | 0.207284106 | 17 | 15 |
| POTEJ | 1.332549941 | 0.213738448 | 36 | 37 |
| HLA-DRB5 | 1.928571429 | 0.21786575 | 78 | 91 |
| DNAH11 | 1.498277842 | 0.217881874 | 15 | 13 |
| ANKLE1 | 1.366946779 | 0.218002235 | 61 | 68 |
| POM121C | 1.382126348 | 0.220862652 | 23 | 22 |
| TBC1D3F | 1.31675243 | 0.222682029 | 49 | 53 |
| TRBV5-4 | 1.307602339 | 0.233971709 | 52 | 57 |
| PER3 | 1.600985222 | 0.237214993 | 75 | 87 |
| TBC1D3C | 1.566510172 | 0.238502968 | 11 | 9 |
| GRIN3A | 3.759493671 | 0.239799831 | 3 | 1 |
| GLIPR1L2 | 3.759493671 | 0.239799831 | 3 | 1 |
| CYP2W1 | 3.759493671 | 0.239799831 | 3 | 1 |
| TCF7L1 | 3.759493671 | 0.239799831 | 3 | 1 |
| CENPE | 3.759493671 | 0.239799831 | 3 | 1 |
| SUPV3L1 | 3.759493671 | 0.239799831 | 3 | 1 |
| TBC1D30 | 3.759493671 | 0.239799831 | 3 | 1 |
| SLC23A3 | 3.759493671 | 0.239799831 | 3 | 1 |
| KIF20B | 3.759493671 | 0.239799831 | 3 | 1 |
| CDK20 | 3.759493671 | 0.239799831 | 3 | 1 |
| SOS1 | 3.759493671 | 0.239799831 | 3 | 1 |
| TET1 | 3.759493671 | 0.239799831 | 3 | 1 |
| LOC101060175 | 3.759493671 | 0.239799831 | 3 | 1 |
| CDYL2 | 3.759493671 | 0.239799831 | 3 | 1 |
| KIF21B | 3.759493671 | 0.239799831 | 3 | 1 |
| TEKT1 | 3.759493671 | 0.239799831 | 3 | 1 |
| GAA | 3.759493671 | 0.239799831 | 3 | 1 |
| DDR1 | 3.759493671 | 0.239799831 | 3 | 1 |
| CEBPA | 3.759493671 | 0.239799831 | 3 | 1 |
| CDH5 | 3.759493671 | 0.239799831 | 3 | 1 |
| TBRG4 | 3.759493671 | 0.239799831 | 3 | 1 |
| CDH16 | 3.759493671 | 0.239799831 | 3 | 1 |
| RASAL3 | 3.759493671 | 0.239799831 | 3 | 1 |
| TCTN2 | 3.759493671 | 0.239799831 | 3 | 1 |
| SEC24D | 3.759493671 | 0.239799831 | 3 | 1 |
| LOC101928050 | 3.759493671 | 0.239799831 | 3 | 1 |
| GTF2F1 | 3.759493671 | 0.239799831 | 3 | 1 |
| CDHR2 | 3.759493671 | 0.239799831 | 3 | 1 |
| H6PD | 3.759493671 | 0.239799831 | 3 | 1 |
| CCDC191 | 3.759493671 | 0.239799831 | 3 | 1 |
| DCAF15 | 3.759493671 | 0.239799831 | 3 | 1 |
| HEG1 | 3.759493671 | 0.239799831 | 3 | 1 |
| GSDMA | 3.759493671 | 0.239799831 | 3 | 1 |
| NCOA2 | 3.759493671 | 0.239799831 | 3 | 1 |
| CHD2 | 3.759493671 | 0.239799831 | 3 | 1 |
| SLC22A16 | 3.759493671 | 0.239799831 | 3 | 1 |
| DISP3 | 3.759493671 | 0.239799831 | 3 | 1 |
| CPT1A | 3.759493671 | 0.239799831 | 3 | 1 |
| DLGAP5 | 3.759493671 | 0.239799831 | 3 | 1 |
| DLGAP2 | 3.759493671 | 0.239799831 | 3 | 1 |
| KRT76 | 3.759493671 | 0.239799831 | 3 | 1 |
| CARS2 | 3.759493671 | 0.239799831 | 3 | 1 |
| ECM2 | 3.759493671 | 0.239799831 | 3 | 1 |
| TRUB1 | 3.759493671 | 0.239799831 | 3 | 1 |
| DLG5 | 3.759493671 | 0.239799831 | 3 | 1 |
| CCDC80 | 3.759493671 | 0.239799831 | 3 | 1 |
| CAPN8 | 3.759493671 | 0.239799831 | 3 | 1 |
| PROB1 | 3.759493671 | 0.239799831 | 3 | 1 |
| TKTL1 | 3.759493671 | 0.239799831 | 3 | 1 |
| IRAK3 | 3.759493671 | 0.239799831 | 3 | 1 |
| TJP1 | 3.759493671 | 0.239799831 | 3 | 1 |
| CCDC88C | 3.759493671 | 0.239799831 | 3 | 1 |
| GPR180 | 3.759493671 | 0.239799831 | 3 | 1 |
| TMEM8A | 3.759493671 | 0.239799831 | 3 | 1 |
| GPR182 | 3.759493671 | 0.239799831 | 3 | 1 |
| HTR2B | 3.759493671 | 0.239799831 | 3 | 1 |
| CAMSAP2 | 3.759493671 | 0.239799831 | 3 | 1 |
| TMEM260 | 3.759493671 | 0.239799831 | 3 | 1 |
| INO80C | 3.759493671 | 0.239799831 | 3 | 1 |
| TAAR5 | 3.759493671 | 0.239799831 | 3 | 1 |
| PTCD3 | 3.759493671 | 0.239799831 | 3 | 1 |
| RNF207 | 3.759493671 | 0.239799831 | 3 | 1 |
| PRTG | 3.759493671 | 0.239799831 | 3 | 1 |
| PSG4 | 3.759493671 | 0.239799831 | 3 | 1 |
| PRSS42 | 3.759493671 | 0.239799831 | 3 | 1 |
| RNF219 | 3.759493671 | 0.239799831 | 3 | 1 |
| RNF222 | 3.759493671 | 0.239799831 | 3 | 1 |
| SLC12A5 | 3.759493671 | 0.239799831 | 3 | 1 |
| ELP1 | 3.759493671 | 0.239799831 | 3 | 1 |
| TTC31 | 3.759493671 | 0.239799831 | 3 | 1 |
| IGHG3 | 3.759493671 | 0.239799831 | 3 | 1 |
| PRKCZ | 3.759493671 | 0.239799831 | 3 | 1 |
| SEZ6 | 3.759493671 | 0.239799831 | 3 | 1 |
| RIIAD1 | 3.759493671 | 0.239799831 | 3 | 1 |
| IL6ST | 3.759493671 | 0.239799831 | 3 | 1 |
| SERPINC1 | 3.759493671 | 0.239799831 | 3 | 1 |
| TTC6 | 3.759493671 | 0.239799831 | 3 | 1 |
| CCDC34 | 3.759493671 | 0.239799831 | 3 | 1 |
| IFT140 | 3.759493671 | 0.239799831 | 3 | 1 |
| SUCNR1 | 3.759493671 | 0.239799831 | 3 | 1 |
| RHPN1 | 3.759493671 | 0.239799831 | 3 | 1 |
| LBX2 | 3.759493671 | 0.239799831 | 3 | 1 |
| IMPG2 | 3.759493671 | 0.239799831 | 3 | 1 |
| RIOK2 | 3.759493671 | 0.239799831 | 3 | 1 |
| REST | 3.759493671 | 0.239799831 | 3 | 1 |
| ITGAD | 3.759493671 | 0.239799831 | 3 | 1 |
| CAMKK1 | 3.759493671 | 0.239799831 | 3 | 1 |
| HOXD11 | 3.759493671 | 0.239799831 | 3 | 1 |
| GPRASP1 | 3.759493671 | 0.239799831 | 3 | 1 |
| SIPA1L1 | 3.759493671 | 0.239799831 | 3 | 1 |
| TH | 3.759493671 | 0.239799831 | 3 | 1 |
| CWF19L2 | 3.759493671 | 0.239799831 | 3 | 1 |
| TGFBI | 3.759493671 | 0.239799831 | 3 | 1 |
| TRIO | 3.759493671 | 0.239799831 | 3 | 1 |
| SEMA5A | 3.759493671 | 0.239799831 | 3 | 1 |
| UGT1A8 | 3.759493671 | 0.239799831 | 3 | 1 |
| SEMA4B | 3.759493671 | 0.239799831 | 3 | 1 |
| RNF17 | 3.759493671 | 0.239799831 | 3 | 1 |
| SPOUT1 | 3.759493671 | 0.239799831 | 3 | 1 |
| TFAP2E | 3.759493671 | 0.239799831 | 3 | 1 |
| SIK3 | 3.759493671 | 0.239799831 | 3 | 1 |
| SELP | 3.759493671 | 0.239799831 | 3 | 1 |
| TOX | 3.759493671 | 0.239799831 | 3 | 1 |
| GLRA3 | 3.759493671 | 0.239799831 | 3 | 1 |
| TRIM6 | 3.759493671 | 0.239799831 | 3 | 1 |
| CFAP206 | 3.759493671 | 0.239799831 | 3 | 1 |
| CYP27A1 | 3.759493671 | 0.239799831 | 3 | 1 |
| GRIN2B | 3.759493671 | 0.239799831 | 3 | 1 |
| HIST1H1A | 3.759493671 | 0.239799831 | 3 | 1 |
| ULK4P1 | 3.759493671 | 0.239799831 | 3 | 1 |
| DPYSL2 | 3.759493671 | 0.239799831 | 3 | 1 |
| SLC1A4 | 3.759493671 | 0.239799831 | 3 | 1 |
| CTRB2 | 3.759493671 | 0.239799831 | 3 | 1 |
| TMOD4 | 3.759493671 | 0.239799831 | 3 | 1 |
| ITGB2 | 3.759493671 | 0.239799831 | 3 | 1 |
| TMPRSS15 | 3.759493671 | 0.239799831 | 3 | 1 |
| GAS1 | 3.759493671 | 0.239799831 | 3 | 1 |
| HSPA4 | 3.759493671 | 0.239799831 | 3 | 1 |
| U2AF1L4 | 3.759493671 | 0.239799831 | 3 | 1 |
| TIGD1 | 3.759493671 | 0.239799831 | 3 | 1 |
| TIAM1 | 3.759493671 | 0.239799831 | 3 | 1 |
| DOCK9 | 3.759493671 | 0.239799831 | 3 | 1 |
| CHRNA7 | 3.759493671 | 0.239799831 | 3 | 1 |
| CTSA | 3.759493671 | 0.239799831 | 3 | 1 |
| REC8 | 3.759493671 | 0.239799831 | 3 | 1 |
| JAG1 | 3.759493671 | 0.239799831 | 3 | 1 |
| DDX11 | 3.759493671 | 0.239799831 | 3 | 1 |
| CRACR2B | 3.759493671 | 0.239799831 | 3 | 1 |
| CTBP2 | 3.759493671 | 0.239799831 | 3 | 1 |
| TNFRSF19 | 3.759493671 | 0.239799831 | 3 | 1 |
| JMJD4 | 3.759493671 | 0.239799831 | 3 | 1 |
| JMJD8 | 3.759493671 | 0.239799831 | 3 | 1 |
| HRC | 3.759493671 | 0.239799831 | 3 | 1 |
| KLK5 | 3.759493671 | 0.239799831 | 3 | 1 |
| LLGL2 | 3.759493671 | 0.239799831 | 3 | 1 |
| GANC | 3.759493671 | 0.239799831 | 3 | 1 |
| IGFALS | 3.759493671 | 0.239799831 | 3 | 1 |
| C3AR1 | 3.759493671 | 0.239799831 | 3 | 1 |
| LY75-CD302 | 3.759493671 | 0.239799831 | 3 | 1 |
| VEPH1 | 3.759493671 | 0.239799831 | 3 | 1 |
| AGXT | 3.759493671 | 0.239799831 | 3 | 1 |
| ANKRD53 | 3.759493671 | 0.239799831 | 3 | 1 |
| PLCL1 | 3.759493671 | 0.239799831 | 3 | 1 |
| ADGRB1 | 3.759493671 | 0.239799831 | 3 | 1 |
| NAP1L1 | 3.759493671 | 0.239799831 | 3 | 1 |
| NCCRP1 | 3.759493671 | 0.239799831 | 3 | 1 |
| LRRC37B | 3.759493671 | 0.239799831 | 3 | 1 |
| PCIF1 | 3.759493671 | 0.239799831 | 3 | 1 |
| SPICE1 | 3.759493671 | 0.239799831 | 3 | 1 |
| ZNF423 | 3.759493671 | 0.239799831 | 3 | 1 |
| ALOX15B | 3.759493671 | 0.239799831 | 3 | 1 |
| ATL3 | 3.759493671 | 0.239799831 | 3 | 1 |
| VCPIP1 | 3.759493671 | 0.239799831 | 3 | 1 |
| PLCB3 | 3.759493671 | 0.239799831 | 3 | 1 |
| NXPE1 | 3.759493671 | 0.239799831 | 3 | 1 |
| VCAM1 | 3.759493671 | 0.239799831 | 3 | 1 |
| PDZRN4 | 3.759493671 | 0.239799831 | 3 | 1 |
| C18orf63 | 3.759493671 | 0.239799831 | 3 | 1 |
| SLC2A10 | 3.759493671 | 0.239799831 | 3 | 1 |
| NCF1 | 3.759493671 | 0.239799831 | 3 | 1 |
| ZNF613 | 3.759493671 | 0.239799831 | 3 | 1 |
| PCDHGA7 | 3.759493671 | 0.239799831 | 3 | 1 |
| AGMO | 3.759493671 | 0.239799831 | 3 | 1 |
| ARHGAP19 | 3.759493671 | 0.239799831 | 3 | 1 |
| MMP8 | 3.759493671 | 0.239799831 | 3 | 1 |
| NPEPL1 | 3.759493671 | 0.239799831 | 3 | 1 |
| BPIFB4 | 3.759493671 | 0.239799831 | 3 | 1 |
| XPO6 | 3.759493671 | 0.239799831 | 3 | 1 |
| LZTR1 | 3.759493671 | 0.239799831 | 3 | 1 |
| LRRC4 | 3.759493671 | 0.239799831 | 3 | 1 |
| FAM129C | 3.759493671 | 0.239799831 | 3 | 1 |
| WDR34 | 3.759493671 | 0.239799831 | 3 | 1 |
| NBPF7 | 3.759493671 | 0.239799831 | 3 | 1 |
| SMC4 | 3.759493671 | 0.239799831 | 3 | 1 |
| ANKRD18B | 3.759493671 | 0.239799831 | 3 | 1 |
| ARPC4-TTLL3 | 3.759493671 | 0.239799831 | 3 | 1 |
| FBXL16 | 3.759493671 | 0.239799831 | 3 | 1 |
| BTNL9 | 3.759493671 | 0.239799831 | 3 | 1 |
| SP100 | 3.759493671 | 0.239799831 | 3 | 1 |
| YJEFN3 | 3.759493671 | 0.239799831 | 3 | 1 |
| ZNF774 | 3.759493671 | 0.239799831 | 3 | 1 |
| NBEA | 3.759493671 | 0.239799831 | 3 | 1 |
| PNPT1 | 3.759493671 | 0.239799831 | 3 | 1 |
| AVPR1B | 3.759493671 | 0.239799831 | 3 | 1 |
| ZNF888 | 3.759493671 | 0.239799831 | 3 | 1 |
| NIM1K | 3.759493671 | 0.239799831 | 3 | 1 |
| ZNF83 | 3.759493671 | 0.239799831 | 3 | 1 |
| DMAC2 | 3.759493671 | 0.239799831 | 3 | 1 |
| ZNF382 | 3.759493671 | 0.239799831 | 3 | 1 |
| FAM83E | 3.759493671 | 0.239799831 | 3 | 1 |
| AIFM3 | 3.759493671 | 0.239799831 | 3 | 1 |
| NFATC3 | 3.759493671 | 0.239799831 | 3 | 1 |
| FLT1 | 3.759493671 | 0.239799831 | 3 | 1 |
| SELENOS | 3.759493671 | 0.239799831 | 3 | 1 |
| OR2A25 | 3.759493671 | 0.239799831 | 3 | 1 |
| FAM184A | 3.759493671 | 0.239799831 | 3 | 1 |
| ARID4B | 3.759493671 | 0.239799831 | 3 | 1 |
| ARID2 | 3.759493671 | 0.239799831 | 3 | 1 |
| ZNF600 | 3.759493671 | 0.239799831 | 3 | 1 |
| C16orf71 | 3.759493671 | 0.239799831 | 3 | 1 |
| FAM129B | 3.759493671 | 0.239799831 | 3 | 1 |
| WDR33 | 3.759493671 | 0.239799831 | 3 | 1 |
| PGM1 | 3.759493671 | 0.239799831 | 3 | 1 |
| ADAMTSL5 | 3.759493671 | 0.239799831 | 3 | 1 |
| FBXL21 | 3.759493671 | 0.239799831 | 3 | 1 |
| AP1AR | 3.759493671 | 0.239799831 | 3 | 1 |
| WDR86 | 3.759493671 | 0.239799831 | 3 | 1 |
| FITM1 | 3.759493671 | 0.239799831 | 3 | 1 |
| ZNF236 | 3.759493671 | 0.239799831 | 3 | 1 |
| ADH6 | 3.759493671 | 0.239799831 | 3 | 1 |
| FAM86B1 | 3.759493671 | 0.239799831 | 3 | 1 |
| BOC | 3.759493671 | 0.239799831 | 3 | 1 |
| FILIP1L | 3.759493671 | 0.239799831 | 3 | 1 |
| PCDHB12 | 3.759493671 | 0.239799831 | 3 | 1 |
| ADCY8 | 3.759493671 | 0.239799831 | 3 | 1 |
| C1orf159 | 3.759493671 | 0.239799831 | 3 | 1 |
| PEX1 | 3.759493671 | 0.239799831 | 3 | 1 |
| AMOTL2 | 3.759493671 | 0.239799831 | 3 | 1 |
| OR5B12 | 3.759493671 | 0.239799831 | 3 | 1 |
| MCM3AP | 3.759493671 | 0.239799831 | 3 | 1 |
| MED12L | 3.759493671 | 0.239799831 | 3 | 1 |
| SON | 3.759493671 | 0.239799831 | 3 | 1 |
| FOXB2 | 3.759493671 | 0.239799831 | 3 | 1 |
| PLA1A | 3.759493671 | 0.239799831 | 3 | 1 |
| AP5Z1 | 3.759493671 | 0.239799831 | 3 | 1 |
| ZNF674 | 3.759493671 | 0.239799831 | 3 | 1 |
| WSCD1 | 3.759493671 | 0.239799831 | 3 | 1 |
| AMPD3 | 3.759493671 | 0.239799831 | 3 | 1 |
| MORN1 | 3.759493671 | 0.239799831 | 3 | 1 |
| ZNF217 | 3.759493671 | 0.239799831 | 3 | 1 |
| ASZ1 | 3.759493671 | 0.239799831 | 3 | 1 |
| SLC26A6 | 3.759493671 | 0.239799831 | 3 | 1 |
| MYBL2 | 3.759493671 | 0.239799831 | 3 | 1 |
| PIWIL3 | 3.759493671 | 0.239799831 | 3 | 1 |
| ZSCAN5A | 3.759493671 | 0.239799831 | 3 | 1 |
| SNRNP200 | 3.759493671 | 0.239799831 | 3 | 1 |
| MORC1 | 3.759493671 | 0.239799831 | 3 | 1 |
| BFSP1 | 3.759493671 | 0.239799831 | 3 | 1 |
| MPG | 3.759493671 | 0.239799831 | 3 | 1 |
| PCDH12 | 3.759493671 | 0.239799831 | 3 | 1 |
| ABCB6 | 3.759493671 | 0.239799831 | 3 | 1 |
| SNPH | 3.759493671 | 0.239799831 | 3 | 1 |
| FBXW10 | 3.759493671 | 0.239799831 | 3 | 1 |
| PLAC8L1 | 3.759493671 | 0.239799831 | 3 | 1 |
| SLC39A10 | 3.759493671 | 0.239799831 | 3 | 1 |
| ANXA13 | 3.759493671 | 0.239799831 | 3 | 1 |
| ZNF296 | 3.759493671 | 0.239799831 | 3 | 1 |
| ALS2 | 3.759493671 | 0.239799831 | 3 | 1 |
| NSUN5 | 3.759493671 | 0.239799831 | 3 | 1 |
| ACSBG2 | 3.759493671 | 0.239799831 | 3 | 1 |
| ZSWIM4 | 3.759493671 | 0.239799831 | 3 | 1 |
| FRMD4A | 3.759493671 | 0.239799831 | 3 | 1 |
| ASPRV1 | 3.759493671 | 0.239799831 | 3 | 1 |
| C10orf71 | 3.759493671 | 0.239799831 | 3 | 1 |
| SLC43A2 | 3.759493671 | 0.239799831 | 3 | 1 |
| SLC37A2 | 3.759493671 | 0.239799831 | 3 | 1 |
| PKNOX1 | 3.759493671 | 0.239799831 | 3 | 1 |
| MDN1 | 1.597222222 | 0.2432957 | 10 | 8 |
| BAHCC1 | 1.597222222 | 0.2432957 | 10 | 8 |
| CLEC18C | 1.283333333 | 0.246181077 | 42 | 45 |
| DCHS1 | 1.637964775 | 0.247773006 | 9 | 7 |
| EVPL | 1.637964775 | 0.247773006 | 9 | 7 |
| OR2T8 | 1.277777778 | 0.251344983 | 46 | 50 |
| ACE | 1.693693694 | 0.251765464 | 8 | 6 |
| COL28A1 | 1.693693694 | 0.251765464 | 8 | 6 |
| C6orf201 | 1.693693694 | 0.251765464 | 8 | 6 |
| ANK3 | 1.693693694 | 0.251765464 | 8 | 6 |
| CSMD2 | 1.693693694 | 0.251765464 | 8 | 6 |
| MUC12 | 3.375 | 0.252050765 | 81 | 96 |
| ASTN2 | 2.512820513 | 0.25269922 | 4 | 2 |
| RANBP17 | 2.512820513 | 0.25269922 | 4 | 2 |
| GALNT10 | 2.512820513 | 0.25269922 | 4 | 2 |
| HHIPL1 | 2.512820513 | 0.25269922 | 4 | 2 |
| NEFH | 2.512820513 | 0.25269922 | 4 | 2 |
| LYST | 2.512820513 | 0.25269922 | 4 | 2 |
| IGSF22 | 2.512820513 | 0.25269922 | 4 | 2 |
| LMF1 | 2.512820513 | 0.25269922 | 4 | 2 |
| HFM1 | 2.512820513 | 0.25269922 | 4 | 2 |
| ABRAXAS2 | 2.512820513 | 0.25269922 | 4 | 2 |
| SHROOM3 | 2.512820513 | 0.25269922 | 4 | 2 |
| C11orf80 | 2.512820513 | 0.25269922 | 4 | 2 |
| HIVEP2 | 2.512820513 | 0.25269922 | 4 | 2 |
| SPG7 | 2.512820513 | 0.25269922 | 4 | 2 |
| VPS13A | 2.512820513 | 0.25269922 | 4 | 2 |
| RAB11FIP4 | 2.512820513 | 0.25269922 | 4 | 2 |
| KIAA1324L | 2.512820513 | 0.25269922 | 4 | 2 |
| IFNLR1 | 2.512820513 | 0.25269922 | 4 | 2 |
| ALPK2 | 2.512820513 | 0.25269922 | 4 | 2 |
| GPRIN2 | 2.512820513 | 0.25269922 | 4 | 2 |
| GLI3 | 2.512820513 | 0.25269922 | 4 | 2 |
| ARFGEF2 | 2.512820513 | 0.25269922 | 4 | 2 |
| PTPN18 | 2.512820513 | 0.25269922 | 4 | 2 |
| LRP5L | 2.512820513 | 0.25269922 | 4 | 2 |
| CLASRP | 2.512820513 | 0.25269922 | 4 | 2 |
| MZF1 | 2.512820513 | 0.25269922 | 4 | 2 |
| NPC1L1 | 2.512820513 | 0.25269922 | 4 | 2 |
| ABCA6 | 2.512820513 | 0.25269922 | 4 | 2 |
| SLC4A1AP | 2.512820513 | 0.25269922 | 4 | 2 |
| LRCH4 | 2.512820513 | 0.25269922 | 4 | 2 |
| CHRD | 2.512820513 | 0.25269922 | 4 | 2 |
| CBX2 | 2.512820513 | 0.25269922 | 4 | 2 |
| ZNF208 | 2.512820513 | 0.25269922 | 4 | 2 |
| RBMY1A1 | 2.512820513 | 0.25269922 | 4 | 2 |
| TRPV1 | 2.512820513 | 0.25269922 | 4 | 2 |
| ST14 | 2.512820513 | 0.25269922 | 4 | 2 |
| BMP6 | 2.512820513 | 0.25269922 | 4 | 2 |
| CTNND2 | 2.512820513 | 0.25269922 | 4 | 2 |
| MYO19 | 2.512820513 | 0.25269922 | 4 | 2 |
| MYO10 | 2.512820513 | 0.25269922 | 4 | 2 |
| WFS1 | 2.512820513 | 0.25269922 | 4 | 2 |
| TSPEAR | 2.512820513 | 0.25269922 | 4 | 2 |
| CCDC178 | 2.512820513 | 0.25269922 | 4 | 2 |
| TTBK1 | 2.512820513 | 0.25269922 | 4 | 2 |
| THSD7A | 2.512820513 | 0.25269922 | 4 | 2 |
| C20orf85 | 2.512820513 | 0.25269922 | 4 | 2 |
| A2M | 2.512820513 | 0.25269922 | 4 | 2 |
| BCAR1 | 2.512820513 | 0.25269922 | 4 | 2 |
| CD276 | 2.512820513 | 0.25269922 | 4 | 2 |
| GSN | 2.512820513 | 0.25269922 | 4 | 2 |
| ZSCAN29 | 2.512820513 | 0.25269922 | 4 | 2 |
| FREM3 | 2.512820513 | 0.25269922 | 4 | 2 |
| CYFIP1 | 2.512820513 | 0.25269922 | 4 | 2 |
| PDE9A | 2.512820513 | 0.25269922 | 4 | 2 |
| DGKQ | 2.512820513 | 0.25269922 | 4 | 2 |
| EGLN1 | 2.512820513 | 0.25269922 | 4 | 2 |
| PHKA2 | 2.512820513 | 0.25269922 | 4 | 2 |
| ID4 | 2.512820513 | 0.25269922 | 4 | 2 |
| ZFR2 | 2.512820513 | 0.25269922 | 4 | 2 |
| CRB2 | 2.512820513 | 0.25269922 | 4 | 2 |
| NUP153 | 2.512820513 | 0.25269922 | 4 | 2 |
| CARD14 | 2.512820513 | 0.25269922 | 4 | 2 |
| KLHL33 | 2.512820513 | 0.25269922 | 4 | 2 |
| MFHAS1 | 2.512820513 | 0.25269922 | 4 | 2 |
| TNK2 | 2.512820513 | 0.25269922 | 4 | 2 |
| WDR19 | 2.512820513 | 0.25269922 | 4 | 2 |
| GPR161 | 2.512820513 | 0.25269922 | 4 | 2 |
| NUTM2G | 2.512820513 | 0.25269922 | 4 | 2 |
| FGD3 | 2.512820513 | 0.25269922 | 4 | 2 |
| CFAP44 | 2.512820513 | 0.25269922 | 4 | 2 |
| PLCG2 | 2.512820513 | 0.25269922 | 4 | 2 |
| RHPN2 | 2.512820513 | 0.25269922 | 4 | 2 |
| KATNAL2 | 2.512820513 | 0.25269922 | 4 | 2 |
| IMMT | 2.512820513 | 0.25269922 | 4 | 2 |
| EHMT1 | 2.512820513 | 0.25269922 | 4 | 2 |
| CGNL1 | 2.512820513 | 0.25269922 | 4 | 2 |
| MAP10 | 2.512820513 | 0.25269922 | 4 | 2 |
| KIAA1211L | 2.512820513 | 0.25269922 | 4 | 2 |
| ADAMTS9 | 1.773333333 | 0.25499698 | 7 | 5 |
| AGAP7P | 1.773333333 | 0.25499698 | 7 | 5 |
| MTCL1 | 1.773333333 | 0.25499698 | 7 | 5 |
| TNS1 | 1.773333333 | 0.25499698 | 7 | 5 |
| RELN | 1.773333333 | 0.25499698 | 7 | 5 |
| ANKRD24 | 1.773333333 | 0.25499698 | 7 | 5 |
| PKDREJ | 2.0995671 | 0.256851121 | 5 | 3 |
| PLPPR3 | 2.0995671 | 0.256851121 | 5 | 3 |
| ESPNL | 2.0995671 | 0.256851121 | 5 | 3 |
| USP42 | 2.0995671 | 0.256851121 | 5 | 3 |
| BMP8B | 2.0995671 | 0.256851121 | 5 | 3 |
| C2orf71 | 2.0995671 | 0.256851121 | 5 | 3 |
| TRANK1 | 2.0995671 | 0.256851121 | 5 | 3 |
| SETD1B | 2.0995671 | 0.256851121 | 5 | 3 |
| EFHC1 | 2.0995671 | 0.256851121 | 5 | 3 |
| ARAP2 | 2.0995671 | 0.256851121 | 5 | 3 |
| EIF6 | 2.0995671 | 0.256851121 | 5 | 3 |
| ANTXRL | 2.0995671 | 0.256851121 | 5 | 3 |
| TULP4 | 2.0995671 | 0.256851121 | 5 | 3 |
| PCSK4 | 2.0995671 | 0.256851121 | 5 | 3 |
| ZNF628 | 2.0995671 | 0.256851121 | 5 | 3 |
| DLEC1 | 2.0995671 | 0.256851121 | 5 | 3 |
| PIDD1 | 2.0995671 | 0.256851121 | 5 | 3 |
| TRRAP | 2.0995671 | 0.256851121 | 5 | 3 |
| TMEM132C | 2.0995671 | 0.256851121 | 5 | 3 |
| ZP2 | 2.0995671 | 0.256851121 | 5 | 3 |
| PCM1 | 2.0995671 | 0.256851121 | 5 | 3 |
| KMT2A | 2.0995671 | 0.256851121 | 5 | 3 |
| IGKV1D-13 | 2.0995671 | 0.256851121 | 5 | 3 |
| ICE1 | 2.0995671 | 0.256851121 | 5 | 3 |
| GCC2 | 2.0995671 | 0.256851121 | 5 | 3 |
| PCDH15 | 2.0995671 | 0.256851121 | 5 | 3 |
| CAPN15 | 2.0995671 | 0.256851121 | 5 | 3 |
| RBM20 | 2.0995671 | 0.256851121 | 5 | 3 |
| CHAF1A | 2.0995671 | 0.256851121 | 5 | 3 |
| QRICH2 | 2.0995671 | 0.256851121 | 5 | 3 |
| AKAP6 | 2.0995671 | 0.256851121 | 5 | 3 |
| MTUS1 | 1.894736842 | 0.256990263 | 6 | 4 |
| CPZ | 1.894736842 | 0.256990263 | 6 | 4 |
| CHD6 | 1.894736842 | 0.256990263 | 6 | 4 |
| PTPRR | 1.894736842 | 0.256990263 | 6 | 4 |
| KMT2B | 1.894736842 | 0.256990263 | 6 | 4 |
| WDR90 | 1.894736842 | 0.256990263 | 6 | 4 |
| ROS1 | 1.894736842 | 0.256990263 | 6 | 4 |
| CFTR | 1.894736842 | 0.256990263 | 6 | 4 |
| GTF2IRD2B | 1.894736842 | 0.256990263 | 6 | 4 |
| RADIL | 1.894736842 | 0.256990263 | 6 | 4 |
| MPDZ | 1.894736842 | 0.256990263 | 6 | 4 |
| DACT2 | 1.894736842 | 0.256990263 | 6 | 4 |
| NOP14 | 1.894736842 | 0.256990263 | 6 | 4 |
| EML6 | 1.894736842 | 0.256990263 | 6 | 4 |
| CEP295 | 1.894736842 | 0.256990263 | 6 | 4 |
| NBPF12 | 1.982078853 | 0.258540356 | 79 | 93 |
| GGT2 | 1.982078853 | 0.258540356 | 79 | 93 |
| POTEG | 1.310344828 | 0.262829146 | 24 | 24 |
| NOMO2 | 1.373076923 | 0.263431467 | 17 | 16 |
| ANKRD30A | 1.305084746 | 0.271014571 | 23 | 23 |
| GOLGA6L10 | 1.373737374 | 0.271087925 | 16 | 15 |
| RIMBP3B | 1.264957265 | 0.277982302 | 56 | 63 |
| FAM136A | 1.23956044 | 0.285522851 | 47 | 52 |
| NPIPA3 | 1.233333333 | 0.292723819 | 37 | 40 |
| MUC17 | 1.233333333 | 0.292723819 | 37 | 40 |
| DCHS2 | inf | 0.300528201 | 82 | 98 |
| GOLGA8K | inf | 0.300528201 | 82 | 98 |
| LOC100131608 | inf | 0.300528201 | 82 | 98 |
| SPATA31A3 | inf | 0.300528201 | 82 | 98 |
| RP1L1 | 1.387012987 | 0.303973183 | 12 | 11 |
| PRAMEF5 | 1.371428571 | 0.304254301 | 72 | 84 |
| GP6 | 1.285714286 | 0.305863582 | 19 | 19 |
| LOC101060183 | 2.105263158 | 0.311526315 | 80 | 95 |
| KIAA1841 | 1.394366197 | 0.312905953 | 11 | 10 |
| CTAGE4 | 1.394366197 | 0.312905953 | 11 | 10 |
| SIMC1 | 1.24137931 | 0.315756996 | 24 | 25 |
| CMYA5 | 1.404320988 | 0.322206326 | 10 | 9 |
| DMBT1 | 1.404320988 | 0.322206326 | 10 | 9 |
| KRTAP2-2 | 1.404320988 | 0.322206326 | 10 | 9 |
| TPM2 | 1.523076923 | 0.328895804 | 77 | 91 |
| USP17L2 | 1.417808219 | 0.331935033 | 9 | 8 |
| CELSR1 | 1.417808219 | 0.331935033 | 9 | 8 |
| LOXHD1 | 1.417808219 | 0.331935033 | 9 | 8 |
| PLEC | 1.272727273 | 0.334891744 | 16 | 16 |
| SKA3 | 1.188571429 | 0.342166694 | 32 | 35 |
| CNTNAP4 | 1.436293436 | 0.342170955 | 8 | 7 |
| VPS13B | 1.436293436 | 0.342170955 | 8 | 7 |
| PKHD1 | 1.436293436 | 0.342170955 | 8 | 7 |
| PCLO | 1.436293436 | 0.342170955 | 8 | 7 |
| CELA1 | 1.280487805 | 0.343237464 | 70 | 82 |
| OBSCN | 1.268656716 | 0.345305559 | 15 | 15 |
| LOC100288142 | 1.268656716 | 0.345305559 | 15 | 15 |
| SLC25A5 | 1.190298507 | 0.352905953 | 58 | 67 |
| ITGB4 | 1.462222222 | 0.353019614 | 7 | 6 |
| SPATA31A6 | 1.462222222 | 0.353019614 | 7 | 6 |
| FAM153B | 1.462222222 | 0.353019614 | 7 | 6 |
| DNAH6 | 1.462222222 | 0.353019614 | 7 | 6 |
| RIF1 | 1.462222222 | 0.353019614 | 7 | 6 |
| RBL1 | 1.264705882 | 0.356174754 | 14 | 14 |
| PCDHA11 | 1.5 | 0.364627557 | 6 | 5 |
| LRBA | 1.5 | 0.364627557 | 6 | 5 |
| SPTB | 1.5 | 0.364627557 | 6 | 5 |
| NLRP8 | 1.5 | 0.364627557 | 6 | 5 |
| SOX1 | 1.5 | 0.364627557 | 6 | 5 |
| GOLGA6A | 1.5 | 0.364627557 | 6 | 5 |
| RTEL1 | 1.5 | 0.364627557 | 6 | 5 |
| RIPOR2 | 1.5 | 0.364627557 | 6 | 5 |
| ACACB | 1.5 | 0.364627557 | 6 | 5 |
| HMMR | 1.5 | 0.364627557 | 6 | 5 |
| ASPM | 1.5 | 0.364627557 | 6 | 5 |
| C5orf42 | 1.5 | 0.364627557 | 6 | 5 |
| LOC101060581 | 1.207430341 | 0.365604261 | 65 | 76 |
| CTAGE5 | 1.186452046 | 0.366775663 | 61 | 71 |
| ELOA3B | 1.260869565 | 0.367569256 | 13 | 13 |
| USP17L4 | 1.558441558 | 0.37720854 | 5 | 4 |
| ABCA3 | 1.558441558 | 0.37720854 | 5 | 4 |
| ATXN7 | 1.558441558 | 0.37720854 | 5 | 4 |
| PDIA2 | 1.558441558 | 0.37720854 | 5 | 4 |
| HERC1 | 1.558441558 | 0.37720854 | 5 | 4 |
| TTC3 | 1.558441558 | 0.37720854 | 5 | 4 |
| RNF123 | 1.558441558 | 0.37720854 | 5 | 4 |
| ADGB | 1.558441558 | 0.37720854 | 5 | 4 |
| NOTCH3 | 1.558441558 | 0.37720854 | 5 | 4 |
| PRUNE2 | 1.558441558 | 0.37720854 | 5 | 4 |
| TMEM131L | 1.558441558 | 0.37720854 | 5 | 4 |
| SVEP1 | 1.558441558 | 0.37720854 | 5 | 4 |
| PRB2 | 1.558441558 | 0.37720854 | 5 | 4 |
| IGHV3-66 | 1.558441558 | 0.37720854 | 5 | 4 |
| ZNF251 | 1.558441558 | 0.37720854 | 5 | 4 |
| LAMB1 | 1.558441558 | 0.37720854 | 5 | 4 |
| FER1L6 | 1.558441558 | 0.37720854 | 5 | 4 |
| TUBGCP6 | 1.558441558 | 0.37720854 | 5 | 4 |
| EEF2KMT | 1.558441558 | 0.37720854 | 5 | 4 |
| NYNRIN | 1.558441558 | 0.37720854 | 5 | 4 |
| DPP3 | 1.558441558 | 0.37720854 | 5 | 4 |
| TRPM4 | 1.558441558 | 0.37720854 | 5 | 4 |
| SPTA1 | 1.558441558 | 0.37720854 | 5 | 4 |
| MEX3D | 1.558441558 | 0.37720854 | 5 | 4 |
| CNTNAP3B | 1.143915344 | 0.38304718 | 47 | 54 |
| BOLA2 | 1.154121864 | 0.38454796 | 28 | 31 |
| CTAGE8 | 2.505154639 | 0.3884854 | 81 | 97 |
| DHX38 | 1.658119658 | 0.391095934 | 4 | 3 |
| SETD1A | 1.658119658 | 0.391095934 | 4 | 3 |
| CTAGE1 | 1.658119658 | 0.391095934 | 4 | 3 |
| NOP2 | 1.658119658 | 0.391095934 | 4 | 3 |
| RET | 1.658119658 | 0.391095934 | 4 | 3 |
| PITRM1 | 1.658119658 | 0.391095934 | 4 | 3 |
| SFT2D3 | 1.658119658 | 0.391095934 | 4 | 3 |
| MYH6 | 1.658119658 | 0.391095934 | 4 | 3 |
| CEP162 | 1.658119658 | 0.391095934 | 4 | 3 |
| FBXO41 | 1.658119658 | 0.391095934 | 4 | 3 |
| RFX2 | 1.658119658 | 0.391095934 | 4 | 3 |
| KIAA1456 | 1.658119658 | 0.391095934 | 4 | 3 |
| DZIP1 | 1.658119658 | 0.391095934 | 4 | 3 |
| MYH4 | 1.658119658 | 0.391095934 | 4 | 3 |
| DHX37 | 1.658119658 | 0.391095934 | 4 | 3 |
| THSD7B | 1.658119658 | 0.391095934 | 4 | 3 |
| SLC25A23 | 1.658119658 | 0.391095934 | 4 | 3 |
| PGAP3 | 1.658119658 | 0.391095934 | 4 | 3 |
| AGRN | 1.658119658 | 0.391095934 | 4 | 3 |
| PCDHB10 | 1.658119658 | 0.391095934 | 4 | 3 |
| COL9A1 | 1.658119658 | 0.391095934 | 4 | 3 |
| ANKZF1 | 1.658119658 | 0.391095934 | 4 | 3 |
| PIF1 | 1.658119658 | 0.391095934 | 4 | 3 |
| KNDC1 | 1.658119658 | 0.391095934 | 4 | 3 |
| MST1R | 1.658119658 | 0.391095934 | 4 | 3 |
| NAA16 | 1.658119658 | 0.391095934 | 4 | 3 |
| STK4 | 1.658119658 | 0.391095934 | 4 | 3 |
| RIMBP3C | 1.658119658 | 0.391095934 | 4 | 3 |
| BAZ2B | 1.658119658 | 0.391095934 | 4 | 3 |
| FGD5 | 1.658119658 | 0.391095934 | 4 | 3 |
| RBBP8NL | 1.658119658 | 0.391095934 | 4 | 3 |
| CHTF18 | 1.658119658 | 0.391095934 | 4 | 3 |
| CNGB1 | 1.658119658 | 0.391095934 | 4 | 3 |
| FNIP1 | 1.658119658 | 0.391095934 | 4 | 3 |
| TAS1R2 | 1.658119658 | 0.391095934 | 4 | 3 |
| NBAS | 1.658119658 | 0.391095934 | 4 | 3 |
| CELSR3 | 1.658119658 | 0.391095934 | 4 | 3 |
| MYO1E | 1.658119658 | 0.391095934 | 4 | 3 |
| ALDH1L2 | 1.658119658 | 0.391095934 | 4 | 3 |
| MROH7 | 1.658119658 | 0.391095934 | 4 | 3 |
| QSER1 | 1.658119658 | 0.391095934 | 4 | 3 |
| NEURL4 | 1.658119658 | 0.391095934 | 4 | 3 |
| ANGEL2 | 1.658119658 | 0.391095934 | 4 | 3 |
| IL17RA | 1.658119658 | 0.391095934 | 4 | 3 |
| AMPH | 1.658119658 | 0.391095934 | 4 | 3 |
| GOLGA3 | 1.658119658 | 0.391095934 | 4 | 3 |
| OCA2 | 1.658119658 | 0.391095934 | 4 | 3 |
| NRXN3 | 1.658119658 | 0.391095934 | 4 | 3 |
| ABCA13 | 1.658119658 | 0.391095934 | 4 | 3 |
| LIMCH1 | 1.658119658 | 0.391095934 | 4 | 3 |
| PLXNA2 | 1.658119658 | 0.391095934 | 4 | 3 |
| SLC27A3 | 1.658119658 | 0.391095934 | 4 | 3 |
| CACNA1I | 1.658119658 | 0.391095934 | 4 | 3 |
| USP17L3 | 1.658119658 | 0.391095934 | 4 | 3 |
| UBR3 | 1.658119658 | 0.391095934 | 4 | 3 |
| ABCA9 | 1.658119658 | 0.391095934 | 4 | 3 |
| UNC13D | 1.658119658 | 0.391095934 | 4 | 3 |
| TSPOAP1 | 1.658119658 | 0.391095934 | 4 | 3 |
| LTK | 1.658119658 | 0.391095934 | 4 | 3 |
| TBC1D32 | 1.658119658 | 0.391095934 | 4 | 3 |
| FRY | 1.658119658 | 0.391095934 | 4 | 3 |
| ZNF99 | 1.658119658 | 0.391095934 | 4 | 3 |
| SAMD9 | 1.658119658 | 0.391095934 | 4 | 3 |
| NUTM1 | 1.658119658 | 0.391095934 | 4 | 3 |
| DDX54 | 1.658119658 | 0.391095934 | 4 | 3 |
| PKP3 | 1.658119658 | 0.391095934 | 4 | 3 |
| ZNF865 | 1.658119658 | 0.391095934 | 4 | 3 |
| GBGT1 | 1.658119658 | 0.391095934 | 4 | 3 |
| NOS2 | 1.658119658 | 0.391095934 | 4 | 3 |
| KRTAP5-5 | 1.658119658 | 0.391095934 | 4 | 3 |
| NUCKS1 | 1.253521127 | 0.392308347 | 11 | 11 |
| DNHD1 | 1.253521127 | 0.392308347 | 11 | 11 |
| OTOG | 1.253521127 | 0.392308347 | 11 | 11 |
| CHST15 | 1.25 | 0.405909683 | 10 | 10 |
| TRERF1 | 1.860759494 | 0.406862649 | 3 | 2 |
| HNF1A-AS1 | 1.860759494 | 0.406862649 | 3 | 2 |
| FLNA | 1.860759494 | 0.406862649 | 3 | 2 |
| PFKL | 1.860759494 | 0.406862649 | 3 | 2 |
| COMMD9 | 1.860759494 | 0.406862649 | 3 | 2 |
| OR4L1 | 1.860759494 | 0.406862649 | 3 | 2 |
| SLC25A47 | 1.860759494 | 0.406862649 | 3 | 2 |
| ILF3 | 1.860759494 | 0.406862649 | 3 | 2 |
| ZC3H18 | 1.860759494 | 0.406862649 | 3 | 2 |
| TAF5L | 1.860759494 | 0.406862649 | 3 | 2 |
| CAPN3 | 1.860759494 | 0.406862649 | 3 | 2 |
| MAG | 1.860759494 | 0.406862649 | 3 | 2 |
| CAPN13 | 1.860759494 | 0.406862649 | 3 | 2 |
| CLSTN2 | 1.860759494 | 0.406862649 | 3 | 2 |
| CCDC183 | 1.860759494 | 0.406862649 | 3 | 2 |
| RNF31 | 1.860759494 | 0.406862649 | 3 | 2 |
| SCARF2 | 1.860759494 | 0.406862649 | 3 | 2 |
| ANKRD6 | 1.860759494 | 0.406862649 | 3 | 2 |
| GAS6 | 1.860759494 | 0.406862649 | 3 | 2 |
| PLG | 1.860759494 | 0.406862649 | 3 | 2 |
| PCK2 | 1.860759494 | 0.406862649 | 3 | 2 |
| CPEB2 | 1.860759494 | 0.406862649 | 3 | 2 |
| ATP13A5 | 1.860759494 | 0.406862649 | 3 | 2 |
| HKR1 | 1.860759494 | 0.406862649 | 3 | 2 |
| CPAMD8 | 1.860759494 | 0.406862649 | 3 | 2 |
| ACAP3 | 1.860759494 | 0.406862649 | 3 | 2 |
| TM9SF4 | 1.860759494 | 0.406862649 | 3 | 2 |
| SLCO1B7 | 1.860759494 | 0.406862649 | 3 | 2 |
| ADGRA3 | 1.860759494 | 0.406862649 | 3 | 2 |
| PCDHGA5 | 1.860759494 | 0.406862649 | 3 | 2 |
| ARHGAP27 | 1.860759494 | 0.406862649 | 3 | 2 |
| EVC2 | 1.860759494 | 0.406862649 | 3 | 2 |
| CALHM4 | 1.860759494 | 0.406862649 | 3 | 2 |
| RNPEPL1 | 1.860759494 | 0.406862649 | 3 | 2 |
| HJURP | 1.860759494 | 0.406862649 | 3 | 2 |
| ANKS6 | 1.860759494 | 0.406862649 | 3 | 2 |
| ZBP1 | 1.860759494 | 0.406862649 | 3 | 2 |
| COX4I2 | 1.860759494 | 0.406862649 | 3 | 2 |
| ADGRF4 | 1.860759494 | 0.406862649 | 3 | 2 |
| KIF2C | 1.860759494 | 0.406862649 | 3 | 2 |
| SETBP1 | 1.860759494 | 0.406862649 | 3 | 2 |
| PLEKHN1 | 1.860759494 | 0.406862649 | 3 | 2 |
| LCOR | 1.860759494 | 0.406862649 | 3 | 2 |
| TMC3 | 1.860759494 | 0.406862649 | 3 | 2 |
| IMPDH1 | 1.860759494 | 0.406862649 | 3 | 2 |
| SLC9C1 | 1.860759494 | 0.406862649 | 3 | 2 |
| SI | 1.860759494 | 0.406862649 | 3 | 2 |
| DPCR1 | 1.860759494 | 0.406862649 | 3 | 2 |
| OR6X1 | 1.860759494 | 0.406862649 | 3 | 2 |
| TNK1 | 1.860759494 | 0.406862649 | 3 | 2 |
| PPP1R3A | 1.860759494 | 0.406862649 | 3 | 2 |
| ADPGK | 1.860759494 | 0.406862649 | 3 | 2 |
| ALS2CL | 1.860759494 | 0.406862649 | 3 | 2 |
| MSH2 | 1.860759494 | 0.406862649 | 3 | 2 |
| SPINK5 | 1.860759494 | 0.406862649 | 3 | 2 |
| VPS41 | 1.860759494 | 0.406862649 | 3 | 2 |
| PNLIPRP3 | 1.860759494 | 0.406862649 | 3 | 2 |
| POLD1 | 1.860759494 | 0.406862649 | 3 | 2 |
| SPDYE2 | 1.860759494 | 0.406862649 | 3 | 2 |
| MPRIP | 1.860759494 | 0.406862649 | 3 | 2 |
| TMEM191C | 1.860759494 | 0.406862649 | 3 | 2 |
| VPS33B | 1.860759494 | 0.406862649 | 3 | 2 |
| PADI6 | 1.860759494 | 0.406862649 | 3 | 2 |
| KCNH2 | 1.860759494 | 0.406862649 | 3 | 2 |
| ACKR2 | 1.860759494 | 0.406862649 | 3 | 2 |
| F12 | 1.860759494 | 0.406862649 | 3 | 2 |
| MRGPRX3 | 1.860759494 | 0.406862649 | 3 | 2 |
| ZFYVE19 | 1.860759494 | 0.406862649 | 3 | 2 |
| PPRC1 | 1.860759494 | 0.406862649 | 3 | 2 |
| C14orf166 | 1.860759494 | 0.406862649 | 3 | 2 |
| CNTNAP1 | 1.860759494 | 0.406862649 | 3 | 2 |
| ZFPM2 | 1.860759494 | 0.406862649 | 3 | 2 |
| LRRN1 | 1.860759494 | 0.406862649 | 3 | 2 |
| TAF1L | 1.860759494 | 0.406862649 | 3 | 2 |
| ANKRD12 | 1.860759494 | 0.406862649 | 3 | 2 |
| GLDC | 1.860759494 | 0.406862649 | 3 | 2 |
| LRRIQ3 | 1.860759494 | 0.406862649 | 3 | 2 |
| ZFHX4 | 1.860759494 | 0.406862649 | 3 | 2 |
| HECW1 | 1.860759494 | 0.406862649 | 3 | 2 |
| FAM193A | 1.860759494 | 0.406862649 | 3 | 2 |
| EN2 | 1.860759494 | 0.406862649 | 3 | 2 |
| UBR5 | 1.860759494 | 0.406862649 | 3 | 2 |
| CDK5RAP1 | 1.860759494 | 0.406862649 | 3 | 2 |
| CES1 | 1.860759494 | 0.406862649 | 3 | 2 |
| TMEM200C | 1.860759494 | 0.406862649 | 3 | 2 |
| C16orf96 | 1.860759494 | 0.406862649 | 3 | 2 |
| UNC5CL | 1.860759494 | 0.406862649 | 3 | 2 |
| CDH19 | 1.860759494 | 0.406862649 | 3 | 2 |
| FILIP1 | 1.860759494 | 0.406862649 | 3 | 2 |
| YME1L1 | 1.860759494 | 0.406862649 | 3 | 2 |
| SMG1 | 1.860759494 | 0.406862649 | 3 | 2 |
| LILRB1 | 1.860759494 | 0.406862649 | 3 | 2 |
| CADPS | 1.860759494 | 0.406862649 | 3 | 2 |
| COL2A1 | 1.860759494 | 0.406862649 | 3 | 2 |
| PREX2 | 1.860759494 | 0.406862649 | 3 | 2 |
| EML1 | 1.860759494 | 0.406862649 | 3 | 2 |
| EYA4 | 1.860759494 | 0.406862649 | 3 | 2 |
| PMF1 | 1.860759494 | 0.406862649 | 3 | 2 |
| COL21A1 | 1.860759494 | 0.406862649 | 3 | 2 |
| ACRBP | 1.860759494 | 0.406862649 | 3 | 2 |
| NDST2 | 1.860759494 | 0.406862649 | 3 | 2 |
| CCDC157 | 1.860759494 | 0.406862649 | 3 | 2 |
| STK32B | 1.860759494 | 0.406862649 | 3 | 2 |
| MSR1 | 1.860759494 | 0.406862649 | 3 | 2 |
| SMG6 | 1.860759494 | 0.406862649 | 3 | 2 |
| CEP72 | 1.860759494 | 0.406862649 | 3 | 2 |
| CEP63 | 1.860759494 | 0.406862649 | 3 | 2 |
| MSH6 | 1.860759494 | 0.406862649 | 3 | 2 |
| KIAA0355 | 1.860759494 | 0.406862649 | 3 | 2 |
| NSMAF | 1.860759494 | 0.406862649 | 3 | 2 |
| SRRM5 | 1.860759494 | 0.406862649 | 3 | 2 |
| HOOK3 | 1.860759494 | 0.406862649 | 3 | 2 |
| RTN4IP1 | 1.860759494 | 0.406862649 | 3 | 2 |
| PPFIBP2 | 1.860759494 | 0.406862649 | 3 | 2 |
| TROAP | 1.860759494 | 0.406862649 | 3 | 2 |
| ITFG1 | 1.860759494 | 0.406862649 | 3 | 2 |
| FARP2 | 1.860759494 | 0.406862649 | 3 | 2 |
| ITIH1 | 1.860759494 | 0.406862649 | 3 | 2 |
| TTI1 | 1.860759494 | 0.406862649 | 3 | 2 |
| LOC402160 | 1.860759494 | 0.406862649 | 3 | 2 |
| DDX25 | 1.860759494 | 0.406862649 | 3 | 2 |
| PRR21 | 1.860759494 | 0.406862649 | 3 | 2 |
| MCOLN1 | 1.860759494 | 0.406862649 | 3 | 2 |
| HPS4 | 1.860759494 | 0.406862649 | 3 | 2 |
| MYO3A | 1.860759494 | 0.406862649 | 3 | 2 |
| MAP3K15 | 1.860759494 | 0.406862649 | 3 | 2 |
| CLEC16A | 1.860759494 | 0.406862649 | 3 | 2 |
| ZSWIM8 | 1.860759494 | 0.406862649 | 3 | 2 |
| WWC1 | 1.860759494 | 0.406862649 | 3 | 2 |
| ITPRIPL1 | 1.860759494 | 0.406862649 | 3 | 2 |
| ELOA2 | 1.860759494 | 0.406862649 | 3 | 2 |
| SGCE | 1.860759494 | 0.406862649 | 3 | 2 |
| KTN1 | 1.860759494 | 0.406862649 | 3 | 2 |
| PKP4 | 1.860759494 | 0.406862649 | 3 | 2 |
| ELOA3 | 1.860759494 | 0.406862649 | 3 | 2 |
| PCDHB13 | 1.860759494 | 0.406862649 | 3 | 2 |
| DLAT | 1.860759494 | 0.406862649 | 3 | 2 |
| TTC22 | 1.860759494 | 0.406862649 | 3 | 2 |
| CATSPERB | 1.860759494 | 0.406862649 | 3 | 2 |
| CCDC66 | 1.860759494 | 0.406862649 | 3 | 2 |
| ENPP7 | 1.860759494 | 0.406862649 | 3 | 2 |
| NPAP1 | 1.860759494 | 0.406862649 | 3 | 2 |
| MED13 | 1.860759494 | 0.406862649 | 3 | 2 |
| PTK7 | 1.860759494 | 0.406862649 | 3 | 2 |
| CBWD3 | 1.860759494 | 0.406862649 | 3 | 2 |
| FANCI | 1.860759494 | 0.406862649 | 3 | 2 |
| ITGA8 | 1.860759494 | 0.406862649 | 3 | 2 |
| THBS4 | 1.860759494 | 0.406862649 | 3 | 2 |
| GPR20 | 1.860759494 | 0.406862649 | 3 | 2 |
| NLRC5 | 1.860759494 | 0.406862649 | 3 | 2 |
| ZNF221 | 1.860759494 | 0.406862649 | 3 | 2 |
| NPC1 | 1.860759494 | 0.406862649 | 3 | 2 |
| ITGAE | 1.860759494 | 0.406862649 | 3 | 2 |
| GCN1 | 1.860759494 | 0.406862649 | 3 | 2 |
| ITGAX | 1.860759494 | 0.406862649 | 3 | 2 |
| LONP1 | 1.860759494 | 0.406862649 | 3 | 2 |
| EXO1 | 1.860759494 | 0.406862649 | 3 | 2 |
| MELK | 1.860759494 | 0.406862649 | 3 | 2 |
| PCDHAC2 | 1.860759494 | 0.406862649 | 3 | 2 |
| SKIV2L | 1.860759494 | 0.406862649 | 3 | 2 |
| SLC13A3 | 1.860759494 | 0.406862649 | 3 | 2 |
| USP39 | 1.860759494 | 0.406862649 | 3 | 2 |
| LRCH1 | 1.860759494 | 0.406862649 | 3 | 2 |
| PIK3R4 | 1.860759494 | 0.406862649 | 3 | 2 |
| RGSL1 | 1.860759494 | 0.406862649 | 3 | 2 |
| DOPEY1 | 1.860759494 | 0.406862649 | 3 | 2 |
| PCDHB5 | 1.860759494 | 0.406862649 | 3 | 2 |
| PHLPP1 | 1.860759494 | 0.406862649 | 3 | 2 |
| TLR2 | 1.860759494 | 0.406862649 | 3 | 2 |
| PKN3 | 1.860759494 | 0.406862649 | 3 | 2 |
| PCDHB8 | 1.860759494 | 0.406862649 | 3 | 2 |
| INPP5B | 1.860759494 | 0.406862649 | 3 | 2 |
| EPHX1 | 1.860759494 | 0.406862649 | 3 | 2 |
| RCL1 | 1.860759494 | 0.406862649 | 3 | 2 |
| PPM1F | 1.860759494 | 0.406862649 | 3 | 2 |
| AHDC1 | 1.860759494 | 0.406862649 | 3 | 2 |
| WDR11 | 1.860759494 | 0.406862649 | 3 | 2 |
| LOC643696 | 1.860759494 | 0.406862649 | 3 | 2 |
| PWP2 | 1.860759494 | 0.406862649 | 3 | 2 |
| SFRP4 | 1.860759494 | 0.406862649 | 3 | 2 |
| CTDP1 | 1.860759494 | 0.406862649 | 3 | 2 |
| DDX60L | 1.860759494 | 0.406862649 | 3 | 2 |
| LRP4 | 1.860759494 | 0.406862649 | 3 | 2 |
| EPHB1 | 1.860759494 | 0.406862649 | 3 | 2 |
| FAM13A | 1.860759494 | 0.406862649 | 3 | 2 |
| SLC25A18 | 1.860759494 | 0.406862649 | 3 | 2 |
| B9D1 | 1.860759494 | 0.406862649 | 3 | 2 |
| LCT | 1.860759494 | 0.406862649 | 3 | 2 |
| CD109 | 1.860759494 | 0.406862649 | 3 | 2 |
| TLR5 | 1.860759494 | 0.406862649 | 3 | 2 |
| APC | 1.860759494 | 0.406862649 | 3 | 2 |
| NHS | 1.860759494 | 0.406862649 | 3 | 2 |
| NOTCH2NL | 1.860759494 | 0.406862649 | 3 | 2 |
| CLCN2 | 1.860759494 | 0.406862649 | 3 | 2 |
| KRTAP5-7 | 1.860759494 | 0.406862649 | 3 | 2 |
| MUC20 | 1.117021277 | 0.414780391 | 35 | 40 |
| SLC6A4 | 1.1124871 | 0.41768851 | 44 | 51 |
| ARMCX4 | 1.246575342 | 0.420574366 | 9 | 9 |
| CSMD1 | 1.246575342 | 0.420574366 | 9 | 9 |
| FOXD4L6 | 1.339130435 | 0.421823132 | 77 | 92 |
| SMIM20 | 2.475 | 0.425657216 | 2 | 1 |
| DBF4B | 2.475 | 0.425657216 | 2 | 1 |
| NUF2 | 2.475 | 0.425657216 | 2 | 1 |
| NAT16 | 2.475 | 0.425657216 | 2 | 1 |
| SETDB2 | 2.475 | 0.425657216 | 2 | 1 |
| DNAJB7 | 2.475 | 0.425657216 | 2 | 1 |
| GYPA | 2.475 | 0.425657216 | 2 | 1 |
| ABL2 | 2.475 | 0.425657216 | 2 | 1 |
| TDRD10 | 2.475 | 0.425657216 | 2 | 1 |
| NUP85 | 2.475 | 0.425657216 | 2 | 1 |
| NAT14 | 2.475 | 0.425657216 | 2 | 1 |
| SNCAIP | 2.475 | 0.425657216 | 2 | 1 |
| IL1F10 | 2.475 | 0.425657216 | 2 | 1 |
| TBX21 | 2.475 | 0.425657216 | 2 | 1 |
| DCAF12 | 2.475 | 0.425657216 | 2 | 1 |
| CLCN6 | 2.475 | 0.425657216 | 2 | 1 |
| RIPK3 | 2.475 | 0.425657216 | 2 | 1 |
| APBB1 | 2.475 | 0.425657216 | 2 | 1 |
| CLCNKA | 2.475 | 0.425657216 | 2 | 1 |
| SEZ6L | 2.475 | 0.425657216 | 2 | 1 |
| RFWD3 | 2.475 | 0.425657216 | 2 | 1 |
| DDX55 | 2.475 | 0.425657216 | 2 | 1 |
| GTF3A | 2.475 | 0.425657216 | 2 | 1 |
| APBA2 | 2.475 | 0.425657216 | 2 | 1 |
| GTF3C1 | 2.475 | 0.425657216 | 2 | 1 |
| TMEM72 | 2.475 | 0.425657216 | 2 | 1 |
| ZNF383 | 2.475 | 0.425657216 | 2 | 1 |
| SCAF4 | 2.475 | 0.425657216 | 2 | 1 |
| PADI4 | 2.475 | 0.425657216 | 2 | 1 |
| RIN2 | 2.475 | 0.425657216 | 2 | 1 |
| IQGAP1 | 2.475 | 0.425657216 | 2 | 1 |
| FAM20C | 2.475 | 0.425657216 | 2 | 1 |
| ZNF385D | 2.475 | 0.425657216 | 2 | 1 |
| DNMT3A | 2.475 | 0.425657216 | 2 | 1 |
| ZNF394 | 2.475 | 0.425657216 | 2 | 1 |
| TBK1 | 2.475 | 0.425657216 | 2 | 1 |
| DDAH1 | 2.475 | 0.425657216 | 2 | 1 |
| DENND5A | 2.475 | 0.425657216 | 2 | 1 |
| GOLM1 | 2.475 | 0.425657216 | 2 | 1 |
| SLC5A6 | 2.475 | 0.425657216 | 2 | 1 |
| HAVCR2 | 2.475 | 0.425657216 | 2 | 1 |
| EXT3 | 2.475 | 0.425657216 | 2 | 1 |
| PARP14 | 2.475 | 0.425657216 | 2 | 1 |
| FCHO1 | 2.475 | 0.425657216 | 2 | 1 |
| EXPH5 | 2.475 | 0.425657216 | 2 | 1 |
| TBCE | 2.475 | 0.425657216 | 2 | 1 |
| ABCA5 | 2.475 | 0.425657216 | 2 | 1 |
| SEC14L5 | 2.475 | 0.425657216 | 2 | 1 |
| ANXA7 | 2.475 | 0.425657216 | 2 | 1 |
| TCF12 | 2.475 | 0.425657216 | 2 | 1 |
| CLEC4F | 2.475 | 0.425657216 | 2 | 1 |
| ZNF324B | 2.475 | 0.425657216 | 2 | 1 |
| FCRL1 | 2.475 | 0.425657216 | 2 | 1 |
| HAUS6 | 2.475 | 0.425657216 | 2 | 1 |
| ANO4 | 2.475 | 0.425657216 | 2 | 1 |
| INPP5J | 2.475 | 0.425657216 | 2 | 1 |
| SMYD1 | 2.475 | 0.425657216 | 2 | 1 |
| PAPD5 | 2.475 | 0.425657216 | 2 | 1 |
| SCN4A | 2.475 | 0.425657216 | 2 | 1 |
| OCSTAMP | 2.475 | 0.425657216 | 2 | 1 |
| OAZ1 | 2.475 | 0.425657216 | 2 | 1 |
| ANPEP | 2.475 | 0.425657216 | 2 | 1 |
| OR13D1 | 2.475 | 0.425657216 | 2 | 1 |
| PAQR6 | 2.475 | 0.425657216 | 2 | 1 |
| ZSWIM1 | 2.475 | 0.425657216 | 2 | 1 |
| TCF7 | 2.475 | 0.425657216 | 2 | 1 |
| ZSCAN4 | 2.475 | 0.425657216 | 2 | 1 |
| ZNF302 | 2.475 | 0.425657216 | 2 | 1 |
| SEC23IP | 2.475 | 0.425657216 | 2 | 1 |
| GYPB | 2.475 | 0.425657216 | 2 | 1 |
| OR1E2 | 2.475 | 0.425657216 | 2 | 1 |
| FAM43B | 2.475 | 0.425657216 | 2 | 1 |
| MYNN | 2.475 | 0.425657216 | 2 | 1 |
| IQCG | 2.475 | 0.425657216 | 2 | 1 |
| HDAC4 | 2.475 | 0.425657216 | 2 | 1 |
| SLC47A1 | 2.475 | 0.425657216 | 2 | 1 |
| SMOC2 | 2.475 | 0.425657216 | 2 | 1 |
| ANKRD44 | 2.475 | 0.425657216 | 2 | 1 |
| ZNRF4 | 2.475 | 0.425657216 | 2 | 1 |
| RFX8 | 2.475 | 0.425657216 | 2 | 1 |
| AASDHPPT | 2.475 | 0.425657216 | 2 | 1 |
| RILPL1 | 2.475 | 0.425657216 | 2 | 1 |
| OR11H6 | 2.475 | 0.425657216 | 2 | 1 |
| DDX31 | 2.475 | 0.425657216 | 2 | 1 |
| DDB1 | 2.475 | 0.425657216 | 2 | 1 |
| PAK2 | 2.475 | 0.425657216 | 2 | 1 |
| DCAF8L2 | 2.475 | 0.425657216 | 2 | 1 |
| GOLGA6L1 | 2.475 | 0.425657216 | 2 | 1 |
| SCARB1 | 2.475 | 0.425657216 | 2 | 1 |
| DDX28 | 2.475 | 0.425657216 | 2 | 1 |
| GZF1 | 2.475 | 0.425657216 | 2 | 1 |
| HCFC2 | 2.475 | 0.425657216 | 2 | 1 |
| ZNF35 | 2.475 | 0.425657216 | 2 | 1 |
| PASD1 | 2.475 | 0.425657216 | 2 | 1 |
| HCAR3 | 2.475 | 0.425657216 | 2 | 1 |
| PALD1 | 2.475 | 0.425657216 | 2 | 1 |
| PALLD | 2.475 | 0.425657216 | 2 | 1 |
| ZNF266 | 2.475 | 0.425657216 | 2 | 1 |
| PALM2 | 2.475 | 0.425657216 | 2 | 1 |
| ABHD15 | 2.475 | 0.425657216 | 2 | 1 |
| PALM3 | 2.475 | 0.425657216 | 2 | 1 |
| ABHD12B | 2.475 | 0.425657216 | 2 | 1 |
| NUDT5 | 2.475 | 0.425657216 | 2 | 1 |
| TMEM132A | 2.475 | 0.425657216 | 2 | 1 |
| HELB | 2.475 | 0.425657216 | 2 | 1 |
| RUFY3 | 2.475 | 0.425657216 | 2 | 1 |
| CRIM1 | 2.475 | 0.425657216 | 2 | 1 |
| GPT2 | 2.475 | 0.425657216 | 2 | 1 |
| FAM84B | 2.475 | 0.425657216 | 2 | 1 |
| SLITRK3 | 2.475 | 0.425657216 | 2 | 1 |
| FAM129A | 2.475 | 0.425657216 | 2 | 1 |
| AHCY | 2.475 | 0.425657216 | 2 | 1 |
| AHCTF1 | 2.475 | 0.425657216 | 2 | 1 |
| TGFBR2 | 2.475 | 0.425657216 | 2 | 1 |
| TJP3 | 2.475 | 0.425657216 | 2 | 1 |
| SEMA6C | 2.475 | 0.425657216 | 2 | 1 |
| TGFBR3 | 2.475 | 0.425657216 | 2 | 1 |
| RTN3 | 2.475 | 0.425657216 | 2 | 1 |
| HOMEZ | 2.475 | 0.425657216 | 2 | 1 |
| ADAMTS4 | 2.475 | 0.425657216 | 2 | 1 |
| NPHS2 | 2.475 | 0.425657216 | 2 | 1 |
| TINF2 | 2.475 | 0.425657216 | 2 | 1 |
| DHRS13 | 2.475 | 0.425657216 | 2 | 1 |
| NPHP1 | 2.475 | 0.425657216 | 2 | 1 |
| NKTR | 2.475 | 0.425657216 | 2 | 1 |
| FAM83H | 2.475 | 0.425657216 | 2 | 1 |
| OR51A4 | 2.475 | 0.425657216 | 2 | 1 |
| CFAP47 | 2.475 | 0.425657216 | 2 | 1 |
| ADGRD1 | 2.475 | 0.425657216 | 2 | 1 |
| OR6C4 | 2.475 | 0.425657216 | 2 | 1 |
| ZNF780A | 2.475 | 0.425657216 | 2 | 1 |
| AKAP8 | 2.475 | 0.425657216 | 2 | 1 |
| HMG20A | 2.475 | 0.425657216 | 2 | 1 |
| ZNF574 | 2.475 | 0.425657216 | 2 | 1 |
| SEMA3A | 2.475 | 0.425657216 | 2 | 1 |
| ZNF772 | 2.475 | 0.425657216 | 2 | 1 |
| TLR4 | 2.475 | 0.425657216 | 2 | 1 |
| TIMM44 | 2.475 | 0.425657216 | 2 | 1 |
| NPRL2 | 2.475 | 0.425657216 | 2 | 1 |
| FAM161A | 2.475 | 0.425657216 | 2 | 1 |
| NPRL2 | 2.475 | 0.425657216 | 2 | 1 |
| FAM83C | 2.475 | 0.425657216 | 2 | 1 |
| RPH3A | 2.475 | 0.425657216 | 2 | 1 |
| ICAM1 | 2.475 | 0.425657216 | 2 | 1 |
| IBA57 | 2.475 | 0.425657216 | 2 | 1 |
| TLK1 | 2.475 | 0.425657216 | 2 | 1 |
| TLE6 | 2.475 | 0.425657216 | 2 | 1 |
| TLE3 | 2.475 | 0.425657216 | 2 | 1 |
| ZNF592 | 2.475 | 0.425657216 | 2 | 1 |
| OR51I1 | 2.475 | 0.425657216 | 2 | 1 |
| CRTC2 | 2.475 | 0.425657216 | 2 | 1 |
| CUL7 | 2.475 | 0.425657216 | 2 | 1 |
| CTNND1 | 2.475 | 0.425657216 | 2 | 1 |
| CSNK1D | 2.475 | 0.425657216 | 2 | 1 |
| ZNF718 | 2.475 | 0.425657216 | 2 | 1 |
| NMU | 2.475 | 0.425657216 | 2 | 1 |
| SERINC4 | 2.475 | 0.425657216 | 2 | 1 |
| TICAM1 | 2.475 | 0.425657216 | 2 | 1 |
| AFF2 | 2.475 | 0.425657216 | 2 | 1 |
| SMAD6 | 2.475 | 0.425657216 | 2 | 1 |
| THEGL | 2.475 | 0.425657216 | 2 | 1 |
| RRH | 2.475 | 0.425657216 | 2 | 1 |
| THTPA | 2.475 | 0.425657216 | 2 | 1 |
| NOMO1 | 2.475 | 0.425657216 | 2 | 1 |
| ZNF716 | 2.475 | 0.425657216 | 2 | 1 |
| HS1BP3 | 2.475 | 0.425657216 | 2 | 1 |
| RPUSD3 | 2.475 | 0.425657216 | 2 | 1 |
| GPR75 | 2.475 | 0.425657216 | 2 | 1 |
| ZNF708 | 2.475 | 0.425657216 | 2 | 1 |
| THNSL2 | 2.475 | 0.425657216 | 2 | 1 |
| ZNF701 | 2.475 | 0.425657216 | 2 | 1 |
| ADRA2B | 2.475 | 0.425657216 | 2 | 1 |
| NOL9 | 2.475 | 0.425657216 | 2 | 1 |
| CYP21A2 | 2.475 | 0.425657216 | 2 | 1 |
| HSPA2 | 2.475 | 0.425657216 | 2 | 1 |
| SERPINA12 | 2.475 | 0.425657216 | 2 | 1 |
| NLRC4 | 2.475 | 0.425657216 | 2 | 1 |
| OR5D16 | 2.475 | 0.425657216 | 2 | 1 |
| NLRP13 | 2.475 | 0.425657216 | 2 | 1 |
| HOXB1 | 2.475 | 0.425657216 | 2 | 1 |
| OR5B3 | 2.475 | 0.425657216 | 2 | 1 |
| ZNF749 | 2.475 | 0.425657216 | 2 | 1 |
| HOXB4 | 2.475 | 0.425657216 | 2 | 1 |
| THAP4 | 2.475 | 0.425657216 | 2 | 1 |
| DIS3L2 | 2.475 | 0.425657216 | 2 | 1 |
| RETREG1 | 2.475 | 0.425657216 | 2 | 1 |
| ZNF747 | 2.475 | 0.425657216 | 2 | 1 |
| DIS3 | 2.475 | 0.425657216 | 2 | 1 |
| TATDN3 | 2.475 | 0.425657216 | 2 | 1 |
| TAT | 2.475 | 0.425657216 | 2 | 1 |
| NMBR | 2.475 | 0.425657216 | 2 | 1 |
| TAS2R10 | 2.475 | 0.425657216 | 2 | 1 |
| NME8 | 2.475 | 0.425657216 | 2 | 1 |
| HPS1 | 2.475 | 0.425657216 | 2 | 1 |
| SERPINA3 | 2.475 | 0.425657216 | 2 | 1 |
| THBS3 | 2.475 | 0.425657216 | 2 | 1 |
| SELENON | 2.475 | 0.425657216 | 2 | 1 |
| PACSIN3 | 2.475 | 0.425657216 | 2 | 1 |
| IFIT3 | 2.475 | 0.425657216 | 2 | 1 |
| AKR1E2 | 2.475 | 0.425657216 | 2 | 1 |
| ZNF837 | 2.475 | 0.425657216 | 2 | 1 |
| IGLV3-21 | 2.475 | 0.425657216 | 2 | 1 |
| ACO2 | 2.475 | 0.425657216 | 2 | 1 |
| FBXW7 | 2.475 | 0.425657216 | 2 | 1 |
| TEKT5 | 2.475 | 0.425657216 | 2 | 1 |
| TMEM143 | 2.475 | 0.425657216 | 2 | 1 |
| SMIM15 | 2.475 | 0.425657216 | 2 | 1 |
| HHIP | 2.475 | 0.425657216 | 2 | 1 |
| CYP4F30P | 2.475 | 0.425657216 | 2 | 1 |
| OXGR1 | 2.475 | 0.425657216 | 2 | 1 |
| IGKV3-11 | 2.475 | 0.425657216 | 2 | 1 |
| SMG8 | 2.475 | 0.425657216 | 2 | 1 |
| OR2T12 | 2.475 | 0.425657216 | 2 | 1 |
| CYP4F11 | 2.475 | 0.425657216 | 2 | 1 |
| HID1 | 2.475 | 0.425657216 | 2 | 1 |
| OXER1 | 2.475 | 0.425657216 | 2 | 1 |
| NTHL1 | 2.475 | 0.425657216 | 2 | 1 |
| SLF2 | 2.475 | 0.425657216 | 2 | 1 |
| ZNF799 | 2.475 | 0.425657216 | 2 | 1 |
| ZNF415 | 2.475 | 0.425657216 | 2 | 1 |
| ANKIB1 | 2.475 | 0.425657216 | 2 | 1 |
| ANKK1 | 2.475 | 0.425657216 | 2 | 1 |
| P4HB | 2.475 | 0.425657216 | 2 | 1 |
| SART3 | 2.475 | 0.425657216 | 2 | 1 |
| TECPR1 | 2.475 | 0.425657216 | 2 | 1 |
| HEMK1 | 2.475 | 0.425657216 | 2 | 1 |
| BRINP3 | 2.475 | 0.425657216 | 2 | 1 |
| ACCS | 2.475 | 0.425657216 | 2 | 1 |
| ZNF404 | 2.475 | 0.425657216 | 2 | 1 |
| CNST | 2.475 | 0.425657216 | 2 | 1 |
| TMEM175 | 2.475 | 0.425657216 | 2 | 1 |
| NUDT16L1 | 2.475 | 0.425657216 | 2 | 1 |
| ACSM2B | 2.475 | 0.425657216 | 2 | 1 |
| DNAI2 | 2.475 | 0.425657216 | 2 | 1 |
| ANKRD10 | 2.475 | 0.425657216 | 2 | 1 |
| GPBP1L1 | 2.475 | 0.425657216 | 2 | 1 |
| HERC4 | 2.475 | 0.425657216 | 2 | 1 |
| NCAPD3 | 2.475 | 0.425657216 | 2 | 1 |
| NUDT16 | 2.475 | 0.425657216 | 2 | 1 |
| TEK | 2.475 | 0.425657216 | 2 | 1 |
| NUDT12 | 2.475 | 0.425657216 | 2 | 1 |
| ANKMY1 | 2.475 | 0.425657216 | 2 | 1 |
| ACIN1 | 2.475 | 0.425657216 | 2 | 1 |
| EZR | 2.475 | 0.425657216 | 2 | 1 |
| NDUFAF5 | 2.475 | 0.425657216 | 2 | 1 |
| CYP2E1 | 2.475 | 0.425657216 | 2 | 1 |
| RNF215 | 2.475 | 0.425657216 | 2 | 1 |
| COPZ2 | 2.475 | 0.425657216 | 2 | 1 |
| FCMR | 2.475 | 0.425657216 | 2 | 1 |
| IGFBPL1 | 2.475 | 0.425657216 | 2 | 1 |
| FBXO2 | 2.475 | 0.425657216 | 2 | 1 |
| NR6A1 | 2.475 | 0.425657216 | 2 | 1 |
| ALDH7A1 | 2.475 | 0.425657216 | 2 | 1 |
| OR4D5 | 2.475 | 0.425657216 | 2 | 1 |
| FBXO17 | 2.475 | 0.425657216 | 2 | 1 |
| NFATC2 | 2.475 | 0.425657216 | 2 | 1 |
| CYP26B1 | 2.475 | 0.425657216 | 2 | 1 |
| SAE1 | 2.475 | 0.425657216 | 2 | 1 |
| ACVR1C | 2.475 | 0.425657216 | 2 | 1 |
| ROBO2 | 2.475 | 0.425657216 | 2 | 1 |
| OR7C1 | 2.475 | 0.425657216 | 2 | 1 |
| ZNF546 | 2.475 | 0.425657216 | 2 | 1 |
| ZNF787 | 2.475 | 0.425657216 | 2 | 1 |
| ACY3 | 2.475 | 0.425657216 | 2 | 1 |
| IFNA13 | 2.475 | 0.425657216 | 2 | 1 |
| TANGO6 | 2.475 | 0.425657216 | 2 | 1 |
| FAM163B | 2.475 | 0.425657216 | 2 | 1 |
| IFT88 | 2.475 | 0.425657216 | 2 | 1 |
| SOAT1 | 2.475 | 0.425657216 | 2 | 1 |
| FAM170A | 2.475 | 0.425657216 | 2 | 1 |
| OSBPL10 | 2.475 | 0.425657216 | 2 | 1 |
| AMER3 | 2.475 | 0.425657216 | 2 | 1 |
| GPD1 | 2.475 | 0.425657216 | 2 | 1 |
| SLC7A9 | 2.475 | 0.425657216 | 2 | 1 |
| AMBP | 2.475 | 0.425657216 | 2 | 1 |
| SPAG5 | 2.475 | 0.425657216 | 2 | 1 |
| FAM170B | 2.475 | 0.425657216 | 2 | 1 |
| NECAB2 | 2.475 | 0.425657216 | 2 | 1 |
| NRK | 2.475 | 0.425657216 | 2 | 1 |
| CYP2B6 | 2.475 | 0.425657216 | 2 | 1 |
| ALG10B | 2.475 | 0.425657216 | 2 | 1 |
| ACTL8 | 2.475 | 0.425657216 | 2 | 1 |
| TMEM102 | 2.475 | 0.425657216 | 2 | 1 |
| HIST1H3A | 2.475 | 0.425657216 | 2 | 1 |
| NEFL | 2.475 | 0.425657216 | 2 | 1 |
| SELENBP1 | 2.475 | 0.425657216 | 2 | 1 |
| FBXO33 | 2.475 | 0.425657216 | 2 | 1 |
| GRIK3 | 2.475 | 0.425657216 | 2 | 1 |
| RNF40 | 2.475 | 0.425657216 | 2 | 1 |
| ZNF48 | 2.475 | 0.425657216 | 2 | 1 |
| NEK4 | 2.475 | 0.425657216 | 2 | 1 |
| COL9A3 | 2.475 | 0.425657216 | 2 | 1 |
| HTATSF1 | 2.475 | 0.425657216 | 2 | 1 |
| USP28 | 2.475 | 0.425657216 | 2 | 1 |
| BBS9 | 2.475 | 0.425657216 | 2 | 1 |
| BMPER | 2.475 | 0.425657216 | 2 | 1 |
| MARCKS | 2.475 | 0.425657216 | 2 | 1 |
| EBF3 | 2.475 | 0.425657216 | 2 | 1 |
| PRSS23 | 2.475 | 0.425657216 | 2 | 1 |
| WEE2 | 2.475 | 0.425657216 | 2 | 1 |
| PLA2G6 | 2.475 | 0.425657216 | 2 | 1 |
| L3MBTL1 | 2.475 | 0.425657216 | 2 | 1 |
| PLA2G4F | 2.475 | 0.425657216 | 2 | 1 |
| MASTL | 2.475 | 0.425657216 | 2 | 1 |
| SREBF2 | 2.475 | 0.425657216 | 2 | 1 |
| WHAMM | 2.475 | 0.425657216 | 2 | 1 |
| SLC2A7 | 2.475 | 0.425657216 | 2 | 1 |
| CCDC13 | 2.475 | 0.425657216 | 2 | 1 |
| TTC14 | 2.475 | 0.425657216 | 2 | 1 |
| STX6 | 2.475 | 0.425657216 | 2 | 1 |
| DRC7 | 2.475 | 0.425657216 | 2 | 1 |
| PSAPL1 | 2.475 | 0.425657216 | 2 | 1 |
| CCDC138 | 2.475 | 0.425657216 | 2 | 1 |
| CCDC14 | 2.475 | 0.425657216 | 2 | 1 |
| KRTAP9-6 | 2.475 | 0.425657216 | 2 | 1 |
| KRTAP9-3 | 2.475 | 0.425657216 | 2 | 1 |
| PSD4 | 2.475 | 0.425657216 | 2 | 1 |
| PSEN2 | 2.475 | 0.425657216 | 2 | 1 |
| ENTPD2 | 2.475 | 0.425657216 | 2 | 1 |
| CCDC154 | 2.475 | 0.425657216 | 2 | 1 |
| BHLHE22 | 2.475 | 0.425657216 | 2 | 1 |
| PKDCC | 2.475 | 0.425657216 | 2 | 1 |
| MARCH9 | 2.475 | 0.425657216 | 2 | 1 |
| SLC2A5 | 2.475 | 0.425657216 | 2 | 1 |
| BNC2 | 2.475 | 0.425657216 | 2 | 1 |
| MARCH10 | 2.475 | 0.425657216 | 2 | 1 |
| MANSC1 | 2.475 | 0.425657216 | 2 | 1 |
| PLEKHA1 | 2.475 | 0.425657216 | 2 | 1 |
| SLC29A1 | 2.475 | 0.425657216 | 2 | 1 |
| PLEK2 | 2.475 | 0.425657216 | 2 | 1 |
| CASC3 | 2.475 | 0.425657216 | 2 | 1 |
| BRPF1 | 2.475 | 0.425657216 | 2 | 1 |
| LCLAT1 | 2.475 | 0.425657216 | 2 | 1 |
| TTPA | 2.475 | 0.425657216 | 2 | 1 |
| LCE6A | 2.475 | 0.425657216 | 2 | 1 |
| MAP3K13 | 2.475 | 0.425657216 | 2 | 1 |
| WDR38 | 2.475 | 0.425657216 | 2 | 1 |
| CASP5 | 2.475 | 0.425657216 | 2 | 1 |
| LCE2D | 2.475 | 0.425657216 | 2 | 1 |
| MBD6 | 2.475 | 0.425657216 | 2 | 1 |
| MAP4K1 | 2.475 | 0.425657216 | 2 | 1 |
| PRR23B | 2.475 | 0.425657216 | 2 | 1 |
| MAPK15 | 2.475 | 0.425657216 | 2 | 1 |
| CATSPERD | 2.475 | 0.425657216 | 2 | 1 |
| GCGR | 2.475 | 0.425657216 | 2 | 1 |
| PRR5 | 2.475 | 0.425657216 | 2 | 1 |
| BPIFB3 | 2.475 | 0.425657216 | 2 | 1 |
| SLC2A2 | 2.475 | 0.425657216 | 2 | 1 |
| MAPKAPK2 | 2.475 | 0.425657216 | 2 | 1 |
| BPI | 2.475 | 0.425657216 | 2 | 1 |
| MAPKBP1 | 2.475 | 0.425657216 | 2 | 1 |
| TTC34 | 2.475 | 0.425657216 | 2 | 1 |
| CBS | 2.475 | 0.425657216 | 2 | 1 |
| CBWD2 | 2.475 | 0.425657216 | 2 | 1 |
| PLCD1 | 2.475 | 0.425657216 | 2 | 1 |
| DZIP3 | 2.475 | 0.425657216 | 2 | 1 |
| STXBP5L | 2.475 | 0.425657216 | 2 | 1 |
| KRTAP2-4 | 2.475 | 0.425657216 | 2 | 1 |
| ATP23 | 2.475 | 0.425657216 | 2 | 1 |
| XRN1 | 2.475 | 0.425657216 | 2 | 1 |
| VCPKMT | 2.475 | 0.425657216 | 2 | 1 |
| CCNF | 2.475 | 0.425657216 | 2 | 1 |
| KLLN | 2.475 | 0.425657216 | 2 | 1 |
| SPTAN1 | 2.475 | 0.425657216 | 2 | 1 |
| XXYLT1 | 2.475 | 0.425657216 | 2 | 1 |
| B4GALT1 | 2.475 | 0.425657216 | 2 | 1 |
| TRIP12 | 2.475 | 0.425657216 | 2 | 1 |
| PUS10 | 2.475 | 0.425657216 | 2 | 1 |
| EPHB3 | 2.475 | 0.425657216 | 2 | 1 |
| MFSD6 | 2.475 | 0.425657216 | 2 | 1 |
| B3GALNT2 | 2.475 | 0.425657216 | 2 | 1 |
| TRMT5 | 2.475 | 0.425657216 | 2 | 1 |
| CCT2 | 2.475 | 0.425657216 | 2 | 1 |
| PHKG1 | 2.475 | 0.425657216 | 2 | 1 |
| SPSB3 | 2.475 | 0.425657216 | 2 | 1 |
| MGST2 | 2.475 | 0.425657216 | 2 | 1 |
| DUSP16 | 2.475 | 0.425657216 | 2 | 1 |
| AVL9 | 2.475 | 0.425657216 | 2 | 1 |
| KLHDC7B | 2.475 | 0.425657216 | 2 | 1 |
| AUH | 2.475 | 0.425657216 | 2 | 1 |
| MICAL2 | 2.475 | 0.425657216 | 2 | 1 |
| PHF23 | 2.475 | 0.425657216 | 2 | 1 |
| SUMF2 | 2.475 | 0.425657216 | 2 | 1 |
| MIDN | 2.475 | 0.425657216 | 2 | 1 |
| ATP9B | 2.475 | 0.425657216 | 2 | 1 |
| TRIM49D2 | 2.475 | 0.425657216 | 2 | 1 |
| PXDN | 2.475 | 0.425657216 | 2 | 1 |
| PROM2 | 2.475 | 0.425657216 | 2 | 1 |
| ADGRB2 | 2.475 | 0.425657216 | 2 | 1 |
| KNOP1 | 2.475 | 0.425657216 | 2 | 1 |
| PSME4 | 2.475 | 0.425657216 | 2 | 1 |
| SFI1 | 2.475 | 0.425657216 | 2 | 1 |
| WNT7A | 2.475 | 0.425657216 | 2 | 1 |
| BCO1 | 2.475 | 0.425657216 | 2 | 1 |
| MCM7 | 2.475 | 0.425657216 | 2 | 1 |
| PSTK | 2.475 | 0.425657216 | 2 | 1 |
| PTCHD1 | 2.475 | 0.425657216 | 2 | 1 |
| PCDHGA5 | 2.475 | 0.425657216 | 2 | 1 |
| EPB41L3 | 2.475 | 0.425657216 | 2 | 1 |
| PINK1 | 2.475 | 0.425657216 | 2 | 1 |
| KRT77 | 2.475 | 0.425657216 | 2 | 1 |
| SIX4 | 2.475 | 0.425657216 | 2 | 1 |
| CCDC71L | 2.475 | 0.425657216 | 2 | 1 |
| KNG1 | 2.475 | 0.425657216 | 2 | 1 |
| BCAS3 | 2.475 | 0.425657216 | 2 | 1 |
| PCDH1 | 2.475 | 0.425657216 | 2 | 1 |
| PIK3CB | 2.475 | 0.425657216 | 2 | 1 |
| KRT32 | 2.475 | 0.425657216 | 2 | 1 |
| PIK3C2B | 2.475 | 0.425657216 | 2 | 1 |
| BBS1 | 2.475 | 0.425657216 | 2 | 1 |
| PTPN7 | 2.475 | 0.425657216 | 2 | 1 |
| MEGF10 | 2.475 | 0.425657216 | 2 | 1 |
| MEIS3P1 | 2.475 | 0.425657216 | 2 | 1 |
| KRT17 | 2.475 | 0.425657216 | 2 | 1 |
| MEP1B | 2.475 | 0.425657216 | 2 | 1 |
| SIRT1 | 2.475 | 0.425657216 | 2 | 1 |
| METRNL | 2.475 | 0.425657216 | 2 | 1 |
| PTPRK | 2.475 | 0.425657216 | 2 | 1 |
| TRPM5 | 2.475 | 0.425657216 | 2 | 1 |
| SRPRA | 2.475 | 0.425657216 | 2 | 1 |
| GBP2 | 2.475 | 0.425657216 | 2 | 1 |
| BTBD2 | 2.475 | 0.425657216 | 2 | 1 |
| GABRG1 | 2.475 | 0.425657216 | 2 | 1 |
| LRRC27 | 2.475 | 0.425657216 | 2 | 1 |
| LOC101928548 | 2.475 | 0.425657216 | 2 | 1 |
| LRRC28 | 2.475 | 0.425657216 | 2 | 1 |
| PPP1R13B | 2.475 | 0.425657216 | 2 | 1 |
| PPP1R1A | 2.475 | 0.425657216 | 2 | 1 |
| C4orf50 | 2.475 | 0.425657216 | 2 | 1 |
| C17orf97 | 2.475 | 0.425657216 | 2 | 1 |
| C17orf75 | 2.475 | 0.425657216 | 2 | 1 |
| PPP1R3F | 2.475 | 0.425657216 | 2 | 1 |
| ELN | 2.475 | 0.425657216 | 2 | 1 |
| VEGFA | 2.475 | 0.425657216 | 2 | 1 |
| POLE2 | 2.475 | 0.425657216 | 2 | 1 |
| LRRC19 | 2.475 | 0.425657216 | 2 | 1 |
| C17orf50 | 2.475 | 0.425657216 | 2 | 1 |
| UNC5D | 2.475 | 0.425657216 | 2 | 1 |
| SDHAF4 | 2.475 | 0.425657216 | 2 | 1 |
| C6orf58 | 2.475 | 0.425657216 | 2 | 1 |
| C6orf89 | 2.475 | 0.425657216 | 2 | 1 |
| UNC13C | 2.475 | 0.425657216 | 2 | 1 |
| C7orf50 | 2.475 | 0.425657216 | 2 | 1 |
| PRR35 | 2.475 | 0.425657216 | 2 | 1 |
| LOC100507003 | 2.475 | 0.425657216 | 2 | 1 |
| C7orf61 | 2.475 | 0.425657216 | 2 | 1 |
| POGLUT1 | 2.475 | 0.425657216 | 2 | 1 |
| LOC100505679 | 2.475 | 0.425657216 | 2 | 1 |
| C8B | 2.475 | 0.425657216 | 2 | 1 |
| TMEM266 | 2.475 | 0.425657216 | 2 | 1 |
| C16orf89 | 2.475 | 0.425657216 | 2 | 1 |
| PODXL2 | 2.475 | 0.425657216 | 2 | 1 |
| USHBP1 | 2.475 | 0.425657216 | 2 | 1 |
| STAR | 2.475 | 0.425657216 | 2 | 1 |
| USP26 | 2.475 | 0.425657216 | 2 | 1 |
| ST3GAL6 | 2.475 | 0.425657216 | 2 | 1 |
| USP25 | 2.475 | 0.425657216 | 2 | 1 |
| LOC730183 | 2.475 | 0.425657216 | 2 | 1 |
| RTL10 | 2.475 | 0.425657216 | 2 | 1 |
| USP19 | 2.475 | 0.425657216 | 2 | 1 |
| USP33 | 2.475 | 0.425657216 | 2 | 1 |
| USP35 | 2.475 | 0.425657216 | 2 | 1 |
| FZD9 | 2.475 | 0.425657216 | 2 | 1 |
| SLC25A29 | 2.475 | 0.425657216 | 2 | 1 |
| C2CD4C | 2.475 | 0.425657216 | 2 | 1 |
| FUT10 | 2.475 | 0.425657216 | 2 | 1 |
| SLC24A1 | 2.475 | 0.425657216 | 2 | 1 |
| FUCA1 | 2.475 | 0.425657216 | 2 | 1 |
| LPIN3 | 2.475 | 0.425657216 | 2 | 1 |
| SLC22A9 | 2.475 | 0.425657216 | 2 | 1 |
| C1orf109 | 2.475 | 0.425657216 | 2 | 1 |
| POTEB | 2.475 | 0.425657216 | 2 | 1 |
| ELFN2 | 2.475 | 0.425657216 | 2 | 1 |
| LRIG3 | 2.475 | 0.425657216 | 2 | 1 |
| SCUBE2 | 2.475 | 0.425657216 | 2 | 1 |
| POM121L12 | 2.475 | 0.425657216 | 2 | 1 |
| GAB4 | 2.475 | 0.425657216 | 2 | 1 |
| USP15 | 2.475 | 0.425657216 | 2 | 1 |
| PROSER3 | 2.475 | 0.425657216 | 2 | 1 |
| USP13 | 2.475 | 0.425657216 | 2 | 1 |
| POLR3A | 2.475 | 0.425657216 | 2 | 1 |
| PPME1 | 2.475 | 0.425657216 | 2 | 1 |
| USP6NL | 2.475 | 0.425657216 | 2 | 1 |
| KLF12 | 2.475 | 0.425657216 | 2 | 1 |
| LRRC8E | 2.475 | 0.425657216 | 2 | 1 |
| EGFL6 | 2.475 | 0.425657216 | 2 | 1 |
| LIMS2 | 2.475 | 0.425657216 | 2 | 1 |
| LIMS1 | 2.475 | 0.425657216 | 2 | 1 |
| UBA5 | 2.475 | 0.425657216 | 2 | 1 |
| GAPDHS | 2.475 | 0.425657216 | 2 | 1 |
| SLC18B1 | 2.475 | 0.425657216 | 2 | 1 |
| GAS2L2 | 2.475 | 0.425657216 | 2 | 1 |
| VWC2 | 2.475 | 0.425657216 | 2 | 1 |
| PLEKHO2 | 2.475 | 0.425657216 | 2 | 1 |
| CAMK1 | 2.475 | 0.425657216 | 2 | 1 |
| EDNRB | 2.475 | 0.425657216 | 2 | 1 |
| TYMP | 2.475 | 0.425657216 | 2 | 1 |
| WAC | 2.475 | 0.425657216 | 2 | 1 |
| BZW1 | 2.475 | 0.425657216 | 2 | 1 |
| PRDM6 | 2.475 | 0.425657216 | 2 | 1 |
| MAGEA6 | 2.475 | 0.425657216 | 2 | 1 |
| MAGEB1 | 2.475 | 0.425657216 | 2 | 1 |
| LGALS12 | 2.475 | 0.425657216 | 2 | 1 |
| CENPX | 2.475 | 0.425657216 | 2 | 1 |
| MAGEC3 | 2.475 | 0.425657216 | 2 | 1 |
| SERPINB8 | 2.475 | 0.425657216 | 2 | 1 |
| ENDOG | 2.475 | 0.425657216 | 2 | 1 |
| GATS | 2.475 | 0.425657216 | 2 | 1 |
| PRLR | 2.475 | 0.425657216 | 2 | 1 |
| PLEKHG6 | 2.475 | 0.425657216 | 2 | 1 |
| TULP2 | 2.475 | 0.425657216 | 2 | 1 |
| ENDOV | 2.475 | 0.425657216 | 2 | 1 |
| LEMD2 | 2.475 | 0.425657216 | 2 | 1 |
| MALSU1 | 2.475 | 0.425657216 | 2 | 1 |
| TXNDC11 | 2.475 | 0.425657216 | 2 | 1 |
| GALNS | 2.475 | 0.425657216 | 2 | 1 |
| EML5 | 2.475 | 0.425657216 | 2 | 1 |
| UBE2O | 2.475 | 0.425657216 | 2 | 1 |
| STEAP3 | 2.475 | 0.425657216 | 2 | 1 |
| EMC1 | 2.475 | 0.425657216 | 2 | 1 |
| STIP1 | 2.475 | 0.425657216 | 2 | 1 |
| EMC10 | 2.475 | 0.425657216 | 2 | 1 |
| UHRF1BP1 | 2.475 | 0.425657216 | 2 | 1 |
| UHMK1 | 2.475 | 0.425657216 | 2 | 1 |
| UGT2B15 | 2.475 | 0.425657216 | 2 | 1 |
| LRRN4 | 2.475 | 0.425657216 | 2 | 1 |
| LRSAM1 | 2.475 | 0.425657216 | 2 | 1 |
| UGT1A10 | 2.475 | 0.425657216 | 2 | 1 |
| C9orf50 | 2.475 | 0.425657216 | 2 | 1 |
| LTF | 2.475 | 0.425657216 | 2 | 1 |
| EFHC2 | 2.475 | 0.425657216 | 2 | 1 |
| CACNB2 | 2.475 | 0.425657216 | 2 | 1 |
| LMF2 | 2.475 | 0.425657216 | 2 | 1 |
| CA2 | 2.475 | 0.425657216 | 2 | 1 |
| FRMPD3 | 2.475 | 0.425657216 | 2 | 1 |
| UBXN1 | 2.475 | 0.425657216 | 2 | 1 |
| PRAMEF6 | 2.475 | 0.425657216 | 2 | 1 |
| LY6G5C | 2.475 | 0.425657216 | 2 | 1 |
| CABP5 | 2.475 | 0.425657216 | 2 | 1 |
| GALR3 | 2.475 | 0.425657216 | 2 | 1 |
| EFCAB7 | 2.475 | 0.425657216 | 2 | 1 |
| C11orf65 | 2.475 | 0.425657216 | 2 | 1 |
| UBE4B | 2.475 | 0.425657216 | 2 | 1 |
| PMS2 | 2.475 | 0.425657216 | 2 | 1 |
| LYSMD1 | 2.475 | 0.425657216 | 2 | 1 |
| GANAB | 2.475 | 0.425657216 | 2 | 1 |
| VPS4A | 2.475 | 0.425657216 | 2 | 1 |
| PYROXD2 | 2.475 | 0.425657216 | 2 | 1 |
| MARCO | 2.475 | 0.425657216 | 2 | 1 |
| MOCS1 | 2.475 | 0.425657216 | 2 | 1 |
| SH2B1 | 2.475 | 0.425657216 | 2 | 1 |
| MORF4L1 | 2.475 | 0.425657216 | 2 | 1 |
| MOV10L1 | 2.475 | 0.425657216 | 2 | 1 |
| GJB3 | 2.475 | 0.425657216 | 2 | 1 |
| KIAA0319 | 2.475 | 0.425657216 | 2 | 1 |
| ASIC1 | 2.475 | 0.425657216 | 2 | 1 |
| TRAPPC10 | 2.475 | 0.425657216 | 2 | 1 |
| PUM3 | 2.475 | 0.425657216 | 2 | 1 |
| FHDC1 | 2.475 | 0.425657216 | 2 | 1 |
| PER2 | 2.475 | 0.425657216 | 2 | 1 |
| FHAD1 | 2.475 | 0.425657216 | 2 | 1 |
| SYNJ2 | 2.475 | 0.425657216 | 2 | 1 |
| PCDHGB6 | 2.475 | 0.425657216 | 2 | 1 |
| TRAK1 | 2.475 | 0.425657216 | 2 | 1 |
| KDM5B | 2.475 | 0.425657216 | 2 | 1 |
| MRE11 | 2.475 | 0.425657216 | 2 | 1 |
| KDM4E | 2.475 | 0.425657216 | 2 | 1 |
| ZMYM3 | 2.475 | 0.425657216 | 2 | 1 |
| PDXK | 2.475 | 0.425657216 | 2 | 1 |
| ZDHHC5 | 2.475 | 0.425657216 | 2 | 1 |
| GLB1L | 2.475 | 0.425657216 | 2 | 1 |
| CHAC1 | 2.475 | 0.425657216 | 2 | 1 |
| KIAA1143 | 2.475 | 0.425657216 | 2 | 1 |
| MTX1 | 2.475 | 0.425657216 | 2 | 1 |
| MMRN2 | 2.475 | 0.425657216 | 2 | 1 |
| ZC3H7B | 2.475 | 0.425657216 | 2 | 1 |
| DSG4 | 2.475 | 0.425657216 | 2 | 1 |
| ZNF175 | 2.475 | 0.425657216 | 2 | 1 |
| RCSD1 | 2.475 | 0.425657216 | 2 | 1 |
| CDCP2 | 2.475 | 0.425657216 | 2 | 1 |
| SGPP2 | 2.475 | 0.425657216 | 2 | 1 |
| ARFGAP3 | 2.475 | 0.425657216 | 2 | 1 |
| CHIA | 2.475 | 0.425657216 | 2 | 1 |
| ARHGAP17 | 2.475 | 0.425657216 | 2 | 1 |
| RCCD1 | 2.475 | 0.425657216 | 2 | 1 |
| ATAT1 | 2.475 | 0.425657216 | 2 | 1 |
| TOR2A | 2.475 | 0.425657216 | 2 | 1 |
| ZCCHC3 | 2.475 | 0.425657216 | 2 | 1 |
| FIGNL2 | 2.475 | 0.425657216 | 2 | 1 |
| VIRMA | 2.475 | 0.425657216 | 2 | 1 |
| JAKMIP3 | 2.475 | 0.425657216 | 2 | 1 |
| RAD51AP2 | 2.475 | 0.425657216 | 2 | 1 |
| CHEK2 | 2.475 | 0.425657216 | 2 | 1 |
| CEMIP | 2.475 | 0.425657216 | 2 | 1 |
| ATCAY | 2.475 | 0.425657216 | 2 | 1 |
| ARHGAP6 | 2.475 | 0.425657216 | 2 | 1 |
| RAPGEF5 | 2.475 | 0.425657216 | 2 | 1 |
| MROH2B | 2.475 | 0.425657216 | 2 | 1 |
| ARMC7 | 2.475 | 0.425657216 | 2 | 1 |
| PDE7B | 2.475 | 0.425657216 | 2 | 1 |
| GLTP | 2.475 | 0.425657216 | 2 | 1 |
| SLC3A1 | 2.475 | 0.425657216 | 2 | 1 |
| SWT1 | 2.475 | 0.425657216 | 2 | 1 |
| ARL6IP5 | 2.475 | 0.425657216 | 2 | 1 |
| RBM15B | 2.475 | 0.425657216 | 2 | 1 |
| MRS2 | 2.475 | 0.425657216 | 2 | 1 |
| SH3BP4 | 2.475 | 0.425657216 | 2 | 1 |
| KCNK5 | 2.475 | 0.425657216 | 2 | 1 |
| CEP120 | 2.475 | 0.425657216 | 2 | 1 |
| PDCD2L | 2.475 | 0.425657216 | 2 | 1 |
| ARL13B | 2.475 | 0.425657216 | 2 | 1 |
| RBL2 | 2.475 | 0.425657216 | 2 | 1 |
| ZIM3 | 2.475 | 0.425657216 | 2 | 1 |
| SYMPK | 2.475 | 0.425657216 | 2 | 1 |
| MSRA | 2.475 | 0.425657216 | 2 | 1 |
| TOPBP1 | 2.475 | 0.425657216 | 2 | 1 |
| KCNG1 | 2.475 | 0.425657216 | 2 | 1 |
| ETV3L | 2.475 | 0.425657216 | 2 | 1 |
| TONSL | 2.475 | 0.425657216 | 2 | 1 |
| ETV5 | 2.475 | 0.425657216 | 2 | 1 |
| CFB | 2.475 | 0.425657216 | 2 | 1 |
| RASGRP4 | 2.475 | 0.425657216 | 2 | 1 |
| SHANK3 | 2.475 | 0.425657216 | 2 | 1 |
| ARHGEF10L | 2.475 | 0.425657216 | 2 | 1 |
| FGFR4 | 2.475 | 0.425657216 | 2 | 1 |
| MRPL10 | 2.475 | 0.425657216 | 2 | 1 |
| DRC1 | 2.475 | 0.425657216 | 2 | 1 |
| FGFR3 | 2.475 | 0.425657216 | 2 | 1 |
| KCNV2 | 2.475 | 0.425657216 | 2 | 1 |
| PDLIM2 | 2.475 | 0.425657216 | 2 | 1 |
| PDK4 | 2.475 | 0.425657216 | 2 | 1 |
| CEBPD | 2.475 | 0.425657216 | 2 | 1 |
| KAT6A | 2.475 | 0.425657216 | 2 | 1 |
| KAT6B | 2.475 | 0.425657216 | 2 | 1 |
| MRPL24 | 2.475 | 0.425657216 | 2 | 1 |
| MTERF3 | 2.475 | 0.425657216 | 2 | 1 |
| MTDH | 2.475 | 0.425657216 | 2 | 1 |
| MRPL38 | 2.475 | 0.425657216 | 2 | 1 |
| ARHGEF26 | 2.475 | 0.425657216 | 2 | 1 |
| FGF22 | 2.475 | 0.425657216 | 2 | 1 |
| KBTBD11 | 2.475 | 0.425657216 | 2 | 1 |
| MTA2 | 2.475 | 0.425657216 | 2 | 1 |
| RASGRP1 | 2.475 | 0.425657216 | 2 | 1 |
| KIAA1671 | 2.475 | 0.425657216 | 2 | 1 |
| TRDV3 | 2.475 | 0.425657216 | 2 | 1 |
| PCSK7 | 2.475 | 0.425657216 | 2 | 1 |
| ATP2A1 | 2.475 | 0.425657216 | 2 | 1 |
| FKRP | 2.475 | 0.425657216 | 2 | 1 |
| PFAS | 2.475 | 0.425657216 | 2 | 1 |
| ATP8A2 | 2.475 | 0.425657216 | 2 | 1 |
| CHST7 | 2.475 | 0.425657216 | 2 | 1 |
| CDC14B | 2.475 | 0.425657216 | 2 | 1 |
| RFESD | 2.475 | 0.425657216 | 2 | 1 |
| FMO4 | 2.475 | 0.425657216 | 2 | 1 |
| MUTYH | 2.475 | 0.425657216 | 2 | 1 |
| CIZ1 | 2.475 | 0.425657216 | 2 | 1 |
| RFC3 | 2.475 | 0.425657216 | 2 | 1 |
| SPATA4 | 2.475 | 0.425657216 | 2 | 1 |
| PHACTR2 | 2.475 | 0.425657216 | 2 | 1 |
| ERCC4 | 2.475 | 0.425657216 | 2 | 1 |
| TRIM42 | 2.475 | 0.425657216 | 2 | 1 |
| MLYCD | 2.475 | 0.425657216 | 2 | 1 |
| TRIM22 | 2.475 | 0.425657216 | 2 | 1 |
| MME | 2.475 | 0.425657216 | 2 | 1 |
| QRICH1 | 2.475 | 0.425657216 | 2 | 1 |
| MYH3 | 2.475 | 0.425657216 | 2 | 1 |
| ZBTB44 | 2.475 | 0.425657216 | 2 | 1 |
| PCDH7 | 2.475 | 0.425657216 | 2 | 1 |
| SIGLEC10 | 2.475 | 0.425657216 | 2 | 1 |
| CILP2 | 2.475 | 0.425657216 | 2 | 1 |
| RAB11FIP5 | 2.475 | 0.425657216 | 2 | 1 |
| EPS8 | 2.475 | 0.425657216 | 2 | 1 |
| SYT17 | 2.475 | 0.425657216 | 2 | 1 |
| DOCK3 | 2.475 | 0.425657216 | 2 | 1 |
| LNPK | 2.475 | 0.425657216 | 2 | 1 |
| CHRND | 2.475 | 0.425657216 | 2 | 1 |
| SPATA7 | 2.475 | 0.425657216 | 2 | 1 |
| APLF | 2.475 | 0.425657216 | 2 | 1 |
| ATP6V0A2 | 2.475 | 0.425657216 | 2 | 1 |
| PCDHA10 | 2.475 | 0.425657216 | 2 | 1 |
| RDH5 | 2.475 | 0.425657216 | 2 | 1 |
| ERCC6L | 2.475 | 0.425657216 | 2 | 1 |
| RETSAT | 2.475 | 0.425657216 | 2 | 1 |
| SLC45A1 | 2.475 | 0.425657216 | 2 | 1 |
| TREML4 | 2.475 | 0.425657216 | 2 | 1 |
| SHPK | 2.475 | 0.425657216 | 2 | 1 |
| QTRT1 | 2.475 | 0.425657216 | 2 | 1 |
| ATP5F1 | 2.475 | 0.425657216 | 2 | 1 |
| ZBTB4 | 2.475 | 0.425657216 | 2 | 1 |
| MLF1 | 2.475 | 0.425657216 | 2 | 1 |
| SYT16 | 2.475 | 0.425657216 | 2 | 1 |
| RDH8 | 2.475 | 0.425657216 | 2 | 1 |
| AQP6 | 2.475 | 0.425657216 | 2 | 1 |
| MMP3 | 2.475 | 0.425657216 | 2 | 1 |
| PCDHA5 | 2.475 | 0.425657216 | 2 | 1 |
| ATG9A | 2.475 | 0.425657216 | 2 | 1 |
| KIT | 2.475 | 0.425657216 | 2 | 1 |
| IVD | 2.475 | 0.425657216 | 2 | 1 |
| EXOC3 | 2.475 | 0.425657216 | 2 | 1 |
| REP15 | 2.475 | 0.425657216 | 2 | 1 |
| SIGLEC11 | 1.102564103 | 0.429243446 | 43 | 50 |
| CR1 | 1.099173554 | 0.433857204 | 38 | 44 |
| CRYBG1 | 1.243243243 | 0.436568904 | 8 | 8 |
| IFNA4 | 1.243243243 | 0.436568904 | 8 | 8 |
| VCX2 | 1.666666667 | 0.438973923 | 80 | 96 |
| FRMPD2 | 1.157349896 | 0.442357274 | 13 | 14 |
| GLUL | inf | 0.450549451 | 1 | 0 |
| FGD2 | inf | 0.450549451 | 1 | 0 |
| SECTM1 | inf | 0.450549451 | 1 | 0 |
| GALNT6 | inf | 0.450549451 | 1 | 0 |
| GALNTL5 | inf | 0.450549451 | 1 | 0 |
| GALNT4 | inf | 0.450549451 | 1 | 0 |
| CPTP | inf | 0.450549451 | 1 | 0 |
| GRK1 | inf | 0.450549451 | 1 | 0 |
| GRIN2D | inf | 0.450549451 | 1 | 0 |
| GOLPH3 | inf | 0.450549451 | 1 | 0 |
| FZD2 | inf | 0.450549451 | 1 | 0 |
| SNCG | inf | 0.450549451 | 1 | 0 |
| SCYL3 | inf | 0.450549451 | 1 | 0 |
| FZD9 | inf | 0.450549451 | 1 | 0 |
| SLC25A17 | inf | 0.450549451 | 1 | 0 |
| FAM216A | inf | 0.450549451 | 1 | 0 |
| SLC25A10 | inf | 0.450549451 | 1 | 0 |
| GNPDA2 | inf | 0.450549451 | 1 | 0 |
| FAM216B | inf | 0.450549451 | 1 | 0 |
| SLC1A1 | inf | 0.450549451 | 1 | 0 |
| SH2D5 | inf | 0.450549451 | 1 | 0 |
| GLT8D2 | inf | 0.450549451 | 1 | 0 |
| FCGR3B | inf | 0.450549451 | 1 | 0 |
| FXR2 | inf | 0.450549451 | 1 | 0 |
| FAM69B | inf | 0.450549451 | 1 | 0 |
| FER | inf | 0.450549451 | 1 | 0 |
| SNAPC2 | inf | 0.450549451 | 1 | 0 |
| GLTPD2 | inf | 0.450549451 | 1 | 0 |
| SDC3 | inf | 0.450549451 | 1 | 0 |
| GRINA | inf | 0.450549451 | 1 | 0 |
| GALNT9 | inf | 0.450549451 | 1 | 0 |
| SLC25A6 | inf | 0.450549451 | 1 | 0 |
| FSCN2 | inf | 0.450549451 | 1 | 0 |
| FZD1 | inf | 0.450549451 | 1 | 0 |
| G3BP1 | inf | 0.450549451 | 1 | 0 |
| FAM35A | inf | 0.450549451 | 1 | 0 |
| GRN | inf | 0.450549451 | 1 | 0 |
| FUCA2 | inf | 0.450549451 | 1 | 0 |
| FSTL1 | inf | 0.450549451 | 1 | 0 |
| SLC45A2 | inf | 0.450549451 | 1 | 0 |
| FAM228A | inf | 0.450549451 | 1 | 0 |
| SLC25A51 | inf | 0.450549451 | 1 | 0 |
| SGTB | inf | 0.450549451 | 1 | 0 |
| SLC22A10 | inf | 0.450549451 | 1 | 0 |
| FDX2 | inf | 0.450549451 | 1 | 0 |
| GMPR2 | inf | 0.450549451 | 1 | 0 |
| GABRB3 | inf | 0.450549451 | 1 | 0 |
| GABRD | inf | 0.450549451 | 1 | 0 |
| SLC25A36 | inf | 0.450549451 | 1 | 0 |
| SGCD | inf | 0.450549451 | 1 | 0 |
| SFRP2 | inf | 0.450549451 | 1 | 0 |
| SLC22A18 | inf | 0.450549451 | 1 | 0 |
| SLC52A3 | inf | 0.450549451 | 1 | 0 |
| GAGE2C | inf | 0.450549451 | 1 | 0 |
| SLC25A38 | inf | 0.450549451 | 1 | 0 |
| SLC22A2 | inf | 0.450549451 | 1 | 0 |
| GAGE12D | inf | 0.450549451 | 1 | 0 |
| GABRG3 | inf | 0.450549451 | 1 | 0 |
| SMURF2 | inf | 0.450549451 | 1 | 0 |
| GAGE1 | inf | 0.450549451 | 1 | 0 |
| SLC4A8 | inf | 0.450549451 | 1 | 0 |
| GAD2 | inf | 0.450549451 | 1 | 0 |
| GABRR2 | inf | 0.450549451 | 1 | 0 |
| GAL | inf | 0.450549451 | 1 | 0 |
| FAM222B | inf | 0.450549451 | 1 | 0 |
| GSTP1 | inf | 0.450549451 | 1 | 0 |
| GABRA1 | inf | 0.450549451 | 1 | 0 |
| FER1L4 | inf | 0.450549451 | 1 | 0 |
| GNMT | inf | 0.450549451 | 1 | 0 |
| SDHB | inf | 0.450549451 | 1 | 0 |
| SLC25A31 | inf | 0.450549451 | 1 | 0 |
| SFR1 | inf | 0.450549451 | 1 | 0 |
| SDHD | inf | 0.450549451 | 1 | 0 |
| SLC45A3 | inf | 0.450549451 | 1 | 0 |
| FGD1 | inf | 0.450549451 | 1 | 0 |
| GNL1 | inf | 0.450549451 | 1 | 0 |
| GMFG | inf | 0.450549451 | 1 | 0 |
| FAM53B | inf | 0.450549451 | 1 | 0 |
| GABBR1 | inf | 0.450549451 | 1 | 0 |
| FCHSD2 | inf | 0.450549451 | 1 | 0 |
| FERMT1 | inf | 0.450549451 | 1 | 0 |
| SMIM3 | inf | 0.450549451 | 1 | 0 |
| GOLGA8G | inf | 0.450549451 | 1 | 0 |
| FSD1 | inf | 0.450549451 | 1 | 0 |
| GNG4 | inf | 0.450549451 | 1 | 0 |
| FSD1L | inf | 0.450549451 | 1 | 0 |
| SLC41A3 | inf | 0.450549451 | 1 | 0 |
| FAM49A | inf | 0.450549451 | 1 | 0 |
| GSTZ1 | inf | 0.450549451 | 1 | 0 |
| FSIP1 | inf | 0.450549451 | 1 | 0 |
| SFXN5 | inf | 0.450549451 | 1 | 0 |
| FFAR2 | inf | 0.450549451 | 1 | 0 |
| FAM47B | inf | 0.450549451 | 1 | 0 |
| SLC25A53 | inf | 0.450549451 | 1 | 0 |
| SERPINB6 | inf | 0.450549451 | 1 | 0 |
| SEMA4D | inf | 0.450549451 | 1 | 0 |
| SH3BP1 | inf | 0.450549451 | 1 | 0 |
| FOLH1B | inf | 0.450549451 | 1 | 0 |
| SLC35A5 | inf | 0.450549451 | 1 | 0 |
| GPR19 | inf | 0.450549451 | 1 | 0 |
| SESN3 | inf | 0.450549451 | 1 | 0 |
| SLITRK6 | inf | 0.450549451 | 1 | 0 |
| FLI1 | inf | 0.450549451 | 1 | 0 |
| SLC9A3 | inf | 0.450549451 | 1 | 0 |
| SLC35G5 | inf | 0.450549451 | 1 | 0 |
| GPR1 | inf | 0.450549451 | 1 | 0 |
| FBXO30 | inf | 0.450549451 | 1 | 0 |
| SIVA1 | inf | 0.450549451 | 1 | 0 |
| GEMIN2 | inf | 0.450549451 | 1 | 0 |
| SIRT7 | inf | 0.450549451 | 1 | 0 |
| SLC9A9 | inf | 0.450549451 | 1 | 0 |
| SERPINA7 | inf | 0.450549451 | 1 | 0 |
| FOLR1 | inf | 0.450549451 | 1 | 0 |
| SLMAP | inf | 0.450549451 | 1 | 0 |
| FKBP9 | inf | 0.450549451 | 1 | 0 |
| GINM1 | inf | 0.450549451 | 1 | 0 |
| SLC8A3 | inf | 0.450549451 | 1 | 0 |
| SLAMF6 | inf | 0.450549451 | 1 | 0 |
| SLC30A7 | inf | 0.450549451 | 1 | 0 |
| SLA2 | inf | 0.450549451 | 1 | 0 |
| SLC31A1 | inf | 0.450549451 | 1 | 0 |
| SERBP1 | inf | 0.450549451 | 1 | 0 |
| SERHL2 | inf | 0.450549451 | 1 | 0 |
| SIRPD | inf | 0.450549451 | 1 | 0 |
| FKBP8 | inf | 0.450549451 | 1 | 0 |
| FOXD2 | inf | 0.450549451 | 1 | 0 |
| FOXB1 | inf | 0.450549451 | 1 | 0 |
| SMAD4 | inf | 0.450549451 | 1 | 0 |
| GPR35 | inf | 0.450549451 | 1 | 0 |
| SLC35A1 | inf | 0.450549451 | 1 | 0 |
| GDI1 | inf | 0.450549451 | 1 | 0 |
| FOLR2 | inf | 0.450549451 | 1 | 0 |
| GPR55 | inf | 0.450549451 | 1 | 0 |
| FLOT2 | inf | 0.450549451 | 1 | 0 |
| GPR162 | inf | 0.450549451 | 1 | 0 |
| SERTAD1 | inf | 0.450549451 | 1 | 0 |
| SERPINB13 | inf | 0.450549451 | 1 | 0 |
| FNBP1 | inf | 0.450549451 | 1 | 0 |
| SLFN13 | inf | 0.450549451 | 1 | 0 |
| FMO5 | inf | 0.450549451 | 1 | 0 |
| SERPINB8 | inf | 0.450549451 | 1 | 0 |
| SLCO1C1 | inf | 0.450549451 | 1 | 0 |
| GH1 | inf | 0.450549451 | 1 | 0 |
| FNBP1L | inf | 0.450549451 | 1 | 0 |
| FN3K | inf | 0.450549451 | 1 | 0 |
| FMR1NB | inf | 0.450549451 | 1 | 0 |
| SERPINB7 | inf | 0.450549451 | 1 | 0 |
| SIGLEC14 | inf | 0.450549451 | 1 | 0 |
| FMR1 | inf | 0.450549451 | 1 | 0 |
| FBXL20 | inf | 0.450549451 | 1 | 0 |
| SIGLECL1 | inf | 0.450549451 | 1 | 0 |
| SIGLEC8 | inf | 0.450549451 | 1 | 0 |
| SIGMAR1 | inf | 0.450549451 | 1 | 0 |
| GCSAM | inf | 0.450549451 | 1 | 0 |
| FBLIM1 | inf | 0.450549451 | 1 | 0 |
| FATE1 | inf | 0.450549451 | 1 | 0 |
| GFM2 | inf | 0.450549451 | 1 | 0 |
| SERPINI2 | inf | 0.450549451 | 1 | 0 |
| SLC35B2 | inf | 0.450549451 | 1 | 0 |
| FLRT2 | inf | 0.450549451 | 1 | 0 |
| GPR160 | inf | 0.450549451 | 1 | 0 |
| SIAH3 | inf | 0.450549451 | 1 | 0 |
| SLC35F6 | inf | 0.450549451 | 1 | 0 |
| SLFNL1 | inf | 0.450549451 | 1 | 0 |
| SERPINH1 | inf | 0.450549451 | 1 | 0 |
| GGACT | inf | 0.450549451 | 1 | 0 |
| GGH | inf | 0.450549451 | 1 | 0 |
| SERPINB9 | inf | 0.450549451 | 1 | 0 |
| SERPINB11 | inf | 0.450549451 | 1 | 0 |
| SLC35E1 | inf | 0.450549451 | 1 | 0 |
| GHITM | inf | 0.450549451 | 1 | 0 |
| FNBP4 | inf | 0.450549451 | 1 | 0 |
| GFRA1 | inf | 0.450549451 | 1 | 0 |
| SLC8A1 | inf | 0.450549451 | 1 | 0 |
| FOXF2 | inf | 0.450549451 | 1 | 0 |
| SLC30A4 | inf | 0.450549451 | 1 | 0 |
| FAM83B | inf | 0.450549451 | 1 | 0 |
| SLC16A7 | inf | 0.450549451 | 1 | 0 |
| SETD9 | inf | 0.450549451 | 1 | 0 |
| FRAT2 | inf | 0.450549451 | 1 | 0 |
| SLC39A7 | inf | 0.450549451 | 1 | 0 |
| SEMA4A | inf | 0.450549451 | 1 | 0 |
| GPATCH11 | inf | 0.450549451 | 1 | 0 |
| FGF13 | inf | 0.450549451 | 1 | 0 |
| GPATCH2L | inf | 0.450549451 | 1 | 0 |
| GBP3 | inf | 0.450549451 | 1 | 0 |
| SLC39A2 | inf | 0.450549451 | 1 | 0 |
| FRA10AC1 | inf | 0.450549451 | 1 | 0 |
| FGFR1OP2 | inf | 0.450549451 | 1 | 0 |
| FPR3 | inf | 0.450549451 | 1 | 0 |
| GPX3 | inf | 0.450549451 | 1 | 0 |
| GPX1 | inf | 0.450549451 | 1 | 0 |
| GRAMD2B | inf | 0.450549451 | 1 | 0 |
| SLC14A1 | inf | 0.450549451 | 1 | 0 |
| GRIA2 | inf | 0.450549451 | 1 | 0 |
| COA7 | inf | 0.450549451 | 1 | 0 |
| GLRB | inf | 0.450549451 | 1 | 0 |
| SLC6A14 | inf | 0.450549451 | 1 | 0 |
| FAM71F1 | inf | 0.450549451 | 1 | 0 |
| FRMD4B | inf | 0.450549451 | 1 | 0 |
| GOT1L1 | inf | 0.450549451 | 1 | 0 |
| GART | inf | 0.450549451 | 1 | 0 |
| FRK | inf | 0.450549451 | 1 | 0 |
| SH3D21 | inf | 0.450549451 | 1 | 0 |
| GAS7 | inf | 0.450549451 | 1 | 0 |
| GLP2R | inf | 0.450549451 | 1 | 0 |
| MIEF1 | inf | 0.450549451 | 1 | 0 |
| FGF12 | inf | 0.450549451 | 1 | 0 |
| FAM81B | inf | 0.450549451 | 1 | 0 |
| GPA33 | inf | 0.450549451 | 1 | 0 |
| GATAD1 | inf | 0.450549451 | 1 | 0 |
| FGF18 | inf | 0.450549451 | 1 | 0 |
| GATA3 | inf | 0.450549451 | 1 | 0 |
| SF3B1 | inf | 0.450549451 | 1 | 0 |
| SLC13A5 | inf | 0.450549451 | 1 | 0 |
| FOXS1 | inf | 0.450549451 | 1 | 0 |
| FBXO6 | inf | 0.450549451 | 1 | 0 |
| SEPT4 | inf | 0.450549451 | 1 | 0 |
| FIBP | inf | 0.450549451 | 1 | 0 |
| SLC7A3 | inf | 0.450549451 | 1 | 0 |
| FBXO46 | inf | 0.450549451 | 1 | 0 |
| FIG4 | inf | 0.450549451 | 1 | 0 |
| SHISA6 | inf | 0.450549451 | 1 | 0 |
| GJA8 | inf | 0.450549451 | 1 | 0 |
| SERAC1 | inf | 0.450549451 | 1 | 0 |
| FIGNL1 | inf | 0.450549451 | 1 | 0 |
| GCNT7 | inf | 0.450549451 | 1 | 0 |
| GPR68 | inf | 0.450549451 | 1 | 0 |
| FITM2 | inf | 0.450549451 | 1 | 0 |
| FBXO4 | inf | 0.450549451 | 1 | 0 |
| FAM98C | inf | 0.450549451 | 1 | 0 |
| FKBP14 | inf | 0.450549451 | 1 | 0 |
| SMARCAD1 | inf | 0.450549451 | 1 | 0 |
| KMT5A | inf | 0.450549451 | 1 | 0 |
| GJD2 | inf | 0.450549451 | 1 | 0 |
| FBXW2 | inf | 0.450549451 | 1 | 0 |
| SENP3 | inf | 0.450549451 | 1 | 0 |
| SENP6 | inf | 0.450549451 | 1 | 0 |
| SLC39A14 | inf | 0.450549451 | 1 | 0 |
| FOXR1 | inf | 0.450549451 | 1 | 0 |
| SLC2A11 | inf | 0.450549451 | 1 | 0 |
| GPRC5A | inf | 0.450549451 | 1 | 0 |
| SETD4 | inf | 0.450549451 | 1 | 0 |
| SMARCD3 | inf | 0.450549451 | 1 | 0 |
| SLC6A4 | inf | 0.450549451 | 1 | 0 |
| GCLC | inf | 0.450549451 | 1 | 0 |
| SLC2A3 | inf | 0.450549451 | 1 | 0 |
| SETD3 | inf | 0.450549451 | 1 | 0 |
| HPLH1 | inf | 0.450549451 | 1 | 0 |
| SMARCE1 | inf | 0.450549451 | 1 | 0 |
| FHL5 | inf | 0.450549451 | 1 | 0 |
| GJB6 | inf | 0.450549451 | 1 | 0 |
| SHC3 | inf | 0.450549451 | 1 | 0 |
| SFTA3 | inf | 0.450549451 | 1 | 0 |
| KLK6 | inf | 0.450549451 | 1 | 0 |
| SCRT2 | inf | 0.450549451 | 1 | 0 |
| MED24 | inf | 0.450549451 | 1 | 0 |
| PIK3R1 | inf | 0.450549451 | 1 | 0 |
| MED27 | inf | 0.450549451 | 1 | 0 |
| PIK3CG | inf | 0.450549451 | 1 | 0 |
| PIGH | inf | 0.450549451 | 1 | 0 |
| MET | inf | 0.450549451 | 1 | 0 |
| METAP1D | inf | 0.450549451 | 1 | 0 |
| METRN | inf | 0.450549451 | 1 | 0 |
| METTL24 | inf | 0.450549451 | 1 | 0 |
| PI4K2B | inf | 0.450549451 | 1 | 0 |
| PI15 | inf | 0.450549451 | 1 | 0 |
| MED21 | inf | 0.450549451 | 1 | 0 |
| METTL9 | inf | 0.450549451 | 1 | 0 |
| PHOSPHO2 | inf | 0.450549451 | 1 | 0 |
| MFF | inf | 0.450549451 | 1 | 0 |
| MFSD1 | inf | 0.450549451 | 1 | 0 |
| MFSD6L | inf | 0.450549451 | 1 | 0 |
| MFSD7 | inf | 0.450549451 | 1 | 0 |
| MOGAT3 | inf | 0.450549451 | 1 | 0 |
| MGAT4B | inf | 0.450549451 | 1 | 0 |
| PHGR1 | inf | 0.450549451 | 1 | 0 |
| PHF8 | inf | 0.450549451 | 1 | 0 |
| MIA2 | inf | 0.450549451 | 1 | 0 |
| PHF21A | inf | 0.450549451 | 1 | 0 |
| MFAP4 | inf | 0.450549451 | 1 | 0 |
| MINK1 | inf | 0.450549451 | 1 | 0 |
| MED18 | inf | 0.450549451 | 1 | 0 |
| PINLYP | inf | 0.450549451 | 1 | 0 |
| MAPK9 | inf | 0.450549451 | 1 | 0 |
| MAPRE2 | inf | 0.450549451 | 1 | 0 |
| MARC1 | inf | 0.450549451 | 1 | 0 |
| PLAC4 | inf | 0.450549451 | 1 | 0 |
| MARCH3 | inf | 0.450549451 | 1 | 0 |
| MARCH8 | inf | 0.450549451 | 1 | 0 |
| PLA2G3 | inf | 0.450549451 | 1 | 0 |
| PLA2G16 | inf | 0.450549451 | 1 | 0 |
| MATN4 | inf | 0.450549451 | 1 | 0 |
| MBD3L2 | inf | 0.450549451 | 1 | 0 |
| PRKD2 | inf | 0.450549451 | 1 | 0 |
| PIN4 | inf | 0.450549451 | 1 | 0 |
| PKD1P1 | inf | 0.450549451 | 1 | 0 |
| PRG2 | inf | 0.450549451 | 1 | 0 |
| MC4R | inf | 0.450549451 | 1 | 0 |
| PIWIL1 | inf | 0.450549451 | 1 | 0 |
| PITX2 | inf | 0.450549451 | 1 | 0 |
| MCHR1 | inf | 0.450549451 | 1 | 0 |
| MCMBP | inf | 0.450549451 | 1 | 0 |
| PIR | inf | 0.450549451 | 1 | 0 |
| MDH2 | inf | 0.450549451 | 1 | 0 |
| PIP4K2B | inf | 0.450549451 | 1 | 0 |
| PCDHB16 | inf | 0.450549451 | 1 | 0 |
| PIP | inf | 0.450549451 | 1 | 0 |
| MBOAT7 | inf | 0.450549451 | 1 | 0 |
| PLAT | inf | 0.450549451 | 1 | 0 |
| PHF10 | inf | 0.450549451 | 1 | 0 |
| MIPEP | inf | 0.450549451 | 1 | 0 |
| MNDA | inf | 0.450549451 | 1 | 0 |
| MOAP1 | inf | 0.450549451 | 1 | 0 |
| PEX19 | inf | 0.450549451 | 1 | 0 |
| MOCS2 | inf | 0.450549451 | 1 | 0 |
| PEX10 | inf | 0.450549451 | 1 | 0 |
| PET117 | inf | 0.450549451 | 1 | 0 |
| PET100 | inf | 0.450549451 | 1 | 0 |
| MON1A | inf | 0.450549451 | 1 | 0 |
| MON1B | inf | 0.450549451 | 1 | 0 |
| MORC2 | inf | 0.450549451 | 1 | 0 |
| MORC3 | inf | 0.450549451 | 1 | 0 |
| MND1 | inf | 0.450549451 | 1 | 0 |
| PEG10 | inf | 0.450549451 | 1 | 0 |
| PEBP4 | inf | 0.450549451 | 1 | 0 |
| PEAR1 | inf | 0.450549451 | 1 | 0 |
| PDZRN3 | inf | 0.450549451 | 1 | 0 |
| MPP4 | inf | 0.450549451 | 1 | 0 |
| PDZD11 | inf | 0.450549451 | 1 | 0 |
| PDYN | inf | 0.450549451 | 1 | 0 |
| MPZL1 | inf | 0.450549451 | 1 | 0 |
| MRFAP1L1 | inf | 0.450549451 | 1 | 0 |
| MRGPRX2 | inf | 0.450549451 | 1 | 0 |
| PDRG1 | inf | 0.450549451 | 1 | 0 |
| MRO | inf | 0.450549451 | 1 | 0 |
| MPC1L | inf | 0.450549451 | 1 | 0 |
| TNPO1 | inf | 0.450549451 | 1 | 0 |
| MNAT1 | inf | 0.450549451 | 1 | 0 |
| PEX26 | inf | 0.450549451 | 1 | 0 |
| MIS12 | inf | 0.450549451 | 1 | 0 |
| PHC2 | inf | 0.450549451 | 1 | 0 |
| PHACTR4 | inf | 0.450549451 | 1 | 0 |
| MIXL1 | inf | 0.450549451 | 1 | 0 |
| NIFK | inf | 0.450549451 | 1 | 0 |
| PGPEP1L | inf | 0.450549451 | 1 | 0 |
| MKL2 | inf | 0.450549451 | 1 | 0 |
| MKNK2 | inf | 0.450549451 | 1 | 0 |
| MKS1 | inf | 0.450549451 | 1 | 0 |
| PGLYRP3 | inf | 0.450549451 | 1 | 0 |
| PGGT1B | inf | 0.450549451 | 1 | 0 |
| MMS22L | inf | 0.450549451 | 1 | 0 |
| PGF | inf | 0.450549451 | 1 | 0 |
| MLX | inf | 0.450549451 | 1 | 0 |
| MMAA | inf | 0.450549451 | 1 | 0 |
| PGA3 | inf | 0.450549451 | 1 | 0 |
| MMD | inf | 0.450549451 | 1 | 0 |
| MMGT1 | inf | 0.450549451 | 1 | 0 |
| PFN3 | inf | 0.450549451 | 1 | 0 |
| PFDN5 | inf | 0.450549451 | 1 | 0 |
| MMP19 | inf | 0.450549451 | 1 | 0 |
| PF4V1 | inf | 0.450549451 | 1 | 0 |
| MMP26 | inf | 0.450549451 | 1 | 0 |
| MMP7 | inf | 0.450549451 | 1 | 0 |
| MLLT3 | inf | 0.450549451 | 1 | 0 |
| MAPK8 | inf | 0.450549451 | 1 | 0 |
| MAPK14 | inf | 0.450549451 | 1 | 0 |
| MAPK13 | inf | 0.450549451 | 1 | 0 |
| LOC286238 | inf | 0.450549451 | 1 | 0 |
| LOC388282 | inf | 0.450549451 | 1 | 0 |
| LOC554223 | inf | 0.450549451 | 1 | 0 |
| LOC595101 | inf | 0.450549451 | 1 | 0 |
| PLPP5 | inf | 0.450549451 | 1 | 0 |
| LOC644086 | inf | 0.450549451 | 1 | 0 |
| LOC645202 | inf | 0.450549451 | 1 | 0 |
| PLPP3 | inf | 0.450549451 | 1 | 0 |
| LOC728763 | inf | 0.450549451 | 1 | 0 |
| PPAN-P2RY11 | inf | 0.450549451 | 1 | 0 |
| LOC730159 | inf | 0.450549451 | 1 | 0 |
| PPIAL4D | inf | 0.450549451 | 1 | 0 |
| POU6F1 | inf | 0.450549451 | 1 | 0 |
| POU5F1 | inf | 0.450549451 | 1 | 0 |
| LOXL2 | inf | 0.450549451 | 1 | 0 |
| POU2F1 | inf | 0.450549451 | 1 | 0 |
| LPAR3 | inf | 0.450549451 | 1 | 0 |
| LPGAT1 | inf | 0.450549451 | 1 | 0 |
| LRCH3 | inf | 0.450549451 | 1 | 0 |
| POT1 | inf | 0.450549451 | 1 | 0 |
| LRG1 | inf | 0.450549451 | 1 | 0 |
| PYDC1 | inf | 0.450549451 | 1 | 0 |
| POLR3F | inf | 0.450549451 | 1 | 0 |
| POLR2B | inf | 0.450549451 | 1 | 0 |
| BORCS5 | inf | 0.450549451 | 1 | 0 |
| POLR1C | inf | 0.450549451 | 1 | 0 |
| LOC149684 | inf | 0.450549451 | 1 | 0 |
| LOC101929708 | inf | 0.450549451 | 1 | 0 |
| PTPA | inf | 0.450549451 | 1 | 0 |
| LOC101927180 | inf | 0.450549451 | 1 | 0 |
| PPP1R3C | inf | 0.450549451 | 1 | 0 |
| PPP1R36 | inf | 0.450549451 | 1 | 0 |
| LOC101927742 | inf | 0.450549451 | 1 | 0 |
| LOC101927939 | inf | 0.450549451 | 1 | 0 |
| PPP1R1B | inf | 0.450549451 | 1 | 0 |
| LOC101928147 | inf | 0.450549451 | 1 | 0 |
| PPP1R14C | inf | 0.450549451 | 1 | 0 |
| PPP1R12C | inf | 0.450549451 | 1 | 0 |
| LOC101928585 | inf | 0.450549451 | 1 | 0 |
| LOC147646 | inf | 0.450549451 | 1 | 0 |
| LOC101928621 | inf | 0.450549451 | 1 | 0 |
| LOC101928749 | inf | 0.450549451 | 1 | 0 |
| LOC101928757 | inf | 0.450549451 | 1 | 0 |
| PPM1N | inf | 0.450549451 | 1 | 0 |
| LOC101928927 | inf | 0.450549451 | 1 | 0 |
| PPM1K | inf | 0.450549451 | 1 | 0 |
| PPM1J | inf | 0.450549451 | 1 | 0 |
| LOC101929186 | inf | 0.450549451 | 1 | 0 |
| LOC101929300 | inf | 0.450549451 | 1 | 0 |
| LOC101929303 | inf | 0.450549451 | 1 | 0 |
| PPIH | inf | 0.450549451 | 1 | 0 |
| PPIF | inf | 0.450549451 | 1 | 0 |
| LOC101928728 | inf | 0.450549451 | 1 | 0 |
| LRRC2 | inf | 0.450549451 | 1 | 0 |
| LRRC20 | inf | 0.450549451 | 1 | 0 |
| LRRC29 | inf | 0.450549451 | 1 | 0 |
| LYPD1 | inf | 0.450549451 | 1 | 0 |
| LZTS3 | inf | 0.450549451 | 1 | 0 |
| M6PR | inf | 0.450549451 | 1 | 0 |
| PLVAP | inf | 0.450549451 | 1 | 0 |
| PLSCR5 | inf | 0.450549451 | 1 | 0 |
| PLSCR4 | inf | 0.450549451 | 1 | 0 |
| PLSCR3 | inf | 0.450549451 | 1 | 0 |
| MAD2L1BP | inf | 0.450549451 | 1 | 0 |
| MAEA | inf | 0.450549451 | 1 | 0 |
| PLK2 | inf | 0.450549451 | 1 | 0 |
| MAGEB3 | inf | 0.450549451 | 1 | 0 |
| LYNX1 | inf | 0.450549451 | 1 | 0 |
| PLEKHJ1 | inf | 0.450549451 | 1 | 0 |
| PLEKHG7 | inf | 0.450549451 | 1 | 0 |
| MKL1 | inf | 0.450549451 | 1 | 0 |
| MAL2 | inf | 0.450549451 | 1 | 0 |
| MAMLD1 | inf | 0.450549451 | 1 | 0 |
| PLEKHF1 | inf | 0.450549451 | 1 | 0 |
| MANBA | inf | 0.450549451 | 1 | 0 |
| PLEKHB1 | inf | 0.450549451 | 1 | 0 |
| MAP1LC3B | inf | 0.450549451 | 1 | 0 |
| MAP1LC3C | inf | 0.450549451 | 1 | 0 |
| MAP3K3 | inf | 0.450549451 | 1 | 0 |
| MAP4K5 | inf | 0.450549451 | 1 | 0 |
| PLEKHH2 | inf | 0.450549451 | 1 | 0 |
| PMS2P4 | inf | 0.450549451 | 1 | 0 |
| LY6G6F | inf | 0.450549451 | 1 | 0 |
| LUZP1 | inf | 0.450549451 | 1 | 0 |
| LRRC36 | inf | 0.450549451 | 1 | 0 |
| LRRC37A4P | inf | 0.450549451 | 1 | 0 |
| LRRC38 | inf | 0.450549451 | 1 | 0 |
| POLDIP3 | inf | 0.450549451 | 1 | 0 |
| LRRC46 | inf | 0.450549451 | 1 | 0 |
| POLDIP2 | inf | 0.450549451 | 1 | 0 |
| LRRC49 | inf | 0.450549451 | 1 | 0 |
| LRRC4B | inf | 0.450549451 | 1 | 0 |
| LRRC52 | inf | 0.450549451 | 1 | 0 |
| LRRC55 | inf | 0.450549451 | 1 | 0 |
| POLA2 | inf | 0.450549451 | 1 | 0 |
| POLA1 | inf | 0.450549451 | 1 | 0 |
| LRRC72 | inf | 0.450549451 | 1 | 0 |
| LRRC8B | inf | 0.450549451 | 1 | 0 |
| LRRCC1 | inf | 0.450549451 | 1 | 0 |
| LRRFIP1 | inf | 0.450549451 | 1 | 0 |
| LRRTM3 | inf | 0.450549451 | 1 | 0 |
| PNPO | inf | 0.450549451 | 1 | 0 |
| PNPLA8 | inf | 0.450549451 | 1 | 0 |
| LSM1 | inf | 0.450549451 | 1 | 0 |
| LSM14A | inf | 0.450549451 | 1 | 0 |
| LSM3 | inf | 0.450549451 | 1 | 0 |
| LTA4H | inf | 0.450549451 | 1 | 0 |
| PNMA8B | inf | 0.450549451 | 1 | 0 |
| PNLIP | inf | 0.450549451 | 1 | 0 |
| PLPP6 | inf | 0.450549451 | 1 | 0 |
| MRPL57 | inf | 0.450549451 | 1 | 0 |
| MRPL11 | inf | 0.450549451 | 1 | 0 |
| MRPL3 | inf | 0.450549451 | 1 | 0 |
| NONO | inf | 0.450549451 | 1 | 0 |
| OR52E2 | inf | 0.450549451 | 1 | 0 |
| NOP56 | inf | 0.450549451 | 1 | 0 |
| NOP58 | inf | 0.450549451 | 1 | 0 |
| NOS1AP | inf | 0.450549451 | 1 | 0 |
| NOSTRIN | inf | 0.450549451 | 1 | 0 |
| OR52B4 | inf | 0.450549451 | 1 | 0 |
| OR52B2 | inf | 0.450549451 | 1 | 0 |
| NOXA1 | inf | 0.450549451 | 1 | 0 |
| OR51Q1 | inf | 0.450549451 | 1 | 0 |
| NPAS4 | inf | 0.450549451 | 1 | 0 |
| NODAL | inf | 0.450549451 | 1 | 0 |
| NPBWR1 | inf | 0.450549451 | 1 | 0 |
| OR51L1 | inf | 0.450549451 | 1 | 0 |
| OR51F1 | inf | 0.450549451 | 1 | 0 |
| OR51D1 | inf | 0.450549451 | 1 | 0 |
| OR51B5 | inf | 0.450549451 | 1 | 0 |
| OR4X2 | inf | 0.450549451 | 1 | 0 |
| OR4Q3 | inf | 0.450549451 | 1 | 0 |
| NPRL3 | inf | 0.450549451 | 1 | 0 |
| NPVF | inf | 0.450549451 | 1 | 0 |
| OR4K5 | inf | 0.450549451 | 1 | 0 |
| OR4K17 | inf | 0.450549451 | 1 | 0 |
| OR4K15 | inf | 0.450549451 | 1 | 0 |
| NPBWR2 | inf | 0.450549451 | 1 | 0 |
| NR1H3 | inf | 0.450549451 | 1 | 0 |
| OR52N2 | inf | 0.450549451 | 1 | 0 |
| NOC2L | inf | 0.450549451 | 1 | 0 |
| OR6C68 | inf | 0.450549451 | 1 | 0 |
| NHLRC1 | inf | 0.450549451 | 1 | 0 |
| OR5V1 | inf | 0.450549451 | 1 | 0 |
| NINJ2 | inf | 0.450549451 | 1 | 0 |
| OR5M9 | inf | 0.450549451 | 1 | 0 |
| OR5M8 | inf | 0.450549451 | 1 | 0 |
| NIPAL3 | inf | 0.450549451 | 1 | 0 |
| OR5M3 | inf | 0.450549451 | 1 | 0 |
| NIT1 | inf | 0.450549451 | 1 | 0 |
| NKAIN2 | inf | 0.450549451 | 1 | 0 |
| NKIRAS2 | inf | 0.450549451 | 1 | 0 |
| NOC3L | inf | 0.450549451 | 1 | 0 |
| NKX2-4 | inf | 0.450549451 | 1 | 0 |
| OR5J2 | inf | 0.450549451 | 1 | 0 |
| OR5I1 | inf | 0.450549451 | 1 | 0 |
| NKX6-3 | inf | 0.450549451 | 1 | 0 |
| NLGN1 | inf | 0.450549451 | 1 | 0 |
| NLGN2 | inf | 0.450549451 | 1 | 0 |
| OR5AC2 | inf | 0.450549451 | 1 | 0 |
| NME7 | inf | 0.450549451 | 1 | 0 |
| OR56B4 | inf | 0.450549451 | 1 | 0 |
| OR52R1 | inf | 0.450549451 | 1 | 0 |
| NMT1 | inf | 0.450549451 | 1 | 0 |
| OR52N4 | inf | 0.450549451 | 1 | 0 |
| OR5K2 | inf | 0.450549451 | 1 | 0 |
| NR2C2 | inf | 0.450549451 | 1 | 0 |
| OR4D10 | inf | 0.450549451 | 1 | 0 |
| OR4D1 | inf | 0.450549451 | 1 | 0 |
| OR2AG1 | inf | 0.450549451 | 1 | 0 |
| NUP50 | inf | 0.450549451 | 1 | 0 |
| NUP54 | inf | 0.450549451 | 1 | 0 |
| NUS1 | inf | 0.450549451 | 1 | 0 |
| OR1N2 | inf | 0.450549451 | 1 | 0 |
| OR1N1 | inf | 0.450549451 | 1 | 0 |
| OR1G1 | inf | 0.450549451 | 1 | 0 |
| NXPE2 | inf | 0.450549451 | 1 | 0 |
| OR1E1 | inf | 0.450549451 | 1 | 0 |
| OR1D2 | inf | 0.450549451 | 1 | 0 |
| OAF | inf | 0.450549451 | 1 | 0 |
| OR2B11 | inf | 0.450549451 | 1 | 0 |
| OR13G1 | inf | 0.450549451 | 1 | 0 |
| OCLN | inf | 0.450549451 | 1 | 0 |
| OCM2 | inf | 0.450549451 | 1 | 0 |
| ODF1 | inf | 0.450549451 | 1 | 0 |
| OR12D3 | inf | 0.450549451 | 1 | 0 |
| OGFRL1 | inf | 0.450549451 | 1 | 0 |
| OR10W1 | inf | 0.450549451 | 1 | 0 |
| OR10V1 | inf | 0.450549451 | 1 | 0 |
| OR10J1 | inf | 0.450549451 | 1 | 0 |
| OPN1SW | inf | 0.450549451 | 1 | 0 |
| OR10G9 | inf | 0.450549451 | 1 | 0 |
| OR10G4 | inf | 0.450549451 | 1 | 0 |
| STN1 | inf | 0.450549451 | 1 | 0 |
| NUFIP2 | inf | 0.450549451 | 1 | 0 |
| OR2B3 | inf | 0.450549451 | 1 | 0 |
| OR2B6 | inf | 0.450549451 | 1 | 0 |
| OR4C3 | inf | 0.450549451 | 1 | 0 |
| OR4A5 | inf | 0.450549451 | 1 | 0 |
| NRG4 | inf | 0.450549451 | 1 | 0 |
| OR2Z1 | inf | 0.450549451 | 1 | 0 |
| NRN1L | inf | 0.450549451 | 1 | 0 |
| NRSN2 | inf | 0.450549451 | 1 | 0 |
| NSA2 | inf | 0.450549451 | 1 | 0 |
| NSG1 | inf | 0.450549451 | 1 | 0 |
| NSRP1 | inf | 0.450549451 | 1 | 0 |
| NSUN2 | inf | 0.450549451 | 1 | 0 |
| NT5C3B | inf | 0.450549451 | 1 | 0 |
| NT5M | inf | 0.450549451 | 1 | 0 |
| NTMT1 | inf | 0.450549451 | 1 | 0 |
| NTNG2 | inf | 0.450549451 | 1 | 0 |
| NTRK2 | inf | 0.450549451 | 1 | 0 |
| OR2M5 | inf | 0.450549451 | 1 | 0 |
| OR2M3 | inf | 0.450549451 | 1 | 0 |
| NUBP1 | inf | 0.450549451 | 1 | 0 |
| OR2J3 | inf | 0.450549451 | 1 | 0 |
| OR2J2 | inf | 0.450549451 | 1 | 0 |
| NUDCD1 | inf | 0.450549451 | 1 | 0 |
| OR2D2 | inf | 0.450549451 | 1 | 0 |
| NUDT4 | inf | 0.450549451 | 1 | 0 |
| OR2C1 | inf | 0.450549451 | 1 | 0 |
| NUDT8 | inf | 0.450549451 | 1 | 0 |
| OR6C70 | inf | 0.450549451 | 1 | 0 |
| LOC100996646 | inf | 0.450549451 | 1 | 0 |
| OR6K2 | inf | 0.450549451 | 1 | 0 |
| NFS1 | inf | 0.450549451 | 1 | 0 |
| MUC5AC | inf | 0.450549451 | 1 | 0 |
| PCDHB4 | inf | 0.450549451 | 1 | 0 |
| MUCL1 | inf | 0.450549451 | 1 | 0 |
| MUL1 | inf | 0.450549451 | 1 | 0 |
| MUM1L1 | inf | 0.450549451 | 1 | 0 |
| MVK | inf | 0.450549451 | 1 | 0 |
| PCDHB1 | inf | 0.450549451 | 1 | 0 |
| MXD4 | inf | 0.450549451 | 1 | 0 |
| MXRA8 | inf | 0.450549451 | 1 | 0 |
| MYBPHL | inf | 0.450549451 | 1 | 0 |
| MYD88 | inf | 0.450549451 | 1 | 0 |
| MTX3 | inf | 0.450549451 | 1 | 0 |
| MYF6 | inf | 0.450549451 | 1 | 0 |
| PAXIP1 | inf | 0.450549451 | 1 | 0 |
| PATE3 | inf | 0.450549451 | 1 | 0 |
| PATE2 | inf | 0.450549451 | 1 | 0 |
| MYOT | inf | 0.450549451 | 1 | 0 |
| PARP12 | inf | 0.450549451 | 1 | 0 |
| PARP1 | inf | 0.450549451 | 1 | 0 |
| MZT2A | inf | 0.450549451 | 1 | 0 |
| N4BP2L1 | inf | 0.450549451 | 1 | 0 |
| N4BP2L2 | inf | 0.450549451 | 1 | 0 |
| PAQR8 | inf | 0.450549451 | 1 | 0 |
| PAQR4 | inf | 0.450549451 | 1 | 0 |
| MYL3 | inf | 0.450549451 | 1 | 0 |
| NAA30 | inf | 0.450549451 | 1 | 0 |
| MTRNR2L2 | inf | 0.450549451 | 1 | 0 |
| MTNR1A | inf | 0.450549451 | 1 | 0 |
| PDHB | inf | 0.450549451 | 1 | 0 |
| MRPL40 | inf | 0.450549451 | 1 | 0 |
| PDGFRA | inf | 0.450549451 | 1 | 0 |
| MRPL50 | inf | 0.450549451 | 1 | 0 |
| MRPL52 | inf | 0.450549451 | 1 | 0 |
| PDE8A | inf | 0.450549451 | 1 | 0 |
| PDE6D | inf | 0.450549451 | 1 | 0 |
| MRPS30 | inf | 0.450549451 | 1 | 0 |
| MRPS33 | inf | 0.450549451 | 1 | 0 |
| PDCL | inf | 0.450549451 | 1 | 0 |
| MS4A10 | inf | 0.450549451 | 1 | 0 |
| PCDHGA4 | inf | 0.450549451 | 1 | 0 |
| PDCD4 | inf | 0.450549451 | 1 | 0 |
| MS4A8 | inf | 0.450549451 | 1 | 0 |
| PCP4 | inf | 0.450549451 | 1 | 0 |
| MSI2 | inf | 0.450549451 | 1 | 0 |
| MSMB | inf | 0.450549451 | 1 | 0 |
| MT2A | inf | 0.450549451 | 1 | 0 |
| PCGF5 | inf | 0.450549451 | 1 | 0 |
| PCGF3 | inf | 0.450549451 | 1 | 0 |
| MTHFD2 | inf | 0.450549451 | 1 | 0 |
| MTM1 | inf | 0.450549451 | 1 | 0 |
| PCDHGA9 | inf | 0.450549451 | 1 | 0 |
| MTMR6 | inf | 0.450549451 | 1 | 0 |
| MS4A18 | inf | 0.450549451 | 1 | 0 |
| NAA40 | inf | 0.450549451 | 1 | 0 |
| PANK4 | inf | 0.450549451 | 1 | 0 |
| NACA | inf | 0.450549451 | 1 | 0 |
| OTUD6B | inf | 0.450549451 | 1 | 0 |
| OTUD4 | inf | 0.450549451 | 1 | 0 |
| NDUFS7 | inf | 0.450549451 | 1 | 0 |
| OTOL1 | inf | 0.450549451 | 1 | 0 |
| OSTM1 | inf | 0.450549451 | 1 | 0 |
| OST4 | inf | 0.450549451 | 1 | 0 |
| OSGEPL1 | inf | 0.450549451 | 1 | 0 |
| OSCAR | inf | 0.450549451 | 1 | 0 |
| NEK7 | inf | 0.450549451 | 1 | 0 |
| ORAOV1 | inf | 0.450549451 | 1 | 0 |
| NETO2 | inf | 0.450549451 | 1 | 0 |
| NDUFB7 | inf | 0.450549451 | 1 | 0 |
| OR9A2 | inf | 0.450549451 | 1 | 0 |
| OR8K1 | inf | 0.450549451 | 1 | 0 |
| OR8H1 | inf | 0.450549451 | 1 | 0 |
| OR8B4 | inf | 0.450549451 | 1 | 0 |
| NFIA | inf | 0.450549451 | 1 | 0 |
| NFIC | inf | 0.450549451 | 1 | 0 |
| OR7A17 | inf | 0.450549451 | 1 | 0 |
| NFKB1 | inf | 0.450549451 | 1 | 0 |
| OR6V1 | inf | 0.450549451 | 1 | 0 |
| OR6Q1 | inf | 0.450549451 | 1 | 0 |
| NFKBID | inf | 0.450549451 | 1 | 0 |
| OR6P1 | inf | 0.450549451 | 1 | 0 |
| NEXN | inf | 0.450549451 | 1 | 0 |
| NDUFB10 | inf | 0.450549451 | 1 | 0 |
| OVOL1 | inf | 0.450549451 | 1 | 0 |
| NDUFAF1 | inf | 0.450549451 | 1 | 0 |
| RDH14 | inf | 0.450549451 | 1 | 0 |
| TNIP1 | inf | 0.450549451 | 1 | 0 |
| NAP1L3 | inf | 0.450549451 | 1 | 0 |
| NAPSA | inf | 0.450549451 | 1 | 0 |
| RNF138 | inf | 0.450549451 | 1 | 0 |
| NARFL | inf | 0.450549451 | 1 | 0 |
| NASP | inf | 0.450549451 | 1 | 0 |
| NAT8L | inf | 0.450549451 | 1 | 0 |
| PABPC4 | inf | 0.450549451 | 1 | 0 |
| PABPC1L | inf | 0.450549451 | 1 | 0 |
| NCAPH2 | inf | 0.450549451 | 1 | 0 |
| NCEH1 | inf | 0.450549451 | 1 | 0 |
| NCK2 | inf | 0.450549451 | 1 | 0 |
| P2RY4 | inf | 0.450549451 | 1 | 0 |
| P2RY13 | inf | 0.450549451 | 1 | 0 |
| P2RY12 | inf | 0.450549451 | 1 | 0 |
| OXT | inf | 0.450549451 | 1 | 0 |
| NSMCE3 | inf | 0.450549451 | 1 | 0 |
| NDRG1 | inf | 0.450549451 | 1 | 0 |
| NDRG3 | inf | 0.450549451 | 1 | 0 |
| NDRG4 | inf | 0.450549451 | 1 | 0 |
| NDUFA11 | inf | 0.450549451 | 1 | 0 |
| NDUFA2 | inf | 0.450549451 | 1 | 0 |
| NDUFA7 | inf | 0.450549451 | 1 | 0 |
| OVOS2 | inf | 0.450549451 | 1 | 0 |
| NGEF | inf | 0.450549451 | 1 | 0 |
| PPP5C | inf | 0.450549451 | 1 | 0 |
| LOC100653515 | inf | 0.450549451 | 1 | 0 |
| PPP6R2 | inf | 0.450549451 | 1 | 0 |
| IFNGR2 | inf | 0.450549451 | 1 | 0 |
| ROM1 | inf | 0.450549451 | 1 | 0 |
| IGBP1 | inf | 0.450549451 | 1 | 0 |
| IGF1 | inf | 0.450549451 | 1 | 0 |
| RNPC3 | inf | 0.450549451 | 1 | 0 |
| IGF2 | inf | 0.450549451 | 1 | 0 |
| IGF2BP2 | inf | 0.450549451 | 1 | 0 |
| IGFBP3 | inf | 0.450549451 | 1 | 0 |
| MRM3 | inf | 0.450549451 | 1 | 0 |
| IGFBP7 | inf | 0.450549451 | 1 | 0 |
| IGFL4 | inf | 0.450549451 | 1 | 0 |
| IFNA8 | inf | 0.450549451 | 1 | 0 |
| IGHJ1P | inf | 0.450549451 | 1 | 0 |
| RNFT2 | inf | 0.450549451 | 1 | 0 |
| RNF32 | inf | 0.450549451 | 1 | 0 |
| IGHV3-35 | inf | 0.450549451 | 1 | 0 |
| RNF26 | inf | 0.450549451 | 1 | 0 |
| IGHV3OR16-8 | inf | 0.450549451 | 1 | 0 |
| IGKV1-8 | inf | 0.450549451 | 1 | 0 |
| IGKV1D-37 | inf | 0.450549451 | 1 | 0 |
| IGKV1D-42 | inf | 0.450549451 | 1 | 0 |
| IGLC2 | inf | 0.450549451 | 1 | 0 |
| RNF139 | inf | 0.450549451 | 1 | 0 |
| IGLL5 | inf | 0.450549451 | 1 | 0 |
| IGHM | inf | 0.450549451 | 1 | 0 |
| RNF135 | inf | 0.450549451 | 1 | 0 |
| IFNA7 | inf | 0.450549451 | 1 | 0 |
| RORB | inf | 0.450549451 | 1 | 0 |
| HTR3C | inf | 0.450549451 | 1 | 0 |
| RPL35A | inf | 0.450549451 | 1 | 0 |
| RPL3 | inf | 0.450549451 | 1 | 0 |
| RPL28 | inf | 0.450549451 | 1 | 0 |
| HTR6 | inf | 0.450549451 | 1 | 0 |
| HYAL3 | inf | 0.450549451 | 1 | 0 |
| HYAL4 | inf | 0.450549451 | 1 | 0 |
| HYKK | inf | 0.450549451 | 1 | 0 |
| RPL11 | inf | 0.450549451 | 1 | 0 |
| HYOU1 | inf | 0.450549451 | 1 | 0 |
| IAPP | inf | 0.450549451 | 1 | 0 |
| IFNA6 | inf | 0.450549451 | 1 | 0 |
| IBSP | inf | 0.450549451 | 1 | 0 |
| IDH2 | inf | 0.450549451 | 1 | 0 |
| RPE | inf | 0.450549451 | 1 | 0 |
| IDH3G | inf | 0.450549451 | 1 | 0 |
| IDI2 | inf | 0.450549451 | 1 | 0 |
| RPAP3 | inf | 0.450549451 | 1 | 0 |
| RPA2 | inf | 0.450549451 | 1 | 0 |
| STK19B | inf | 0.450549451 | 1 | 0 |
| IFIT1 | inf | 0.450549451 | 1 | 0 |
| IFIT5 | inf | 0.450549451 | 1 | 0 |
| IFITM3 | inf | 0.450549451 | 1 | 0 |
| IFNA14 | inf | 0.450549451 | 1 | 0 |
| IDE | inf | 0.450549451 | 1 | 0 |
| IGLV2-33 | inf | 0.450549451 | 1 | 0 |
| IGLV3-9 | inf | 0.450549451 | 1 | 0 |
| IGLV4-3 | inf | 0.450549451 | 1 | 0 |
| ING1 | inf | 0.450549451 | 1 | 0 |
| RHOJ | inf | 0.450549451 | 1 | 0 |
| INHA | inf | 0.450549451 | 1 | 0 |
| RHO | inf | 0.450549451 | 1 | 0 |
| INO80 | inf | 0.450549451 | 1 | 0 |
| RHBG | inf | 0.450549451 | 1 | 0 |
| INPP5A | inf | 0.450549451 | 1 | 0 |
| PMPCA | inf | 0.450549451 | 1 | 0 |
| RHAG | inf | 0.450549451 | 1 | 0 |
| INPP5K | inf | 0.450549451 | 1 | 0 |
| RGS2 | inf | 0.450549451 | 1 | 0 |
| IMPG1 | inf | 0.450549451 | 1 | 0 |
| RGS18 | inf | 0.450549451 | 1 | 0 |
| RGMA | inf | 0.450549451 | 1 | 0 |
| IPPK | inf | 0.450549451 | 1 | 0 |
| IQCF1 | inf | 0.450549451 | 1 | 0 |
| IQCJ | inf | 0.450549451 | 1 | 0 |
| IQCK | inf | 0.450549451 | 1 | 0 |
| RFWD2 | inf | 0.450549451 | 1 | 0 |
| IRF2 | inf | 0.450549451 | 1 | 0 |
| IRF2BP2 | inf | 0.450549451 | 1 | 0 |
| RFTN2 | inf | 0.450549451 | 1 | 0 |
| RFPL4AL1 | inf | 0.450549451 | 1 | 0 |
| IRF9 | inf | 0.450549451 | 1 | 0 |
| INTS9 | inf | 0.450549451 | 1 | 0 |
| RHOXF2 | inf | 0.450549451 | 1 | 0 |
| ILK | inf | 0.450549451 | 1 | 0 |
| ILDR1 | inf | 0.450549451 | 1 | 0 |
| IGLV4-69 | inf | 0.450549451 | 1 | 0 |
| IGLV5-52 | inf | 0.450549451 | 1 | 0 |
| IGSF11 | inf | 0.450549451 | 1 | 0 |
| RNF112 | inf | 0.450549451 | 1 | 0 |
| RNASEL | inf | 0.450549451 | 1 | 0 |
| IKBIP | inf | 0.450549451 | 1 | 0 |
| RNASEH2B | inf | 0.450549451 | 1 | 0 |
| IL11 | inf | 0.450549451 | 1 | 0 |
| RNASE1 | inf | 0.450549451 | 1 | 0 |
| RMND5A | inf | 0.450549451 | 1 | 0 |
| RMI2 | inf | 0.450549451 | 1 | 0 |
| IL15 | inf | 0.450549451 | 1 | 0 |
| IL17B | inf | 0.450549451 | 1 | 0 |
| RLN2 | inf | 0.450549451 | 1 | 0 |
| IL17RE | inf | 0.450549451 | 1 | 0 |
| IL17REL | inf | 0.450549451 | 1 | 0 |
| IL18BP | inf | 0.450549451 | 1 | 0 |
| RIPPLY1 | inf | 0.450549451 | 1 | 0 |
| RIPK2 | inf | 0.450549451 | 1 | 0 |
| IL1R1 | inf | 0.450549451 | 1 | 0 |
| IL1RAP | inf | 0.450549451 | 1 | 0 |
| RIMS4 | inf | 0.450549451 | 1 | 0 |
| IL23R | inf | 0.450549451 | 1 | 0 |
| RIMKLB | inf | 0.450549451 | 1 | 0 |
| IL2RG | inf | 0.450549451 | 1 | 0 |
| RPL36AL | inf | 0.450549451 | 1 | 0 |
| IRGC | inf | 0.450549451 | 1 | 0 |
| HTR1F | inf | 0.450549451 | 1 | 0 |
| HTATIP2 | inf | 0.450549451 | 1 | 0 |
| SCAMP4 | inf | 0.450549451 | 1 | 0 |
| PUDP | inf | 0.450549451 | 1 | 0 |
| SCAMP3 | inf | 0.450549451 | 1 | 0 |
| SC5D | inf | 0.450549451 | 1 | 0 |
| SATB1 | inf | 0.450549451 | 1 | 0 |
| HEMGN | inf | 0.450549451 | 1 | 0 |
| SARS2 | inf | 0.450549451 | 1 | 0 |
| HEPACAM | inf | 0.450549451 | 1 | 0 |
| SAP30L | inf | 0.450549451 | 1 | 0 |
| HES2 | inf | 0.450549451 | 1 | 0 |
| SAMSN1 | inf | 0.450549451 | 1 | 0 |
| HDDC2 | inf | 0.450549451 | 1 | 0 |
| HEXA | inf | 0.450549451 | 1 | 0 |
| HEXIM2 | inf | 0.450549451 | 1 | 0 |
| HHAT | inf | 0.450549451 | 1 | 0 |
| HHEX | inf | 0.450549451 | 1 | 0 |
| HIBCH | inf | 0.450549451 | 1 | 0 |
| HINFP | inf | 0.450549451 | 1 | 0 |
| HINT1 | inf | 0.450549451 | 1 | 0 |
| SAMD12 | inf | 0.450549451 | 1 | 0 |
| HIST1H1B | inf | 0.450549451 | 1 | 0 |
| SALL2 | inf | 0.450549451 | 1 | 0 |
| HIST1H3E | inf | 0.450549451 | 1 | 0 |
| HIST1H4B | inf | 0.450549451 | 1 | 0 |
| HEXB | inf | 0.450549451 | 1 | 0 |
| HIST1H4H | inf | 0.450549451 | 1 | 0 |
| HDAC6 | inf | 0.450549451 | 1 | 0 |
| HCN2 | inf | 0.450549451 | 1 | 0 |
| GTSF1 | inf | 0.450549451 | 1 | 0 |
| GUCD1 | inf | 0.450549451 | 1 | 0 |
| SCPEP1 | inf | 0.450549451 | 1 | 0 |
| SCNN1G | inf | 0.450549451 | 1 | 0 |
| GUCY2EP | inf | 0.450549451 | 1 | 0 |
| GXYLT2 | inf | 0.450549451 | 1 | 0 |
| GYPC | inf | 0.450549451 | 1 | 0 |
| SCN4B | inf | 0.450549451 | 1 | 0 |
| GZMB | inf | 0.450549451 | 1 | 0 |
| GZMM | inf | 0.450549451 | 1 | 0 |
| H1FNT | inf | 0.450549451 | 1 | 0 |
| HDAC1 | inf | 0.450549451 | 1 | 0 |
| H2AFV | inf | 0.450549451 | 1 | 0 |
| SCN1B | inf | 0.450549451 | 1 | 0 |
| HAND1 | inf | 0.450549451 | 1 | 0 |
| SCMH1 | inf | 0.450549451 | 1 | 0 |
| HAPLN2 | inf | 0.450549451 | 1 | 0 |
| HAPLN4 | inf | 0.450549451 | 1 | 0 |
| HARS2 | inf | 0.450549451 | 1 | 0 |
| SCLT1 | inf | 0.450549451 | 1 | 0 |
| HAUS2 | inf | 0.450549451 | 1 | 0 |
| HAUS8 | inf | 0.450549451 | 1 | 0 |
| SCGB1C1 | inf | 0.450549451 | 1 | 0 |
| SCG5 | inf | 0.450549451 | 1 | 0 |
| H2AFY2 | inf | 0.450549451 | 1 | 0 |
| S100A9 | inf | 0.450549451 | 1 | 0 |
| S100A7L2 | inf | 0.450549451 | 1 | 0 |
| HLA-DOA | inf | 0.450549451 | 1 | 0 |
| HS6ST2 | inf | 0.450549451 | 1 | 0 |
| RPSA | inf | 0.450549451 | 1 | 0 |
| HSD11B1L | inf | 0.450549451 | 1 | 0 |
| HSD17B11 | inf | 0.450549451 | 1 | 0 |
| HSD17B6 | inf | 0.450549451 | 1 | 0 |
| HSD17B7P2 | inf | 0.450549451 | 1 | 0 |
| HSDL1 | inf | 0.450549451 | 1 | 0 |
| HSF2 | inf | 0.450549451 | 1 | 0 |
| HSH2D | inf | 0.450549451 | 1 | 0 |
| RPS6 | inf | 0.450549451 | 1 | 0 |
| HSP90AB1 | inf | 0.450549451 | 1 | 0 |
| HS3ST6 | inf | 0.450549451 | 1 | 0 |
| HSP90B1 | inf | 0.450549451 | 1 | 0 |
| HSPA12B | inf | 0.450549451 | 1 | 0 |
| HSPA13 | inf | 0.450549451 | 1 | 0 |
| HSPA1B | inf | 0.450549451 | 1 | 0 |
| HSPA5 | inf | 0.450549451 | 1 | 0 |
| HSPA8 | inf | 0.450549451 | 1 | 0 |
| HSPB1 | inf | 0.450549451 | 1 | 0 |
| HSPB11 | inf | 0.450549451 | 1 | 0 |
| RPLP2 | inf | 0.450549451 | 1 | 0 |
| HSPBAP1 | inf | 0.450549451 | 1 | 0 |
| RPLP1 | inf | 0.450549451 | 1 | 0 |
| RPL3L | inf | 0.450549451 | 1 | 0 |
| RPS25 | inf | 0.450549451 | 1 | 0 |
| HS3ST1 | inf | 0.450549451 | 1 | 0 |
| RRAGB | inf | 0.450549451 | 1 | 0 |
| HRAS | inf | 0.450549451 | 1 | 0 |
| S100A16 | inf | 0.450549451 | 1 | 0 |
| S100A10 | inf | 0.450549451 | 1 | 0 |
| HMBS | inf | 0.450549451 | 1 | 0 |
| RXFP1 | inf | 0.450549451 | 1 | 0 |
| RWDD3 | inf | 0.450549451 | 1 | 0 |
| HMGCR | inf | 0.450549451 | 1 | 0 |
| HMGCS2 | inf | 0.450549451 | 1 | 0 |
| HMGN1 | inf | 0.450549451 | 1 | 0 |
| RUSC1-AS1 | inf | 0.450549451 | 1 | 0 |
| HMOX1 | inf | 0.450549451 | 1 | 0 |
| JPT2 | inf | 0.450549451 | 1 | 0 |
| RTP3 | inf | 0.450549451 | 1 | 0 |
| HNRNPD | inf | 0.450549451 | 1 | 0 |
| RTF1 | inf | 0.450549451 | 1 | 0 |
| HORMAD1 | inf | 0.450549451 | 1 | 0 |
| HOXA10 | inf | 0.450549451 | 1 | 0 |
| RSRC2 | inf | 0.450549451 | 1 | 0 |
| RSRC1 | inf | 0.450549451 | 1 | 0 |
| HOXA6 | inf | 0.450549451 | 1 | 0 |
| RSPO1 | inf | 0.450549451 | 1 | 0 |
| RSPH6A | inf | 0.450549451 | 1 | 0 |
| HOXC8 | inf | 0.450549451 | 1 | 0 |
| HOXC9 | inf | 0.450549451 | 1 | 0 |
| HOXD10 | inf | 0.450549451 | 1 | 0 |
| HOXD12 | inf | 0.450549451 | 1 | 0 |
| HTR1A | inf | 0.450549451 | 1 | 0 |
| GTPBP3 | inf | 0.450549451 | 1 | 0 |
| IRGM | inf | 0.450549451 | 1 | 0 |
| RFFL | inf | 0.450549451 | 1 | 0 |
| PSMC2 | inf | 0.450549451 | 1 | 0 |
| KRTAP4-4 | inf | 0.450549451 | 1 | 0 |
| PSCA | inf | 0.450549451 | 1 | 0 |
| KRTCAP3 | inf | 0.450549451 | 1 | 0 |
| PRUNE1 | inf | 0.450549451 | 1 | 0 |
| PRSS54 | inf | 0.450549451 | 1 | 0 |
| PRSS48 | inf | 0.450549451 | 1 | 0 |
| PRSS38 | inf | 0.450549451 | 1 | 0 |
| PRSS27 | inf | 0.450549451 | 1 | 0 |
| LAIR2 | inf | 0.450549451 | 1 | 0 |
| PRRG4 | inf | 0.450549451 | 1 | 0 |
| KRTAP29-1 | inf | 0.450549451 | 1 | 0 |
| LAMTOR1 | inf | 0.450549451 | 1 | 0 |
| LAP3 | inf | 0.450549451 | 1 | 0 |
| LARP4 | inf | 0.450549451 | 1 | 0 |
| LASP1 | inf | 0.450549451 | 1 | 0 |
| SPNS1 | inf | 0.450549451 | 1 | 0 |
| LAX1 | inf | 0.450549451 | 1 | 0 |
| LAYN | inf | 0.450549451 | 1 | 0 |
| LCE1A | inf | 0.450549451 | 1 | 0 |
| PRR16 | inf | 0.450549451 | 1 | 0 |
| LCE3A | inf | 0.450549451 | 1 | 0 |
| PRR11 | inf | 0.450549451 | 1 | 0 |
| LCN10 | inf | 0.450549451 | 1 | 0 |
| LAMTOR5 | inf | 0.450549451 | 1 | 0 |
| LCN12 | inf | 0.450549451 | 1 | 0 |
| PSMD3 | inf | 0.450549451 | 1 | 0 |
| KRTAP22-1 | inf | 0.450549451 | 1 | 0 |
| KMO | inf | 0.450549451 | 1 | 0 |
| KPNA2 | inf | 0.450549451 | 1 | 0 |
| KPNA5 | inf | 0.450549451 | 1 | 0 |
| KPNB1 | inf | 0.450549451 | 1 | 0 |
| KPRP | inf | 0.450549451 | 1 | 0 |
| KRBA2 | inf | 0.450549451 | 1 | 0 |
| KRBOX1 | inf | 0.450549451 | 1 | 0 |
| KRT1 | inf | 0.450549451 | 1 | 0 |
| PTPRCAP | inf | 0.450549451 | 1 | 0 |
| KRT19 | inf | 0.450549451 | 1 | 0 |
| KRT40 | inf | 0.450549451 | 1 | 0 |
| KRTAP25-1 | inf | 0.450549451 | 1 | 0 |
| PTGIR | inf | 0.450549451 | 1 | 0 |
| KRT80 | inf | 0.450549451 | 1 | 0 |
| PTCHD4 | inf | 0.450549451 | 1 | 0 |
| KRTAP1-5 | inf | 0.450549451 | 1 | 0 |
| KRTAP10-8 | inf | 0.450549451 | 1 | 0 |
| PSRC1 | inf | 0.450549451 | 1 | 0 |
| KRTAP13-2 | inf | 0.450549451 | 1 | 0 |
| PSME3 | inf | 0.450549451 | 1 | 0 |
| KRTAP19-4 | inf | 0.450549451 | 1 | 0 |
| PSME1 | inf | 0.450549451 | 1 | 0 |
| PSMD8 | inf | 0.450549451 | 1 | 0 |
| KRTAP21-1 | inf | 0.450549451 | 1 | 0 |
| PTF1A | inf | 0.450549451 | 1 | 0 |
| PROSER1 | inf | 0.450549451 | 1 | 0 |
| LDB2 | inf | 0.450549451 | 1 | 0 |
| LDB2 | inf | 0.450549451 | 1 | 0 |
| LINGO3 | inf | 0.450549451 | 1 | 0 |
| LINS1 | inf | 0.450549451 | 1 | 0 |
| SCGB1D1 | inf | 0.450549451 | 1 | 0 |
| LIPG | inf | 0.450549451 | 1 | 0 |
| LIPH | inf | 0.450549451 | 1 | 0 |
| LIPT2 | inf | 0.450549451 | 1 | 0 |
| LMAN2L | inf | 0.450549451 | 1 | 0 |
| LMCD1 | inf | 0.450549451 | 1 | 0 |
| LMNA | inf | 0.450549451 | 1 | 0 |
| LMNB2 | inf | 0.450549451 | 1 | 0 |
| LMO1 | inf | 0.450549451 | 1 | 0 |
| LINC00955 | inf | 0.450549451 | 1 | 0 |
| LMTK2 | inf | 0.450549451 | 1 | 0 |
| LOC100130097 | inf | 0.450549451 | 1 | 0 |
| PRAME | inf | 0.450549451 | 1 | 0 |
| PRAF2 | inf | 0.450549451 | 1 | 0 |
| LOC100131107 | inf | 0.450549451 | 1 | 0 |
| LOC100132146 | inf | 0.450549451 | 1 | 0 |
| LOC100132731 | inf | 0.450549451 | 1 | 0 |
| PRADC1 | inf | 0.450549451 | 1 | 0 |
| LOC100144595 | inf | 0.450549451 | 1 | 0 |
| LOC100287036 | inf | 0.450549451 | 1 | 0 |
| PQLC3 | inf | 0.450549451 | 1 | 0 |
| PPT1 | inf | 0.450549451 | 1 | 0 |
| LOC100129924 | inf | 0.450549451 | 1 | 0 |
| LINC00910 | inf | 0.450549451 | 1 | 0 |
| LIME1 | inf | 0.450549451 | 1 | 0 |
| PXDN | inf | 0.450549451 | 1 | 0 |
| PROP1 | inf | 0.450549451 | 1 | 0 |
| LDLRAD2 | inf | 0.450549451 | 1 | 0 |
| LDLRAD3 | inf | 0.450549451 | 1 | 0 |
| PROKR1 | inf | 0.450549451 | 1 | 0 |
| PROK2 | inf | 0.450549451 | 1 | 0 |
| CNMD | inf | 0.450549451 | 1 | 0 |
| LENG9 | inf | 0.450549451 | 1 | 0 |
| PRLHR | inf | 0.450549451 | 1 | 0 |
| PRKX | inf | 0.450549451 | 1 | 0 |
| PRKD3 | inf | 0.450549451 | 1 | 0 |
| LGALS13 | inf | 0.450549451 | 1 | 0 |
| PRKD2 | inf | 0.450549451 | 1 | 0 |
| LGALS3 | inf | 0.450549451 | 1 | 0 |
| LGALSL | inf | 0.450549451 | 1 | 0 |
| LGI3 | inf | 0.450549451 | 1 | 0 |
| PRKCG | inf | 0.450549451 | 1 | 0 |
| LGMN | inf | 0.450549451 | 1 | 0 |
| PRKCD | inf | 0.450549451 | 1 | 0 |
| PRKAG2 | inf | 0.450549451 | 1 | 0 |
| PRKAA2 | inf | 0.450549451 | 1 | 0 |
| LHX1 | inf | 0.450549451 | 1 | 0 |
| PRH1-PRR4&TAS2R14 | inf | 0.450549451 | 1 | 0 |
| LRIG1 | inf | 0.450549451 | 1 | 0 |
| PRG3 | inf | 0.450549451 | 1 | 0 |
| LILRA4 | inf | 0.450549451 | 1 | 0 |
| KLRC1 | inf | 0.450549451 | 1 | 0 |
| RFPL1 | inf | 0.450549451 | 1 | 0 |
| KLKB1 | inf | 0.450549451 | 1 | 0 |
| KLHL41 | inf | 0.450549451 | 1 | 0 |
| RBM26 | inf | 0.450549451 | 1 | 0 |
| KATNBL1 | inf | 0.450549451 | 1 | 0 |
| RBM25 | inf | 0.450549451 | 1 | 0 |
| KBTBD2 | inf | 0.450549451 | 1 | 0 |
| RBM17 | inf | 0.450549451 | 1 | 0 |
| KCNA4 | inf | 0.450549451 | 1 | 0 |
| KCNA6 | inf | 0.450549451 | 1 | 0 |
| RBM11 | inf | 0.450549451 | 1 | 0 |
| KCNAB3 | inf | 0.450549451 | 1 | 0 |
| RBFOX2 | inf | 0.450549451 | 1 | 0 |
| KCNC4 | inf | 0.450549451 | 1 | 0 |
| RBM4B | inf | 0.450549451 | 1 | 0 |
| KCND3 | inf | 0.450549451 | 1 | 0 |
| RBBP9 | inf | 0.450549451 | 1 | 0 |
| RBBP8 | inf | 0.450549451 | 1 | 0 |
| KCNIP3 | inf | 0.450549451 | 1 | 0 |
| KCNJ15 | inf | 0.450549451 | 1 | 0 |
| RBAK | inf | 0.450549451 | 1 | 0 |
| RB1 | inf | 0.450549451 | 1 | 0 |
| RASSF8 | inf | 0.450549451 | 1 | 0 |
| RASSF5 | inf | 0.450549451 | 1 | 0 |
| KCNK18 | inf | 0.450549451 | 1 | 0 |
| KCNK3 | inf | 0.450549451 | 1 | 0 |
| RASL11A | inf | 0.450549451 | 1 | 0 |
| KCNE5 | inf | 0.450549451 | 1 | 0 |
| KCNN2 | inf | 0.450549451 | 1 | 0 |
| RBM7 | inf | 0.450549451 | 1 | 0 |
| KAAG1 | inf | 0.450549451 | 1 | 0 |
| ISCU | inf | 0.450549451 | 1 | 0 |
| ISG20 | inf | 0.450549451 | 1 | 0 |
| ISM1 | inf | 0.450549451 | 1 | 0 |
| ISPD | inf | 0.450549451 | 1 | 0 |
| RESP18 | inf | 0.450549451 | 1 | 0 |
| ITGA5 | inf | 0.450549451 | 1 | 0 |
| REPS1 | inf | 0.450549451 | 1 | 0 |
| ITGB1 | inf | 0.450549451 | 1 | 0 |
| ITGB3BP | inf | 0.450549451 | 1 | 0 |
| REEP6 | inf | 0.450549451 | 1 | 0 |
| REEP1 | inf | 0.450549451 | 1 | 0 |
| ANOS1 | inf | 0.450549451 | 1 | 0 |
| ITM2B | inf | 0.450549451 | 1 | 0 |
| IZUMO1 | inf | 0.450549451 | 1 | 0 |
| RDH16 | inf | 0.450549451 | 1 | 0 |
| RDH13 | inf | 0.450549451 | 1 | 0 |
| RCOR2 | inf | 0.450549451 | 1 | 0 |
| JAKMIP1 | inf | 0.450549451 | 1 | 0 |
| JAKMIP2-AS1 | inf | 0.450549451 | 1 | 0 |
| JAM2 | inf | 0.450549451 | 1 | 0 |
| RCAN1 | inf | 0.450549451 | 1 | 0 |
| RBPMS | inf | 0.450549451 | 1 | 0 |
| RBMS2 | inf | 0.450549451 | 1 | 0 |
| JTB | inf | 0.450549451 | 1 | 0 |
| ITPRIPL2 | inf | 0.450549451 | 1 | 0 |
| KCNQ3 | inf | 0.450549451 | 1 | 0 |
| RASA2 | inf | 0.450549451 | 1 | 0 |
| KCNT2 | inf | 0.450549451 | 1 | 0 |
| RAB43 | inf | 0.450549451 | 1 | 0 |
| R3HDM4 | inf | 0.450549451 | 1 | 0 |
| KIF2A | inf | 0.450549451 | 1 | 0 |
| QSOX2 | inf | 0.450549451 | 1 | 0 |
| KIR2DS4 | inf | 0.450549451 | 1 | 0 |
| KIR3DL1 | inf | 0.450549451 | 1 | 0 |
| KIR3DL3 | inf | 0.450549451 | 1 | 0 |
| QARS | inf | 0.450549451 | 1 | 0 |
| KLC3 | inf | 0.450549451 | 1 | 0 |
| KLF1 | inf | 0.450549451 | 1 | 0 |
| KLF11 | inf | 0.450549451 | 1 | 0 |
| RAB11FIP1 | inf | 0.450549451 | 1 | 0 |
| SP6 | inf | 0.450549451 | 1 | 0 |
| KLHDC1 | inf | 0.450549451 | 1 | 0 |
| KLHDC10 | inf | 0.450549451 | 1 | 0 |
| KLHDC8A | inf | 0.450549451 | 1 | 0 |
| PYCR2 | inf | 0.450549451 | 1 | 0 |
| KLHL11 | inf | 0.450549451 | 1 | 0 |
| KLHL14 | inf | 0.450549451 | 1 | 0 |
| KLHL3 | inf | 0.450549451 | 1 | 0 |
| NECTIN1 | inf | 0.450549451 | 1 | 0 |
| PVRIG | inf | 0.450549451 | 1 | 0 |
| KLHL32 | inf | 0.450549451 | 1 | 0 |
| PUSL1 | inf | 0.450549451 | 1 | 0 |
| KLF15 | inf | 0.450549451 | 1 | 0 |
| RAB11FIP2 | inf | 0.450549451 | 1 | 0 |
| KIF21A | inf | 0.450549451 | 1 | 0 |
| RAB19 | inf | 0.450549451 | 1 | 0 |
| KCTD18 | inf | 0.450549451 | 1 | 0 |
| KCTD4 | inf | 0.450549451 | 1 | 0 |
| KDELR3 | inf | 0.450549451 | 1 | 0 |
| KDM4A | inf | 0.450549451 | 1 | 0 |
| RAPGEF1 | inf | 0.450549451 | 1 | 0 |
| RAP1GDS1 | inf | 0.450549451 | 1 | 0 |
| RAP1GAP2 | inf | 0.450549451 | 1 | 0 |
| RAP1GAP | inf | 0.450549451 | 1 | 0 |
| RANBP9 | inf | 0.450549451 | 1 | 0 |
| KHK | inf | 0.450549451 | 1 | 0 |
| RANBP3 | inf | 0.450549451 | 1 | 0 |
| RAMP2 | inf | 0.450549451 | 1 | 0 |
| RALGDS | inf | 0.450549451 | 1 | 0 |
| RAI14 | inf | 0.450549451 | 1 | 0 |
| RAD51D | inf | 0.450549451 | 1 | 0 |
| RAD23B | inf | 0.450549451 | 1 | 0 |
| CIP2A | inf | 0.450549451 | 1 | 0 |
| RABGAP1 | inf | 0.450549451 | 1 | 0 |
| RAB6C | inf | 0.450549451 | 1 | 0 |
| RAB5A | inf | 0.450549451 | 1 | 0 |
| RAB42P1 | inf | 0.450549451 | 1 | 0 |
| RAB3B | inf | 0.450549451 | 1 | 0 |
| RAB39A | inf | 0.450549451 | 1 | 0 |
| RAB27B | inf | 0.450549451 | 1 | 0 |
| RAB20 | inf | 0.450549451 | 1 | 0 |
| KLK7 | inf | 0.450549451 | 1 | 0 |
| SND1 | inf | 0.450549451 | 1 | 0 |
| OR10A5 | inf | 0.450549451 | 1 | 0 |
| FAM212B | inf | 0.450549451 | 1 | 0 |
| CDO1 | inf | 0.450549451 | 1 | 0 |
| CDR2L | inf | 0.450549451 | 1 | 0 |
| CDRT15 | inf | 0.450549451 | 1 | 0 |
| CHEK2 | inf | 0.450549451 | 1 | 0 |
| CDX2 | inf | 0.450549451 | 1 | 0 |
| TRAF3 | inf | 0.450549451 | 1 | 0 |
| CEACAM7 | inf | 0.450549451 | 1 | 0 |
| TMEM121B | inf | 0.450549451 | 1 | 0 |
| CELA3B | inf | 0.450549451 | 1 | 0 |
| TPMT | inf | 0.450549451 | 1 | 0 |
| CELF6 | inf | 0.450549451 | 1 | 0 |
| TPGS1 | inf | 0.450549451 | 1 | 0 |
| CENPQ | inf | 0.450549451 | 1 | 0 |
| TP53INP2 | inf | 0.450549451 | 1 | 0 |
| TP53 | inf | 0.450549451 | 1 | 0 |
| CEP19 | inf | 0.450549451 | 1 | 0 |
| CEP85L | inf | 0.450549451 | 1 | 0 |
| TOP1 | inf | 0.450549451 | 1 | 0 |
| TOM1L2 | inf | 0.450549451 | 1 | 0 |
| CERS2 | inf | 0.450549451 | 1 | 0 |
| TOM1L1 | inf | 0.450549451 | 1 | 0 |
| TOE1 | inf | 0.450549451 | 1 | 0 |
| CH25H | inf | 0.450549451 | 1 | 0 |
| TRAK2 | inf | 0.450549451 | 1 | 0 |
| CHCHD10 | inf | 0.450549451 | 1 | 0 |
| TRAP1 | inf | 0.450549451 | 1 | 0 |
| CDK7 | inf | 0.450549451 | 1 | 0 |
| TREM2 | inf | 0.450549451 | 1 | 0 |
| CDC6 | inf | 0.450549451 | 1 | 0 |
| CDC7 | inf | 0.450549451 | 1 | 0 |
| TRDN | inf | 0.450549451 | 1 | 0 |
| TRDMT1 | inf | 0.450549451 | 1 | 0 |
| TRBV27 | inf | 0.450549451 | 1 | 0 |
| CDH6 | inf | 0.450549451 | 1 | 0 |
| TRAV8-7 | inf | 0.450549451 | 1 | 0 |
| TRAV8-2 | inf | 0.450549451 | 1 | 0 |
| TRAV7 | inf | 0.450549451 | 1 | 0 |
| TRAV38-1 | inf | 0.450549451 | 1 | 0 |
| CDK1 | inf | 0.450549451 | 1 | 0 |
| CDK10 | inf | 0.450549451 | 1 | 0 |
| CDK11B | inf | 0.450549451 | 1 | 0 |
| TRAV34 | inf | 0.450549451 | 1 | 0 |
| TRAV3 | inf | 0.450549451 | 1 | 0 |
| CDK16 | inf | 0.450549451 | 1 | 0 |
| CDK18 | inf | 0.450549451 | 1 | 0 |
| CDK2 | inf | 0.450549451 | 1 | 0 |
| TRAV29DV5 | inf | 0.450549451 | 1 | 0 |
| TRAV17 | inf | 0.450549451 | 1 | 0 |
| CDK5R1 | inf | 0.450549451 | 1 | 0 |
| TRAV12-2 | inf | 0.450549451 | 1 | 0 |
| CDKN1A | inf | 0.450549451 | 1 | 0 |
| CHCHD3 | inf | 0.450549451 | 1 | 0 |
| TNKS | inf | 0.450549451 | 1 | 0 |
| TNIP2 | inf | 0.450549451 | 1 | 0 |
| CLCF1 | inf | 0.450549451 | 1 | 0 |
| CLDN10 | inf | 0.450549451 | 1 | 0 |
| CLDN11 | inf | 0.450549451 | 1 | 0 |
| TMEM61 | inf | 0.450549451 | 1 | 0 |
| CLDN18 | inf | 0.450549451 | 1 | 0 |
| CLDN19 | inf | 0.450549451 | 1 | 0 |
| CLDN20 | inf | 0.450549451 | 1 | 0 |
| CLDN6 | inf | 0.450549451 | 1 | 0 |
| PIP4P2 | inf | 0.450549451 | 1 | 0 |
| CLEC10A | inf | 0.450549451 | 1 | 0 |
| TMEM45B | inf | 0.450549451 | 1 | 0 |
| CLEC12B | inf | 0.450549451 | 1 | 0 |
| CLEC1B | inf | 0.450549451 | 1 | 0 |
| CLEC3B | inf | 0.450549451 | 1 | 0 |
| TMEM39A | inf | 0.450549451 | 1 | 0 |
| TMEM38B | inf | 0.450549451 | 1 | 0 |
| CLEC7A | inf | 0.450549451 | 1 | 0 |
| TMEM31 | inf | 0.450549451 | 1 | 0 |
| CLIC6 | inf | 0.450549451 | 1 | 0 |
| SLITRK2 | inf | 0.450549451 | 1 | 0 |
| TMEM253 | inf | 0.450549451 | 1 | 0 |
| CLK2 | inf | 0.450549451 | 1 | 0 |
| CLK4 | inf | 0.450549451 | 1 | 0 |
| TMEM74B | inf | 0.450549451 | 1 | 0 |
| TMEM79 | inf | 0.450549451 | 1 | 0 |
| TMEM87A | inf | 0.450549451 | 1 | 0 |
| TMEM88B | inf | 0.450549451 | 1 | 0 |
| TNFSF15 | inf | 0.450549451 | 1 | 0 |
| CHGB | inf | 0.450549451 | 1 | 0 |
| TNFRSF14 | inf | 0.450549451 | 1 | 0 |
| TNFRSF13C | inf | 0.450549451 | 1 | 0 |
| CHMP1A | inf | 0.450549451 | 1 | 0 |
| TNFRSF13B | inf | 0.450549451 | 1 | 0 |
| CHP1 | inf | 0.450549451 | 1 | 0 |
| CHPT1 | inf | 0.450549451 | 1 | 0 |
| CHRFAM7A | inf | 0.450549451 | 1 | 0 |
| CHRM5 | inf | 0.450549451 | 1 | 0 |
| TMPRSS4 | inf | 0.450549451 | 1 | 0 |
| CDC40 | inf | 0.450549451 | 1 | 0 |
| CHRNE | inf | 0.450549451 | 1 | 0 |
| CHST1 | inf | 0.450549451 | 1 | 0 |
| CHST14 | inf | 0.450549451 | 1 | 0 |
| TMOD3 | inf | 0.450549451 | 1 | 0 |
| CHSY3 | inf | 0.450549451 | 1 | 0 |
| CHURC1-FNTB | inf | 0.450549451 | 1 | 0 |
| CIAPIN1 | inf | 0.450549451 | 1 | 0 |
| CIB2 | inf | 0.450549451 | 1 | 0 |
| CIDEB | inf | 0.450549451 | 1 | 0 |
| CIDEC | inf | 0.450549451 | 1 | 0 |
| CKAP2 | inf | 0.450549451 | 1 | 0 |
| CKB | inf | 0.450549451 | 1 | 0 |
| TMPRSS11D | inf | 0.450549451 | 1 | 0 |
| CLMN | inf | 0.450549451 | 1 | 0 |
| TRGV1 | inf | 0.450549451 | 1 | 0 |
| CDC20B | inf | 0.450549451 | 1 | 0 |
| CBWD5 | inf | 0.450549451 | 1 | 0 |
| KYAT1 | inf | 0.450549451 | 1 | 0 |
| CFAP36 | inf | 0.450549451 | 1 | 0 |
| TTC23 | inf | 0.450549451 | 1 | 0 |
| PRIMPOL | inf | 0.450549451 | 1 | 0 |
| VPS50 | inf | 0.450549451 | 1 | 0 |
| CCDC134 | inf | 0.450549451 | 1 | 0 |
| TSTD2 | inf | 0.450549451 | 1 | 0 |
| TSSK4 | inf | 0.450549451 | 1 | 0 |
| CCDC152 | inf | 0.450549451 | 1 | 0 |
| CCDC159 | inf | 0.450549451 | 1 | 0 |
| CCDC17 | inf | 0.450549451 | 1 | 0 |
| TSR1 | inf | 0.450549451 | 1 | 0 |
| CCDC174 | inf | 0.450549451 | 1 | 0 |
| TSPY2 | inf | 0.450549451 | 1 | 0 |
| CCDC181 | inf | 0.450549451 | 1 | 0 |
| TSPAN7 | inf | 0.450549451 | 1 | 0 |
| TSPAN4 | inf | 0.450549451 | 1 | 0 |
| CCDC25 | inf | 0.450549451 | 1 | 0 |
| CCDC28B | inf | 0.450549451 | 1 | 0 |
| CEP83 | inf | 0.450549451 | 1 | 0 |
| CCDC51 | inf | 0.450549451 | 1 | 0 |
| TSEN2 | inf | 0.450549451 | 1 | 0 |
| TTC36 | inf | 0.450549451 | 1 | 0 |
| TSC22D3 | inf | 0.450549451 | 1 | 0 |
| TTC39A | inf | 0.450549451 | 1 | 0 |
| TTC5 | inf | 0.450549451 | 1 | 0 |
| CABYR | inf | 0.450549451 | 1 | 0 |
| UBE2W | inf | 0.450549451 | 1 | 0 |
| UBE2S | inf | 0.450549451 | 1 | 0 |
| CACNB4 | inf | 0.450549451 | 1 | 0 |
| CACYBP | inf | 0.450549451 | 1 | 0 |
| UBALD1 | inf | 0.450549451 | 1 | 0 |
| CALCA | inf | 0.450549451 | 1 | 0 |
| U2SURP | inf | 0.450549451 | 1 | 0 |
| TYRP1 | inf | 0.450549451 | 1 | 0 |
| CALML4 | inf | 0.450549451 | 1 | 0 |
| CALR | inf | 0.450549451 | 1 | 0 |
| TUFM | inf | 0.450549451 | 1 | 0 |
| CAPNS2 | inf | 0.450549451 | 1 | 0 |
| TUBB6 | inf | 0.450549451 | 1 | 0 |
| TUBAL3 | inf | 0.450549451 | 1 | 0 |
| CARD8 | inf | 0.450549451 | 1 | 0 |
| TUBA3D | inf | 0.450549451 | 1 | 0 |
| TUBA3C | inf | 0.450549451 | 1 | 0 |
| TUBA1B | inf | 0.450549451 | 1 | 0 |
| CASC1 | inf | 0.450549451 | 1 | 0 |
| TTPAL | inf | 0.450549451 | 1 | 0 |
| TTL | inf | 0.450549451 | 1 | 0 |
| CAV1 | inf | 0.450549451 | 1 | 0 |
| CAV3 | inf | 0.450549451 | 1 | 0 |
| CCDC69 | inf | 0.450549451 | 1 | 0 |
| CCDC83 | inf | 0.450549451 | 1 | 0 |
| CCDC84 | inf | 0.450549451 | 1 | 0 |
| CD14 | inf | 0.450549451 | 1 | 0 |
| CD163L1 | inf | 0.450549451 | 1 | 0 |
| CD200 | inf | 0.450549451 | 1 | 0 |
| CD200R1L | inf | 0.450549451 | 1 | 0 |
| TRIM49D1 | inf | 0.450549451 | 1 | 0 |
| TRIM49B | inf | 0.450549451 | 1 | 0 |
| CD244 | inf | 0.450549451 | 1 | 0 |
| TRIM49 | inf | 0.450549451 | 1 | 0 |
| CD2AP | inf | 0.450549451 | 1 | 0 |
| TRIM43B | inf | 0.450549451 | 1 | 0 |
| CD3G | inf | 0.450549451 | 1 | 0 |
| TRIM40 | inf | 0.450549451 | 1 | 0 |
| TRIM37 | inf | 0.450549451 | 1 | 0 |
| TRIM3 | inf | 0.450549451 | 1 | 0 |
| CD59 | inf | 0.450549451 | 1 | 0 |
| CD5L | inf | 0.450549451 | 1 | 0 |
| CD79B | inf | 0.450549451 | 1 | 0 |
| CD80 | inf | 0.450549451 | 1 | 0 |
| TRIM27 | inf | 0.450549451 | 1 | 0 |
| TRIM2 | inf | 0.450549451 | 1 | 0 |
| TRIM14 | inf | 0.450549451 | 1 | 0 |
| TRIM13 | inf | 0.450549451 | 1 | 0 |
| CDC20 | inf | 0.450549451 | 1 | 0 |
| TRIM63 | inf | 0.450549451 | 1 | 0 |
| CCT6B | inf | 0.450549451 | 1 | 0 |
| CCR9 | inf | 0.450549451 | 1 | 0 |
| CCR8 | inf | 0.450549451 | 1 | 0 |
| CCDC85C | inf | 0.450549451 | 1 | 0 |
| CCDC89 | inf | 0.450549451 | 1 | 0 |
| TROVE2 | inf | 0.450549451 | 1 | 0 |
| CCIN | inf | 0.450549451 | 1 | 0 |
| TRH-GTG1-7 | inf | 0.450549451 | 1 | 0 |
| CCL2 | inf | 0.450549451 | 1 | 0 |
| CCL25 | inf | 0.450549451 | 1 | 0 |
| CCL28 | inf | 0.450549451 | 1 | 0 |
| TRMT6 | inf | 0.450549451 | 1 | 0 |
| CCNA1 | inf | 0.450549451 | 1 | 0 |
| CCNB1 | inf | 0.450549451 | 1 | 0 |
| TRGV3 | inf | 0.450549451 | 1 | 0 |
| TRMT13 | inf | 0.450549451 | 1 | 0 |
| TRMT11 | inf | 0.450549451 | 1 | 0 |
| TRMT10C | inf | 0.450549451 | 1 | 0 |
| CCNG2 | inf | 0.450549451 | 1 | 0 |
| CCNI2 | inf | 0.450549451 | 1 | 0 |
| FAM213A | inf | 0.450549451 | 1 | 0 |
| CCNK | inf | 0.450549451 | 1 | 0 |
| TRIT1 | inf | 0.450549451 | 1 | 0 |
| TRIP4 | inf | 0.450549451 | 1 | 0 |
| CCR10 | inf | 0.450549451 | 1 | 0 |
| CCR2 | inf | 0.450549451 | 1 | 0 |
| CCR6 | inf | 0.450549451 | 1 | 0 |
| TRMT12 | inf | 0.450549451 | 1 | 0 |
| TMEM230 | inf | 0.450549451 | 1 | 0 |
| TMEM223 | inf | 0.450549451 | 1 | 0 |
| CLRN3 | inf | 0.450549451 | 1 | 0 |
| CYSLTR1 | inf | 0.450549451 | 1 | 0 |
| CYTIP | inf | 0.450549451 | 1 | 0 |
| DACH1 | inf | 0.450549451 | 1 | 0 |
| DACH2 | inf | 0.450549451 | 1 | 0 |
| DAG1 | inf | 0.450549451 | 1 | 0 |
| TEC | inf | 0.450549451 | 1 | 0 |
| TEAD2 | inf | 0.450549451 | 1 | 0 |
| TDRD3 | inf | 0.450549451 | 1 | 0 |
| DBF4 | inf | 0.450549451 | 1 | 0 |
| TDP2 | inf | 0.450549451 | 1 | 0 |
| DBT | inf | 0.450549451 | 1 | 0 |
| DCAF12L1 | inf | 0.450549451 | 1 | 0 |
| DCAF13 | inf | 0.450549451 | 1 | 0 |
| DCAF17 | inf | 0.450549451 | 1 | 0 |
| TCP11X2 | inf | 0.450549451 | 1 | 0 |
| DCD | inf | 0.450549451 | 1 | 0 |
| DCDC2 | inf | 0.450549451 | 1 | 0 |
| DCDC2C | inf | 0.450549451 | 1 | 0 |
| DCLK3 | inf | 0.450549451 | 1 | 0 |
| DCN | inf | 0.450549451 | 1 | 0 |
| TCL1B | inf | 0.450549451 | 1 | 0 |
| TCHP | inf | 0.450549451 | 1 | 0 |
| TCHHL1 | inf | 0.450549451 | 1 | 0 |
| CYP4Z1 | inf | 0.450549451 | 1 | 0 |
| TCF7L2 | inf | 0.450549451 | 1 | 0 |
| TES | inf | 0.450549451 | 1 | 0 |
| CYP39A1 | inf | 0.450549451 | 1 | 0 |
| CXCL3 | inf | 0.450549451 | 1 | 0 |
| CXCL9 | inf | 0.450549451 | 1 | 0 |
| CXCR2 | inf | 0.450549451 | 1 | 0 |
| CXCR3 | inf | 0.450549451 | 1 | 0 |
| CXCR6 | inf | 0.450549451 | 1 | 0 |
| TGFB1I1 | inf | 0.450549451 | 1 | 0 |
| CXorf21 | inf | 0.450549451 | 1 | 0 |
| CXorf36 | inf | 0.450549451 | 1 | 0 |
| CXorf38 | inf | 0.450549451 | 1 | 0 |
| TFPT | inf | 0.450549451 | 1 | 0 |
| CXorf58 | inf | 0.450549451 | 1 | 0 |
| CYB5R2 | inf | 0.450549451 | 1 | 0 |
| CYGB | inf | 0.450549451 | 1 | 0 |
| TFAP2D | inf | 0.450549451 | 1 | 0 |
| TFAP2B | inf | 0.450549451 | 1 | 0 |
| CYP17A1 | inf | 0.450549451 | 1 | 0 |
| TEX37 | inf | 0.450549451 | 1 | 0 |
| CYP1A1 | inf | 0.450549451 | 1 | 0 |
| CYP1A2 | inf | 0.450549451 | 1 | 0 |
| TEX29 | inf | 0.450549451 | 1 | 0 |
| CYP26A1 | inf | 0.450549451 | 1 | 0 |
| TEX13B | inf | 0.450549451 | 1 | 0 |
| CYP2A7 | inf | 0.450549451 | 1 | 0 |
| TESPA1 | inf | 0.450549451 | 1 | 0 |
| DCTN4 | inf | 0.450549451 | 1 | 0 |
| TCF7L1 | inf | 0.450549451 | 1 | 0 |
| DCUN1D1 | inf | 0.450549451 | 1 | 0 |
| DFNA5 | inf | 0.450549451 | 1 | 0 |
| PJVK | inf | 0.450549451 | 1 | 0 |
| TBC1D19 | inf | 0.450549451 | 1 | 0 |
| DGCR2 | inf | 0.450549451 | 1 | 0 |
| DGCR6L | inf | 0.450549451 | 1 | 0 |
| DGCR8 | inf | 0.450549451 | 1 | 0 |
| DHCR24 | inf | 0.450549451 | 1 | 0 |
| DHDDS | inf | 0.450549451 | 1 | 0 |
| TBC1D10A | inf | 0.450549451 | 1 | 0 |
| DHFR2 | inf | 0.450549451 | 1 | 0 |
| DHH | inf | 0.450549451 | 1 | 0 |
| TBC1D1 | inf | 0.450549451 | 1 | 0 |
| DHPS | inf | 0.450549451 | 1 | 0 |
| DHRS1 | inf | 0.450549451 | 1 | 0 |
| TAX1BP3 | inf | 0.450549451 | 1 | 0 |
| DHRS12 | inf | 0.450549451 | 1 | 0 |
| DHRS3 | inf | 0.450549451 | 1 | 0 |
| DHRS4 | inf | 0.450549451 | 1 | 0 |
| DHRS4L2 | inf | 0.450549451 | 1 | 0 |
| DHRS7B | inf | 0.450549451 | 1 | 0 |
| TAS2R4 | inf | 0.450549451 | 1 | 0 |
| TAS2R3 | inf | 0.450549451 | 1 | 0 |
| TAS2R14 | inf | 0.450549451 | 1 | 0 |
| DES | inf | 0.450549451 | 1 | 0 |
| TBC1D29 | inf | 0.450549451 | 1 | 0 |
| DERA | inf | 0.450549451 | 1 | 0 |
| DEFB136 | inf | 0.450549451 | 1 | 0 |
| TCF23 | inf | 0.450549451 | 1 | 0 |
| DDHD2 | inf | 0.450549451 | 1 | 0 |
| TCF20 | inf | 0.450549451 | 1 | 0 |
| DDX17 | inf | 0.450549451 | 1 | 0 |
| DDX20 | inf | 0.450549451 | 1 | 0 |
| DDX39A | inf | 0.450549451 | 1 | 0 |
| DDX3X | inf | 0.450549451 | 1 | 0 |
| DDX4 | inf | 0.450549451 | 1 | 0 |
| TBX6 | inf | 0.450549451 | 1 | 0 |
| TBX3 | inf | 0.450549451 | 1 | 0 |
| TBX22 | inf | 0.450549451 | 1 | 0 |
| CXCL2 | inf | 0.450549451 | 1 | 0 |
| DDX5 | inf | 0.450549451 | 1 | 0 |
| TBX1 | inf | 0.450549451 | 1 | 0 |
| TBR1 | inf | 0.450549451 | 1 | 0 |
| DEFA6 | inf | 0.450549451 | 1 | 0 |
| TBL1X | inf | 0.450549451 | 1 | 0 |
| DEFB108B | inf | 0.450549451 | 1 | 0 |
| DEFB114 | inf | 0.450549451 | 1 | 0 |
| DEFB119 | inf | 0.450549451 | 1 | 0 |
| DEFB121 | inf | 0.450549451 | 1 | 0 |
| DEFB125 | inf | 0.450549451 | 1 | 0 |
| DEFB128 | inf | 0.450549451 | 1 | 0 |
| TBCB | inf | 0.450549451 | 1 | 0 |
| TBX18 | inf | 0.450549451 | 1 | 0 |
| CXCL17 | inf | 0.450549451 | 1 | 0 |
| CXCL11 | inf | 0.450549451 | 1 | 0 |
| TGFBRAP1 | inf | 0.450549451 | 1 | 0 |
| TMEM151A | inf | 0.450549451 | 1 | 0 |
| TMEM150A | inf | 0.450549451 | 1 | 0 |
| COG8 | inf | 0.450549451 | 1 | 0 |
| TMEM141 | inf | 0.450549451 | 1 | 0 |
| TMEM134 | inf | 0.450549451 | 1 | 0 |
| TMEM133 | inf | 0.450549451 | 1 | 0 |
| COL4A2 | inf | 0.450549451 | 1 | 0 |
| TMEM108 | inf | 0.450549451 | 1 | 0 |
| TMEM105 | inf | 0.450549451 | 1 | 0 |
| COL8A1 | inf | 0.450549451 | 1 | 0 |
| COLQ | inf | 0.450549451 | 1 | 0 |
| TMED1 | inf | 0.450549451 | 1 | 0 |
| COMMD4 | inf | 0.450549451 | 1 | 0 |
| COPB1 | inf | 0.450549451 | 1 | 0 |
| COPB2 | inf | 0.450549451 | 1 | 0 |
| COPRS | inf | 0.450549451 | 1 | 0 |
| COPS6 | inf | 0.450549451 | 1 | 0 |
| COPS7B | inf | 0.450549451 | 1 | 0 |
| COQ10A | inf | 0.450549451 | 1 | 0 |
| COQ3 | inf | 0.450549451 | 1 | 0 |
| COX5A | inf | 0.450549451 | 1 | 0 |
| TMBIM1 | inf | 0.450549451 | 1 | 0 |
| TMA16 | inf | 0.450549451 | 1 | 0 |
| TMEM151B | inf | 0.450549451 | 1 | 0 |
| TMEM161B | inf | 0.450549451 | 1 | 0 |
| TMEM163 | inf | 0.450549451 | 1 | 0 |
| TMEM174 | inf | 0.450549451 | 1 | 0 |
| TMEM217 | inf | 0.450549451 | 1 | 0 |
| TMEM212 | inf | 0.450549451 | 1 | 0 |
| CLTC | inf | 0.450549451 | 1 | 0 |
| TMEM210 | inf | 0.450549451 | 1 | 0 |
| CMPK1 | inf | 0.450549451 | 1 | 0 |
| TMEM200A | inf | 0.450549451 | 1 | 0 |
| CMSS1 | inf | 0.450549451 | 1 | 0 |
| CMTM4 | inf | 0.450549451 | 1 | 0 |
| CMTM5 | inf | 0.450549451 | 1 | 0 |
| NEMP1 | inf | 0.450549451 | 1 | 0 |
| TMEM19 | inf | 0.450549451 | 1 | 0 |
| COX8C | inf | 0.450549451 | 1 | 0 |
| CNGB3 | inf | 0.450549451 | 1 | 0 |
| TMEM184B | inf | 0.450549451 | 1 | 0 |
| TMEM183B | inf | 0.450549451 | 1 | 0 |
| CNOT10 | inf | 0.450549451 | 1 | 0 |
| MFSD13A | inf | 0.450549451 | 1 | 0 |
| CNOT3 | inf | 0.450549451 | 1 | 0 |
| TMEM178B | inf | 0.450549451 | 1 | 0 |
| TMEM178A | inf | 0.450549451 | 1 | 0 |
| CNOT6L | inf | 0.450549451 | 1 | 0 |
| TMEM177 | inf | 0.450549451 | 1 | 0 |
| CNTD2 | inf | 0.450549451 | 1 | 0 |
| CNTF | inf | 0.450549451 | 1 | 0 |
| CNKSR2 | inf | 0.450549451 | 1 | 0 |
| UCKL1 | inf | 0.450549451 | 1 | 0 |
| TM7SF3 | inf | 0.450549451 | 1 | 0 |
| CPM | inf | 0.450549451 | 1 | 0 |
| CRYZL1 | inf | 0.450549451 | 1 | 0 |
| TIGD2 | inf | 0.450549451 | 1 | 0 |
| CSNK1A1L | inf | 0.450549451 | 1 | 0 |
| CSNK1G3 | inf | 0.450549451 | 1 | 0 |
| CSNK2A2 | inf | 0.450549451 | 1 | 0 |
| THY1 | inf | 0.450549451 | 1 | 0 |
| CST1 | inf | 0.450549451 | 1 | 0 |
| CST5 | inf | 0.450549451 | 1 | 0 |
| THRSP | inf | 0.450549451 | 1 | 0 |
| THOC2 | inf | 0.450549451 | 1 | 0 |
| THOC1 | inf | 0.450549451 | 1 | 0 |
| CTDSPL | inf | 0.450549451 | 1 | 0 |
| THEM4 | inf | 0.450549451 | 1 | 0 |
| CTIF | inf | 0.450549451 | 1 | 0 |
| CTLA4 | inf | 0.450549451 | 1 | 0 |
| CTNNA1 | inf | 0.450549451 | 1 | 0 |
| THEG5 | inf | 0.450549451 | 1 | 0 |
| CTNNBL1 | inf | 0.450549451 | 1 | 0 |
| CTPS1 | inf | 0.450549451 | 1 | 0 |
| CTPS2 | inf | 0.450549451 | 1 | 0 |
| THBD | inf | 0.450549451 | 1 | 0 |
| CTSF | inf | 0.450549451 | 1 | 0 |
| THAP3 | inf | 0.450549451 | 1 | 0 |
| CRYL1 | inf | 0.450549451 | 1 | 0 |
| CRYGD | inf | 0.450549451 | 1 | 0 |
| CRYGA | inf | 0.450549451 | 1 | 0 |
| CRYBB1 | inf | 0.450549451 | 1 | 0 |
| CYP11B2 | inf | 0.450549451 | 1 | 0 |
| CPNE2 | inf | 0.450549451 | 1 | 0 |
| CPNE3 | inf | 0.450549451 | 1 | 0 |
| CPNE7 | inf | 0.450549451 | 1 | 0 |
| CPOX | inf | 0.450549451 | 1 | 0 |
| TM4SF1 | inf | 0.450549451 | 1 | 0 |
| TM2D3 | inf | 0.450549451 | 1 | 0 |
| CPSF3 | inf | 0.450549451 | 1 | 0 |
| TM2D2 | inf | 0.450549451 | 1 | 0 |
| CPSF4L | inf | 0.450549451 | 1 | 0 |
| TLX2 | inf | 0.450549451 | 1 | 0 |
| TM6SF2 | inf | 0.450549451 | 1 | 0 |
| TLX1 | inf | 0.450549451 | 1 | 0 |
| CREB3L1 | inf | 0.450549451 | 1 | 0 |
| CREB5 | inf | 0.450549451 | 1 | 0 |
| TLL1 | inf | 0.450549451 | 1 | 0 |
| CRISP1 | inf | 0.450549451 | 1 | 0 |
| TIMP3 | inf | 0.450549451 | 1 | 0 |
| CRTAC1 | inf | 0.450549451 | 1 | 0 |
| CRTC1 | inf | 0.450549451 | 1 | 0 |
| TIMM21 | inf | 0.450549451 | 1 | 0 |
| CRY1 | inf | 0.450549451 | 1 | 0 |
| TIMM17A | inf | 0.450549451 | 1 | 0 |
| TIMM10 | inf | 0.450549451 | 1 | 0 |
| CRADD | inf | 0.450549451 | 1 | 0 |
| GKN1 | inf | 0.450549451 | 1 | 0 |
| UFSP2 | inf | 0.450549451 | 1 | 0 |
| UGDH | inf | 0.450549451 | 1 | 0 |
| APOBEC3F | inf | 0.450549451 | 1 | 0 |
| ZNF219 | inf | 0.450549451 | 1 | 0 |
| APOLD1 | inf | 0.450549451 | 1 | 0 |
| APOPT1 | inf | 0.450549451 | 1 | 0 |
| ZNF211 | inf | 0.450549451 | 1 | 0 |
| ZNF19 | inf | 0.450549451 | 1 | 0 |
| ARAF | inf | 0.450549451 | 1 | 0 |
| ZNF17 | inf | 0.450549451 | 1 | 0 |
| AREG | inf | 0.450549451 | 1 | 0 |
| AREG | inf | 0.450549451 | 1 | 0 |
| ZNF160 | inf | 0.450549451 | 1 | 0 |
| ARGFX | inf | 0.450549451 | 1 | 0 |
| ARHGAP11A | inf | 0.450549451 | 1 | 0 |
| ZNF136 | inf | 0.450549451 | 1 | 0 |
| ZNF124 | inf | 0.450549451 | 1 | 0 |
| ZNF114 | inf | 0.450549451 | 1 | 0 |
| ZMYND12 | inf | 0.450549451 | 1 | 0 |
| ARHGAP4 | inf | 0.450549451 | 1 | 0 |
| ARHGAP44 | inf | 0.450549451 | 1 | 0 |
| ARHGAP5 | inf | 0.450549451 | 1 | 0 |
| ZMAT5 | inf | 0.450549451 | 1 | 0 |
| ZMAT3 | inf | 0.450549451 | 1 | 0 |
| ZMAT1 | inf | 0.450549451 | 1 | 0 |
| APOBEC3C | inf | 0.450549451 | 1 | 0 |
| ZKSCAN1 | inf | 0.450549451 | 1 | 0 |
| ZNF223 | inf | 0.450549451 | 1 | 0 |
| APOA4 | inf | 0.450549451 | 1 | 0 |
| ANKRD52 | inf | 0.450549451 | 1 | 0 |
| ZNF350 | inf | 0.450549451 | 1 | 0 |
| ANKRD61 | inf | 0.450549451 | 1 | 0 |
| ANKRD7 | inf | 0.450549451 | 1 | 0 |
| ANKUB1 | inf | 0.450549451 | 1 | 0 |
| ANO1 | inf | 0.450549451 | 1 | 0 |
| ZNF320 | inf | 0.450549451 | 1 | 0 |
| ZNF317 | inf | 0.450549451 | 1 | 0 |
| ZNF311 | inf | 0.450549451 | 1 | 0 |
| ANXA1 | inf | 0.450549451 | 1 | 0 |
| ANXA2 | inf | 0.450549451 | 1 | 0 |
| ZNF280A | inf | 0.450549451 | 1 | 0 |
| ZNF267 | inf | 0.450549451 | 1 | 0 |
| AP1G1 | inf | 0.450549451 | 1 | 0 |
| AP1G2 | inf | 0.450549451 | 1 | 0 |
| ZNF253 | inf | 0.450549451 | 1 | 0 |
| AP3S2 | inf | 0.450549451 | 1 | 0 |
| ZNF248 | inf | 0.450549451 | 1 | 0 |
| ZNF239 | inf | 0.450549451 | 1 | 0 |
| AP5S1 | inf | 0.450549451 | 1 | 0 |
| APCDD1 | inf | 0.450549451 | 1 | 0 |
| CENPS-CORT | inf | 0.450549451 | 1 | 0 |
| APOA2 | inf | 0.450549451 | 1 | 0 |
| APOA5 | inf | 0.450549451 | 1 | 0 |
| ZIC3 | inf | 0.450549451 | 1 | 0 |
| ZHX1 | inf | 0.450549451 | 1 | 0 |
| ZGPAT | inf | 0.450549451 | 1 | 0 |
| ZDHHC20 | inf | 0.450549451 | 1 | 0 |
| ZDHHC12 | inf | 0.450549451 | 1 | 0 |
| ASNA1 | inf | 0.450549451 | 1 | 0 |
| ASPA | inf | 0.450549451 | 1 | 0 |
| ASPN | inf | 0.450549451 | 1 | 0 |
| ZCCHC7 | inf | 0.450549451 | 1 | 0 |
| ATAD2 | inf | 0.450549451 | 1 | 0 |
| ZCCHC24 | inf | 0.450549451 | 1 | 0 |
| ZC4H2 | inf | 0.450549451 | 1 | 0 |
| ATF7 | inf | 0.450549451 | 1 | 0 |
| ZC3HC1 | inf | 0.450549451 | 1 | 0 |
| ATG13 | inf | 0.450549451 | 1 | 0 |
| ZC3H7A | inf | 0.450549451 | 1 | 0 |
| ATG4C | inf | 0.450549451 | 1 | 0 |
| ZC3H15 | inf | 0.450549451 | 1 | 0 |
| ZC2HC1B | inf | 0.450549451 | 1 | 0 |
| ZC2HC1A | inf | 0.450549451 | 1 | 0 |
| ATP11C | inf | 0.450549451 | 1 | 0 |
| ZBTB6 | inf | 0.450549451 | 1 | 0 |
| ZBTB5 | inf | 0.450549451 | 1 | 0 |
| ATP13A3 | inf | 0.450549451 | 1 | 0 |
| ZBTB49 | inf | 0.450549451 | 1 | 0 |
| ZBTB39 | inf | 0.450549451 | 1 | 0 |
| ASF1B | inf | 0.450549451 | 1 | 0 |
| ASCL4 | inf | 0.450549451 | 1 | 0 |
| ASB9 | inf | 0.450549451 | 1 | 0 |
| ASB5 | inf | 0.450549451 | 1 | 0 |
| ARL10 | inf | 0.450549451 | 1 | 0 |
| ZFYVE27 | inf | 0.450549451 | 1 | 0 |
| ARL2 | inf | 0.450549451 | 1 | 0 |
| ARL5B | inf | 0.450549451 | 1 | 0 |
| ARL5C | inf | 0.450549451 | 1 | 0 |
| ARL6IP4 | inf | 0.450549451 | 1 | 0 |
| ZFP62 | inf | 0.450549451 | 1 | 0 |
| ZFP3 | inf | 0.450549451 | 1 | 0 |
| ARMCX2 | inf | 0.450549451 | 1 | 0 |
| ZFP28 | inf | 0.450549451 | 1 | 0 |
| ZFP1 | inf | 0.450549451 | 1 | 0 |
| ANKRD37 | inf | 0.450549451 | 1 | 0 |
| ARRDC5 | inf | 0.450549451 | 1 | 0 |
| ARSG | inf | 0.450549451 | 1 | 0 |
| ZDHHC9 | inf | 0.450549451 | 1 | 0 |
| ZDHHC8 | inf | 0.450549451 | 1 | 0 |
| ART5 | inf | 0.450549451 | 1 | 0 |
| ZDHHC6 | inf | 0.450549451 | 1 | 0 |
| UBA2 | inf | 0.450549451 | 1 | 0 |
| ASAH1 | inf | 0.450549451 | 1 | 0 |
| ASB11 | inf | 0.450549451 | 1 | 0 |
| ZDHHC4 | inf | 0.450549451 | 1 | 0 |
| ASB17 | inf | 0.450549451 | 1 | 0 |
| ZDHHC22 | inf | 0.450549451 | 1 | 0 |
| ZFAND3 | inf | 0.450549451 | 1 | 0 |
| ZNF37A | inf | 0.450549451 | 1 | 0 |
| ANKRD34C | inf | 0.450549451 | 1 | 0 |
| ANKRD19P | inf | 0.450549451 | 1 | 0 |
| ACOXL | inf | 0.450549451 | 1 | 0 |
| ZNF816-ZNF321P | inf | 0.450549451 | 1 | 0 |
| ZNF812P | inf | 0.450549451 | 1 | 0 |
| ZNF793 | inf | 0.450549451 | 1 | 0 |
| ACTL6B | inf | 0.450549451 | 1 | 0 |
| ACTL7A | inf | 0.450549451 | 1 | 0 |
| ACTN4 | inf | 0.450549451 | 1 | 0 |
| ACTR1A | inf | 0.450549451 | 1 | 0 |
| ACTR2 | inf | 0.450549451 | 1 | 0 |
| ACTR3B | inf | 0.450549451 | 1 | 0 |
| ACTR5 | inf | 0.450549451 | 1 | 0 |
| ACTR8 | inf | 0.450549451 | 1 | 0 |
| ACVR1 | inf | 0.450549451 | 1 | 0 |
| ACVR1B | inf | 0.450549451 | 1 | 0 |
| ACY1 | inf | 0.450549451 | 1 | 0 |
| ADAD1 | inf | 0.450549451 | 1 | 0 |
| ADAL | inf | 0.450549451 | 1 | 0 |
| ADAM29 | inf | 0.450549451 | 1 | 0 |
| ZNF765 | inf | 0.450549451 | 1 | 0 |
| ADAMTS20 | inf | 0.450549451 | 1 | 0 |
| ADAMTSL2 | inf | 0.450549451 | 1 | 0 |
| ZNF75A | inf | 0.450549451 | 1 | 0 |
| ADCYAP1R1 | inf | 0.450549451 | 1 | 0 |
| ACOT9 | inf | 0.450549451 | 1 | 0 |
| ZNF830 | inf | 0.450549451 | 1 | 0 |
| ZNF836 | inf | 0.450549451 | 1 | 0 |
| ACER1 | inf | 0.450549451 | 1 | 0 |
| id189362 | inf | 0.450549451 | 1 | 0 |
| AADACL4 | inf | 0.450549451 | 1 | 0 |
| id118212 | inf | 0.450549451 | 1 | 0 |
| AAMP | inf | 0.450549451 | 1 | 0 |
| AANAT | inf | 0.450549451 | 1 | 0 |
| AARD | inf | 0.450549451 | 1 | 0 |
| ZYG11B | inf | 0.450549451 | 1 | 0 |
| ZXDB | inf | 0.450549451 | 1 | 0 |
| ZW10 | inf | 0.450549451 | 1 | 0 |
| ABCB5 | inf | 0.450549451 | 1 | 0 |
| ABHD10 | inf | 0.450549451 | 1 | 0 |
| ADH4 | inf | 0.450549451 | 1 | 0 |
| ZRANB2 | inf | 0.450549451 | 1 | 0 |
| ZRANB1 | inf | 0.450549451 | 1 | 0 |
| ABHD17B | inf | 0.450549451 | 1 | 0 |
| ABHD3 | inf | 0.450549451 | 1 | 0 |
| ZPLD1 | inf | 0.450549451 | 1 | 0 |
| ABI3 | inf | 0.450549451 | 1 | 0 |
| ZNHIT1 | inf | 0.450549451 | 1 | 0 |
| ABO | inf | 0.450549451 | 1 | 0 |
| ACAA2 | inf | 0.450549451 | 1 | 0 |
| ACADM | inf | 0.450549451 | 1 | 0 |
| ACADSB | inf | 0.450549451 | 1 | 0 |
| ACE2 | inf | 0.450549451 | 1 | 0 |
| ABHD14B | inf | 0.450549451 | 1 | 0 |
| ATP4B | inf | 0.450549451 | 1 | 0 |
| ADIPOQ | inf | 0.450549451 | 1 | 0 |
| ADPRHL1 | inf | 0.450549451 | 1 | 0 |
| AKR1D1 | inf | 0.450549451 | 1 | 0 |
| AKT3 | inf | 0.450549451 | 1 | 0 |
| ZNF549 | inf | 0.450549451 | 1 | 0 |
| ALDH1A3 | inf | 0.450549451 | 1 | 0 |
| ALDH2 | inf | 0.450549451 | 1 | 0 |
| ZNF521 | inf | 0.450549451 | 1 | 0 |
| ALG1L2 | inf | 0.450549451 | 1 | 0 |
| ZNF501 | inf | 0.450549451 | 1 | 0 |
| ALG8 | inf | 0.450549451 | 1 | 0 |
| ALKBH4 | inf | 0.450549451 | 1 | 0 |
| ZNF490 | inf | 0.450549451 | 1 | 0 |
| ALPP | inf | 0.450549451 | 1 | 0 |
| ALYREF | inf | 0.450549451 | 1 | 0 |
| ZNF45 | inf | 0.450549451 | 1 | 0 |
| AMFR | inf | 0.450549451 | 1 | 0 |
| AMIGO1 | inf | 0.450549451 | 1 | 0 |
| AMIGO2 | inf | 0.450549451 | 1 | 0 |
| ZNF441 | inf | 0.450549451 | 1 | 0 |
| ANAPC2 | inf | 0.450549451 | 1 | 0 |
| ANGPTL2 | inf | 0.450549451 | 1 | 0 |
| ZNF43 | inf | 0.450549451 | 1 | 0 |
| ANGPTL6 | inf | 0.450549451 | 1 | 0 |
| ANKRD13C | inf | 0.450549451 | 1 | 0 |
| AKR1C3 | inf | 0.450549451 | 1 | 0 |
| ZNF559 | inf | 0.450549451 | 1 | 0 |
| ZNF561 | inf | 0.450549451 | 1 | 0 |
| ZNF566 | inf | 0.450549451 | 1 | 0 |
| ADPRHL2 | inf | 0.450549451 | 1 | 0 |
| ADRA1D | inf | 0.450549451 | 1 | 0 |
| ZNF704 | inf | 0.450549451 | 1 | 0 |
| ADRB2 | inf | 0.450549451 | 1 | 0 |
| AEBP2 | inf | 0.450549451 | 1 | 0 |
| ZNF695 | inf | 0.450549451 | 1 | 0 |
| ZNF688 | inf | 0.450549451 | 1 | 0 |
| ZNF670 | inf | 0.450549451 | 1 | 0 |
| ZNF667 | inf | 0.450549451 | 1 | 0 |
| ZNF629 | inf | 0.450549451 | 1 | 0 |
| ZNF627 | inf | 0.450549451 | 1 | 0 |
| ADORA3 | inf | 0.450549451 | 1 | 0 |
| ZNF626 | inf | 0.450549451 | 1 | 0 |
| AGR3 | inf | 0.450549451 | 1 | 0 |
| AGTRAP | inf | 0.450549451 | 1 | 0 |
| AIF1L | inf | 0.450549451 | 1 | 0 |
| AIPL1 | inf | 0.450549451 | 1 | 0 |
| ZNF578 | inf | 0.450549451 | 1 | 0 |
| ZNF577 | inf | 0.450549451 | 1 | 0 |
| AKAP10 | inf | 0.450549451 | 1 | 0 |
| ZNF575 | inf | 0.450549451 | 1 | 0 |
| AKAP2 | inf | 0.450549451 | 1 | 0 |
| ZNF570 | inf | 0.450549451 | 1 | 0 |
| ZNF569 | inf | 0.450549451 | 1 | 0 |
| AGPAT5 | inf | 0.450549451 | 1 | 0 |
| DIO1 | inf | 0.450549451 | 1 | 0 |
| ZBTB37 | inf | 0.450549451 | 1 | 0 |
| ZBTB33 | inf | 0.450549451 | 1 | 0 |
| SMIM24 | inf | 0.450549451 | 1 | 0 |
| ANGPTL8 | inf | 0.450549451 | 1 | 0 |
| C19orf81 | inf | 0.450549451 | 1 | 0 |
| C1QL1 | inf | 0.450549451 | 1 | 0 |
| C1QTNF6 | inf | 0.450549451 | 1 | 0 |
| UTP23 | inf | 0.450549451 | 1 | 0 |
| UTP14A | inf | 0.450549451 | 1 | 0 |
| USP9X | inf | 0.450549451 | 1 | 0 |
| SPATA46 | inf | 0.450549451 | 1 | 0 |
| C1orf141 | inf | 0.450549451 | 1 | 0 |
| USP47 | inf | 0.450549451 | 1 | 0 |
| USP46 | inf | 0.450549451 | 1 | 0 |
| USP45 | inf | 0.450549451 | 1 | 0 |
| LEXM | inf | 0.450549451 | 1 | 0 |
| C1orf185 | inf | 0.450549451 | 1 | 0 |
| C1orf50 | inf | 0.450549451 | 1 | 0 |
| CCDC185 | inf | 0.450549451 | 1 | 0 |
| NOL4L | inf | 0.450549451 | 1 | 0 |
| C20orf141 | inf | 0.450549451 | 1 | 0 |
| C20orf173 | inf | 0.450549451 | 1 | 0 |
| FNDC11 | inf | 0.450549451 | 1 | 0 |
| C20orf196 | inf | 0.450549451 | 1 | 0 |
| C20orf24 | inf | 0.450549451 | 1 | 0 |
| C19orf66 | inf | 0.450549451 | 1 | 0 |
| C20orf27 | inf | 0.450549451 | 1 | 0 |
| REX1BD | inf | 0.450549451 | 1 | 0 |
| VAT1 | inf | 0.450549451 | 1 | 0 |
| VPS25 | inf | 0.450549451 | 1 | 0 |
| C12orf80 | inf | 0.450549451 | 1 | 0 |
| C14orf132 | inf | 0.450549451 | 1 | 0 |
| C14orf177 | inf | 0.450549451 | 1 | 0 |
| C14orf28 | inf | 0.450549451 | 1 | 0 |
| C14orf93 | inf | 0.450549451 | 1 | 0 |
| C15orf38-AP3S2 | inf | 0.450549451 | 1 | 0 |
| VMAC | inf | 0.450549451 | 1 | 0 |
| C15orf41 | inf | 0.450549451 | 1 | 0 |
| C15orf52 | inf | 0.450549451 | 1 | 0 |
| C15orf53 | inf | 0.450549451 | 1 | 0 |
| VIPR1 | inf | 0.450549451 | 1 | 0 |
| C16orf74 | inf | 0.450549451 | 1 | 0 |
| VGLL3 | inf | 0.450549451 | 1 | 0 |
| C17orf105 | inf | 0.450549451 | 1 | 0 |
| BORCS6 | inf | 0.450549451 | 1 | 0 |
| C17orf82 | inf | 0.450549451 | 1 | 0 |
| NCBP3 | inf | 0.450549451 | 1 | 0 |
| EPOP | inf | 0.450549451 | 1 | 0 |
| C19orf18 | inf | 0.450549451 | 1 | 0 |
| C19orf25 | inf | 0.450549451 | 1 | 0 |
| PEAK3 | inf | 0.450549451 | 1 | 0 |
| C19orf38 | inf | 0.450549451 | 1 | 0 |
| VANGL2 | inf | 0.450549451 | 1 | 0 |
| C20orf96 | inf | 0.450549451 | 1 | 0 |
| USP22 | inf | 0.450549451 | 1 | 0 |
| C22orf15 | inf | 0.450549451 | 1 | 0 |
| UPK3A | inf | 0.450549451 | 1 | 0 |
| C5orf63 | inf | 0.450549451 | 1 | 0 |
| C5orf64 | inf | 0.450549451 | 1 | 0 |
| UPK2 | inf | 0.450549451 | 1 | 0 |
| C6orf10 | inf | 0.450549451 | 1 | 0 |
| UPK1A | inf | 0.450549451 | 1 | 0 |
| UNC93A | inf | 0.450549451 | 1 | 0 |
| C7orf26 | inf | 0.450549451 | 1 | 0 |
| FMC1 | inf | 0.450549451 | 1 | 0 |
| C7orf55-LUC7L2 | inf | 0.450549451 | 1 | 0 |
| C7orf57 | inf | 0.450549451 | 1 | 0 |
| ULK3 | inf | 0.450549451 | 1 | 0 |
| ULK2 | inf | 0.450549451 | 1 | 0 |
| C8orf34 | inf | 0.450549451 | 1 | 0 |
| C8orf37 | inf | 0.450549451 | 1 | 0 |
| ULBP2 | inf | 0.450549451 | 1 | 0 |
| C8orf48 | inf | 0.450549451 | 1 | 0 |
| C8orf86 | inf | 0.450549451 | 1 | 0 |
| UHRF1BP1L | inf | 0.450549451 | 1 | 0 |
| PAXX | inf | 0.450549451 | 1 | 0 |
| CARNMT1 | inf | 0.450549451 | 1 | 0 |
| C9orf62 | inf | 0.450549451 | 1 | 0 |
| C9orf72 | inf | 0.450549451 | 1 | 0 |
| UPK3B | inf | 0.450549451 | 1 | 0 |
| C5orf47 | inf | 0.450549451 | 1 | 0 |
| C5orf46 | inf | 0.450549451 | 1 | 0 |
| UPP2 | inf | 0.450549451 | 1 | 0 |
| C2CD2L | inf | 0.450549451 | 1 | 0 |
| C2CD4B | inf | 0.450549451 | 1 | 0 |
| C2CD5 | inf | 0.450549451 | 1 | 0 |
| LDAH | inf | 0.450549451 | 1 | 0 |
| C2orf48 | inf | 0.450549451 | 1 | 0 |
| PRR30 | inf | 0.450549451 | 1 | 0 |
| C2orf54 | inf | 0.450549451 | 1 | 0 |
| C2orf69 | inf | 0.450549451 | 1 | 0 |
| C2orf82 | inf | 0.450549451 | 1 | 0 |
| C2orf91 | inf | 0.450549451 | 1 | 0 |
| C3orf18 | inf | 0.450549451 | 1 | 0 |
| VPS26A | inf | 0.450549451 | 1 | 0 |
| LINC01565 | inf | 0.450549451 | 1 | 0 |
| SMCO1 | inf | 0.450549451 | 1 | 0 |
| C3orf52 | inf | 0.450549451 | 1 | 0 |
| C3orf62 | inf | 0.450549451 | 1 | 0 |
| USO1 | inf | 0.450549451 | 1 | 0 |
| C3orf79 | inf | 0.450549451 | 1 | 0 |
| USH1G | inf | 0.450549451 | 1 | 0 |
| USE1 | inf | 0.450549451 | 1 | 0 |
| C4orf22 | inf | 0.450549451 | 1 | 0 |
| USB1 | inf | 0.450549451 | 1 | 0 |
| C4orf45 | inf | 0.450549451 | 1 | 0 |
| TMEM267 | inf | 0.450549451 | 1 | 0 |
| C3orf33 | inf | 0.450549451 | 1 | 0 |
| LINC01465 | inf | 0.450549451 | 1 | 0 |
| C12orf60 | inf | 0.450549451 | 1 | 0 |
| C12orf56 | inf | 0.450549451 | 1 | 0 |
| BBS5 | inf | 0.450549451 | 1 | 0 |
| BCAP31 | inf | 0.450549451 | 1 | 0 |
| XCL1 | inf | 0.450549451 | 1 | 0 |
| WTH3DI | inf | 0.450549451 | 1 | 0 |
| WSCD2 | inf | 0.450549451 | 1 | 0 |
| BCKDHA | inf | 0.450549451 | 1 | 0 |
| BCL10 | inf | 0.450549451 | 1 | 0 |
| BCL2A1 | inf | 0.450549451 | 1 | 0 |
| WSB2 | inf | 0.450549451 | 1 | 0 |
| BCL2L10 | inf | 0.450549451 | 1 | 0 |
| WSB1 | inf | 0.450549451 | 1 | 0 |
| WNT9A | inf | 0.450549451 | 1 | 0 |
| BCL6B | inf | 0.450549451 | 1 | 0 |
| BCL7C | inf | 0.450549451 | 1 | 0 |
| WNT6 | inf | 0.450549451 | 1 | 0 |
| BDH1 | inf | 0.450549451 | 1 | 0 |
| BDKRB1 | inf | 0.450549451 | 1 | 0 |
| BDKRB2 | inf | 0.450549451 | 1 | 0 |
| WNT4 | inf | 0.450549451 | 1 | 0 |
| WNT3A | inf | 0.450549451 | 1 | 0 |
| BEST3 | inf | 0.450549451 | 1 | 0 |
| BET1 | inf | 0.450549451 | 1 | 0 |
| BGLAP | inf | 0.450549451 | 1 | 0 |
| XKR8 | inf | 0.450549451 | 1 | 0 |
| BBIP1 | inf | 0.450549451 | 1 | 0 |
| XPO4 | inf | 0.450549451 | 1 | 0 |
| XPO5 | inf | 0.450549451 | 1 | 0 |
| ATP5G3 | inf | 0.450549451 | 1 | 0 |
| ATP6V1C1 | inf | 0.450549451 | 1 | 0 |
| ATP6V1G1 | inf | 0.450549451 | 1 | 0 |
| ATP7A | inf | 0.450549451 | 1 | 0 |
| YTHDF3 | inf | 0.450549451 | 1 | 0 |
| ATRX | inf | 0.450549451 | 1 | 0 |
| YTHDF2 | inf | 0.450549451 | 1 | 0 |
| YRDC | inf | 0.450549451 | 1 | 0 |
| ATXN7L3B | inf | 0.450549451 | 1 | 0 |
| AVPR1A | inf | 0.450549451 | 1 | 0 |
| YIF1B | inf | 0.450549451 | 1 | 0 |
| BHLHA15 | inf | 0.450549451 | 1 | 0 |
| AZGP1 | inf | 0.450549451 | 1 | 0 |
| AZU1 | inf | 0.450549451 | 1 | 0 |
| B3GALNT1 | inf | 0.450549451 | 1 | 0 |
| B3GALT1 | inf | 0.450549451 | 1 | 0 |
| B3GALT5 | inf | 0.450549451 | 1 | 0 |
| B3GAT1 | inf | 0.450549451 | 1 | 0 |
| YBX1 | inf | 0.450549451 | 1 | 0 |
| B4GALT2 | inf | 0.450549451 | 1 | 0 |
| BABAM1 | inf | 0.450549451 | 1 | 0 |
| BAG1 | inf | 0.450549451 | 1 | 0 |
| BAG5 | inf | 0.450549451 | 1 | 0 |
| BAIAP2 | inf | 0.450549451 | 1 | 0 |
| YIF1A | inf | 0.450549451 | 1 | 0 |
| ATP5D | inf | 0.450549451 | 1 | 0 |
| BHMT | inf | 0.450549451 | 1 | 0 |
| PYM1 | inf | 0.450549451 | 1 | 0 |
| WASF1 | inf | 0.450549451 | 1 | 0 |
| WAS | inf | 0.450549451 | 1 | 0 |
| C10orf111 | inf | 0.450549451 | 1 | 0 |
| ACSM6 | inf | 0.450549451 | 1 | 0 |
| INTS14 | inf | 0.450549451 | 1 | 0 |
| C10orf53 | inf | 0.450549451 | 1 | 0 |
| C10orf62 | inf | 0.450549451 | 1 | 0 |
| C10orf67 | inf | 0.450549451 | 1 | 0 |
| C10orf91 | inf | 0.450549451 | 1 | 0 |
| VTI1B | inf | 0.450549451 | 1 | 0 |
| VTI1A | inf | 0.450549451 | 1 | 0 |
| VSX2 | inf | 0.450549451 | 1 | 0 |
| VSTM5 | inf | 0.450549451 | 1 | 0 |
| VSIG8 | inf | 0.450549451 | 1 | 0 |
| VSIG1 | inf | 0.450549451 | 1 | 0 |
| C11orf84 | inf | 0.450549451 | 1 | 0 |
| VPS54 | inf | 0.450549451 | 1 | 0 |
| VPS52 | inf | 0.450549451 | 1 | 0 |
| VPS51 | inf | 0.450549451 | 1 | 0 |
| VPS39 | inf | 0.450549451 | 1 | 0 |
| VPS37B | inf | 0.450549451 | 1 | 0 |
| C12orf42 | inf | 0.450549451 | 1 | 0 |
| C12orf43 | inf | 0.450549451 | 1 | 0 |
| BUD13 | inf | 0.450549451 | 1 | 0 |
| WBP1L | inf | 0.450549451 | 1 | 0 |
| BTN3A3 | inf | 0.450549451 | 1 | 0 |
| BTN3A2 | inf | 0.450549451 | 1 | 0 |
| WFDC6 | inf | 0.450549451 | 1 | 0 |
| BLOC1S4 | inf | 0.450549451 | 1 | 0 |
| WFDC2 | inf | 0.450549451 | 1 | 0 |
| BMP3 | inf | 0.450549451 | 1 | 0 |
| WDTC1 | inf | 0.450549451 | 1 | 0 |
| BMPR1A | inf | 0.450549451 | 1 | 0 |
| WDR92 | inf | 0.450549451 | 1 | 0 |
| BNIP1 | inf | 0.450549451 | 1 | 0 |
| WDR78 | inf | 0.450549451 | 1 | 0 |
| WDR77 | inf | 0.450549451 | 1 | 0 |
| BPIFA2 | inf | 0.450549451 | 1 | 0 |
| WISP3 | inf | 0.450549451 | 1 | 0 |
| BPIFB2 | inf | 0.450549451 | 1 | 0 |
| WDR7 | inf | 0.450549451 | 1 | 0 |
| DEPDC1B | inf | 0.450549451 | 1 | 0 |
| WDR46 | inf | 0.450549451 | 1 | 0 |
| WDR44 | inf | 0.450549451 | 1 | 0 |
| BRIX1 | inf | 0.450549451 | 1 | 0 |
| BRSK2 | inf | 0.450549451 | 1 | 0 |
| WDR20 | inf | 0.450549451 | 1 | 0 |
| WDFY2 | inf | 0.450549451 | 1 | 0 |
| BTC | inf | 0.450549451 | 1 | 0 |
| WBP4 | inf | 0.450549451 | 1 | 0 |
| BTN3A1 | inf | 0.450549451 | 1 | 0 |
| BPIFC | inf | 0.450549451 | 1 | 0 |
| DIO2 | inf | 0.450549451 | 1 | 0 |
| CCNJL | inf | 0.450549451 | 1 | 0 |
| ELP5 | inf | 0.450549451 | 1 | 0 |
| DTX2P1-UPK3BP1-PMS2P11 | inf | 0.450549451 | 1 | 0 |
| FAIM2 | inf | 0.450549451 | 1 | 0 |
| DTX2 | inf | 0.450549451 | 1 | 0 |
| FAM104B | inf | 0.450549451 | 1 | 0 |
| SUPT7L | inf | 0.450549451 | 1 | 0 |
| SPAG11A | inf | 0.450549451 | 1 | 0 |
| DTNBP1 | inf | 0.450549451 | 1 | 0 |
| DTNB | inf | 0.450549451 | 1 | 0 |
| DTNA | inf | 0.450549451 | 1 | 0 |
| SUPT5H | inf | 0.450549451 | 1 | 0 |
| DTD2 | inf | 0.450549451 | 1 | 0 |
| FAM109A | inf | 0.450549451 | 1 | 0 |
| FAM109B | inf | 0.450549451 | 1 | 0 |
| SUSD5 | inf | 0.450549451 | 1 | 0 |
| SPA17 | inf | 0.450549451 | 1 | 0 |
| DSCC1 | inf | 0.450549451 | 1 | 0 |
| DSC3 | inf | 0.450549451 | 1 | 0 |
| FAM120C | inf | 0.450549451 | 1 | 0 |
| DRAP1 | inf | 0.450549451 | 1 | 0 |
| FAM122C | inf | 0.450549451 | 1 | 0 |
| SPACA3 | inf | 0.450549451 | 1 | 0 |
| FANCA | inf | 0.450549451 | 1 | 0 |
| DTYMK | inf | 0.450549451 | 1 | 0 |
| SUPT3H | inf | 0.450549451 | 1 | 0 |
| SPATA31A5 | inf | 0.450549451 | 1 | 0 |
| EXT1 | inf | 0.450549451 | 1 | 0 |
| DYNC2LI1 | inf | 0.450549451 | 1 | 0 |
| SUGP1 | inf | 0.450549451 | 1 | 0 |
| SPATA25 | inf | 0.450549451 | 1 | 0 |
| EZH2 | inf | 0.450549451 | 1 | 0 |
| ZNF160 | inf | 0.450549451 | 1 | 0 |
| DUXA | inf | 0.450549451 | 1 | 0 |
| F2RL1 | inf | 0.450549451 | 1 | 0 |
| F2RL2 | inf | 0.450549451 | 1 | 0 |
| EMX2 | inf | 0.450549451 | 1 | 0 |
| SULT1B1 | inf | 0.450549451 | 1 | 0 |
| SPANXN2 | inf | 0.450549451 | 1 | 0 |
| FABP6 | inf | 0.450549451 | 1 | 0 |
| FABP7 | inf | 0.450549451 | 1 | 0 |
| FADS2 | inf | 0.450549451 | 1 | 0 |
| FAF1 | inf | 0.450549451 | 1 | 0 |
| FAF2 | inf | 0.450549451 | 1 | 0 |
| DUS4L | inf | 0.450549451 | 1 | 0 |
| SUOX | inf | 0.450549451 | 1 | 0 |
| DUPD1 | inf | 0.450549451 | 1 | 0 |
| DRAM1 | inf | 0.450549451 | 1 | 0 |
| DYRK3 | inf | 0.450549451 | 1 | 0 |
| SOX21 | inf | 0.450549451 | 1 | 0 |
| SOX2 | inf | 0.450549451 | 1 | 0 |
| FAM167B | inf | 0.450549451 | 1 | 0 |
| FAM168B | inf | 0.450549451 | 1 | 0 |
| SNX5 | inf | 0.450549451 | 1 | 0 |
| DNAJC28 | inf | 0.450549451 | 1 | 0 |
| SNX24 | inf | 0.450549451 | 1 | 0 |
| TAB2 | inf | 0.450549451 | 1 | 0 |
| DNAJC19 | inf | 0.450549451 | 1 | 0 |
| TAB3 | inf | 0.450549451 | 1 | 0 |
| TAC3 | inf | 0.450549451 | 1 | 0 |
| DNAJC3 | inf | 0.450549451 | 1 | 0 |
| FAM181A | inf | 0.450549451 | 1 | 0 |
| SNX16 | inf | 0.450549451 | 1 | 0 |
| SNX13 | inf | 0.450549451 | 1 | 0 |
| TACR1 | inf | 0.450549451 | 1 | 0 |
| TAF1 | inf | 0.450549451 | 1 | 0 |
| SNTB2 | inf | 0.450549451 | 1 | 0 |
| DNAJB4 | inf | 0.450549451 | 1 | 0 |
| DNAJA2 | inf | 0.450549451 | 1 | 0 |
| SNTA1 | inf | 0.450549451 | 1 | 0 |
| DNAJA1 | inf | 0.450549451 | 1 | 0 |
| DNAJC12 | inf | 0.450549451 | 1 | 0 |
| SYTL4 | inf | 0.450549451 | 1 | 0 |
| DNM1L | inf | 0.450549451 | 1 | 0 |
| FAM166A | inf | 0.450549451 | 1 | 0 |
| SWSAP1 | inf | 0.450549451 | 1 | 0 |
| FAM126A | inf | 0.450549451 | 1 | 0 |
| SYCP3 | inf | 0.450549451 | 1 | 0 |
| DPPA4 | inf | 0.450549451 | 1 | 0 |
| SYNCRIP | inf | 0.450549451 | 1 | 0 |
| DPH6 | inf | 0.450549451 | 1 | 0 |
| SOX10 | inf | 0.450549451 | 1 | 0 |
| DONSON | inf | 0.450549451 | 1 | 0 |
| SYPL2 | inf | 0.450549451 | 1 | 0 |
| DOLK | inf | 0.450549451 | 1 | 0 |
| SYT10 | inf | 0.450549451 | 1 | 0 |
| SYT11 | inf | 0.450549451 | 1 | 0 |
| RETREG3 | inf | 0.450549451 | 1 | 0 |
| DOK1 | inf | 0.450549451 | 1 | 0 |
| ALKAL2 | inf | 0.450549451 | 1 | 0 |
| SAXO2 | inf | 0.450549451 | 1 | 0 |
| DOC2A | inf | 0.450549451 | 1 | 0 |
| DNTTIP1 | inf | 0.450549451 | 1 | 0 |
| FAM157A | inf | 0.450549451 | 1 | 0 |
| FAM160A2 | inf | 0.450549451 | 1 | 0 |
| SOAT2 | inf | 0.450549451 | 1 | 0 |
| DPYSL5 | inf | 0.450549451 | 1 | 0 |
| ERICH6 | inf | 0.450549451 | 1 | 0 |
| EXOSC5 | inf | 0.450549451 | 1 | 0 |
| SPATA31D4 | inf | 0.450549451 | 1 | 0 |
| ST6GALNAC2 | inf | 0.450549451 | 1 | 0 |
| EPN2 | inf | 0.450549451 | 1 | 0 |
| ST6GALNAC3 | inf | 0.450549451 | 1 | 0 |
| ST6GALNAC4 | inf | 0.450549451 | 1 | 0 |
| TIMP1 | inf | 0.450549451 | 1 | 0 |
| SPRY4 | inf | 0.450549451 | 1 | 0 |
| ST6GALNAC6 | inf | 0.450549451 | 1 | 0 |
| EPS15 | inf | 0.450549451 | 1 | 0 |
| ST7 | inf | 0.450549451 | 1 | 0 |
| EIF5A | inf | 0.450549451 | 1 | 0 |
| ST7L | inf | 0.450549451 | 1 | 0 |
| EPS8L2 | inf | 0.450549451 | 1 | 0 |
| SPRED3 | inf | 0.450549451 | 1 | 0 |
| SPRED2 | inf | 0.450549451 | 1 | 0 |
| EIF3D | inf | 0.450549451 | 1 | 0 |
| EQTN | inf | 0.450549451 | 1 | 0 |
| EIF2D | inf | 0.450549451 | 1 | 0 |
| ERBB4 | inf | 0.450549451 | 1 | 0 |
| STAT1 | inf | 0.450549451 | 1 | 0 |
| ERCC3 | inf | 0.450549451 | 1 | 0 |
| EIF4E1B | inf | 0.450549451 | 1 | 0 |
| EPHA3 | inf | 0.450549451 | 1 | 0 |
| EPCAM | inf | 0.450549451 | 1 | 0 |
| EPC1 | inf | 0.450549451 | 1 | 0 |
| ENC1 | inf | 0.450549451 | 1 | 0 |
| SRSF6 | inf | 0.450549451 | 1 | 0 |
| ENO1 | inf | 0.450549451 | 1 | 0 |
| ENO3 | inf | 0.450549451 | 1 | 0 |
| ELSPBP1 | inf | 0.450549451 | 1 | 0 |
| ENOPH1 | inf | 0.450549451 | 1 | 0 |
| ELOVL7 | inf | 0.450549451 | 1 | 0 |
| ELOVL2&ELOVL2-AS1 | inf | 0.450549451 | 1 | 0 |
| ENOX2 | inf | 0.450549451 | 1 | 0 |
| SSR1 | inf | 0.450549451 | 1 | 0 |
| SSTR4 | inf | 0.450549451 | 1 | 0 |
| SSTR5 | inf | 0.450549451 | 1 | 0 |
| SSUH2 | inf | 0.450549451 | 1 | 0 |
| SSX7 | inf | 0.450549451 | 1 | 0 |
| ENTPD4 | inf | 0.450549451 | 1 | 0 |
| ST20 | inf | 0.450549451 | 1 | 0 |
| ELAC1 | inf | 0.450549451 | 1 | 0 |
| ENY2 | inf | 0.450549451 | 1 | 0 |
| EOGT | inf | 0.450549451 | 1 | 0 |
| EOMES | inf | 0.450549451 | 1 | 0 |
| EIF5B | inf | 0.450549451 | 1 | 0 |
| ERG | inf | 0.450549451 | 1 | 0 |
| EXOG | inf | 0.450549451 | 1 | 0 |
| STAT2 | inf | 0.450549451 | 1 | 0 |
| STAT4 | inf | 0.450549451 | 1 | 0 |
| SPECC1 | inf | 0.450549451 | 1 | 0 |
| STPG2 | inf | 0.450549451 | 1 | 0 |
| EDN2 | inf | 0.450549451 | 1 | 0 |
| ETNPPL | inf | 0.450549451 | 1 | 0 |
| STRBP | inf | 0.450549451 | 1 | 0 |
| EDEM1 | inf | 0.450549451 | 1 | 0 |
| EDDM3B | inf | 0.450549451 | 1 | 0 |
| SPDYA | inf | 0.450549451 | 1 | 0 |
| EVA1A | inf | 0.450549451 | 1 | 0 |
| EEF1E1 | inf | 0.450549451 | 1 | 0 |
| SPCS1 | inf | 0.450549451 | 1 | 0 |
| STRIP1 | inf | 0.450549451 | 1 | 0 |
| STRN4 | inf | 0.450549451 | 1 | 0 |
| ECHDC1 | inf | 0.450549451 | 1 | 0 |
| EVX1 | inf | 0.450549451 | 1 | 0 |
| SPATS2 | inf | 0.450549451 | 1 | 0 |
| SPATA6 | inf | 0.450549451 | 1 | 0 |
| EXO5 | inf | 0.450549451 | 1 | 0 |
| EAPP | inf | 0.450549451 | 1 | 0 |
| STX1A | inf | 0.450549451 | 1 | 0 |
| EDA2R | inf | 0.450549451 | 1 | 0 |
| EEF2 | inf | 0.450549451 | 1 | 0 |
| ESRRB | inf | 0.450549451 | 1 | 0 |
| ESRRA | inf | 0.450549451 | 1 | 0 |
| SPP1 | inf | 0.450549451 | 1 | 0 |
| EHD4 | inf | 0.450549451 | 1 | 0 |
| SPOCD1 | inf | 0.450549451 | 1 | 0 |
| SPNS3 | inf | 0.450549451 | 1 | 0 |
| ERLEC1 | inf | 0.450549451 | 1 | 0 |
| ERMN | inf | 0.450549451 | 1 | 0 |
| SPINK8 | inf | 0.450549451 | 1 | 0 |
| ERO1A | inf | 0.450549451 | 1 | 0 |
| ERP27 | inf | 0.450549451 | 1 | 0 |
| STAU2 | inf | 0.450549451 | 1 | 0 |
| ERV3-1 | inf | 0.450549451 | 1 | 0 |
| SPINK2 | inf | 0.450549451 | 1 | 0 |
| STK25 | inf | 0.450549451 | 1 | 0 |
| EFNA3 | inf | 0.450549451 | 1 | 0 |
| ERVV-1 | inf | 0.450549451 | 1 | 0 |
| EFHB | inf | 0.450549451 | 1 | 0 |
| STK24 | inf | 0.450549451 | 1 | 0 |
| STK32A | inf | 0.450549451 | 1 | 0 |
| EFCAB3 | inf | 0.450549451 | 1 | 0 |
| ESR1 | inf | 0.450549451 | 1 | 0 |
| EFCAB11 | inf | 0.450549451 | 1 | 0 |
| ERGIC1 | inf | 0.450549451 | 1 | 0 |
| TAF3 | inf | 0.450549451 | 1 | 0 |
| SULT1A3 | inf | 0.450549451 | 1 | 0 |
| FAM204A | inf | 0.450549451 | 1 | 0 |
| SNRNP27 | inf | 0.450549451 | 1 | 0 |
| DMTF1 | inf | 0.450549451 | 1 | 0 |
| FAM200A | inf | 0.450549451 | 1 | 0 |
| SNRNP25 | inf | 0.450549451 | 1 | 0 |
| DKK2 | inf | 0.450549451 | 1 | 0 |
| DLX5 | inf | 0.450549451 | 1 | 0 |
| HGH1 | inf | 0.450549451 | 1 | 0 |
| DLST | inf | 0.450549451 | 1 | 0 |
| TARBP2 | inf | 0.450549451 | 1 | 0 |
| FAM198A | inf | 0.450549451 | 1 | 0 |
| DIRC2 | inf | 0.450549451 | 1 | 0 |
| SNRNP70 | inf | 0.450549451 | 1 | 0 |
| TAGAP | inf | 0.450549451 | 1 | 0 |
| MPP3 | inf | 0.450549451 | 1 | 0 |
| TARM1 | inf | 0.450549451 | 1 | 0 |
| FAM210A | inf | 0.450549451 | 1 | 0 |
| TAF9 | inf | 0.450549451 | 1 | 0 |
| LRRC75A | inf | 0.450549451 | 1 | 0 |
| DMRTC1 | inf | 0.450549451 | 1 | 0 |
| TENM4 | 1.24 | 0.454273764 | 7 | 7 |
| FREM1 | 1.24 | 0.454273764 | 7 | 7 |
| APOB | 1.24 | 0.454273764 | 7 | 7 |
| TLN2 | 1.24 | 0.454273764 | 7 | 7 |
| RNF213 | 1.24 | 0.454273764 | 7 | 7 |
| VWF | 1.24 | 0.454273764 | 7 | 7 |
| MLST8 | 1.139037433 | 0.465760304 | 71 | 85 |
| LFNG | 1.19047619 | 0.470423211 | 75 | 90 |
| OR4F21 | 1.071428571 | 0.472871315 | 30 | 35 |
| XIRP2 | 1.136150235 | 0.473282246 | 11 | 12 |
| CCDC168 | 1.136150235 | 0.473282246 | 11 | 12 |
| DSP | 1.236842105 | 0.474258135 | 6 | 6 |
| XIRP1 | 1.236842105 | 0.474258135 | 6 | 6 |
| ANK2 | 1.236842105 | 0.474258135 | 6 | 6 |
| FAT2 | 1.236842105 | 0.474258135 | 6 | 6 |
| TTC28 | 1.236842105 | 0.474258135 | 6 | 6 |
| SYT15 | 1.236842105 | 0.474258135 | 6 | 6 |
| UNC79 | 1.236842105 | 0.474258135 | 6 | 6 |
| VWDE | 1.236842105 | 0.474258135 | 6 | 6 |
| DNAH8 | 1.236842105 | 0.474258135 | 6 | 6 |
| LTBP4 | 1.236842105 | 0.474258135 | 6 | 6 |
| VPS13D | 1.236842105 | 0.474258135 | 6 | 6 |
| ZNF678 | 1.236842105 | 0.474258135 | 6 | 6 |
| MBD3L5 | 1.093064091 | 0.485466516 | 15 | 17 |
| CRIPAK | 1.123737374 | 0.490458528 | 10 | 11 |
| NPIPB15 | 1.123737374 | 0.490458528 | 10 | 11 |
| PRAMEF10 | 1.123737374 | 0.490458528 | 10 | 11 |
| POTEM | 1.069264069 | 0.494791534 | 19 | 22 |
| IGHV3-11 | 1.051824432 | 0.495773297 | 51 | 61 |
| NOTCH1 | 1.233766234 | 0.497426817 | 5 | 5 |
| HLA-DQB1 | 1.233766234 | 0.497426817 | 5 | 5 |
| CC2D1A | 1.233766234 | 0.497426817 | 5 | 5 |
| TG | 1.233766234 | 0.497426817 | 5 | 5 |
| PKD1L3 | 1.233766234 | 0.497426817 | 5 | 5 |
| THADA | 1.233766234 | 0.497426817 | 5 | 5 |
| PLXNA3 | 1.233766234 | 0.497426817 | 5 | 5 |
| FN1 | 1.233766234 | 0.497426817 | 5 | 5 |
| UBR4 | 1.233766234 | 0.497426817 | 5 | 5 |
| PTPRB | 1.233766234 | 0.497426817 | 5 | 5 |
| FAM208B | 1.233766234 | 0.497426817 | 5 | 5 |
| COL6A3 | 1.233766234 | 0.497426817 | 5 | 5 |
| SLC4A3 | 1.233766234 | 0.497426817 | 5 | 5 |
| A2ML1 | 1.233766234 | 0.497426817 | 5 | 5 |
| PDPR | 1.233766234 | 0.497426817 | 5 | 5 |
| ABCC2 | 1.233766234 | 0.497426817 | 5 | 5 |
| AGAP9 | 1.062049062 | 0.505208466 | 64 | 77 |
| VCX | 1.109589041 | 0.509105602 | 9 | 10 |
| USP41 | 1.031746032 | 0.517564485 | 40 | 48 |
| WASHC1 | 1.033410138 | 0.522410431 | 26 | 31 |
| IGKV1-16 | 1.033410138 | 0.522410431 | 26 | 31 |
| NPIPB11 | 1.033410138 | 0.522410431 | 26 | 31 |
| PCNX4 | 1.230769231 | 0.525340747 | 4 | 4 |
| PDZD7 | 1.230769231 | 0.525340747 | 4 | 4 |
| THBS2 | 1.230769231 | 0.525340747 | 4 | 4 |
| SNAPC4 | 1.230769231 | 0.525340747 | 4 | 4 |
| SPTBN2 | 1.230769231 | 0.525340747 | 4 | 4 |
| RBMXL3 | 1.230769231 | 0.525340747 | 4 | 4 |
| SPATA13 | 1.230769231 | 0.525340747 | 4 | 4 |
| GOLGA4 | 1.230769231 | 0.525340747 | 4 | 4 |
| PDZD2 | 1.230769231 | 0.525340747 | 4 | 4 |
| PRAM1 | 1.230769231 | 0.525340747 | 4 | 4 |
| EXTL1 | 1.230769231 | 0.525340747 | 4 | 4 |
| ZNF638 | 1.230769231 | 0.525340747 | 4 | 4 |
| ITGB6 | 1.230769231 | 0.525340747 | 4 | 4 |
| SIPA1L3 | 1.230769231 | 0.525340747 | 4 | 4 |
| AGBL3 | 1.230769231 | 0.525340747 | 4 | 4 |
| ZNF646 | 1.230769231 | 0.525340747 | 4 | 4 |
| ARHGEF37 | 1.230769231 | 0.525340747 | 4 | 4 |
| ARHGEF10 | 1.230769231 | 0.525340747 | 4 | 4 |
| GOLGA6D | 1.230769231 | 0.525340747 | 4 | 4 |
| SRBD1 | 1.230769231 | 0.525340747 | 4 | 4 |
| CACNA1D | 1.230769231 | 0.525340747 | 4 | 4 |
| PNPLA7 | 1.230769231 | 0.525340747 | 4 | 4 |
| C2CD4A | 1.230769231 | 0.525340747 | 4 | 4 |
| COL6A2 | 1.230769231 | 0.525340747 | 4 | 4 |
| FANCM | 1.230769231 | 0.525340747 | 4 | 4 |
| COL24A1 | 1.230769231 | 0.525340747 | 4 | 4 |
| KRTAP10-12 | 1.230769231 | 0.525340747 | 4 | 4 |
| ANAPC1 | 1.230769231 | 0.525340747 | 4 | 4 |
| CARMIL2 | 1.230769231 | 0.525340747 | 4 | 4 |
| DNAAF3 | 1.230769231 | 0.525340747 | 4 | 4 |
| ZNF280D | 1.230769231 | 0.525340747 | 4 | 4 |
| ANKRD11 | 1.230769231 | 0.525340747 | 4 | 4 |
| CIC | 1.230769231 | 0.525340747 | 4 | 4 |
| KIF1C | 1.230769231 | 0.525340747 | 4 | 4 |
| PIGR | 1.230769231 | 0.525340747 | 4 | 4 |
| TDRD6 | 1.230769231 | 0.525340747 | 4 | 4 |
| PRSS1 | 1.230769231 | 0.525340747 | 4 | 4 |
| SCN5A | 1.230769231 | 0.525340747 | 4 | 4 |
| LOC441155 | 1.230769231 | 0.525340747 | 4 | 4 |
| ATG2A | 1.230769231 | 0.525340747 | 4 | 4 |
| IRF2BPL | 1.230769231 | 0.525340747 | 4 | 4 |
| NUP214 | 1.230769231 | 0.525340747 | 4 | 4 |
| HS6ST3 | 1.230769231 | 0.525340747 | 4 | 4 |
| ZBTB40 | 1.230769231 | 0.525340747 | 4 | 4 |
| MYBPC3 | 1.230769231 | 0.525340747 | 4 | 4 |
| ARFGEF3 | 1.230769231 | 0.525340747 | 4 | 4 |
| MLLT10 | 1.230769231 | 0.525340747 | 4 | 4 |
| SIGLEC1 | 1.230769231 | 0.525340747 | 4 | 4 |
| NBPF11 | 1.230769231 | 0.525340747 | 4 | 4 |
| HLA-DRB1 | 1.159139785 | 0.525741865 | 77 | 93 |
| GOLGA8H | 1.025641026 | 0.528602416 | 30 | 36 |
| IGHGP | 1.047736626 | 0.5291654 | 67 | 81 |
| HYDIN | 1.093093093 | 0.529576789 | 8 | 9 |
| PRAMEF1 | 1.053061224 | 0.534239696 | 12 | 14 |
| CDON | inf | 0.549450549 | 82 | 99 |
| SPATA31A1 | inf | 0.549450549 | 82 | 99 |
| LOC100133128 | inf | 0.549450549 | 82 | 99 |
| OBSL1 | 1.073333333 | 0.552363721 | 7 | 8 |
| ZFHX3 | 1.073333333 | 0.552363721 | 7 | 8 |
| LOC101060022 | 1.073333333 | 0.552363721 | 7 | 8 |
| LAD1 | 1.036836403 | 0.552571638 | 11 | 13 |
| LILRB4 | 1.003968254 | 0.554646175 | 46 | 56 |
| DYNAP | 1.019900498 | 0.554898216 | 15 | 18 |
| NBPF15 | 1.029411765 | 0.557642726 | 70 | 85 |
| ARHGEF5 | 1.00331675 | 0.559483878 | 55 | 67 |
| CCR5 | 1.227848101 | 0.561026077 | 3 | 3 |
| USP24 | 1.227848101 | 0.561026077 | 3 | 3 |
| SPAG17 | 1.227848101 | 0.561026077 | 3 | 3 |
| SUN1 | 1.227848101 | 0.561026077 | 3 | 3 |
| MYPOP | 1.227848101 | 0.561026077 | 3 | 3 |
| KEL | 1.227848101 | 0.561026077 | 3 | 3 |
| TMEM82 | 1.227848101 | 0.561026077 | 3 | 3 |
| FAM47C | 1.227848101 | 0.561026077 | 3 | 3 |
| TBC1D2 | 1.227848101 | 0.561026077 | 3 | 3 |
| EHD3 | 1.227848101 | 0.561026077 | 3 | 3 |
| IREB2 | 1.227848101 | 0.561026077 | 3 | 3 |
| ADAMTS12 | 1.227848101 | 0.561026077 | 3 | 3 |
| AXIN1 | 1.227848101 | 0.561026077 | 3 | 3 |
| TRIM32 | 1.227848101 | 0.561026077 | 3 | 3 |
| MCOLN2 | 1.227848101 | 0.561026077 | 3 | 3 |
| OR2W3 | 1.227848101 | 0.561026077 | 3 | 3 |
| PLEKHG4B | 1.227848101 | 0.561026077 | 3 | 3 |
| P2RY2 | 1.227848101 | 0.561026077 | 3 | 3 |
| CEP131 | 1.227848101 | 0.561026077 | 3 | 3 |
| PPP1R18 | 1.227848101 | 0.561026077 | 3 | 3 |
| ANXA6 | 1.227848101 | 0.561026077 | 3 | 3 |
| TRPM1 | 1.227848101 | 0.561026077 | 3 | 3 |
| THBS1 | 1.227848101 | 0.561026077 | 3 | 3 |
| SERPINA6 | 1.227848101 | 0.561026077 | 3 | 3 |
| TECPR2 | 1.227848101 | 0.561026077 | 3 | 3 |
| SLC4A11 | 1.227848101 | 0.561026077 | 3 | 3 |
| TACC1 | 1.227848101 | 0.561026077 | 3 | 3 |
| SOGA1 | 1.227848101 | 0.561026077 | 3 | 3 |
| HPS5 | 1.227848101 | 0.561026077 | 3 | 3 |
| AIFM2 | 1.227848101 | 0.561026077 | 3 | 3 |
| PTPN21 | 1.227848101 | 0.561026077 | 3 | 3 |
| TXNRD3 | 1.227848101 | 0.561026077 | 3 | 3 |
| ACOX3 | 1.227848101 | 0.561026077 | 3 | 3 |
| ZNF117 | 1.227848101 | 0.561026077 | 3 | 3 |
| ELOA3D | 1.227848101 | 0.561026077 | 3 | 3 |
| ASPSCR1 | 1.227848101 | 0.561026077 | 3 | 3 |
| LOC101928195 | 1.227848101 | 0.561026077 | 3 | 3 |
| NCAPD2 | 1.227848101 | 0.561026077 | 3 | 3 |
| FER1L5 | 1.227848101 | 0.561026077 | 3 | 3 |
| DUOX1 | 1.227848101 | 0.561026077 | 3 | 3 |
| CECR2 | 1.227848101 | 0.561026077 | 3 | 3 |
| MAML2 | 1.227848101 | 0.561026077 | 3 | 3 |
| KRT6B | 1.227848101 | 0.561026077 | 3 | 3 |
| ASCC3 | 1.227848101 | 0.561026077 | 3 | 3 |
| NWD2 | 1.227848101 | 0.561026077 | 3 | 3 |
| INSRR | 1.227848101 | 0.561026077 | 3 | 3 |
| RGS3 | 1.227848101 | 0.561026077 | 3 | 3 |
| NUMB | 1.227848101 | 0.561026077 | 3 | 3 |
| CP | 1.227848101 | 0.561026077 | 3 | 3 |
| ALOX12B | 1.227848101 | 0.561026077 | 3 | 3 |
| SLC26A11 | 1.227848101 | 0.561026077 | 3 | 3 |
| SPAG1 | 1.227848101 | 0.561026077 | 3 | 3 |
| ATP4A | 1.227848101 | 0.561026077 | 3 | 3 |
| USP32 | 1.227848101 | 0.561026077 | 3 | 3 |
| GRK4 | 1.227848101 | 0.561026077 | 3 | 3 |
| OTOGL | 1.227848101 | 0.561026077 | 3 | 3 |
| LONRF2 | 1.227848101 | 0.561026077 | 3 | 3 |
| DSTYK | 1.227848101 | 0.561026077 | 3 | 3 |
| ERBB3 | 1.227848101 | 0.561026077 | 3 | 3 |
| MAST1 | 1.227848101 | 0.561026077 | 3 | 3 |
| SH2D3A | 1.227848101 | 0.561026077 | 3 | 3 |
| RHOT2 | 1.227848101 | 0.561026077 | 3 | 3 |
| FLII | 1.227848101 | 0.561026077 | 3 | 3 |
| AMY2B | 1.227848101 | 0.561026077 | 3 | 3 |
| MCM9 | 1.227848101 | 0.561026077 | 3 | 3 |
| DIDO1 | 1.227848101 | 0.561026077 | 3 | 3 |
| ERCC6 | 1.227848101 | 0.561026077 | 3 | 3 |
| MMEL1 | 1.227848101 | 0.561026077 | 3 | 3 |
| EEF1D | 1.227848101 | 0.561026077 | 3 | 3 |
| MAN2B2 | 1.227848101 | 0.561026077 | 3 | 3 |
| ZSCAN32 | 1.227848101 | 0.561026077 | 3 | 3 |
| MPP2 | 1.227848101 | 0.561026077 | 3 | 3 |
| YLPM1 | 1.227848101 | 0.561026077 | 3 | 3 |
| PIWIL4 | 1.227848101 | 0.561026077 | 3 | 3 |
| NEU4 | 1.227848101 | 0.561026077 | 3 | 3 |
| LARP1 | 1.227848101 | 0.561026077 | 3 | 3 |
| MESP1 | 1.227848101 | 0.561026077 | 3 | 3 |
| CRMP1 | 1.227848101 | 0.561026077 | 3 | 3 |
| RGPD8 | 1.227848101 | 0.561026077 | 3 | 3 |
| DMGDH | 1.227848101 | 0.561026077 | 3 | 3 |
| METAP2 | 1.227848101 | 0.561026077 | 3 | 3 |
| SEMA5B | 1.227848101 | 0.561026077 | 3 | 3 |
| EME2 | 1.227848101 | 0.561026077 | 3 | 3 |
| LOC101927601 | 1.227848101 | 0.561026077 | 3 | 3 |
| MYO16 | 1.227848101 | 0.561026077 | 3 | 3 |
| FANCE | 1.227848101 | 0.561026077 | 3 | 3 |
| LAMB3 | 1.227848101 | 0.561026077 | 3 | 3 |
| EPHA8 | 1.227848101 | 0.561026077 | 3 | 3 |
| SLC45A4 | 1.227848101 | 0.561026077 | 3 | 3 |
| BICRAL | 1.227848101 | 0.561026077 | 3 | 3 |
| NLRP4 | 1.227848101 | 0.561026077 | 3 | 3 |
| KRBA1 | 1.227848101 | 0.561026077 | 3 | 3 |
| FAM178B | 1.227848101 | 0.561026077 | 3 | 3 |
| MSANTD1 | 1.227848101 | 0.561026077 | 3 | 3 |
| TET3 | 1.227848101 | 0.561026077 | 3 | 3 |
| ACP4 | 1.227848101 | 0.561026077 | 3 | 3 |
| KLHDC7A | 1.227848101 | 0.561026077 | 3 | 3 |
| EHBP1L1 | 1.227848101 | 0.561026077 | 3 | 3 |
| SAMD11 | 1.227848101 | 0.561026077 | 3 | 3 |
| GRID2IP | 1.227848101 | 0.561026077 | 3 | 3 |
| SHC2 | 1.227848101 | 0.561026077 | 3 | 3 |
| OR2A12 | 1.227848101 | 0.561026077 | 3 | 3 |
| CHI3L1 | 1.227848101 | 0.561026077 | 3 | 3 |
| ADAMTSL3 | 1.227848101 | 0.561026077 | 3 | 3 |
| SVIL | 1.227848101 | 0.561026077 | 3 | 3 |
| FRMD8 | 1.227848101 | 0.561026077 | 3 | 3 |
| GALNT14 | 1.227848101 | 0.561026077 | 3 | 3 |
| SCN3A | 1.227848101 | 0.561026077 | 3 | 3 |
| ZNF658 | 1.227848101 | 0.561026077 | 3 | 3 |
| EP400 | 1.227848101 | 0.561026077 | 3 | 3 |
| MROH8 | 1.227848101 | 0.561026077 | 3 | 3 |
| MEGF8 | 1.227848101 | 0.561026077 | 3 | 3 |
| ADNP2 | 1.227848101 | 0.561026077 | 3 | 3 |
| COL15A1 | 1.227848101 | 0.561026077 | 3 | 3 |
| DMXL2 | 1.227848101 | 0.561026077 | 3 | 3 |
| LRRIQ1 | 1.227848101 | 0.561026077 | 3 | 3 |
| PPFIA4 | 1.227848101 | 0.561026077 | 3 | 3 |
| NPHP3 | 1.227848101 | 0.561026077 | 3 | 3 |
| TKFC | 1.227848101 | 0.561026077 | 3 | 3 |
| COL23A1 | 1.227848101 | 0.561026077 | 3 | 3 |
| FBN2 | 1.227848101 | 0.561026077 | 3 | 3 |
| GLI2 | 1.227848101 | 0.561026077 | 3 | 3 |
| GRID2 | 1.227848101 | 0.561026077 | 3 | 3 |
| SPIDR | 1.227848101 | 0.561026077 | 3 | 3 |
| POU2F3 | 1.227848101 | 0.561026077 | 3 | 3 |
| CTTNBP2 | 1.227848101 | 0.561026077 | 3 | 3 |
| PRF1 | 1.227848101 | 0.561026077 | 3 | 3 |
| NLRP1 | 1.227848101 | 0.561026077 | 3 | 3 |
| FAM160B2 | 1.227848101 | 0.561026077 | 3 | 3 |
| USP17L13 | 1.227848101 | 0.561026077 | 3 | 3 |
| ZHX2 | 1.227848101 | 0.561026077 | 3 | 3 |
| SLC22A15 | 1.227848101 | 0.561026077 | 3 | 3 |
| CHAMP1 | 1.227848101 | 0.561026077 | 3 | 3 |
| CENPJ | 1.227848101 | 0.561026077 | 3 | 3 |
| ZNF714 | 1.227848101 | 0.561026077 | 3 | 3 |
| PTPRN2 | 1.227848101 | 0.561026077 | 3 | 3 |
| ZFP64 | 1.227848101 | 0.561026077 | 3 | 3 |
| HKDC1 | 1.227848101 | 0.561026077 | 3 | 3 |
| COBLL1 | 1.227848101 | 0.561026077 | 3 | 3 |
| UBAP1L | 1.227848101 | 0.561026077 | 3 | 3 |
| ZNF316 | 1.227848101 | 0.561026077 | 3 | 3 |
| LCN8 | 1.002421308 | 0.562004244 | 23 | 28 |
| DNAH14 | 1.005190311 | 0.571617982 | 14 | 17 |
| USP17L17 | 1.010989011 | 0.57174532 | 69 | 84 |
| LOC101927530 | 1.018518519 | 0.572250346 | 10 | 12 |
| MYO15A | 1.018518519 | 0.572250346 | 10 | 12 |
| LOC100129307 | 0.997159091 | 0.573106088 | 18 | 22 |
| MST1L | 1.653061224 | 0.574342784 | 81 | 98 |
| NACAD | 0.991358025 | 0.575773462 | 22 | 27 |
| MROH5 | 1.04887218 | 0.578176868 | 6 | 7 |
| DNAH1 | 1.04887218 | 0.578176868 | 6 | 7 |
| NWD1 | 1.04887218 | 0.578176868 | 6 | 7 |
| SIPA1L2 | 1.04887218 | 0.578176868 | 6 | 7 |
| POLE | 1.04887218 | 0.578176868 | 6 | 7 |
| MACF1 | 1.04887218 | 0.578176868 | 6 | 7 |
| TRIOBP | 1.04887218 | 0.578176868 | 6 | 7 |
| LOC642643 | 0.987089202 | 0.581452358 | 58 | 71 |
| ACSM2A | 0.978174603 | 0.588300441 | 34 | 42 |
| PRAMEF5 | 0.975 | 0.594006007 | 52 | 64 |
| DNAH5 | 1.017316017 | 0.608092867 | 5 | 6 |
| SMAP1 | 1.017316017 | 0.608092867 | 5 | 6 |
| ARHGEF35 | 1.017316017 | 0.608092867 | 5 | 6 |
| CELSR2 | 1.017316017 | 0.608092867 | 5 | 6 |
| FNDC1 | 1.017316017 | 0.608092867 | 5 | 6 |
| IGHV4-4 | 1.017316017 | 0.608092867 | 5 | 6 |
| TSPY1 | 1.017316017 | 0.608092867 | 5 | 6 |
| ZNF142 | 1.017316017 | 0.608092867 | 5 | 6 |
| LAMC2 | 1.017316017 | 0.608092867 | 5 | 6 |
| MYH13 | 1.017316017 | 0.608092867 | 5 | 6 |
| PRR12 | 1.017316017 | 0.608092867 | 5 | 6 |
| LOC101927662 | 0.960784314 | 0.611474411 | 41 | 51 |
| CDAN1 | 1.225 | 0.6115146 | 2 | 2 |
| GPR151 | 1.225 | 0.6115146 | 2 | 2 |
| FGL1 | 1.225 | 0.6115146 | 2 | 2 |
| RABEP2 | 1.225 | 0.6115146 | 2 | 2 |
| SLCO3A1 | 1.225 | 0.6115146 | 2 | 2 |
| DDX58 | 1.225 | 0.6115146 | 2 | 2 |
| TMEM221 | 1.225 | 0.6115146 | 2 | 2 |
| KIF15 | 1.225 | 0.6115146 | 2 | 2 |
| SHISA8 | 1.225 | 0.6115146 | 2 | 2 |
| PWWP2B | 1.225 | 0.6115146 | 2 | 2 |
| PPP1R12B | 1.225 | 0.6115146 | 2 | 2 |
| BACH2 | 1.225 | 0.6115146 | 2 | 2 |
| ZNF229 | 1.225 | 0.6115146 | 2 | 2 |
| GTSE1 | 1.225 | 0.6115146 | 2 | 2 |
| MIPOL1 | 1.225 | 0.6115146 | 2 | 2 |
| CCT8L2 | 1.225 | 0.6115146 | 2 | 2 |
| KLHL29 | 1.225 | 0.6115146 | 2 | 2 |
| TMEM214 | 1.225 | 0.6115146 | 2 | 2 |
| IL17RB | 1.225 | 0.6115146 | 2 | 2 |
| CMIP | 1.225 | 0.6115146 | 2 | 2 |
| B4GALNT2 | 1.225 | 0.6115146 | 2 | 2 |
| IL1R2 | 1.225 | 0.6115146 | 2 | 2 |
| GAS2L1 | 1.225 | 0.6115146 | 2 | 2 |
| QRSL1 | 1.225 | 0.6115146 | 2 | 2 |
| XYLT1 | 1.225 | 0.6115146 | 2 | 2 |
| ACAP1 | 1.225 | 0.6115146 | 2 | 2 |
| FBP1 | 1.225 | 0.6115146 | 2 | 2 |
| PHLDB3 | 1.225 | 0.6115146 | 2 | 2 |
| PCCB | 1.225 | 0.6115146 | 2 | 2 |
| GRIN2C | 1.225 | 0.6115146 | 2 | 2 |
| SHD | 1.225 | 0.6115146 | 2 | 2 |
| SBF2 | 1.225 | 0.6115146 | 2 | 2 |
| CD33 | 1.225 | 0.6115146 | 2 | 2 |
| KIRREL3 | 1.225 | 0.6115146 | 2 | 2 |
| GARS | 1.225 | 0.6115146 | 2 | 2 |
| SHCBP1 | 1.225 | 0.6115146 | 2 | 2 |
| TDRD7 | 1.225 | 0.6115146 | 2 | 2 |
| SLC19A1 | 1.225 | 0.6115146 | 2 | 2 |
| MFN1 | 1.225 | 0.6115146 | 2 | 2 |
| SV2C | 1.225 | 0.6115146 | 2 | 2 |
| GAN | 1.225 | 0.6115146 | 2 | 2 |
| CNBD2 | 1.225 | 0.6115146 | 2 | 2 |
| GJB4 | 1.225 | 0.6115146 | 2 | 2 |
| ZNF25 | 1.225 | 0.6115146 | 2 | 2 |
| FBXL17 | 1.225 | 0.6115146 | 2 | 2 |
| CD99L2 | 1.225 | 0.6115146 | 2 | 2 |
| RIMBP2 | 1.225 | 0.6115146 | 2 | 2 |
| MICAL1 | 1.225 | 0.6115146 | 2 | 2 |
| ACTRT2 | 1.225 | 0.6115146 | 2 | 2 |
| FAM118A | 1.225 | 0.6115146 | 2 | 2 |
| HECA | 1.225 | 0.6115146 | 2 | 2 |
| MIA3 | 1.225 | 0.6115146 | 2 | 2 |
| PAPD4 | 1.225 | 0.6115146 | 2 | 2 |
| RAB11FIP3 | 1.225 | 0.6115146 | 2 | 2 |
| TRIM29 | 1.225 | 0.6115146 | 2 | 2 |
| PHYKPL | 1.225 | 0.6115146 | 2 | 2 |
| GAS2L3 | 1.225 | 0.6115146 | 2 | 2 |
| IL12RB1 | 1.225 | 0.6115146 | 2 | 2 |
| CD55 | 1.225 | 0.6115146 | 2 | 2 |
| TAOK2 | 1.225 | 0.6115146 | 2 | 2 |
| TRIM56 | 1.225 | 0.6115146 | 2 | 2 |
| MYOM1 | 1.225 | 0.6115146 | 2 | 2 |
| SPECC1L | 1.225 | 0.6115146 | 2 | 2 |
| LOXL2 | 1.225 | 0.6115146 | 2 | 2 |
| NOTCH2 | 1.225 | 0.6115146 | 2 | 2 |
| GBF1 | 1.225 | 0.6115146 | 2 | 2 |
| ARHGEF12 | 1.225 | 0.6115146 | 2 | 2 |
| ITGB7 | 1.225 | 0.6115146 | 2 | 2 |
| ACSS1 | 1.225 | 0.6115146 | 2 | 2 |
| ASB2 | 1.225 | 0.6115146 | 2 | 2 |
| SCARB2 | 1.225 | 0.6115146 | 2 | 2 |
| PDZD3 | 1.225 | 0.6115146 | 2 | 2 |
| ZMYM6 | 1.225 | 0.6115146 | 2 | 2 |
| RAVER1 | 1.225 | 0.6115146 | 2 | 2 |
| MPST | 1.225 | 0.6115146 | 2 | 2 |
| ITGAV | 1.225 | 0.6115146 | 2 | 2 |
| KCNK4 | 1.225 | 0.6115146 | 2 | 2 |
| HCN4 | 1.225 | 0.6115146 | 2 | 2 |
| ASCC2 | 1.225 | 0.6115146 | 2 | 2 |
| PCDHGA8 | 1.225 | 0.6115146 | 2 | 2 |
| GGA3 | 1.225 | 0.6115146 | 2 | 2 |
| RASGRF2 | 1.225 | 0.6115146 | 2 | 2 |
| ARHGAP31 | 1.225 | 0.6115146 | 2 | 2 |
| RASA4 | 1.225 | 0.6115146 | 2 | 2 |
| MOSPD3 | 1.225 | 0.6115146 | 2 | 2 |
| GGCX | 1.225 | 0.6115146 | 2 | 2 |
| GSC2 | 1.225 | 0.6115146 | 2 | 2 |
| NRROS | 1.225 | 0.6115146 | 2 | 2 |
| PCDHGA3 | 1.225 | 0.6115146 | 2 | 2 |
| KDELC1 | 1.225 | 0.6115146 | 2 | 2 |
| CIITA | 1.225 | 0.6115146 | 2 | 2 |
| ATAD5 | 1.225 | 0.6115146 | 2 | 2 |
| CIT | 1.225 | 0.6115146 | 2 | 2 |
| DCC | 1.225 | 0.6115146 | 2 | 2 |
| CITED4 | 1.225 | 0.6115146 | 2 | 2 |
| FAM227B | 1.225 | 0.6115146 | 2 | 2 |
| MROH9 | 1.225 | 0.6115146 | 2 | 2 |
| TCN2 | 1.225 | 0.6115146 | 2 | 2 |
| DYM | 1.225 | 0.6115146 | 2 | 2 |
| SLC6A9 | 1.225 | 0.6115146 | 2 | 2 |
| RC3H1 | 1.225 | 0.6115146 | 2 | 2 |
| RBM27 | 1.225 | 0.6115146 | 2 | 2 |
| GSR | 1.225 | 0.6115146 | 2 | 2 |
| JMJD1C | 1.225 | 0.6115146 | 2 | 2 |
| TNPO3 | 1.225 | 0.6115146 | 2 | 2 |
| ARID5A | 1.225 | 0.6115146 | 2 | 2 |
| TNR | 1.225 | 0.6115146 | 2 | 2 |
| PDGFD | 1.225 | 0.6115146 | 2 | 2 |
| F13A1 | 1.225 | 0.6115146 | 2 | 2 |
| SEC14L3 | 1.225 | 0.6115146 | 2 | 2 |
| SULT1A1 | 1.225 | 0.6115146 | 2 | 2 |
| CFH | 1.225 | 0.6115146 | 2 | 2 |
| ACSL1 | 1.225 | 0.6115146 | 2 | 2 |
| SIRPA | 1.225 | 0.6115146 | 2 | 2 |
| ARRDC1 | 1.225 | 0.6115146 | 2 | 2 |
| MSS51 | 1.225 | 0.6115146 | 2 | 2 |
| SIX5 | 1.225 | 0.6115146 | 2 | 2 |
| ITSN2 | 1.225 | 0.6115146 | 2 | 2 |
| DUSP27 | 1.225 | 0.6115146 | 2 | 2 |
| ZEB1 | 1.225 | 0.6115146 | 2 | 2 |
| ARHGEF38 | 1.225 | 0.6115146 | 2 | 2 |
| RECQL4 | 1.225 | 0.6115146 | 2 | 2 |
| LOC101929630 | 1.225 | 0.6115146 | 2 | 2 |
| TMPRSS7 | 1.225 | 0.6115146 | 2 | 2 |
| ARHGEF2 | 1.225 | 0.6115146 | 2 | 2 |
| DSPP | 1.225 | 0.6115146 | 2 | 2 |
| SEC24C | 1.225 | 0.6115146 | 2 | 2 |
| MN1 | 1.225 | 0.6115146 | 2 | 2 |
| RAG1 | 1.225 | 0.6115146 | 2 | 2 |
| KIAA1107 | 1.225 | 0.6115146 | 2 | 2 |
| POU6F2 | 1.225 | 0.6115146 | 2 | 2 |
| TMEM43 | 1.225 | 0.6115146 | 2 | 2 |
| FFAR3 | 1.225 | 0.6115146 | 2 | 2 |
| ACSF3 | 1.225 | 0.6115146 | 2 | 2 |
| RAD9A | 1.225 | 0.6115146 | 2 | 2 |
| AFDN | 1.225 | 0.6115146 | 2 | 2 |
| PCDHA3 | 1.225 | 0.6115146 | 2 | 2 |
| KIAA1217 | 1.225 | 0.6115146 | 2 | 2 |
| NUP155 | 1.225 | 0.6115146 | 2 | 2 |
| INTS10 | 1.225 | 0.6115146 | 2 | 2 |
| GPAT2 | 1.225 | 0.6115146 | 2 | 2 |
| ZNF224 | 1.225 | 0.6115146 | 2 | 2 |
| DLL3 | 1.225 | 0.6115146 | 2 | 2 |
| USP40 | 1.225 | 0.6115146 | 2 | 2 |
| RHBDF2 | 1.225 | 0.6115146 | 2 | 2 |
| SCUBE1 | 1.225 | 0.6115146 | 2 | 2 |
| ZNF804A | 1.225 | 0.6115146 | 2 | 2 |
| MYH2 | 1.225 | 0.6115146 | 2 | 2 |
| RIC1 | 1.225 | 0.6115146 | 2 | 2 |
| ZNF225 | 1.225 | 0.6115146 | 2 | 2 |
| CENPU | 1.225 | 0.6115146 | 2 | 2 |
| ETV7 | 1.225 | 0.6115146 | 2 | 2 |
| OAS3 | 1.225 | 0.6115146 | 2 | 2 |
| MKRN3 | 1.225 | 0.6115146 | 2 | 2 |
| TRBV6-7 | 1.225 | 0.6115146 | 2 | 2 |
| OR2AK2 | 1.225 | 0.6115146 | 2 | 2 |
| NUP88 | 1.225 | 0.6115146 | 2 | 2 |
| ABT1 | 1.225 | 0.6115146 | 2 | 2 |
| IPO9 | 1.225 | 0.6115146 | 2 | 2 |
| KDM6A | 1.225 | 0.6115146 | 2 | 2 |
| PCDHB6 | 1.225 | 0.6115146 | 2 | 2 |
| PPM1G | 1.225 | 0.6115146 | 2 | 2 |
| MMS19 | 1.225 | 0.6115146 | 2 | 2 |
| CLASP2 | 1.225 | 0.6115146 | 2 | 2 |
| LOC101929037 | 1.225 | 0.6115146 | 2 | 2 |
| EXOC1 | 1.225 | 0.6115146 | 2 | 2 |
| SLC12A2 | 1.225 | 0.6115146 | 2 | 2 |
| ABHD11 | 1.225 | 0.6115146 | 2 | 2 |
| ZNF202 | 1.225 | 0.6115146 | 2 | 2 |
| EARS2 | 1.225 | 0.6115146 | 2 | 2 |
| ATG4D | 1.225 | 0.6115146 | 2 | 2 |
| PGGHG | 1.225 | 0.6115146 | 2 | 2 |
| CLCNKB | 1.225 | 0.6115146 | 2 | 2 |
| APPL1 | 1.225 | 0.6115146 | 2 | 2 |
| EXD3 | 1.225 | 0.6115146 | 2 | 2 |
| SEC31B | 1.225 | 0.6115146 | 2 | 2 |
| MMP11 | 1.225 | 0.6115146 | 2 | 2 |
| TCTE1 | 1.225 | 0.6115146 | 2 | 2 |
| SLC12A7 | 1.225 | 0.6115146 | 2 | 2 |
| MX1 | 1.225 | 0.6115146 | 2 | 2 |
| PFKP | 1.225 | 0.6115146 | 2 | 2 |
| TARSL2 | 1.225 | 0.6115146 | 2 | 2 |
| SPATS2L | 1.225 | 0.6115146 | 2 | 2 |
| KIAA0753 | 1.225 | 0.6115146 | 2 | 2 |
| SLC13A2 | 1.225 | 0.6115146 | 2 | 2 |
| IPP | 1.225 | 0.6115146 | 2 | 2 |
| ECE1 | 1.225 | 0.6115146 | 2 | 2 |
| ECEL1 | 1.225 | 0.6115146 | 2 | 2 |
| ABCA12 | 1.225 | 0.6115146 | 2 | 2 |
| MERTK | 1.225 | 0.6115146 | 2 | 2 |
| GNL3 | 1.225 | 0.6115146 | 2 | 2 |
| TENM2 | 1.225 | 0.6115146 | 2 | 2 |
| ADAMTS1 | 1.225 | 0.6115146 | 2 | 2 |
| CAPRIN1 | 1.225 | 0.6115146 | 2 | 2 |
| OR4X1 | 1.225 | 0.6115146 | 2 | 2 |
| PLOD1 | 1.225 | 0.6115146 | 2 | 2 |
| LOC101927462 | 1.225 | 0.6115146 | 2 | 2 |
| C1orf116 | 1.225 | 0.6115146 | 2 | 2 |
| RPUSD1 | 1.225 | 0.6115146 | 2 | 2 |
| C10orf88 | 1.225 | 0.6115146 | 2 | 2 |
| CAPG | 1.225 | 0.6115146 | 2 | 2 |
| PLXNA1 | 1.225 | 0.6115146 | 2 | 2 |
| FSHR | 1.225 | 0.6115146 | 2 | 2 |
| RPS6KB2 | 1.225 | 0.6115146 | 2 | 2 |
| SESN1 | 1.225 | 0.6115146 | 2 | 2 |
| DOCK1 | 1.225 | 0.6115146 | 2 | 2 |
| FAM86B2 | 1.225 | 0.6115146 | 2 | 2 |
| TXNDC12 | 1.225 | 0.6115146 | 2 | 2 |
| AK2 | 1.225 | 0.6115146 | 2 | 2 |
| ENOSF1 | 1.225 | 0.6115146 | 2 | 2 |
| EPHX2 | 1.225 | 0.6115146 | 2 | 2 |
| DDX1 | 1.225 | 0.6115146 | 2 | 2 |
| OR5K4 | 1.225 | 0.6115146 | 2 | 2 |
| HPX | 1.225 | 0.6115146 | 2 | 2 |
| TYR | 1.225 | 0.6115146 | 2 | 2 |
| EIF4G3 | 1.225 | 0.6115146 | 2 | 2 |
| NKPD1 | 1.225 | 0.6115146 | 2 | 2 |
| DNTT | 1.225 | 0.6115146 | 2 | 2 |
| TXLNB | 1.225 | 0.6115146 | 2 | 2 |
| SENP2 | 1.225 | 0.6115146 | 2 | 2 |
| PROM1 | 1.225 | 0.6115146 | 2 | 2 |
| EPRS | 1.225 | 0.6115146 | 2 | 2 |
| SGSM1 | 1.225 | 0.6115146 | 2 | 2 |
| FAM181B | 1.225 | 0.6115146 | 2 | 2 |
| CRYBB3 | 1.225 | 0.6115146 | 2 | 2 |
| TRIP10 | 1.225 | 0.6115146 | 2 | 2 |
| CFAP52 | 1.225 | 0.6115146 | 2 | 2 |
| TIMELESS | 1.225 | 0.6115146 | 2 | 2 |
| BTBD16 | 1.225 | 0.6115146 | 2 | 2 |
| PLEKHG3 | 1.225 | 0.6115146 | 2 | 2 |
| CATSPER2 | 1.225 | 0.6115146 | 2 | 2 |
| CRYZ | 1.225 | 0.6115146 | 2 | 2 |
| CASZ1 | 1.225 | 0.6115146 | 2 | 2 |
| CSF1R | 1.225 | 0.6115146 | 2 | 2 |
| TTLL11 | 1.225 | 0.6115146 | 2 | 2 |
| WDFY1 | 1.225 | 0.6115146 | 2 | 2 |
| CSH1 | 1.225 | 0.6115146 | 2 | 2 |
| MAGI3 | 1.225 | 0.6115146 | 2 | 2 |
| TTLL4 | 1.225 | 0.6115146 | 2 | 2 |
| ZNF541 | 1.225 | 0.6115146 | 2 | 2 |
| CASD1 | 1.225 | 0.6115146 | 2 | 2 |
| SOS2 | 1.225 | 0.6115146 | 2 | 2 |
| TICRR | 1.225 | 0.6115146 | 2 | 2 |
| HHATL | 1.225 | 0.6115146 | 2 | 2 |
| C5orf38 | 1.225 | 0.6115146 | 2 | 2 |
| ZNF554 | 1.225 | 0.6115146 | 2 | 2 |
| CSPP1 | 1.225 | 0.6115146 | 2 | 2 |
| OLFML2B | 1.225 | 0.6115146 | 2 | 2 |
| SEMG1 | 1.225 | 0.6115146 | 2 | 2 |
| NFRKB | 1.225 | 0.6115146 | 2 | 2 |
| HSF1 | 1.225 | 0.6115146 | 2 | 2 |
| PMM1 | 1.225 | 0.6115146 | 2 | 2 |
| UTRN | 1.225 | 0.6115146 | 2 | 2 |
| UTS2R | 1.225 | 0.6115146 | 2 | 2 |
| OPA1 | 1.225 | 0.6115146 | 2 | 2 |
| UBR1 | 1.225 | 0.6115146 | 2 | 2 |
| SLC2A14 | 1.225 | 0.6115146 | 2 | 2 |
| LRRC8A | 1.225 | 0.6115146 | 2 | 2 |
| NPAS3 | 1.225 | 0.6115146 | 2 | 2 |
| NPAS2 | 1.225 | 0.6115146 | 2 | 2 |
| DIXDC1 | 1.225 | 0.6115146 | 2 | 2 |
| LRRC63 | 1.225 | 0.6115146 | 2 | 2 |
| ADCY4 | 1.225 | 0.6115146 | 2 | 2 |
| MIGA2 | 1.225 | 0.6115146 | 2 | 2 |
| UGGT1 | 1.225 | 0.6115146 | 2 | 2 |
| ZNF696 | 1.225 | 0.6115146 | 2 | 2 |
| TFDP2 | 1.225 | 0.6115146 | 2 | 2 |
| RXFP4 | 1.225 | 0.6115146 | 2 | 2 |
| PRAMEF20 | 1.225 | 0.6115146 | 2 | 2 |
| NTN4 | 1.225 | 0.6115146 | 2 | 2 |
| DHTKD1 | 1.225 | 0.6115146 | 2 | 2 |
| VANGL1 | 1.225 | 0.6115146 | 2 | 2 |
| ADCY6 | 1.225 | 0.6115146 | 2 | 2 |
| id368803 | 1.225 | 0.6115146 | 2 | 2 |
| TAB1 | 1.225 | 0.6115146 | 2 | 2 |
| POLR1B | 1.225 | 0.6115146 | 2 | 2 |
| TEX14 | 1.225 | 0.6115146 | 2 | 2 |
| ENTPD3 | 1.225 | 0.6115146 | 2 | 2 |
| FAM71A | 1.225 | 0.6115146 | 2 | 2 |
| ELFN1 | 1.225 | 0.6115146 | 2 | 2 |
| POLL | 1.225 | 0.6115146 | 2 | 2 |
| C8orf82 | 1.225 | 0.6115146 | 2 | 2 |
| UHRF2 | 1.225 | 0.6115146 | 2 | 2 |
| DNAJC16 | 1.225 | 0.6115146 | 2 | 2 |
| UNC5B | 1.225 | 0.6115146 | 2 | 2 |
| SGSM2 | 1.225 | 0.6115146 | 2 | 2 |
| TEDC1 | 1.225 | 0.6115146 | 2 | 2 |
| PRDM1 | 1.225 | 0.6115146 | 2 | 2 |
| TAF6 | 1.225 | 0.6115146 | 2 | 2 |
| FNIP2 | 1.225 | 0.6115146 | 2 | 2 |
| TAX1BP1 | 1.225 | 0.6115146 | 2 | 2 |
| LTN1 | 1.225 | 0.6115146 | 2 | 2 |
| CYP2S1 | 1.225 | 0.6115146 | 2 | 2 |
| LOC101059957 | 1.225 | 0.6115146 | 2 | 2 |
| TGS1 | 1.225 | 0.6115146 | 2 | 2 |
| LTBP3 | 1.225 | 0.6115146 | 2 | 2 |
| CUX1 | 1.225 | 0.6115146 | 2 | 2 |
| CWC22 | 1.225 | 0.6115146 | 2 | 2 |
| C6orf136 | 1.225 | 0.6115146 | 2 | 2 |
| CYP2C8 | 1.225 | 0.6115146 | 2 | 2 |
| NLRP6 | 1.225 | 0.6115146 | 2 | 2 |
| NLRP7 | 1.225 | 0.6115146 | 2 | 2 |
| SLC34A3 | 1.225 | 0.6115146 | 2 | 2 |
| NLRP9 | 1.225 | 0.6115146 | 2 | 2 |
| POMT2 | 1.225 | 0.6115146 | 2 | 2 |
| SLC34A1 | 1.225 | 0.6115146 | 2 | 2 |
| UNC5C | 1.225 | 0.6115146 | 2 | 2 |
| SELENOP | 1.225 | 0.6115146 | 2 | 2 |
| ZNF662 | 1.225 | 0.6115146 | 2 | 2 |
| HNF4A | 1.225 | 0.6115146 | 2 | 2 |
| FAM167A | 1.225 | 0.6115146 | 2 | 2 |
| HNF1A | 1.225 | 0.6115146 | 2 | 2 |
| UBN1 | 1.225 | 0.6115146 | 2 | 2 |
| NOB1 | 1.225 | 0.6115146 | 2 | 2 |
| HMX2 | 1.225 | 0.6115146 | 2 | 2 |
| HMX1 | 1.225 | 0.6115146 | 2 | 2 |
| LIPE | 1.225 | 0.6115146 | 2 | 2 |
| SALL4 | 1.225 | 0.6115146 | 2 | 2 |
| PRRG2 | 1.225 | 0.6115146 | 2 | 2 |
| PRR5-ARHGAP8 | 1.225 | 0.6115146 | 2 | 2 |
| ZZZ3 | 1.225 | 0.6115146 | 2 | 2 |
| ANKLE2 | 1.225 | 0.6115146 | 2 | 2 |
| GLMN | 1.225 | 0.6115146 | 2 | 2 |
| UROC1 | 1.225 | 0.6115146 | 2 | 2 |
| KRT3 | 1.225 | 0.6115146 | 2 | 2 |
| ZNF786 | 1.225 | 0.6115146 | 2 | 2 |
| NCF2 | 1.225 | 0.6115146 | 2 | 2 |
| P4HA3 | 1.225 | 0.6115146 | 2 | 2 |
| PIP5K1A | 1.225 | 0.6115146 | 2 | 2 |
| MDFIC | 1.225 | 0.6115146 | 2 | 2 |
| DIEXF | 1.225 | 0.6115146 | 2 | 2 |
| MCPH1 | 1.225 | 0.6115146 | 2 | 2 |
| IGHG1 | 1.225 | 0.6115146 | 2 | 2 |
| KRT5 | 1.225 | 0.6115146 | 2 | 2 |
| IGHE | 1.225 | 0.6115146 | 2 | 2 |
| SCNN1A | 1.225 | 0.6115146 | 2 | 2 |
| IGHA1 | 1.225 | 0.6115146 | 2 | 2 |
| GLRA4 | 1.225 | 0.6115146 | 2 | 2 |
| SYF2 | 1.225 | 0.6115146 | 2 | 2 |
| COQ2 | 1.225 | 0.6115146 | 2 | 2 |
| NCOA5 | 1.225 | 0.6115146 | 2 | 2 |
| PTGIS | 1.225 | 0.6115146 | 2 | 2 |
| SEMA3F | 1.225 | 0.6115146 | 2 | 2 |
| BEAN1 | 1.225 | 0.6115146 | 2 | 2 |
| BEND7 | 1.225 | 0.6115146 | 2 | 2 |
| TMC6 | 1.225 | 0.6115146 | 2 | 2 |
| TEKT3 | 1.225 | 0.6115146 | 2 | 2 |
| ADGRL4 | 1.225 | 0.6115146 | 2 | 2 |
| ZNF517 | 1.225 | 0.6115146 | 2 | 2 |
| WNK4 | 1.225 | 0.6115146 | 2 | 2 |
| COL9A2 | 1.225 | 0.6115146 | 2 | 2 |
| SLC22A17 | 1.225 | 0.6115146 | 2 | 2 |
| NCAM2 | 1.225 | 0.6115146 | 2 | 2 |
| SH3BP5L | 1.225 | 0.6115146 | 2 | 2 |
| PUM1 | 1.225 | 0.6115146 | 2 | 2 |
| STK10 | 1.225 | 0.6115146 | 2 | 2 |
| MEI1 | 1.225 | 0.6115146 | 2 | 2 |
| OR4E2 | 1.225 | 0.6115146 | 2 | 2 |
| ICE2 | 1.225 | 0.6115146 | 2 | 2 |
| RNF128 | 1.225 | 0.6115146 | 2 | 2 |
| XKR9 | 1.225 | 0.6115146 | 2 | 2 |
| SH3PXD2B | 1.225 | 0.6115146 | 2 | 2 |
| COL11A2 | 1.225 | 0.6115146 | 2 | 2 |
| HELLS | 1.225 | 0.6115146 | 2 | 2 |
| OR13C2 | 1.225 | 0.6115146 | 2 | 2 |
| SASH1 | 1.225 | 0.6115146 | 2 | 2 |
| CCNQ | 1.225 | 0.6115146 | 2 | 2 |
| ZNF839 | 1.225 | 0.6115146 | 2 | 2 |
| RIPOR1 | 1.225 | 0.6115146 | 2 | 2 |
| ACD | 1.225 | 0.6115146 | 2 | 2 |
| RNF167 | 1.225 | 0.6115146 | 2 | 2 |
| PTPRN | 1.225 | 0.6115146 | 2 | 2 |
| SLC22A1 | 1.225 | 0.6115146 | 2 | 2 |
| PADI2 | 1.225 | 0.6115146 | 2 | 2 |
| COL19A1 | 1.225 | 0.6115146 | 2 | 2 |
| DAGLB | 1.225 | 0.6115146 | 2 | 2 |
| PIK3R6 | 1.225 | 0.6115146 | 2 | 2 |
| ANKRD17 | 1.225 | 0.6115146 | 2 | 2 |
| FAM71B | 1.225 | 0.6115146 | 2 | 2 |
| PTPRD | 1.225 | 0.6115146 | 2 | 2 |
| DNAJC10 | 1.225 | 0.6115146 | 2 | 2 |
| PABPC4L | 1.225 | 0.6115146 | 2 | 2 |
| CCDC97 | 1.225 | 0.6115146 | 2 | 2 |
| HEPHL1 | 1.225 | 0.6115146 | 2 | 2 |
| SARS2 | 1.225 | 0.6115146 | 2 | 2 |
| SLC36A4 | 1.225 | 0.6115146 | 2 | 2 |
| PTCH1 | 1.225 | 0.6115146 | 2 | 2 |
| DHDH | 1.225 | 0.6115146 | 2 | 2 |
| CCDC136 | 1.225 | 0.6115146 | 2 | 2 |
| PRX | 1.225 | 0.6115146 | 2 | 2 |
| KSR2 | 1.225 | 0.6115146 | 2 | 2 |
| FYB2 | 1.225 | 0.6115146 | 2 | 2 |
| WDR49 | 1.225 | 0.6115146 | 2 | 2 |
| SERPINA9 | 1.225 | 0.6115146 | 2 | 2 |
| C5 | 1.225 | 0.6115146 | 2 | 2 |
| WDR4 | 1.225 | 0.6115146 | 2 | 2 |
| NEIL1 | 1.225 | 0.6115146 | 2 | 2 |
| WDR35 | 1.225 | 0.6115146 | 2 | 2 |
| FLT4 | 1.225 | 0.6115146 | 2 | 2 |
| MAP3K11 | 1.225 | 0.6115146 | 2 | 2 |
| ICK | 1.225 | 0.6115146 | 2 | 2 |
| OTUD3 | 1.225 | 0.6115146 | 2 | 2 |
| NEK11 | 1.225 | 0.6115146 | 2 | 2 |
| FOXRED2 | 1.225 | 0.6115146 | 2 | 2 |
| IBTK | 1.225 | 0.6115146 | 2 | 2 |
| NEK5 | 1.225 | 0.6115146 | 2 | 2 |
| OR2G6 | 1.225 | 0.6115146 | 2 | 2 |
| NELFA | 1.225 | 0.6115146 | 2 | 2 |
| ORC4 | 1.225 | 0.6115146 | 2 | 2 |
| TELO2 | 1.225 | 0.6115146 | 2 | 2 |
| WDR31 | 1.225 | 0.6115146 | 2 | 2 |
| CRISPLD1 | 1.225 | 0.6115146 | 2 | 2 |
| TIRAP | 1.225 | 0.6115146 | 2 | 2 |
| TINAG | 1.225 | 0.6115146 | 2 | 2 |
| NPSR1 | 1.225 | 0.6115146 | 2 | 2 |
| ZNF516 | 1.225 | 0.6115146 | 2 | 2 |
| SYNPO2L | 1.225 | 0.6115146 | 2 | 2 |
| STARD3 | 1.225 | 0.6115146 | 2 | 2 |
| WDR60 | 1.225 | 0.6115146 | 2 | 2 |
| FAP | 1.225 | 0.6115146 | 2 | 2 |
| CPA1 | 1.225 | 0.6115146 | 2 | 2 |
| TNIP1 | 1.225 | 0.6115146 | 2 | 2 |
| IFRD1 | 1.225 | 0.6115146 | 2 | 2 |
| NSD2 | 1.225 | 0.6115146 | 2 | 2 |
| ZNF433 | 1.225 | 0.6115146 | 2 | 2 |
| ERCC2 | 1.225 | 0.6115146 | 2 | 2 |
| TM7SF2 | 1.225 | 0.6115146 | 2 | 2 |
| MAS1 | 1.225 | 0.6115146 | 2 | 2 |
| KRTAP4-7 | 1.225 | 0.6115146 | 2 | 2 |
| PLA2G7 | 1.225 | 0.6115146 | 2 | 2 |
| PLA2G10 | 1.225 | 0.6115146 | 2 | 2 |
| NOP53 | 1.225 | 0.6115146 | 2 | 2 |
| GLYR1 | 1.225 | 0.6115146 | 2 | 2 |
| ZNF468 | 1.225 | 0.6115146 | 2 | 2 |
| CFAP58 | 1.225 | 0.6115146 | 2 | 2 |
| MAPT | 1.225 | 0.6115146 | 2 | 2 |
| NDST3 | 1.225 | 0.6115146 | 2 | 2 |
| TSSK2 | 1.225 | 0.6115146 | 2 | 2 |
| IGHV3-23 | 0.972972973 | 0.616778728 | 8 | 10 |
| TNFAIP6 | 0.972972973 | 0.616778728 | 8 | 10 |
| LAMA5 | 0.972972973 | 0.616778728 | 8 | 10 |
| AHNAK2 | 0.954438335 | 0.621264229 | 15 | 19 |
| TPTE | 0.952380952 | 0.622678877 | 40 | 50 |
| PKD1 | 0.951710262 | 0.627416236 | 11 | 14 |
| LOC101927985 | 0.947775629 | 0.628512796 | 35 | 44 |
| ATG3 | 0.982978723 | 0.635372443 | 77 | 94 |
| ATXN2 | 0.943703704 | 0.642426357 | 7 | 9 |
| LPA | 0.943703704 | 0.642426357 | 7 | 9 |
| COL7A1 | 0.943703704 | 0.642426357 | 7 | 9 |
| NRG1 | 0.943703704 | 0.642426357 | 7 | 9 |
| STAB2 | 0.943703704 | 0.642426357 | 7 | 9 |
| GOLGA6C | 0.943703704 | 0.642426357 | 7 | 9 |
| POTEE | 0.974358974 | 0.643846513 | 4 | 5 |
| KCP | 0.974358974 | 0.643846513 | 4 | 5 |
| GRIP2 | 0.974358974 | 0.643846513 | 4 | 5 |
| MAML3 | 0.974358974 | 0.643846513 | 4 | 5 |
| WDR87 | 0.974358974 | 0.643846513 | 4 | 5 |
| CENPF | 0.974358974 | 0.643846513 | 4 | 5 |
| BIRC6 | 0.974358974 | 0.643846513 | 4 | 5 |
| FLNB | 0.974358974 | 0.643846513 | 4 | 5 |
| MMP25 | 0.974358974 | 0.643846513 | 4 | 5 |
| MAGI1 | 0.974358974 | 0.643846513 | 4 | 5 |
| SLC2A8 | 0.974358974 | 0.643846513 | 4 | 5 |
| C2CD3 | 0.974358974 | 0.643846513 | 4 | 5 |
| C2orf16 | 0.974358974 | 0.643846513 | 4 | 5 |
| ARHGAP33 | 0.974358974 | 0.643846513 | 4 | 5 |
| ALPK3 | 0.974358974 | 0.643846513 | 4 | 5 |
| TMEM2 | 0.974358974 | 0.643846513 | 4 | 5 |
| TANC2 | 0.974358974 | 0.643846513 | 4 | 5 |
| DMXL1 | 0.974358974 | 0.643846513 | 4 | 5 |
| SYNM | 0.974358974 | 0.643846513 | 4 | 5 |
| TECTA | 0.974358974 | 0.643846513 | 4 | 5 |
| CSHL1 | 0.974358974 | 0.643846513 | 4 | 5 |
| NCKAP5 | 0.974358974 | 0.643846513 | 4 | 5 |
| NOBOX | 0.974358974 | 0.643846513 | 4 | 5 |
| MYO9B | 0.974358974 | 0.643846513 | 4 | 5 |
| SH3TC1 | 0.974358974 | 0.643846513 | 4 | 5 |
| POTEC | 0.935222672 | 0.643870672 | 63 | 78 |
| LOC101927767 | 0.929487179 | 0.648379654 | 10 | 13 |
| RGPD4 | 0.929487179 | 0.648379654 | 10 | 13 |
| FOXD4L3 | 0.931267606 | 0.649247682 | 57 | 71 |
| CLEC18B | 0.919863598 | 0.656762536 | 13 | 17 |
| PCNT | 0.904109589 | 0.670848702 | 9 | 12 |
| DNAH3 | 0.904109589 | 0.670848702 | 9 | 12 |
| HIVEP3 | 0.907894737 | 0.671104196 | 6 | 8 |
| ANO7 | 0.907894737 | 0.671104196 | 6 | 8 |
| CNTRL | 0.907894737 | 0.671104196 | 6 | 8 |
| PRAMEF11 | 0.907894737 | 0.671104196 | 6 | 8 |
| ATP5A1 | 0.907894737 | 0.671104196 | 6 | 8 |
| HLA-DQA1 | 0.90960452 | 0.67367675 | 23 | 30 |
| USP6 | 0.909090909 | 0.674561992 | 60 | 75 |
| PRAMEF4 | 0.904761905 | 0.676505308 | 19 | 25 |
| HERC2 | 0.895522388 | 0.683011525 | 15 | 20 |
| PTPRJ | 0.911392405 | 0.688473685 | 3 | 4 |
| CDHR4 | 0.911392405 | 0.688473685 | 3 | 4 |
| PRODH | 0.911392405 | 0.688473685 | 3 | 4 |
| ADAMTS5 | 0.911392405 | 0.688473685 | 3 | 4 |
| AFF1 | 0.911392405 | 0.688473685 | 3 | 4 |
| AFF3 | 0.911392405 | 0.688473685 | 3 | 4 |
| IQCE | 0.911392405 | 0.688473685 | 3 | 4 |
| GPR158 | 0.911392405 | 0.688473685 | 3 | 4 |
| OGDHL | 0.911392405 | 0.688473685 | 3 | 4 |
| NTN3 | 0.911392405 | 0.688473685 | 3 | 4 |
| NOA1 | 0.911392405 | 0.688473685 | 3 | 4 |
| FOXQ1 | 0.911392405 | 0.688473685 | 3 | 4 |
| CYP11B1 | 0.911392405 | 0.688473685 | 3 | 4 |
| NCOA7 | 0.911392405 | 0.688473685 | 3 | 4 |
| GUCY2C | 0.911392405 | 0.688473685 | 3 | 4 |
| GALNT11 | 0.911392405 | 0.688473685 | 3 | 4 |
| TNS2 | 0.911392405 | 0.688473685 | 3 | 4 |
| NES | 0.911392405 | 0.688473685 | 3 | 4 |
| PELP1 | 0.911392405 | 0.688473685 | 3 | 4 |
| PCDHGA6 | 0.911392405 | 0.688473685 | 3 | 4 |
| NOD1 | 0.911392405 | 0.688473685 | 3 | 4 |
| SZT2 | 0.911392405 | 0.688473685 | 3 | 4 |
| PRR14L | 0.911392405 | 0.688473685 | 3 | 4 |
| PIKFYVE | 0.911392405 | 0.688473685 | 3 | 4 |
| TBCD | 0.911392405 | 0.688473685 | 3 | 4 |
| CNTN4 | 0.911392405 | 0.688473685 | 3 | 4 |
| VIPR2 | 0.911392405 | 0.688473685 | 3 | 4 |
| IRAK2 | 0.911392405 | 0.688473685 | 3 | 4 |
| SLC9A3R2 | 0.911392405 | 0.688473685 | 3 | 4 |
| LRRC41 | 0.911392405 | 0.688473685 | 3 | 4 |
| CLTCL1 | 0.911392405 | 0.688473685 | 3 | 4 |
| ADAMTSL1 | 0.911392405 | 0.688473685 | 3 | 4 |
| XPC | 0.911392405 | 0.688473685 | 3 | 4 |
| ELMSAN1 | 0.911392405 | 0.688473685 | 3 | 4 |
| MYCBP2 | 0.911392405 | 0.688473685 | 3 | 4 |
| LRRC70 | 0.911392405 | 0.688473685 | 3 | 4 |
| ABL1 | 0.911392405 | 0.688473685 | 3 | 4 |
| LRIG1 | 0.911392405 | 0.688473685 | 3 | 4 |
| CNTLN | 0.911392405 | 0.688473685 | 3 | 4 |
| EPN3 | 0.911392405 | 0.688473685 | 3 | 4 |
| ZSCAN5B | 0.911392405 | 0.688473685 | 3 | 4 |
| ZNF197 | 0.911392405 | 0.688473685 | 3 | 4 |
| KIAA1683 | 0.911392405 | 0.688473685 | 3 | 4 |
| COL3A1 | 0.911392405 | 0.688473685 | 3 | 4 |
| RERE | 0.911392405 | 0.688473685 | 3 | 4 |
| CAPN10 | 0.911392405 | 0.688473685 | 3 | 4 |
| IGDCC4 | 0.911392405 | 0.688473685 | 3 | 4 |
| HEATR5B | 0.911392405 | 0.688473685 | 3 | 4 |
| CHD9 | 0.911392405 | 0.688473685 | 3 | 4 |
| NRXN1 | 0.911392405 | 0.688473685 | 3 | 4 |
| CAND2 | 0.911392405 | 0.688473685 | 3 | 4 |
| RBM12B | 0.911392405 | 0.688473685 | 3 | 4 |
| MAST2 | 0.911392405 | 0.688473685 | 3 | 4 |
| R3HCC1L | 0.911392405 | 0.688473685 | 3 | 4 |
| GOLGA6L2 | 0.911392405 | 0.688473685 | 3 | 4 |
| PP2D1 | 0.911392405 | 0.688473685 | 3 | 4 |
| GLB1L2 | 0.911392405 | 0.688473685 | 3 | 4 |
| ZFAT | 0.911392405 | 0.688473685 | 3 | 4 |
| LILRA2 | 0.911392405 | 0.688473685 | 3 | 4 |
| FREM2 | 0.911392405 | 0.688473685 | 3 | 4 |
| PLXNB1 | 0.911392405 | 0.688473685 | 3 | 4 |
| CCDC27 | 0.911392405 | 0.688473685 | 3 | 4 |
| CCDC105 | 0.911392405 | 0.688473685 | 3 | 4 |
| RTN1 | 0.911392405 | 0.688473685 | 3 | 4 |
| CFAP65 | 0.911392405 | 0.688473685 | 3 | 4 |
| LILRA6 | 0.911392405 | 0.688473685 | 3 | 4 |
| SAMD9L | 0.911392405 | 0.688473685 | 3 | 4 |
| URB1 | 0.911392405 | 0.688473685 | 3 | 4 |
| MCF2L | 0.911392405 | 0.688473685 | 3 | 4 |
| ALK | 0.911392405 | 0.688473685 | 3 | 4 |
| SLX4 | 0.911392405 | 0.688473685 | 3 | 4 |
| NUAK2 | 0.911392405 | 0.688473685 | 3 | 4 |
| CAPN12 | 0.911392405 | 0.688473685 | 3 | 4 |
| KIR3DL2 | 0.911392405 | 0.688473685 | 3 | 4 |
| CEP128 | 0.911392405 | 0.688473685 | 3 | 4 |
| KCNK15 | 0.911392405 | 0.688473685 | 3 | 4 |
| TSC2 | 0.911392405 | 0.688473685 | 3 | 4 |
| IKBKE | 0.911392405 | 0.688473685 | 3 | 4 |
| ZNF609 | 0.911392405 | 0.688473685 | 3 | 4 |
| ARHGAP23 | 0.890625 | 0.691574057 | 18 | 24 |
| FAM230A | 0.874692875 | 0.695086455 | 8 | 11 |
| CD34 | 0.880165289 | 0.696026817 | 71 | 88 |
| OR6M1 | 1.222222222 | 0.699471799 | 1 | 1 |
| AHSG | 1.222222222 | 0.699471799 | 1 | 1 |
| EPM2A | 1.222222222 | 0.699471799 | 1 | 1 |
| OR5L2 | 1.222222222 | 0.699471799 | 1 | 1 |
| WASF3 | 1.222222222 | 0.699471799 | 1 | 1 |
| OR5K3 | 1.222222222 | 0.699471799 | 1 | 1 |
| C11orf49 | 1.222222222 | 0.699471799 | 1 | 1 |
| PLEKHM3 | 1.222222222 | 0.699471799 | 1 | 1 |
| NIT2 | 1.222222222 | 0.699471799 | 1 | 1 |
| LYPD3 | 1.222222222 | 0.699471799 | 1 | 1 |
| LYSMD4 | 1.222222222 | 0.699471799 | 1 | 1 |
| AARS | 1.222222222 | 0.699471799 | 1 | 1 |
| AIMP2 | 1.222222222 | 0.699471799 | 1 | 1 |
| LBHD1 | 1.222222222 | 0.699471799 | 1 | 1 |
| AICDA | 1.222222222 | 0.699471799 | 1 | 1 |
| AKR7A3 | 1.222222222 | 0.699471799 | 1 | 1 |
| PM20D2 | 1.222222222 | 0.699471799 | 1 | 1 |
| FMO3 | 1.222222222 | 0.699471799 | 1 | 1 |
| AKAP8L | 1.222222222 | 0.699471799 | 1 | 1 |
| SOHLH2 | 1.222222222 | 0.699471799 | 1 | 1 |
| OR10K1 | 1.222222222 | 0.699471799 | 1 | 1 |
| AKAP5 | 1.222222222 | 0.699471799 | 1 | 1 |
| MAD2L2 | 1.222222222 | 0.699471799 | 1 | 1 |
| VWA3B | 1.222222222 | 0.699471799 | 1 | 1 |
| AKIP1 | 1.222222222 | 0.699471799 | 1 | 1 |
| SPRYD4 | 1.222222222 | 0.699471799 | 1 | 1 |
| C11orf1 | 1.222222222 | 0.699471799 | 1 | 1 |
| PLXDC1 | 1.222222222 | 0.699471799 | 1 | 1 |
| NHSL2 | 1.222222222 | 0.699471799 | 1 | 1 |
| KIZ | 1.222222222 | 0.699471799 | 1 | 1 |
| ALKAL1 | 1.222222222 | 0.699471799 | 1 | 1 |
| FAM151A | 1.222222222 | 0.699471799 | 1 | 1 |
| ZNF589 | 1.222222222 | 0.699471799 | 1 | 1 |
| NINJ1 | 1.222222222 | 0.699471799 | 1 | 1 |
| NGLY1 | 1.222222222 | 0.699471799 | 1 | 1 |
| VTN | 1.222222222 | 0.699471799 | 1 | 1 |
| ZNF564 | 1.222222222 | 0.699471799 | 1 | 1 |
| OMG | 1.222222222 | 0.699471799 | 1 | 1 |
| ZNF576 | 1.222222222 | 0.699471799 | 1 | 1 |
| OR6K3 | 1.222222222 | 0.699471799 | 1 | 1 |
| ZNF560 | 1.222222222 | 0.699471799 | 1 | 1 |
| NFX1 | 1.222222222 | 0.699471799 | 1 | 1 |
| FMOD | 1.222222222 | 0.699471799 | 1 | 1 |
| FAM98A | 1.222222222 | 0.699471799 | 1 | 1 |
| MAGEA8 | 1.222222222 | 0.699471799 | 1 | 1 |
| OMD | 1.222222222 | 0.699471799 | 1 | 1 |
| C11orf24 | 1.222222222 | 0.699471799 | 1 | 1 |
| SMARCA1 | 1.222222222 | 0.699471799 | 1 | 1 |
| OR10A7 | 1.222222222 | 0.699471799 | 1 | 1 |
| PLEKHM1 | 1.222222222 | 0.699471799 | 1 | 1 |
| MAP6 | 1.222222222 | 0.699471799 | 1 | 1 |
| MAP4K4 | 1.222222222 | 0.699471799 | 1 | 1 |
| NEDD4L | 1.222222222 | 0.699471799 | 1 | 1 |
| MAP4K3 | 1.222222222 | 0.699471799 | 1 | 1 |
| SPRED1 | 1.222222222 | 0.699471799 | 1 | 1 |
| OLFM3 | 1.222222222 | 0.699471799 | 1 | 1 |
| PLCH1 | 1.222222222 | 0.699471799 | 1 | 1 |
| ALPI | 1.222222222 | 0.699471799 | 1 | 1 |
| BRDT | 1.222222222 | 0.699471799 | 1 | 1 |
| PLCD4 | 1.222222222 | 0.699471799 | 1 | 1 |
| MAP3K6 | 1.222222222 | 0.699471799 | 1 | 1 |
| NEIL2 | 1.222222222 | 0.699471799 | 1 | 1 |
| ALOX5 | 1.222222222 | 0.699471799 | 1 | 1 |
| NEK10 | 1.222222222 | 0.699471799 | 1 | 1 |
| ALLC | 1.222222222 | 0.699471799 | 1 | 1 |
| OSBPL8 | 1.222222222 | 0.699471799 | 1 | 1 |
| ORC3 | 1.222222222 | 0.699471799 | 1 | 1 |
| PLD6 | 1.222222222 | 0.699471799 | 1 | 1 |
| SMUG1 | 1.222222222 | 0.699471799 | 1 | 1 |
| WDR24 | 1.222222222 | 0.699471799 | 1 | 1 |
| WDR37 | 1.222222222 | 0.699471799 | 1 | 1 |
| SLC6A5 | 1.222222222 | 0.699471799 | 1 | 1 |
| NECAB3 | 1.222222222 | 0.699471799 | 1 | 1 |
| PLBD2 | 1.222222222 | 0.699471799 | 1 | 1 |
| OR10X1 | 1.222222222 | 0.699471799 | 1 | 1 |
| FAM131C | 1.222222222 | 0.699471799 | 1 | 1 |
| MARCH1 | 1.222222222 | 0.699471799 | 1 | 1 |
| AMER2 | 1.222222222 | 0.699471799 | 1 | 1 |
| MARC2 | 1.222222222 | 0.699471799 | 1 | 1 |
| SLK | 1.222222222 | 0.699471799 | 1 | 1 |
| AMBRA1 | 1.222222222 | 0.699471799 | 1 | 1 |
| BPGM | 1.222222222 | 0.699471799 | 1 | 1 |
| BPHL | 1.222222222 | 0.699471799 | 1 | 1 |
| SPPL2A | 1.222222222 | 0.699471799 | 1 | 1 |
| NDUFB9 | 1.222222222 | 0.699471799 | 1 | 1 |
| ALX1 | 1.222222222 | 0.699471799 | 1 | 1 |
| ZNF473 | 1.222222222 | 0.699471799 | 1 | 1 |
| SOX13 | 1.222222222 | 0.699471799 | 1 | 1 |
| BPIFA3 | 1.222222222 | 0.699471799 | 1 | 1 |
| WDR75 | 1.222222222 | 0.699471799 | 1 | 1 |
| SLC35G2 | 1.222222222 | 0.699471799 | 1 | 1 |
| ALPL | 1.222222222 | 0.699471799 | 1 | 1 |
| MAPK7 | 1.222222222 | 0.699471799 | 1 | 1 |
| MAPK6 | 1.222222222 | 0.699471799 | 1 | 1 |
| ALX3 | 1.222222222 | 0.699471799 | 1 | 1 |
| ZNF550 | 1.222222222 | 0.699471799 | 1 | 1 |
| ZNF512 | 1.222222222 | 0.699471799 | 1 | 1 |
| BRSK1 | 1.222222222 | 0.699471799 | 1 | 1 |
| MAMDC2 | 1.222222222 | 0.699471799 | 1 | 1 |
| BTBD7 | 1.222222222 | 0.699471799 | 1 | 1 |
| BTBD9 | 1.222222222 | 0.699471799 | 1 | 1 |
| OLFML1 | 1.222222222 | 0.699471799 | 1 | 1 |
| MAK | 1.222222222 | 0.699471799 | 1 | 1 |
| ZNF536 | 1.222222222 | 0.699471799 | 1 | 1 |
| OR8D1 | 1.222222222 | 0.699471799 | 1 | 1 |
| ZNF540 | 1.222222222 | 0.699471799 | 1 | 1 |
| FANCD2 | 1.222222222 | 0.699471799 | 1 | 1 |
| ZNF529 | 1.222222222 | 0.699471799 | 1 | 1 |
| METTL27 | 1.222222222 | 0.699471799 | 1 | 1 |
| BTG3 | 1.222222222 | 0.699471799 | 1 | 1 |
| SOWAHC | 1.222222222 | 0.699471799 | 1 | 1 |
| RCC1L | 1.222222222 | 0.699471799 | 1 | 1 |
| EPS8L3 | 1.222222222 | 0.699471799 | 1 | 1 |
| OLFML2A | 1.222222222 | 0.699471799 | 1 | 1 |
| SLC35E2 | 1.222222222 | 0.699471799 | 1 | 1 |
| SPRTN | 1.222222222 | 0.699471799 | 1 | 1 |
| ZNF544 | 1.222222222 | 0.699471799 | 1 | 1 |
| WBP11 | 1.222222222 | 0.699471799 | 1 | 1 |
| OR7D4 | 1.222222222 | 0.699471799 | 1 | 1 |
| MAP1S | 1.222222222 | 0.699471799 | 1 | 1 |
| SLC35F5 | 1.222222222 | 0.699471799 | 1 | 1 |
| ALDH5A1 | 1.222222222 | 0.699471799 | 1 | 1 |
| OR2H2 | 1.222222222 | 0.699471799 | 1 | 1 |
| FLYWCH1 | 1.222222222 | 0.699471799 | 1 | 1 |
| BRWD3 | 1.222222222 | 0.699471799 | 1 | 1 |
| OR9Q1 | 1.222222222 | 0.699471799 | 1 | 1 |
| NEU3 | 1.222222222 | 0.699471799 | 1 | 1 |
| BSG | 1.222222222 | 0.699471799 | 1 | 1 |
| PLEKHA8 | 1.222222222 | 0.699471799 | 1 | 1 |
| ZNF518B | 1.222222222 | 0.699471799 | 1 | 1 |
| BSPRY | 1.222222222 | 0.699471799 | 1 | 1 |
| ALDH4A1 | 1.222222222 | 0.699471799 | 1 | 1 |
| BST2 | 1.222222222 | 0.699471799 | 1 | 1 |
| MAN2C1 | 1.222222222 | 0.699471799 | 1 | 1 |
| ZNF519 | 1.222222222 | 0.699471799 | 1 | 1 |
| NEUROD6 | 1.222222222 | 0.699471799 | 1 | 1 |
| ZNF823 | 1.222222222 | 0.699471799 | 1 | 1 |
| WDHD1 | 1.222222222 | 0.699471799 | 1 | 1 |
| ZNF526 | 1.222222222 | 0.699471799 | 1 | 1 |
| MAN1A1 | 1.222222222 | 0.699471799 | 1 | 1 |
| NF1 | 1.222222222 | 0.699471799 | 1 | 1 |
| NF2 | 1.222222222 | 0.699471799 | 1 | 1 |
| ACOT12 | 1.222222222 | 0.699471799 | 1 | 1 |
| VSTM1 | 1.222222222 | 0.699471799 | 1 | 1 |
| ZNF645 | 1.222222222 | 0.699471799 | 1 | 1 |
| ZNF607 | 1.222222222 | 0.699471799 | 1 | 1 |
| SNX20 | 1.222222222 | 0.699471799 | 1 | 1 |
| ADAMTS19 | 1.222222222 | 0.699471799 | 1 | 1 |
| FOXP3 | 1.222222222 | 0.699471799 | 1 | 1 |
| OR10G8 | 1.222222222 | 0.699471799 | 1 | 1 |
| PLPPR2 | 1.222222222 | 0.699471799 | 1 | 1 |
| ZNF763 | 1.222222222 | 0.699471799 | 1 | 1 |
| ZNF764 | 1.222222222 | 0.699471799 | 1 | 1 |
| NTAN1 | 1.222222222 | 0.699471799 | 1 | 1 |
| ADAM9 | 1.222222222 | 0.699471799 | 1 | 1 |
| ZNF776 | 1.222222222 | 0.699471799 | 1 | 1 |
| ADGRL3 | 1.222222222 | 0.699471799 | 1 | 1 |
| ZNF781 | 1.222222222 | 0.699471799 | 1 | 1 |
| OR4M1 | 1.222222222 | 0.699471799 | 1 | 1 |
| ENO4 | 1.222222222 | 0.699471799 | 1 | 1 |
| ZNF782 | 1.222222222 | 0.699471799 | 1 | 1 |
| id47685 | 1.222222222 | 0.699471799 | 1 | 1 |
| ADAM12 | 1.222222222 | 0.699471799 | 1 | 1 |
| ZNF783 | 1.222222222 | 0.699471799 | 1 | 1 |
| NR0B2 | 1.222222222 | 0.699471799 | 1 | 1 |
| SRGAP3 | 1.222222222 | 0.699471799 | 1 | 1 |
| OR4F6 | 1.222222222 | 0.699471799 | 1 | 1 |
| C1QTNF3 | 1.222222222 | 0.699471799 | 1 | 1 |
| OPRK1 | 1.222222222 | 0.699471799 | 1 | 1 |
| LRP6 | 1.222222222 | 0.699471799 | 1 | 1 |
| FOXN1 | 1.222222222 | 0.699471799 | 1 | 1 |
| ENPP2 | 1.222222222 | 0.699471799 | 1 | 1 |
| OR51T1 | 1.222222222 | 0.699471799 | 1 | 1 |
| LRP2BP | 1.222222222 | 0.699471799 | 1 | 1 |
| ERICH4 | 1.222222222 | 0.699471799 | 1 | 1 |
| OPRD1 | 1.222222222 | 0.699471799 | 1 | 1 |
| NPAT | 1.222222222 | 0.699471799 | 1 | 1 |
| NPB | 1.222222222 | 0.699471799 | 1 | 1 |
| ADAP1 | 1.222222222 | 0.699471799 | 1 | 1 |
| POMC | 1.222222222 | 0.699471799 | 1 | 1 |
| LRMP | 1.222222222 | 0.699471799 | 1 | 1 |
| NPFFR1 | 1.222222222 | 0.699471799 | 1 | 1 |
| OR51E2 | 1.222222222 | 0.699471799 | 1 | 1 |
| C1D | 1.222222222 | 0.699471799 | 1 | 1 |
| C1QA | 1.222222222 | 0.699471799 | 1 | 1 |
| ADAMTS8 | 1.222222222 | 0.699471799 | 1 | 1 |
| ZNF75D | 1.222222222 | 0.699471799 | 1 | 1 |
| ADAMTS6 | 1.222222222 | 0.699471799 | 1 | 1 |
| C1QTNF2 | 1.222222222 | 0.699471799 | 1 | 1 |
| ADCY7 | 1.222222222 | 0.699471799 | 1 | 1 |
| ADA | 1.222222222 | 0.699471799 | 1 | 1 |
| NR2C1 | 1.222222222 | 0.699471799 | 1 | 1 |
| ACTL7B | 1.222222222 | 0.699471799 | 1 | 1 |
| OR2T3 | 1.222222222 | 0.699471799 | 1 | 1 |
| NSUN6 | 1.222222222 | 0.699471799 | 1 | 1 |
| SRRM1 | 1.222222222 | 0.699471799 | 1 | 1 |
| USP25 | 1.222222222 | 0.699471799 | 1 | 1 |
| OR2T6 | 1.222222222 | 0.699471799 | 1 | 1 |
| SLC27A6 | 1.222222222 | 0.699471799 | 1 | 1 |
| ACSS3 | 1.222222222 | 0.699471799 | 1 | 1 |
| LOC390956 | 1.222222222 | 0.699471799 | 1 | 1 |
| PPFIA3 | 1.222222222 | 0.699471799 | 1 | 1 |
| OR2T5 | 1.222222222 | 0.699471799 | 1 | 1 |
| NSD1 | 1.222222222 | 0.699471799 | 1 | 1 |
| NSFL1C | 1.222222222 | 0.699471799 | 1 | 1 |
| ZNF790 | 1.222222222 | 0.699471799 | 1 | 1 |
| LOC377711 | 1.222222222 | 0.699471799 | 1 | 1 |
| ZNF791 | 1.222222222 | 0.699471799 | 1 | 1 |
| SLC27A5 | 1.222222222 | 0.699471799 | 1 | 1 |
| NSMF | 1.222222222 | 0.699471799 | 1 | 1 |
| C2CD4D | 1.222222222 | 0.699471799 | 1 | 1 |
| PLPP2 | 1.222222222 | 0.699471799 | 1 | 1 |
| MINDY1 | 1.222222222 | 0.699471799 | 1 | 1 |
| SRPRB | 1.222222222 | 0.699471799 | 1 | 1 |
| ZNF79 | 1.222222222 | 0.699471799 | 1 | 1 |
| OR4F15 | 1.222222222 | 0.699471799 | 1 | 1 |
| NR3C2 | 1.222222222 | 0.699471799 | 1 | 1 |
| FAM57A | 1.222222222 | 0.699471799 | 1 | 1 |
| C1orf195 | 1.222222222 | 0.699471799 | 1 | 1 |
| C1orf198 | 1.222222222 | 0.699471799 | 1 | 1 |
| LONRF3 | 1.222222222 | 0.699471799 | 1 | 1 |
| SNTG1 | 1.222222222 | 0.699471799 | 1 | 1 |
| OR4C46 | 1.222222222 | 0.699471799 | 1 | 1 |
| POU3F3 | 1.222222222 | 0.699471799 | 1 | 1 |
| ACSF2 | 1.222222222 | 0.699471799 | 1 | 1 |
| POU5F2 | 1.222222222 | 0.699471799 | 1 | 1 |
| ZNF789 | 1.222222222 | 0.699471799 | 1 | 1 |
| OR4C12 | 1.222222222 | 0.699471799 | 1 | 1 |
| SLC28A2 | 1.222222222 | 0.699471799 | 1 | 1 |
| OR2T27 | 1.222222222 | 0.699471799 | 1 | 1 |
| USP29 | 1.222222222 | 0.699471799 | 1 | 1 |
| OR4A15 | 1.222222222 | 0.699471799 | 1 | 1 |
| PPA2 | 1.222222222 | 0.699471799 | 1 | 1 |
| FPR2 | 1.222222222 | 0.699471799 | 1 | 1 |
| ACTL9 | 1.222222222 | 0.699471799 | 1 | 1 |
| POLR3B | 1.222222222 | 0.699471799 | 1 | 1 |
| VASH1 | 1.222222222 | 0.699471799 | 1 | 1 |
| LRRC14B | 1.222222222 | 0.699471799 | 1 | 1 |
| CFAP54 | 1.222222222 | 0.699471799 | 1 | 1 |
| LSR | 1.222222222 | 0.699471799 | 1 | 1 |
| SLC36A1 | 1.222222222 | 0.699471799 | 1 | 1 |
| FOSL1 | 1.222222222 | 0.699471799 | 1 | 1 |
| ZNF653 | 1.222222222 | 0.699471799 | 1 | 1 |
| NMD3 | 1.222222222 | 0.699471799 | 1 | 1 |
| NTPCR | 1.222222222 | 0.699471799 | 1 | 1 |
| DGLUCY | 1.222222222 | 0.699471799 | 1 | 1 |
| VOPP1 | 1.222222222 | 0.699471799 | 1 | 1 |
| NME1-NME2 | 1.222222222 | 0.699471799 | 1 | 1 |
| POC1A | 1.222222222 | 0.699471799 | 1 | 1 |
| VNN2 | 1.222222222 | 0.699471799 | 1 | 1 |
| NNMT | 1.222222222 | 0.699471799 | 1 | 1 |
| FOXC1 | 1.222222222 | 0.699471799 | 1 | 1 |
| PODXL | 1.222222222 | 0.699471799 | 1 | 1 |
| SLC33A1 | 1.222222222 | 0.699471799 | 1 | 1 |
| POFUT2 | 1.222222222 | 0.699471799 | 1 | 1 |
| ACPP | 1.222222222 | 0.699471799 | 1 | 1 |
| C15orf39 | 1.222222222 | 0.699471799 | 1 | 1 |
| NTRK1 | 1.222222222 | 0.699471799 | 1 | 1 |
| FOXD3 | 1.222222222 | 0.699471799 | 1 | 1 |
| VPS28 | 1.222222222 | 0.699471799 | 1 | 1 |
| PNN | 1.222222222 | 0.699471799 | 1 | 1 |
| PMP2 | 1.222222222 | 0.699471799 | 1 | 1 |
| PMS1 | 1.222222222 | 0.699471799 | 1 | 1 |
| OR10J3 | 1.222222222 | 0.699471799 | 1 | 1 |
| VRK3 | 1.222222222 | 0.699471799 | 1 | 1 |
| AGXT2 | 1.222222222 | 0.699471799 | 1 | 1 |
| MAJIN | 1.222222222 | 0.699471799 | 1 | 1 |
| PNKD | 1.222222222 | 0.699471799 | 1 | 1 |
| PNLDC1 | 1.222222222 | 0.699471799 | 1 | 1 |
| ZNF610 | 1.222222222 | 0.699471799 | 1 | 1 |
| SMC1B | 1.222222222 | 0.699471799 | 1 | 1 |
| EPC2 | 1.222222222 | 0.699471799 | 1 | 1 |
| C11orf94 | 1.222222222 | 0.699471799 | 1 | 1 |
| LPCAT1 | 1.222222222 | 0.699471799 | 1 | 1 |
| OR5H2 | 1.222222222 | 0.699471799 | 1 | 1 |
| C12orf40 | 1.222222222 | 0.699471799 | 1 | 1 |
| OR5H1 | 1.222222222 | 0.699471799 | 1 | 1 |
| ZNF621 | 1.222222222 | 0.699471799 | 1 | 1 |
| AGGF1 | 1.222222222 | 0.699471799 | 1 | 1 |
| FAM46B | 1.222222222 | 0.699471799 | 1 | 1 |
| SLC35A4 | 1.222222222 | 0.699471799 | 1 | 1 |
| FAM20A | 1.222222222 | 0.699471799 | 1 | 1 |
| OR52I2 | 1.222222222 | 0.699471799 | 1 | 1 |
| OR52E8 | 1.222222222 | 0.699471799 | 1 | 1 |
| OR52B6 | 1.222222222 | 0.699471799 | 1 | 1 |
| SLC2A9 | 1.222222222 | 0.699471799 | 1 | 1 |
| FOXI2 | 1.222222222 | 0.699471799 | 1 | 1 |
| NTN1 | 1.222222222 | 0.699471799 | 1 | 1 |
| ACR | 1.222222222 | 0.699471799 | 1 | 1 |
| GCNA | 1.222222222 | 0.699471799 | 1 | 1 |
| ZNF726 | 1.222222222 | 0.699471799 | 1 | 1 |
| LRRC34 | 1.222222222 | 0.699471799 | 1 | 1 |
| ADD2 | 1.222222222 | 0.699471799 | 1 | 1 |
| OPN3 | 1.222222222 | 0.699471799 | 1 | 1 |
| LRRC3 | 1.222222222 | 0.699471799 | 1 | 1 |
| OR52A5 | 1.222222222 | 0.699471799 | 1 | 1 |
| VAT1L | 1.222222222 | 0.699471799 | 1 | 1 |
| ADCY8 | 1.222222222 | 0.699471799 | 1 | 1 |
| POLM | 1.222222222 | 0.699471799 | 1 | 1 |
| POLR1A | 1.222222222 | 0.699471799 | 1 | 1 |
| OR51V1 | 1.222222222 | 0.699471799 | 1 | 1 |
| ENPP5 | 1.222222222 | 0.699471799 | 1 | 1 |
| POLR1E | 1.222222222 | 0.699471799 | 1 | 1 |
| ADIPOR2 | 1.222222222 | 0.699471799 | 1 | 1 |
| OPN1MW2 | 1.222222222 | 0.699471799 | 1 | 1 |
| SPEM2 | 1.222222222 | 0.699471799 | 1 | 1 |
| HEATR9 | 1.222222222 | 0.699471799 | 1 | 1 |
| LRRC56 | 1.222222222 | 0.699471799 | 1 | 1 |
| VLDLR | 1.222222222 | 0.699471799 | 1 | 1 |
| GAS8-AS1 | 1.222222222 | 0.699471799 | 1 | 1 |
| LRRC4C | 1.222222222 | 0.699471799 | 1 | 1 |
| VIM | 1.222222222 | 0.699471799 | 1 | 1 |
| NOL4 | 1.222222222 | 0.699471799 | 1 | 1 |
| AADACL3 | 1.222222222 | 0.699471799 | 1 | 1 |
| FAM168A | 1.222222222 | 0.699471799 | 1 | 1 |
| ENTPD6 | 1.222222222 | 0.699471799 | 1 | 1 |
| NKX1-2 | 1.222222222 | 0.699471799 | 1 | 1 |
| NOM1 | 1.222222222 | 0.699471799 | 1 | 1 |
| LRRC42 | 1.222222222 | 0.699471799 | 1 | 1 |
| ZNF70 | 1.222222222 | 0.699471799 | 1 | 1 |
| ABRAXAS1 | 1.222222222 | 0.699471799 | 1 | 1 |
| OR10H3 | 1.222222222 | 0.699471799 | 1 | 1 |
| VGF | 1.222222222 | 0.699471799 | 1 | 1 |
| C17orf47 | 1.222222222 | 0.699471799 | 1 | 1 |
| VEGFB | 1.222222222 | 0.699471799 | 1 | 1 |
| FOXG1 | 1.222222222 | 0.699471799 | 1 | 1 |
| ZNF705G | 1.222222222 | 0.699471799 | 1 | 1 |
| ADTRP | 1.222222222 | 0.699471799 | 1 | 1 |
| ZNF446 | 1.222222222 | 0.699471799 | 1 | 1 |
| AMY2A | 1.222222222 | 0.699471799 | 1 | 1 |
| BMX | 1.222222222 | 0.699471799 | 1 | 1 |
| ATP10D | 1.222222222 | 0.699471799 | 1 | 1 |
| SLC44A3 | 1.222222222 | 0.699471799 | 1 | 1 |
| ATP12A | 1.222222222 | 0.699471799 | 1 | 1 |
| MVD | 1.222222222 | 0.699471799 | 1 | 1 |
| ABRA | 1.222222222 | 0.699471799 | 1 | 1 |
| ZNF215 | 1.222222222 | 0.699471799 | 1 | 1 |
| OR1F1 | 1.222222222 | 0.699471799 | 1 | 1 |
| APOL5 | 1.222222222 | 0.699471799 | 1 | 1 |
| APOL4 | 1.222222222 | 0.699471799 | 1 | 1 |
| ZNF212 | 1.222222222 | 0.699471799 | 1 | 1 |
| APOL2 | 1.222222222 | 0.699471799 | 1 | 1 |
| APOL1 | 1.222222222 | 0.699471799 | 1 | 1 |
| ATP1A3 | 1.222222222 | 0.699471799 | 1 | 1 |
| ATP1A4 | 1.222222222 | 0.699471799 | 1 | 1 |
| MYADML2 | 1.222222222 | 0.699471799 | 1 | 1 |
| APOF | 1.222222222 | 0.699471799 | 1 | 1 |
| ZSCAN12 | 1.222222222 | 0.699471799 | 1 | 1 |
| APOE | 1.222222222 | 0.699471799 | 1 | 1 |
| APOBEC3G | 1.222222222 | 0.699471799 | 1 | 1 |
| PGBD2 | 1.222222222 | 0.699471799 | 1 | 1 |
| ZBTB48 | 1.222222222 | 0.699471799 | 1 | 1 |
| OR1D5 | 1.222222222 | 0.699471799 | 1 | 1 |
| NXPE3 | 1.222222222 | 0.699471799 | 1 | 1 |
| SMYD3 | 1.222222222 | 0.699471799 | 1 | 1 |
| PEX12 | 1.222222222 | 0.699471799 | 1 | 1 |
| MOB3A | 1.222222222 | 0.699471799 | 1 | 1 |
| FEN1 | 1.222222222 | 0.699471799 | 1 | 1 |
| ATF5 | 1.222222222 | 0.699471799 | 1 | 1 |
| UQCC2 | 1.222222222 | 0.699471799 | 1 | 1 |
| SPATA33 | 1.222222222 | 0.699471799 | 1 | 1 |
| ATF7IP2 | 1.222222222 | 0.699471799 | 1 | 1 |
| ZNF154 | 1.222222222 | 0.699471799 | 1 | 1 |
| MUC1 | 1.222222222 | 0.699471799 | 1 | 1 |
| ATOH8 | 1.222222222 | 0.699471799 | 1 | 1 |
| ARFGEF1 | 1.222222222 | 0.699471799 | 1 | 1 |
| FBXO36 | 1.222222222 | 0.699471799 | 1 | 1 |
| SNAP47 | 1.222222222 | 0.699471799 | 1 | 1 |
| NOL3 | 1.222222222 | 0.699471799 | 1 | 1 |
| SLC9A3R1 | 1.222222222 | 0.699471799 | 1 | 1 |
| PCDHB3 | 1.222222222 | 0.699471799 | 1 | 1 |
| ABLIM3 | 1.222222222 | 0.699471799 | 1 | 1 |
| MUS81 | 1.222222222 | 0.699471799 | 1 | 1 |
| ZNF205 | 1.222222222 | 0.699471799 | 1 | 1 |
| ATMIN | 1.222222222 | 0.699471799 | 1 | 1 |
| ARFGAP2 | 1.222222222 | 0.699471799 | 1 | 1 |
| ZCCHC14 | 1.222222222 | 0.699471799 | 1 | 1 |
| RFLNA | 1.222222222 | 0.699471799 | 1 | 1 |
| OR2AT4 | 1.222222222 | 0.699471799 | 1 | 1 |
| ZAR1 | 1.222222222 | 0.699471799 | 1 | 1 |
| FGD6 | 1.222222222 | 0.699471799 | 1 | 1 |
| ATP8A1 | 1.222222222 | 0.699471799 | 1 | 1 |
| PBK | 1.222222222 | 0.699471799 | 1 | 1 |
| ATP8B4 | 1.222222222 | 0.699471799 | 1 | 1 |
| ZADH2 | 1.222222222 | 0.699471799 | 1 | 1 |
| PHF11 | 1.222222222 | 0.699471799 | 1 | 1 |
| YY1 | 1.222222222 | 0.699471799 | 1 | 1 |
| ABCD3 | 1.222222222 | 0.699471799 | 1 | 1 |
| MYL5 | 1.222222222 | 0.699471799 | 1 | 1 |
| YWHAB | 1.222222222 | 0.699471799 | 1 | 1 |
| ABCD1 | 1.222222222 | 0.699471799 | 1 | 1 |
| PHF20L1 | 1.222222222 | 0.699471799 | 1 | 1 |
| ZSCAN30 | 1.222222222 | 0.699471799 | 1 | 1 |
| SPDYE4 | 1.222222222 | 0.699471799 | 1 | 1 |
| MID1 | 1.222222222 | 0.699471799 | 1 | 1 |
| MYLK4 | 1.222222222 | 0.699471799 | 1 | 1 |
| PAWR | 1.222222222 | 0.699471799 | 1 | 1 |
| ZNF860 | 1.222222222 | 0.699471799 | 1 | 1 |
| PATL2 | 1.222222222 | 0.699471799 | 1 | 1 |
| FGF10 | 1.222222222 | 0.699471799 | 1 | 1 |
| APOBEC3D | 1.222222222 | 0.699471799 | 1 | 1 |
| ZNF226 | 1.222222222 | 0.699471799 | 1 | 1 |
| ZBED2 | 1.222222222 | 0.699471799 | 1 | 1 |
| PCDHA1 | 1.222222222 | 0.699471799 | 1 | 1 |
| ZNF92 | 1.222222222 | 0.699471799 | 1 | 1 |
| ACAD8 | 1.222222222 | 0.699471799 | 1 | 1 |
| SLC43A1 | 1.222222222 | 0.699471799 | 1 | 1 |
| ZSCAN2 | 1.222222222 | 0.699471799 | 1 | 1 |
| MLH3 | 1.222222222 | 0.699471799 | 1 | 1 |
| MYH14 | 1.222222222 | 0.699471799 | 1 | 1 |
| ZSCAN22 | 1.222222222 | 0.699471799 | 1 | 1 |
| APMAP | 1.222222222 | 0.699471799 | 1 | 1 |
| ABCF1 | 1.222222222 | 0.699471799 | 1 | 1 |
| FBXL6 | 1.222222222 | 0.699471799 | 1 | 1 |
| PCDH19 | 1.222222222 | 0.699471799 | 1 | 1 |
| PCDH17 | 1.222222222 | 0.699471799 | 1 | 1 |
| APH1B | 1.222222222 | 0.699471799 | 1 | 1 |
| MKRN2 | 1.222222222 | 0.699471799 | 1 | 1 |
| APEX2 | 1.222222222 | 0.699471799 | 1 | 1 |
| ATP5S | 1.222222222 | 0.699471799 | 1 | 1 |
| ATP6V0D2 | 1.222222222 | 0.699471799 | 1 | 1 |
| ZBTB20 | 1.222222222 | 0.699471799 | 1 | 1 |
| FGD4 | 1.222222222 | 0.699471799 | 1 | 1 |
| ZSCAN26 | 1.222222222 | 0.699471799 | 1 | 1 |
| PCDHGA11 | 1.222222222 | 0.699471799 | 1 | 1 |
| FEM1C | 1.222222222 | 0.699471799 | 1 | 1 |
| PEX11A | 1.222222222 | 0.699471799 | 1 | 1 |
| MSL3 | 1.222222222 | 0.699471799 | 1 | 1 |
| SLC7A13 | 1.222222222 | 0.699471799 | 1 | 1 |
| MRPL37 | 1.222222222 | 0.699471799 | 1 | 1 |
| ZFAND6 | 1.222222222 | 0.699471799 | 1 | 1 |
| FAM229A | 1.222222222 | 0.699471799 | 1 | 1 |
| PDIA5 | 1.222222222 | 0.699471799 | 1 | 1 |
| PDIA6 | 1.222222222 | 0.699471799 | 1 | 1 |
| NVL | 1.222222222 | 0.699471799 | 1 | 1 |
| ARSF | 1.222222222 | 0.699471799 | 1 | 1 |
| PCOLCE2 | 1.222222222 | 0.699471799 | 1 | 1 |
| SLC5A7 | 1.222222222 | 0.699471799 | 1 | 1 |
| MSX2 | 1.222222222 | 0.699471799 | 1 | 1 |
| FBXO7 | 1.222222222 | 0.699471799 | 1 | 1 |
| SPATA18 | 1.222222222 | 0.699471799 | 1 | 1 |
| MTA3 | 1.222222222 | 0.699471799 | 1 | 1 |
| F8 | 1.222222222 | 0.699471799 | 1 | 1 |
| MTERF1 | 1.222222222 | 0.699471799 | 1 | 1 |
| SMYD5 | 1.222222222 | 0.699471799 | 1 | 1 |
| MTERF4 | 1.222222222 | 0.699471799 | 1 | 1 |
| ABHD16B | 1.222222222 | 0.699471799 | 1 | 1 |
| ARSJ | 1.222222222 | 0.699471799 | 1 | 1 |
| ABHD16A | 1.222222222 | 0.699471799 | 1 | 1 |
| ZGLP1 | 1.222222222 | 0.699471799 | 1 | 1 |
| ARNT2 | 1.222222222 | 0.699471799 | 1 | 1 |
| ARL6IP6 | 1.222222222 | 0.699471799 | 1 | 1 |
| F11R | 1.222222222 | 0.699471799 | 1 | 1 |
| PDE11A | 1.222222222 | 0.699471799 | 1 | 1 |
| ARL13A | 1.222222222 | 0.699471799 | 1 | 1 |
| ARL8B | 1.222222222 | 0.699471799 | 1 | 1 |
| PDE1A | 1.222222222 | 0.699471799 | 1 | 1 |
| MS4A4E | 1.222222222 | 0.699471799 | 1 | 1 |
| ZFP90 | 1.222222222 | 0.699471799 | 1 | 1 |
| ARMC10 | 1.222222222 | 0.699471799 | 1 | 1 |
| MRPL46 | 1.222222222 | 0.699471799 | 1 | 1 |
| MRPS24 | 1.222222222 | 0.699471799 | 1 | 1 |
| SLC6A2 | 1.222222222 | 0.699471799 | 1 | 1 |
| ZFP36L2 | 1.222222222 | 0.699471799 | 1 | 1 |
| FBXW4 | 1.222222222 | 0.699471799 | 1 | 1 |
| FCER1A | 1.222222222 | 0.699471799 | 1 | 1 |
| SLC6A17 | 1.222222222 | 0.699471799 | 1 | 1 |
| ARID5B | 1.222222222 | 0.699471799 | 1 | 1 |
| MRPL49 | 1.222222222 | 0.699471799 | 1 | 1 |
| PCSK2 | 1.222222222 | 0.699471799 | 1 | 1 |
| MSH3 | 1.222222222 | 0.699471799 | 1 | 1 |
| PDE4B | 1.222222222 | 0.699471799 | 1 | 1 |
| OR1J2 | 1.222222222 | 0.699471799 | 1 | 1 |
| MTERF2 | 1.222222222 | 0.699471799 | 1 | 1 |
| MRI1 | 1.222222222 | 0.699471799 | 1 | 1 |
| MPHOSPH8 | 1.222222222 | 0.699471799 | 1 | 1 |
| ASGR2 | 1.222222222 | 0.699471799 | 1 | 1 |
| MPHOSPH10 | 1.222222222 | 0.699471799 | 1 | 1 |
| SLC4A1 | 1.222222222 | 0.699471799 | 1 | 1 |
| ARHGAP30 | 1.222222222 | 0.699471799 | 1 | 1 |
| MPEG1 | 1.222222222 | 0.699471799 | 1 | 1 |
| ASIC4 | 1.222222222 | 0.699471799 | 1 | 1 |
| FAAH | 1.222222222 | 0.699471799 | 1 | 1 |
| SLC47A2 | 1.222222222 | 0.699471799 | 1 | 1 |
| ASGR1 | 1.222222222 | 0.699471799 | 1 | 1 |
| ASNSD1 | 1.222222222 | 0.699471799 | 1 | 1 |
| ZCWPW1 | 1.222222222 | 0.699471799 | 1 | 1 |
| SPATA1 | 1.222222222 | 0.699471799 | 1 | 1 |
| ZNF133 | 1.222222222 | 0.699471799 | 1 | 1 |
| MTRR | 1.222222222 | 0.699471799 | 1 | 1 |
| ASRGL1 | 1.222222222 | 0.699471799 | 1 | 1 |
| ASTE1 | 1.222222222 | 0.699471799 | 1 | 1 |
| OR2A2 | 1.222222222 | 0.699471799 | 1 | 1 |
| ZCCHC4 | 1.222222222 | 0.699471799 | 1 | 1 |
| MOGAT3 | 1.222222222 | 0.699471799 | 1 | 1 |
| FDXACB1 | 1.222222222 | 0.699471799 | 1 | 1 |
| MPL | 1.222222222 | 0.699471799 | 1 | 1 |
| ZNHIT2 | 1.222222222 | 0.699471799 | 1 | 1 |
| ASCL2 | 1.222222222 | 0.699471799 | 1 | 1 |
| EXOSC3 | 1.222222222 | 0.699471799 | 1 | 1 |
| ASAH2 | 1.222222222 | 0.699471799 | 1 | 1 |
| PDSS1 | 1.222222222 | 0.699471799 | 1 | 1 |
| ASB13 | 1.222222222 | 0.699471799 | 1 | 1 |
| SNIP1 | 1.222222222 | 0.699471799 | 1 | 1 |
| MRC2 | 1.222222222 | 0.699471799 | 1 | 1 |
| MPZL2 | 1.222222222 | 0.699471799 | 1 | 1 |
| MTFMT | 1.222222222 | 0.699471799 | 1 | 1 |
| ASB3 | 1.222222222 | 0.699471799 | 1 | 1 |
| MTFP1 | 1.222222222 | 0.699471799 | 1 | 1 |
| ARHGAP9 | 1.222222222 | 0.699471799 | 1 | 1 |
| MTHFD2L | 1.222222222 | 0.699471799 | 1 | 1 |
| SLC7A7 | 1.222222222 | 0.699471799 | 1 | 1 |
| PCDHGB5 | 1.222222222 | 0.699471799 | 1 | 1 |
| PCDHGB2 | 1.222222222 | 0.699471799 | 1 | 1 |
| PCDHGB1 | 1.222222222 | 0.699471799 | 1 | 1 |
| SLC4A2 | 1.222222222 | 0.699471799 | 1 | 1 |
| ZNF100 | 1.222222222 | 0.699471799 | 1 | 1 |
| ASCC1 | 1.222222222 | 0.699471799 | 1 | 1 |
| MTMR11 | 1.222222222 | 0.699471799 | 1 | 1 |
| MTMR14 | 1.222222222 | 0.699471799 | 1 | 1 |
| MICU2 | 1.222222222 | 0.699471799 | 1 | 1 |
| MICALL1 | 1.222222222 | 0.699471799 | 1 | 1 |
| SP6 | 1.222222222 | 0.699471799 | 1 | 1 |
| MYO1B | 1.222222222 | 0.699471799 | 1 | 1 |
| SPN | 1.222222222 | 0.699471799 | 1 | 1 |
| NCBP1 | 1.222222222 | 0.699471799 | 1 | 1 |
| SPNS2 | 1.222222222 | 0.699471799 | 1 | 1 |
| OR11H4 | 1.222222222 | 0.699471799 | 1 | 1 |
| ANKDD1B | 1.222222222 | 0.699471799 | 1 | 1 |
| MDH1B | 1.222222222 | 0.699471799 | 1 | 1 |
| NCK1 | 1.222222222 | 0.699471799 | 1 | 1 |
| ZNF429 | 1.222222222 | 0.699471799 | 1 | 1 |
| PIPOX | 1.222222222 | 0.699471799 | 1 | 1 |
| ANKFN1 | 1.222222222 | 0.699471799 | 1 | 1 |
| FIS1 | 1.222222222 | 0.699471799 | 1 | 1 |
| P4HA1 | 1.222222222 | 0.699471799 | 1 | 1 |
| MCOLN3 | 1.222222222 | 0.699471799 | 1 | 1 |
| FASTKD3 | 1.222222222 | 0.699471799 | 1 | 1 |
| NCOA1 | 1.222222222 | 0.699471799 | 1 | 1 |
| RTL8B | 1.222222222 | 0.699471799 | 1 | 1 |
| BCOR | 1.222222222 | 0.699471799 | 1 | 1 |
| ANGEL1 | 1.222222222 | 0.699471799 | 1 | 1 |
| MCM3 | 1.222222222 | 0.699471799 | 1 | 1 |
| FJX1 | 1.222222222 | 0.699471799 | 1 | 1 |
| FAM3A | 1.222222222 | 0.699471799 | 1 | 1 |
| FKBP10 | 1.222222222 | 0.699471799 | 1 | 1 |
| MEA1 | 1.222222222 | 0.699471799 | 1 | 1 |
| NCAPH | 1.222222222 | 0.699471799 | 1 | 1 |
| ZNF385C | 1.222222222 | 0.699471799 | 1 | 1 |
| ANKRD29 | 1.222222222 | 0.699471799 | 1 | 1 |
| ODF3 | 1.222222222 | 0.699471799 | 1 | 1 |
| ANKRD23 | 1.222222222 | 0.699471799 | 1 | 1 |
| MED23 | 1.222222222 | 0.699471799 | 1 | 1 |
| MED19 | 1.222222222 | 0.699471799 | 1 | 1 |
| SLITRK1 | 1.222222222 | 0.699471799 | 1 | 1 |
| WWC3 | 1.222222222 | 0.699471799 | 1 | 1 |
| PACSIN2 | 1.222222222 | 0.699471799 | 1 | 1 |
| MEAF6 | 1.222222222 | 0.699471799 | 1 | 1 |
| ANKRD13D | 1.222222222 | 0.699471799 | 1 | 1 |
| ODF4 | 1.222222222 | 0.699471799 | 1 | 1 |
| OR2F1 | 1.222222222 | 0.699471799 | 1 | 1 |
| ANKRD13B | 1.222222222 | 0.699471799 | 1 | 1 |
| ZNF408 | 1.222222222 | 0.699471799 | 1 | 1 |
| OFCC1 | 1.222222222 | 0.699471799 | 1 | 1 |
| SLC38A10 | 1.222222222 | 0.699471799 | 1 | 1 |
| BCL11A | 1.222222222 | 0.699471799 | 1 | 1 |
| PIP4K2A | 1.222222222 | 0.699471799 | 1 | 1 |
| ANKRD1 | 1.222222222 | 0.699471799 | 1 | 1 |
| BCDIN3D | 1.222222222 | 0.699471799 | 1 | 1 |
| NCR2 | 1.222222222 | 0.699471799 | 1 | 1 |
| P2RX6 | 1.222222222 | 0.699471799 | 1 | 1 |
| OGFOD1 | 1.222222222 | 0.699471799 | 1 | 1 |
| BIN3 | 1.222222222 | 0.699471799 | 1 | 1 |
| NDST4 | 1.222222222 | 0.699471799 | 1 | 1 |
| BIRC5 | 1.222222222 | 0.699471799 | 1 | 1 |
| AMY1C | 1.222222222 | 0.699471799 | 1 | 1 |
| OXCT2 | 1.222222222 | 0.699471799 | 1 | 1 |
| BLOC1S1 | 1.222222222 | 0.699471799 | 1 | 1 |
| OR2G3 | 1.222222222 | 0.699471799 | 1 | 1 |
| SLC36A3 | 1.222222222 | 0.699471799 | 1 | 1 |
| AMT | 1.222222222 | 0.699471799 | 1 | 1 |
| PKNOX2 | 1.222222222 | 0.699471799 | 1 | 1 |
| FARSA | 1.222222222 | 0.699471799 | 1 | 1 |
| BMP15 | 1.222222222 | 0.699471799 | 1 | 1 |
| MARVELD1 | 1.222222222 | 0.699471799 | 1 | 1 |
| WFDC12 | 1.222222222 | 0.699471799 | 1 | 1 |
| id589972 | 1.222222222 | 0.699471799 | 1 | 1 |
| SLITRK5 | 1.222222222 | 0.699471799 | 1 | 1 |
| SLC36A2 | 1.222222222 | 0.699471799 | 1 | 1 |
| ERC1 | 1.222222222 | 0.699471799 | 1 | 1 |
| FAM198B | 1.222222222 | 0.699471799 | 1 | 1 |
| WDR93 | 1.222222222 | 0.699471799 | 1 | 1 |
| ZNF444 | 1.222222222 | 0.699471799 | 1 | 1 |
| OGG1 | 1.222222222 | 0.699471799 | 1 | 1 |
| PDCD7 | 1.222222222 | 0.699471799 | 1 | 1 |
| WISP1 | 1.222222222 | 0.699471799 | 1 | 1 |
| OGFOD3 | 1.222222222 | 0.699471799 | 1 | 1 |
| WNT2B | 1.222222222 | 0.699471799 | 1 | 1 |
| P2RX4 | 1.222222222 | 0.699471799 | 1 | 1 |
| NDC1 | 1.222222222 | 0.699471799 | 1 | 1 |
| BEST1 | 1.222222222 | 0.699471799 | 1 | 1 |
| BEST2 | 1.222222222 | 0.699471799 | 1 | 1 |
| PJA2 | 1.222222222 | 0.699471799 | 1 | 1 |
| NDC80 | 1.222222222 | 0.699471799 | 1 | 1 |
| FKBP4 | 1.222222222 | 0.699471799 | 1 | 1 |
| AASS | 1.222222222 | 0.699471799 | 1 | 1 |
| SLITRK4 | 1.222222222 | 0.699471799 | 1 | 1 |
| BEST4 | 1.222222222 | 0.699471799 | 1 | 1 |
| OXR1 | 1.222222222 | 0.699471799 | 1 | 1 |
| FKBP10 | 1.222222222 | 0.699471799 | 1 | 1 |
| ZNF430 | 1.222222222 | 0.699471799 | 1 | 1 |
| AMZ2 | 1.222222222 | 0.699471799 | 1 | 1 |
| ZYG11A | 1.222222222 | 0.699471799 | 1 | 1 |
| NDRG2 | 1.222222222 | 0.699471799 | 1 | 1 |
| ZNF431 | 1.222222222 | 0.699471799 | 1 | 1 |
| BHMT2 | 1.222222222 | 0.699471799 | 1 | 1 |
| FKBPL | 1.222222222 | 0.699471799 | 1 | 1 |
| MED26 | 1.222222222 | 0.699471799 | 1 | 1 |
| SOX14 | 1.222222222 | 0.699471799 | 1 | 1 |
| PAFAH2 | 1.222222222 | 0.699471799 | 1 | 1 |
| ZNF365 | 1.222222222 | 0.699471799 | 1 | 1 |
| OR13C9 | 1.222222222 | 0.699471799 | 1 | 1 |
| SPESP1 | 1.222222222 | 0.699471799 | 1 | 1 |
| MYT1L | 1.222222222 | 0.699471799 | 1 | 1 |
| MFSD12 | 1.222222222 | 0.699471799 | 1 | 1 |
| ANXA4 | 1.222222222 | 0.699471799 | 1 | 1 |
| MFSD11 | 1.222222222 | 0.699471799 | 1 | 1 |
| ZNF285 | 1.222222222 | 0.699471799 | 1 | 1 |
| MFSD10 | 1.222222222 | 0.699471799 | 1 | 1 |
| PARD6A | 1.222222222 | 0.699471799 | 1 | 1 |
| ZNF273 | 1.222222222 | 0.699471799 | 1 | 1 |
| YBX3 | 1.222222222 | 0.699471799 | 1 | 1 |
| YAP1 | 1.222222222 | 0.699471799 | 1 | 1 |
| MFN2 | 1.222222222 | 0.699471799 | 1 | 1 |
| N4BP3 | 1.222222222 | 0.699471799 | 1 | 1 |
| EEF1AKMT1 | 1.222222222 | 0.699471799 | 1 | 1 |
| ACAT1 | 1.222222222 | 0.699471799 | 1 | 1 |
| PHOSPHO1 | 1.222222222 | 0.699471799 | 1 | 1 |
| FBLN7 | 1.222222222 | 0.699471799 | 1 | 1 |
| B3GNTL1 | 1.222222222 | 0.699471799 | 1 | 1 |
| B4GALNT4 | 1.222222222 | 0.699471799 | 1 | 1 |
| SPART | 1.222222222 | 0.699471799 | 1 | 1 |
| ZNF319 | 1.222222222 | 0.699471799 | 1 | 1 |
| B3GALT2 | 1.222222222 | 0.699471799 | 1 | 1 |
| PARP15 | 1.222222222 | 0.699471799 | 1 | 1 |
| AP3M1 | 1.222222222 | 0.699471799 | 1 | 1 |
| ZNF256 | 1.222222222 | 0.699471799 | 1 | 1 |
| YIPF5 | 1.222222222 | 0.699471799 | 1 | 1 |
| ZPR1 | 1.222222222 | 0.699471799 | 1 | 1 |
| YIPF3 | 1.222222222 | 0.699471799 | 1 | 1 |
| AWAT2 | 1.222222222 | 0.699471799 | 1 | 1 |
| MGRN1 | 1.222222222 | 0.699471799 | 1 | 1 |
| MYO5A | 1.222222222 | 0.699471799 | 1 | 1 |
| MYO6 | 1.222222222 | 0.699471799 | 1 | 1 |
| FAM117A | 1.222222222 | 0.699471799 | 1 | 1 |
| SP140L | 1.222222222 | 0.699471799 | 1 | 1 |
| PARP6 | 1.222222222 | 0.699471799 | 1 | 1 |
| PARP2 | 1.222222222 | 0.699471799 | 1 | 1 |
| FAM114A1 | 1.222222222 | 0.699471799 | 1 | 1 |
| AP1B1 | 1.222222222 | 0.699471799 | 1 | 1 |
| MYOZ1 | 1.222222222 | 0.699471799 | 1 | 1 |
| FGF21 | 1.222222222 | 0.699471799 | 1 | 1 |
| PARP16 | 1.222222222 | 0.699471799 | 1 | 1 |
| MGARP | 1.222222222 | 0.699471799 | 1 | 1 |
| SLC39A6 | 1.222222222 | 0.699471799 | 1 | 1 |
| PARP8 | 1.222222222 | 0.699471799 | 1 | 1 |
| SLFN12 | 1.222222222 | 0.699471799 | 1 | 1 |
| B9D2 | 1.222222222 | 0.699471799 | 1 | 1 |
| PAOX | 1.222222222 | 0.699471799 | 1 | 1 |
| RNF2 | 1.222222222 | 0.699471799 | 1 | 1 |
| FBF1 | 1.222222222 | 0.699471799 | 1 | 1 |
| BARX1 | 1.222222222 | 0.699471799 | 1 | 1 |
| MEOX1 | 1.222222222 | 0.699471799 | 1 | 1 |
| OR13C5 | 1.222222222 | 0.699471799 | 1 | 1 |
| ABCB8 | 1.222222222 | 0.699471799 | 1 | 1 |
| OR2D3 | 1.222222222 | 0.699471799 | 1 | 1 |
| XPNPEP1 | 1.222222222 | 0.699471799 | 1 | 1 |
| NAPRT | 1.222222222 | 0.699471799 | 1 | 1 |
| MEPCE | 1.222222222 | 0.699471799 | 1 | 1 |
| PIGN | 1.222222222 | 0.699471799 | 1 | 1 |
| ANKRD50 | 1.222222222 | 0.699471799 | 1 | 1 |
| BAZ1B | 1.222222222 | 0.699471799 | 1 | 1 |
| PIK3AP1 | 1.222222222 | 0.699471799 | 1 | 1 |
| ANKRD46 | 1.222222222 | 0.699471799 | 1 | 1 |
| SLC39A11 | 1.222222222 | 0.699471799 | 1 | 1 |
| BBS2 | 1.222222222 | 0.699471799 | 1 | 1 |
| WASHC2C | 1.222222222 | 0.699471799 | 1 | 1 |
| XKR5 | 1.222222222 | 0.699471799 | 1 | 1 |
| PAICS | 1.222222222 | 0.699471799 | 1 | 1 |
| NAPSA | 1.222222222 | 0.699471799 | 1 | 1 |
| FAM122B | 1.222222222 | 0.699471799 | 1 | 1 |
| TLNRD1 | 1.222222222 | 0.699471799 | 1 | 1 |
| NANOS3 | 1.222222222 | 0.699471799 | 1 | 1 |
| NACC1 | 1.222222222 | 0.699471799 | 1 | 1 |
| BAAT | 1.222222222 | 0.699471799 | 1 | 1 |
| METTL3 | 1.222222222 | 0.699471799 | 1 | 1 |
| SLFN14 | 1.222222222 | 0.699471799 | 1 | 1 |
| BACE2 | 1.222222222 | 0.699471799 | 1 | 1 |
| OCEL1 | 1.222222222 | 0.699471799 | 1 | 1 |
| PIAS2 | 1.222222222 | 0.699471799 | 1 | 1 |
| NAGLU | 1.222222222 | 0.699471799 | 1 | 1 |
| ANKS1B | 1.222222222 | 0.699471799 | 1 | 1 |
| BAG2 | 1.222222222 | 0.699471799 | 1 | 1 |
| SLC39A12 | 1.222222222 | 0.699471799 | 1 | 1 |
| NAIF1 | 1.222222222 | 0.699471799 | 1 | 1 |
| BAGE2 | 1.222222222 | 0.699471799 | 1 | 1 |
| METTL15 | 1.222222222 | 0.699471799 | 1 | 1 |
| METTL13 | 1.222222222 | 0.699471799 | 1 | 1 |
| NUDT22 | 1.222222222 | 0.699471799 | 1 | 1 |
| ZNF343 | 1.222222222 | 0.699471799 | 1 | 1 |
| BAIAP2L1 | 1.222222222 | 0.699471799 | 1 | 1 |
| PALB2 | 1.222222222 | 0.699471799 | 1 | 1 |
| ACBD4 | 1.222222222 | 0.699471799 | 1 | 1 |
| MEST | 1.222222222 | 0.699471799 | 1 | 1 |
| SLC38A1 | 1.222222222 | 0.699471799 | 1 | 1 |
| ACSL3 | 1.222222222 | 0.699471799 | 1 | 1 |
| GPR27 | 1.222222222 | 0.699471799 | 1 | 1 |
| FCAMR | 1.222222222 | 0.699471799 | 1 | 1 |
| TNNI1 | 1.222222222 | 0.699471799 | 1 | 1 |
| KANK1 | 1.222222222 | 0.699471799 | 1 | 1 |
| TNKS2 | 1.222222222 | 0.699471799 | 1 | 1 |
| DVL2 | 1.222222222 | 0.699471799 | 1 | 1 |
| HCCS | 1.222222222 | 0.699471799 | 1 | 1 |
| RBMX2 | 1.222222222 | 0.699471799 | 1 | 1 |
| JRK | 1.222222222 | 0.699471799 | 1 | 1 |
| HCFC1 | 1.222222222 | 0.699471799 | 1 | 1 |
| SIRT4 | 1.222222222 | 0.699471799 | 1 | 1 |
| RBP4 | 1.222222222 | 0.699471799 | 1 | 1 |
| TCP10L | 1.222222222 | 0.699471799 | 1 | 1 |
| JMJD7 | 1.222222222 | 0.699471799 | 1 | 1 |
| TNFRSF21 | 1.222222222 | 0.699471799 | 1 | 1 |
| CHGA | 1.222222222 | 0.699471799 | 1 | 1 |
| DVL1 | 1.222222222 | 0.699471799 | 1 | 1 |
| CHM | 1.222222222 | 0.699471799 | 1 | 1 |
| CHML | 1.222222222 | 0.699471799 | 1 | 1 |
| CHMP1B | 1.222222222 | 0.699471799 | 1 | 1 |
| TNFRSF11B | 1.222222222 | 0.699471799 | 1 | 1 |
| DCBLD1 | 1.222222222 | 0.699471799 | 1 | 1 |
| TNFRSF10D | 1.222222222 | 0.699471799 | 1 | 1 |
| TNFRSF10B | 1.222222222 | 0.699471799 | 1 | 1 |
| TNFAIP8L2 | 1.222222222 | 0.699471799 | 1 | 1 |
| DUSP9 | 1.222222222 | 0.699471799 | 1 | 1 |
| RECK | 1.222222222 | 0.699471799 | 1 | 1 |
| SCARA5 | 1.222222222 | 0.699471799 | 1 | 1 |
| GFOD2 | 1.222222222 | 0.699471799 | 1 | 1 |
| CHRNA1 | 1.222222222 | 0.699471799 | 1 | 1 |
| ITPR1 | 1.222222222 | 0.699471799 | 1 | 1 |
| KANSL1 | 1.222222222 | 0.699471799 | 1 | 1 |
| PPIAL4E | 1.222222222 | 0.699471799 | 1 | 1 |
| TNNT1 | 1.222222222 | 0.699471799 | 1 | 1 |
| RBM33 | 1.222222222 | 0.699471799 | 1 | 1 |
| KCNH5 | 1.222222222 | 0.699471799 | 1 | 1 |
| KCNH4 | 1.222222222 | 0.699471799 | 1 | 1 |
| SKIV2L2 | 1.222222222 | 0.699471799 | 1 | 1 |
| RBCK1 | 1.222222222 | 0.699471799 | 1 | 1 |
| TOX3 | 1.222222222 | 0.699471799 | 1 | 1 |
| CEP192 | 1.222222222 | 0.699471799 | 1 | 1 |
| TOR4A | 1.222222222 | 0.699471799 | 1 | 1 |
| DCPS | 1.222222222 | 0.699471799 | 1 | 1 |
| CERCAM | 1.222222222 | 0.699471799 | 1 | 1 |
| TOMM40 | 1.222222222 | 0.699471799 | 1 | 1 |
| SUGP2 | 1.222222222 | 0.699471799 | 1 | 1 |
| KCNA7 | 1.222222222 | 0.699471799 | 1 | 1 |
| GDPD2 | 1.222222222 | 0.699471799 | 1 | 1 |
| CERS4 | 1.222222222 | 0.699471799 | 1 | 1 |
| GPC5 | 1.222222222 | 0.699471799 | 1 | 1 |
| TOLLIP | 1.222222222 | 0.699471799 | 1 | 1 |
| CES4A | 1.222222222 | 0.699471799 | 1 | 1 |
| DCLRE1B | 1.222222222 | 0.699471799 | 1 | 1 |
| RBM19 | 1.222222222 | 0.699471799 | 1 | 1 |
| DCLK2 | 1.222222222 | 0.699471799 | 1 | 1 |
| CETP | 1.222222222 | 0.699471799 | 1 | 1 |
| GPBAR1 | 1.222222222 | 0.699471799 | 1 | 1 |
| TNS4 | 1.222222222 | 0.699471799 | 1 | 1 |
| CFP | 1.222222222 | 0.699471799 | 1 | 1 |
| HCAR1 | 1.222222222 | 0.699471799 | 1 | 1 |
| CGREF1 | 1.222222222 | 0.699471799 | 1 | 1 |
| KAT5 | 1.222222222 | 0.699471799 | 1 | 1 |
| KAT2B | 1.222222222 | 0.699471799 | 1 | 1 |
| TNNT2 | 1.222222222 | 0.699471799 | 1 | 1 |
| SCCPDH | 1.222222222 | 0.699471799 | 1 | 1 |
| DCSTAMP | 1.222222222 | 0.699471799 | 1 | 1 |
| ITPK1 | 1.222222222 | 0.699471799 | 1 | 1 |
| CHRNA5 | 1.222222222 | 0.699471799 | 1 | 1 |
| IRS4 | 1.222222222 | 0.699471799 | 1 | 1 |
| DNAJB1 | 1.222222222 | 0.699471799 | 1 | 1 |
| DUSP13 | 1.222222222 | 0.699471799 | 1 | 1 |
| SULT4A1 | 1.222222222 | 0.699471799 | 1 | 1 |
| ACOD1 | 1.222222222 | 0.699471799 | 1 | 1 |
| DBX1 | 1.222222222 | 0.699471799 | 1 | 1 |
| RFTN1 | 1.222222222 | 0.699471799 | 1 | 1 |
| IRF4 | 1.222222222 | 0.699471799 | 1 | 1 |
| DUS2 | 1.222222222 | 0.699471799 | 1 | 1 |
| IRAK4 | 1.222222222 | 0.699471799 | 1 | 1 |
| CLCN1 | 1.222222222 | 0.699471799 | 1 | 1 |
| IRAK1BP1 | 1.222222222 | 0.699471799 | 1 | 1 |
| TMEM67 | 1.222222222 | 0.699471799 | 1 | 1 |
| HDHD2 | 1.222222222 | 0.699471799 | 1 | 1 |
| TMEM57 | 1.222222222 | 0.699471799 | 1 | 1 |
| GHR | 1.222222222 | 0.699471799 | 1 | 1 |
| TDO2 | 1.222222222 | 0.699471799 | 1 | 1 |
| RFX7 | 1.222222222 | 0.699471799 | 1 | 1 |
| DTX4 | 1.222222222 | 0.699471799 | 1 | 1 |
| TMEM52 | 1.222222222 | 0.699471799 | 1 | 1 |
| TMEM51 | 1.222222222 | 0.699471799 | 1 | 1 |
| RGL1 | 1.222222222 | 0.699471799 | 1 | 1 |
| CLEC12A | 1.222222222 | 0.699471799 | 1 | 1 |
| SIGIRR | 1.222222222 | 0.699471799 | 1 | 1 |
| RGS1 | 1.222222222 | 0.699471799 | 1 | 1 |
| INVS | 1.222222222 | 0.699471799 | 1 | 1 |
| INTS8 | 1.222222222 | 0.699471799 | 1 | 1 |
| TMEM41A | 1.222222222 | 0.699471799 | 1 | 1 |
| SIDT1 | 1.222222222 | 0.699471799 | 1 | 1 |
| TMEM89 | 1.222222222 | 0.699471799 | 1 | 1 |
| CHRNA4 | 1.222222222 | 0.699471799 | 1 | 1 |
| CKAP2L | 1.222222222 | 0.699471799 | 1 | 1 |
| IRX6 | 1.222222222 | 0.699471799 | 1 | 1 |
| ITK | 1.222222222 | 0.699471799 | 1 | 1 |
| ITIH6 | 1.222222222 | 0.699471799 | 1 | 1 |
| ITIH4 | 1.222222222 | 0.699471799 | 1 | 1 |
| REEP5 | 1.222222222 | 0.699471799 | 1 | 1 |
| DUSP28 | 1.222222222 | 0.699471799 | 1 | 1 |
| RELL2 | 1.222222222 | 0.699471799 | 1 | 1 |
| CHRNB1 | 1.222222222 | 0.699471799 | 1 | 1 |
| TCP11L2 | 1.222222222 | 0.699471799 | 1 | 1 |
| TMPRSS2 | 1.222222222 | 0.699471799 | 1 | 1 |
| HDAC5 | 1.222222222 | 0.699471799 | 1 | 1 |
| SCAPER | 1.222222222 | 0.699471799 | 1 | 1 |
| ITGB1BP2 | 1.222222222 | 0.699471799 | 1 | 1 |
| SULT1A2 | 1.222222222 | 0.699471799 | 1 | 1 |
| GFRAL | 1.222222222 | 0.699471799 | 1 | 1 |
| REM2 | 1.222222222 | 0.699471799 | 1 | 1 |
| CHST13 | 1.222222222 | 0.699471799 | 1 | 1 |
| CHST3 | 1.222222222 | 0.699471799 | 1 | 1 |
| ITGA9 | 1.222222222 | 0.699471799 | 1 | 1 |
| TMIE | 1.222222222 | 0.699471799 | 1 | 1 |
| CHUK | 1.222222222 | 0.699471799 | 1 | 1 |
| SIM2 | 1.222222222 | 0.699471799 | 1 | 1 |
| ITGA10 | 1.222222222 | 0.699471799 | 1 | 1 |
| CIB1 | 1.222222222 | 0.699471799 | 1 | 1 |
| MYMK | 1.222222222 | 0.699471799 | 1 | 1 |
| ISX | 1.222222222 | 0.699471799 | 1 | 1 |
| DUSP2 | 1.222222222 | 0.699471799 | 1 | 1 |
| ISM2 | 1.222222222 | 0.699471799 | 1 | 1 |
| SULT1C4 | 1.222222222 | 0.699471799 | 1 | 1 |
| TMEM8B | 1.222222222 | 0.699471799 | 1 | 1 |
| TAF1D | 1.222222222 | 0.699471799 | 1 | 1 |
| CENPV | 1.222222222 | 0.699471799 | 1 | 1 |
| KCNJ10 | 1.222222222 | 0.699471799 | 1 | 1 |
| HAPLN3 | 1.222222222 | 0.699471799 | 1 | 1 |
| CDC16 | 1.222222222 | 0.699471799 | 1 | 1 |
| EDN3 | 1.222222222 | 0.699471799 | 1 | 1 |
| SYCP2 | 1.222222222 | 0.699471799 | 1 | 1 |
| TRIB3 | 1.222222222 | 0.699471799 | 1 | 1 |
| CDC25C | 1.222222222 | 0.699471799 | 1 | 1 |
| GUF1 | 1.222222222 | 0.699471799 | 1 | 1 |
| CDC37L1 | 1.222222222 | 0.699471799 | 1 | 1 |
| SLC16A1 | 1.222222222 | 0.699471799 | 1 | 1 |
| RAB29 | 1.222222222 | 0.699471799 | 1 | 1 |
| SLC15A5 | 1.222222222 | 0.699471799 | 1 | 1 |
| GPR107 | 1.222222222 | 0.699471799 | 1 | 1 |
| EDDM3A | 1.222222222 | 0.699471799 | 1 | 1 |
| LARGE2 | 1.222222222 | 0.699471799 | 1 | 1 |
| DDX41 | 1.222222222 | 0.699471799 | 1 | 1 |
| TRBV7-4 | 1.222222222 | 0.699471799 | 1 | 1 |
| CDCP1 | 1.222222222 | 0.699471799 | 1 | 1 |
| CDH13 | 1.222222222 | 0.699471799 | 1 | 1 |
| DMTF1 | 1.222222222 | 0.699471799 | 1 | 1 |
| EDC4 | 1.222222222 | 0.699471799 | 1 | 1 |
| TRBD1 | 1.222222222 | 0.699471799 | 1 | 1 |
| CDH8 | 1.222222222 | 0.699471799 | 1 | 1 |
| KIF1BP | 1.222222222 | 0.699471799 | 1 | 1 |
| STRN3 | 1.222222222 | 0.699471799 | 1 | 1 |
| GCA | 1.222222222 | 0.699471799 | 1 | 1 |
| RAET1G | 1.222222222 | 0.699471799 | 1 | 1 |
| ECI1 | 1.222222222 | 0.699471799 | 1 | 1 |
| ECHS1 | 1.222222222 | 0.699471799 | 1 | 1 |
| STT3A | 1.222222222 | 0.699471799 | 1 | 1 |
| KIAA0930 | 1.222222222 | 0.699471799 | 1 | 1 |
| DMRT2 | 1.222222222 | 0.699471799 | 1 | 1 |
| KIAA0895 | 1.222222222 | 0.699471799 | 1 | 1 |
| CDA | 1.222222222 | 0.699471799 | 1 | 1 |
| RAB18 | 1.222222222 | 0.699471799 | 1 | 1 |
| DEFA5 | 1.222222222 | 0.699471799 | 1 | 1 |
| PYGM | 1.222222222 | 0.699471799 | 1 | 1 |
| KLF17 | 1.222222222 | 0.699471799 | 1 | 1 |
| SCYL1 | 1.222222222 | 0.699471799 | 1 | 1 |
| GAPDH | 1.222222222 | 0.699471799 | 1 | 1 |
| STMND1 | 1.222222222 | 0.699471799 | 1 | 1 |
| SLC18A2 | 1.222222222 | 0.699471799 | 1 | 1 |
| TRIM45 | 1.222222222 | 0.699471799 | 1 | 1 |
| SLC18A1 | 1.222222222 | 0.699471799 | 1 | 1 |
| QKI | 1.222222222 | 0.699471799 | 1 | 1 |
| CD300A | 1.222222222 | 0.699471799 | 1 | 1 |
| CD300LG | 1.222222222 | 0.699471799 | 1 | 1 |
| TRIM41 | 1.222222222 | 0.699471799 | 1 | 1 |
| CD3EAP | 1.222222222 | 0.699471799 | 1 | 1 |
| EEFSEC | 1.222222222 | 0.699471799 | 1 | 1 |
| STON1 | 1.222222222 | 0.699471799 | 1 | 1 |
| KIFAP3 | 1.222222222 | 0.699471799 | 1 | 1 |
| SCUBE3 | 1.222222222 | 0.699471799 | 1 | 1 |
| TRIM33 | 1.222222222 | 0.699471799 | 1 | 1 |
| TBPL2 | 1.222222222 | 0.699471799 | 1 | 1 |
| DMRT1 | 1.222222222 | 0.699471799 | 1 | 1 |
| KIF5A | 1.222222222 | 0.699471799 | 1 | 1 |
| R3HDM2 | 1.222222222 | 0.699471799 | 1 | 1 |
| KIF3C | 1.222222222 | 0.699471799 | 1 | 1 |
| SLC17A9 | 1.222222222 | 0.699471799 | 1 | 1 |
| CD82 | 1.222222222 | 0.699471799 | 1 | 1 |
| SLC17A8 | 1.222222222 | 0.699471799 | 1 | 1 |
| SERPINE3 | 1.222222222 | 0.699471799 | 1 | 1 |
| CD96 | 1.222222222 | 0.699471799 | 1 | 1 |
| SLC17A6 | 1.222222222 | 0.699471799 | 1 | 1 |
| STT3B | 1.222222222 | 0.699471799 | 1 | 1 |
| RALB | 1.222222222 | 0.699471799 | 1 | 1 |
| KIAA0513 | 1.222222222 | 0.699471799 | 1 | 1 |
| CEACAM4 | 1.222222222 | 0.699471799 | 1 | 1 |
| RARRES1 | 1.222222222 | 0.699471799 | 1 | 1 |
| HAAO | 1.222222222 | 0.699471799 | 1 | 1 |
| SLC10A1 | 1.222222222 | 0.699471799 | 1 | 1 |
| HABP2 | 1.222222222 | 0.699471799 | 1 | 1 |
| TPRX1 | 1.222222222 | 0.699471799 | 1 | 1 |
| DDI2 | 1.222222222 | 0.699471799 | 1 | 1 |
| HAL | 1.222222222 | 0.699471799 | 1 | 1 |
| KCNS2 | 1.222222222 | 0.699471799 | 1 | 1 |
| STYK1 | 1.222222222 | 0.699471799 | 1 | 1 |
| KCNQ4 | 1.222222222 | 0.699471799 | 1 | 1 |
| TPP1 | 1.222222222 | 0.699471799 | 1 | 1 |
| CELA3A | 1.222222222 | 0.699471799 | 1 | 1 |
| SCML4 | 1.222222222 | 0.699471799 | 1 | 1 |
| TCF25 | 1.222222222 | 0.699471799 | 1 | 1 |
| SLAIN2 | 1.222222222 | 0.699471799 | 1 | 1 |
| CELF4 | 1.222222222 | 0.699471799 | 1 | 1 |
| KCNN1 | 1.222222222 | 0.699471799 | 1 | 1 |
| TCF7L2 | 1.222222222 | 0.699471799 | 1 | 1 |
| CENPA | 1.222222222 | 0.699471799 | 1 | 1 |
| DCUN1D2 | 1.222222222 | 0.699471799 | 1 | 1 |
| TPH2 | 1.222222222 | 0.699471799 | 1 | 1 |
| CENPCP1 | 1.222222222 | 0.699471799 | 1 | 1 |
| CENPH | 1.222222222 | 0.699471799 | 1 | 1 |
| RAVER2 | 1.222222222 | 0.699471799 | 1 | 1 |
| TPD52L2 | 1.222222222 | 0.699471799 | 1 | 1 |
| TPD52L1 | 1.222222222 | 0.699471799 | 1 | 1 |
| SKOR1 | 1.222222222 | 0.699471799 | 1 | 1 |
| CENPT | 1.222222222 | 0.699471799 | 1 | 1 |
| KCTD19 | 1.222222222 | 0.699471799 | 1 | 1 |
| SESN2 | 1.222222222 | 0.699471799 | 1 | 1 |
| CEACAM20 | 1.222222222 | 0.699471799 | 1 | 1 |
| KDELC2 | 1.222222222 | 0.699471799 | 1 | 1 |
| DDX27 | 1.222222222 | 0.699471799 | 1 | 1 |
| TBXA2R | 1.222222222 | 0.699471799 | 1 | 1 |
| TCEANC2 | 1.222222222 | 0.699471799 | 1 | 1 |
| STX10 | 1.222222222 | 0.699471799 | 1 | 1 |
| STX16 | 1.222222222 | 0.699471799 | 1 | 1 |
| KIAA0232 | 1.222222222 | 0.699471799 | 1 | 1 |
| EBF4 | 1.222222222 | 0.699471799 | 1 | 1 |
| RANBP10 | 1.222222222 | 0.699471799 | 1 | 1 |
| TRAV12-1 | 1.222222222 | 0.699471799 | 1 | 1 |
| TRAPPC11 | 1.222222222 | 0.699471799 | 1 | 1 |
| EBF1 | 1.222222222 | 0.699471799 | 1 | 1 |
| KHNYN | 1.222222222 | 0.699471799 | 1 | 1 |
| CDKN2AIP | 1.222222222 | 0.699471799 | 1 | 1 |
| KERA | 1.222222222 | 0.699471799 | 1 | 1 |
| INTS3 | 1.222222222 | 0.699471799 | 1 | 1 |
| KEAP1 | 1.222222222 | 0.699471799 | 1 | 1 |
| CDPF1 | 1.222222222 | 0.699471799 | 1 | 1 |
| STX3 | 1.222222222 | 0.699471799 | 1 | 1 |
| E2F6 | 1.222222222 | 0.699471799 | 1 | 1 |
| GPN2 | 1.222222222 | 0.699471799 | 1 | 1 |
| CDX1 | 1.222222222 | 0.699471799 | 1 | 1 |
| TCERG1 | 1.222222222 | 0.699471799 | 1 | 1 |
| CDYL | 1.222222222 | 0.699471799 | 1 | 1 |
| TRAF3IP2 | 1.222222222 | 0.699471799 | 1 | 1 |
| CEACAM16 | 1.222222222 | 0.699471799 | 1 | 1 |
| RXRA | 1.222222222 | 0.699471799 | 1 | 1 |
| RAPGEF6 | 1.222222222 | 0.699471799 | 1 | 1 |
| KDM1A | 1.222222222 | 0.699471799 | 1 | 1 |
| TCF19 | 1.222222222 | 0.699471799 | 1 | 1 |
| RAPGEFL1 | 1.222222222 | 0.699471799 | 1 | 1 |
| CDKN2B | 1.222222222 | 0.699471799 | 1 | 1 |
| CD1C | 1.222222222 | 0.699471799 | 1 | 1 |
| RGS22 | 1.222222222 | 0.699471799 | 1 | 1 |
| RGS9BP | 1.222222222 | 0.699471799 | 1 | 1 |
| HUS1B | 1.222222222 | 0.699471799 | 1 | 1 |
| HTRA4 | 1.222222222 | 0.699471799 | 1 | 1 |
| RPL13A | 1.222222222 | 0.699471799 | 1 | 1 |
| HTR3E | 1.222222222 | 0.699471799 | 1 | 1 |
| CRX | 1.222222222 | 0.699471799 | 1 | 1 |
| SGSH | 1.222222222 | 0.699471799 | 1 | 1 |
| CRYGN | 1.222222222 | 0.699471799 | 1 | 1 |
| RPL6 | 1.222222222 | 0.699471799 | 1 | 1 |
| RPL8 | 1.222222222 | 0.699471799 | 1 | 1 |
| GNB1L | 1.222222222 | 0.699471799 | 1 | 1 |
| TIGD6 | 1.222222222 | 0.699471799 | 1 | 1 |
| HIST1H1E | 1.222222222 | 0.699471799 | 1 | 1 |
| DOK5 | 1.222222222 | 0.699471799 | 1 | 1 |
| RPN1 | 1.222222222 | 0.699471799 | 1 | 1 |
| SYT12 | 1.222222222 | 0.699471799 | 1 | 1 |
| CSGALNACT1 | 1.222222222 | 0.699471799 | 1 | 1 |
| HSPA6 | 1.222222222 | 0.699471799 | 1 | 1 |
| CSGALNACT2 | 1.222222222 | 0.699471799 | 1 | 1 |
| CSH2 | 1.222222222 | 0.699471799 | 1 | 1 |
| RPP25 | 1.222222222 | 0.699471799 | 1 | 1 |
| HSPA14 | 1.222222222 | 0.699471799 | 1 | 1 |
| SFXN3 | 1.222222222 | 0.699471799 | 1 | 1 |
| DOHH | 1.222222222 | 0.699471799 | 1 | 1 |
| HIST1H2AB | 1.222222222 | 0.699471799 | 1 | 1 |
| RPS6KA1 | 1.222222222 | 0.699471799 | 1 | 1 |
| HIST1H2AD | 1.222222222 | 0.699471799 | 1 | 1 |
| THUMPD1 | 1.222222222 | 0.699471799 | 1 | 1 |
| HSDL2 | 1.222222222 | 0.699471799 | 1 | 1 |
| RPS6KA4 | 1.222222222 | 0.699471799 | 1 | 1 |
| HIRA | 1.222222222 | 0.699471799 | 1 | 1 |
| RPS6KA5 | 1.222222222 | 0.699471799 | 1 | 1 |
| CRIPT | 1.222222222 | 0.699471799 | 1 | 1 |
| CYP2J2 | 1.222222222 | 0.699471799 | 1 | 1 |
| CPA4 | 1.222222222 | 0.699471799 | 1 | 1 |
| TM9SF1 | 1.222222222 | 0.699471799 | 1 | 1 |
| ROPN1B | 1.222222222 | 0.699471799 | 1 | 1 |
| HOMER2 | 1.222222222 | 0.699471799 | 1 | 1 |
| DPH7 | 1.222222222 | 0.699471799 | 1 | 1 |
| SYNDIG1L | 1.222222222 | 0.699471799 | 1 | 1 |
| DPH1 | 1.222222222 | 0.699471799 | 1 | 1 |
| IFIT1B | 1.222222222 | 0.699471799 | 1 | 1 |
| IFI6 | 1.222222222 | 0.699471799 | 1 | 1 |
| GM2A | 1.222222222 | 0.699471799 | 1 | 1 |
| RP9 | 1.222222222 | 0.699471799 | 1 | 1 |
| SH2B3 | 1.222222222 | 0.699471799 | 1 | 1 |
| HIF3A | 1.222222222 | 0.699471799 | 1 | 1 |
| RPAP2 | 1.222222222 | 0.699471799 | 1 | 1 |
| SAMD15 | 1.222222222 | 0.699471799 | 1 | 1 |
| IDNK | 1.222222222 | 0.699471799 | 1 | 1 |
| CYP3A5 | 1.222222222 | 0.699471799 | 1 | 1 |
| CR1L | 1.222222222 | 0.699471799 | 1 | 1 |
| CRAMP1 | 1.222222222 | 0.699471799 | 1 | 1 |
| RPE65 | 1.222222222 | 0.699471799 | 1 | 1 |
| RPGR | 1.222222222 | 0.699471799 | 1 | 1 |
| GMIP | 1.222222222 | 0.699471799 | 1 | 1 |
| ICA1L | 1.222222222 | 0.699471799 | 1 | 1 |
| HIPK1 | 1.222222222 | 0.699471799 | 1 | 1 |
| GMNC | 1.222222222 | 0.699471799 | 1 | 1 |
| CYP2R1 | 1.222222222 | 0.699471799 | 1 | 1 |
| RPL10L | 1.222222222 | 0.699471799 | 1 | 1 |
| TLE1 | 1.222222222 | 0.699471799 | 1 | 1 |
| CRHR2 | 1.222222222 | 0.699471799 | 1 | 1 |
| TLDC1 | 1.222222222 | 0.699471799 | 1 | 1 |
| ROCK2 | 1.222222222 | 0.699471799 | 1 | 1 |
| THSD1 | 1.222222222 | 0.699471799 | 1 | 1 |
| THPO | 1.222222222 | 0.699471799 | 1 | 1 |
| HOXC12 | 1.222222222 | 0.699471799 | 1 | 1 |
| HOXC10 | 1.222222222 | 0.699471799 | 1 | 1 |
| HOXB5 | 1.222222222 | 0.699471799 | 1 | 1 |
| HIST1H4K | 1.222222222 | 0.699471799 | 1 | 1 |
| SAFB | 1.222222222 | 0.699471799 | 1 | 1 |
| CYP20A1 | 1.222222222 | 0.699471799 | 1 | 1 |
| CX3CR1 | 1.222222222 | 0.699471799 | 1 | 1 |
| CXADR | 1.222222222 | 0.699471799 | 1 | 1 |
| RTL1 | 1.222222222 | 0.699471799 | 1 | 1 |
| SFMBT1 | 1.222222222 | 0.699471799 | 1 | 1 |
| RTN4 | 1.222222222 | 0.699471799 | 1 | 1 |
| HNRNPDL | 1.222222222 | 0.699471799 | 1 | 1 |
| RTP1 | 1.222222222 | 0.699471799 | 1 | 1 |
| CYLD | 1.222222222 | 0.699471799 | 1 | 1 |
| HNMT | 1.222222222 | 0.699471799 | 1 | 1 |
| CYHR1 | 1.222222222 | 0.699471799 | 1 | 1 |
| DNER | 1.222222222 | 0.699471799 | 1 | 1 |
| RUNDC1 | 1.222222222 | 0.699471799 | 1 | 1 |
| HMX3 | 1.222222222 | 0.699471799 | 1 | 1 |
| SYVN1 | 1.222222222 | 0.699471799 | 1 | 1 |
| TGDS | 1.222222222 | 0.699471799 | 1 | 1 |
| DNASE1 | 1.222222222 | 0.699471799 | 1 | 1 |
| TFPI2 | 1.222222222 | 0.699471799 | 1 | 1 |
| HLA-E | 1.222222222 | 0.699471799 | 1 | 1 |
| RUVBL1 | 1.222222222 | 0.699471799 | 1 | 1 |
| RWDD2A | 1.222222222 | 0.699471799 | 1 | 1 |
| TFAP4 | 1.222222222 | 0.699471799 | 1 | 1 |
| CYB5R1 | 1.222222222 | 0.699471799 | 1 | 1 |
| TFEB | 1.222222222 | 0.699471799 | 1 | 1 |
| GOLPH3L | 1.222222222 | 0.699471799 | 1 | 1 |
| GNL3L | 1.222222222 | 0.699471799 | 1 | 1 |
| RSPH3 | 1.222222222 | 0.699471799 | 1 | 1 |
| CTTN | 1.222222222 | 0.699471799 | 1 | 1 |
| HSD17B1 | 1.222222222 | 0.699471799 | 1 | 1 |
| CT45A5 | 1.222222222 | 0.699471799 | 1 | 1 |
| GNLY | 1.222222222 | 0.699471799 | 1 | 1 |
| CTBS | 1.222222222 | 0.699471799 | 1 | 1 |
| HIST1H2AH | 1.222222222 | 0.699471799 | 1 | 1 |
| THEM6 | 1.222222222 | 0.699471799 | 1 | 1 |
| HS3ST2 | 1.222222222 | 0.699471799 | 1 | 1 |
| SFXN2 | 1.222222222 | 0.699471799 | 1 | 1 |
| VSIG2 | 1.222222222 | 0.699471799 | 1 | 1 |
| HRH3 | 1.222222222 | 0.699471799 | 1 | 1 |
| HRH1 | 1.222222222 | 0.699471799 | 1 | 1 |
| NRG1 | 1.222222222 | 0.699471799 | 1 | 1 |
| TEX10 | 1.222222222 | 0.699471799 | 1 | 1 |
| GNRHR | 1.222222222 | 0.699471799 | 1 | 1 |
| HPSE2 | 1.222222222 | 0.699471799 | 1 | 1 |
| DNAJC15 | 1.222222222 | 0.699471799 | 1 | 1 |
| GNS | 1.222222222 | 0.699471799 | 1 | 1 |
| GOLGA1 | 1.222222222 | 0.699471799 | 1 | 1 |
| HPR | 1.222222222 | 0.699471799 | 1 | 1 |
| RRM2B | 1.222222222 | 0.699471799 | 1 | 1 |
| HPGDS | 1.222222222 | 0.699471799 | 1 | 1 |
| CYP2A6 | 1.222222222 | 0.699471799 | 1 | 1 |
| HP | 1.222222222 | 0.699471799 | 1 | 1 |
| THAP9 | 1.222222222 | 0.699471799 | 1 | 1 |
| SYT2 | 1.222222222 | 0.699471799 | 1 | 1 |
| THAP8 | 1.222222222 | 0.699471799 | 1 | 1 |
| CTSO | 1.222222222 | 0.699471799 | 1 | 1 |
| RSL1D1 | 1.222222222 | 0.699471799 | 1 | 1 |
| THAP7 | 1.222222222 | 0.699471799 | 1 | 1 |
| CYP26C1 | 1.222222222 | 0.699471799 | 1 | 1 |
| ROCK1 | 1.222222222 | 0.699471799 | 1 | 1 |
| SYNC | 1.222222222 | 0.699471799 | 1 | 1 |
| IFT43 | 1.222222222 | 0.699471799 | 1 | 1 |
| TDRD9 | 1.222222222 | 0.699471799 | 1 | 1 |
| IL36RN | 1.222222222 | 0.699471799 | 1 | 1 |
| DSE | 1.222222222 | 0.699471799 | 1 | 1 |
| DAGLA | 1.222222222 | 0.699471799 | 1 | 1 |
| IL31RA | 1.222222222 | 0.699471799 | 1 | 1 |
| DSCR4 | 1.222222222 | 0.699471799 | 1 | 1 |
| SARDH | 1.222222222 | 0.699471799 | 1 | 1 |
| CMTR1 | 1.222222222 | 0.699471799 | 1 | 1 |
| IL22RA1 | 1.222222222 | 0.699471799 | 1 | 1 |
| TMEM192 | 1.222222222 | 0.699471799 | 1 | 1 |
| IL20RA | 1.222222222 | 0.699471799 | 1 | 1 |
| GP2 | 1.222222222 | 0.699471799 | 1 | 1 |
| TMEM191B | 1.222222222 | 0.699471799 | 1 | 1 |
| DNAJC11 | 1.222222222 | 0.699471799 | 1 | 1 |
| CNIH4 | 1.222222222 | 0.699471799 | 1 | 1 |
| RIPK4 | 1.222222222 | 0.699471799 | 1 | 1 |
| SHCBP1L | 1.222222222 | 0.699471799 | 1 | 1 |
| CNN1 | 1.222222222 | 0.699471799 | 1 | 1 |
| IL17RD | 1.222222222 | 0.699471799 | 1 | 1 |
| SAR1A | 1.222222222 | 0.699471799 | 1 | 1 |
| CNNM2 | 1.222222222 | 0.699471799 | 1 | 1 |
| TEF | 1.222222222 | 0.699471799 | 1 | 1 |
| HERPUD2 | 1.222222222 | 0.699471799 | 1 | 1 |
| RMDN3 | 1.222222222 | 0.699471799 | 1 | 1 |
| CYYR1 | 1.222222222 | 0.699471799 | 1 | 1 |
| DSC3 | 1.222222222 | 0.699471799 | 1 | 1 |
| IL12RB2 | 1.222222222 | 0.699471799 | 1 | 1 |
| RMND5B | 1.222222222 | 0.699471799 | 1 | 1 |
| TEKT4 | 1.222222222 | 0.699471799 | 1 | 1 |
| ACKR1 | 1.222222222 | 0.699471799 | 1 | 1 |
| IL10RB | 1.222222222 | 0.699471799 | 1 | 1 |
| IL3RA | 1.222222222 | 0.699471799 | 1 | 1 |
| IL4R | 1.222222222 | 0.699471799 | 1 | 1 |
| INSL3 | 1.222222222 | 0.699471799 | 1 | 1 |
| DTWD2 | 1.222222222 | 0.699471799 | 1 | 1 |
| CLEC6A | 1.222222222 | 0.699471799 | 1 | 1 |
| CLEC9A | 1.222222222 | 0.699471799 | 1 | 1 |
| TDRD1 | 1.222222222 | 0.699471799 | 1 | 1 |
| SURF2 | 1.222222222 | 0.699471799 | 1 | 1 |
| RHBDL1 | 1.222222222 | 0.699471799 | 1 | 1 |
| TMEM251 | 1.222222222 | 0.699471799 | 1 | 1 |
| CLK3 | 1.222222222 | 0.699471799 | 1 | 1 |
| SHQ1 | 1.222222222 | 0.699471799 | 1 | 1 |
| SUSD3 | 1.222222222 | 0.699471799 | 1 | 1 |
| TMEM249 | 1.222222222 | 0.699471799 | 1 | 1 |
| TMEM246 | 1.222222222 | 0.699471799 | 1 | 1 |
| RHOU | 1.222222222 | 0.699471799 | 1 | 1 |
| SHOX2 | 1.222222222 | 0.699471799 | 1 | 1 |
| TMEM229A | 1.222222222 | 0.699471799 | 1 | 1 |
| TMEM222 | 1.222222222 | 0.699471799 | 1 | 1 |
| CLPTM1L | 1.222222222 | 0.699471799 | 1 | 1 |
| ILKAP | 1.222222222 | 0.699471799 | 1 | 1 |
| GJA10 | 1.222222222 | 0.699471799 | 1 | 1 |
| DAXX | 1.222222222 | 0.699471799 | 1 | 1 |
| SBK2 | 1.222222222 | 0.699471799 | 1 | 1 |
| CLUAP1 | 1.222222222 | 0.699471799 | 1 | 1 |
| CLUH | 1.222222222 | 0.699471799 | 1 | 1 |
| SHISA7 | 1.222222222 | 0.699471799 | 1 | 1 |
| TMEM209 | 1.222222222 | 0.699471799 | 1 | 1 |
| IL6R | 1.222222222 | 0.699471799 | 1 | 1 |
| RICTOR | 1.222222222 | 0.699471799 | 1 | 1 |
| TMEM200B | 1.222222222 | 0.699471799 | 1 | 1 |
| DSG2 | 1.222222222 | 0.699471799 | 1 | 1 |
| GOT2 | 1.222222222 | 0.699471799 | 1 | 1 |
| IL10RA | 1.222222222 | 0.699471799 | 1 | 1 |
| RNASE7 | 1.222222222 | 0.699471799 | 1 | 1 |
| RNF224 | 1.222222222 | 0.699471799 | 1 | 1 |
| HHLA2 | 1.222222222 | 0.699471799 | 1 | 1 |
| SYCP2 | 1.222222222 | 0.699471799 | 1 | 1 |
| COL8A2 | 1.222222222 | 0.699471799 | 1 | 1 |
| IGHV3-49 | 1.222222222 | 0.699471799 | 1 | 1 |
| COLEC12 | 1.222222222 | 0.699471799 | 1 | 1 |
| TMED8 | 1.222222222 | 0.699471799 | 1 | 1 |
| IGHV1-69 | 1.222222222 | 0.699471799 | 1 | 1 |
| COMMD3 | 1.222222222 | 0.699471799 | 1 | 1 |
| TERF2 | 1.222222222 | 0.699471799 | 1 | 1 |
| RNF43 | 1.222222222 | 0.699471799 | 1 | 1 |
| GLP1R | 1.222222222 | 0.699471799 | 1 | 1 |
| COMT | 1.222222222 | 0.699471799 | 1 | 1 |
| CYP4B1 | 1.222222222 | 0.699471799 | 1 | 1 |
| GLRA1 | 1.222222222 | 0.699471799 | 1 | 1 |
| TMCO4 | 1.222222222 | 0.699471799 | 1 | 1 |
| IGHD | 1.222222222 | 0.699471799 | 1 | 1 |
| COQ10B | 1.222222222 | 0.699471799 | 1 | 1 |
| SH3BGR | 1.222222222 | 0.699471799 | 1 | 1 |
| TMCC2 | 1.222222222 | 0.699471799 | 1 | 1 |
| COQ5 | 1.222222222 | 0.699471799 | 1 | 1 |
| IGF2BP3 | 1.222222222 | 0.699471799 | 1 | 1 |
| CORIN | 1.222222222 | 0.699471799 | 1 | 1 |
| TMBIM6 | 1.222222222 | 0.699471799 | 1 | 1 |
| COX10 | 1.222222222 | 0.699471799 | 1 | 1 |
| RNPEP | 1.222222222 | 0.699471799 | 1 | 1 |
| TMBIM4 | 1.222222222 | 0.699471799 | 1 | 1 |
| GLT1D1 | 1.222222222 | 0.699471799 | 1 | 1 |
| SYN3 | 1.222222222 | 0.699471799 | 1 | 1 |
| IGHV4-61 | 1.222222222 | 0.699471799 | 1 | 1 |
| SYBU | 1.222222222 | 0.699471799 | 1 | 1 |
| TMEM115 | 1.222222222 | 0.699471799 | 1 | 1 |
| HHLA1 | 1.222222222 | 0.699471799 | 1 | 1 |
| IKZF4 | 1.222222222 | 0.699471799 | 1 | 1 |
| CYS1 | 1.222222222 | 0.699471799 | 1 | 1 |
| IKZF3 | 1.222222222 | 0.699471799 | 1 | 1 |
| HFE2 | 1.222222222 | 0.699471799 | 1 | 1 |
| RNASE10 | 1.222222222 | 0.699471799 | 1 | 1 |
| PCDHA4 | 1.222222222 | 0.699471799 | 1 | 1 |
| CYR61 | 1.222222222 | 0.699471799 | 1 | 1 |
| DROSHA | 1.222222222 | 0.699471799 | 1 | 1 |
| CYP7B1 | 1.222222222 | 0.699471799 | 1 | 1 |
| KCNN4 | 1.222222222 | 0.699471799 | 1 | 1 |
| IHH | 1.222222222 | 0.699471799 | 1 | 1 |
| TMEM169 | 1.222222222 | 0.699471799 | 1 | 1 |
| TMEM165 | 1.222222222 | 0.699471799 | 1 | 1 |
| RNF113B | 1.222222222 | 0.699471799 | 1 | 1 |
| RGS5 | 1.222222222 | 0.699471799 | 1 | 1 |
| CNTNAP2 | 1.222222222 | 0.699471799 | 1 | 1 |
| DR1 | 1.222222222 | 0.699471799 | 1 | 1 |
| TMEM145 | 1.222222222 | 0.699471799 | 1 | 1 |
| IGLV1-47 | 1.222222222 | 0.699471799 | 1 | 1 |
| SAMD7 | 1.222222222 | 0.699471799 | 1 | 1 |
| RNF152 | 1.222222222 | 0.699471799 | 1 | 1 |
| TMEM132D | 1.222222222 | 0.699471799 | 1 | 1 |
| HGSNAT | 1.222222222 | 0.699471799 | 1 | 1 |
| GOT1 | 1.222222222 | 0.699471799 | 1 | 1 |
| TENM1 | 1.222222222 | 0.699471799 | 1 | 1 |
| IGKV2-26 | 1.222222222 | 0.699471799 | 1 | 1 |
| TMEM130 | 1.222222222 | 0.699471799 | 1 | 1 |
| GLIPR2 | 1.222222222 | 0.699471799 | 1 | 1 |
| COL4A3BP | 1.222222222 | 0.699471799 | 1 | 1 |
| SAMD4A | 1.222222222 | 0.699471799 | 1 | 1 |
| IGLV3-27 | 1.222222222 | 0.699471799 | 1 | 1 |
| KLHDC2 | 1.222222222 | 0.699471799 | 1 | 1 |
| RYK | 1.222222222 | 0.699471799 | 1 | 1 |
| TUBB1 | 1.222222222 | 0.699471799 | 1 | 1 |
| C9orf47 | 1.222222222 | 0.699471799 | 1 | 1 |
| SLC25A3 | 1.222222222 | 0.699471799 | 1 | 1 |
| PRAMEF16 | 1.222222222 | 0.699471799 | 1 | 1 |
| C9orf40 | 1.222222222 | 0.699471799 | 1 | 1 |
| ARRDC1-AS1 | 1.222222222 | 0.699471799 | 1 | 1 |
| PRSS16 | 1.222222222 | 0.699471799 | 1 | 1 |
| LALBA | 1.222222222 | 0.699471799 | 1 | 1 |
| LOC100129636 | 1.222222222 | 0.699471799 | 1 | 1 |
| STAM2 | 1.222222222 | 0.699471799 | 1 | 1 |
| C9orf64 | 1.222222222 | 0.699471799 | 1 | 1 |
| UGT2B11 | 1.222222222 | 0.699471799 | 1 | 1 |
| UGT8 | 1.222222222 | 0.699471799 | 1 | 1 |
| GPR149 | 1.222222222 | 0.699471799 | 1 | 1 |
| PRSS37 | 1.222222222 | 0.699471799 | 1 | 1 |
| LACTB | 1.222222222 | 0.699471799 | 1 | 1 |
| LACC1 | 1.222222222 | 0.699471799 | 1 | 1 |
| PRSS45 | 1.222222222 | 0.699471799 | 1 | 1 |
| STAMBP | 1.222222222 | 0.699471799 | 1 | 1 |
| CCDC112 | 1.222222222 | 0.699471799 | 1 | 1 |
| LOC100134391 | 1.222222222 | 0.699471799 | 1 | 1 |
| C9orf139 | 1.222222222 | 0.699471799 | 1 | 1 |
| PRSS12 | 1.222222222 | 0.699471799 | 1 | 1 |
| GRIN1 | 1.222222222 | 0.699471799 | 1 | 1 |
| SLC25A25 | 1.222222222 | 0.699471799 | 1 | 1 |
| CABP7 | 1.222222222 | 0.699471799 | 1 | 1 |
| CABP4 | 1.222222222 | 0.699471799 | 1 | 1 |
| TTC38 | 1.222222222 | 0.699471799 | 1 | 1 |
| UBTFL1 | 1.222222222 | 0.699471799 | 1 | 1 |
| SELL | 1.222222222 | 0.699471799 | 1 | 1 |
| UBXN6 | 1.222222222 | 0.699471799 | 1 | 1 |
| STAG1 | 1.222222222 | 0.699471799 | 1 | 1 |
| CA4 | 1.222222222 | 0.699471799 | 1 | 1 |
| TBC1D15 | 1.222222222 | 0.699471799 | 1 | 1 |
| PRRG3 | 1.222222222 | 0.699471799 | 1 | 1 |
| FUT8 | 1.222222222 | 0.699471799 | 1 | 1 |
| LANCL2 | 1.222222222 | 0.699471799 | 1 | 1 |
| LANCL1 | 1.222222222 | 0.699471799 | 1 | 1 |
| SLC25A21 | 1.222222222 | 0.699471799 | 1 | 1 |
| UCP3 | 1.222222222 | 0.699471799 | 1 | 1 |
| EIF3F | 1.222222222 | 0.699471799 | 1 | 1 |
| UEVLD | 1.222222222 | 0.699471799 | 1 | 1 |
| UFL1 | 1.222222222 | 0.699471799 | 1 | 1 |
| GPR150 | 1.222222222 | 0.699471799 | 1 | 1 |
| EIF6 | 1.222222222 | 0.699471799 | 1 | 1 |
| LAMC1 | 1.222222222 | 0.699471799 | 1 | 1 |
| TTC21A | 1.222222222 | 0.699471799 | 1 | 1 |
| TTC39B | 1.222222222 | 0.699471799 | 1 | 1 |
| SEMA6B | 1.222222222 | 0.699471799 | 1 | 1 |
| C8G | 1.222222222 | 0.699471799 | 1 | 1 |
| PSMA3 | 1.222222222 | 0.699471799 | 1 | 1 |
| KRTAP4-2 | 1.222222222 | 0.699471799 | 1 | 1 |
| PPP6R3 | 1.222222222 | 0.699471799 | 1 | 1 |
| GPR135 | 1.222222222 | 0.699471799 | 1 | 1 |
| PSMB8 | 1.222222222 | 0.699471799 | 1 | 1 |
| SLC25A42 | 1.222222222 | 0.699471799 | 1 | 1 |
| GABRG2 | 1.222222222 | 0.699471799 | 1 | 1 |
| TBC1D2B | 1.222222222 | 0.699471799 | 1 | 1 |
| SLC25A45 | 1.222222222 | 0.699471799 | 1 | 1 |
| GPR176 | 1.222222222 | 0.699471799 | 1 | 1 |
| EIF2S1 | 1.222222222 | 0.699471799 | 1 | 1 |
| CCDC173 | 1.222222222 | 0.699471799 | 1 | 1 |
| SLC22A4 | 1.222222222 | 0.699471799 | 1 | 1 |
| LLCFC1 | 1.222222222 | 0.699471799 | 1 | 1 |
| GABRQ | 1.222222222 | 0.699471799 | 1 | 1 |
| CFAP45 | 1.222222222 | 0.699471799 | 1 | 1 |
| FTHL17 | 1.222222222 | 0.699471799 | 1 | 1 |
| PPP3CC | 1.222222222 | 0.699471799 | 1 | 1 |
| EIF1AD | 1.222222222 | 0.699471799 | 1 | 1 |
| EID3 | 1.222222222 | 0.699471799 | 1 | 1 |
| TSPYL6 | 1.222222222 | 0.699471799 | 1 | 1 |
| KRTAP4-5 | 1.222222222 | 0.699471799 | 1 | 1 |
| TSR3 | 1.222222222 | 0.699471799 | 1 | 1 |
| TEX47 | 1.222222222 | 0.699471799 | 1 | 1 |
| GRK6 | 1.222222222 | 0.699471799 | 1 | 1 |
| CFAP70 | 1.222222222 | 0.699471799 | 1 | 1 |
| PPWD1 | 1.222222222 | 0.699471799 | 1 | 1 |
| DERL3 | 1.222222222 | 0.699471799 | 1 | 1 |
| CCDC127 | 1.222222222 | 0.699471799 | 1 | 1 |
| GRM1 | 1.222222222 | 0.699471799 | 1 | 1 |
| SLC22A6 | 1.222222222 | 0.699471799 | 1 | 1 |
| KRTCAP2 | 1.222222222 | 0.699471799 | 1 | 1 |
| ELMO1 | 1.222222222 | 0.699471799 | 1 | 1 |
| KRTAP9-1 | 1.222222222 | 0.699471799 | 1 | 1 |
| STARD8 | 1.222222222 | 0.699471799 | 1 | 1 |
| LOC100505478 | 1.222222222 | 0.699471799 | 1 | 1 |
| TAPT1 | 1.222222222 | 0.699471799 | 1 | 1 |
| CCDC148 | 1.222222222 | 0.699471799 | 1 | 1 |
| UNC119B | 1.222222222 | 0.699471799 | 1 | 1 |
| CCDC15 | 1.222222222 | 0.699471799 | 1 | 1 |
| EIF2AK2 | 1.222222222 | 0.699471799 | 1 | 1 |
| TSSK1B | 1.222222222 | 0.699471799 | 1 | 1 |
| EIPR1 | 1.222222222 | 0.699471799 | 1 | 1 |
| LOC100506388 | 1.222222222 | 0.699471799 | 1 | 1 |
| PSG7 | 1.222222222 | 0.699471799 | 1 | 1 |
| SERPINB3 | 1.222222222 | 0.699471799 | 1 | 1 |
| KRTAP19-1 | 1.222222222 | 0.699471799 | 1 | 1 |
| FUZ | 1.222222222 | 0.699471799 | 1 | 1 |
| CACHD1 | 1.222222222 | 0.699471799 | 1 | 1 |
| LGALS7B | 1.222222222 | 0.699471799 | 1 | 1 |
| CADM3 | 1.222222222 | 0.699471799 | 1 | 1 |
| SEMA3D | 1.222222222 | 0.699471799 | 1 | 1 |
| PRKD1 | 1.222222222 | 0.699471799 | 1 | 1 |
| LGALS3BP | 1.222222222 | 0.699471799 | 1 | 1 |
| TWF2 | 1.222222222 | 0.699471799 | 1 | 1 |
| CAND1 | 1.222222222 | 0.699471799 | 1 | 1 |
| TUT1 | 1.222222222 | 0.699471799 | 1 | 1 |
| SLC25A19 | 1.222222222 | 0.699471799 | 1 | 1 |
| GRAMD4 | 1.222222222 | 0.699471799 | 1 | 1 |
| PRDX2 | 1.222222222 | 0.699471799 | 1 | 1 |
| FXYD3 | 1.222222222 | 0.699471799 | 1 | 1 |
| CACUL1 | 1.222222222 | 0.699471799 | 1 | 1 |
| PRMT9 | 1.222222222 | 0.699471799 | 1 | 1 |
| CAPN14 | 1.222222222 | 0.699471799 | 1 | 1 |
| SELENOV | 1.222222222 | 0.699471799 | 1 | 1 |
| PROC | 1.222222222 | 0.699471799 | 1 | 1 |
| CAPN7 | 1.222222222 | 0.699471799 | 1 | 1 |
| PROCA1 | 1.222222222 | 0.699471799 | 1 | 1 |
| CAPN9 | 1.222222222 | 0.699471799 | 1 | 1 |
| PRDX1 | 1.222222222 | 0.699471799 | 1 | 1 |
| LGALS8 | 1.222222222 | 0.699471799 | 1 | 1 |
| CADPS2 | 1.222222222 | 0.699471799 | 1 | 1 |
| GRAP2 | 1.222222222 | 0.699471799 | 1 | 1 |
| LHX2 | 1.222222222 | 0.699471799 | 1 | 1 |
| CALHM1 | 1.222222222 | 0.699471799 | 1 | 1 |
| PRICKLE2 | 1.222222222 | 0.699471799 | 1 | 1 |
| PRICKLE1 | 1.222222222 | 0.699471799 | 1 | 1 |
| PRKACB | 1.222222222 | 0.699471799 | 1 | 1 |
| CALCR | 1.222222222 | 0.699471799 | 1 | 1 |
| PRH1-PRR4 | 1.222222222 | 0.699471799 | 1 | 1 |
| CALM2 | 1.222222222 | 0.699471799 | 1 | 1 |
| PRKAR1A | 1.222222222 | 0.699471799 | 1 | 1 |
| CALML5 | 1.222222222 | 0.699471799 | 1 | 1 |
| CALML6 | 1.222222222 | 0.699471799 | 1 | 1 |
| FYN | 1.222222222 | 0.699471799 | 1 | 1 |
| CAMK2B | 1.222222222 | 0.699471799 | 1 | 1 |
| UAP1 | 1.222222222 | 0.699471799 | 1 | 1 |
| LIG4 | 1.222222222 | 0.699471799 | 1 | 1 |
| TBC1D10B | 1.222222222 | 0.699471799 | 1 | 1 |
| SERPINB10 | 1.222222222 | 0.699471799 | 1 | 1 |
| LGALS9B | 1.222222222 | 0.699471799 | 1 | 1 |
| SEMA3E | 1.222222222 | 0.699471799 | 1 | 1 |
| PRKCH | 1.222222222 | 0.699471799 | 1 | 1 |
| CALB1 | 1.222222222 | 0.699471799 | 1 | 1 |
| LEF1 | 1.222222222 | 0.699471799 | 1 | 1 |
| TTC39C | 1.222222222 | 0.699471799 | 1 | 1 |
| TUBGCP5 | 1.222222222 | 0.699471799 | 1 | 1 |
| CAPS2 | 1.222222222 | 0.699471799 | 1 | 1 |
| LCE2A | 1.222222222 | 0.699471799 | 1 | 1 |
| CASP8AP2 | 1.222222222 | 0.699471799 | 1 | 1 |
| CASP9 | 1.222222222 | 0.699471799 | 1 | 1 |
| LCE1B | 1.222222222 | 0.699471799 | 1 | 1 |
| GRAMD1A | 1.222222222 | 0.699471799 | 1 | 1 |
| CASQ2 | 1.222222222 | 0.699471799 | 1 | 1 |
| LCAT | 1.222222222 | 0.699471799 | 1 | 1 |
| PRR23A | 1.222222222 | 0.699471799 | 1 | 1 |
| SLC24A2 | 1.222222222 | 0.699471799 | 1 | 1 |
| LINGO1 | 1.222222222 | 0.699471799 | 1 | 1 |
| SLC25A2 | 1.222222222 | 0.699471799 | 1 | 1 |
| UBQLN2 | 1.222222222 | 0.699471799 | 1 | 1 |
| DLGAP5 | 1.222222222 | 0.699471799 | 1 | 1 |
| G6PC3 | 1.222222222 | 0.699471799 | 1 | 1 |
| EIF4B | 1.222222222 | 0.699471799 | 1 | 1 |
| DGKE | 1.222222222 | 0.699471799 | 1 | 1 |
| ST8SIA5 | 1.222222222 | 0.699471799 | 1 | 1 |
| LATS1 | 1.222222222 | 0.699471799 | 1 | 1 |
| PRC1 | 1.222222222 | 0.699471799 | 1 | 1 |
| LIPN | 1.222222222 | 0.699471799 | 1 | 1 |
| UBE3B | 1.222222222 | 0.699471799 | 1 | 1 |
| CASP4 | 1.222222222 | 0.699471799 | 1 | 1 |
| CACNA2D3 | 1.222222222 | 0.699471799 | 1 | 1 |
| CASP10 | 1.222222222 | 0.699471799 | 1 | 1 |
| LDLRAD1 | 1.222222222 | 0.699471799 | 1 | 1 |
| FZD4 | 1.222222222 | 0.699471799 | 1 | 1 |
| CD164 | 1.222222222 | 0.699471799 | 1 | 1 |
| EIF4ENIF1 | 1.222222222 | 0.699471799 | 1 | 1 |
| LDHA | 1.222222222 | 0.699471799 | 1 | 1 |
| PROS1 | 1.222222222 | 0.699471799 | 1 | 1 |
| TUBA3E | 1.222222222 | 0.699471799 | 1 | 1 |
| CDKN2AIP | 1.222222222 | 0.699471799 | 1 | 1 |
| PROX2 | 1.222222222 | 0.699471799 | 1 | 1 |
| FZR1 | 1.222222222 | 0.699471799 | 1 | 1 |
| TTYH3 | 1.222222222 | 0.699471799 | 1 | 1 |
| PRPF39 | 1.222222222 | 0.699471799 | 1 | 1 |
| DGKH | 1.222222222 | 0.699471799 | 1 | 1 |
| FXYD2 | 1.222222222 | 0.699471799 | 1 | 1 |
| UBE2J2 | 1.222222222 | 0.699471799 | 1 | 1 |
| TTYH1 | 1.222222222 | 0.699471799 | 1 | 1 |
| G3BP2 | 1.222222222 | 0.699471799 | 1 | 1 |
| PRPF8 | 1.222222222 | 0.699471799 | 1 | 1 |
| CASK | 1.222222222 | 0.699471799 | 1 | 1 |
| LINC00452 | 1.222222222 | 0.699471799 | 1 | 1 |
| GRAMD1C | 1.222222222 | 0.699471799 | 1 | 1 |
| TUBE1 | 1.222222222 | 0.699471799 | 1 | 1 |
| C6orf163 | 1.222222222 | 0.699471799 | 1 | 1 |
| EIF2AK2 | 1.222222222 | 0.699471799 | 1 | 1 |
| CCDC93 | 1.222222222 | 0.699471799 | 1 | 1 |
| TRPM3 | 1.222222222 | 0.699471799 | 1 | 1 |
| PRNP | 1.222222222 | 0.699471799 | 1 | 1 |
| GPR37 | 1.222222222 | 0.699471799 | 1 | 1 |
| KRT27 | 1.222222222 | 0.699471799 | 1 | 1 |
| C5AR2 | 1.222222222 | 0.699471799 | 1 | 1 |
| CCNT2 | 1.222222222 | 0.699471799 | 1 | 1 |
| KRT26 | 1.222222222 | 0.699471799 | 1 | 1 |
| STAT6 | 1.222222222 | 0.699471799 | 1 | 1 |
| KLHL7 | 1.222222222 | 0.699471799 | 1 | 1 |
| C4orf47 | 1.222222222 | 0.699471799 | 1 | 1 |
| GALNT8 | 1.222222222 | 0.699471799 | 1 | 1 |
| KRT25 | 1.222222222 | 0.699471799 | 1 | 1 |
| KRT23 | 1.222222222 | 0.699471799 | 1 | 1 |
| PTPN6 | 1.222222222 | 0.699471799 | 1 | 1 |
| TRPC3 | 1.222222222 | 0.699471799 | 1 | 1 |
| CCDC91 | 1.222222222 | 0.699471799 | 1 | 1 |
| PUS1 | 1.222222222 | 0.699471799 | 1 | 1 |
| SRSF4 | 1.222222222 | 0.699471799 | 1 | 1 |
| C5orf22 | 1.222222222 | 0.699471799 | 1 | 1 |
| EFHD2 | 1.222222222 | 0.699471799 | 1 | 1 |
| PUS3 | 1.222222222 | 0.699471799 | 1 | 1 |
| PPP1R9A | 1.222222222 | 0.699471799 | 1 | 1 |
| PPP1R7 | 1.222222222 | 0.699471799 | 1 | 1 |
| FSD2 | 1.222222222 | 0.699471799 | 1 | 1 |
| LOC101927470 | 1.222222222 | 0.699471799 | 1 | 1 |
| GTF2IRD1 | 1.222222222 | 0.699471799 | 1 | 1 |
| PTK2 | 1.222222222 | 0.699471799 | 1 | 1 |
| GPR50 | 1.222222222 | 0.699471799 | 1 | 1 |
| CCDC77 | 1.222222222 | 0.699471799 | 1 | 1 |
| PTK2B | 1.222222222 | 0.699471799 | 1 | 1 |
| HASPIN | 1.222222222 | 0.699471799 | 1 | 1 |
| SSH1 | 1.222222222 | 0.699471799 | 1 | 1 |
| CCDC8 | 1.222222222 | 0.699471799 | 1 | 1 |
| SRRT | 1.222222222 | 0.699471799 | 1 | 1 |
| PTOV1 | 1.222222222 | 0.699471799 | 1 | 1 |
| DENND4A | 1.222222222 | 0.699471799 | 1 | 1 |
| PTPN11 | 1.222222222 | 0.699471799 | 1 | 1 |
| LOC101928229 | 1.222222222 | 0.699471799 | 1 | 1 |
| PTH2R | 1.222222222 | 0.699471799 | 1 | 1 |
| TSPAN10 | 1.222222222 | 0.699471799 | 1 | 1 |
| STAU1 | 1.222222222 | 0.699471799 | 1 | 1 |
| LOC101928638 | 1.222222222 | 0.699471799 | 1 | 1 |
| TBC1D9B | 1.222222222 | 0.699471799 | 1 | 1 |
| C3orf80 | 1.222222222 | 0.699471799 | 1 | 1 |
| SDR39U1 | 1.222222222 | 0.699471799 | 1 | 1 |
| EFR3B | 1.222222222 | 0.699471799 | 1 | 1 |
| GPR132 | 1.222222222 | 0.699471799 | 1 | 1 |
| SLC20A2 | 1.222222222 | 0.699471799 | 1 | 1 |
| MKRN2OS | 1.222222222 | 0.699471799 | 1 | 1 |
| USP32 | 1.222222222 | 0.699471799 | 1 | 1 |
| EFR3A | 1.222222222 | 0.699471799 | 1 | 1 |
| PQLC2L | 1.222222222 | 0.699471799 | 1 | 1 |
| SDHA | 1.222222222 | 0.699471799 | 1 | 1 |
| C3orf36 | 1.222222222 | 0.699471799 | 1 | 1 |
| LOC101928871 | 1.222222222 | 0.699471799 | 1 | 1 |
| DEFB115 | 1.222222222 | 0.699471799 | 1 | 1 |
| GPR39 | 1.222222222 | 0.699471799 | 1 | 1 |
| GTF2E1 | 1.222222222 | 0.699471799 | 1 | 1 |
| CCNI | 1.222222222 | 0.699471799 | 1 | 1 |
| SLC26A5 | 1.222222222 | 0.699471799 | 1 | 1 |
| PTRHD1 | 1.222222222 | 0.699471799 | 1 | 1 |
| LOC101928239 | 1.222222222 | 0.699471799 | 1 | 1 |
| SEC14L1 | 1.222222222 | 0.699471799 | 1 | 1 |
| CCKAR | 1.222222222 | 0.699471799 | 1 | 1 |
| TAS2R40 | 1.222222222 | 0.699471799 | 1 | 1 |
| TBC1D9 | 1.222222222 | 0.699471799 | 1 | 1 |
| GSTM5 | 1.222222222 | 0.699471799 | 1 | 1 |
| LOC101929253 | 1.222222222 | 0.699471799 | 1 | 1 |
| GALNT5 | 1.222222222 | 0.699471799 | 1 | 1 |
| SDAD1 | 1.222222222 | 0.699471799 | 1 | 1 |
| TRD-GTC1-1 | 1.222222222 | 0.699471799 | 1 | 1 |
| UROD | 1.222222222 | 0.699471799 | 1 | 1 |
| C3orf22 | 1.222222222 | 0.699471799 | 1 | 1 |
| DLL4 | 1.222222222 | 0.699471799 | 1 | 1 |
| SERPINA4 | 1.222222222 | 0.699471799 | 1 | 1 |
| EMC8 | 1.222222222 | 0.699471799 | 1 | 1 |
| SRSF8 | 1.222222222 | 0.699471799 | 1 | 1 |
| TRMT61B | 1.222222222 | 0.699471799 | 1 | 1 |
| CCDC96 | 1.222222222 | 0.699471799 | 1 | 1 |
| DIP2A | 1.222222222 | 0.699471799 | 1 | 1 |
| LOC101927581 | 1.222222222 | 0.699471799 | 1 | 1 |
| C2orf50 | 1.222222222 | 0.699471799 | 1 | 1 |
| KLHL18 | 1.222222222 | 0.699471799 | 1 | 1 |
| CCT4 | 1.222222222 | 0.699471799 | 1 | 1 |
| CCDC43 | 1.222222222 | 0.699471799 | 1 | 1 |
| GTF3C2 | 1.222222222 | 0.699471799 | 1 | 1 |
| GPR85 | 1.222222222 | 0.699471799 | 1 | 1 |
| KRTAP10-10 | 1.222222222 | 0.699471799 | 1 | 1 |
| ELOVL3 | 1.222222222 | 0.699471799 | 1 | 1 |
| SEC22C | 1.222222222 | 0.699471799 | 1 | 1 |
| FSTL3 | 1.222222222 | 0.699471799 | 1 | 1 |
| TRIM77 | 1.222222222 | 0.699471799 | 1 | 1 |
| TSG101 | 1.222222222 | 0.699471799 | 1 | 1 |
| KRT85 | 1.222222222 | 0.699471799 | 1 | 1 |
| DEPDC1B | 1.222222222 | 0.699471799 | 1 | 1 |
| CCDC59 | 1.222222222 | 0.699471799 | 1 | 1 |
| GRXCR2 | 1.222222222 | 0.699471799 | 1 | 1 |
| CCDC42 | 1.222222222 | 0.699471799 | 1 | 1 |
| PXK | 1.222222222 | 0.699471799 | 1 | 1 |
| DEPDC4 | 1.222222222 | 0.699471799 | 1 | 1 |
| TRIM62 | 1.222222222 | 0.699471799 | 1 | 1 |
| LOC100996586 | 1.222222222 | 0.699471799 | 1 | 1 |
| FRMD5 | 1.222222222 | 0.699471799 | 1 | 1 |
| PSPC1 | 1.222222222 | 0.699471799 | 1 | 1 |
| CFAP100 | 1.222222222 | 0.699471799 | 1 | 1 |
| SEC61B | 1.222222222 | 0.699471799 | 1 | 1 |
| KLHDC9 | 1.222222222 | 0.699471799 | 1 | 1 |
| GAGE10 | 1.222222222 | 0.699471799 | 1 | 1 |
| PPP2R5B | 1.222222222 | 0.699471799 | 1 | 1 |
| UPF1 | 1.222222222 | 0.699471799 | 1 | 1 |
| CCZ1 | 1.222222222 | 0.699471799 | 1 | 1 |
| PPP2R5A | 1.222222222 | 0.699471799 | 1 | 1 |
| SLC19A2 | 1.222222222 | 0.699471799 | 1 | 1 |
| PTBP3 | 1.222222222 | 0.699471799 | 1 | 1 |
| KRTAP10-5 | 1.222222222 | 0.699471799 | 1 | 1 |
| TASP1 | 1.222222222 | 0.699471799 | 1 | 1 |
| TSEN34 | 1.222222222 | 0.699471799 | 1 | 1 |
| DIABLO | 1.222222222 | 0.699471799 | 1 | 1 |
| PPID | 1.222222222 | 0.699471799 | 1 | 1 |
| SSNA1 | 1.222222222 | 0.699471799 | 1 | 1 |
| KRT75 | 1.222222222 | 0.699471799 | 1 | 1 |
| C2orf81 | 1.222222222 | 0.699471799 | 1 | 1 |
| TBL1XR1 | 1.222222222 | 0.699471799 | 1 | 1 |
| DEPDC1 | 1.222222222 | 0.699471799 | 1 | 1 |
| GAGE2B | 1.222222222 | 0.699471799 | 1 | 1 |
| TRUB2 | 1.222222222 | 0.699471799 | 1 | 1 |
| PPP1R9B | 1.222222222 | 0.699471799 | 1 | 1 |
| KRT72 | 1.222222222 | 0.699471799 | 1 | 1 |
| PTGES2 | 1.222222222 | 0.699471799 | 1 | 1 |
| KRT78 | 1.222222222 | 0.699471799 | 1 | 1 |
| KLHL28 | 1.222222222 | 0.699471799 | 1 | 1 |
| DENND4B | 1.222222222 | 0.699471799 | 1 | 1 |
| CCDC62 | 1.222222222 | 0.699471799 | 1 | 1 |
| AKAP9 | 0.86270872 | 0.703689103 | 5 | 7 |
| ALMS1 | 0.86270872 | 0.703689103 | 5 | 7 |
| IGSF10 | 0.86270872 | 0.703689103 | 5 | 7 |
| LAMA4 | 0.86270872 | 0.703689103 | 5 | 7 |
| NAV2 | 0.86270872 | 0.703689103 | 5 | 7 |
| POLQ | 0.86270872 | 0.703689103 | 5 | 7 |
| CFAP46 | 0.86270872 | 0.703689103 | 5 | 7 |
| HLA-B | 0.890740741 | 0.704049818 | 37 | 48 |
| GOLGA8J | 0.882882883 | 0.71018558 | 28 | 37 |
| OR11H1 | 0.879739978 | 0.711950208 | 56 | 71 |
| RYR3 | 0.84 | 0.721421054 | 7 | 10 |
| TCHH | 0.84 | 0.721421054 | 7 | 10 |
| KIR2DL3 | 0.84 | 0.721421054 | 7 | 10 |
| NPIPB4 | 0.8747011 | 0.722409385 | 31 | 41 |
| HMCN2 | 0.859504132 | 0.72280737 | 16 | 22 |
| HLA-A | 0.867687261 | 0.724058618 | 23 | 31 |
| AS3MT | 0.858363858 | 0.729258512 | 19 | 26 |
| FOXK2 | 0.825079031 | 0.739109102 | 9 | 13 |
| ENTPD1 | 0.803418803 | 0.741459644 | 4 | 6 |
| PTCHD3 | 0.803418803 | 0.741459644 | 4 | 6 |
| NPIPB6 | 0.803418803 | 0.741459644 | 4 | 6 |
| CYP21A2 | 0.803418803 | 0.741459644 | 4 | 6 |
| ATAD3B | 0.803418803 | 0.741459644 | 4 | 6 |
| KCTD1 | 0.803418803 | 0.741459644 | 4 | 6 |
| COL20A1 | 0.803418803 | 0.741459644 | 4 | 6 |
| FRYL | 0.803418803 | 0.741459644 | 4 | 6 |
| LRP1 | 0.803418803 | 0.741459644 | 4 | 6 |
| ARSA | 0.803418803 | 0.741459644 | 4 | 6 |
| LOC100996335 | 0.803418803 | 0.741459644 | 4 | 6 |
| LOC101929522 | 0.803418803 | 0.741459644 | 4 | 6 |
| PRDM9 | 0.803418803 | 0.741459644 | 4 | 6 |
| TNKS1BP1 | 0.803418803 | 0.741459644 | 4 | 6 |
| MYOM3 | 0.803418803 | 0.741459644 | 4 | 6 |
| KIAA1755 | 0.803418803 | 0.741459644 | 4 | 6 |
| MEGF6 | 0.803418803 | 0.741459644 | 4 | 6 |
| SNX8 | 0.808333333 | 0.747949235 | 2 | 3 |
| FYCO1 | 0.808333333 | 0.747949235 | 2 | 3 |
| NLRP12 | 0.808333333 | 0.747949235 | 2 | 3 |
| RUFY4 | 0.808333333 | 0.747949235 | 2 | 3 |
| EML2 | 0.808333333 | 0.747949235 | 2 | 3 |
| PAMR1 | 0.808333333 | 0.747949235 | 2 | 3 |
| EML3 | 0.808333333 | 0.747949235 | 2 | 3 |
| LOC400682 | 0.808333333 | 0.747949235 | 2 | 3 |
| OR52M1 | 0.808333333 | 0.747949235 | 2 | 3 |
| CALD1 | 0.808333333 | 0.747949235 | 2 | 3 |
| DNAJC21 | 0.808333333 | 0.747949235 | 2 | 3 |
| FOXA1 | 0.808333333 | 0.747949235 | 2 | 3 |
| IGSF9B | 0.808333333 | 0.747949235 | 2 | 3 |
| SNX18 | 0.808333333 | 0.747949235 | 2 | 3 |
| NOL8 | 0.808333333 | 0.747949235 | 2 | 3 |
| C3orf20 | 0.808333333 | 0.747949235 | 2 | 3 |
| ANO9 | 0.808333333 | 0.747949235 | 2 | 3 |
| HMOX2 | 0.808333333 | 0.747949235 | 2 | 3 |
| SMG5 | 0.808333333 | 0.747949235 | 2 | 3 |
| PNPLA6 | 0.808333333 | 0.747949235 | 2 | 3 |
| EPB41L4A | 0.808333333 | 0.747949235 | 2 | 3 |
| IGSF5 | 0.808333333 | 0.747949235 | 2 | 3 |
| SHANK2 | 0.808333333 | 0.747949235 | 2 | 3 |
| OPRM1 | 0.808333333 | 0.747949235 | 2 | 3 |
| CYP51A1 | 0.808333333 | 0.747949235 | 2 | 3 |
| NOL6 | 0.808333333 | 0.747949235 | 2 | 3 |
| FRRS1 | 0.808333333 | 0.747949235 | 2 | 3 |
| SGIP1 | 0.808333333 | 0.747949235 | 2 | 3 |
| PPM1M | 0.808333333 | 0.747949235 | 2 | 3 |
| ALOXE3 | 0.808333333 | 0.747949235 | 2 | 3 |
| NEDD9 | 0.808333333 | 0.747949235 | 2 | 3 |
| LRRC15 | 0.808333333 | 0.747949235 | 2 | 3 |
| ZNF737 | 0.808333333 | 0.747949235 | 2 | 3 |
| CPT1C | 0.808333333 | 0.747949235 | 2 | 3 |
| IDUA | 0.808333333 | 0.747949235 | 2 | 3 |
| ALPPL2 | 0.808333333 | 0.747949235 | 2 | 3 |
| COQ8B | 0.808333333 | 0.747949235 | 2 | 3 |
| SYNE3 | 0.808333333 | 0.747949235 | 2 | 3 |
| CYP4A11 | 0.808333333 | 0.747949235 | 2 | 3 |
| CBARP | 0.808333333 | 0.747949235 | 2 | 3 |
| INAVA | 0.808333333 | 0.747949235 | 2 | 3 |
| CPED1 | 0.808333333 | 0.747949235 | 2 | 3 |
| AMPD1 | 0.808333333 | 0.747949235 | 2 | 3 |
| RORA | 0.808333333 | 0.747949235 | 2 | 3 |
| GPRC6A | 0.808333333 | 0.747949235 | 2 | 3 |
| C1RL | 0.808333333 | 0.747949235 | 2 | 3 |
| SLC2A4RG | 0.808333333 | 0.747949235 | 2 | 3 |
| PARD3 | 0.808333333 | 0.747949235 | 2 | 3 |
| HIST1H1D | 0.808333333 | 0.747949235 | 2 | 3 |
| TOGARAM1 | 0.808333333 | 0.747949235 | 2 | 3 |
| GNA14 | 0.808333333 | 0.747949235 | 2 | 3 |
| SGSM3 | 0.808333333 | 0.747949235 | 2 | 3 |
| UNC45A | 0.808333333 | 0.747949235 | 2 | 3 |
| CRIP1 | 0.808333333 | 0.747949235 | 2 | 3 |
| FAM209B | 0.808333333 | 0.747949235 | 2 | 3 |
| LPP | 0.808333333 | 0.747949235 | 2 | 3 |
| FOXO3 | 0.808333333 | 0.747949235 | 2 | 3 |
| FANCG | 0.808333333 | 0.747949235 | 2 | 3 |
| UNC13B | 0.808333333 | 0.747949235 | 2 | 3 |
| C19orf57 | 0.808333333 | 0.747949235 | 2 | 3 |
| LOC101060181 | 0.808333333 | 0.747949235 | 2 | 3 |
| FSTL4 | 0.808333333 | 0.747949235 | 2 | 3 |
| LRPAP1 | 0.808333333 | 0.747949235 | 2 | 3 |
| CREB3L3 | 0.808333333 | 0.747949235 | 2 | 3 |
| POSTN | 0.808333333 | 0.747949235 | 2 | 3 |
| TAP2 | 0.808333333 | 0.747949235 | 2 | 3 |
| POLG | 0.808333333 | 0.747949235 | 2 | 3 |
| UHRF1 | 0.808333333 | 0.747949235 | 2 | 3 |
| CTNNA3 | 0.808333333 | 0.747949235 | 2 | 3 |
| EP300 | 0.808333333 | 0.747949235 | 2 | 3 |
| SALL3 | 0.808333333 | 0.747949235 | 2 | 3 |
| CACNA1A | 0.808333333 | 0.747949235 | 2 | 3 |
| USP43 | 0.808333333 | 0.747949235 | 2 | 3 |
| NKD2 | 0.808333333 | 0.747949235 | 2 | 3 |
| RNF169 | 0.808333333 | 0.747949235 | 2 | 3 |
| ZNF587B | 0.808333333 | 0.747949235 | 2 | 3 |
| SLC26A1 | 0.808333333 | 0.747949235 | 2 | 3 |
| FPGT-TNNI3K | 0.808333333 | 0.747949235 | 2 | 3 |
| RRS1 | 0.808333333 | 0.747949235 | 2 | 3 |
| COL12A1 | 0.808333333 | 0.747949235 | 2 | 3 |
| ZNF366 | 0.808333333 | 0.747949235 | 2 | 3 |
| SAGE1 | 0.808333333 | 0.747949235 | 2 | 3 |
| FRS3 | 0.808333333 | 0.747949235 | 2 | 3 |
| CNTNAP5 | 0.808333333 | 0.747949235 | 2 | 3 |
| CACNA1S | 0.808333333 | 0.747949235 | 2 | 3 |
| ZNF780B | 0.808333333 | 0.747949235 | 2 | 3 |
| DHX30 | 0.808333333 | 0.747949235 | 2 | 3 |
| RNF34 | 0.808333333 | 0.747949235 | 2 | 3 |
| LOC100131094 | 0.808333333 | 0.747949235 | 2 | 3 |
| SMARCA2 | 0.808333333 | 0.747949235 | 2 | 3 |
| DPP7 | 0.808333333 | 0.747949235 | 2 | 3 |
| SORCS1 | 0.808333333 | 0.747949235 | 2 | 3 |
| PRAMEF2 | 0.808333333 | 0.747949235 | 2 | 3 |
| DOCK7 | 0.808333333 | 0.747949235 | 2 | 3 |
| C9orf66 | 0.808333333 | 0.747949235 | 2 | 3 |
| PRAMEF7 | 0.808333333 | 0.747949235 | 2 | 3 |
| SYDE2 | 0.808333333 | 0.747949235 | 2 | 3 |
| AKAP3 | 0.808333333 | 0.747949235 | 2 | 3 |
| DHX16 | 0.808333333 | 0.747949235 | 2 | 3 |
| VIT | 0.808333333 | 0.747949235 | 2 | 3 |
| NID1 | 0.808333333 | 0.747949235 | 2 | 3 |
| ERICH3 | 0.808333333 | 0.747949235 | 2 | 3 |
| GNPTAB | 0.808333333 | 0.747949235 | 2 | 3 |
| SH3BP2 | 0.808333333 | 0.747949235 | 2 | 3 |
| NCL | 0.808333333 | 0.747949235 | 2 | 3 |
| PARD3B | 0.808333333 | 0.747949235 | 2 | 3 |
| FAM200B | 0.808333333 | 0.747949235 | 2 | 3 |
| VRTN | 0.808333333 | 0.747949235 | 2 | 3 |
| MAP3K19 | 0.808333333 | 0.747949235 | 2 | 3 |
| FHOD3 | 0.808333333 | 0.747949235 | 2 | 3 |
| TMPRSS6 | 0.808333333 | 0.747949235 | 2 | 3 |
| MAP3K5 | 0.808333333 | 0.747949235 | 2 | 3 |
| WWOX | 0.808333333 | 0.747949235 | 2 | 3 |
| NUTM2A | 0.808333333 | 0.747949235 | 2 | 3 |
| ARHGEF18 | 0.808333333 | 0.747949235 | 2 | 3 |
| ACTBL2 | 0.808333333 | 0.747949235 | 2 | 3 |
| ADGRD2 | 0.808333333 | 0.747949235 | 2 | 3 |
| BRD3 | 0.808333333 | 0.747949235 | 2 | 3 |
| PLCG1 | 0.808333333 | 0.747949235 | 2 | 3 |
| PTPRG | 0.808333333 | 0.747949235 | 2 | 3 |
| TBC1D22A | 0.808333333 | 0.747949235 | 2 | 3 |
| PRSS56 | 0.808333333 | 0.747949235 | 2 | 3 |
| CDK5RAP3 | 0.808333333 | 0.747949235 | 2 | 3 |
| TRAPPC8 | 0.808333333 | 0.747949235 | 2 | 3 |
| PEX6 | 0.808333333 | 0.747949235 | 2 | 3 |
| GALC | 0.808333333 | 0.747949235 | 2 | 3 |
| ATL2 | 0.808333333 | 0.747949235 | 2 | 3 |
| NRP2 | 0.808333333 | 0.747949235 | 2 | 3 |
| WDR36 | 0.808333333 | 0.747949235 | 2 | 3 |
| ITGA11 | 0.808333333 | 0.747949235 | 2 | 3 |
| MTMR7 | 0.808333333 | 0.747949235 | 2 | 3 |
| MTMR3 | 0.808333333 | 0.747949235 | 2 | 3 |
| ITGA3 | 0.808333333 | 0.747949235 | 2 | 3 |
| KIAA1549L | 0.808333333 | 0.747949235 | 2 | 3 |
| MAP2K3 | 0.808333333 | 0.747949235 | 2 | 3 |
| ZBTB38 | 0.808333333 | 0.747949235 | 2 | 3 |
| SIN3B | 0.808333333 | 0.747949235 | 2 | 3 |
| NSD3 | 0.808333333 | 0.747949235 | 2 | 3 |
| ECT2L | 0.808333333 | 0.747949235 | 2 | 3 |
| DMWD | 0.808333333 | 0.747949235 | 2 | 3 |
| CCDC103 | 0.808333333 | 0.747949235 | 2 | 3 |
| TRMU | 0.808333333 | 0.747949235 | 2 | 3 |
| PLCXD1 | 0.808333333 | 0.747949235 | 2 | 3 |
| MLLT6 | 0.808333333 | 0.747949235 | 2 | 3 |
| ATP2B4 | 0.808333333 | 0.747949235 | 2 | 3 |
| HDAC10 | 0.808333333 | 0.747949235 | 2 | 3 |
| CFAP221 | 0.808333333 | 0.747949235 | 2 | 3 |
| MTOR | 0.808333333 | 0.747949235 | 2 | 3 |
| SCARF1 | 0.808333333 | 0.747949235 | 2 | 3 |
| ACSM5 | 0.808333333 | 0.747949235 | 2 | 3 |
| TNRC6C | 0.808333333 | 0.747949235 | 2 | 3 |
| TSEN54 | 0.808333333 | 0.747949235 | 2 | 3 |
| DYTN | 0.808333333 | 0.747949235 | 2 | 3 |
| BMP1 | 0.808333333 | 0.747949235 | 2 | 3 |
| SLC22A25 | 0.808333333 | 0.747949235 | 2 | 3 |
| WFIKKN2 | 0.808333333 | 0.747949235 | 2 | 3 |
| EXOC7 | 0.808333333 | 0.747949235 | 2 | 3 |
| DYRK4 | 0.808333333 | 0.747949235 | 2 | 3 |
| MBD4 | 0.808333333 | 0.747949235 | 2 | 3 |
| ASB18 | 0.808333333 | 0.747949235 | 2 | 3 |
| OR2T29 | 0.808333333 | 0.747949235 | 2 | 3 |
| BIVM-ERCC5 | 0.808333333 | 0.747949235 | 2 | 3 |
| WNK1 | 0.808333333 | 0.747949235 | 2 | 3 |
| NT5DC2 | 0.808333333 | 0.747949235 | 2 | 3 |
| PKP1 | 0.808333333 | 0.747949235 | 2 | 3 |
| KRTAP16-1 | 0.808333333 | 0.747949235 | 2 | 3 |
| NT5E | 0.808333333 | 0.747949235 | 2 | 3 |
| SCN11A | 0.808333333 | 0.747949235 | 2 | 3 |
| PEX2 | 0.808333333 | 0.747949235 | 2 | 3 |
| BMP2K | 0.808333333 | 0.747949235 | 2 | 3 |
| PITPNM1 | 0.808333333 | 0.747949235 | 2 | 3 |
| TNFRSF6B | 0.808333333 | 0.747949235 | 2 | 3 |
| GAK | 0.808333333 | 0.747949235 | 2 | 3 |
| PDCD11 | 0.808333333 | 0.747949235 | 2 | 3 |
| EXOC3L1 | 0.808333333 | 0.747949235 | 2 | 3 |
| WRB | 0.808333333 | 0.747949235 | 2 | 3 |
| ZFYVE26 | 0.808333333 | 0.747949235 | 2 | 3 |
| JPH3 | 0.808333333 | 0.747949235 | 2 | 3 |
| SLC6A5 | 0.808333333 | 0.747949235 | 2 | 3 |
| GRM4 | 0.808333333 | 0.747949235 | 2 | 3 |
| SLC10A5 | 0.808333333 | 0.747949235 | 2 | 3 |
| BCL9 | 0.808333333 | 0.747949235 | 2 | 3 |
| PSG2 | 0.808333333 | 0.747949235 | 2 | 3 |
| RBM5 | 0.808333333 | 0.747949235 | 2 | 3 |
| PDE3B | 0.808333333 | 0.747949235 | 2 | 3 |
| DYNC1H1 | 0.808333333 | 0.747949235 | 2 | 3 |
| TRABD2A | 0.808333333 | 0.747949235 | 2 | 3 |
| SEC16B | 0.808333333 | 0.747949235 | 2 | 3 |
| ZDHHC11 | 0.808333333 | 0.747949235 | 2 | 3 |
| ZNF132 | 0.808333333 | 0.747949235 | 2 | 3 |
| ABCC12 | 0.808333333 | 0.747949235 | 2 | 3 |
| ABLIM2 | 0.808333333 | 0.747949235 | 2 | 3 |
| ETAA1 | 0.808333333 | 0.747949235 | 2 | 3 |
| ATXN7L1 | 0.808333333 | 0.747949235 | 2 | 3 |
| REV3L | 0.808333333 | 0.747949235 | 2 | 3 |
| SLC35E2B | 0.808333333 | 0.747949235 | 2 | 3 |
| ZNFX1 | 0.808333333 | 0.747949235 | 2 | 3 |
| PCDHA4 | 0.808333333 | 0.747949235 | 2 | 3 |
| MYH11 | 0.808333333 | 0.747949235 | 2 | 3 |
| NUP210 | 0.808333333 | 0.747949235 | 2 | 3 |
| NUMA1 | 0.808333333 | 0.747949235 | 2 | 3 |
| IL2RA | 0.808333333 | 0.747949235 | 2 | 3 |
| IPO4 | 0.808333333 | 0.747949235 | 2 | 3 |
| NEMP2 | 0.808333333 | 0.747949235 | 2 | 3 |
| PLXNC1 | 0.808333333 | 0.747949235 | 2 | 3 |
| PCDHA7 | 0.808333333 | 0.747949235 | 2 | 3 |
| CD101 | 0.808333333 | 0.747949235 | 2 | 3 |
| APIP | 0.808333333 | 0.747949235 | 2 | 3 |
| TRIM24 | 0.808333333 | 0.747949235 | 2 | 3 |
| PCDH11X | 0.808333333 | 0.747949235 | 2 | 3 |
| MYO1D | 0.808333333 | 0.747949235 | 2 | 3 |
| EFCC1 | 0.808333333 | 0.747949235 | 2 | 3 |
| P3H2 | 0.808333333 | 0.747949235 | 2 | 3 |
| GALNTL5 | 0.808333333 | 0.747949235 | 2 | 3 |
| TACC3 | 0.808333333 | 0.747949235 | 2 | 3 |
| TEAD4 | 0.808333333 | 0.747949235 | 2 | 3 |
| ZNF845 | 0.808333333 | 0.747949235 | 2 | 3 |
| P3H1 | 0.808333333 | 0.747949235 | 2 | 3 |
| DGKZ | 0.808333333 | 0.747949235 | 2 | 3 |
| R3HDM1 | 0.808333333 | 0.747949235 | 2 | 3 |
| KLHL40 | 0.808333333 | 0.747949235 | 2 | 3 |
| FAM50B | 0.808333333 | 0.747949235 | 2 | 3 |
| METTL25 | 0.808333333 | 0.747949235 | 2 | 3 |
| TBC1D12 | 0.808333333 | 0.747949235 | 2 | 3 |
| TRIP11 | 0.808333333 | 0.747949235 | 2 | 3 |
| SLC39A13 | 0.808333333 | 0.747949235 | 2 | 3 |
| SCAF8 | 0.808333333 | 0.747949235 | 2 | 3 |
| RIMS1 | 0.808333333 | 0.747949235 | 2 | 3 |
| WASHC2A | 0.808333333 | 0.747949235 | 2 | 3 |
| LARS | 0.808333333 | 0.747949235 | 2 | 3 |
| SLC19A1 | 0.808333333 | 0.747949235 | 2 | 3 |
| ATP6V1H | 0.808333333 | 0.747949235 | 2 | 3 |
| ABCB11 | 0.808333333 | 0.747949235 | 2 | 3 |
| IRS2 | 0.808333333 | 0.747949235 | 2 | 3 |
| CKAP5 | 0.808333333 | 0.747949235 | 2 | 3 |
| CNKSR1 | 0.808333333 | 0.747949235 | 2 | 3 |
| GPR108 | 0.808333333 | 0.747949235 | 2 | 3 |
| VSIG10L | 0.808333333 | 0.747949235 | 2 | 3 |
| PCDHAC1 | 0.808333333 | 0.747949235 | 2 | 3 |
| HDGFRP2 | 0.808333333 | 0.747949235 | 2 | 3 |
| AOAH | 0.808333333 | 0.747949235 | 2 | 3 |
| SCNN1D | 0.808333333 | 0.747949235 | 2 | 3 |
| CNGA4 | 0.808333333 | 0.747949235 | 2 | 3 |
| TTC37 | 0.808333333 | 0.747949235 | 2 | 3 |
| ETS2 | 0.808333333 | 0.747949235 | 2 | 3 |
| TRGC2 | 0.808333333 | 0.747949235 | 2 | 3 |
| RTL9 | 0.808333333 | 0.747949235 | 2 | 3 |
| CASP7 | 0.808333333 | 0.747949235 | 2 | 3 |
| NEXMIF | 0.808333333 | 0.747949235 | 2 | 3 |
| GJB5 | 0.808333333 | 0.747949235 | 2 | 3 |
| ZNF135 | 0.808333333 | 0.747949235 | 2 | 3 |
| KLHDC4 | 0.808333333 | 0.747949235 | 2 | 3 |
| KIF13A | 0.808333333 | 0.747949235 | 2 | 3 |
| SERPINA2 | 0.808333333 | 0.747949235 | 2 | 3 |
| PLEKHG4 | 0.808333333 | 0.747949235 | 2 | 3 |
| CLDN16 | 0.808333333 | 0.747949235 | 2 | 3 |
| TTN | 0.855782313 | 0.748655017 | 37 | 49 |
| PRRC2B | 0.798245614 | 0.750264513 | 6 | 9 |
| MUC22 | 0.798245614 | 0.750264513 | 6 | 9 |
| CSMD3 | 0.798245614 | 0.750264513 | 6 | 9 |
| EYS | 0.798245614 | 0.750264513 | 6 | 9 |
| NBPF11 | 0.798245614 | 0.750264513 | 6 | 9 |
| SRRM2 | 0.798245614 | 0.750264513 | 6 | 9 |
| IGHJ6 | 0.804347826 | 0.752226994 | 74 | 92 |
| FLG | 0.848372093 | 0.755789128 | 32 | 43 |
| LRP8 | 0.813380282 | 0.756245745 | 11 | 16 |
| MUC19 | 0.802197802 | 0.7567043 | 73 | 91 |
| ZDHHC11B | 0.828205128 | 0.758428705 | 17 | 24 |
| LOC101927859 | 0.816326531 | 0.760200169 | 80 | 98 |
| CCL3L3 | 0.845167201 | 0.761078781 | 45 | 59 |
| USH2A | 0.792792793 | 0.762785007 | 8 | 12 |
| ZNF469 | 0.792792793 | 0.762785007 | 8 | 12 |
| SPATA31C2 | 0.787037037 | 0.776412427 | 10 | 15 |
| ALOX5AP | 0.825396825 | 0.777628351 | 26 | 36 |
| KIAA0040 | 0.816129032 | 0.781997765 | 22 | 31 |
| ADGRV1 | 0.746753247 | 0.78213425 | 5 | 8 |
| RAD54L | 0.746753247 | 0.78213425 | 5 | 8 |
| SYNE2 | 0.746753247 | 0.78213425 | 5 | 8 |
| OVOS | 0.746753247 | 0.78213425 | 5 | 8 |
| ABCC6 | 0.746753247 | 0.78213425 | 5 | 8 |
| LRRK1 | 0.746753247 | 0.78213425 | 5 | 8 |
| SACS | 0.746753247 | 0.78213425 | 5 | 8 |
| USP17L1 | 0.721518987 | 0.786353447 | 3 | 5 |
| SRL | 0.721518987 | 0.786353447 | 3 | 5 |
| ATP8B3 | 0.721518987 | 0.786353447 | 3 | 5 |
| ATPAF1 | 0.721518987 | 0.786353447 | 3 | 5 |
| PTPRZ1 | 0.721518987 | 0.786353447 | 3 | 5 |
| CFAP61 | 0.721518987 | 0.786353447 | 3 | 5 |
| CCHCR1 | 0.721518987 | 0.786353447 | 3 | 5 |
| PTPRF | 0.721518987 | 0.786353447 | 3 | 5 |
| PPL | 0.721518987 | 0.786353447 | 3 | 5 |
| ESPN | 0.721518987 | 0.786353447 | 3 | 5 |
| NID2 | 0.721518987 | 0.786353447 | 3 | 5 |
| PKD1L1 | 0.721518987 | 0.786353447 | 3 | 5 |
| ATM | 0.721518987 | 0.786353447 | 3 | 5 |
| USP17L10 | 0.721518987 | 0.786353447 | 3 | 5 |
| BCL9L | 0.721518987 | 0.786353447 | 3 | 5 |
| ATAD3A | 0.721518987 | 0.786353447 | 3 | 5 |
| XRCC1 | 0.721518987 | 0.786353447 | 3 | 5 |
| SORCS2 | 0.721518987 | 0.786353447 | 3 | 5 |
| NIN | 0.721518987 | 0.786353447 | 3 | 5 |
| NUTM2F | 0.721518987 | 0.786353447 | 3 | 5 |
| ARMC5 | 0.721518987 | 0.786353447 | 3 | 5 |
| SVOPL | 0.721518987 | 0.786353447 | 3 | 5 |
| APBA1 | 0.721518987 | 0.786353447 | 3 | 5 |
| ADAMTS18 | 0.721518987 | 0.786353447 | 3 | 5 |
| MADD | 0.721518987 | 0.786353447 | 3 | 5 |
| TLN1 | 0.721518987 | 0.786353447 | 3 | 5 |
| JARID2 | 0.721518987 | 0.786353447 | 3 | 5 |
| NCOA6 | 0.721518987 | 0.786353447 | 3 | 5 |
| DYNLL1 | 0.721518987 | 0.786353447 | 3 | 5 |
| WDR27 | 0.721518987 | 0.786353447 | 3 | 5 |
| RPGRIP1 | 0.721518987 | 0.786353447 | 3 | 5 |
| RLF | 0.721518987 | 0.786353447 | 3 | 5 |
| GPR179 | 0.721518987 | 0.786353447 | 3 | 5 |
| NCKAP5L | 0.721518987 | 0.786353447 | 3 | 5 |
| C14orf37 | 0.721518987 | 0.786353447 | 3 | 5 |
| CGN | 0.721518987 | 0.786353447 | 3 | 5 |
| ACTN2 | 0.721518987 | 0.786353447 | 3 | 5 |
| CILP | 0.721518987 | 0.786353447 | 3 | 5 |
| TET2 | 0.721518987 | 0.786353447 | 3 | 5 |
| ARHGEF15 | 0.721518987 | 0.786353447 | 3 | 5 |
| MAST4 | 0.721518987 | 0.786353447 | 3 | 5 |
| ARID1B | 0.721518987 | 0.786353447 | 3 | 5 |
| EPB41L1 | 0.721518987 | 0.786353447 | 3 | 5 |
| ITIH5 | 0.721518987 | 0.786353447 | 3 | 5 |
| DCST1 | 0.721518987 | 0.786353447 | 3 | 5 |
| FOXI3 | 0.721518987 | 0.786353447 | 3 | 5 |
| ZFHX2 | 0.721518987 | 0.786353447 | 3 | 5 |
| COL4A4 | 0.721518987 | 0.786353447 | 3 | 5 |
| MYO9A | 0.721518987 | 0.786353447 | 3 | 5 |
| PML | 0.721518987 | 0.786353447 | 3 | 5 |
| FBN1 | 0.721518987 | 0.786353447 | 3 | 5 |
| IFT172 | 0.721518987 | 0.786353447 | 3 | 5 |
| NPIPA5 | 0.823129252 | 0.786749574 | 33 | 45 |
| DNAH2 | 0.755151515 | 0.788011625 | 7 | 11 |
| NBPF15 | 0.800480769 | 0.790254404 | 18 | 26 |
| MAGEC1 | 0.757338552 | 0.797435177 | 9 | 14 |
| USP17L8 | 0.757338552 | 0.797435177 | 9 | 14 |
| AGAP4 | 0.757338552 | 0.797435177 | 9 | 14 |
| MST1P2 | 0.78313253 | 0.798013651 | 65 | 83 |
| FOXD4L5 | 0.818181818 | 0.798372898 | 81 | 99 |
| SNED1 | 0.805745554 | 0.805431417 | 31 | 43 |
| KMT2C | 0.756420878 | 0.808319408 | 11 | 17 |
| LOC100996350 | 0.756420878 | 0.808319408 | 11 | 17 |
| OR2T33 | 0.791474063 | 0.810788239 | 23 | 33 |
| SSC5D | 0.710526316 | 0.814967406 | 6 | 10 |
| BCLAF1 | 0.710526316 | 0.814967406 | 6 | 10 |
| AHSA2 | 0.710526316 | 0.814967406 | 6 | 10 |
| AR | 0.710526316 | 0.814967406 | 6 | 10 |
| NPIPB3 | 0.798758865 | 0.815264785 | 34 | 47 |
| NCOR2 | 0.681318681 | 0.817669232 | 4 | 7 |
| VPS13C | 0.681318681 | 0.817669232 | 4 | 7 |
| SFI1 | 0.681318681 | 0.817669232 | 4 | 7 |
| IGF2R | 0.681318681 | 0.817669232 | 4 | 7 |
| FLNC | 0.681318681 | 0.817669232 | 4 | 7 |
| DOCK4 | 0.681318681 | 0.817669232 | 4 | 7 |
| SPATA31D1 | 0.681318681 | 0.817669232 | 4 | 7 |
| WDFY4 | 0.681318681 | 0.817669232 | 4 | 7 |
| DYSF | 0.681318681 | 0.817669232 | 4 | 7 |
| TACC2 | 0.681318681 | 0.817669232 | 4 | 7 |
| PLXNB2 | 0.681318681 | 0.817669232 | 4 | 7 |
| KRTAP4-11 | 0.681318681 | 0.817669232 | 4 | 7 |
| OTOF | 0.681318681 | 0.817669232 | 4 | 7 |
| BAIAP3 | 0.681318681 | 0.817669232 | 4 | 7 |
| NOC4L | 0.681318681 | 0.817669232 | 4 | 7 |
| VWA5B1 | 0.681318681 | 0.817669232 | 4 | 7 |
| ADGRA1 | 0.681318681 | 0.817669232 | 4 | 7 |
| STK19 | 0.681318681 | 0.817669232 | 4 | 7 |
| MYO7B | 0.681318681 | 0.817669232 | 4 | 7 |
| POTEF | 0.681318681 | 0.817669232 | 4 | 7 |
| TRBV11-1 | 0.753623188 | 0.819814874 | 13 | 20 |
| USP17L18 | 0.787390704 | 0.823865132 | 29 | 41 |
| PIEZO1 | 0.729166667 | 0.826829131 | 10 | 16 |
| TBP | 0.70531401 | 0.827037684 | 73 | 92 |
| NBPF1 | 0.717171717 | 0.829136108 | 71 | 90 |
| PDE4DIP | 0.777777778 | 0.832983588 | 28 | 40 |
| LOC645359 | 0.730827068 | 0.835786606 | 12 | 19 |
| OR4N2 | 0.604938272 | 0.836379091 | 1 | 2 |
| ZNF844 | 0.604938272 | 0.836379091 | 1 | 2 |
| OR4C15 | 0.604938272 | 0.836379091 | 1 | 2 |
| SNAP91 | 0.604938272 | 0.836379091 | 1 | 2 |
| SCRN1 | 0.604938272 | 0.836379091 | 1 | 2 |
| SNAPC3 | 0.604938272 | 0.836379091 | 1 | 2 |
| OR52D1 | 0.604938272 | 0.836379091 | 1 | 2 |
| SBK1 | 0.604938272 | 0.836379091 | 1 | 2 |
| SNX14 | 0.604938272 | 0.836379091 | 1 | 2 |
| OR4A16 | 0.604938272 | 0.836379091 | 1 | 2 |
| SCRN2 | 0.604938272 | 0.836379091 | 1 | 2 |
| SEMA3C | 0.604938272 | 0.836379091 | 1 | 2 |
| SEMA4F | 0.604938272 | 0.836379091 | 1 | 2 |
| ZNF784 | 0.604938272 | 0.836379091 | 1 | 2 |
| OR4D6 | 0.604938272 | 0.836379091 | 1 | 2 |
| SERPINA11 | 0.604938272 | 0.836379091 | 1 | 2 |
| ZNF891 | 0.604938272 | 0.836379091 | 1 | 2 |
| ZNF98 | 0.604938272 | 0.836379091 | 1 | 2 |
| OR11G2 | 0.604938272 | 0.836379091 | 1 | 2 |
| OR10G3 | 0.604938272 | 0.836379091 | 1 | 2 |
| TBC1D17 | 0.604938272 | 0.836379091 | 1 | 2 |
| ZNF768 | 0.604938272 | 0.836379091 | 1 | 2 |
| ZNF732 | 0.604938272 | 0.836379091 | 1 | 2 |
| SEL1L2 | 0.604938272 | 0.836379091 | 1 | 2 |
| TBC1D5 | 0.604938272 | 0.836379091 | 1 | 2 |
| ZSCAN21 | 0.604938272 | 0.836379091 | 1 | 2 |
| SEZ6L2 | 0.604938272 | 0.836379091 | 1 | 2 |
| SETD5 | 0.604938272 | 0.836379091 | 1 | 2 |
| OR2T10 | 0.604938272 | 0.836379091 | 1 | 2 |
| OR2T34 | 0.604938272 | 0.836379091 | 1 | 2 |
| ZNF735 | 0.604938272 | 0.836379091 | 1 | 2 |
| SECISBP2L | 0.604938272 | 0.836379091 | 1 | 2 |
| ZNF74 | 0.604938272 | 0.836379091 | 1 | 2 |
| SAMD1 | 0.604938272 | 0.836379091 | 1 | 2 |
| ZSCAN1 | 0.604938272 | 0.836379091 | 1 | 2 |
| ZSCAN10 | 0.604938272 | 0.836379091 | 1 | 2 |
| SEC31A | 0.604938272 | 0.836379091 | 1 | 2 |
| SMG9 | 0.604938272 | 0.836379091 | 1 | 2 |
| SEPT10 | 0.604938272 | 0.836379091 | 1 | 2 |
| SEL1L3 | 0.604938272 | 0.836379091 | 1 | 2 |
| TERF2IP | 0.604938272 | 0.836379091 | 1 | 2 |
| SEMG2 | 0.604938272 | 0.836379091 | 1 | 2 |
| TCP10L2 | 0.604938272 | 0.836379091 | 1 | 2 |
| SEMA6A | 0.604938272 | 0.836379091 | 1 | 2 |
| ZNF728 | 0.604938272 | 0.836379091 | 1 | 2 |
| OR2F2 | 0.604938272 | 0.836379091 | 1 | 2 |
| TALDO1 | 0.604938272 | 0.836379091 | 1 | 2 |
| ZBED9 | 0.604938272 | 0.836379091 | 1 | 2 |
| SAMD3 | 0.604938272 | 0.836379091 | 1 | 2 |
| SCN9A | 0.604938272 | 0.836379091 | 1 | 2 |
| OR2A14 | 0.604938272 | 0.836379091 | 1 | 2 |
| ZSWIM6 | 0.604938272 | 0.836379091 | 1 | 2 |
| SERTAD4 | 0.604938272 | 0.836379091 | 1 | 2 |
| OR2V1 | 0.604938272 | 0.836379091 | 1 | 2 |
| OR51B6 | 0.604938272 | 0.836379091 | 1 | 2 |
| ZNF761 | 0.604938272 | 0.836379091 | 1 | 2 |
| ZNF730 | 0.604938272 | 0.836379091 | 1 | 2 |
| SELE | 0.604938272 | 0.836379091 | 1 | 2 |
| OR2L3 | 0.604938272 | 0.836379091 | 1 | 2 |
| TAS2R5 | 0.604938272 | 0.836379091 | 1 | 2 |
| ZSCAN31 | 0.604938272 | 0.836379091 | 1 | 2 |
| SALL1 | 0.604938272 | 0.836379091 | 1 | 2 |
| SMTN | 0.604938272 | 0.836379091 | 1 | 2 |
| SRRM3 | 0.604938272 | 0.836379091 | 1 | 2 |
| TFG | 0.604938272 | 0.836379091 | 1 | 2 |
| STEAP1B | 0.604938272 | 0.836379091 | 1 | 2 |
| TRMT44 | 0.604938272 | 0.836379091 | 1 | 2 |
| PTPRU | 0.604938272 | 0.836379091 | 1 | 2 |
| PTPRS | 0.604938272 | 0.836379091 | 1 | 2 |
| PIGT | 0.604938272 | 0.836379091 | 1 | 2 |
| SLC1A7 | 0.604938272 | 0.836379091 | 1 | 2 |
| SLC1A5 | 0.604938272 | 0.836379091 | 1 | 2 |
| PIGQ | 0.604938272 | 0.836379091 | 1 | 2 |
| PIGM | 0.604938272 | 0.836379091 | 1 | 2 |
| XPNPEP2 | 0.604938272 | 0.836379091 | 1 | 2 |
| PTX4 | 0.604938272 | 0.836379091 | 1 | 2 |
| PIFO | 0.604938272 | 0.836379091 | 1 | 2 |
| XPOT | 0.604938272 | 0.836379091 | 1 | 2 |
| XRCC2 | 0.604938272 | 0.836379091 | 1 | 2 |
| XRCC3 | 0.604938272 | 0.836379091 | 1 | 2 |
| PIK3CD | 0.604938272 | 0.836379091 | 1 | 2 |
| TRIM74 | 0.604938272 | 0.836379091 | 1 | 2 |
| XG | 0.604938272 | 0.836379091 | 1 | 2 |
| TRO | 0.604938272 | 0.836379091 | 1 | 2 |
| WNT1 | 0.604938272 | 0.836379091 | 1 | 2 |
| TSKS | 0.604938272 | 0.836379091 | 1 | 2 |
| TSGA10IP | 0.604938272 | 0.836379091 | 1 | 2 |
| TSFM | 0.604938272 | 0.836379091 | 1 | 2 |
| PITPNM2 | 0.604938272 | 0.836379091 | 1 | 2 |
| SLC37A3 | 0.604938272 | 0.836379091 | 1 | 2 |
| WNT5A | 0.604938272 | 0.836379091 | 1 | 2 |
| SPOCK2 | 0.604938272 | 0.836379091 | 1 | 2 |
| TRPS1 | 0.604938272 | 0.836379091 | 1 | 2 |
| WNT8B | 0.604938272 | 0.836379091 | 1 | 2 |
| PIP5KL1 | 0.604938272 | 0.836379091 | 1 | 2 |
| SLC37A4 | 0.604938272 | 0.836379091 | 1 | 2 |
| TRPM2 | 0.604938272 | 0.836379091 | 1 | 2 |
| PIM1 | 0.604938272 | 0.836379091 | 1 | 2 |
| PTPRH | 0.604938272 | 0.836379091 | 1 | 2 |
| PIK3R3 | 0.604938272 | 0.836379091 | 1 | 2 |
| PKD2L1 | 0.604938272 | 0.836379091 | 1 | 2 |
| PHYHD1 | 0.604938272 | 0.836379091 | 1 | 2 |
| STK36 | 0.604938272 | 0.836379091 | 1 | 2 |
| ZBTB11 | 0.604938272 | 0.836379091 | 1 | 2 |
| RABGGTA | 0.604938272 | 0.836379091 | 1 | 2 |
| TRBV5-3 | 0.604938272 | 0.836379091 | 1 | 2 |
| RAD50 | 0.604938272 | 0.836379091 | 1 | 2 |
| RAD52 | 0.604938272 | 0.836379091 | 1 | 2 |
| SLC44A1 | 0.604938272 | 0.836379091 | 1 | 2 |
| SLC44A2 | 0.604938272 | 0.836379091 | 1 | 2 |
| RALGAPA1 | 0.604938272 | 0.836379091 | 1 | 2 |
| RAMP3 | 0.604938272 | 0.836379091 | 1 | 2 |
| TRAF3IP3 | 0.604938272 | 0.836379091 | 1 | 2 |
| RAPGEF2 | 0.604938272 | 0.836379091 | 1 | 2 |
| STXBP5 | 0.604938272 | 0.836379091 | 1 | 2 |
| SPATA32 | 0.604938272 | 0.836379091 | 1 | 2 |
| ZCCHC8 | 0.604938272 | 0.836379091 | 1 | 2 |
| RARA | 0.604938272 | 0.836379091 | 1 | 2 |
| ZBTB1 | 0.604938272 | 0.836379091 | 1 | 2 |
| XYLB | 0.604938272 | 0.836379091 | 1 | 2 |
| PGR | 0.604938272 | 0.836379091 | 1 | 2 |
| TREML2 | 0.604938272 | 0.836379091 | 1 | 2 |
| XYLT2 | 0.604938272 | 0.836379091 | 1 | 2 |
| PHLDB1 | 0.604938272 | 0.836379091 | 1 | 2 |
| SLC39A3 | 0.604938272 | 0.836379091 | 1 | 2 |
| PHKG2 | 0.604938272 | 0.836379091 | 1 | 2 |
| SPERT | 0.604938272 | 0.836379091 | 1 | 2 |
| R3HCC1 | 0.604938272 | 0.836379091 | 1 | 2 |
| PHKA1 | 0.604938272 | 0.836379091 | 1 | 2 |
| PHIP | 0.604938272 | 0.836379091 | 1 | 2 |
| YIPF1 | 0.604938272 | 0.836379091 | 1 | 2 |
| STOX1 | 0.604938272 | 0.836379091 | 1 | 2 |
| SPDYE5 | 0.604938272 | 0.836379091 | 1 | 2 |
| RAB25 | 0.604938272 | 0.836379091 | 1 | 2 |
| TRIM11 | 0.604938272 | 0.836379091 | 1 | 2 |
| RAB3GAP1 | 0.604938272 | 0.836379091 | 1 | 2 |
| SLC16A11 | 0.604938272 | 0.836379091 | 1 | 2 |
| MAP3K20 | 0.604938272 | 0.836379091 | 1 | 2 |
| TPSG1 | 0.604938272 | 0.836379091 | 1 | 2 |
| PSTPIP2 | 0.604938272 | 0.836379091 | 1 | 2 |
| SLC22A24 | 0.604938272 | 0.836379091 | 1 | 2 |
| POLR2M | 0.604938272 | 0.836379091 | 1 | 2 |
| VARS2 | 0.604938272 | 0.836379091 | 1 | 2 |
| ULK4 | 0.604938272 | 0.836379091 | 1 | 2 |
| SSTR3 | 0.604938272 | 0.836379091 | 1 | 2 |
| VAV2 | 0.604938272 | 0.836379091 | 1 | 2 |
| SLC25A35 | 0.604938272 | 0.836379091 | 1 | 2 |
| SLC25A32 | 0.604938272 | 0.836379091 | 1 | 2 |
| PRCP | 0.604938272 | 0.836379091 | 1 | 2 |
| VN1R1 | 0.604938272 | 0.836379091 | 1 | 2 |
| PRDM15 | 0.604938272 | 0.836379091 | 1 | 2 |
| VPS11 | 0.604938272 | 0.836379091 | 1 | 2 |
| UBAP2 | 0.604938272 | 0.836379091 | 1 | 2 |
| SQSTM1 | 0.604938272 | 0.836379091 | 1 | 2 |
| PREPL | 0.604938272 | 0.836379091 | 1 | 2 |
| PREX1 | 0.604938272 | 0.836379091 | 1 | 2 |
| POM121L2 | 0.604938272 | 0.836379091 | 1 | 2 |
| UBA6 | 0.604938272 | 0.836379091 | 1 | 2 |
| SLC2A13 | 0.604938272 | 0.836379091 | 1 | 2 |
| UNC93B1 | 0.604938272 | 0.836379091 | 1 | 2 |
| SLC26A7 | 0.604938272 | 0.836379091 | 1 | 2 |
| PPFIA2 | 0.604938272 | 0.836379091 | 1 | 2 |
| PPEF2 | 0.604938272 | 0.836379091 | 1 | 2 |
| PPIL2 | 0.604938272 | 0.836379091 | 1 | 2 |
| SLC28A1 | 0.604938272 | 0.836379091 | 1 | 2 |
| SRPK3 | 0.604938272 | 0.836379091 | 1 | 2 |
| PLPP1 | 0.604938272 | 0.836379091 | 1 | 2 |
| PPP1R14B | 0.604938272 | 0.836379091 | 1 | 2 |
| PPP1R16B | 0.604938272 | 0.836379091 | 1 | 2 |
| SLC28A3 | 0.604938272 | 0.836379091 | 1 | 2 |
| URGCP | 0.604938272 | 0.836379091 | 1 | 2 |
| SRI | 0.604938272 | 0.836379091 | 1 | 2 |
| UTP14C | 0.604938272 | 0.836379091 | 1 | 2 |
| PPP2R3A | 0.604938272 | 0.836379091 | 1 | 2 |
| UXS1 | 0.604938272 | 0.836379091 | 1 | 2 |
| POMGNT1 | 0.604938272 | 0.836379091 | 1 | 2 |
| TSKU | 0.604938272 | 0.836379091 | 1 | 2 |
| SPTLC2 | 0.604938272 | 0.836379091 | 1 | 2 |
| PRKAR1B | 0.604938272 | 0.836379091 | 1 | 2 |
| PRSS41 | 0.604938272 | 0.836379091 | 1 | 2 |
| WDR54 | 0.604938272 | 0.836379091 | 1 | 2 |
| SPPL2C | 0.604938272 | 0.836379091 | 1 | 2 |
| CFAP57 | 0.604938272 | 0.836379091 | 1 | 2 |
| PRTFDC1 | 0.604938272 | 0.836379091 | 1 | 2 |
| WDR72 | 0.604938272 | 0.836379091 | 1 | 2 |
| TTC17 | 0.604938272 | 0.836379091 | 1 | 2 |
| WDR73 | 0.604938272 | 0.836379091 | 1 | 2 |
| SLC22A7 | 0.604938272 | 0.836379091 | 1 | 2 |
| TSTA3 | 0.604938272 | 0.836379091 | 1 | 2 |
| PSD | 0.604938272 | 0.836379091 | 1 | 2 |
| SLC35G3 | 0.604938272 | 0.836379091 | 1 | 2 |
| PLA2G4E | 0.604938272 | 0.836379091 | 1 | 2 |
| PLA2G4D | 0.604938272 | 0.836379091 | 1 | 2 |
| PSMD2 | 0.604938272 | 0.836379091 | 1 | 2 |
| TTC24 | 0.604938272 | 0.836379091 | 1 | 2 |
| UBA1 | 0.604938272 | 0.836379091 | 1 | 2 |
| PLCL2 | 0.604938272 | 0.836379091 | 1 | 2 |
| PLD2 | 0.604938272 | 0.836379091 | 1 | 2 |
| PMFBP1 | 0.604938272 | 0.836379091 | 1 | 2 |
| SPSB4 | 0.604938272 | 0.836379091 | 1 | 2 |
| TXNRD2 | 0.604938272 | 0.836379091 | 1 | 2 |
| VWA1 | 0.604938272 | 0.836379091 | 1 | 2 |
| TULP3 | 0.604938272 | 0.836379091 | 1 | 2 |
| TULP1 | 0.604938272 | 0.836379091 | 1 | 2 |
| PRODH2 | 0.604938272 | 0.836379091 | 1 | 2 |
| PRPF40A | 0.604938272 | 0.836379091 | 1 | 2 |
| TTF2 | 0.604938272 | 0.836379091 | 1 | 2 |
| PLEKHG1 | 0.604938272 | 0.836379091 | 1 | 2 |
| PLEKHB2 | 0.604938272 | 0.836379091 | 1 | 2 |
| PLEKHA7 | 0.604938272 | 0.836379091 | 1 | 2 |
| PLEKHA5 | 0.604938272 | 0.836379091 | 1 | 2 |
| TTC30A | 0.604938272 | 0.836379091 | 1 | 2 |
| TTC29 | 0.604938272 | 0.836379091 | 1 | 2 |
| PRRT4 | 0.604938272 | 0.836379091 | 1 | 2 |
| RXFP2 | 0.604938272 | 0.836379091 | 1 | 2 |
| RARS2 | 0.604938272 | 0.836379091 | 1 | 2 |
| RASA3 | 0.604938272 | 0.836379091 | 1 | 2 |
| TM4SF5 | 0.604938272 | 0.836379091 | 1 | 2 |
| OVCH1 | 0.604938272 | 0.836379091 | 1 | 2 |
| SH2D3C | 0.604938272 | 0.836379091 | 1 | 2 |
| RPA1 | 0.604938272 | 0.836379091 | 1 | 2 |
| OTUD1 | 0.604938272 | 0.836379091 | 1 | 2 |
| OTOP3 | 0.604938272 | 0.836379091 | 1 | 2 |
| SH2B2 | 0.604938272 | 0.836379091 | 1 | 2 |
| TLR10 | 0.604938272 | 0.836379091 | 1 | 2 |
| ZNF474 | 0.604938272 | 0.836379091 | 1 | 2 |
| TLL2 | 0.604938272 | 0.836379091 | 1 | 2 |
| RPH3AL | 0.604938272 | 0.836379091 | 1 | 2 |
| ZNF507 | 0.604938272 | 0.836379091 | 1 | 2 |
| SLC25A15 | 0.604938272 | 0.836379091 | 1 | 2 |
| RPL13 | 0.604938272 | 0.836379091 | 1 | 2 |
| OR9K2 | 0.604938272 | 0.836379091 | 1 | 2 |
| P2RX1 | 0.604938272 | 0.836379091 | 1 | 2 |
| OR9I1 | 0.604938272 | 0.836379091 | 1 | 2 |
| P2RX2 | 0.604938272 | 0.836379091 | 1 | 2 |
| SOX18 | 0.604938272 | 0.836379091 | 1 | 2 |
| ZNF33B | 0.604938272 | 0.836379091 | 1 | 2 |
| PAK6 | 0.604938272 | 0.836379091 | 1 | 2 |
| ZNF354A | 0.604938272 | 0.836379091 | 1 | 2 |
| ZNF354C | 0.604938272 | 0.836379091 | 1 | 2 |
| RNF144A | 0.604938272 | 0.836379091 | 1 | 2 |
| ZNF396 | 0.604938272 | 0.836379091 | 1 | 2 |
| RNF168 | 0.604938272 | 0.836379091 | 1 | 2 |
| ZNF398 | 0.604938272 | 0.836379091 | 1 | 2 |
| TMEM131 | 0.604938272 | 0.836379091 | 1 | 2 |
| PACSIN1 | 0.604938272 | 0.836379091 | 1 | 2 |
| PACRGL | 0.604938272 | 0.836379091 | 1 | 2 |
| RNF25 | 0.604938272 | 0.836379091 | 1 | 2 |
| ZNF417 | 0.604938272 | 0.836379091 | 1 | 2 |
| ZNF425 | 0.604938272 | 0.836379091 | 1 | 2 |
| RNH1 | 0.604938272 | 0.836379091 | 1 | 2 |
| TMC8 | 0.604938272 | 0.836379091 | 1 | 2 |
| ZNF335 | 0.604938272 | 0.836379091 | 1 | 2 |
| OR8K3 | 0.604938272 | 0.836379091 | 1 | 2 |
| RPRD2 | 0.604938272 | 0.836379091 | 1 | 2 |
| TGOLN2 | 0.604938272 | 0.836379091 | 1 | 2 |
| TGM5 | 0.604938272 | 0.836379091 | 1 | 2 |
| TGM1 | 0.604938272 | 0.836379091 | 1 | 2 |
| SYT6 | 0.604938272 | 0.836379091 | 1 | 2 |
| RTKN | 0.604938272 | 0.836379091 | 1 | 2 |
| SYTL1 | 0.604938272 | 0.836379091 | 1 | 2 |
| SYTL3 | 0.604938272 | 0.836379091 | 1 | 2 |
| RTN4RL1 | 0.604938272 | 0.836379091 | 1 | 2 |
| ZNF654 | 0.604938272 | 0.836379091 | 1 | 2 |
| RUFY1 | 0.604938272 | 0.836379091 | 1 | 2 |
| SMC6 | 0.604938272 | 0.836379091 | 1 | 2 |
| ZNF671 | 0.604938272 | 0.836379091 | 1 | 2 |
| ZNF675 | 0.604938272 | 0.836379091 | 1 | 2 |
| T | 0.604938272 | 0.836379091 | 1 | 2 |
| ZNF689 | 0.604938272 | 0.836379091 | 1 | 2 |
| RSPRY1 | 0.604938272 | 0.836379091 | 1 | 2 |
| ZNF528 | 0.604938272 | 0.836379091 | 1 | 2 |
| ZNF616 | 0.604938272 | 0.836379091 | 1 | 2 |
| ZNF611 | 0.604938272 | 0.836379091 | 1 | 2 |
| SYT14 | 0.604938272 | 0.836379091 | 1 | 2 |
| ZNF551 | 0.604938272 | 0.836379091 | 1 | 2 |
| SOS1 | 0.604938272 | 0.836379091 | 1 | 2 |
| THUMPD3 | 0.604938272 | 0.836379091 | 1 | 2 |
| ZNF558 | 0.604938272 | 0.836379091 | 1 | 2 |
| RPS6KA2 | 0.604938272 | 0.836379091 | 1 | 2 |
| OR6C65 | 0.604938272 | 0.836379091 | 1 | 2 |
| THNSL1 | 0.604938272 | 0.836379091 | 1 | 2 |
| OR5L1 | 0.604938272 | 0.836379091 | 1 | 2 |
| RRAGD | 0.604938272 | 0.836379091 | 1 | 2 |
| THEG | 0.604938272 | 0.836379091 | 1 | 2 |
| RREB1 | 0.604938272 | 0.836379091 | 1 | 2 |
| RRP1B | 0.604938272 | 0.836379091 | 1 | 2 |
| ZNF605 | 0.604938272 | 0.836379091 | 1 | 2 |
| OR5K1 | 0.604938272 | 0.836379091 | 1 | 2 |
| SOBP | 0.604938272 | 0.836379091 | 1 | 2 |
| ZDHHC13 | 0.604938272 | 0.836379091 | 1 | 2 |
| ZNF333 | 0.604938272 | 0.836379091 | 1 | 2 |
| PAN3 | 0.604938272 | 0.836379091 | 1 | 2 |
| ZFP2 | 0.604938272 | 0.836379091 | 1 | 2 |
| ZFP41 | 0.604938272 | 0.836379091 | 1 | 2 |
| PDE6C | 0.604938272 | 0.836379091 | 1 | 2 |
| PDE3A | 0.604938272 | 0.836379091 | 1 | 2 |
| SIRT6 | 0.604938272 | 0.836379091 | 1 | 2 |
| PDCD2 | 0.604938272 | 0.836379091 | 1 | 2 |
| TNFSF10 | 0.604938272 | 0.836379091 | 1 | 2 |
| TNFRSF4 | 0.604938272 | 0.836379091 | 1 | 2 |
| SIRPB1 | 0.604938272 | 0.836379091 | 1 | 2 |
| PCNX2 | 0.604938272 | 0.836379091 | 1 | 2 |
| ZIC5 | 0.604938272 | 0.836379091 | 1 | 2 |
| TMX3 | 0.604938272 | 0.836379091 | 1 | 2 |
| ZMIZ2 | 0.604938272 | 0.836379091 | 1 | 2 |
| PCGF2 | 0.604938272 | 0.836379091 | 1 | 2 |
| PCF11 | 0.604938272 | 0.836379091 | 1 | 2 |
| SLC6A16 | 0.604938272 | 0.836379091 | 1 | 2 |
| SPATA17 | 0.604938272 | 0.836379091 | 1 | 2 |
| ZFC3H1 | 0.604938272 | 0.836379091 | 1 | 2 |
| PDIA4 | 0.604938272 | 0.836379091 | 1 | 2 |
| ZDHHC19 | 0.604938272 | 0.836379091 | 1 | 2 |
| RASAL2 | 0.604938272 | 0.836379091 | 1 | 2 |
| SLC4A10 | 0.604938272 | 0.836379091 | 1 | 2 |
| PDZK1IP1 | 0.604938272 | 0.836379091 | 1 | 2 |
| RASSF1 | 0.604938272 | 0.836379091 | 1 | 2 |
| ZDHHC23 | 0.604938272 | 0.836379091 | 1 | 2 |
| TPCN1 | 0.604938272 | 0.836379091 | 1 | 2 |
| SPATA31C1 | 0.604938272 | 0.836379091 | 1 | 2 |
| TP73 | 0.604938272 | 0.836379091 | 1 | 2 |
| TP53BP2 | 0.604938272 | 0.836379091 | 1 | 2 |
| RBFOX1 | 0.604938272 | 0.836379091 | 1 | 2 |
| TOR1AIP2 | 0.604938272 | 0.836379091 | 1 | 2 |
| RBL1 | 0.604938272 | 0.836379091 | 1 | 2 |
| SKIDA1 | 0.604938272 | 0.836379091 | 1 | 2 |
| ZEB2 | 0.604938272 | 0.836379091 | 1 | 2 |
| SULF1 | 0.604938272 | 0.836379091 | 1 | 2 |
| ZNF331 | 0.604938272 | 0.836379091 | 1 | 2 |
| SLC7A4 | 0.604938272 | 0.836379091 | 1 | 2 |
| TMPRSS11A | 0.604938272 | 0.836379091 | 1 | 2 |
| ZNF254 | 0.604938272 | 0.836379091 | 1 | 2 |
| PARVB | 0.604938272 | 0.836379091 | 1 | 2 |
| SHF | 0.604938272 | 0.836379091 | 1 | 2 |
| PARS2 | 0.604938272 | 0.836379091 | 1 | 2 |
| PARP9 | 0.604938272 | 0.836379091 | 1 | 2 |
| RIMKLA | 0.604938272 | 0.836379091 | 1 | 2 |
| RINT1 | 0.604938272 | 0.836379091 | 1 | 2 |
| TMEM190 | 0.604938272 | 0.836379091 | 1 | 2 |
| SOX5 | 0.604938272 | 0.836379091 | 1 | 2 |
| RLN1 | 0.604938272 | 0.836379091 | 1 | 2 |
| TMEM184A | 0.604938272 | 0.836379091 | 1 | 2 |
| SHC4 | 0.604938272 | 0.836379091 | 1 | 2 |
| ACP7 | 0.604938272 | 0.836379091 | 1 | 2 |
| SHC1 | 0.604938272 | 0.836379091 | 1 | 2 |
| ZNF329 | 0.604938272 | 0.836379091 | 1 | 2 |
| PATE1 | 0.604938272 | 0.836379091 | 1 | 2 |
| TMPRSS12 | 0.604938272 | 0.836379091 | 1 | 2 |
| SV2B | 0.604938272 | 0.836379091 | 1 | 2 |
| RIC8A | 0.604938272 | 0.836379091 | 1 | 2 |
| SULT1C3 | 0.604938272 | 0.836379091 | 1 | 2 |
| ZNF155 | 0.604938272 | 0.836379091 | 1 | 2 |
| RFPL3 | 0.604938272 | 0.836379091 | 1 | 2 |
| ZNF180 | 0.604938272 | 0.836379091 | 1 | 2 |
| ZNF185 | 0.604938272 | 0.836379091 | 1 | 2 |
| SUN5 | 0.604938272 | 0.836379091 | 1 | 2 |
| ZNF121 | 0.604938272 | 0.836379091 | 1 | 2 |
| RFX4 | 0.604938272 | 0.836379091 | 1 | 2 |
| SPAG11B | 0.604938272 | 0.836379091 | 1 | 2 |
| PCDHA8 | 0.604938272 | 0.836379091 | 1 | 2 |
| RGS11 | 0.604938272 | 0.836379091 | 1 | 2 |
| TMEM259 | 0.604938272 | 0.836379091 | 1 | 2 |
| RHOXF1 | 0.604938272 | 0.836379091 | 1 | 2 |
| ZNF232 | 0.604938272 | 0.836379091 | 1 | 2 |
| ZNF234 | 0.604938272 | 0.836379091 | 1 | 2 |
| SP8 | 0.604938272 | 0.836379091 | 1 | 2 |
| RECQL5 | 0.604938272 | 0.836379091 | 1 | 2 |
| A1BG | 0.604938272 | 0.836379091 | 1 | 2 |
| CDCA7L | 0.604938272 | 0.836379091 | 1 | 2 |
| KCNN3 | 0.604938272 | 0.836379091 | 1 | 2 |
| CAMSAP3 | 0.604938272 | 0.836379091 | 1 | 2 |
| KCNK10 | 0.604938272 | 0.836379091 | 1 | 2 |
| KCNIP2 | 0.604938272 | 0.836379091 | 1 | 2 |
| CARD6 | 0.604938272 | 0.836379091 | 1 | 2 |
| KCNA5 | 0.604938272 | 0.836379091 | 1 | 2 |
| NAXD | 0.604938272 | 0.836379091 | 1 | 2 |
| KAZN | 0.604938272 | 0.836379091 | 1 | 2 |
| KATNB1 | 0.604938272 | 0.836379091 | 1 | 2 |
| AMOTL1 | 0.604938272 | 0.836379091 | 1 | 2 |
| KANK3 | 0.604938272 | 0.836379091 | 1 | 2 |
| AMH | 0.604938272 | 0.836379091 | 1 | 2 |
| KALRN | 0.604938272 | 0.836379091 | 1 | 2 |
| CATSPER1 | 0.604938272 | 0.836379091 | 1 | 2 |
| CHAMP1 | 0.604938272 | 0.836379091 | 1 | 2 |
| JAK2 | 0.604938272 | 0.836379091 | 1 | 2 |
| AMDHD2 | 0.604938272 | 0.836379091 | 1 | 2 |
| CBLB | 0.604938272 | 0.836379091 | 1 | 2 |
| IZUMO4 | 0.604938272 | 0.836379091 | 1 | 2 |
| C2CD6 | 0.604938272 | 0.836379091 | 1 | 2 |
| ALPK1 | 0.604938272 | 0.836379091 | 1 | 2 |
| NAALADL2 | 0.604938272 | 0.836379091 | 1 | 2 |
| ALOX12 | 0.604938272 | 0.836379091 | 1 | 2 |
| CCDC120 | 0.604938272 | 0.836379091 | 1 | 2 |
| ALKBH8 | 0.604938272 | 0.836379091 | 1 | 2 |
| NADSYN1 | 0.604938272 | 0.836379091 | 1 | 2 |
| NAGK | 0.604938272 | 0.836379091 | 1 | 2 |
| NAGPA | 0.604938272 | 0.836379091 | 1 | 2 |
| IRX1 | 0.604938272 | 0.836379091 | 1 | 2 |
| IPO8 | 0.604938272 | 0.836379091 | 1 | 2 |
| NAA25 | 0.604938272 | 0.836379091 | 1 | 2 |
| KCNS3 | 0.604938272 | 0.836379091 | 1 | 2 |
| KCNT1 | 0.604938272 | 0.836379091 | 1 | 2 |
| CALHM3 | 0.604938272 | 0.836379091 | 1 | 2 |
| KRT10 | 0.604938272 | 0.836379091 | 1 | 2 |
| C8orf58 | 0.604938272 | 0.836379091 | 1 | 2 |
| C9orf131 | 0.604938272 | 0.836379091 | 1 | 2 |
| KLHL30 | 0.604938272 | 0.836379091 | 1 | 2 |
| KLHL26 | 0.604938272 | 0.836379091 | 1 | 2 |
| C9orf3 | 0.604938272 | 0.836379091 | 1 | 2 |
| KLHL17 | 0.604938272 | 0.836379091 | 1 | 2 |
| KLHL12 | 0.604938272 | 0.836379091 | 1 | 2 |
| CARD19 | 0.604938272 | 0.836379091 | 1 | 2 |
| KLB | 0.604938272 | 0.836379091 | 1 | 2 |
| KIRREL2 | 0.604938272 | 0.836379091 | 1 | 2 |
| KIRREL1 | 0.604938272 | 0.836379091 | 1 | 2 |
| KIFC2 | 0.604938272 | 0.836379091 | 1 | 2 |
| MYCN | 0.604938272 | 0.836379091 | 1 | 2 |
| ANKRD34A | 0.604938272 | 0.836379091 | 1 | 2 |
| MYEOV | 0.604938272 | 0.836379091 | 1 | 2 |
| KIF27 | 0.604938272 | 0.836379091 | 1 | 2 |
| KDM4B | 0.604938272 | 0.836379091 | 1 | 2 |
| KDR | 0.604938272 | 0.836379091 | 1 | 2 |
| KIAA0319L | 0.604938272 | 0.836379091 | 1 | 2 |
| KIAA1210 | 0.604938272 | 0.836379091 | 1 | 2 |
| KIAA1324 | 0.604938272 | 0.836379091 | 1 | 2 |
| KIAA1328 | 0.604938272 | 0.836379091 | 1 | 2 |
| IP6K3 | 0.604938272 | 0.836379091 | 1 | 2 |
| JCAD | 0.604938272 | 0.836379091 | 1 | 2 |
| ANKRD16 | 0.604938272 | 0.836379091 | 1 | 2 |
| SLF1 | 0.604938272 | 0.836379091 | 1 | 2 |
| CCAR2 | 0.604938272 | 0.836379091 | 1 | 2 |
| KIF19 | 0.604938272 | 0.836379091 | 1 | 2 |
| ANKRD33B | 0.604938272 | 0.836379091 | 1 | 2 |
| MYH7 | 0.604938272 | 0.836379091 | 1 | 2 |
| ANKMY2 | 0.604938272 | 0.836379091 | 1 | 2 |
| ANKS1A | 0.604938272 | 0.836379091 | 1 | 2 |
| NAPG | 0.604938272 | 0.836379091 | 1 | 2 |
| BICDL2 | 0.604938272 | 0.836379091 | 1 | 2 |
| CEACAM6 | 0.604938272 | 0.836379091 | 1 | 2 |
| NDNF | 0.604938272 | 0.836379091 | 1 | 2 |
| CEACAM8 | 0.604938272 | 0.836379091 | 1 | 2 |
| NDOR1 | 0.604938272 | 0.836379091 | 1 | 2 |
| CEND1 | 0.604938272 | 0.836379091 | 1 | 2 |
| NDUFAF4 | 0.604938272 | 0.836379091 | 1 | 2 |
| CEP135 | 0.604938272 | 0.836379091 | 1 | 2 |
| CEP164 | 0.604938272 | 0.836379091 | 1 | 2 |
| HLTF | 0.604938272 | 0.836379091 | 1 | 2 |
| AKNA | 0.604938272 | 0.836379091 | 1 | 2 |
| HLCS | 0.604938272 | 0.836379091 | 1 | 2 |
| CEP170B | 0.604938272 | 0.836379091 | 1 | 2 |
| NDUFS1 | 0.604938272 | 0.836379091 | 1 | 2 |
| CERKL | 0.604938272 | 0.836379091 | 1 | 2 |
| HPN | 0.604938272 | 0.836379091 | 1 | 2 |
| HHIPL2 | 0.604938272 | 0.836379091 | 1 | 2 |
| AK8 | 0.604938272 | 0.836379091 | 1 | 2 |
| HERC6 | 0.604938272 | 0.836379091 | 1 | 2 |
| CHD8 | 0.604938272 | 0.836379091 | 1 | 2 |
| HCFC1R1 | 0.604938272 | 0.836379091 | 1 | 2 |
| CHPF2 | 0.604938272 | 0.836379091 | 1 | 2 |
| GYS1 | 0.604938272 | 0.836379091 | 1 | 2 |
| CHRNB4 | 0.604938272 | 0.836379091 | 1 | 2 |
| GUCY1A3 | 0.604938272 | 0.836379091 | 1 | 2 |
| GTDC1 | 0.604938272 | 0.836379091 | 1 | 2 |
| GSX1 | 0.604938272 | 0.836379091 | 1 | 2 |
| CLCC1 | 0.604938272 | 0.836379091 | 1 | 2 |
| GSTA1 | 0.604938272 | 0.836379091 | 1 | 2 |
| CLDND1 | 0.604938272 | 0.836379091 | 1 | 2 |
| NELL1 | 0.604938272 | 0.836379091 | 1 | 2 |
| NDUFV2 | 0.604938272 | 0.836379091 | 1 | 2 |
| HPSE | 0.604938272 | 0.836379091 | 1 | 2 |
| CDKL3 | 0.604938272 | 0.836379091 | 1 | 2 |
| CDKL1 | 0.604938272 | 0.836379091 | 1 | 2 |
| INF2 | 0.604938272 | 0.836379091 | 1 | 2 |
| SPPL2B | 0.604938272 | 0.836379091 | 1 | 2 |
| ILDR2 | 0.604938272 | 0.836379091 | 1 | 2 |
| CCDC88A | 0.604938272 | 0.836379091 | 1 | 2 |
| ALG10 | 0.604938272 | 0.836379091 | 1 | 2 |
| IKBKB | 0.604938272 | 0.836379091 | 1 | 2 |
| CCSER2 | 0.604938272 | 0.836379091 | 1 | 2 |
| IGSF1 | 0.604938272 | 0.836379091 | 1 | 2 |
| CD2 | 0.604938272 | 0.836379091 | 1 | 2 |
| CD44 | 0.604938272 | 0.836379091 | 1 | 2 |
| IGHV3-21 | 0.604938272 | 0.836379091 | 1 | 2 |
| CD86 | 0.604938272 | 0.836379091 | 1 | 2 |
| IGDCC3 | 0.604938272 | 0.836379091 | 1 | 2 |
| ALDH8A1 | 0.604938272 | 0.836379091 | 1 | 2 |
| NBPF8 | 0.604938272 | 0.836379091 | 1 | 2 |
| IFNL3 | 0.604938272 | 0.836379091 | 1 | 2 |
| CDC42BPA | 0.604938272 | 0.836379091 | 1 | 2 |
| HSD3B7 | 0.604938272 | 0.836379091 | 1 | 2 |
| CDK14 | 0.604938272 | 0.836379091 | 1 | 2 |
| CDHR5 | 0.604938272 | 0.836379091 | 1 | 2 |
| CDH19 | 0.604938272 | 0.836379091 | 1 | 2 |
| HTR3D | 0.604938272 | 0.836379091 | 1 | 2 |
| CDH17 | 0.604938272 | 0.836379091 | 1 | 2 |
| INPP1 | 0.604938272 | 0.836379091 | 1 | 2 |
| CDH12 | 0.604938272 | 0.836379091 | 1 | 2 |
| FZR1 | 0.604938272 | 0.836379091 | 1 | 2 |
| HYLS1 | 0.604938272 | 0.836379091 | 1 | 2 |
| IARS2 | 0.604938272 | 0.836379091 | 1 | 2 |
| NCF4 | 0.604938272 | 0.836379091 | 1 | 2 |
| ALDH3B1 | 0.604938272 | 0.836379091 | 1 | 2 |
| ALDH3B2 | 0.604938272 | 0.836379091 | 1 | 2 |
| ALDH1A1 | 0.604938272 | 0.836379091 | 1 | 2 |
| GRIK1 | 0.604938272 | 0.836379091 | 1 | 2 |
| KRT28 | 0.604938272 | 0.836379091 | 1 | 2 |
| C3orf58 | 0.604938272 | 0.836379091 | 1 | 2 |
| MOG | 0.604938272 | 0.836379091 | 1 | 2 |
| MAP7D1 | 0.604938272 | 0.836379091 | 1 | 2 |
| MAP3K2 | 0.604938272 | 0.836379091 | 1 | 2 |
| MAP2K7 | 0.604938272 | 0.836379091 | 1 | 2 |
| ARHGEF16 | 0.604938272 | 0.836379091 | 1 | 2 |
| ATRIP | 0.604938272 | 0.836379091 | 1 | 2 |
| MAP2K1 | 0.604938272 | 0.836379091 | 1 | 2 |
| ARHGAP40 | 0.604938272 | 0.836379091 | 1 | 2 |
| MAN1A2 | 0.604938272 | 0.836379091 | 1 | 2 |
| AUNIP | 0.604938272 | 0.836379091 | 1 | 2 |
| MOV10 | 0.604938272 | 0.836379091 | 1 | 2 |
| MAGEB6 | 0.604938272 | 0.836379091 | 1 | 2 |
| AXDND1 | 0.604938272 | 0.836379091 | 1 | 2 |
| AXL | 0.604938272 | 0.836379091 | 1 | 2 |
| MAP7D3 | 0.604938272 | 0.836379091 | 1 | 2 |
| B3GLCT | 0.604938272 | 0.836379091 | 1 | 2 |
| B3GNTL1 | 0.604938272 | 0.836379091 | 1 | 2 |
| MACC1 | 0.604938272 | 0.836379091 | 1 | 2 |
| ARHGAP18 | 0.604938272 | 0.836379091 | 1 | 2 |
| BAHD1 | 0.604938272 | 0.836379091 | 1 | 2 |
| LZTS2 | 0.604938272 | 0.836379091 | 1 | 2 |
| LZTS1 | 0.604938272 | 0.836379091 | 1 | 2 |
| BAZ1A | 0.604938272 | 0.836379091 | 1 | 2 |
| BBS10 | 0.604938272 | 0.836379091 | 1 | 2 |
| BBX | 0.604938272 | 0.836379091 | 1 | 2 |
| LTV1 | 0.604938272 | 0.836379091 | 1 | 2 |
| BCAT1 | 0.604938272 | 0.836379091 | 1 | 2 |
| LRRC45 | 0.604938272 | 0.836379091 | 1 | 2 |
| BEND2 | 0.604938272 | 0.836379091 | 1 | 2 |
| BHLHE41 | 0.604938272 | 0.836379091 | 1 | 2 |
| MAFA | 0.604938272 | 0.836379091 | 1 | 2 |
| MAP9 | 0.604938272 | 0.836379091 | 1 | 2 |
| MAPK8IP2 | 0.604938272 | 0.836379091 | 1 | 2 |
| MARK3 | 0.604938272 | 0.836379091 | 1 | 2 |
| MGST1 | 0.604938272 | 0.836379091 | 1 | 2 |
| MITF | 0.604938272 | 0.836379091 | 1 | 2 |
| MOGAT1 | 0.604938272 | 0.836379091 | 1 | 2 |
| ARSB | 0.604938272 | 0.836379091 | 1 | 2 |
| MEX3B | 0.604938272 | 0.836379091 | 1 | 2 |
| MEP1A | 0.604938272 | 0.836379091 | 1 | 2 |
| MEGF9 | 0.604938272 | 0.836379091 | 1 | 2 |
| MLF2 | 0.604938272 | 0.836379091 | 1 | 2 |
| MLH1 | 0.604938272 | 0.836379091 | 1 | 2 |
| ASMT | 0.604938272 | 0.836379091 | 1 | 2 |
| MEGF11 | 0.604938272 | 0.836379091 | 1 | 2 |
| GDNF | 0.604938272 | 0.836379091 | 1 | 2 |
| MLNR | 0.604938272 | 0.836379091 | 1 | 2 |
| MLPH | 0.604938272 | 0.836379091 | 1 | 2 |
| MED1 | 0.604938272 | 0.836379091 | 1 | 2 |
| MECP2 | 0.604938272 | 0.836379091 | 1 | 2 |
| MCTP2 | 0.604938272 | 0.836379091 | 1 | 2 |
| MARK4 | 0.604938272 | 0.836379091 | 1 | 2 |
| ATP6V0D1 | 0.604938272 | 0.836379091 | 1 | 2 |
| MMP28 | 0.604938272 | 0.836379091 | 1 | 2 |
| MATN2 | 0.604938272 | 0.836379091 | 1 | 2 |
| MAZ | 0.604938272 | 0.836379091 | 1 | 2 |
| MBD1 | 0.604938272 | 0.836379091 | 1 | 2 |
| BICD1 | 0.604938272 | 0.836379091 | 1 | 2 |
| ARHGEF7 | 0.604938272 | 0.836379091 | 1 | 2 |
| ARID1A | 0.604938272 | 0.836379091 | 1 | 2 |
| MCCD1 | 0.604938272 | 0.836379091 | 1 | 2 |
| MMP1 | 0.604938272 | 0.836379091 | 1 | 2 |
| MMACHC | 0.604938272 | 0.836379091 | 1 | 2 |
| ATIC | 0.604938272 | 0.836379091 | 1 | 2 |
| ARID3C | 0.604938272 | 0.836379091 | 1 | 2 |
| ATP1A1 | 0.604938272 | 0.836379091 | 1 | 2 |
| KRT79 | 0.604938272 | 0.836379091 | 1 | 2 |
| LRR1 | 0.604938272 | 0.836379091 | 1 | 2 |
| BIK | 0.604938272 | 0.836379091 | 1 | 2 |
| AOC3 | 0.604938272 | 0.836379091 | 1 | 2 |
| TEDC2 | 0.604938272 | 0.836379091 | 1 | 2 |
| LGALS14 | 0.604938272 | 0.836379091 | 1 | 2 |
| MSL1 | 0.604938272 | 0.836379091 | 1 | 2 |
| MSL2 | 0.604938272 | 0.836379091 | 1 | 2 |
| P3H4 | 0.604938272 | 0.836379091 | 1 | 2 |
| C18orf54 | 0.604938272 | 0.836379091 | 1 | 2 |
| LENG1 | 0.604938272 | 0.836379091 | 1 | 2 |
| MTBP | 0.604938272 | 0.836379091 | 1 | 2 |
| C18orf8 | 0.604938272 | 0.836379091 | 1 | 2 |
| LCTL | 0.604938272 | 0.836379091 | 1 | 2 |
| LCN1 | 0.604938272 | 0.836379091 | 1 | 2 |
| C1orf100 | 0.604938272 | 0.836379091 | 1 | 2 |
| LCE1E | 0.604938272 | 0.836379091 | 1 | 2 |
| C15orf40 | 0.604938272 | 0.836379091 | 1 | 2 |
| LCE1D | 0.604938272 | 0.836379091 | 1 | 2 |
| LARP4B | 0.604938272 | 0.836379091 | 1 | 2 |
| C1orf167 | 0.604938272 | 0.836379091 | 1 | 2 |
| MTG1 | 0.604938272 | 0.836379091 | 1 | 2 |
| MTG2 | 0.604938272 | 0.836379091 | 1 | 2 |
| L3MBTL4 | 0.604938272 | 0.836379091 | 1 | 2 |
| C1orf35 | 0.604938272 | 0.836379091 | 1 | 2 |
| L1TD1 | 0.604938272 | 0.836379091 | 1 | 2 |
| KYNU | 0.604938272 | 0.836379091 | 1 | 2 |
| KRTAP9-7 | 0.604938272 | 0.836379091 | 1 | 2 |
| KRTAP5-8 | 0.604938272 | 0.836379091 | 1 | 2 |
| KRTAP5-4 | 0.604938272 | 0.836379091 | 1 | 2 |
| MTMR2 | 0.604938272 | 0.836379091 | 1 | 2 |
| KRTAP5-11 | 0.604938272 | 0.836379091 | 1 | 2 |
| KRTAP4-8 | 0.604938272 | 0.836379091 | 1 | 2 |
| LARS2 | 0.604938272 | 0.836379091 | 1 | 2 |
| RIOX1 | 0.604938272 | 0.836379091 | 1 | 2 |
| LRRC74A | 0.604938272 | 0.836379091 | 1 | 2 |
| LIMK1 | 0.604938272 | 0.836379091 | 1 | 2 |
| LRP3 | 0.604938272 | 0.836379091 | 1 | 2 |
| LRGUK | 0.604938272 | 0.836379091 | 1 | 2 |
| BLOC1S3 | 0.604938272 | 0.836379091 | 1 | 2 |
| LRCH2 | 0.604938272 | 0.836379091 | 1 | 2 |
| LPCAT1 | 0.604938272 | 0.836379091 | 1 | 2 |
| LONRF1 | 0.604938272 | 0.836379091 | 1 | 2 |
| BOK | 0.604938272 | 0.836379091 | 1 | 2 |
| LOC730268 | 0.604938272 | 0.836379091 | 1 | 2 |
| BPIFB1 | 0.604938272 | 0.836379091 | 1 | 2 |
| BRAF | 0.604938272 | 0.836379091 | 1 | 2 |
| BRD1 | 0.604938272 | 0.836379091 | 1 | 2 |
| MRPL48 | 0.604938272 | 0.836379091 | 1 | 2 |
| LOC400499 | 0.604938272 | 0.836379091 | 1 | 2 |
| BRD2 | 0.604938272 | 0.836379091 | 1 | 2 |
| BRD4 | 0.604938272 | 0.836379091 | 1 | 2 |
| BRD7 | 0.604938272 | 0.836379091 | 1 | 2 |
| LOC152586 | 0.604938272 | 0.836379091 | 1 | 2 |
| C11orf86 | 0.604938272 | 0.836379091 | 1 | 2 |
| LMLN | 0.604938272 | 0.836379091 | 1 | 2 |
| LMOD3 | 0.604938272 | 0.836379091 | 1 | 2 |
| MRTO4 | 0.604938272 | 0.836379091 | 1 | 2 |
| MRPS9 | 0.604938272 | 0.836379091 | 1 | 2 |
| LOC100288524 | 0.604938272 | 0.836379091 | 1 | 2 |
| ARAP3 | 0.604938272 | 0.836379091 | 1 | 2 |
| LMNTD2 | 0.604938272 | 0.836379091 | 1 | 2 |
| VSIR | 0.604938272 | 0.836379091 | 1 | 2 |
| APBB2 | 0.604938272 | 0.836379091 | 1 | 2 |
| LOC101927423 | 0.604938272 | 0.836379091 | 1 | 2 |
| BTNL3 | 0.604938272 | 0.836379091 | 1 | 2 |
| LOC101928664 | 0.604938272 | 0.836379091 | 1 | 2 |
| BRINP1 | 0.604938272 | 0.836379091 | 1 | 2 |
| EMSY | 0.604938272 | 0.836379091 | 1 | 2 |
| GRHPR | 0.604938272 | 0.836379091 | 1 | 2 |
| ARNT | 0.604938272 | 0.836379091 | 1 | 2 |
| DBR1 | 0.604938272 | 0.836379091 | 1 | 2 |
| NUDT17 | 0.604938272 | 0.836379091 | 1 | 2 |
| GADL1 | 0.604938272 | 0.836379091 | 1 | 2 |
| PIGU | 0.604938272 | 0.836379091 | 1 | 2 |
| DNAJB9 | 0.604938272 | 0.836379091 | 1 | 2 |
| FZD5 | 0.604938272 | 0.836379091 | 1 | 2 |
| TOGARAM2 | 0.604938272 | 0.836379091 | 1 | 2 |
| FAM180B | 0.604938272 | 0.836379091 | 1 | 2 |
| FAM186B | 0.604938272 | 0.836379091 | 1 | 2 |
| ADCK2 | 0.604938272 | 0.836379091 | 1 | 2 |
| FUT1 | 0.604938272 | 0.836379091 | 1 | 2 |
| DNAJC4 | 0.604938272 | 0.836379091 | 1 | 2 |
| FUBP3 | 0.604938272 | 0.836379091 | 1 | 2 |
| ADAT1 | 0.604938272 | 0.836379091 | 1 | 2 |
| FAM189B | 0.604938272 | 0.836379091 | 1 | 2 |
| MRM2 | 0.604938272 | 0.836379091 | 1 | 2 |
| CYFIP2 | 0.604938272 | 0.836379091 | 1 | 2 |
| FAM196A | 0.604938272 | 0.836379091 | 1 | 2 |
| FSTL5 | 0.604938272 | 0.836379091 | 1 | 2 |
| FAM196B | 0.604938272 | 0.836379091 | 1 | 2 |
| FRZB | 0.604938272 | 0.836379091 | 1 | 2 |
| DMPK | 0.604938272 | 0.836379091 | 1 | 2 |
| NOTCH4 | 0.604938272 | 0.836379091 | 1 | 2 |
| FAM189A1 | 0.604938272 | 0.836379091 | 1 | 2 |
| FAM162B | 0.604938272 | 0.836379091 | 1 | 2 |
| GAL3ST3 | 0.604938272 | 0.836379091 | 1 | 2 |
| GALK1 | 0.604938272 | 0.836379091 | 1 | 2 |
| CRISPLD2 | 0.604938272 | 0.836379091 | 1 | 2 |
| GEN1 | 0.604938272 | 0.836379091 | 1 | 2 |
| FAM111A | 0.604938272 | 0.836379091 | 1 | 2 |
| TCAF1 | 0.604938272 | 0.836379091 | 1 | 2 |
| GEMIN4 | 0.604938272 | 0.836379091 | 1 | 2 |
| NUP98 | 0.604938272 | 0.836379091 | 1 | 2 |
| CRNKL1 | 0.604938272 | 0.836379091 | 1 | 2 |
| CSF2RA | 0.604938272 | 0.836379091 | 1 | 2 |
| DCP1B | 0.604938272 | 0.836379091 | 1 | 2 |
| GCG | 0.604938272 | 0.836379091 | 1 | 2 |
| C1QTNF12 | 0.604938272 | 0.836379091 | 1 | 2 |
| GBA2 | 0.604938272 | 0.836379091 | 1 | 2 |
| DOK3 | 0.604938272 | 0.836379091 | 1 | 2 |
| GAREM2 | 0.604938272 | 0.836379091 | 1 | 2 |
| FAM153A | 0.604938272 | 0.836379091 | 1 | 2 |
| ABRACL | 0.604938272 | 0.836379091 | 1 | 2 |
| NUP133 | 0.604938272 | 0.836379091 | 1 | 2 |
| FAM155B | 0.604938272 | 0.836379091 | 1 | 2 |
| NUFIP1 | 0.604938272 | 0.836379091 | 1 | 2 |
| GALNTL6 | 0.604938272 | 0.836379091 | 1 | 2 |
| DNLZ | 0.604938272 | 0.836379091 | 1 | 2 |
| GALNT12 | 0.604938272 | 0.836379091 | 1 | 2 |
| DNAJC6 | 0.604938272 | 0.836379091 | 1 | 2 |
| NUB1 | 0.604938272 | 0.836379091 | 1 | 2 |
| CRIP2 | 0.604938272 | 0.836379091 | 1 | 2 |
| FRMPD4 | 0.604938272 | 0.836379091 | 1 | 2 |
| CYP24A1 | 0.604938272 | 0.836379091 | 1 | 2 |
| FBLN1 | 0.604938272 | 0.836379091 | 1 | 2 |
| NPS | 0.604938272 | 0.836379091 | 1 | 2 |
| DARS | 0.604938272 | 0.836379091 | 1 | 2 |
| DBX2 | 0.604938272 | 0.836379091 | 1 | 2 |
| DCAF4 | 0.604938272 | 0.836379091 | 1 | 2 |
| ACOT4 | 0.604938272 | 0.836379091 | 1 | 2 |
| DCAF5 | 0.604938272 | 0.836379091 | 1 | 2 |
| NQO2 | 0.604938272 | 0.836379091 | 1 | 2 |
| NRG2 | 0.604938272 | 0.836379091 | 1 | 2 |
| ACTN1 | 0.604938272 | 0.836379091 | 1 | 2 |
| NPR1 | 0.604938272 | 0.836379091 | 1 | 2 |
| FBXO10 | 0.604938272 | 0.836379091 | 1 | 2 |
| ACRV1 | 0.604938272 | 0.836379091 | 1 | 2 |
| FBXO39 | 0.604938272 | 0.836379091 | 1 | 2 |
| DDX56 | 0.604938272 | 0.836379091 | 1 | 2 |
| NR1H2 | 0.604938272 | 0.836379091 | 1 | 2 |
| FBXW12 | 0.604938272 | 0.836379091 | 1 | 2 |
| FGA | 0.604938272 | 0.836379091 | 1 | 2 |
| FBXW8 | 0.604938272 | 0.836379091 | 1 | 2 |
| FFAR4 | 0.604938272 | 0.836379091 | 1 | 2 |
| DDX50 | 0.604938272 | 0.836379091 | 1 | 2 |
| NR4A2 | 0.604938272 | 0.836379091 | 1 | 2 |
| FBXO16 | 0.604938272 | 0.836379091 | 1 | 2 |
| DEPDC5 | 0.604938272 | 0.836379091 | 1 | 2 |
| FKBP7 | 0.604938272 | 0.836379091 | 1 | 2 |
| FLAD1 | 0.604938272 | 0.836379091 | 1 | 2 |
| FRMD1 | 0.604938272 | 0.836379091 | 1 | 2 |
| NTSR2 | 0.604938272 | 0.836379091 | 1 | 2 |
| NTSR1 | 0.604938272 | 0.836379091 | 1 | 2 |
| FPGS | 0.604938272 | 0.836379091 | 1 | 2 |
| FAM53A | 0.604938272 | 0.836379091 | 1 | 2 |
| FOXN4 | 0.604938272 | 0.836379091 | 1 | 2 |
| DHX8 | 0.604938272 | 0.836379091 | 1 | 2 |
| FOXN2 | 0.604938272 | 0.836379091 | 1 | 2 |
| FAM71C | 0.604938272 | 0.836379091 | 1 | 2 |
| FOXJ2 | 0.604938272 | 0.836379091 | 1 | 2 |
| DHX57 | 0.604938272 | 0.836379091 | 1 | 2 |
| MIGA1 | 0.604938272 | 0.836379091 | 1 | 2 |
| NPFFR2 | 0.604938272 | 0.836379091 | 1 | 2 |
| ACCSL | 0.604938272 | 0.836379091 | 1 | 2 |
| CYP4F8 | 0.604938272 | 0.836379091 | 1 | 2 |
| CYP4V2 | 0.604938272 | 0.836379091 | 1 | 2 |
| NSMCE1 | 0.604938272 | 0.836379091 | 1 | 2 |
| FAM91A1 | 0.604938272 | 0.836379091 | 1 | 2 |
| ADAM30 | 0.604938272 | 0.836379091 | 1 | 2 |
| FANCL | 0.604938272 | 0.836379091 | 1 | 2 |
| DAO | 0.604938272 | 0.836379091 | 1 | 2 |
| NPNT | 0.604938272 | 0.836379091 | 1 | 2 |
| FMNL1 | 0.604938272 | 0.836379091 | 1 | 2 |
| NUAK1 | 0.604938272 | 0.836379091 | 1 | 2 |
| FAM107B | 0.604938272 | 0.836379091 | 1 | 2 |
| FDPS | 0.604938272 | 0.836379091 | 1 | 2 |
| FAHD1 | 0.604938272 | 0.836379091 | 1 | 2 |
| ENKD1 | 0.604938272 | 0.836379091 | 1 | 2 |
| GPR153 | 0.604938272 | 0.836379091 | 1 | 2 |
| ENPEP | 0.604938272 | 0.836379091 | 1 | 2 |
| GPR142 | 0.604938272 | 0.836379091 | 1 | 2 |
| ENTPD7 | 0.604938272 | 0.836379091 | 1 | 2 |
| GPR139 | 0.604938272 | 0.836379091 | 1 | 2 |
| CNNM3 | 0.604938272 | 0.836379091 | 1 | 2 |
| ENTPD8 | 0.604938272 | 0.836379091 | 1 | 2 |
| NFE2L1 | 0.604938272 | 0.836379091 | 1 | 2 |
| NPPC | 0.604938272 | 0.836379091 | 1 | 2 |
| FAM105A | 0.604938272 | 0.836379091 | 1 | 2 |
| EPAS1 | 0.604938272 | 0.836379091 | 1 | 2 |
| GPKOW | 0.604938272 | 0.836379091 | 1 | 2 |
| OLAH | 0.604938272 | 0.836379091 | 1 | 2 |
| ABCA10 | 0.604938272 | 0.836379091 | 1 | 2 |
| CNTN5 | 0.604938272 | 0.836379091 | 1 | 2 |
| OGFR | 0.604938272 | 0.836379091 | 1 | 2 |
| EPB41L5 | 0.604938272 | 0.836379091 | 1 | 2 |
| COG1 | 0.604938272 | 0.836379091 | 1 | 2 |
| COG6 | 0.604938272 | 0.836379091 | 1 | 2 |
| COL14A1 | 0.604938272 | 0.836379091 | 1 | 2 |
| CMKLR1 | 0.604938272 | 0.836379091 | 1 | 2 |
| ADGRG1 | 0.604938272 | 0.836379091 | 1 | 2 |
| GPR62 | 0.604938272 | 0.836379091 | 1 | 2 |
| ENG | 0.604938272 | 0.836379091 | 1 | 2 |
| NEMF | 0.604938272 | 0.836379091 | 1 | 2 |
| UBASH3A | 0.604938272 | 0.836379091 | 1 | 2 |
| CLN3 | 0.604938272 | 0.836379091 | 1 | 2 |
| EIF5 | 0.604938272 | 0.836379091 | 1 | 2 |
| AGPAT2 | 0.604938272 | 0.836379091 | 1 | 2 |
| EHMT2 | 0.604938272 | 0.836379091 | 1 | 2 |
| CLN5 | 0.604938272 | 0.836379091 | 1 | 2 |
| EHBP1 | 0.604938272 | 0.836379091 | 1 | 2 |
| GPT | 0.604938272 | 0.836379091 | 1 | 2 |
| CLPX | 0.604938272 | 0.836379091 | 1 | 2 |
| EPB42 | 0.604938272 | 0.836379091 | 1 | 2 |
| NEURL2 | 0.604938272 | 0.836379091 | 1 | 2 |
| OPRL1 | 0.604938272 | 0.836379091 | 1 | 2 |
| CMA1 | 0.604938272 | 0.836379091 | 1 | 2 |
| AAAS | 0.604938272 | 0.836379091 | 1 | 2 |
| AACS | 0.604938272 | 0.836379091 | 1 | 2 |
| ELL | 0.604938272 | 0.836379091 | 1 | 2 |
| GPR84 | 0.604938272 | 0.836379091 | 1 | 2 |
| GPR78 | 0.604938272 | 0.836379091 | 1 | 2 |
| EMC7 | 0.604938272 | 0.836379091 | 1 | 2 |
| EFEMP1 | 0.604938272 | 0.836379091 | 1 | 2 |
| MMRN2 | 0.604938272 | 0.836379091 | 1 | 2 |
| GPRC5B | 0.604938272 | 0.836379091 | 1 | 2 |
| COL1A1 | 0.604938272 | 0.836379091 | 1 | 2 |
| AATK | 0.604938272 | 0.836379091 | 1 | 2 |
| AFMID | 0.604938272 | 0.836379091 | 1 | 2 |
| OBP2B | 0.604938272 | 0.836379091 | 1 | 2 |
| DYRK2 | 0.604938272 | 0.836379091 | 1 | 2 |
| GJD4 | 0.604938272 | 0.836379091 | 1 | 2 |
| ESYT3 | 0.604938272 | 0.836379091 | 1 | 2 |
| DUSP15 | 0.604938272 | 0.836379091 | 1 | 2 |
| AEN | 0.604938272 | 0.836379091 | 1 | 2 |
| GMEB2 | 0.604938272 | 0.836379091 | 1 | 2 |
| DTX3L | 0.604938272 | 0.836379091 | 1 | 2 |
| DTX1 | 0.604938272 | 0.836379091 | 1 | 2 |
| GLB1 | 0.604938272 | 0.836379091 | 1 | 2 |
| CORO7-PAM16 | 0.604938272 | 0.836379091 | 1 | 2 |
| DTD1 | 0.604938272 | 0.836379091 | 1 | 2 |
| NIPBL | 0.604938272 | 0.836379091 | 1 | 2 |
| EXOSC6 | 0.604938272 | 0.836379091 | 1 | 2 |
| DSG3 | 0.604938272 | 0.836379091 | 1 | 2 |
| INTS11 | 0.604938272 | 0.836379091 | 1 | 2 |
| DSEL | 0.604938272 | 0.836379091 | 1 | 2 |
| COLGALT1 | 0.604938272 | 0.836379091 | 1 | 2 |
| COL4A6 | 0.604938272 | 0.836379091 | 1 | 2 |
| DPY19L4 | 0.604938272 | 0.836379091 | 1 | 2 |
| ECSIT | 0.604938272 | 0.836379091 | 1 | 2 |
| ECHDC2 | 0.604938272 | 0.836379091 | 1 | 2 |
| DPYSL4 | 0.604938272 | 0.836379091 | 1 | 2 |
| EPHB6 | 0.604938272 | 0.836379091 | 1 | 2 |
| DQX1 | 0.604938272 | 0.836379091 | 1 | 2 |
| AFAP1 | 0.604938272 | 0.836379091 | 1 | 2 |
| EPX | 0.604938272 | 0.836379091 | 1 | 2 |
| GLG1 | 0.604938272 | 0.836379091 | 1 | 2 |
| DNAAF4 | 0.604938272 | 0.836379091 | 1 | 2 |
| ABCC1 | 0.604938272 | 0.836379091 | 1 | 2 |
| CPT1B | 0.604938272 | 0.836379091 | 1 | 2 |
| ERI1 | 0.604938272 | 0.836379091 | 1 | 2 |
| GIMAP2 | 0.604938272 | 0.836379091 | 1 | 2 |
| VCX3B | 0.768472906 | 0.836693932 | 24 | 35 |
| SYNRG | 0.6 | 0.841410814 | 2 | 4 |
| NUP210L | 0.6 | 0.841410814 | 2 | 4 |
| MPHOSPH9 | 0.6 | 0.841410814 | 2 | 4 |
| ST18 | 0.6 | 0.841410814 | 2 | 4 |
| FBLN2 | 0.6 | 0.841410814 | 2 | 4 |
| SLC4A9 | 0.6 | 0.841410814 | 2 | 4 |
| MROH6 | 0.6 | 0.841410814 | 2 | 4 |
| WWC2 | 0.6 | 0.841410814 | 2 | 4 |
| MT1E | 0.6 | 0.841410814 | 2 | 4 |
| DOPEY2 | 0.6 | 0.841410814 | 2 | 4 |
| MFSD3 | 0.6 | 0.841410814 | 2 | 4 |
| PRSS55 | 0.6 | 0.841410814 | 2 | 4 |
| ARFGAP3 | 0.6 | 0.841410814 | 2 | 4 |
| C1orf127 | 0.6 | 0.841410814 | 2 | 4 |
| PHLPP2 | 0.6 | 0.841410814 | 2 | 4 |
| ZFPM1 | 0.6 | 0.841410814 | 2 | 4 |
| FBRSL1 | 0.6 | 0.841410814 | 2 | 4 |
| PRKDC | 0.6 | 0.841410814 | 2 | 4 |
| PIK3C2G | 0.6 | 0.841410814 | 2 | 4 |
| ABCA2 | 0.6 | 0.841410814 | 2 | 4 |
| MARVELD3 | 0.6 | 0.841410814 | 2 | 4 |
| BTRC | 0.6 | 0.841410814 | 2 | 4 |
| MBD3L3 | 0.6 | 0.841410814 | 2 | 4 |
| MBD5 | 0.6 | 0.841410814 | 2 | 4 |
| ERBIN | 0.6 | 0.841410814 | 2 | 4 |
| VWA2 | 0.6 | 0.841410814 | 2 | 4 |
| TAS1R3 | 0.6 | 0.841410814 | 2 | 4 |
| BOP1 | 0.6 | 0.841410814 | 2 | 4 |
| MAP3K9 | 0.6 | 0.841410814 | 2 | 4 |
| SPG11 | 0.6 | 0.841410814 | 2 | 4 |
| AP3D1 | 0.6 | 0.841410814 | 2 | 4 |
| TEPSIN | 0.6 | 0.841410814 | 2 | 4 |
| ZNF827 | 0.6 | 0.841410814 | 2 | 4 |
| VPS53 | 0.6 | 0.841410814 | 2 | 4 |
| LILRB5 | 0.6 | 0.841410814 | 2 | 4 |
| ZC3H4 | 0.6 | 0.841410814 | 2 | 4 |
| ATRNL1 | 0.6 | 0.841410814 | 2 | 4 |
| DHRS7 | 0.6 | 0.841410814 | 2 | 4 |
| PCSK5 | 0.6 | 0.841410814 | 2 | 4 |
| DGKK | 0.6 | 0.841410814 | 2 | 4 |
| MAST3 | 0.6 | 0.841410814 | 2 | 4 |
| DGKD | 0.6 | 0.841410814 | 2 | 4 |
| LEPR | 0.6 | 0.841410814 | 2 | 4 |
| ZCCHC11 | 0.6 | 0.841410814 | 2 | 4 |
| EFCAB6 | 0.6 | 0.841410814 | 2 | 4 |
| PCNX1 | 0.6 | 0.841410814 | 2 | 4 |
| MAGI2 | 0.6 | 0.841410814 | 2 | 4 |
| DHX29 | 0.6 | 0.841410814 | 2 | 4 |
| DOK7 | 0.6 | 0.841410814 | 2 | 4 |
| id75979 | 0.6 | 0.841410814 | 2 | 4 |
| TTLL10 | 0.6 | 0.841410814 | 2 | 4 |
| CEPT1 | 0.6 | 0.841410814 | 2 | 4 |
| AKAP1 | 0.6 | 0.841410814 | 2 | 4 |
| ITGA6 | 0.6 | 0.841410814 | 2 | 4 |
| CT45A3 | 0.6 | 0.841410814 | 2 | 4 |
| ITGA7 | 0.6 | 0.841410814 | 2 | 4 |
| GPR17 | 0.6 | 0.841410814 | 2 | 4 |
| ITGAM | 0.6 | 0.841410814 | 2 | 4 |
| REL | 0.6 | 0.841410814 | 2 | 4 |
| CC2D2A | 0.6 | 0.841410814 | 2 | 4 |
| JAG2 | 0.6 | 0.841410814 | 2 | 4 |
| NLRP5 | 0.6 | 0.841410814 | 2 | 4 |
| SLC22A20 | 0.6 | 0.841410814 | 2 | 4 |
| TTLL12 | 0.6 | 0.841410814 | 2 | 4 |
| N4BP2 | 0.6 | 0.841410814 | 2 | 4 |
| AGFG2 | 0.6 | 0.841410814 | 2 | 4 |
| HEATR6 | 0.6 | 0.841410814 | 2 | 4 |
| NOL11 | 0.6 | 0.841410814 | 2 | 4 |
| RBM15 | 0.6 | 0.841410814 | 2 | 4 |
| MYPN | 0.6 | 0.841410814 | 2 | 4 |
| ZNF432 | 0.6 | 0.841410814 | 2 | 4 |
| MYOF | 0.6 | 0.841410814 | 2 | 4 |
| MYO5C | 0.6 | 0.841410814 | 2 | 4 |
| ADAMTS2 | 0.6 | 0.841410814 | 2 | 4 |
| GDPD3 | 0.6 | 0.841410814 | 2 | 4 |
| CCDC158 | 0.6 | 0.841410814 | 2 | 4 |
| RFX5 | 0.6 | 0.841410814 | 2 | 4 |
| RGL3 | 0.6 | 0.841410814 | 2 | 4 |
| BEX3 | 0.6 | 0.841410814 | 2 | 4 |
| COL6A1 | 0.6 | 0.841410814 | 2 | 4 |
| AFAP1L1 | 0.6 | 0.841410814 | 2 | 4 |
| SGO2 | 0.6 | 0.841410814 | 2 | 4 |
| P2RY6 | 0.6 | 0.841410814 | 2 | 4 |
| TMC7 | 0.6 | 0.841410814 | 2 | 4 |
| TMC5 | 0.6 | 0.841410814 | 2 | 4 |
| TPRN | 0.6 | 0.841410814 | 2 | 4 |
| GLIS3 | 0.6 | 0.841410814 | 2 | 4 |
| TMC2 | 0.6 | 0.841410814 | 2 | 4 |
| TPO | 0.6 | 0.841410814 | 2 | 4 |
| SCAF1 | 0.6 | 0.841410814 | 2 | 4 |
| RUSC1 | 0.6 | 0.841410814 | 2 | 4 |
| NBEAL2 | 0.6 | 0.841410814 | 2 | 4 |
| TRIM68 | 0.6 | 0.841410814 | 2 | 4 |
| RUSC2 | 0.6 | 0.841410814 | 2 | 4 |
| ADGRF3 | 0.6 | 0.841410814 | 2 | 4 |
| TLR1 | 0.6 | 0.841410814 | 2 | 4 |
| HIVEP1 | 0.6 | 0.841410814 | 2 | 4 |
| CCDC74A | 0.6 | 0.841410814 | 2 | 4 |
| GIGYF1 | 0.6 | 0.841410814 | 2 | 4 |
| CEP95 | 0.6 | 0.841410814 | 2 | 4 |
| TLE2 | 0.6 | 0.841410814 | 2 | 4 |
| CCDC18 | 0.6 | 0.841410814 | 2 | 4 |
| TRIM65 | 0.6 | 0.841410814 | 2 | 4 |
| KCNU1 | 0.6 | 0.841410814 | 2 | 4 |
| SFRP5 | 0.6 | 0.841410814 | 2 | 4 |
| SCN7A | 0.6 | 0.841410814 | 2 | 4 |
| CXCL10 | 0.6 | 0.841410814 | 2 | 4 |
| DAAM2 | 0.6 | 0.841410814 | 2 | 4 |
| PTPN23 | 0.6 | 0.841410814 | 2 | 4 |
| GPRIN1 | 0.6 | 0.841410814 | 2 | 4 |
| UBR2 | 0.6 | 0.841410814 | 2 | 4 |
| PYGB | 0.6 | 0.841410814 | 2 | 4 |
| SCN10A | 0.6 | 0.841410814 | 2 | 4 |
| RABEP1 | 0.6 | 0.841410814 | 2 | 4 |
| HAP1 | 0.6 | 0.841410814 | 2 | 4 |
| UNC80 | 0.6 | 0.841410814 | 2 | 4 |
| C8A | 0.6 | 0.841410814 | 2 | 4 |
| KIF26B | 0.6 | 0.841410814 | 2 | 4 |
| UGT2B7 | 0.6 | 0.841410814 | 2 | 4 |
| MYH9 | 0.6 | 0.841410814 | 2 | 4 |
| GTF3C5 | 0.6 | 0.841410814 | 2 | 4 |
| CABIN1 | 0.6 | 0.841410814 | 2 | 4 |
| RACGAP1 | 0.6 | 0.841410814 | 2 | 4 |
| C9orf84 | 0.6 | 0.841410814 | 2 | 4 |
| UBASH3A | 0.6 | 0.841410814 | 2 | 4 |
| DFFB | 0.6 | 0.841410814 | 2 | 4 |
| ZNF341 | 0.6 | 0.841410814 | 2 | 4 |
| CAGE1 | 0.6 | 0.841410814 | 2 | 4 |
| DACT1 | 0.6 | 0.841410814 | 2 | 4 |
| GRSF1 | 0.6 | 0.841410814 | 2 | 4 |
| GRM2 | 0.6 | 0.841410814 | 2 | 4 |
| HEATR1 | 0.6 | 0.841410814 | 2 | 4 |
| CYP4F2 | 0.6 | 0.841410814 | 2 | 4 |
| CDH23 | 0.684444444 | 0.842217974 | 7 | 12 |
| HSPG2 | 0.684444444 | 0.842217974 | 7 | 12 |
| ZNF721 | 0.684444444 | 0.842217974 | 7 | 12 |
| MKI67 | 0.656565657 | 0.843817614 | 5 | 9 |
| LAMA1 | 0.656565657 | 0.843817614 | 5 | 9 |
| PKD1L2 | 0.656565657 | 0.843817614 | 5 | 9 |
| LOC728728 | 0.698630137 | 0.845807912 | 9 | 15 |
| ZNF778 | 0.603092784 | 0.851098316 | 78 | 97 |
| GIGYF2 | 0.759444203 | 0.856309299 | 33 | 47 |
| SLC3A2 | 0.755615454 | 0.856753296 | 29 | 42 |
| PPP4R4 | 0.594936709 | 0.857606914 | 3 | 6 |
| SPDYE3 | 0.594936709 | 0.857606914 | 3 | 6 |
| MAP1B | 0.594936709 | 0.857606914 | 3 | 6 |
| CDC42BPB | 0.594936709 | 0.857606914 | 3 | 6 |
| SPIRE2 | 0.594936709 | 0.857606914 | 3 | 6 |
| CNTROB | 0.594936709 | 0.857606914 | 3 | 6 |
| COL6A6 | 0.594936709 | 0.857606914 | 3 | 6 |
| DENND4C | 0.594936709 | 0.857606914 | 3 | 6 |
| PCDHB7 | 0.594936709 | 0.857606914 | 3 | 6 |
| FLG2 | 0.594936709 | 0.857606914 | 3 | 6 |
| SORL1 | 0.594936709 | 0.857606914 | 3 | 6 |
| COL5A3 | 0.594936709 | 0.857606914 | 3 | 6 |
| MYBPC2 | 0.594936709 | 0.857606914 | 3 | 6 |
| ANKRD31 | 0.594936709 | 0.857606914 | 3 | 6 |
| HELZ | 0.594936709 | 0.857606914 | 3 | 6 |
| OSMR | 0.594936709 | 0.857606914 | 3 | 6 |
| MED16 | 0.594936709 | 0.857606914 | 3 | 6 |
| MYO3B | 0.594936709 | 0.857606914 | 3 | 6 |
| TARBP1 | 0.594936709 | 0.857606914 | 3 | 6 |
| DST | 0.664092664 | 0.865219533 | 8 | 14 |
| PKHD1L1 | 0.638755981 | 0.865992689 | 6 | 11 |
| FAT3 | 0.638755981 | 0.865992689 | 6 | 11 |
| PRH2 | 0.638755981 | 0.865992689 | 6 | 11 |
| SYNE1 | 0.685714286 | 0.873614395 | 12 | 20 |
| SSPO | 0.685714286 | 0.873614395 | 12 | 20 |
| RANBP2 | 0.58974359 | 0.874659296 | 4 | 8 |
| CROCC | 0.58974359 | 0.874659296 | 4 | 8 |
| KRTAP10-6 | 0.58974359 | 0.874659296 | 4 | 8 |
| GSE1 | 0.58974359 | 0.874659296 | 4 | 8 |
| MGAM | 0.58974359 | 0.874659296 | 4 | 8 |
| KIF26A | 0.58974359 | 0.874659296 | 4 | 8 |
| CSPG4 | 0.58974359 | 0.874659296 | 4 | 8 |
| MADCAM1 | 0.58974359 | 0.874659296 | 4 | 8 |
| LOC101928498 | 0.58974359 | 0.874659296 | 4 | 8 |
| ZNF77 | 0.711764706 | 0.886010047 | 22 | 34 |
| KRTAP4-6 | 0.713307241 | 0.886141564 | 54 | 73 |
| REXO1L1P | 0.713307241 | 0.886141564 | 54 | 73 |
| OPN1MW | 0.660489251 | 0.88769025 | 11 | 19 |
| PRAMEF17 | 0.584415584 | 0.890594165 | 5 | 10 |
| LRP2 | 0.584415584 | 0.890594165 | 5 | 10 |
| MYO18A | 0.584415584 | 0.890594165 | 5 | 10 |
| ZNF527 | 0.66798419 | 0.892297359 | 13 | 22 |
| FZD8 | 0.612612613 | 0.90137119 | 8 | 15 |
| SEC13 | 0.475 | 0.902585665 | 2 | 5 |
| CGB8 | 0.475 | 0.902585665 | 2 | 5 |
| SETD2 | 0.475 | 0.902585665 | 2 | 5 |
| CEP112 | 0.475 | 0.902585665 | 2 | 5 |
| SERTAD2 | 0.475 | 0.902585665 | 2 | 5 |
| GON4L | 0.475 | 0.902585665 | 2 | 5 |
| ASH1L | 0.475 | 0.902585665 | 2 | 5 |
| SCAF11 | 0.475 | 0.902585665 | 2 | 5 |
| HAGH | 0.475 | 0.902585665 | 2 | 5 |
| PTPN18 | 0.475 | 0.902585665 | 2 | 5 |
| PLCB1 | 0.475 | 0.902585665 | 2 | 5 |
| C10orf90 | 0.475 | 0.902585665 | 2 | 5 |
| TEX15 | 0.475 | 0.902585665 | 2 | 5 |
| CACNA1G | 0.475 | 0.902585665 | 2 | 5 |
| KIAA1614 | 0.475 | 0.902585665 | 2 | 5 |
| DHX34 | 0.475 | 0.902585665 | 2 | 5 |
| MAP3K21 | 0.475 | 0.902585665 | 2 | 5 |
| KIF1A | 0.475 | 0.902585665 | 2 | 5 |
| FMNL3 | 0.475 | 0.902585665 | 2 | 5 |
| SMPD4 | 0.475 | 0.902585665 | 2 | 5 |
| VCAN | 0.475 | 0.902585665 | 2 | 5 |
| CLIP1 | 0.475 | 0.902585665 | 2 | 5 |
| TBC1D31 | 0.475 | 0.902585665 | 2 | 5 |
| ULK1 | 0.475 | 0.902585665 | 2 | 5 |
| C6orf132 | 0.475 | 0.902585665 | 2 | 5 |
| URB2 | 0.475 | 0.902585665 | 2 | 5 |
| PSKH2 | 0.475 | 0.902585665 | 2 | 5 |
| TBXAS1 | 0.475 | 0.902585665 | 2 | 5 |
| DDX11 | 0.475 | 0.902585665 | 2 | 5 |
| PZP | 0.475 | 0.902585665 | 2 | 5 |
| COL1A2 | 0.475 | 0.902585665 | 2 | 5 |
| KNL1 | 0.475 | 0.902585665 | 2 | 5 |
| BRCA2 | 0.475 | 0.902585665 | 2 | 5 |
| MICALL2 | 0.475 | 0.902585665 | 2 | 5 |
| CDCA2 | 0.475 | 0.902585665 | 2 | 5 |
| CDC42BPG | 0.475 | 0.902585665 | 2 | 5 |
| MAN2B1 | 0.475 | 0.902585665 | 2 | 5 |
| SH3PXD2A | 0.475 | 0.902585665 | 2 | 5 |
| SH3RF2 | 0.475 | 0.902585665 | 2 | 5 |
| PLEKHH1 | 0.475 | 0.902585665 | 2 | 5 |
| SHANK1 | 0.475 | 0.902585665 | 2 | 5 |
| CASKIN2 | 0.475 | 0.902585665 | 2 | 5 |
| SPATA20 | 0.475 | 0.902585665 | 2 | 5 |
| B4GALNT3 | 0.475 | 0.902585665 | 2 | 5 |
| TRPV3 | 0.475 | 0.902585665 | 2 | 5 |
| PLXND1 | 0.475 | 0.902585665 | 2 | 5 |
| TCIRG1 | 0.475 | 0.902585665 | 2 | 5 |
| REXO1 | 0.475 | 0.902585665 | 2 | 5 |
| POLRMT | 0.475 | 0.902585665 | 2 | 5 |
| POR | 0.475 | 0.902585665 | 2 | 5 |
| ITIH2 | 0.475 | 0.902585665 | 2 | 5 |
| F5 | 0.475 | 0.902585665 | 2 | 5 |
| SEMA3G | 0.475 | 0.902585665 | 2 | 5 |
| CAMTA2 | 0.475 | 0.902585665 | 2 | 5 |
| ALDH16A1 | 0.475 | 0.902585665 | 2 | 5 |
| AGBL1 | 0.475 | 0.902585665 | 2 | 5 |
| ZNF804B | 0.475 | 0.902585665 | 2 | 5 |
| ANO10 | 0.475 | 0.902585665 | 2 | 5 |
| NINL | 0.475 | 0.902585665 | 2 | 5 |
| MYLK | 0.475 | 0.902585665 | 2 | 5 |
| AKAP13 | 0.475 | 0.902585665 | 2 | 5 |
| ACOT11 | 0.475 | 0.902585665 | 2 | 5 |
| ABCC3 | 0.475 | 0.902585665 | 2 | 5 |
| ACAD11 | 0.475 | 0.902585665 | 2 | 5 |
| ZFYVE28 | 0.475 | 0.902585665 | 2 | 5 |
| ZNF419 | 0.475 | 0.902585665 | 2 | 5 |
| NRCAM | 0.475 | 0.902585665 | 2 | 5 |
| MOGS | 0.475 | 0.902585665 | 2 | 5 |
| ADAMTS17 | 0.475 | 0.902585665 | 2 | 5 |
| AMZ1 | 0.475 | 0.902585665 | 2 | 5 |
| ZNF814 | 0.475 | 0.902585665 | 2 | 5 |
| NOD2 | 0.475 | 0.902585665 | 2 | 5 |
| ADCY2 | 0.475 | 0.902585665 | 2 | 5 |
| ACAN | 0.578947368 | 0.904983221 | 6 | 12 |
| UBN2 | 0.504520796 | 0.907384315 | 3 | 7 |
| COL27A1 | 0.504520796 | 0.907384315 | 3 | 7 |
| ZNF462 | 0.504520796 | 0.907384315 | 3 | 7 |
| CEL | 0.504520796 | 0.907384315 | 3 | 7 |
| ZZEF1 | 0.504520796 | 0.907384315 | 3 | 7 |
| BRD9 | 0.504520796 | 0.907384315 | 3 | 7 |
| TCAF2 | 0.504520796 | 0.907384315 | 3 | 7 |
| CCDC171 | 0.504520796 | 0.907384315 | 3 | 7 |
| MUM1 | 0.399176955 | 0.911333921 | 1 | 3 |
| PCDHB15 | 0.399176955 | 0.911333921 | 1 | 3 |
| FMNL2 | 0.399176955 | 0.911333921 | 1 | 3 |
| KRT13 | 0.399176955 | 0.911333921 | 1 | 3 |
| ADAM15 | 0.399176955 | 0.911333921 | 1 | 3 |
| KREMEN2 | 0.399176955 | 0.911333921 | 1 | 3 |
| UNC45B | 0.399176955 | 0.911333921 | 1 | 3 |
| MIS18BP1 | 0.399176955 | 0.911333921 | 1 | 3 |
| C6orf222 | 0.399176955 | 0.911333921 | 1 | 3 |
| KRTAP10-9 | 0.399176955 | 0.911333921 | 1 | 3 |
| PSTPIP1 | 0.399176955 | 0.911333921 | 1 | 3 |
| ACSM1 | 0.399176955 | 0.911333921 | 1 | 3 |
| KRTAP10-4 | 0.399176955 | 0.911333921 | 1 | 3 |
| OR4K13 | 0.399176955 | 0.911333921 | 1 | 3 |
| FGF20 | 0.399176955 | 0.911333921 | 1 | 3 |
| UNC5A | 0.399176955 | 0.911333921 | 1 | 3 |
| KRT71 | 0.399176955 | 0.911333921 | 1 | 3 |
| UPF2 | 0.399176955 | 0.911333921 | 1 | 3 |
| PTPDC1 | 0.399176955 | 0.911333921 | 1 | 3 |
| UIMC1 | 0.399176955 | 0.911333921 | 1 | 3 |
| KRT31 | 0.399176955 | 0.911333921 | 1 | 3 |
| PCDHB9 | 0.399176955 | 0.911333921 | 1 | 3 |
| KRT24 | 0.399176955 | 0.911333921 | 1 | 3 |
| KRT7 | 0.399176955 | 0.911333921 | 1 | 3 |
| MUSK | 0.399176955 | 0.911333921 | 1 | 3 |
| ZNF347 | 0.399176955 | 0.911333921 | 1 | 3 |
| TMPRSS5 | 0.399176955 | 0.911333921 | 1 | 3 |
| UACA | 0.399176955 | 0.911333921 | 1 | 3 |
| WASHC5 | 0.399176955 | 0.911333921 | 1 | 3 |
| KIAA0368 | 0.399176955 | 0.911333921 | 1 | 3 |
| FURIN | 0.399176955 | 0.911333921 | 1 | 3 |
| CACNG1 | 0.399176955 | 0.911333921 | 1 | 3 |
| ANKEF1 | 0.399176955 | 0.911333921 | 1 | 3 |
| FTSJ3 | 0.399176955 | 0.911333921 | 1 | 3 |
| CACNB1 | 0.399176955 | 0.911333921 | 1 | 3 |
| RABL6 | 0.399176955 | 0.911333921 | 1 | 3 |
| RABGAP1L | 0.399176955 | 0.911333921 | 1 | 3 |
| CYP11B2 | 0.399176955 | 0.911333921 | 1 | 3 |
| MFSD4B | 0.399176955 | 0.911333921 | 1 | 3 |
| RAB44 | 0.399176955 | 0.911333921 | 1 | 3 |
| CACNA1E | 0.399176955 | 0.911333921 | 1 | 3 |
| FMO1 | 0.399176955 | 0.911333921 | 1 | 3 |
| PCDH10 | 0.399176955 | 0.911333921 | 1 | 3 |
| FRMPD1 | 0.399176955 | 0.911333921 | 1 | 3 |
| KIF23 | 0.399176955 | 0.911333921 | 1 | 3 |
| CYP2C18 | 0.399176955 | 0.911333921 | 1 | 3 |
| CYP2C9 | 0.399176955 | 0.911333921 | 1 | 3 |
| ANKRD35 | 0.399176955 | 0.911333921 | 1 | 3 |
| MYCBPAP | 0.399176955 | 0.911333921 | 1 | 3 |
| OR51F2 | 0.399176955 | 0.911333921 | 1 | 3 |
| UGGT2 | 0.399176955 | 0.911333921 | 1 | 3 |
| NPHP4 | 0.399176955 | 0.911333921 | 1 | 3 |
| D2HGDH | 0.399176955 | 0.911333921 | 1 | 3 |
| UGT1A9 | 0.399176955 | 0.911333921 | 1 | 3 |
| UGT2A3 | 0.399176955 | 0.911333921 | 1 | 3 |
| IZUMO1R | 0.399176955 | 0.911333921 | 1 | 3 |
| ADAM28 | 0.399176955 | 0.911333921 | 1 | 3 |
| RAB26 | 0.399176955 | 0.911333921 | 1 | 3 |
| C3orf67 | 0.399176955 | 0.911333921 | 1 | 3 |
| ANXA11 | 0.399176955 | 0.911333921 | 1 | 3 |
| GLMP | 0.399176955 | 0.911333921 | 1 | 3 |
| MINDY4 | 0.399176955 | 0.911333921 | 1 | 3 |
| BTBD11 | 0.399176955 | 0.911333921 | 1 | 3 |
| BTBD18 | 0.399176955 | 0.911333921 | 1 | 3 |
| PPP1R13L | 0.399176955 | 0.911333921 | 1 | 3 |
| TAF2 | 0.399176955 | 0.911333921 | 1 | 3 |
| PPP1R21 | 0.399176955 | 0.911333921 | 1 | 3 |
| LOC101929220 | 0.399176955 | 0.911333921 | 1 | 3 |
| DNAAF2 | 0.399176955 | 0.911333921 | 1 | 3 |
| ZNF862 | 0.399176955 | 0.911333921 | 1 | 3 |
| LNX1 | 0.399176955 | 0.911333921 | 1 | 3 |
| ZNF850 | 0.399176955 | 0.911333921 | 1 | 3 |
| AP4B1 | 0.399176955 | 0.911333921 | 1 | 3 |
| TAS2R19 | 0.399176955 | 0.911333921 | 1 | 3 |
| NTN5 | 0.399176955 | 0.911333921 | 1 | 3 |
| DMRTB1 | 0.399176955 | 0.911333921 | 1 | 3 |
| BRPF3 | 0.399176955 | 0.911333921 | 1 | 3 |
| SNX21 | 0.399176955 | 0.911333921 | 1 | 3 |
| ZFP36L2 | 0.399176955 | 0.911333921 | 1 | 3 |
| DOT1L | 0.399176955 | 0.911333921 | 1 | 3 |
| ZNHIT3 | 0.399176955 | 0.911333921 | 1 | 3 |
| SOWAHB | 0.399176955 | 0.911333921 | 1 | 3 |
| MRPL21 | 0.399176955 | 0.911333921 | 1 | 3 |
| SORT1 | 0.399176955 | 0.911333921 | 1 | 3 |
| LPIN1 | 0.399176955 | 0.911333921 | 1 | 3 |
| BMS1 | 0.399176955 | 0.911333921 | 1 | 3 |
| LOXL4 | 0.399176955 | 0.911333921 | 1 | 3 |
| NUP107 | 0.399176955 | 0.911333921 | 1 | 3 |
| PDHX | 0.399176955 | 0.911333921 | 1 | 3 |
| LOC729159 | 0.399176955 | 0.911333921 | 1 | 3 |
| BRAT1 | 0.399176955 | 0.911333921 | 1 | 3 |
| PDGFA | 0.399176955 | 0.911333921 | 1 | 3 |
| WDR55 | 0.399176955 | 0.911333921 | 1 | 3 |
| PPIAL4G | 0.399176955 | 0.911333921 | 1 | 3 |
| LMOD1 | 0.399176955 | 0.911333921 | 1 | 3 |
| KRTAP5-8 | 0.399176955 | 0.911333921 | 1 | 3 |
| AP2A1 | 0.399176955 | 0.911333921 | 1 | 3 |
| VPS37C | 0.399176955 | 0.911333921 | 1 | 3 |
| FBXO40 | 0.399176955 | 0.911333921 | 1 | 3 |
| SLC7A2 | 0.399176955 | 0.911333921 | 1 | 3 |
| LARP7 | 0.399176955 | 0.911333921 | 1 | 3 |
| PRRT3 | 0.399176955 | 0.911333921 | 1 | 3 |
| LAMC1 | 0.399176955 | 0.911333921 | 1 | 3 |
| MTHFD1L | 0.399176955 | 0.911333921 | 1 | 3 |
| USPL1 | 0.399176955 | 0.911333921 | 1 | 3 |
| SLC6A7 | 0.399176955 | 0.911333921 | 1 | 3 |
| OR4C6 | 0.399176955 | 0.911333921 | 1 | 3 |
| SLC25A13 | 0.399176955 | 0.911333921 | 1 | 3 |
| ACSBG1 | 0.399176955 | 0.911333921 | 1 | 3 |
| SLC6A12 | 0.399176955 | 0.911333921 | 1 | 3 |
| USP48 | 0.399176955 | 0.911333921 | 1 | 3 |
| TC2N | 0.399176955 | 0.911333921 | 1 | 3 |
| LACTBL1 | 0.399176955 | 0.911333921 | 1 | 3 |
| TBKBP1 | 0.399176955 | 0.911333921 | 1 | 3 |
| TBC1D4 | 0.399176955 | 0.911333921 | 1 | 3 |
| ZNF813 | 0.399176955 | 0.911333921 | 1 | 3 |
| VPS37A | 0.399176955 | 0.911333921 | 1 | 3 |
| SLTM | 0.399176955 | 0.911333921 | 1 | 3 |
| PCSK6 | 0.399176955 | 0.911333921 | 1 | 3 |
| DGAT1 | 0.399176955 | 0.911333921 | 1 | 3 |
| PRKCSH | 0.399176955 | 0.911333921 | 1 | 3 |
| OR2Y1 | 0.399176955 | 0.911333921 | 1 | 3 |
| NRIP1 | 0.399176955 | 0.911333921 | 1 | 3 |
| MSLN | 0.399176955 | 0.911333921 | 1 | 3 |
| AOC2 | 0.399176955 | 0.911333921 | 1 | 3 |
| VAV3 | 0.399176955 | 0.911333921 | 1 | 3 |
| FBXL13 | 0.399176955 | 0.911333921 | 1 | 3 |
| TRIR | 0.399176955 | 0.911333921 | 1 | 3 |
| VASN | 0.399176955 | 0.911333921 | 1 | 3 |
| SLCO2B1 | 0.399176955 | 0.911333921 | 1 | 3 |
| DENND2C | 0.399176955 | 0.911333921 | 1 | 3 |
| DHX33 | 0.399176955 | 0.911333921 | 1 | 3 |
| SLC24A5 | 0.399176955 | 0.911333921 | 1 | 3 |
| PARPBP | 0.399176955 | 0.911333921 | 1 | 3 |
| MYO1F | 0.399176955 | 0.911333921 | 1 | 3 |
| IGF1R | 0.399176955 | 0.911333921 | 1 | 3 |
| OR6N2 | 0.399176955 | 0.911333921 | 1 | 3 |
| CDC25B | 0.399176955 | 0.911333921 | 1 | 3 |
| IFIH1 | 0.399176955 | 0.911333921 | 1 | 3 |
| TREH | 0.399176955 | 0.911333921 | 1 | 3 |
| GPC4 | 0.399176955 | 0.911333921 | 1 | 3 |
| TMEM132E | 0.399176955 | 0.911333921 | 1 | 3 |
| OR7C2 | 0.399176955 | 0.911333921 | 1 | 3 |
| HTR4 | 0.399176955 | 0.911333921 | 1 | 3 |
| ADGRG5 | 0.399176955 | 0.911333921 | 1 | 3 |
| NFATC4 | 0.399176955 | 0.911333921 | 1 | 3 |
| GPR146 | 0.399176955 | 0.911333921 | 1 | 3 |
| NDE1 | 0.399176955 | 0.911333921 | 1 | 3 |
| TRAPPC12 | 0.399176955 | 0.911333921 | 1 | 3 |
| TMEM168 | 0.399176955 | 0.911333921 | 1 | 3 |
| ZNF669 | 0.399176955 | 0.911333921 | 1 | 3 |
| NFKBIZ | 0.399176955 | 0.911333921 | 1 | 3 |
| TRIM31 | 0.399176955 | 0.911333921 | 1 | 3 |
| NAV1 | 0.399176955 | 0.911333921 | 1 | 3 |
| TRIML1 | 0.399176955 | 0.911333921 | 1 | 3 |
| IGSF8 | 0.399176955 | 0.911333921 | 1 | 3 |
| ZNF69 | 0.399176955 | 0.911333921 | 1 | 3 |
| IGSF21 | 0.399176955 | 0.911333921 | 1 | 3 |
| PADI1 | 0.399176955 | 0.911333921 | 1 | 3 |
| TRIM59 | 0.399176955 | 0.911333921 | 1 | 3 |
| CD1A | 0.399176955 | 0.911333921 | 1 | 3 |
| SGK223 | 0.399176955 | 0.911333921 | 1 | 3 |
| TRIM48 | 0.399176955 | 0.911333921 | 1 | 3 |
| COL5A2 | 0.399176955 | 0.911333921 | 1 | 3 |
| GNB5 | 0.399176955 | 0.911333921 | 1 | 3 |
| RNF214 | 0.399176955 | 0.911333921 | 1 | 3 |
| AFM | 0.399176955 | 0.911333921 | 1 | 3 |
| ALDOB | 0.399176955 | 0.911333921 | 1 | 3 |
| NFASC | 0.399176955 | 0.911333921 | 1 | 3 |
| CCRL2 | 0.399176955 | 0.911333921 | 1 | 3 |
| ZNF556 | 0.399176955 | 0.911333921 | 1 | 3 |
| AGL | 0.399176955 | 0.911333921 | 1 | 3 |
| CEP97 | 0.399176955 | 0.911333921 | 1 | 3 |
| CLDN17 | 0.399176955 | 0.911333921 | 1 | 3 |
| AHI1 | 0.399176955 | 0.911333921 | 1 | 3 |
| SEC14L4 | 0.399176955 | 0.911333921 | 1 | 3 |
| OSBPL7 | 0.399176955 | 0.911333921 | 1 | 3 |
| AK7 | 0.399176955 | 0.911333921 | 1 | 3 |
| SACM1L | 0.399176955 | 0.911333921 | 1 | 3 |
| GTF2E2 | 0.399176955 | 0.911333921 | 1 | 3 |
| SDCCAG3 | 0.399176955 | 0.911333921 | 1 | 3 |
| OTOA | 0.399176955 | 0.911333921 | 1 | 3 |
| TNFRSF18 | 0.399176955 | 0.911333921 | 1 | 3 |
| HDAC7 | 0.399176955 | 0.911333921 | 1 | 3 |
| HCN3 | 0.399176955 | 0.911333921 | 1 | 3 |
| TNFRSF11A | 0.399176955 | 0.911333921 | 1 | 3 |
| HECTD3 | 0.399176955 | 0.911333921 | 1 | 3 |
| SELENOO | 0.399176955 | 0.911333921 | 1 | 3 |
| CEP170 | 0.399176955 | 0.911333921 | 1 | 3 |
| OTUD7B | 0.399176955 | 0.911333921 | 1 | 3 |
| RSF1 | 0.399176955 | 0.911333921 | 1 | 3 |
| CEACAM18 | 0.399176955 | 0.911333921 | 1 | 3 |
| ADGRG3 | 0.399176955 | 0.911333921 | 1 | 3 |
| CLSTN1 | 0.399176955 | 0.911333921 | 1 | 3 |
| RSPH4A | 0.399176955 | 0.911333921 | 1 | 3 |
| HOXA13 | 0.399176955 | 0.911333921 | 1 | 3 |
| RSPH14 | 0.399176955 | 0.911333921 | 1 | 3 |
| CEBPZ | 0.399176955 | 0.911333921 | 1 | 3 |
| HDHD5 | 0.399176955 | 0.911333921 | 1 | 3 |
| TPH1 | 0.399176955 | 0.911333921 | 1 | 3 |
| NEO1 | 0.399176955 | 0.911333921 | 1 | 3 |
| CEP104 | 0.399176955 | 0.911333921 | 1 | 3 |
| NELL2 | 0.399176955 | 0.911333921 | 1 | 3 |
| GREB1 | 0.399176955 | 0.911333921 | 1 | 3 |
| TP53AIP1 | 0.399176955 | 0.911333921 | 1 | 3 |
| AKR7A2 | 0.399176955 | 0.911333921 | 1 | 3 |
| RAPGEF4 | 0.399176955 | 0.911333921 | 1 | 3 |
| ZNF7 | 0.399176955 | 0.911333921 | 1 | 3 |
| GLE1 | 0.399176955 | 0.911333921 | 1 | 3 |
| JSRP1 | 0.399176955 | 0.911333921 | 1 | 3 |
| CASP8 | 0.399176955 | 0.911333921 | 1 | 3 |
| JMJD7-PLA2G4B | 0.399176955 | 0.911333921 | 1 | 3 |
| TTF1 | 0.399176955 | 0.911333921 | 1 | 3 |
| RCBTB2 | 0.399176955 | 0.911333921 | 1 | 3 |
| TTC7A | 0.399176955 | 0.911333921 | 1 | 3 |
| CTNNAL1 | 0.399176955 | 0.911333921 | 1 | 3 |
| JAK3 | 0.399176955 | 0.911333921 | 1 | 3 |
| SLC13A1 | 0.399176955 | 0.911333921 | 1 | 3 |
| ITSN1 | 0.399176955 | 0.911333921 | 1 | 3 |
| CBX8 | 0.399176955 | 0.911333921 | 1 | 3 |
| ITLN2 | 0.399176955 | 0.911333921 | 1 | 3 |
| CCDC102A | 0.399176955 | 0.911333921 | 1 | 3 |
| GCNT2 | 0.399176955 | 0.911333921 | 1 | 3 |
| RCN3 | 0.399176955 | 0.911333921 | 1 | 3 |
| KANK4 | 0.399176955 | 0.911333921 | 1 | 3 |
| AMPD2 | 0.399176955 | 0.911333921 | 1 | 3 |
| TTYH2 | 0.399176955 | 0.911333921 | 1 | 3 |
| CAMK4 | 0.399176955 | 0.911333921 | 1 | 3 |
| GAB2 | 0.399176955 | 0.911333921 | 1 | 3 |
| RASEF | 0.399176955 | 0.911333921 | 1 | 3 |
| RASGRF1 | 0.399176955 | 0.911333921 | 1 | 3 |
| PARP4 | 0.399176955 | 0.911333921 | 1 | 3 |
| RASIP1 | 0.399176955 | 0.911333921 | 1 | 3 |
| TGM3 | 0.399176955 | 0.911333921 | 1 | 3 |
| CAPN1 | 0.399176955 | 0.911333921 | 1 | 3 |
| ADCY9 | 0.399176955 | 0.911333921 | 1 | 3 |
| SLC22A12 | 0.399176955 | 0.911333921 | 1 | 3 |
| TGM7 | 0.399176955 | 0.911333921 | 1 | 3 |
| MYRF | 0.399176955 | 0.911333921 | 1 | 3 |
| PKMYT1 | 0.399176955 | 0.911333921 | 1 | 3 |
| CARS | 0.399176955 | 0.911333921 | 1 | 3 |
| KARS | 0.399176955 | 0.911333921 | 1 | 3 |
| ITGB3 | 0.399176955 | 0.911333921 | 1 | 3 |
| RMDN1 | 0.399176955 | 0.911333921 | 1 | 3 |
| TTC21B | 0.399176955 | 0.911333921 | 1 | 3 |
| FAM234A | 0.399176955 | 0.911333921 | 1 | 3 |
| RHD | 0.399176955 | 0.911333921 | 1 | 3 |
| GIMAP8 | 0.399176955 | 0.911333921 | 1 | 3 |
| NISCH | 0.399176955 | 0.911333921 | 1 | 3 |
| IL4I1 | 0.399176955 | 0.911333921 | 1 | 3 |
| CPSF1 | 0.399176955 | 0.911333921 | 1 | 3 |
| CCDC88B | 0.399176955 | 0.911333921 | 1 | 3 |
| RHCG | 0.399176955 | 0.911333921 | 1 | 3 |
| SHISA5 | 0.399176955 | 0.911333921 | 1 | 3 |
| RIN3 | 0.399176955 | 0.911333921 | 1 | 3 |
| CCKBR | 0.399176955 | 0.911333921 | 1 | 3 |
| ZNF703 | 0.399176955 | 0.911333921 | 1 | 3 |
| CYP2A6 | 0.399176955 | 0.911333921 | 1 | 3 |
| ADRM1 | 0.399176955 | 0.911333921 | 1 | 3 |
| IL17F | 0.399176955 | 0.911333921 | 1 | 3 |
| TRPA1 | 0.399176955 | 0.911333921 | 1 | 3 |
| CRB1 | 0.399176955 | 0.911333921 | 1 | 3 |
| CREBBP | 0.399176955 | 0.911333921 | 1 | 3 |
| SIGLEC12 | 0.399176955 | 0.911333921 | 1 | 3 |
| NLRP2 | 0.399176955 | 0.911333921 | 1 | 3 |
| NADK | 0.399176955 | 0.911333921 | 1 | 3 |
| CCDC144A | 0.399176955 | 0.911333921 | 1 | 3 |
| ALG9 | 0.399176955 | 0.911333921 | 1 | 3 |
| ZNF493 | 0.399176955 | 0.911333921 | 1 | 3 |
| GEMIN5 | 0.399176955 | 0.911333921 | 1 | 3 |
| NLRC3 | 0.399176955 | 0.911333921 | 1 | 3 |
| RGL4 | 0.399176955 | 0.911333921 | 1 | 3 |
| IPO5 | 0.399176955 | 0.911333921 | 1 | 3 |
| NLN | 0.399176955 | 0.911333921 | 1 | 3 |
| SIPA1 | 0.399176955 | 0.911333921 | 1 | 3 |
| GGA1 | 0.399176955 | 0.911333921 | 1 | 3 |
| GGA2 | 0.399176955 | 0.911333921 | 1 | 3 |
| GGNBP2 | 0.399176955 | 0.911333921 | 1 | 3 |
| CCDC39 | 0.399176955 | 0.911333921 | 1 | 3 |
| ITGA1 | 0.399176955 | 0.911333921 | 1 | 3 |
| SYNGR2 | 0.399176955 | 0.911333921 | 1 | 3 |
| ANKRD65 | 0.399176955 | 0.911333921 | 1 | 3 |
| MAD1L1 | 0.399176955 | 0.911333921 | 1 | 3 |
| ARPP21 | 0.399176955 | 0.911333921 | 1 | 3 |
| PLD4 | 0.399176955 | 0.911333921 | 1 | 3 |
| ZBTB8B | 0.399176955 | 0.911333921 | 1 | 3 |
| ODF3B | 0.399176955 | 0.911333921 | 1 | 3 |
| ABCB1 | 0.399176955 | 0.911333921 | 1 | 3 |
| ZCCHC6 | 0.399176955 | 0.911333921 | 1 | 3 |
| MFSD9 | 0.399176955 | 0.911333921 | 1 | 3 |
| MPP5 | 0.399176955 | 0.911333921 | 1 | 3 |
| DPM1 | 0.399176955 | 0.911333921 | 1 | 3 |
| MMP27 | 0.399176955 | 0.911333921 | 1 | 3 |
| MAN2A1 | 0.399176955 | 0.911333921 | 1 | 3 |
| MMP9 | 0.399176955 | 0.911333921 | 1 | 3 |
| STIM1 | 0.399176955 | 0.911333921 | 1 | 3 |
| PLIN1 | 0.399176955 | 0.911333921 | 1 | 3 |
| PGK2 | 0.399176955 | 0.911333921 | 1 | 3 |
| ESCO1 | 0.399176955 | 0.911333921 | 1 | 3 |
| ATP11A | 0.399176955 | 0.911333921 | 1 | 3 |
| LRWD1 | 0.399176955 | 0.911333921 | 1 | 3 |
| EIF2AK3 | 0.399176955 | 0.911333921 | 1 | 3 |
| PIGG | 0.399176955 | 0.911333921 | 1 | 3 |
| STRIP2 | 0.399176955 | 0.911333921 | 1 | 3 |
| DPP9 | 0.399176955 | 0.911333921 | 1 | 3 |
| DPP10 | 0.399176955 | 0.911333921 | 1 | 3 |
| ARMC9 | 0.399176955 | 0.911333921 | 1 | 3 |
| AARS2 | 0.399176955 | 0.911333921 | 1 | 3 |
| ZNF16 | 0.399176955 | 0.911333921 | 1 | 3 |
| ARHGAP29 | 0.399176955 | 0.911333921 | 1 | 3 |
| DPYD | 0.399176955 | 0.911333921 | 1 | 3 |
| PKP2 | 0.399176955 | 0.911333921 | 1 | 3 |
| MANEAL | 0.399176955 | 0.911333921 | 1 | 3 |
| PLK5 | 0.399176955 | 0.911333921 | 1 | 3 |
| BCCIP | 0.399176955 | 0.911333921 | 1 | 3 |
| MICU3 | 0.399176955 | 0.911333921 | 1 | 3 |
| STARD13 | 0.399176955 | 0.911333921 | 1 | 3 |
[truncated: 133,076 more chars]
