# Supplementary material for: Identification of potential candidate genes and pathways in atrioventricular nodal reentry tachycardia by whole‐exome sequencing
Source: Clin Transl Med. 2020 Apr 30;10(1):238–57. doi: 10.1002/ctm2.25 (PMC7240861; doi:10.1002/ctm2.25)
Supplement: Supplementary file 15 — Supporting Information S14 [file CTM2-10-238-s015.doc]

**S11: Rare variant information in 5 Candidate genes**

| **Gene**  **name** | **Variant**  **type** | **Functional** | **Hgv. c** | **Hgv. p** | **KEGG**  **EAS AF** | **ExAC**  **EAS AF** | **GnomAD**  **Exome AF** | **Cases**  **(n)** | **Controls**  **(n)** |
| --- | --- | --- | --- | --- | --- | --- | --- | --- | --- |
| CFTR | SNP | missense_variant | c.1251C>A | p.Asn417Lys | 0 | 0.009 | 5.94E-03 | 66 | 49 |
|  |  |  | c.1265C>T | p.Ser422Phe | 0 | 0.001 | 3.12E-04 | 27 | 28 |
|  |  |  | c.1312A>G | p.Thr438Ala | 0 | 0.002 | 1.88E-04 | 15 | 24 |
|  |  |  | c.1392G>T | p.Lys464Asn | 0 | 0 | 0 | 2 | 2 |
|  |  |  | c.1407G>T | p.Met469Ile | 0 | 0.000 | 0 | 0 | 1 |
|  |  |  | c.2042A>T | p.Glu681Val | 0.001 | 0.002 | 1.31E-04 | 0 | 1 |
|  |  |  | c.220C>T | p.Arg74Trp | 0 | 0.001 | 1.42E-03 | 0 | 1 |
|  |  |  | c.2563G>A | p.Val855Ile | 0 | 0.001 | 1.54E-04 | 0 | 1 |
|  |  |  | c.2684G>A | p.Ser895Asn | 0.003 | 0.005 | 3.37E-04 | 0 | 2 |
|  |  |  | c.3263A>G | p.Asn1088Ser | 0 | 0 | 0 | 1 | 0 |
|  |  |  | c.374T>C | p.Ile125Thr | 0.008 | 0.008 | 6.88E-04 | 1 | 1 |
|  |  |  | c.4157G>T | p.Arg1386Ile | 0 | 0 | 0 | 0 | 1 |
|  |  |  | c.509G>A | p.Arg170His | 0 | 0 | 5.18E-04 | 1 | 0 |
|  |  |  | c.890G>A | p.Arg297Gln | 0 | 0 | 5.62E-04 | 1 | 0 |
|  |  | stop_gained | c.1657C>T | p.Arg553* | 0 | 0 | 7.33E-05 | 1 | 0 |
| NOS1 | InDel | frameshift_variant | c.3557-1_3569dupACACACACACACAC | p.Leu1191fs | 0 | 0 | 0 | 11 | 12 |
|  |  |  | c.3557-3_3569dupACACACACACACACAC | p.Leu1191fs | 0 | 0 | 0 | 25 | 18 |
|  |  |  | c.3568_3569dupAC | p.Leu1191fs | 0 | 0 | 0 | 7 | 3 |
|  | SNP | missense_variant | c.102G>C | p.Glu34Asp | 0 | 0 | 0 | 0 | 1 |
|  |  |  | c.1631A>G | p.Glu544Gly | 0 | 0 | 0 | 1 | 0 |
|  |  |  | c.2579C>T | p.Pro860Leu | 0 | 0 | 0 | 1 | 0 |
|  |  | splice_acceptor_variant | c.3557-1A>T | . | 0 | 0 | 3.27E-05 | 0 | 1 |
| PRKAG2 | SNP | missense_variant | c.130G>A | p.Ala44Thr | 0.005 | 0.003 | 0 | 1 | 0 |
|  |  |  | c.250C>T | p.Arg84Trp | 0 | 0 | 1.46E-04 | 1 | 0 |
|  |  |  | c.83A>C | p.His28Pro | 0.008 | 0 | 3.84E-04 | 2 | 0 |
| RYR2 | SNP | missense_variant | c.7076G>A | p.Arg2359Gln | 0 | 0.001 | 3.25E-05 | 1 | 0 |
|  |  |  | c.3143A>G | p.Asp1048Gly | 0 | 0 | 9.25E-06 | 1 | 0 |
|  |  |  | c.6040G>T | p.Asp2014Tyr | 0 | 0 | 0.00E+00 | 1 | 0 |
|  |  |  | c.5774T>C | p.Ile1925Thr | 0 | 0 | 3.26E-05 | 1 | 0 |
|  |  |  | c.11352T>G | p.Ile3784Met | 0 | 0 | 0.00E+00 | 1 | 0 |
|  |  |  | c.13050A>C | p.Leu4350Phe | 0 | 0 | 0.00E+00 | 0 | 1 |
|  |  |  | c.5923A>G | p.Met1975Val | 0 | 0.000 | 6.96E-05 | 0 | 1 |
|  |  |  | c.5570C>T | p.Pro1857Leu | 0 | 0.000 | 6.11E-05 | 1 | 0 |
|  |  |  | c.6092C>T | p.Ser2031Phe | 0 | 0.000 | 1.22E-05 | 1 | 0 |
|  |  |  | c.3683C>A | p.Thr1228Asn | 0 | 0 | 0.00E+00 | 1 | 1 |
| SCN1A | SNP | missense_variant | c.135C>G | p.Asp45Glu | 0.001 | 0.001 | 5.28E-05 | 1 | 0 |
|  |  |  | c.2141T>G | p.Met714Arg | 0 | 0 | 0 | 1 | 0 |
|  |  |  | c.3053G>A | p.Arg1018Lys | 0 | 0.000 | 2.52E-05 | 1 | 0 |
|  |  |  | c.3176A>T | p.Asp1059Val | 0 | 0.000 | 4.48E-05 | 1 | 0 |

*Note: KEGG, Kyoto Encyclopedia of Genes and Genomes；ExAC, Exome Aggregation Consortium; EAS, East Asian; GnomAD, Genome Aggregation Database; AF, allele frequency.*
