# Supplementary material for: Identification of potential candidate genes and pathways in atrioventricular nodal reentry tachycardia by whole‐exome sequencing
Source: Clin Transl Med. 2020 Apr 30;10(1):238–57. doi: 10.1002/ctm2.25 (PMC7240861; doi:10.1002/ctm2.25)
Supplement: Supplementary file 17 — Supporting Information S16 [file CTM2-10-238-s017.docx]

SUPPLEMENT CONTENT

**Content of Tables**

**S1 Reference target genes list**

**S2 Rare variants of reference genes (MAF<0.001)**

**S3 Rare variants of reference genes (MAF<0.01)**

**S4 SNPs in GWAS analysis (P<10-E6)**

**S5 Gene-based burden analysis (MAF< 0.01)**

**S6 Gene-based burden analysis (MAF< 0.001)**

**S7 Reactome-kobas pathway enrichment (MAF <0.01)**

**S8**  **Reactome-kobas pathway enrichment (MAF <0.001)**

**S9 Rare variant information in candidate gene from burden analysis (MAF<0.01)**

**S10** **Rare variant information in candidate gene from burden analysis (MAF<0.001)**

**S11**  **Rare variant information in 5 candidate genes**

**S12 Validation of candidate genes in UK Biobank**

**S13 Enrichment analysis for phenotype category in UK Biobank**

**Content of Figures**

**S14 Average sequencing cover of exons**

**S15 Hardy-Weinberg equilibrium, dendrogram and quantile- quantile plot in GWAS**

**S16 Quantile-quantile and Manhatton plots in pathway enrichment(MAF<0.01 or MAF<0.001)**
